# Systematic microRNAome profiling reveals the roles of microRNAs in milk protein metabolism and quality: insights on low-quality forage utilization

**DOI:** 10.1038/srep21194

**Published:** 2016-02-17

**Authors:** Diming Wang, Guanxiang Liang, Bing Wang, Huizeng Sun, Jianxin Liu, Le Luo Guan

**Affiliations:** 1 Institute of Dairy Sciences, College of Animal Sciences, Zhejiang University, Hangzhou, P R, China; 2 Department of Agricultural, Food and Nutritional Science, University of Alberta, Edmonton, Canada

## Abstract

In this study, we investigated the molecular regulatory mechanisms of milk protein production in dairy cows by studying the miRNAomes of five key metabolic tissues involved in protein synthesis and metabolism from dairy cows fed high- and low-quality diets. In total, 340, 338, 337, 330, and 328 miRNAs were expressed in the rumen, duodenum, jejunum, liver, and mammary gland tissues, respectively. Some miRNAs were highly correlated with feed and nitrogen efficiency, with target genes involved in transportation and phosphorylation of amino acid (AA). Additionally, low-quality forage diets (corn stover and rice straw) influenced the expression of feed and nitrogen efficiency-associated miRNAs such as miR-99b in rumen, miR-2336 in duodenum, miR-652 in jejunum, miR-1 in liver, and miR-181a in mammary gland. Ruminal miR-21-3p and liver miR-2285f were predicted to regulate AA transportation by targeting *ATP1A*2 and *SLC7A*8, respectively. Furthermore, bovine-specific miRNAs regulated the proliferation and morphology of rumen epithelium, as well as the metabolism of liver lipids and branched-chain AAs, revealing bovine-specific mechanisms. Our results suggest that miRNAs expressed in these five tissues play roles in regulating transportation of AA for downstream milk production, which is an important mechanism that may be associated with low milk protein under low-quality forage feed.

Enhancing the protein content in milk is essential for improvement of milk quality in developing countries, especially under a lack of high-quality forage. Milk protein has been considered one of the best protein sources for humans^1^, especially whey protein, whose amino acids (AA) and associated compounds can provide nutrition and bioactive substrates for humans^2^. With the improvement of Chinese quality of life, total milk production has continuously increased, leading to the rapid development of the Chinese dairy industry, which has increased 12.8% annually on average since 2000^3^,^4^,^5^. However, in China, milk production per cow is relatively low compared with that in the developed counties such as the USA^6^. The poor milk quality has been a huge concern due to its low nutrient content, especially protein^7^.

Nitrogen (N) metabolism can directly impact milk protein yield and quality. N efficiency, the ratio of milk protein yield to crude protein intake, has been considered a key regulator for milk protein synthesis^8^. When N efficiency is low, a large proportion of N will be excreted through urine, reducing the N flow to the mammary gland for milk protein synthesis^9^. Therefore, N efficiency can also be estimated by measuring the content of milk urea N (MUN), with a lower MUN content representing higher N efficiency^10^. Feed efficiency, the ratio of milk production to dry matter intake, is also an important trait that indicates the efficiency of grocery nutrients transferred to the milk of dairy cows^11^. Therefore, lower feed and N efficiency can not only decrease the nutrients enriched in the milk but also increase the energy waste and negative environmental impact, such as methane emission and N enrichment in the urine and fecal matter^12^. Therefore, improvement of feed and N efficiency is vital for the dairy industry to meet the challenges in food security and food quality that China is facing.

The dairy industry in China has heavily relied on imported alfalfa hay (AL) to maintain a high production yield in recent years. Alfalfa hay, as high-quality forage, can enhance the feed efficiency and N efficiency in lactating dairy cows^13^,^14^, however, China does not produce sufficient AL locally. Therefore, the enhancement of the utilization of local forage sources such as corn stover (CS) or rice straw (RS) has been one of the main tasks for China’s dairy industry. More than 2.0×10^11^kg of CS are produced annually in China, most of which is wasted, and very little is used as feed sources for livestock^15^. Some have speculated that cows undergo physiological changes when fed low-quality CS, due to the reduced milk productivity and quality, compared to those fed high-quality AL^13^,^14^. This suggests that a fundamental understanding of the molecular mechanisms of milk production and milk protein synthesis processes under low-quality forages is essential to develop strategies to enhance the potentials of CS as feed for dairy cows.

Therefore, we aimed to investigate the systematic molecular regulatory mechanisms of milk protein production in dairy cows by studying the microRNAomes of five key metabolic tissues, the rumen, duodenum, jejunum, liver and mammary gland, and their roles in dairy feed efficiency and N efficiency because miRNAs are non-coding RNAs that regulate gene expression^16^. We hypothesized that different miRNAs are involved in regulating feed efficiency and N efficiency in these tissues, which can also be influenced by the different forage sources.

## Materials and Methods

The experimental protocol was approved by the Animal Care Committee, Zhejiang University, Hangzhou, P. R. China, and all procedures were conducted in accordance with the approved protocol.

### Animal study and sample collection

A total of 18 cows were fed three diets with different forage sources: AL (n = 6), RS (n = 6), or CS (n = 6), for 90 days. The details of ingredients and nutritional composition of diets have been described in our previous study^14^. Briefly, diets were isonitrogenous, with a forage-to-concentrate ratio of 45:55 [dry matter (DM) basis] and contained identical concentrate mixtures and 15% corn silage, with different forage sources (on a DM basis): 23% alfalfa hay and 7% Chinese wild rye hay (AL), 30% corn stover (CS), and 30% rice straw (RS). All animals were slaughtered as described in a previous study^17^. Samples of five tissues were collected immediately after slaughter following the methods described by Ash & Baird^18^ for rumen epithelium, Burrin *et al.*^19^ for duodenum epithelium and jejunum epithelium, and Laffitte *et al.*^20^ for liver and mammary gland. Tissue samples were immediately frozen in liquid nitrogen and then moved to −80 °C until they were used for RNA extraction.

### Total RNA extraction and miRNA-Seq library construction

Total RNA was isolated from ~100 mg tissue using the mirVana miRNA Isolation Kit (Ambion®, Life Technologies, Carlsbad, CA, USA) following manufacturer’s protocol. The RNA concentration was determined using a Qubit 2.0 Fluorometer (Life Technologies). The quality of RNA was evaluated using the Agilent 2100 Bioanalyzer (Agilent Technologies, CA, USA) with the RNA 6000 NanoLabchip Kit. RNA samples with the RNA integrity number (RIN) more than 7.0 were used for small RNA library construction and quantitative real-time PCR validation.

Equal amounts (1000 ng) of total RNA from each sample were used to construct small RNA libraries using the TruSeq Small RNA Sample Preparation kit (Illumina, San Diego, CA, USA). PCR amplification was performed for 11 cycles. Overall, 90 small RNA libraries were constructed and pooled in equal amounts for gel purification and sequencing, as described in a previous study^21^. All libraries were sequenced at Génome Québec (Montréal, Canada) using the HiSeq 2000 system (Illumina) to generate 50 bp single reads.

### Sequencing data analysis

Small RNA sequencing data were analyzed using the methods as outlined by Liang *et al.*^22^. Briefly, the low-quality reads were discarded from the raw data with CASAVA 1.8 based on chastity, while the reads with acceptable quality were subjected to 3′ adaptor sequence trimming. The sequences with size from 18 to 30 nt were then mapped to the ncRNA sequences (Rfam) to remove non-miRNA sequences (including tRNA, snoRNA, rRNA, and other non-coding RNAs), and the known and novel miRNAs were determined using miRDeep2^23^. Briefly, the filtered sequences were then aligned against the corresponding known miRNA precursor sequences (miRBase release version 21) with the module of quantifier.pl in miRDeep2 (default parameters) to quantify known miRNAs.

The conservation of known miRNAs was described based on the TargetScan definitions for “highly conserved”, “conserved”, “poorly conserved”, and “bovine-specific”^24^. A highly conserved miRNA is conserved across most vertebrates; a conserved miRNA is conserved across most mammals but usually not beyond placental mammals; a poorly conserved miRNA not present in the above three groups; and a bovine-specific miRNA is only identified in bovine species.

### Identification of differentially expressed miRNAs

The expression of known miRNAs in each library was normalized to counts per million reads (CPM) as follows: CPM = (miRNA reads number/total mapped reads number per library) × 1,000,000. The miRNAs with CPM > 1 in at least 50% of the samples were defined to be expressed miRNAs in each tissue. Tissue-specific miRNAs were defined as miRNAs uniquely expressed in one tissue. A miRNA expressed in all 5 tissues was defined as a commonly expressed miRNA, and miRNAs that were expressed significantly higher or lower in one tissue than any others (P < 0.05, log_2_ (fold change (FC)) >1 or <−1, ANOVA) were defined as tissue-dependent differentially expressed (DE) miRNAs. Two-way ANOVA (considering diet and tissue as main effects) and the PROC GLM procedure in SAS (SAS Institute, 2000) were applied to identify tissue-dependent DE miRNAs^25^. The diet effect on miRNAs expression was determined by a pair-wise comparison (AL vs CS, AL vs RS, and CS vs RS) using Student’s t-test with significance indicated at P < 0.05 and log_2_ (FC) >1 or <−1.

### Correlation analysis

Under each diet, the possible relationships between miRNA expression (CPM) in each tissue and phenotypic traits [feed efficiency (milk yield/DM intake, kg/kg), N efficiency (milk protein yield/crude protein intake (DM basis), kg/kg) and MUN content (mg/dL, an important indicator for N efficiency)] were identified by Pearson’s correlation by using R software. The significant correlations were declared at P < 0.05, R > 0.81 (positive correlation) or R < −0.81 (negative correlation). The miRNAs positively associated with feed and N efficiency (R > 0.81, P < 0.05) and negatively associated with MUN (R < −0.81, P < 0.05) were considered as positive for dairy performance. These miRNAs and those negatively associated with feed and N efficiency (R < −0.81, P < 0.05) and positively associated with MUN (R > 0.81, P < 0.05) were subjected to further functional analysis. Correlation between the expression of miRNAs and the expression of their predicted targeted were conducted with Pearson’s correlation with R < −0.81 and P < 0.05 as criteria of significantly targeting.

### miRNA targets prediction and functional analysis

Target genes for selected miRNAs were predicted by using TargetScan Release 6.0 (http://www.targetscan.org/) and miRanda (http://www.microrna.org/microrna). The target genes that were predicted by both TargetScan (default parameters) and miRanda (Total score ≥ 145, Total energy ≤ −10) for each miRNA were further analyzed through IPA (Ingenuity Systems, www.ingenuity.com). The multiple testing corrected *P* value calculated by Benjamini-Hochberg method (FDR) was used to determine the significance of the predicted function in IPA. A threshold of FDR < 0.05 and enriched gene number ≥ 2 were applied to enrich significant biological functions of each miRNA.

### Validation of selected miRNA expression results using stem-loop RT-qPCR

The TAQMAN miRNA assay (Applied Biosystems, Carlsbad, CA) was used to validate miRNA expression detected by RNA-Seq following the methods described in a previous study^22^. Briefly, cDNAs were reverse-transcribed from 10 ng total RNA using specific miRNART primers (Applied Biosystems), and DNA was amplified using a 20× TAQMANmiRNA assay (Applied Biosystems). A StepOnePlus™ Real-Time PCR System (Applied Biosystems) was used to detect the fluorescence signal. The calculation of relative expression of selected miRNAs were conducted as following equation: ΔCt_target miRNA_ = Ct_target miRNA_ – Ct_U6_, with U6 snRNA as internal control^22^. One-way ANOVA was used to compare the differences among groups, and P < 0.05 was considered significant.

## Results

### miRNAome profiling of five bovine metabolic tissues

**Figure 1:**
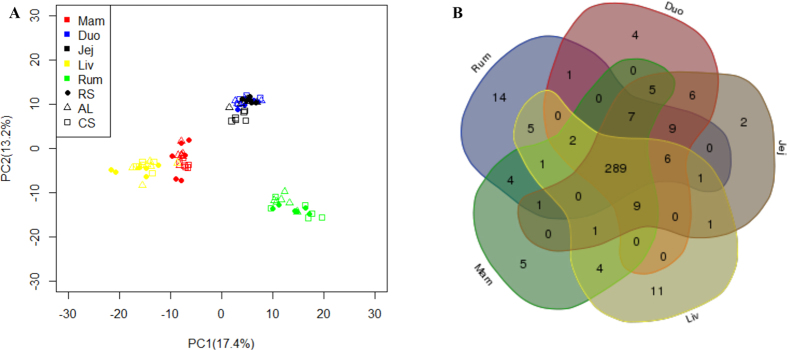
Basic microRNAs(miRNAs) profiles in the rumen (Rum), duodenum (Duo), jejunum (Jej), liver (Liv), and mammary gland (Mam). (**A**) PCA analysis of miRNA transcriptome profile among the 5 tissues and their variation among different diets (AL: alfalfa hay; CS: corn stover; RS: rice straw). *Note*: n = 18 for each tissue, n = 30 for each diet. (**B**) Commonly and specific known miRNAs identified in the Rum, Duo, Jej, Liv, and Mam.

The miRNA profiles were generated by sequencing 90 small RNA libraries prepared from rumen epithelium, duodenum epithelium, jejunum epithelium, liver, and mammary gland tissues collected from eighteen mid-lactating cows (164 ± 25 days in milk) fed AL (n = 6), RS (n = 6) or CS diet (n = 6) for 90 days. In total, 546 million out of 606 million reads (average 6.73 M per tissue) were mapped to the known miRNAs database (miRBase release version 21), resulting in 340, 338, 337, 330 and 328 known miRNAs and 32, 37, 37, 26, 36 novel miRNAs identified in rumen, duodenum, jejunum, liver, and mammary gland, respectively (Table 1). In total, 388 known miRNAs were expressed in these five tissues. The principal component analysis (PCA) showed clear separations in the miRNA profiles between any two tissues except between duodenum and jejunum. In addition, no clear separations were observed among the three diets (Fig. 1A).

### Tissue commonly and specifically expressed miRNAs

**Figure 2:**
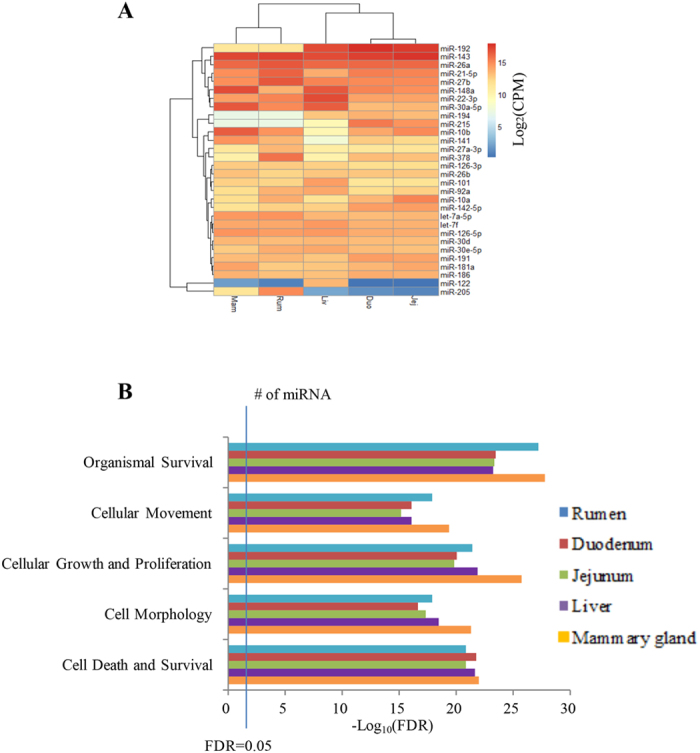
(**A**) Expression of top 20 commonly expressed microRNAs (miRNAs) in rumen, duodenum, jejunum, liver and mammary gland. *Note:* CPM = read counts per million of mapped reads. (**B**) Predominant function categories targeted by top 20 commonly expressed miRNAs in rumen, duodenum, jejunum, liver and mammary gland tissues. *Note:* -log_10_(FDR) values indicate the relevance of the function, with bigger values suggesting higher relevance.

In total, 289 miRNAs were commonly expressed in all five tissues (Fig. 1B). Among the top 20 most highly expressed miRNAs in the five tissues, miR-143 was highly expressed (~16%) in all tissues (Table 1), while the expression of some miRNAs varied between tissues, such as miR-122 and miR-205 (Fig. 2A). The predicted functions of the top 20 commonly expressed miRNAs included cell death and survival, cell morphology, cellular growth and proliferation, cellular movement and organismal survival (Fig. 2B and Table S1).

**Figure 3:**
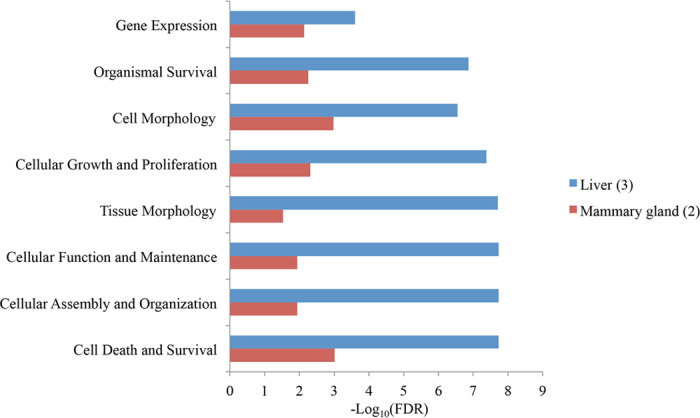
Predominant function categories targeted by tissue-specific microRNAs (miRNAs) in liver (n = 3) and mammary gland (n = 2). *Note:* -log_10_(FDR) values indicate the relevance of the function, with bigger values suggesting higher relevance, and n indicates the number of tissue-specific miRNAs included.

Tissue-specific miRNAs were defined as miRNAs uniquely expressed in one specific tissue (Table S2). In total, 14, 4, 2, 12 and 5 tissue-specific miRNAs were detected in the rumen, duodenum, jejunum, liver and mammary gland, respectively. Only 3 miRNAs (miR-122, -2415-3p, and -2483-3p) in liver and 2 (miR-196b and -2284j) in mammary gland were highly expressed (CPM > 5, Table S2), and the functional analysis showed that these 5 miRNAs were related to cell death and survival, cell morphology and cellular growth and proliferation (Fig. 3).

### Tissue-dependent differentially expressed miRNAs

**Figure 4:**
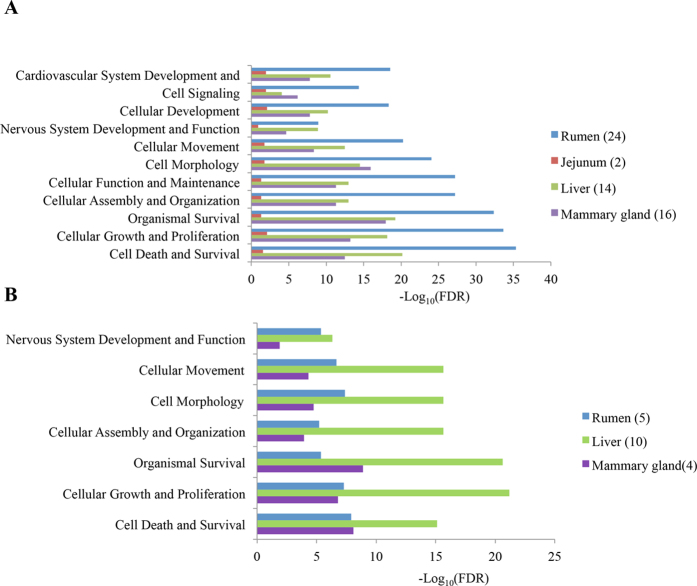
Predominant function categories targeted by differentially expressed microRNAs (miRNAs) in rumen, duodenum, jejunum, liver and mammary gland (**A**) miRNAs up-regulated in rumen (n = 24),jejunum (n=2), liver (n=14), and mammary gland (n=16); (**B**) miRNAs down-regulated in rumen (n = 5), liver (n = 10), and mammary gland (n = 4). *Note:* -log_10_(FDR) values indicate the relevance of the function, with bigger values suggesting higher relevance, and n indicates the number of tissue-specific miRNAs included.

In total, 29 rumen DE (24 up-regulated and 5 down-regulated), 2 jejunum up-regulated, 24 liver DE (14 up-regulated and 10 down-regulated), and 20 mammary gland DE (16 up-regulated and 4 down-regulated) miRNAs were identified (Table S3). For example, miR-21-3p and -2285f were up-regulated (P < 0.001) in rumen and liver, respectively. The predominant functions targeted by the tissue-DE miRNAs were cell death and survival, cellular growth and proliferation, and organismal survival for both tissue up-regulated miRNAs (Fig. 4A) and down-regulated miRNAs (Fig. 4B). The detailed information on predicted functions of all identified tissue-dependent DE miRNAs are listed in Table S4.

### Characterization of bovine-specific miRNAs

**Figure 5:**
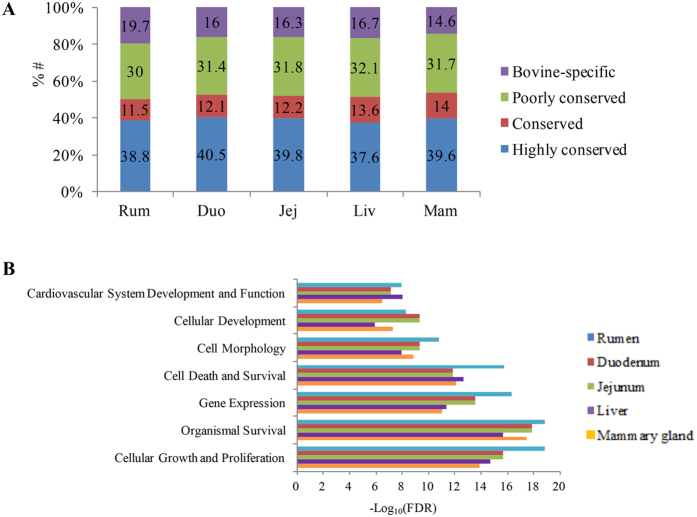
(**A**) Conservation profile of microRNAs (miRNAs) in the rumen (Rum), duodenum (Duo), jejunum (Jej), liver (Liv), and mammary gland (Mam); (**B**) Predominant function categories targeted by bovine-specific miRNAs in Rum, Duo, Jej, Liv and Mam. *Note:* -log_10_(FDR) values indicate the relevance of the function, with bigger values suggesting higher relevance.

Bovine-specific miRNAs were also characterized through the conservation analysis for the known miRNAs (n = 388) that were detected from all tissues. In total, 139 highly conserved, 48 conserved, 120 poorly conserved and 81 bovine-specific miRNAs were categorized (Table S5). In total, 67 (19.7%), 54 (16.0%), 55 (16.3%), 55 (16.7%), and 48 (14.6%) of the known miRNAs belonged to bovine-specific miRNA profiles in rumen epithelium tissue, duodenum, jejunum, liver and mammary gland, respectively (Fig. 5A).

For each tissue, the top 10 most highly expressed bovine-specific miRNAs covered more than 80% of the bovine-specific reads (data not shown). Based on the functional analysis, the three most predominant function categories targeted by the top 10 most highly expressed bovine-specific miRNAs in the rumen, duodenum and jejunum were cellular growth and proliferation, organismal survival, and gene expression (Fig. 5B and Table S6), with specific predicted functions in regulating abnormal morphology of epithelial tissue (FDR = 1.98E-05) and proliferation of epithelial cells (FDR = 1.46E-04) in rumen (Table S6). For duodenum and jejunum, the main predicted functions were organism survival, cellular growth and proliferation and gene expression (Fig. 5B), with specific functions in regulating epithelial cell death (FDR = 9.74E-04 (duodenum) and 1.05E-03 (jejunum)) and abnormal morphology of epithelial tissue (FDR = 1.12E-05 (duodenum and jejunum), Table S6). For liver and mammary gland, the main predicted functions were organismal survival, cellular growth and proliferation, and cell death and survival (Fig. 5B), with specific functions in regulating fatty acid metabolism (FDR = 0.01) and accumulation of lipids (FDR = 4.38E-04), and gluconeogenesis (FDR = 1.56E-03) in the liver. The functions targeted by bovine-specific miRNAs included synthesis of lipid, synthesis of carbohydrate, and catabolism of AAs (FDR = 0.02) in mammary gland (Table S6).

### Predicted target genes of identified tissue specific and DE miRNAs

To further predict the function of identified miRNAs that were expressed uniquely among tissues (DE or specific), RNA-Seq data (data not shown) were used to determine the association between the miRNA levels and their predicted targets (Table 2). Among these genes, the expression of the ATPase Na^+^/K^+^ transportation alpha 2 polypeptide (*ATP1A2*) gene was predicted to be negatively correlated with miR-21-3p (R = −0.54, P = 0.02). Additionally, the solute carrier family 7, member 8 (*SLC7A8*) transcript was predicted to be targeted by miR-2285f (R = −0.53, P = 0.02). These two genes were predicted to be involved in AA transportation by Panther.

### Quantitative real-time PCR validation of expression of dairy efficiency-associated miRNAs

**Figure 6:**
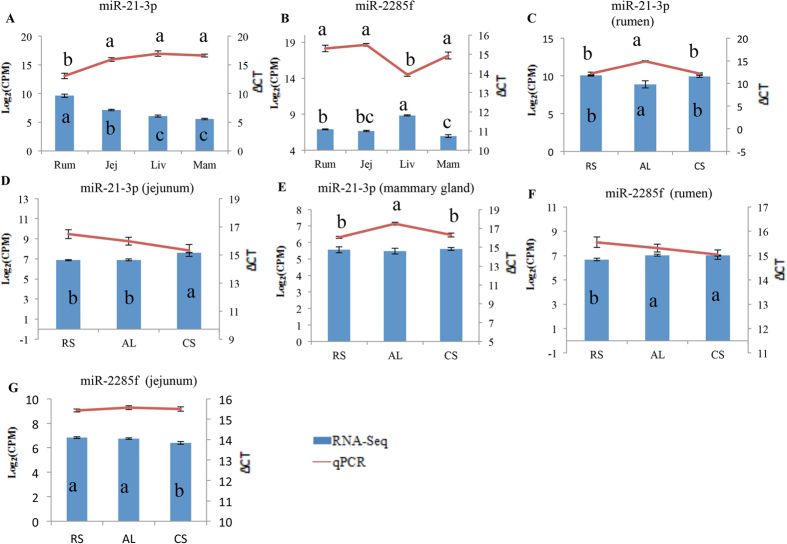
Quantitative real-time PCR validation of miR-21-3p and miR--2285f expressions (**A**) Differential expression of miR-21-3p among different tissues. (**B**) Differential expression of miR-2285f among different tissues. (**C–E**) Dietary effect on miR-21-3p expression in rumen, jejunum and mammary gland, respectively; (**F,G**) dietary effect on miR-2285f expression in rumen and jejunum, respectively. *Note*: (a–c) indicate significant different; Rum: rumen; Jej: jejunum; Liv: liver; Mam: mammary gland; AL: alfalfa hay diet; RS: rice straw diet; CS: corn stover diet.

MiR-21-3p (rumen up-regulated) and miR-2285f (bovine-specific and liver up-regulated) were selected for qPCR validation due to their targeting of genes involved in amino acids transport. The qPCR analysis confirmed that the expression of miR-21-3p was higher in the rumen and miR-2285f was up-regulated in the liver, compared with the other four tissues (P < 0.05, Fig. 6). Additionally, miR-21-3p was down-regulated by the AL diet in the rumen and mammary gland compared with the RS and CS diets (P < 0.05, Fig. 6).

### Association between miRNA expression and dairy efficiency phenotypes

In comparison with RS and CS diets, the dairy efficiency including feed efficiency (P < 0.01) and N efficiency (P = 0.01) were significantly higher and the MUN content (P < 0.01) was significantly lower under AL diet (Table 3). Correlation analysis of miRNA expression and the dairy efficiency traits (feed efficiency, N efficiency, and MUN content) identified the miRNAs significantly correlated with these traits under AL, CS and RS diet, respectively (Table S7). Specifically, when cows were fed with AL diet, the expression of 53 (11-rumen, 4-duodenum, 1-jejunum, 2-liver, and 35-mammary gland) and 32 (7-rumen, 2-duodenum, 5-jejunum, 8-liver, and 10-mammary gland) miRNAs was positively (R > 0.81, P < 0.05) or negatively correlated (R < −0.81, P < 0.05) with N efficiency, respectively (Table 4). Under CS diet, the expression of 39 (12-rumen, 5-duodenum, 10-jejunum, 9-liver, and 3- mammary gland and 24 (5-rumen, 6-duodenum, 2-jejunum, 6-liver, and 5-mammary gland) miRNAs was positively (R > 0.81, P < 0.05) or negatively correlated (R < −0.81, P < 0.05) with N efficiency, respectively. Similarly, under RS diet, this trait was positively (R > 0.81, P < 0.05) or negatively correlated (R < −0.81, P < 0.05) with the expression of 29 (5-rumen, 17-jejunum, 3-liver, and 4-mammary gland) and 23 (9-rumen, 3-duodenum, 3-jejunum, 7-liver, and 1-mammary gland) miRNAs, respectively.

Among the identified dairy efficiency-correlated miRNAs, the expression of some miRNAs was correlated with both feed and N efficiency in the same tissue, under certain diet (Table S7). When cows were fed AL diet, expression of three ruminal miRNAs including miR-125-5p, -130a, -2376, three duodenal miRNAs including miR-2483-5p, -2286l, -2336, two hepatic miRNAs including miR-199a-3p, -2399-5p and fourteen mammary gland miRNAs such as miR-196a, -205 was positively correlated with feed (R ranged from 0.81 ~ 0.99, P < 0.05) and N efficiency (R ranged from 0.81 ~ 0.98, P < 0.05), whereas the expression ruminal miR-1296, duodenal miR-6123, two jejunal miRNAs including miR-30b-3p, -652, five hepatic miRNAs including miR-1, -2285e, -421, -455-3p, -671, and mammary gland miR-99b was negatively correlated with feed (R ranged from −0.82 ~ −0.94, P < 0.05) and N efficiency (R ranged from −0.84 ~ −0.92, P < 0.05) (Table S6). When cows were fed RS diet, the expression of two ruminal miRNAs including miR-101, -361, six jejunal miRNAs including miR-125b, -199c, -221, -2285c, -324, -33b, and two hepatic miRNAs including miR-219-5p, -3613, was positively correlated with feed (R ranged from 0.81 ~ 0.96, P < 0.05) and N efficiency (R ranged from 0.81 ~ 0.97, P < 0.05), whereas the expression of jejunal miR-378b, three hepatic miRNAs including miR-154b, -2403, -493, and three mammary gland miRNAs including miR-192, -29d, -411c-3p was negatively correlated with feed (R ranged from −0.81 ~ −0.93, P < 0.05) and N efficiency (R ranged from −0.83 ~ −0.97, P < 0.05) (Table S7). When cows consumed CS diet, the expression of duodenal miR-148a, was positively correlated with feed (R = 0.84) and N efficiency (R = 0.85), where as the expression of ruminal miR-99b, two hepatic miRNAs including miR-502b, −874, and mammary gland let-7b was negatively correlated with feed (R ranged from −0.81 ~ −0.89, P < 0.05) and N efficiency (R ranged from −0.82 ~ −0.99, P < 0.05) (Table S7)

Moreover, the expression of some miRNAs was correlated with the same trait in different tissues, under certain diet (Table S7). For example, when cows were fed AL diet, out of ten such dairy efficiency associated miRNAs the expression of miR-6119-5p was positively correlated with N efficiency in rumen (R = 0.87, P < 0.05) and mammary gland (R = 0.84, P < 0.05), while the expression of miR-21-3p was negatively correlated with this trait in the same tissues (R = −0.85 for rumen and R = −0.93 for mammary gland) (Table 4). Similarly, when cows were fed RS diet, out of eight such dairy efficiency associated miRNAs the expression of miR-502a was positively correlated with N efficiency in rumen (R = 0.86) and jejunum (R = 0.81), while the expression of miR-6524 was negatively correlated with this trait in the same tissues (R = −0.91 for rumen and R = −0.85 for jejunum) (Table 4). When cows were fed CS diet, out of 8 such dairy efficiency associated miRNAs, none of them was associated with N efficiency, while the expression of miR-15b was positively correlated with feed efficiency in jejunum (R = 0.83) and mammary gland (R = 0.84) and the expression of miR-2285f was negatively correlated with feed efficiency in rumen (R = −0.83) and duodenum (R = −0.98) (Table S7).

### Effects of diet on dairy efficiency and expression of dairy efficiency-associated miRNAs

The expression of some dairy efficiency-associated miRNAs was also affected by the diet. When cows were fed AL diet, the expression of some of the dairy efficiency positively associated miRNAs was different comparing to those under RS and/or CS diets. For example, the expression of rumen specific miRNA miR-2299-3p (one of the N efficiency positively associated miRNAs) tended to be lower than that that under RS (P = 0.094) or CS (P = 0.056) diet (Table S7), while the expression of miR-103 in the rumen under RS (P = 0.004) or CS (P = 0.034) diet was higher than that under AL diet (Table S7).

### Identification of dairy efficiency associated tissue-specific, tissue DE miRNAs

As shown in Table S6, some of dairy efficiency associated miRNAs were tissue specific or tissue DE (up-regulated or down-regulated, Tables S2, S3). For example, from N efficiency associated miRNAs identified under AL diet, five of them were tissue specific including miR-2387, -2425-5p, and -2299-3p in the rumen, miR-2285l in the duodenum and miR-2399-5p in the liver and two were tissue DE (mammary gland up-regualted: miR-10b and -99a-5p). From those identified under RS diet, three rumen DE miRNAs (up-regulated, miR-149-5p and -21-3p; down-regulated, miR-2419-5p) and two mammary gland up-regulated miRNAs (miR-96 and -99a-5p) were identified. Similarly, for those indentified under CS diet, one was rumen specific (miR-2349), and five were tissue DE including three rumen up-regulated (miR-147, -93, and -27b), two liver DE (up-regulated, miR-874; down-regulated, miR-145down-regulated miRNA (miR-145) and one up-regulated miRNA (miR-874) and one mammary gland upregulated (let-7c).

### Functional analysis of dairy efficiency associated miRNAs

The functional analysis revealed that dairy efficiency associated miRNAs targeted specific functions such as AA metabolism, glucose metabolism, cellular proliferation/apoptosis (Table S8). In the current study, we focused on AA metabolism related functions (Table 5). When cows were fed AL diet, transport (FDR = 1.53E-04, n = 13, such as miR-130a) and metabolism (FDR = 5.15E-03, n = 13, miR-130a) of AAs predicted to be targeted by rumen positively associated miRNAs and release of L-AAs (FDR = 4.51E-04, n = 13, such as miR-1296) were predicted to be controlled by rumen negatively associated miRNAs. Transport of AAs (FDR = 4.30E-02, n = 12, such as miR-2483-5p) was predicted to be regulated by duodenum positively correlated miRNAs. Phosphorylation of L-AAs (FDR = 5.45E-04, n = 20, such as miR-30b-3p), and transport of AAs (FDR = 7.96E-04, n = 20, such as miR-30b-3p) was predicted to be impacted by jejunum negatively associated miRNAs. Phosphorylation of AAs (FDR = 2.36E-06, n = 14, such as miR-199a-3p) was predicted to be controlled by hepatic positively correlated miRNAs. The phosphorylation of L-amino acid (FDR = 2.62E-05, n = 13, such as miR-1) was targeted by liver negatively associated miRNAs. Phosphorylation of AAs (FDR = 1.83E-08, n = 44, such as miR-205) was predicted to be impacted by mammary gland positively correlated miRNAs. Transport of AAs (FDR = 9.23E-03, n = 10, such as miR-99b) was predicted to be regulated by mammary gland negatively associated miRNAs.

When cows were fed RS diet, phosphorylation of L-AAs (FDR = 2.26E-06, n = 29, such as miR-493) was predicted to be impacted by liver negatively associated miRNAs. phosphorylation of L-AAs (FDR = 2.66E-06, n = 31, such as miR-192) was predicted to be regulated by mammary negatively correlated miRNAs. When cows consumed CS diet, release of AAs (FDR = 9.01E-05, n = 32, such as miR-148b) was predicted to be impacted by duodenum positively correlated miRNAs. Transportation of AAs (FDR = 4.15E-05, n = 22, such as miR-2285f) was predicted to be controlled by duodenum negatively associated miRNAs. Phosphorylation of L-AAs (FDR = 4.11E-04, n = 17, such as miR-15b) was predicted to be regulated by jejunum positively miRNAs. Phosphorylation of L-AAs (FDR = 3.45E-06, n = 36, such as let-7b) was regulated by liver positively associated miRNAs. phosphorylation of L-AAs (FDR = 7.76E-04, n = 13, such as miR-15b) was predicted to be influenced by mammary positively correlated miRNAs and phosphorylation of amino acids (FDR = 1.83E-08, n = 44, such as miR-20a) was predicted to be controlled by mammary negatively correlated miRNAs.

## Discussion

The functions of miRNA in mammalian animals have been widely studied^26^, including their potential roles in regulation of nutrient metabolism in different tissues in humans^27^, pigs^28^ and cows^29^,^30^. MiRNAs may play important roles in lactation initiation and milk component (protein and fat) synthesis because certain miRNAs are highly expressed in the mammary gland of dairy cows^31^,^32^ and expression of mammary gland miRNAs can be affected by the diet^33^. Thus, miRNA-mediated regulation in the mammary gland is considered important for the lactation process^31^,^32^. However, most of these studies only focused on mammary gland tissue and lactation, which precludes a comprehensive understanding of the molecular mechanisms of milk production, especially milk protein synthesis.

In the current study, we focus on studying the miRNAs and their roles in protein metabolism from various metabolic tissues in dairy cows. To our knowledge, this is the first study to compare miRNA expression profiles and their functions in different tissues of the same animal and their changes under different diets using RNA-Seq. Among all the tissues of the cows, rumen, duodenum, jejunum, liver, and mammary gland are considered to be the most important organs for protein metabolism^33^,^34^,^35^,^36^. Rumen is the specific organ in ruminant animals where microbial crude protein synthesis is performed and N sources (AAs, peptides and ammonia) are absorbed^36^. Duodenum and jejunum are sites for protein digestion and absorption^35^, while liver is the key site for AA re-synthesis and urea cycle^34^. Finally, milk protein is synthesized in mammary gland^33^.

In this study, the 289 commonly expressed miRNAs identified across these five tissues suggest that the functions involved in the same biological or physiological processes in these tissues may be partially regulated by these miRNAs. For both tissue-DE and tissue-specific miRNAs, the most commonly affected enriched functions were basic cellular functions, such as cellular morphology and cellular movement, which were similar to each other, suggesting basic cellular functions could be mediated by different miRNAs. At the same time, the identification of tissue-DE and tissue-specific miRNAs suggests that these miRNAs may play critical roles in the specific functions of these cell types^37^, contributing to the different physiological functions of the five tissues^33^,^34^,^35^,^36^. More fundamental studies should be performed to investigate how tissue-DE and tissue-specific miRNAs play their roles in each tissue. Regardless, abundant miRNAs from these five tissues suggest their unique roles in the digestive tract.

From detected miRNA in mammary gland, only a few of them overlapped between our current study and previous reports^31^,^32^. This could possibly due to different physiological stages (dry and early lactating^31^,^32^ vs. mid-lactating in this study) and different methods for sampling tissues (biopsy^31^,^32^ vs. slaughter in this study). However, some of the previously reported miRNAs that regulate lactation initiation in mammary gland^31^,^32^ were detected in all five metabolic tissues and were likely to be involved in feed and N efficiency regulation of the dairy cows. For example, miR-125b, miR-141 and miR-181a have been associated with milk lipid synthesis in mammary gland of lactating cows^31^, and the expression of miR-221 and miR-205 is correlated with lactating initiation^32^. In our study, the expression of miR-125b, miR-141, miR-181a, miR-221 and miR-15b were detected in all five tissues (Table S2) and they were associated with feed or N efficiency under different diets (Table 2 and Table S7). Cell proliferation is an important function in lactation initiation, milk precursor absorption and milk component synthesis in mammary gland as well as in rumen and adipose tissues^38^,^39^. There is evidence that miR-125b, miR-141, miR-181a, miR-221 and miR-205 are involved in cellular proliferation^40^,^41^,^42^,^43^,^44^, suggesting that these miRNAs play roles in lactation initiation, feed efficiency and N efficiency in dairy cows at different physiological stages by regulating cell proliferation. Moreover, the expression of miR-125b in jejunum and miR-141 in mammary gland were impacted by the diet (Table S7), suggesting that nutritional changes could influence the expression of those miRNAs which could regulate milk initiation as reported as well as feed efficiency, and N efficiency as observed in this study.

Our results on identification of miRNAs associated with feed efficiency, N efficiency or MUN content in all five tissues suggested that miRNAs may be involved in nutrient utilization (indicated by feed efficiency) and N source utilization (indicated by N efficiency or MUN content) through the gut, in addition to mammary gland. For example, miR-130b, a dairy efficiency negatively associated miRNA with lower expression under AL comparing to both RS and CS in the rumen (Table S7), has been reported to regulate cellular proliferation in muscle. Another dairy efficiency negatively associated miRNA, miR-378, which had highest expression in the rumen under CS (Table S7), is associated with cellular proliferation and differentiation^46^. In addition, hepatic miRNAs such as miR-493 (under RS diet) and mammary let-7b (higher in AL diet, compared with CS diet) have been reported to be involved in cellular proliferation^47^,^48^. Thus, let-7b may regulate AAs metabolism by impacting cell proliferation in mammary gland. These further suggest that enhanced cellular proliferation and differentiation in various tissues by AL could be an import mechanism that could contribute to the variation in diary efficiency.

Some miRNAs were associated with more than one of the dairy efficiency traits in different tissues. For example, under RS diet, the expression of miR-125b was positively associated with feed efficiency in duodenum and jejunum (Table S7). MiR-125b were previously reported to regulate cellular proliferation^49^, so the expression change of this miRNA may impact the proliferation rates of the duodenum and jejunum epithelium and further regulate nutrients utilization. Similarly, miR-15b may impact cellular proliferation^50^ in mammary gland by regulating feed efficiency under CS diet. Moreover, AA metabolism in dairy cow impacted N efficiency^51^. When cows were fed AL diet, lower expression of ruminal miR-16b, -181d, indicating their less inhibitory effect on the genes that may regulate the AA metabolism. Overall, the data show that miRNAs may regulate protein metabolism in all tissues, suggesting the miRNAs have systematic regulatory functions in feed efficiency and N efficiency (Table 2). Our results suggest that milk production is a process that could be regulated by miRNAs in all five of these tissues. Moreover, regulating AAs transport function was a method to regulate N efficiency^52^, and phosphorylation of AAs was one mechanism for regulation of AAs transporter^53^. Our study revealed that systematic phosphorylation (duodenal miR-1296, jejunal miR-30b-3p, hepatic miR-199a-3p and mammary miR-196 (data not shown)) and transport of AAs (ruminal miR-130a, duodenal miR-1296, jejunal miR-30b-3p and mammary miR-196 (data not shown)) may be conducted by mediated-miRNAs, suggesting that miRNA-mediated mechanisms regulated N efficiency by regulating AAs transport and phosphorylation throughout all these five tissues. More studies should be conducted to reveal the mechanism of the systematic association between miRNAs and efficiency (feed and N) in different tissues.

It is known that transport of AAs plays an important role in dairy feed and N efficiency^55^. MiRNAs associated with N or feed efficiency in the five tissues targeted genes involved in AA transportation and metabolism, some of which are tissue-specific or tissue-DE. Different tissues may have unique miRNA-mediated regulation mechanisms for the same function, possibly induced by tissue-DE or tissue-specific miRNAs^21^. For example, *ATP1A2* may regulate Na^ + ^/K^ + ^pump and hydrolysis of ATP^56^, which is important in AA absorption of gut epithelial cells^36^. The negative association (R = −0.87, P < 0.05) between miR-21-3p and *ATP1A2* identified in the current study suggests the importance of miR-21-3p in AA transportation through the rumen epithelium tissue, which could impact milk production. In addition, the relatively higher expression of miR-21-3p in rumen under RS diet (Fig. 6A) indicate the diet may affect this function, which may lead to the altered AA metabolism contributing lower milk protein quality. Furthermore, functions including transport, metabolism and oxidation of AAs regulated by dietary DE (between high- and low-quality forage diets) miRNAs were associated with N efficiency or MUN content. For example, miR-21-3p was down-regulated in both rumen (Fig. 6C) and mammary gland (Fig. 6E) in the AL compared with the RS and CS groups, suggesting that miRNAs may respond systematically to dietary changes and target similar functions. In summary, miRNA-mediated regulation may play a role in AA metabolism in the five tissues, but the mechanisms could be varied among tissues.

Due to anatomical differences, many physiological metabolic processes are different between ruminants and monogastric animals, and these processes can be regulated by host-specific miRNAs. The current study indicates that bovine-specific miRNAs regulate abnormal morphology and proliferation of epithelial tissue, which may contribute to the nutrient absorption by the rumen^57^. Therefore, although bovine-specific miRNAs had low expression (accounting for 0.3% of the total reads), we speculate that they are involved in ruminant-specific functions. Branched chain amino acid (BCAA) (such as leucine) catabolism is lower in ruminants than monogastric animals due to a lack of BCAA aminotransferase^58^. Our results showed that a liver-up-regulated bovine-specific miRNA, miR-2285f (Fig. 6B), targets *SLC7A8*, a gene involved in high-affinity transport of small and large neutral AAs, including leucine. This suggests that miR-2285f may inhibit leucine membrane transportation by down-regulating *SLC7A8* to reduce the potential accumulation of leucine in the liver^58^. In addition to AA metabolism, bovine-specific miRNAs may regulate lipid and fatty acids metabolism in liver, which is also different from monogastric animals, especially in terms of the relatively lower-density lipoprotein in dairy cows^59^. Some of the bovine-specific miRNAs, such as miR-2285t (Higher expressed under RS, compared with CS) and miR-2419-5p (lower expressed under AL diet, compared with CS) in jejunum, were also involved in AA transport. These results suggest the potential functions of bovine-specific miRNAs in epithelium proliferation/morphology, AA metabolism, and other specific functions.

**Figure 7:**
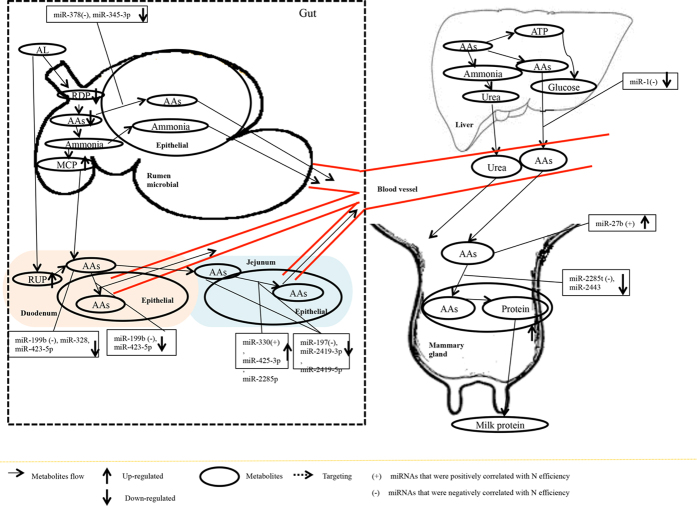
A working hypothesis of mechanism through which quality of forage affects amino acids and energy metabolism function in dairy cows, suggesting roles that could be played by microRNAs (miRNAs). *Note*: positively-associated miRNAs (positively correlated with nitrogen efficiency and feed efficiency or negatively correlated with milk urea nitrogen content) and differentially expressed (DE, AL vs RS or (and) AL vs CS) in cows fed AL is indicated by (+) while negative-associated miRNAs (negatively associated with nitrogen efficiency and efficiency or positively associated with milk urea nitrogen content) and differentially expressed (DE, AL vs RS or (and) AL vs CS) in cows fed AL is indicated by (−). The molecular flows are indicated by slender arrows. Bolded solid arrows indicate increase (up) and decrease (down) for either molecules quantity or expression of miRNA. Broken line indicates certain metabolites regulate biological process of molecules. Abbreviations: AL = alfalfa hay diet; RS = rice straw diet; CS = corn stover diet; RDP = rumen degradable protein; AAs = amino acids; MCP = microbial crude protein; VFAs = volatile fatty acids; RUP = rumen undegradable protein.

Compared with CS or RS diet with the similar N content, high-quality forage (AL) has a higher content of soluble carbohydrate, improved N efficiency, feed efficiency, and increased milk yield and protein yield by enhancing microbial protein synthesis, short-chain fatty acid production, and AA utilization^13^,^14^. Based on previous studies and our results, we propose that diet can regulate AA transportation and metabolism in rumen, duodenum and jejunum through pathways mediated by miRNAs (Fig. 7, Fig. S1 and Fig. S2). Compared with low quality forage (CS and RS), high quality forage diet (AL) can potentially change AAs uptake by changing the expression of miRNAs associated with dairy efficiency (P < 0.05, Fig. 7) in rumen (miR-378 and -345-3p), duodenum (miR-199b), jejunum (miR-330, -425-3p, -2285p, -197, 2419-3p and 2419-5p), liver (miR-1), and mammary gland (miR-2285t and -2443), and miRNA driven phosphorylation of AAs in duodenum (miR-199b, -328, and -423-5p) and jejunum (miR-197, -2419-3p, and -2419-5p). The feeding of low quality forage diet, however, seemed to impact AA metabolism by other mRNAs. For example, compared with AL diet, RS diet regulated AA transport in the rumen by changing expression of ruminal miR-103, -155, -504, -21-3p, and -142-3p (P < 0.05), and AAs phosphorylation through rumen (miR-103, -155, -504, -21-3p, and -142-3p) and liver (miR-497) (Fig. S1). Similarly, under CS diet (Fig. S2), the N and feed efficiency was decreased by regulating AAs phosphorylation in jejunum (by miR-2285r, -2483-5p, and -181c) and mammary gland (let-7b, -7c, -7a-3p, miR-106b, -1296, -188, -149-5p and -6119-3p), as well as AA transport in mammary gland (let-7c). The changes in the expression of miRNAs correlated with N efficiency and feed efficiency in all five tissues (Table S7) could explain the metabolic and productive changes under low-quality forage diets, as observed in our previous studies^13^,^14^. For example, metabolomics profiles of dairy bio-fluids suggest diet induced altered AA metabolic pathways which leads to reduced milk production and quality under RS and CS diets^13^. Our study further revealed that AA metabolism, phosphorylation and transport in the gut region (rumen, duodenum and jejunum) and metabolic organs (liver and mammary gland) may be changed in miRNA-mediated pathways, which are related to the reduction in feed and N efficiency under low quality forage diets^12^.

## Conclusions

By using the high throughput RNA-Seq, our results showed how miRNAs are involved in biological process or physiological functions in five key metabolic tissues in the dairy cow, considering the animal as a whole biological system. By profiling miRNAs in key tissues of dairy protein metabolism (rumen, duodenum, jejunum, liver and mammary gland), our study reveals that 1) in addition to mammary gland, miRNAs from rumen, duodenum, jejunum and liver could contribute to feed and N efficiency systematically in mid-lactating dairy cows; 2) each tissue has its functional uniqueness due to their physiological and functional differences, which could be regulated by miRNAs, and miRNA-mediated mechanisms could be involved in AA metabolism in the five tissues; 3) bovine-specific miRNAs may be involved in unique functions, such as BCAA metabolism; 4) diet can significantly change the expression of dairy efficiency associated miRNAs which suggests that nutritional alterations could be a method to regulate the miRNA profile in these protein metabolism-related tissues, and may help with the feeding management of the dairy cows; 5) Low-quality forage diets can impact AA metabolism in the gut by altering miRNA expression, and post-transcriptional regulation patterns in regulation AAs metabolism were differed among different diets, suggesting potential ways to improve milk protein quality by manipulation of miRNAs under low-quality forage. By identifying miRNAs that regulate feed and N efficiency and measuring their changes under diets with different forages, our results provide fundamental understanding on the molecular regulatory mechanism through miRNA regulation which could explain the reduced feed and N efficiency under low-quality forage diets. Whether these miRNAs regulate these traits are undefined and future studies using *in vitro* system such as cell-lines (mammary gland and/or epithelial cells) or *in vivo* feeding trials (feeding with nucleotides based on identified miRNAs) are needed to verify the findings from current study.

## Additional Information

**How to cite this article**: Wang, D. *et al.* Systematic microRNAome profiling reveals the roles of microRNAs in milk protein metabolism and quality: insights on low-quality forage utilization. *Sci. Rep.* **6**, 21194; doi: 10.1038/srep21194 (2016).

## Supplementary Material

Supplementary Information

## Figures and Tables

**Table 1:** Predominant highly expressed miRNAs in rumen, duodenum, jejunum, liver and mammary gland.

Tissue	# of identified miRNAs		Top highly expressed known miRNAs (% of total mapped reads)
	Known	Novel	
Rumen	340	32	miR-143 (17.4), miR-27b (10.4), miR-26a (10.2)
Duodenum	338	37	miR-192 (25.7), miR-143 (16.1), miR-26a (7.4)
Jejunum	337	37	miR-143(21.1), miR-192(19.4) miR-26a (6.9)
Liver	330	26	miR-192 (13.8), miR-22-3p (12.9), miR-143 (12.7)
Mammary gland	328	36	miR-143 (14.6), miR-148 (13.5), miR-30-5p (10.5)

**Table 2:** Predicted targets of tissue differential or specific expressed microRNAs (miRNAs).

MiRNA	Tissue variation	Predicted targets
miR-378	Rumen(up)	OS9, PDE1B, THY1, ARAP3, CCDC150, FBLN5, FZD1, Bt.28572, GYPC, HOXB3, JOSD1, LEPREL1, MXRA8, PEA15, PID1, PPP1R16B, RHBDD1, S1PR1, SCARA3, SCRN1
miR-93	Rumen(up)	APP, CTSA, EGLN3, FEM1C, KCNJ8, TMBIM6, HNF4G, LAMP1, RDX, SYBU
miR-1271	Rumen(up)	—
miR-21-3p	Rumen(up)	HEY2, SSFA2, ST6GAL2, CYP39A1, RIC3, FAM13A, HEYL, FMO5, COBL, ANK3, PLA2G2F, ACVR2A, SCRN1, TMBIM6, ANK3, ATP1A2, ENC1, MAP7, ALS2
miR-27a-3p	Rumen(up)	CR2, FANCI, LAMC1
miR-27b	Rumen(up)	FANCI
miR-24-3p	Rumen(up)	ACSL6, LHFPL2, NUP210, RAP1A, CASC3, DNAJC27, DPF2, EIF2AK3, GOLGA5, HMG20A, IL33, KPNB1, PHACTR1, POLD3, SASH3, SYK, TGM2
miR-122	Liver(specific)	BCAT2, GYS1, P4HA1, DBNDD1, HLX, LDLR, NAB2, RCE1, RHOF, SPOCK2
miR-2285f	Liver(up)	SLC7A8, DDIT4L, PI15, DCLK1, NCALD
let-7c	Mammary gland (up)	—
miR-2285t	Mammary gland (up)	—
miR-99a-5p	Mammary gland (up)	SSR1, LAMP3, SGTA, SSR3, SLMAP

^#^No highly negative correlated targets were identified.

**Table 3:** Effect of diets with different forage sources on feed intake, performance and efficiency in mid-lactating dairy cows.

Traits	Means*			P-value		
	AL	RS	CS	AL vs. RS	AL vs. CS	RS vs. CS
Dry matter intake, kg/d	18.26	18.31	18.10	0.917	0.764	0.760
Milk yield, kg/d	25.63	20.68	19.23	0.002	<0.001	0.230
4% fat-corrected milk yield, kg/d	26.29	21.73	20.37	0.032	0.001	0.437
Milk urea nitrogen yield, kg/d	3.83	3.55	3.25	0.288	0.013	0.206
Milk urea nitrogen content, mg/dL	15.43	17.70	17.47	0.003	0.009	0.635
Somatic cell count, 10^3^/mL	892.79	417.01	211.65	0.223	0.082	0.241
Total solid yield, kg/d	3.49	2.81	2.67	0.017	0.001	0.537
Total solid content, g/100g	13.60	13.45	13.87	0.784	0.491	0.465
Lactose yield, kg/d	1.26	0.98	0.93	0.006	<0.001	0.461
Lactose content, g/100g	4.91	4.72	4.82	0.222	0.211	0.469
Fat yield, kg/d	1.07	0.90	0.85	0.095	0.009	0.569
Fat content, g/100g	4.17	4.28	4.40	0.687	0.302	0.672
Protein yield, kg/d	0.84	0.66	0.64	0.004	<0.001	0.699
Protein content, g/100g	3.29	3.19	3.35	0.389	0.668	0.296
Nitrogen efficiency	0.28	0.23	0.22	0.016	0.002	0.699
Feed efficiency	1.41	1.14	1.06	0.004	<0.001	0.276

^*^AL = alfalfa hay; RS = rice straw; CS = corn stover.

**Table 4:** Correlation between N efficiency and expression of microRNAs in dairy rumen, duodenum, jejunum, liver, and mammary gland under different diets.

Tissue	Alfalfa hay		Rice straw		Corn stover	
	Positive	Negative	Positive	Negative	Positive	Negative
Rumen	miR-126-5p (0.85, 0.03)	miR-1 (−0.82, 0.05)	miR-101 (0.94, <0.01)	miR-142-3p (−0.84, 0.04)	miR-103 (0.85, 0.03)	miR-10a (−0.9, 0.01)
	miR-130a (0.82, 0.05)	miR-1296 (−0.92, <0.01)	miR-2419-5p (0.81, 0.05)	miR-146b (−0.81, 0.05)	miR-107 (0.87, 0.03)	miR-146a (−0.86, 0.03)
	miR-16b (0.84, 0.04)	miR-2387 (−0.91, 0.01)	miR-328 (0.88, 0.02)	miR-149-5p (−0.86, 0.03)	miR-138 (0.82, 0.05)	miR-2349 (−0.87, 0.03)
	miR-181d (0.86, 0.03)	miR-2425-5p (−0.82, 0.05)	miR-361 (0.84, 0.04)	miR-155 (−0.84, 0.04)	miR-147 (0.94, <0.01)	miR-27b (−0.9, 0.01)
	miR-2299-3p (0.85, 0.03)	miR-379 (−0.84, 0.04)	miR-502a (0.86, 0.03)	miR-18b (−0.85, 0.04)	miR-2284b (0.86, 0.03)	miR-99b (−0.83, 0.05)
	miR-2376 (0.86, 0.03)	miR-491 (−0.82, 0.05)	—	miR-21-3p (−0.85, 0.04)	miR-2284x (0.93, <0.01)	—
	miR-26b (0.82, 0.05)	miR-7 (−0.83, 0.05)	—	miR-2284aa (−0.83, 0.05)	miR-301a (0.83, 0.05)	—
	miR-6119-5p (0.87, 0.03)	—	—	miR-25 (−0.84, 0.04)	miR-335 (0.87, 0.02)	—
	miR-6123 (0.85, 0.03)	—	—	miR-6524 (−0.91, 0.01)	miR-545-3p (0.86, 0.03)	—
	miR-652 (0.93, <0.01)	—	—	—	miR-582 (0.96, <0.01)	—
	miR-660 (0.86, 0.03)	—	—	—	miR-877 (0.84, 0.04)	—
	—	—	—	—	miR-93 (0.83, 0.04)	—
Duodenum	miR-2285l (0.84, 0.04)	miR-145 (−0.85, 0.04)	—	miR-124a (−0.93, <0.01)	miR-148a (0.85, 0.03)	miR-210 (−0.89, 0.02)
	miR-2336 (0.84, 0.04)	miR-6123 (−0.89, 0.02)	—	miR-124b (−0.93, <0.01)	miR-193a-3p (0.88, 0.02)	miR-221 (−0.84, 0.04)
	miR-2483-5p (0.87, 0.02)	—	—	miR-376e (−0.92, <0.01)	miR-411c-5p (0.83, 0.04)	miR-2483-5p (−0.89, 0.02)
	miR-379 (0.91, 0.01)	—	—	—	miR-6520 (0.89, 0.02)	miR-454 (−0.83, 0.05)
	—	—	—	—	miR-671 (0.83, 0.05)	miR-6123 (−0.84, 0.04)
	—	—	—	—	—	miR-6529 (−0.83, 0.04)
Jejunum	miR-486 (0.9, 0.02)	miR-197 (−0.86, 0.03)	miR-125b (0.86, 0.03)	miR-1246 (−0.82, 0.05)	miR-106a (0.92, <0.01)	miR-2285b (−0.84, 0.04)
	—	miR-2285k (−0.9, 0.01)	miR-17-3p (0.93, <0.01)	miR-378b (−0.87, 0.03)	miR-1296 (0.97, <0.01)	miR-2285u (−0.84, 0.04)
	—	miR-30b-3p (−0.86, 0.03)	miR-18a (0.92, 0.01)	miR-6524 (−0.85, 0.04)	miR-150 (0.83, 0.05)	—
	—	miR-505 (−0.88, 0.02)	miR-199c (0.93, <0.01)	—	miR-181c (0.91, 0.01)	—
	—	miR-652 (−0.91, 0.01)	miR-19a (0.89, 0.02)	—	miR-2904 (0.85, 0.04)	—
	—	—	miR-200a (0.92, <0.01)	—	miR-320a (0.84, 0.04)	—
	—	—	miR-20a (0.95, <0.01)	—	miR-323 (0.83, 0.05)	—
	—	—	miR-221 (0.96, <0.01)	—	miR-363 (0.98, <0.01)	—
	—	—	miR-2285c (0.81, 0.05)	—	miR-449a (0.84, 0.04)	—
	—	—	miR-324 (0.91, 0.01)	—	miR-484 (0.82, 0.05)	—
	—	—	miR-33b (0.84, 0.04)	—	—	—
	—	—	miR-429 (0.81, 0.05)	—	—	—
	—	—	miR-455-3p (0.83, 0.04)	—	—	—
	—	—	miR-502a (0.81, 0.05)	—	—	—
	—	—	miR-6119-3p (0.88, 0.02)	—	—	—
	—	—	miR-671 (0.82, 0.05)	—	—	—
	—	—	miR-99a-3p (0.86, 0.03)	—	—	—
Liver	miR-199a-3p (0.83, 0.04)	miR-1 (−0.85, 0.04)	miR-219-5p (0.85, 0.03)	miR-154b (−0.83, 0.05)	miR-152 (0.83, 0.05)	miR-129-3p (−0.82, 0.05)
	miR-2399-5p (0.92, <0.01)	miR-133a (−0.9, 0.01)	miR-2468 (0.85, 0.03)	miR-181c (−0.96, <0.01)	miR-205 (0.82, 0.05)	miR-145 (−0.86, 0.03)
	—	miR-2285e (−0.86, 0.03)	miR-3613 (0.81, 0.05)	miR-182 (−0.89, 0.02)	miR-2284aa (0.94, <0.01)	miR-204 (−0.86, 0.03)
	—	miR-376e (−0.91, 0.01)	—	miR-2403 (−0.98, <0.01)	miR-2284y (0.83, 0.04)	miR-502b (−0.84, 0.04)
	—	miR-421 (−0.89, 0.02)	—	miR-363 (−0.83, 0.04)	miR-24 (0.85, 0.04)	miR-592 (−0.84, 0.04)
	—	miR-455-3p (−0.9, 0.02)	—	miR-493 (−0.85, 0.03)	miR-2419-5p (0.86, 0.03)	miR-874 (−0.89, 0.02)
	—	miR-502a (−0.87, 0.03)	—	miR-665 (−0.82, 0.05)	miR-30b-3p (0.84, 0.04)	—
	—	miR-671 (−0.84, 0.04)	—	—	miR-365-5p (0.88, 0.02)	—
	—	—	—	—	miR-532 (0.88, 0.02)	—
Mammary gland	miR-101 (0.97, <0.01)	miR-10b (−0.85, 0.03)	let-7e (0.91, 0.01)	miR-127 (−0.82, 0.05)	miR-1388-3p (0.82, 0.05)	let-7b (−0.83, 0.05)
	miR-1247-5p (0.94, <0.01)	miR-1271 (−0.84, 0.04)	miR-2411-5p (0.87, 0.03)	miR-1271 (−0.85, 0.04)	miR-17-3p (0.91, 0.01)	let-7c (−0.85, 0.03)
	miR-1296 (0.88, 0.02)	miR-146b (−0.88, 0.02)	miR-2478 (0.81, 0.05)	miR-148b (−0.85, 0.03)	miR-2285c (0.84, 0.04)	—
	miR-1306 (0.9, 0.02)	miR-99a-5p (−0.87, 0.02)	miR-375 (0.85, 0.04)	miR-183 (−0.81, 0.05)	—	—
	miR-1468 (0.89, 0.02)	miR-99b (−0.92, <0.01)	—	miR-18a (−0.84, 0.04)	—	—
	miR-148a (0.86, 0.03)	—	—	miR-191 (−0.91, 0.01)	—	—
	miR-16b (0.84, 0.04)	—	—	miR-192 (−0.97, <0.01)	—	—
	miR-181a (0.92, 0.01)	—	—	miR-21-3p (−0.93, <0.01)	—	—
	miR-181b (0.94, <0.01)	—	—	miR-2285k (−0.87, 0.02)	—	—
	miR-196a (0.91, 0.01)	—	—	miR-2313-3p (−0.86, 0.03)	—	—
	miR-205 (0.91, 0.01)	—	—	miR-29d (−0.84, 0.04)	—	—
	miR-210 (0.86, 0.03)	—	—	miR-411c-3p (−0.83, 0.04)	—	—
	miR-222 (0.85, 0.03)	—	—	miR-874 (−0.82, 0.05)	—	—
	miR-2284k (0.98, <0.01)	—	—	miR-96 (−0.89, 0.02)	—	—
	miR-2284n (0.98, <0.01)	—	—	miR-99a-5p (−0.93, <0.01)	—	—
	miR-2284w (0.83, 0.05)	—	—	—	—	—
	miR-2284x (0.92, 0.01)	—	—	—	—	—
	miR-2284y (0.81, 0.05)	—	—	—	—	—
	miR-2332 (0.93, <0.01)	—	—	—	—	—
	miR-2339 (0.83, 0.05)	—	—	—	—	—
	miR-25 (0.9, 0.01)	—	—	—	—	—
	miR-27b (0.92, <0.01)	—	—	—	—	—
	miR-320a (0.84, 0.04)	—	—	—	—	—
	miR-328 (0.95, <0.01)	—	—	—	—	—
	miR-331 (0.81, 0.05)	—	—	—	—	—
	miR-34b (0.81, 0.05)	—	—	—	—	—
	miR-363 (0.95, <0.01)	—	—	—	—	—
	miR-592 (0.9, 0.02)	—	—	—	—	—
	miR-6119-5p (0.83, 0.04)	—	—	—	—	—
	miR-6520 (0.94, <0.01)	—	—	—	—	—
	miR-769 (0.93, <0.01)	—	—	—	—	—
	miR-92b (0.82, 0.05)	—	—	—	—	—
	miR-95 (0.85, 0.03)	—	—	—	—	—
	miR-9-5p (0.84, 0.04)	—	—	—	—	—
	miR-99a-3p (0.87, 0.03)	—	—	—	—	—

**Table 5:** Amino acids metabolism related functions targeted by microRNAs associated with dairy efficiency of cows under different diets.

Item	Alfalfa hay		Rice straw		Corn stover	
	Positive	Negative	Positive	Negative	Positive	Negative
Rumen	Transport of amino acids (FDR = 1.53E-04, n = 13)	Release of L-amino acid (FDR = 4.51E-04, n = 13)	—*	Phosphorylation of L-amino acid (FDR = 2.72E-06, n = 18)	**—**	**—**
	Metabolism of amino acids (FDR = 5.15E-03, n = 13)	**—**	**—**	Transport of amino acids (FDR = 8.01E-06, n = 13)	**—**	**—**
	**—**	**—**	**—**	Release of amino acids (FDR = 5.01E-05, n = 17)	**—**	**—**
Duodenum	Transport of amino acids (FDR = 4.30E-02, n = 12)	Phosphorylation of L-amino acid (FDR = 3.93E-09, n = 68)	**—**	**—**	Release of amino acids (FDR = 9.01E-05, n = 32)	Transport of amino acids (FDR = 4.15E-05, n = 22)
	**—**	Release of L-amino acid (FDR = 8.89E-06, n = 58)	**—**	**—**	**—**	Phosphorylation of L-amino acid (FDR = 5.30E-05, n = 30)
	**—**	**—**	**—**	**—**	**—**	Abnormal quantity of amino acids (FDR = 2.55E-04, n = 26)
Jejunum	Transport of alpha-amino acid (FDR = 2.45E-04, n = 17)	Phosphorylation of L-amino acid (FDR = 5.45E-04, n = 20)	Phosphorylation of L-amino acid (FDR = 8.20E-07, n = 14)	Transport of amino acids (FDR = 1.36E-03, n = 6)	Phosphorylation of L-amino acid (FDR = 4.11E-04, n = 17)	**—**
	**—**	Transport of amino acids (FDR = 7.96E-04, n = 20)	Transport of amino acids (FDR = 1.59E-05, n = 15)	**—**	**—**	**—**
Liver	Phosphorylation of amino acids (FDR = 2.36E-06, n = 14)	Phosphorylation of L-amino acid (FDR = 2.62E-05, n = 13)	**—**	Phosphorylation of L-amino acid (FDR = 2.26E-06, n = 29)	Phosphorylation of L-amino acid (FDR = 3.45E-06, n = 36)	**—**
Mammary gland	Phosphorylation of amino acids (FDR = 1.83E-08, n = 44)	Transport of amino acids (FDR = 9.23E-03, n = 10)	**—**	Phosphorylation of L-amino acid (FDR = 2.66E-06, n = 31)	Phosphorylation of L-amino acid (FDR = 7.76E-04, n = 13)	Phosphorylation of L-amino acid (FDR = 3.93E-08, n = 49)
	**—**	**—**	**—**	Transport of amino acids (FDR = 3.91E-04, n = 28)	Release of amino acids (FDR = 2.74E-03, n = 14)	**—**

^*^No function identified. n represents the number of dairy efficiency associated miRNAs.
