# Supplementary material for: Systematic microRNAome profiling reveals the roles of microRNAs in milk protein metabolism and quality: insights on low-quality forage utilization
Source: Sci Rep. 2016 Feb 17;6:21194. doi: 10.1038/srep21194 (PMC4756660; doi:10.1038/srep21194)
Supplement: Supplementary Information [file srep21194-s1.pdf]

Systematic genome-wide microRNAome reveals their roles in protein metabolism associated with dairy milk quality: omics based insights on utilization of low quality forage resources

Diming Wang[1,*,#] , Guanxiang Liang [1,#], Bing Wang[*], Huizeng Sun[*], Jianxin Liu[*, §], Le Luo Guan[#, §]

[*]Institute of Dairy Sciences, College of Animal Sciences, Zhejiang University, Hangzhou, P R, China; [#] Department of Agricultural, Food and Nutritional Science, University of Alberta, Edmonton, Canada

[1]These authors contributed equally to this work.

[§]**Corresponding authors**:

Dr. Jianxin Liu: Tel: 86-571-88982097; Fax: 86-571-88982097; E-mail: liujx@zju.edu.cn

Dr. Le Luo Guan: Tel: 1-780-4922480; Fax: 1-780-4924265; E-mail: lguan@ualberta.ca

Supplementary information

Table S1. Function categories of the predicted targets of 20 top commonly expressed microRNAs in rumen, duodenum, jejunum, liver and mammary gland.

**Function category**	**FDR**	**# of gene**
Organismal Survival	6.64E-28	759
Cellular Growth and Proliferation	4.29E-22	1083
Cell Death and Survival	1.32E-21	995
Cell Morphology	1.23E-18	697
Cellular Movement	1.23E-18	643
Cardiovascular System Development and Function	1.02E-16	427
Organismal Development	1.61E-15	927
Cellular Function and Maintenance	8.58E-15	858
Tissue Morphology	5.19E-14	710
Cellular Development	8.72E-13	999
Molecular Transport	2.24E-12	613
Gene Expression	2.49E-11	649
Hematological System Development and Function	2.86E-11	539
Cell Cycle	8.95E-10	393
Organ Morphology	1.52E-09	403
Tissue Development	2.50E-09	820
Cellular Assembly and Organization	3.77E-09	560
Embryonic Development	5.47E-09	677
Lipid Metabolism	7.73E-09	394
Small Molecule Biochemistry	7.73E-09	599

Table S2. Tissue-specific miRNAs in the rumen, duodenum, jejunum, liver and mammary gland.

MicroRNA	Tissue	Average CPM	standard error
miR-1434-3p	Rumen	1.3	0.18
miR-2284f	Rumen	1	0.14
miR-2299-3p	Rumen	1.4	0.15
miR-2320-5p	Rumen	1.7	0.15
miR-2344	Rumen	1.8	0.3
miR-2349	Rumen	1.3	0.15
miR-2368-3p	Rumen	1.6	0.14
miR-2387	Rumen	1.9	0.41
miR-2425-5p	Rumen	1.5	0.4
miR-2448-3p	Rumen	1.6	0.25
miR-6121-3p	Rumen	1.4	0.26
miR-615	Rumen	2.4	0.28
miR-6525	Rumen	1.1	0.16
miR-6527	Rumen	1.9	0.3
miR-216b	Duodenum	1.2	0.11
miR-2284p	Duodenum	0.9	0.08
miR-2285l	Duodenum	1	0.13
miR-383	Duodenum	1.3	0.3
miR-2320-3p	Jejunum	1	0.1
miR-6526	Jejunum	2.9	0.47
miR-122	Liver	11020.9	277.62
miR-1247-3p	Liver	1.9	0.55
miR-1940	Liver	1	0.17
miR-2399-5p	Liver	1.7	0.19
miR-2415-3p	Liver	8.4	1.86
miR-2483-3p	Liver	29.2	4.23
miR-3957	Liver	1.9	0.42
miR-433	Liver	2.6	0.34
miR-551b	Liver	1.5	0.22
miR-6518	Liver	0.8	0.14
miR-6521	Liver	1.9	0.25
miR-758	Liver	1.7	0.19
miR-1298	Mammary gland	2.8	0.92
miR-134	Mammary gland	0.9	0.12
miR-196b	Mammary gland	62.6	8.01
miR-2284j	Mammary gland	12.3	1.19
miR-376d	Mammary gland	1.1	0.2

Table S3. Tissue-dependent differentially expressed miRNAs in the rumen, duodenum, jejunum, liver and mammary gland.

**MicroRNA**	**Rumen**	**Duodenum**	**Jejunum**	**Liver**	**Mammary gland**	**P**	**Standard error mean**	**Different expressed**
miR-10a	14558B	13736B	41168A	3275C	4047C	<0.001	2700.09	Jejunum(up)
miR-223	65.3C	244.1B	553.5A	107.7C	37C	<0.001	32.74	Jejunum(up)
miR-145	2296.5A	2241.2A	2383.5A	586.1C	1247.3B	<0.001	169.16	Liver(down)
miR-199b	1132.6A	1102.1A	1187.6A	215.6C	821.6B	<0.001	66.63	Liver(down)
miR-200a	3815.4A	2144.7B	1403B	100.8C	4264.8A	<0.001	385.28	Liver(down)
miR-2336	53.4A	52.8A	53.4A	19C	44.5B	<0.001	2.54	Liver(down)
miR-324	12B	23.5A	25.5A	5C	13.9B	<0.001	1.32	Liver(down)
miR-34a	185.7A	108.7B	105B	51.1C	119.1B	<0.001	11.16	Liver(down)
miR-362-5p	23A	14.4B	13B	5.2C	21.6A	<0.001	1.39	Liver(down)
miR-6120-3p	25.2B	29.5BA	28.2B	7C	35.2A	<0.001	2.32	Liver(down)
miR-101	5357.2B	2686.8C	3050C	21244.7A	6421.2B	<0.001	512.32	Liver(up)
miR-144	9.1B	7.4B	9.4B	84.5A	13.6B	<0.001	4.31	Liver(up)
miR-154c	9.7C	19CB	21.2CB	106A	36.1B	<0.001	6.08	Liver(up)
miR-185	25.5C	39.2CB	33.9CB	86.4A	43.2B	<0.001	5.68	Liver(up)
miR-22-3p	39540B	18005C	19203C	129073A	24654C	<0.001	3329.83	Liver(up)
miR-22-5p	34CB	26.8CD	23.2D	85.3A	39.3B	<0.001	3.42	Liver(up)
miR-2285f	133.9B	118.8B	114B	468.7A	76.4C	<0.001	9.48	Liver(up)
miR-339a	436.3C	470.8C	480.8C	1349.2A	659.9B	<0.001	42.91	Liver(up)
miR-410	14.7D	32.1CD	38.5CB	131.4A	57.8B	<0.001	8.04	Liver(up)
miR-411c-5p	13.8C	13.3C	17.5C	65.6A	27.7B	<0.001	3.37	Liver(up)
miR-451	203.9B	105.9B	131.6B	1063.7A	275.1B	<0.001	67.46	Liver(up)
miR-543	1.8CB	1.2C	1.4C	10.3A	3.1B	<0.001	0.55	Liver(up)
miR-874	23.4B	20.9B	26.4B	176.3A	15.7B	<0.001	4.41	Liver(up)
miR-487b	5C	10.3CB	11.4CB	42.8A	17B	<0.001	2.57	Liver(up),Rumen(down)
miR-146a	1225B	3664.6A	3802A	1204.7B	503.2C	<0.001	199.37	Mammary gland(down)
miR-455-3p	106.8A	78.1B	96.2BA	90.2BA	14.4C	<0.001	7.91	Mammary gland(down)
miR-505	35.9B	33.4B	30.7B	64.2A	14.4C	<0.001	2.18	Mammary gland(down)
let-7c	1064.5B	519.6C	530.1C	1105.7B	2324.6A	<0.001	123.61	Mammary gland(up)
miR-10b	28356CB	15974CD	37713B	1349D	98658A	<0.001	5898.92	Mammary gland(up)
miR-141	11165B	6511C	4630C	285D	29858A	<0.001	1523.83	Mammary gland(up)
miR-1839	145.6B	129B	125B	118.9B	362.6A	<0.001	19.69	Mammary gland(up)
miR-190a	15.8B	35.5B	44.1B	4.5B	108.7A	<0.001	15.8	Mammary gland(up)
miR-200c	1756.7B	1031.3C	682.5DC	29.3D	5113.8A	<0.001	245.28	Mammary gland(up)
miR-2285t	29.2B	33.2B	30B	73.5B	879.8A	<0.001	69.31	Mammary gland(up)
miR-340	12.8B	11.8B	10.8B	9B	28.3A	<0.001	2.08	Mammary gland(up)
miR-34c	4.2B	15B	10.3B	6.2B	61.8A	<0.001	4.82	Mammary gland(up)

miR-362-3p	8.4B	10.9B	9B	4.5B	32.9A	<0.001	2.28	Mammary gland(up)
miR-379	5.7B	16.4B	11.7B	13.7B	67.5A	0.045953539	15.38	Mammary gland(up)
miR-502a	2.4B	1.9B	2B	1.8B	12A	<0.001	0.6	Mammary gland(up)
miR-6522	5.9B	4B	5.5B	2.4B	95.1A	<0.001	2.15	Mammary gland(up)
miR-660	1465.7B	1139.4B	1166.1B	936.4B	3656.3A	<0.001	179.88	Mammary gland(up)
miR-96	22.9B	13.7CB	9.3CB	1.1C	93.1A	<0.001	5.27	Mammary gland(up)
miR-99a-5p	684.8B	441.1B	364B	607.3B	2487.4A	<0.001	207.02	Mammary gland(up)
miR-139	33.9E	92.8D	129.3C	226.2A	161.4B	<0.001	8.66	Rumen(down)
miR-2419-5p	25.4C	196BA	240.5A	141.5B	196.7BA	<0.001	22.84	Rumen(down)
miR-450b	23.9C	122.9BA	128.6A	142.9A	96.9B	<0.001	9.8	Rumen(down)
miR-574	16C	89.7A	84.5A	42B	37.4B	<0.001	3.4	Rumen(down)
miR-1271	132.7A	48.2B	38B	4.1B	46.3B	0.007178804	26.43	Rumen(up)
miR-147	688.4A	40.5B	48.5B	11.7B	17.6B	<0.001	14.49	Rumen(up)
miR-149-5p	183.1A	16.1B	17.1B	22.3B	20B	<0.001	6.13	Rumen(up)
miR-193a-3p	1225.9A	27.1C	28.6C	240.3CB	284.9B	<0.001	84.05	Rumen(up)
miR-19a	454.4A	179.4B	160CB	74D	97.2CD	<0.001	25.31	Rumen(up)
miR-210	736.2A	41.6B	41.3B	77.9B	92.1B	<0.001	18.04	Rumen(up)
miR-21-3p	808.9A	91.1B	148.4B	80.1B	79.3B	<0.001	34.14	Rumen(up)
miR-224	104.6A	19.7D	21.3D	49.5B	32.5C	<0.001	3.71	Rumen(up)
miR-23b-3p	5088.2A	2392B	2085.4CB	970.7D	1753.3C	<0.001	170.77	Rumen(up)
miR-27a-3p	11433.5A	2590.8B	2833.2B	1615.9C	2736.7B	<0.001	263.84	Rumen(up)
miR-27a-5p	23.1A	2.5C	2.6C	2.1C	7.2B	<0.001	0.82	Rumen(up)
miR-27b	103506A	31482CB	33606CB	36298B	30081C	<0.001	1833.8	Rumen(up)
miR-31	4519A	331.7B	278B	654.1B	520B	<0.001	145.1	Rumen(up)
miR-3431	125.6A	22.2C	21.7C	43.7B	40.3B	<0.001	3.97	Rumen(up)
miR-378	55197A	9994B	8847B	1220C	1408C	<0.001	1462.14	Rumen(up)
miR-378b	182.2A	48.8B	38.8B	8.1C	7C	<0.001	5.71	Rumen(up)
miR-378c	2115.8A	362.2B	350.3B	61.2C	95.7C	<0.001	51.77	Rumen(up)
miR-429	2576.7A	1097.8B	1003.3CB	70.2D	820.1C	<0.001	90.13	Rumen(up)
miR-452	22.1A	4.8D	4.7D	6.8C	10.6B	<0.001	0.7	Rumen(up)
miR-6523	9.3A	2.1C	3.3B	2.3C	1.5C	<0.001	0.36	Rumen(up)
miR-93	2398.1A	1133.5B	1170.7B	728.1C	509.9D	<0.001	58.08	Rumen(up)
miR-23a	3014.6A	1000.8B	1041.4B	432.9C	1094.7B	<0.001	91.12	Rumen(up),Liver(down)
miR-24-3p	3344.6A	1659.7B	1479.8B	610.3C	1462.4B	<0.001	200.7	Rumen(up),Liver(down)
miR-708	179.6A	41.5B	46.4B	65.8B	14.7C	<0.001	8.71	Rumen(up),Mammary gland(down)

Table S4. Predicted targets of tissue-dependent differentially expressed microRNAs in rumen, duodenum, jejunum, liver and mammary gland and function categories predominantly targeted by them.

MicroRNA category	Function category	FDR	# Molecules
Rumen-up regulated	Cell Death and Survival	4.62E-36	1411
	Cellular Growth and Proliferation	2.34E-34	1505
	Organismal Survival	4.02E-33	1016
	Cellular Assembly and Organization	6.23E-28	849
	Cellular Function and Maintenance	6.23E-28	1253
	Cell Morphology	8.97E-25	1078
	Gene Expression	2.93E-23	942
	Cellular Movement	5.38E-21	863
	Molecular Transport	6.31E-21	966
	Cardiovascular System Development and Function	2.73E-19	606
	Cellular Development	4.53E-19	1394
Rumen-down regulated	Cell Death and Survival	1.26E-08	513
	Cell Morphology	4.14E-08	417
	Cellular Growth and Proliferation	5.14E-08	546
	Cellular Movement	2.15E-07	321
	Nervous System Development and Function	4.47E-06	325
	Organismal Survival	4.47E-06	336
	Cellular Assembly and Organization	6.31E-06	275
	Cellular Function and Maintenance	6.31E-06	465
	Cellular Compromise	3.45E-05	84
	Tissue Morphology	3.45E-05	330
	Cellular Development	4.63E-05	511
	Gene Expression	5.13E-05	318
Jejunum up-regulated	Cellular Development	0.00807	113
	Cellular Growth and Proliferation	0.00807	127
	Cell Signaling	0.011	55
	Cardiovascular System Development and Function	0.011	56
	Cellular Movement	0.0173	86
	Cell Morphology	0.0173	100
	Organismal Development	0.0242	74
	Cell Death and Survival	0.0294	113
	Tissue Morphology	0.0352	82
	Skeletal and Muscular System Development and Function	0.0383	47
	Tissue Development	0.0383	77
	Nucleic Acid Metabolism	0.0424	9
	Small Molecule Biochemistry	0.0424	44

	Hair and Skin Development and Function	0.0473	22
	Organismal Survival	0.0473	83
	Organ Morphology	0.0476	31
	Cellular Assembly and Organization	0.0492	71
	Cellular Function and Maintenance	0.0492	71
Liver up-regulated	Cell Death and Survival	6.9E-21	961
	Organismal Survival	6.09E-20	706
	Cellular Growth and Proliferation	6.95E-19	1033
	Cell Morphology	3.05E-15	736
	Cellular Assembly and Organization	1.02E-13	572
	Cellular Function and Maintenance	1.02E-13	838
	Cellular Movement	3.27E-13	587
	Molecular Transport	1.97E-12	672
	Cardiovascular System Development and Function	2.78E-11	376
	Organismal Development	2.78E-11	879
	Cellular Development	5.72E-11	920
Liver down-regulated	Cellular Growth and Proliferation	6.67E-22	843
	Organismal Survival	2.38E-21	553
	Cell Morphology	2.37E-16	659
	Cellular Movement	2.37E-16	496
	Cellular Assembly and Organization	2.37E-16	476
	Cellular Function and Maintenance	2.37E-16	715
	Cell Death and Survival	7.71E-16	756
	Molecular Transport	1.85E-12	491
	Organismal Development	1.5E-11	681
	Tissue Morphology	6.78E-11	518
Mammary gland up-regulated	Organismal Survival	1.1E-18	589
	Cell Morphology	1.24E-16	683
	Cellular Growth and Proliferation	6.17E-14	881
	Cell Death and Survival	3.21E-13	800
	Cellular Assembly and Organization	5.07E-12	487
	Cellular Function and Maintenance	5.07E-12	695
	Molecular Transport	2.66E-10	507
	Gene Expression	3.53E-09	516
	Cellular Movement	4.29E-09	502
	Cardiovascular System Development and Function	1.48E-08	335
Mammary gland down-regulated	Organismal Survival	1.31E-09	334
	Cell Death and Survival	8.34E-09	439
	Cellular Growth and Proliferation	0.000000162	482
	Cell Morphology	0.0000176	314
	Cellular Movement	0.0000483	260
	Immune Cell Trafficking	0.0000483	144
	Cellular Function and Maintenance	0.000072	354

	Cellular Assembly and Organization	0.000113	243
	Cellular Development	0.00014	409
	Cardiovascular System Development and Function	0.000141	183
	Tissue Development	0.000141	331
	Hematological System Development and Function	0.000152	248
	Post-Translational Modification	0.000164	142

Table S5. Conservation analysis miRNAs.

MicroRNA	Conservation
let-7a-3p	Poorly conserved
let-7a-5p	Highly conserved
let-7b	Highly conserved
let-7d	Highly conserved
let-7e	Highly conserved
let-7f	Highly conserved
let-7g	Highly conserved
let-7i	Highly conserved
miR-1	Highly conserved
miR-100	Highly conserved
miR-103	Highly conserved
miR-106a	Highly conserved
miR-106b	Highly conserved
miR-107	Highly conserved
miR-1185	Poorly conserved
miR-1246	Poorly conserved
miR-1249	Poorly conserved
miR-125a	Highly conserved
miR-125b	Highly conserved
miR-1260b	Poorly conserved
miR-126-3p	Poorly conserved
miR-126-5p	Poorly conserved
miR-127	Conserved
miR-128	Highly conserved
miR-1291	Poorly conserved
miR-1296	Poorly conserved
miR-1306	Poorly conserved
miR-1307	Poorly conserved
miR-130a	Highly conserved
miR-130b	Highly conserved
miR-132	Highly conserved
miR-133a	Highly conserved
miR-133b	Highly conserved
miR-1343-3p	Poorly conserved
miR-136	Conserved
miR-138	Highly conserved
miR-1388-3p	Poorly conserved
miR-1388-5p	Poorly conserved
miR-140	Poorly conserved
miR-142-3p	Poorly conserved
miR-142-5p	Poorly conserved
miR-143	Highly conserved

miR-1468	Poorly conserved
miR-146b	Highly conserved
miR-148a	Highly conserved
miR-148b	Highly conserved
miR-150	Highly conserved
miR-151-3p	Poorly conserved
miR-151-5p	Poorly conserved
miR-152	Highly conserved
miR-154b	Poorly conserved
miR-155	Highly conserved
miR-15a	Highly conserved
miR-15b	Highly conserved
miR-16a	Highly conserved
miR-16b	Highly conserved
miR-17-3p	Poorly conserved
miR-17-5p	Highly conserved
miR-1814c	Bovine specific
miR-181a	Highly conserved
miR-181b	Highly conserved
miR-181c	Highly conserved
miR-181d	Highly conserved
miR-182	Highly conserved
miR-183	Highly conserved
miR-186	Conserved
miR-188	Poorly conserved
miR-18a	Highly conserved
miR-190b	Highly conserved
miR-191	Highly conserved
miR-192	Highly conserved
miR-193a-5p	Poorly conserved
miR-193b	Highly conserved
miR-194	Highly conserved
miR-195	Highly conserved
miR-197	Conserved
miR-199a-3p	Poorly conserved
miR-199a-5p	Highly conserved
miR-199c	Poorly conserved
miR-19b	Highly conserved
miR-200b	Highly conserved
miR-204	Highly conserved
miR-20a	Highly conserved
miR-214	Highly conserved
miR-215	Highly conserved
miR-21-5p	Highly conserved

miR-218	Highly conserved
miR-219-5p	Highly conserved
miR-221	Highly conserved
miR-222	Highly conserved
miR-2284aa	Bovine specific
miR-2284k	Bovine specific
miR-2284v	Bovine specific
miR-2284w	Bovine specific
miR-2284x	Bovine specific
miR-2284y	Bovine specific
miR-2284z	Bovine specific
miR-2285b	Bovine specific
miR-2285c	Bovine specific
miR-2285e	Bovine specific
miR-2285g	Bovine specific
miR-2285k	Bovine specific
miR-2285o	Bovine specific
miR-2285q	Bovine specific
miR-2285u	Bovine specific
miR-2299-5p	Bovine specific
miR-2310	Bovine specific
miR-2332	Poorly conserved
miR-2339	Bovine specific
miR-24	Poorly conserved
miR-2411-3p	Poorly conserved
miR-2424	Poorly conserved
miR-2443	Bovine specific
miR-2478	Bovine specific
miR-2484	Bovine specific
miR-2487	Bovine specific
miR-25	Highly conserved
miR-26a	Highly conserved
miR-26b	Highly conserved
miR-28	Conserved
miR-2887	Bovine specific
miR-2904	Bovine specific
miR-296	Poorly conserved
miR-29a	Poorly conserved
miR-29b	Highly conserved
miR-29c	Highly conserved
miR-29d	Poorly conserved
miR-301a	Highly conserved
miR-301b	Highly conserved
miR-30a-5p	Highly conserved

miR-30b-3p	Poorly conserved
miR-30b-5p	Highly conserved
miR-30c	Highly conserved
miR-30d	Highly conserved
miR-30e-5p	Highly conserved
miR-30f	Highly conserved
miR-32	Highly conserved
miR-320a	Conserved
miR-326	Conserved
miR-328	Conserved
miR-331	Poorly conserved
miR-335	Conserved
miR-338	Highly conserved
miR-339b	Conserved
miR-33a	Highly conserved
miR-33b	Highly conserved
miR-342	Conserved
miR-3432	Poorly conserved
miR-345-3p	Poorly conserved
miR-345-5p	Poorly conserved
miR-361	Conserved
miR-3613	Poorly conserved
miR-363	Poorly conserved
miR-365-3p	Highly conserved
miR-369-3p	Poorly conserved
miR-369-5p	Poorly conserved
miR-374a	Conserved
miR-374b	Conserved
miR-375	Poorly conserved
miR-376b	Conserved
miR-376e	Conserved
miR-380-3p	Poorly conserved
miR-381	Conserved
miR-411a	Poorly conserved
miR-411c-3p	Bovine specific
miR-421	Conserved
miR-423-3p	Poorly conserved
miR-423-5p	Poorly conserved
miR-424-5p	Highly conserved
miR-425-3p	Poorly conserved
miR-425-5p	Poorly conserved
miR-4286	Poorly conserved
miR-432	Poorly conserved
miR-449a	Highly conserved

miR-450a	Conserved
miR-454	Highly conserved
miR-455-5p	Highly conserved
miR-484	Poorly conserved
miR-486	Conserved
miR-490	Highly conserved
miR-491	Conserved
miR-493	Poorly conserved
miR-494	Conserved
miR-495	Conserved
miR-497	Highly conserved
miR-499	Highly conserved
miR-500	Poorly conserved
miR-502b	Poorly conserved
miR-504	Conserved
miR-532	Poorly conserved
miR-545-3p	Poorly conserved
miR-545-5p	Poorly conserved
miR-582	Poorly conserved
miR-6119-3p	Bovine specific
miR-6119-5p	Bovine specific
miR-6123	Bovine specific
miR-628	Poorly conserved
miR-6517	Bovine specific
miR-652	Poorly conserved
miR-6520	Bovine specific
miR-6524	Bovine specific
miR-6529	Poorly conserved
miR-655	Poorly conserved
miR-665	Poorly conserved
miR-671	Poorly conserved
miR-677	Poorly conserved
miR-7	Highly conserved
miR-744	Poorly conserved
miR-760-3p	Poorly conserved
miR-769	Poorly conserved
miR-877	Poorly conserved
miR-92a	Highly conserved
miR-92b	Highly conserved
miR-95	Poorly conserved
miR-9-5p	Highly conserved
miR-98	Highly conserved
miR-99a-3p	Poorly conserved
miR-99b	Highly conserved

miR-10a	Highly conserved
miR-223	Highly conserved
miR-145	Highly conserved
miR-199b	Highly conserved
miR-200a	Highly conserved
miR-2336	Bovine specific
miR-324	Conserved
miR-34a	Highly conserved
miR-362-5p	Poorly conserved
miR-6120-3p	Bovine specific
miR-101	Highly conserved
miR-144	Highly conserved
miR-154c	Poorly conserved
miR-185	Conserved
miR-22-3p	Highly conserved
miR-22-5p	Poorly conserved
miR-2285f	Bovine specific
miR-339a	Conserved
miR-410	Conserved
miR-411c-5p	Bovine specific
miR-451	Highly conserved
miR-543	Conserved
miR-874	Conserved
miR-487b	Conserved
miR-146a	Highly conserved
miR-455-3p	Poorly conserved
miR-505	Conserved
let-7c	Highly conserved
miR-10b	Highly conserved
miR-141	Highly conserved
miR-1839	Poorly conserved
miR-190a	Highly conserved
miR-200c	Highly conserved
miR-2285t	Bovine specific
miR-340	Poorly conserved
miR-34c	Highly conserved
miR-362-3p	Conserved
miR-379	Conserved
miR-502a	Poorly conserved
miR-6522	Bovine specific
miR-660	Poorly conserved
miR-96	Highly conserved
miR-99a-5p	Highly conserved
miR-139	Highly conserved

miR-2419-5p	Bovine specific
miR-450b	Poorly conserved
miR-574	Poorly conserved
miR-1271	Highly conserved
miR-147	Poorly conserved
miR-149-5p	Conserved
miR-193a-3p	Highly conserved
miR-19a	Highly conserved
miR-210	Poorly conserved
miR-21-3p	Poorly conserved
miR-224	Conserved
miR-23b-3p	Highly conserved
miR-27a-3p	Highly conserved
miR-27a-5p	Poorly conserved
miR-27b	Highly conserved
miR-31	Highly conserved
miR-3431	Poorly conserved
miR-378	Conserved
miR-378b	Poorly conserved
miR-378c	Conserved
miR-429	Highly conserved
miR-452	Poorly conserved
miR-6523	Bovine specific
miR-93	Highly conserved
miR-23a	Highly conserved
miR-24-3p	Highly conserved
miR-708	Conserved
miR-1434-3p	Bovine specific
miR-2284f	Bovine specific
miR-2299-3p	Bovine specific
miR-2320-5p	Poorly conserved
miR-2344	Bovine specific
miR-2349	Bovine specific
miR-2368-3p	Bovine specific
miR-2387	Poorly conserved
miR-2425-5p	Bovine specific
miR-2448-3p	Bovine specific
miR-6121-3p	Bovine specific
miR-615	Poorly conserved
miR-6525	Bovine specific
miR-6527	Bovine specific
miR-216b	Highly conserved
miR-2284p	Bovine specific
miR-2285l	Bovine specific

miR-383	Highly conserved
miR-2320-3p	Poorly conserved
miR-6526	Bovine specific
miR-122	Highly conserved
miR-1247-3p	Poorly conserved
miR-1940	Poorly conserved
miR-2399-5p	Bovine specific
miR-2415-3p	Bovine specific
miR-2483-3p	Poorly conserved
miR-3957	Poorly conserved
miR-433	Conserved
miR-551b	Poorly conserved
miR-6518	Bovine specific
miR-6521	Bovine specific
miR-758	Conserved
miR-1298	Poorly conserved
miR-134	Conserved
miR-196b	Highly conserved
miR-2284j	Bovine specific
miR-376d	Conserved

Table S6. Predicted function categories of top 10 highly expressed bovine specific microRNAs in rumen, duodenum, jejunum, liver and mammary gland.

Tissue	Diseases or Functions Annotation	FDR	# Molecules
	Proliferation Of Cells	1.62E-19	881
	Organismal Death	1.62E-19	618
	Expression Of Rna	4.67E-17	547
	Transcription	1.21E-16	493
	Transcription Of Rna	1.79E-16	485
	Necrosis	1.79E-16	636
	Cancer	3.46E-16	2219
	Cell Death Of Tumor Cell Lines	1.40E-15	402
Rumen	Apoptosis	2.94E-15	645
	Organismal Death	1.34E-18	589
	Proliferation Of Cells	2.41E-16	825
	Expression Of Rna	2.97E-14	511
	Transcription	7.26E-14	460
	Transcription Of Rna	1.33E-13	452
	Cancer	1.36E-13	2097
	Neoplasia Of Epithelial Tissue	2.27E-13	1772
	Epithelial Cancer	8.27E-13	1737
	Solid Tumor	1.05E-12	2027
Duodenum	Cell Death Of Tumor Cell Lines	1.53E-12	372
	Organismal Death	1.34E-18	589
	Proliferation Of Cells	2.41E-16	825
	Expression Of Rna	2.97E-14	511
	Transcription	7.26E-14	460
	Transcription Of Rna	1.33E-13	452
	Cancer	1.36E-13	2097
	Neoplasia Of Epithelial Tissue	2.27E-13	1772
	Epithelial Cancer	8.27E-13	1737
	Solid Tumor	1.05E-12	2027
Jejunum	Cell Death Of Tumor Cell Lines	1.53E-12	372
	Organismal Death	2.23E-16	640
	Cancer	2.99E-16	2370
	Malignant Solid Tumor	1.39E-15	2277
	Proliferation Of Cells	1.94E-15	909
	Solid Tumor	3.60E-15	2290
	Epithelial Cancer	4.32E-14	1955
	Neoplasia Of Epithelial Tissue	8.34E-14	1988
	Necrosis	2.42E-13	656
	Cell Death	1.73E-12	823
Liver	Expression Of Rna	4.31E-12	551
Mammary gland	Organismal Death	3.60E-18	630

Proliferation Of Cells	1.41E-14	878
Malignant Solid Tumor	1.41E-14	2197
Epithelial Cancer	1.41E-14	1896
Cancer	1.42E-14	2282
Solid Tumor	3.56E-14	2209
Neoplasia Of Epithelial Tissue	3.56E-14	1926
Necrosis	7.71E-13	634
Cell Death Of Tumor Cell Lines	1.61E-12	399
Cell Death	2.44E-12	797
Expression Of Rna	9.32E-12	533

Table S7. Correlation between feed efficiency and microRNAs in dairy rumen, duodenum, jejunum, liver, and mammary gland under different diets.

			Phenotype (P<0.05)				AL vs RS		RS vs CS		AL vs CS		
			Feed			Positive/negative							
	Di		efficien	Nitrogen	Milk urea	associated with		log2(folde		log2(folde		log2(folde	
Tissue	et	miRNA	cy	efficiency	nitrogen	traits	P	r change)	P	r change)	P	r change)	
	A	miR-66		0.860195			0.1		0.6		0.2		
Rumen	L	0	-	741	-	Positive	2	-0.20	3	0.03	3	-0.17	
	A	miR-23			0.815858		0.8		0.8		0.9		
Rumen	L	32	-	-	345	Positive	5	0.20	0	-0.23	9	-0.02	
	A	miR-24			0.819167		0.8		0.8		0.9		
Rumen	L	11-3p	-	-	9	Positive	3	0.21	4	-0.14	4	0.07	
	A	miR-34			0.821346		0.0		0.6		0.1		
Rumen	L	5-3p	-	-	631	Positive	4	-0.50	8	0.08	2	-0.43	
	A	miR-22			0.845071		0.6		0.6		0.5		
Rumen	L	84x	-	-	236	Positive	4	0.10	2	0.04	3	0.14	
	A	miR-37			0.872258		0.7		0.1		0.3		
Rumen	L	8b	-	-	971	Positive	5	0.08	2	-0.32	6	-0.24	Rumen(Up)
	A	miR-37			0.961157		0.2		0.1		0.0		
Rumen	L	8	-	-	147	Positive	7	-0.19	6	-0.26	3	-0.46	Rumen(Up)
	A	miR-13	0.83866	0.816061			0.0		0.1		0.6		
Rumen	L	0a	3266	23	-	Positive	3	-0.28	3	0.20	6	-0.08	
	A	miR-26		0.822689			0.1		0.1		0.8		
Rumen	L	b	-	925	-	Positive	3	-0.23	1	0.20	8	-0.03	
	A	miR-16		0.836281			0.0		0.7		0.0		
Rumen	L	b	-	83	-	Positive	7	-0.34	2	-0.04	7	-0.39	
Rumen	A	miR-12	0.87180	0.852690	-	Positive	0.3	-0.21	0.3	0.18	0.8	-0.03	

	L	6-5p	2328	754			2		5		9		
	A	miR-22		0.853611			0.0		0.8		0.0		Rumen(Speci
Rumen	L	99-3p	-	268	-	Positive	9	-0.83	4	0.00	6	-0.83	fic)
	A	miR-61		0.854564			0.1		0.4		0.0		
Rumen	L	23	-	557	-	Positive	4	-0.27	1	-0.15	8	-0.42	
	A	miR-18		0.862239			0.0		0.3		0.0		
Rumen	L	1d	-	651	-	Positive	6	-0.26	3	0.12	8	-0.14	
	A	miR-23	0.93358	0.862844			0.5		0.0		0.0		
Rumen	L	76	3849	117	-	Positive	7	0.21	3	-0.82	7	-0.62	
	A	miR-61		0.866912			0.0		0.6		0.0		
Rumen	L	19-5p	-	636	-	Positive	6	-0.55	7	0.07	5	-0.48	
	A	miR-65		0.931872			0.7		0.9		0.7		
Rumen	L	2	-	658	-	Positive	2	-0.11	8	-0.01	5	-0.12	
	A	miR-33	0.81016				0.6		0.7		0.9		
Rumen	L	5	6749	-	-	Positive	9	-0.10	3	0.09	8	-0.01	
	A	miR-19	0.81936				0.9		0.2		0.2		
Rumen	L	2	2592	-	-	Positive	0	0.02	5	-0.18	2	-0.16	
	A	miR-61	0.86295				0.0		0.0		0.3		
Rumen	L	20-3p	8742	-	-	Positive	1	-0.64	4	0.42	5	-0.22	
	A	miR-23	0.94363				0.3		0.9		0.4		
Rumen	L	46	1573	-	-	Positive	1	-0.49	1	0.04	5	-0.44	
	A	miR-48			-0.827917		0.2		0.3		0.8		
Rumen	L	6	-	-	489	Negative	2	0.66	0	-0.58	7	0.08	
	A	miR-48			-0.819711		0.5		0.2		0.7		
Rumen	L	4	-	-	329	Negative	5	0.12	0	-0.19	3	-0.08	
	A	miR-12	-0.8213	-0.922619			0.7		0.5		0.3		
Rumen	L	96	24046	737	-	Negative	6	-0.07	2	-0.11	1	-0.18	
Rumen	A	miR-23	-	-0.914016	-	Negative	0.2	1.24	0.1	-0.71	0.5	0.53	Rumen(Speci

	L	87		374				3		1		1		fic)
	A	miR-37		-0.844973				0.2		0.9		0.3		
Rumen	L	9	-	1	-		Negative	5	1.09	3	-0.07	2	1.02	
	A			-0.827354				0.1		0.0		0.9		
Rumen	L	miR-7	-	199	-		Negative	8	0.54	3	-0.50	1	0.03	
	A	miR-24		-0.822645				0.3		0.7		0.3		Rumen(Speci
Rumen	L	25-5p	-	303	-		Negative	1	1.06	7	-0.13	7	0.94	fic)
	A	miR-49		-0.819513				0.7		0.6		0.9		
Rumen	L	1	-	06	-		Negative	1	0.17	3	-0.17	8	0.00	
	A			-0.818840				0.3		0.7		0.4		
Rumen	L	miR-1	-	46	-		Negative	6	1.40	0	-0.24	0	1.16	
	C	miR-13				0.850884		0.0		0.3		0.0		
Rumen	S	0b	-	-		72	Positive	0	-0.77	9	-0.18	1	-0.95	
	C	miR-30				0.858901		0.1		0.1		0.6		
Rumen	S	a-5p	-	-		214	Positive	8	0.23	6	-0.15	3	0.08	
	C	miR-13		0.816963				0.0		0.2		0.1		
Rumen	S	8	-	946	-		Positive	2	-1.03	6	0.42	7	-0.62	
	C	miR-30		0.829365				0.0		0.2		0.1		
Rumen	S	1a	-	703	-		Positive	4	-0.40	6	0.11	2	-0.28	
	C			0.831316				0.0		0.6		0.0		
Rumen	S	miR-93	-	326	-		Positive	2	-0.39	9	0.05	3	-0.34	Rumen(Up)
	C	miR-87		0.840341				0.0		0.4		0.2		
Rumen	S	7	-	938	-		Positive	5	-0.56	5	0.18	1	-0.38	
	C	miR-10		0.854437				0.0		0.1		0.0		
Rumen	S	3	-	391	-		Positive	0	-0.49	3	0.13	3	-0.36	
	C	miR-22		0.856305				0.2		0.4		0.1		
Rumen	S	84b	-	204	-		Positive	7	0.24	7	0.29	4	0.53	
Rumen	C	miR-54	-	0.861168	-		Positive	0.8	0.10	0.2	0.27	0.3	0.37	

	S	5-3p		765			1		4		7		
	C	miR-10		0.868992			0.0		0.6		0.1		
Rumen	S	7	-	043	-	Positive	2	-0.35	3	0.06	2	-0.29	
	C	miR-33		0.872153			0.6		0.7		0.9		
Rumen	S	5	-	436	-	Positive	9	-0.10	3	0.09	8	-0.01	
	C	miR-22		0.926048			0.6		0.6		0.5		
Rumen	S	84x	-	841	-	Positive	4	0.10	2	0.04	3	0.14	
	C	miR-14					0.2		0.2		0.7		
Rumen	S	7	-	0.937943	-	Positive	0	-0.24	2	0.18	2	-0.06	Rumen(Up)
	C	miR-58		0.956953			0.1		0.8		0.4		
Rumen	S	2	-	612	-	Positive	8	0.15	4	-0.03	8	0.13	
	C	miR-45	0.81850				0.1		0.0		0.4		
Rumen	S	5-5p	538	-	-	Positive	9	-0.22	3	0.34	8	0.12	
	C	miR-24	0.85445				0.0		0.0		0.7		
Rumen	S	31-3p	3352	-	-	Positive	5	1.10	0	-1.00	3	0.10	
	C	miR-14	0.86704				0.0		0.3		0.0		
Rumen	S	0	6385	-	-	Positive	4	-0.45	0	0.13	7	-0.32	
	C	miR-22	0.90612				0.0		0.3		0.0		
Rumen	S	84y	1668	-	-	Positive	5	1.10	4	0.13	4	1.23	
	C	miR-29	0.92281				0.6		0.0		0.4		
Rumen	S	c	1064	-	-	Positive	8	-0.09	3	0.27	6	0.18	
	C	miR-36	0.92825				0.2		0.1		0.7		
Rumen	S	2-3p	7749	-	-	Positive	1	-0.45	1	0.34	8	-0.11	
	C	miR-12			-0.940310		0.0		0.5		0.2		
Rumen	S	49	-	-	531	Negative	7	-0.62	0	0.17	2	-0.45	
	C	miR-29			-0.925167		0.2		0.2		1.0		
Rumen	S	04	-	-	695	Negative	4	-0.59	2	0.59	0	0.00	
Rumen	C	miR-28	-	-	-0.877797	Negative	0.0	-1.66	0.2	0.61	0.0	-1.05	

	S	87			315		5		7		8		
	C	miR-41			-0.841724		0.5		0.4		0.6		
Rumen	S	1c-3p	-	-	437	Negative	7	-0.42	0	0.68	7	0.26	
	C	miR-50			-0.836241		0.0		0.6		0.2		
Rumen	S	4	-	-	525	Negative	1	-0.68	7	0.12	0	-0.56	
	C	miR-12			-0.832971		0.0		0.0		0.9		
Rumen	S	60b	-	-	249	Negative	2	-0.75	1	0.72	0	-0.03	
	C	miR-37			-0.830032		0.2		0.5		0.5		
Rumen	S	6e	-	-	367	Negative	9	-0.68	5	0.34	9	-0.34	
	C	miR-37			-0.820203		0.2		0.9		0.3		
Rumen	S	9	-	-	364	Negative	5	1.09	3	-0.07	2	1.02	
	C	miR-33			-0.813215		0.0		0.1		0.0		
Rumen	S	1	-	-	458	Negative	0	-1.06	1	0.34	1	-0.72	
	C	miR-10		-0.904270			0.1		0.9		0.1		
Rumen	S	a	-	768	-	Negative	1	0.41	5	0.01	5	0.42	
	C	miR-27		-0.901282			0.0		0.2		0.0		
Rumen	S	b	-	071	-	Negative	1	0.30	3	-0.11	7	0.19	Rumen(Up)
	C	miR-23		-0.868055			0.7		0.7		0.5		Rumen(Speci
Rumen	S	49	-	792	-	Negative	9	0.11	9	0.12	9	0.22	fic)
	C	miR-14		-0.859660			0.2		0.3		0.0		
Rumen	S	6a	-	962	-	Negative	7	-0.37	0	-0.22	6	-0.60	
	C	miR-99	-0.8812	-0.829890			0.0		0.3		0.0		
Rumen	S	b	04568	824	-	Negative	2	2.40	4	-0.17	2	2.23	
	C	miR-22	-0.9664				0.0		0.4		0.3		
Rumen	S	3	19612	-	-	Negative	4	-1.39	7	0.45	1	-0.94	
	C	miR-19	-0.9614				0.1		0.0		0.5		
Rumen	S	1	99601	-	-	Negative	1	0.49	6	-0.34	9	0.15	
Rumen	C	miR-12	-0.9398	-	-	Negative	0.0	4.25	0.2	-0.21	0.0	4.04	Rumen(Up)

	S	71	44159				2		1		2		
	C	miR-14	-0.9309				0.0		0.3		0.0		
Rumen	S	6b	24203	-	-	Negative	6	-0.53	3	-0.32	4	-0.85	
	C	miR-14	-0.9116				0.2		0.0		0.9		
Rumen	S	8b	80821	-	-	Negative	0	0.22	1	-0.23	4	-0.01	
	C	miR-30	-0.8616				0.5		0.1		0.6		
Rumen	S	b-3p	71238	-	-	Negative	7	0.26	9	-0.42	6	-0.15	
	C	miR-22	-0.8466				0.0		0.0		0.1		
Rumen	S	85k	37693	-	-	Negative	2	1.30	4	-0.71	7	0.59	
	C	miR-22	-0.8283				0.0		0.0		0.8		
Rumen	S	85f	70327	-	-	Negative	1	0.48	0	-0.44	0	0.04	
	R	miR-36			0.813394		0.4		0.6		0.7		
Rumen	S	9-5p	-	-	167	Positive	1	-0.63	3	0.33	0	-0.30	
	R	miR-22			0.816177		0.0		0.8		0.0		Rumen(Speci
Rumen	S	99-3p	-	-	357	Positive	9	-0.83	4	0.00	6	-0.83	fic)
	R	miR-50			0.833421		0.0		0.6		0.2		
Rumen	S	4	-	-	003	Positive	1	-0.68	7	0.12	0	-0.56	
	R	miR-30			0.836946		0.5		0.1		0.6		
Rumen	S	b-3p	-	-	142	Positive	7	0.26	9	-0.42	6	-0.15	
	R	miR-21			0.838352		0.2		0.0		0.0		
Rumen	S	1	-	-	434	Positive	6	0.83	3	-2.15	9	-1.31	
	R	miR-24			0.846324		0.7		0.5		0.4		
Rumen	S	74	-	-	225	Positive	5	-0.07	0	-0.43	0	-0.51	
	R	miR-14			0.945421		0.0		0.0		0.3		
Rumen	S	68	-	-	919	Positive	5	0.66	7	-0.38	5	0.27	
	R	miR-24		0.810343			0.6		0.0		0.2		Rumen(Dow
Rumen	S	19-5p	-	213	-	Positive	0	0.23	9	-0.62	6	-0.39	n)
Rumen	R	miR-36	0.96587	0.842919	-	Positive	0.2	0.34	0.2	-0.16	0.4	0.18	

	S	1	9926	341			0		1		9		
	R	miR-50		0.856407			0.3		0.1		0.9		
Rumen	S	2a	-	014	-	Positive	2	-0.51	9	0.58	5	0.07	
	R	miR-32		0.878410			0.0		0.7		0.0		
Rumen	S	8	-	199	-	Positive	4	0.46	2	-0.06	8	0.40	
	R	miR-10	0.81986	0.938065			0.6		0.0		0.7		
Rumen	S	1	3872	246	-	Positive	3	-0.16	1	0.30	0	0.14	
	R	miR-32	0.87392				0.0		0.4		0.3		
Rumen	S	4	4823	-	-	Positive	7	-0.41	1	0.19	9	-0.22	
	R	miR-18	0.87822				0.0		0.2		0.1		
Rumen	S	5	9456	-	-	Positive	3	0.77	6	-0.29	5	0.47	
	R	miR-15	0.89868				0.0		0.2		0.0		
Rumen	S	3	572	-	-	Positive	2	1.58	0	-0.42	5	1.17	
	R	miR-30	0.92720				0.8		0.3		0.7		
Rumen	S	f	6075	-	-	Positive	8	0.04	6	-0.12	7	-0.07	
	R	miR-24			-0.942249		0.1		0.3		0.4		
Rumen	S	31-5p	-	-	645	Negative	5	-0.58	8	0.26	4	-0.32	
	R	miR-22			-0.939555		0.0		0.6		0.0		
Rumen	S	85p	-	-	912	Negative	2	0.80	6	-0.12	7	0.68	
	R	miR-70			-0.881009		0.6		0.7		0.4		
Rumen	S	8	-	-	526	Negative	3	0.17	1	0.12	6	0.29	Rumen(Up)
	R	miR-42			-0.871909		0.1		0.0		0.2		
Rumen	S	5-3p	-	-	428	Negative	2	0.80	3	-0.29	6	0.51	
	R	miR-19			-0.870687		0.7		0.6		0.9		
Rumen	S	b	-	-	341	Negative	7	-0.12	1	0.13	8	0.01	
	R	miR-34			-0.851278		0.6		0.3		0.8		
Rumen	S	31	-	-	91	Negative	1	-0.13	2	0.09	8	-0.04	Rumen(Up)
Rumen	R	miR-18	-	-	-0.847735	Negative	0.1	1.06	0.1	-0.21	0.2	0.85	

	S	3			496		4		3		0		
	R	miR-22			-0.847059		0.8		0.6		0.7		
Rumen	S	84n	-	-	822	Negative	8	-0.13	7	0.28	8	0.14	
	R	miR-23			-0.846559		0.8		0.8		0.9		
Rumen	S	32	-	-	414	Negative	5	0.20	0	-0.23	9	-0.02	
	R	miR-18			-0.837302		0.7		0.7		0.9		
Rumen	S	1c	-	-	202	Negative	8	-0.05	5	0.04	6	-0.01	
	R	miR-14			-0.836072		0.2		0.0		0.9		
Rumen	S	8b	-	-	775	Negative	0	0.22	1	-0.23	4	-0.01	
	R	miR-20			-0.811624		0.1		0.6		0.3		
Rumen	S	5	-	-	777	Negative	5	0.29	3	-0.09	5	0.20	
	R	miR-65		-0.907990			0.3		0.9		0.4		
Rumen	S	24	-	21	-	Negative	8	-0.15	9	0.00	1	-0.16	
	R	miR-14		-0.858744			0.5		0.4		0.9		
Rumen	S	9-5p	-	213	-	Negative	0	-0.20	0	0.22	3	0.02	Rumen(Up)
	R	miR-18		-0.847369			0.3		0.8		0.5		
Rumen	S	b	-	955	-	Negative	9	0.62	4	-0.09	1	0.52	
	R	miR-21		-0.846933			0.0		0.7		0.0		
Rumen	S	-3p	-	456	-	Negative	3	-0.75	7	-0.06	1	-0.81	Rumen(Up)
	R	miR-15		-0.839930			0.0		0.0		0.0		
Rumen	S	5	-	009	-	Negative	1	-1.01	9	0.26	4	-0.75	
	R	miR-14		-0.836764			0.0		0.0		0.0		
Rumen	S	2-3p	-	737	-	Negative	2	-0.71	4	0.19	7	-0.52	
	R			-0.835139			0.3		0.8		0.4		
Rumen	S	miR-25	-	285	-	Negative	3	-0.08	8	-0.02	5	-0.10	
	R	miR-22		-0.827572			0.6		0.3		0.5		
Rumen	S	84aa	-	6	-	Negative	4	-0.09	5	0.19	3	0.10	
Rumen	R	miR-14	-	-0.813965	-	Negative	0.0	-0.53	0.3	-0.32	0.0	-0.85	

	S	6b			151		6		3		4		
Rumen	RS	miR-1434-3p	-0.844856017	-	-	Negative	0.40	-0.45	0.85	0.10	0.43	-0.35	Rumen(Specific)
Rumen	RS	miR-103	-0.826927141	-	-	Negative	0.00	-0.49	0.13	0.13	0.03	-0.36	
Duodenum	AL	miR-2443	-	-	0.810982665	Positive	0.5897	-0.2410081	0.515	0.2410081	0.8885	0	
Duodenum	AL	miR-23b-3p	-	-	0.812296138	Positive	0.1025	0.209836795	0.7126	-0.052327543	0.1602	0.157509251	
Duodenum	AL	miR-190a	-	-	0.812982074	Positive	0.9356	0.069041644	0.309	0.833221554	0.3145	0.902263198	
Duodenum	AL	miR-342	-	-	0.813453674	Positive	0.6331	0.074989375	0.128	-0.233397794	0.0997	-0.158408419	
Duodenum	AL	miR-219-5p	-	-	0.814748819	Positive	0.684	0.106915204	0.2205	-0.621488377	0.311	-0.514573173	
Duodenum	AL	miR-365-3p	-	-	0.814758008	Positive	0.5814	-0.094068324	0.1925	0.291105171	0.3911	0.197036847	
Duodenum	AL	miR-22-3p	-	-	0.816938566	Positive	0.3515	-0.153320778	0.0348	0.330241345	0.3122	0.176920568	
Duodenum	AL	miR-885	-	-	0.822157502	Positive	0.289	-0.415037499	0.711	0.125530882	0.493	-0.289506617	

							7		8			
Duodenum	AL	miR-3613	-	-	0.82251826	Positive	0.9155	0.020037753	0.6859	-0.068947354	0.7083	-0.0489096
Duodenum	AL	miR-374b	-	-	0.8228229	Positive	0.178	0.160663432	0.3288	-0.107903998	0.5388	0.052759434
Duodenum	AL	miR-6119-3p	-	-	0.823510652	Positive	0.5685	-0.116644919	0.4899	0.101538026	0.9524	-0.015106892
Duodenum	AL	miR-29d	-	-	0.825596822	Positive	0.1655	-0.349334252	0.2088	0.27237227	0.7317	-0.076961982
Duodenum	AL	miR-188	-	-	0.829384432	Positive	0.4848	-0.185555653	0.2048	0.303392143	0.6338	0.11783649
Duodenum	AL	miR-324	-	-	0.830851898	Positive	0.5299	0.144389909	0.9573	0.012711052	0.4435	0.157100961
Duodenum	AL	miR-152	-	-	0.833766091	Positive	0.4974	0.11376511	0.6965	-0.078838518	0.8326	0.034926592
Duodenum	AL	miR-2376	-	-	0.837978483	Positive	0.2003	-0.289506617	0.6425	0.211504105	0.8421	-0.078002512
Duodenum	AL	miR-214	-	-	0.840868146	Positive	0.9726	0.006614844	0.6617	0.084647826	0.5623	0.09126267

Duodenum	AL	miR-3432	-	-	0.843690338	Positive	0.6345	-0.095280503	0.212	0.307089586	0.1669	0.211809082
Duodenum	AL	let-7a-5p	-	-	0.845262454	Positive	0.547	-0.100447265	0.0288	0.340437417	0.0932	0.239990152
Duodenum	AL	miR-2903	-	-	0.845272175	Positive	0.8785	0	0.6269	0.125530882	0.7729	0.125530882
Duodenum	AL	miR-92a	-	-	0.847287831	Positive	0.0796	-0.264781707	0.0012	0.427462509	0.238	0.162680802
Duodenum	AL	miR-15a	-	-	0.850055995	Positive	0.1246	-0.245350515	0.5371	0.103744167	0.3426	-0.141606348
Duodenum	AL	miR-200c	-	-	0.850683517	Positive	0.2802	0.229560939	0.0781	-0.286829281	0.7845	-0.057268342
Duodenum	AL	let-7a-3p	-	-	0.852091371	Positive	0.5922	-0.091895866	0.3908	0.133462359	0.8188	0.041566493
Duodenum	AL	miR-150	-	-	0.852273189	Positive	0.3529	-0.307011582	0.8159	-0.114130399	0.3974	-0.421141981
Duodenum	AL	miR-154b	-	-	0.854291492	Positive	0.79	-0.087462841	0.9352	-0.08246216	0.7881	-0.169925001
Duoden	A	miR-36	-	-	0.857537	Positive	0.7	-0.039892	0.9	-0.013056	0.6	-0.052948

um	L	2-3p			303		48	727	30	153	77	88
							4		6		6	
							0.6		0.5			
Duoden	A	miR-45			0.862009		51	-0.078297	27	0.104735	0.8	0.026437
um	L	5-3p	-	-	356	Positive	7	934	7	494	43	56
							0.8		0.0		0.0	
Duoden	A				0.864873		31	-0.036536	81	0.303729	73	0.267192
um	L	let-7b	-	-	631	Positive	9	155	6	076	7	921
							0.9		0.2		0.2	
Duoden	A				0.865617		96	0.000509	18	0.141408	35	0.141918
um	L	let-7g	-	-	627	Positive	9	172	2	898	6	07
							0.6				0.5	
Duoden	A	miR-10			0.866894		27	0.072491	0.1	-0.162659	44	-0.090167
um	L	6b	-	-	296	Positive	5	871	67	362	3	491
							0.2		0.4		0.8	
Duoden	A	miR-27			0.868068		72	-0.136131	07	0.114846	37	-0.021285
um	L	a-3p	-	-	927	Positive	8	483	4	232	8	251
											0.9	
Duoden	A				0.871626		0.7	-0.034309	0.7	0.024441	23	-0.009867
um	L	miR-93	-	-	533	Positive	12	144	26	238	6	906
							0.3		0.9		0.3	
Duoden	A	miR-17			0.873529		43	-0.135959	96		57	-0.135959
um	L	-3p	-	-	074	Positive	6	709	5	0	2	709
									0.2		0.1	
Duoden	A	miR-15			0.874984		0.9	0.034194	81	-0.377282	29	-0.343087
um	L	3	-	-	947	Positive	17	92	3	577	7	657
Duoden	A	miR-22			0.878602		0.3	0.169925	0.3	-0.321928	0.6	-0.152003
um	L	84t-3p	-	-	664	Positive	85	001	45	095	38	093

						5		8		6	
Duodenum	AL	miR-6523	-	-	0.882800251	Positive	0.0717	0.337034987	0.2281	-0.211504105	0.4187	0.125530882
Duodenum	AL	miR-542-5p	-	-	0.885762694	Positive	0.1651	-0.374395515	0.8542	0.041820176	0.1748	-0.332575339
Duodenum	AL	miR-186	-	-	0.887670904	Positive	0.6808	-0.03653037	0.6367	0.042357565	0.9387	0.005827195
Duodenum	AL	miR-410	-	-	0.88787233	Positive	0.9755	-0.004274655	0.2441	0.225953754	0.2045	0.221679099
Duodenum	AL	miR-877	-	-	0.891740562	Positive	0.148	-0.464403577	0.0117	0.790904401	0.2366	0.326500825
Duodenum	AL	miR-30c	-	-	0.892497828	Positive	0.1374	-0.175530175	0.4508	0.078948273	0.4147	-0.096581902
Duodenum	AL	miR-30b-5p	-	-	0.893313505	Positive	0.8196	-0.039946286	0.3008	0.150182457	0.4353	0.110236171
Duodenum	AL	miR-328	-	-	0.893999514	Positive	0.0401	-0.669354688	0.0363	0.669354688	0.9766	0
Duodenum	AL	miR-221	-	-	0.900193569	Positive	0.3819	0.117335281	0.5391	-0.053860648	0.6708	0.063474633

Duodenum	AL	miR-200b	-	-	0.902021907	Positive	0.7435	-0.046606768	0.6138	-0.07545401	0.4105	-0.122060778
Duodenum	AL	miR-1296	-	-	0.904171252	Positive	0.1068	-0.432959407	0.2276	0.231325546	0.3883	-0.201633861
Duodenum	AL	miR-505	-	-	0.905423573	Positive	0.7767	-0.05508756	0.4463	0.116863758	0.7234	0.061776198
Duodenum	AL	miR-30b-3p	-	-	0.90617243	Positive	0.571	-0.222392421	0.4783	0.347923303	0.7199	0.125530882
Duodenum	AL	miR-193a-5p	-	-	0.906311928	Positive	0.9718	0	0.4157	0.236067358	0.3124	0.236067358
Duodenum	AL	miR-1306	-	-	0.90920563	Positive	0.3597	-0.325400287	0.3287	0.257496222	0.8052	-0.067904065
Duodenum	AL	miR-34a	-	-	0.912161491	Positive	0.0085	0.329722252	0.0766	-0.351936986	0.8859	-0.022214734
Duodenum	AL	miR-574	-	-	0.913736896	Positive	0.7232	-0.082819528	0.245	0.17410728	0.6953	0.091287752
Duodenum	AL	miR-210	-	-	0.915042284	Positive	0.2357	-0.224462285	0.0929	0.276124405	0.8121	0.05166212
Duoden	A	miR-44	-	-	0.916034	Positive	0.8	0.061400	0.6	0.131244	0.4	0.192645

um	L	9a			326		905	545	398	533	039	078
Duodenum	AL	miR-423-3p	-	-	0.918202096	Positive	0.1652	-0.460751098	0.0679	0.592585536	0.6169	0.131834439
Duodenum	AL	miR-652	-	-	0.92009286	Positive	0.9602	-0.008752474	0.2971	0.172836597	0.2199	0.164084123
Duodenum	AL	miR-423-5p	-	-	0.922849551	Positive	0.0341	-0.399520581	0.0617	0.29755622	0.6024	-0.101964361
Duodenum	AL	miR-339a	-	-	0.924549264	Positive	0.1106	-0.292747393	0.2072	0.193548444	0.4855	-0.099198949
Duodenum	AL	miR-497	-	-	0.926215204	Positive	0.189	-0.372564401	0.1683	0.416247734	0.7772	0.043683333
Duodenum	AL	miR-222	-	-	0.930944539	Positive	0.1397	-0.24894747	0.2847	0.131019018	0.5448	-0.117928451
Duodenum	AL	miR-769	-	-	0.936235135	Positive	0.2399	-0.145157096	0.1057	0.224823248	0.4878	0.079666151
Duodenum	AL	miR-23a	-	-	0.940364759	Positive	0.5738	-0.07234678	0.9216	-0.013874897	0.3224	-0.086221677
Duodenum	AL	miR-1249	-	-	0.940664233	Positive	0.203	-0.493332025	0.074	0.596836601	0.711	0.103504576

							6		8		4	
Duodenum	AL	miR-1307	-	-	0.941824408	Positive	0.0519	-0.449856477	0.0062	0.545968369	0.6701	0.096111893
Duodenum	AL	miR-29b	-	-	0.941957783	Positive	0.2503	0.152549465	0.4647	-0.094019909	0.5623	0.058529556
Duodenum	AL	miR-339b	-	-	0.942487774	Positive	0.1177	-0.272569768	0.0907	0.254317189	0.8789	-0.018252579
Duodenum	AL	miR-345-3p	-	-	0.950959897	Positive	0.727	0.04662616	0.0257	0.265142069	0.0541	0.311768229
Duodenum	AL	miR-199b	-	-	0.952199411	Positive	0.035	-0.304233097	0.0916	0.281856556	0.8747	-0.022376541
Duodenum	AL	miR-149-5p	-	-	0.955298435	Positive	0.349	-0.174833802	0.0137	0.436099115	0.1865	0.261265313
Duodenum	AL	let-7d	-	-	0.959253788	Positive	0.9613	0.008460075	0.7484	0.046508866	0.717	0.054968941
Duodenum	AL	miR-654	-	-	0.96239023	Positive	0.0831	0.5360529	0.1997	-0.5360529	0.951	0
Duodenum	AL	miR-320a	-	-	0.970013577	Positive	0.11	-0.290571415	0.0833	0.342186934	0.7467	0.051615518

							0.7		0.9		0.7	
Duoden	A	miR-15			0.971892		93	0.052578	45	0.010124	49	0.062702
um	L	b	-	-	256	Positive	6	281	1	217	9	499
									0.0			
Duoden	A	miR-33			0.973661		0.1	-0.385834	65	0.413633	0.8	0.027798
um	L	1	-	-	571	Positive	36	964	9	416	73	452
							0.3		0.3			
Duoden	A	miR-12			0.975771		01	-0.193413	26	0.200603	0.9	0.007190
um	L	8	-	-	081	Positive	9	354	3	733	75	378
							0.2		0.4		0.8	
Duoden	A				0.978838		91	-0.081355	57	0.065326	85	-0.016028
um	L	miR-25	-	-	568	Positive	9	299	1	725	3	574
							0.4		0.8		0.4	
Duoden	A	miR-67			0.979122		98	0.222392	75	0.026967	38	0.249359
um	L	1	-	-	836	Positive	1	421	9	048	5	469
							0.7		0.5		0.7	
Duoden	A	miR-26			0.986191		37	-0.036859	34	0.063125	09	0.026266
um	L	b	-	-	707	Positive	2	449	4	451	3	001
							0.3		0.1		0.8	
Duoden	A	miR-76			0.988567		61	-0.351472	80	0.402098	89	0.050626
um	L	0-3p	-	-	746	Positive	8	371	1	444	8	073
							0.2		0.6		0.3	
Duoden	A	miR-48			0.990642		32	-0.272969	01	0.110728	86	-0.162241
um	L	4	-	-	616	Positive	2	959	4	477	3	482
							0.0		0.1		0.8	
Duoden	A	miR-23	0.87279	0.842397			09	-0.261570	24	0.238347	82	-0.023222
um	L	36	6746	165	-	Positive	8	484	7	527	3	957
Duoden	A	miR-22	0.90800	0.844005	-	Positive	0.0	1	0.0	-0.652076	0.3	0.347923

um	L	85l	7997	417			56		76	697	93	303
							2		2		6	
							0.1		0.7		0.1	
Duoden	A	miR-24	0.97524	0.873785			79	-0.700439	61	-0.206450	39	-0.906890
um	L	83-5p	8074	105	-	Positive	1	718	6	877	5	596
							0.5				0.9	
Duoden	A	miR-37		0.908119			94	0.209718	0.5	-0.243271	34	-0.033552
um	L	9	-	303	-	Positive	6	591	54	151	7	56
							0.2		0.3		0.6	
Duoden	A		0.81262				34	0.354108	92	-0.255784	93	0.098323
um	L	miR-7	237	-	-	Positive	1	501	9	667	7	834
							0.5		0.1		0.4	
Duoden	A	miR-22	0.81366				37	-0.321928	54	0.862496	31	0.540568
um	L	85i	7379	-	-	Positive	3	095	4	476	2	381
							0.7		0.9		0.8	
Duoden	A	miR-48	0.84237				86	-0.070389	37	0.027744	87	-0.042644
um	L	7b	1752	-	-	Positive	2	328	2	99	5	337
							0.0		0.3		0.8	
Duoden	A	miR-12	0.89126				92	0.259895	53	-0.306476	67	-0.046580
um	L	7	4436	-	-	Positive	3	862	9	467	6	605
							0.4		0.8		0.7	
Duoden	A	miR-65	0.93357				40	0.175086	15	-0.090197	65	0.084888
um	L	5	9115	-	-	Positive	9	707	2	809	9	898
							0.1		0.2		0.9	
Duoden	A	miR-48			-0.969074		26	-0.265358	53	0.279840	40	0.014481
um	L	6	-	-	577	Negative	5	975	7	352	3	377
Duoden	A	miR-10			-0.906889		0.2	-0.395545	0.1	0.521094	0.4	0.125549
um	L	0	-	-	981	Negative	30	483	48	892	85	409

							9		5		1	
Duodenum	AL	miR-10b	-	-	-0.884770039	Negative	0.5216	0.121049776	0.721	-0.092438719	0.8958	0.028611057
Duodenum	AL	miR-124a	-	-	-0.875522747	Negative	0.8985	-0.079226691	0.6884	0.278535499	0.6602	0.199308808
Duodenum	AL	miR-124b	-	-	-0.875522747	Negative	0.8985	-0.079226691	0.6884	0.278535499	0.6602	0.199308808
Duodenum	AL	miR-215	-	-	-0.82500821	Negative	0.6038	0.224443388	0.1417	-0.578122883	0.1424	-0.353679495
Duodenum	AL	miR-6123	-0.937506875	-0.889658443	-	Negative	0.9582	0	0.1715	0.205318908	0.1589	0.205318908
Duodenum	AL	miR-145	-	-0.848890303	-	Negative	0.6569	0.083000234	0.2748	0.228624375	0.0802	0.311624609
Duodenum	AL	miR-133b	-0.884415424	-	-	Negative	0.4574	0.145850866	0.4307	-0.226770862	0.7358	-0.080919995
Duodenum	AL	miR-193b	-0.884257079	-	-	Negative	0.0745	-0.359620385	0.3358	0.194417794	0.3257	-0.165202591
Duodenum	AL	miR-2285r	-0.851134433	-	-	Negative	0.3315	0.415037499	0.5013	-0.415037499	0.9144	0

Duodenum	CS	miR-2284z	-	-	0.833202808	Positive	0.0587	-0.240532407	0.0697	0.263383346	0.8412	0.022850939
Duodenum	CS	miR-137	-	-	0.861116436	Positive	0.1407	0.299560282	0.5144	0.115477217	0.0213	0.415037499
Duodenum	CS	miR-671	-	0.82684913	-	Positive	0.4981	0.222392421	0.8759	0.026967048	0.4385	0.249359469
Duodenum	CS	miR-411c-5p	-	0.83480792	-	Positive	0.748	0.054861935	0.8478	-0.076235586	0.9729	-0.021373651
Duodenum	CS	miR-148a	0.837945408	0.851740198	-	Positive	0.9569	0.006586365	0.5153	0.09626695	0.4262	0.102853314
Duodenum	CS	miR-193a-3p	-	0.876737291	-	Positive	0.4698	-0.195324178	0.104	0.384143491	0.4116	0.188819313
Duodenum	CS	miR-6520	-	0.892277348	-	Positive	0.8011	0.054447784	0.1232	0.308122295	0.1651	0.362570079
Duodenum	CS	miR-29b	0.811805006	-	-	Positive	0.2503	0.152549465	0.4647	-0.094019909	0.5623	0.058529556
Duodenum	CS	miR-190a	0.816659325	-	-	Positive	0.9356	0.069041644	0.309	0.833221554	0.3145	0.902263198
Duoden	C	miR-1	0.81773	-	-	Positive	0.5	0.153805	0.7	0.083523	0.2	0.237328

um	S		172				77	336	84	356	67	692
							7		8		9	
							0.4		0.6		0.8	
Duoden	C	miR-15	0.83336				97	0.113765	96	-0.078838	32	0.034926
um	S	2	0605	-	-	Positive	4	11	5	518	6	592
							0.6		0.2		0.0	
Duoden	C	miR-14	0.83890				56	0.083000	74	0.228624	80	0.311624
um	S	5	6519	-	-	Positive	9	234	8	375	2	609
							0.2		0.1		0.6	
Duoden	C	miR-22	0.85520				05	-0.289506	06	0.459431	93	0.169925
um	S	84p	8764	-	-	Positive	6	617	6	619	6	001
									0.2		0.1	
Duoden	C	miR-15	0.85938				0.9	0.034194	81	-0.377282	29	-0.343087
um	S	3	025	-	-	Positive	17	92	3	577	7	657
									0.9		0.0	
Duoden	C	miR-37	0.86231				0.0	0.684498	14	-0.050626	92	0.633872
um	S	6e	2232	-	-	Positive	51	174	3	073	2	101
							0.2		0.1		0.5	
Duoden	C	miR-24	0.86674				50	0.248705	62	-0.334425	95	-0.085720
um	S	-3p	6827	-	-	Positive	8	763	8	931	6	169
							0.7		0.6		0.4	
Duoden	C	miR-20	0.90399				43	-0.046606	13	-0.075454	10	-0.122060
um	S	0b	6346	-	-	Positive	5	768	8	01	5	778
							0.2		0.2		0.8	
Duoden	C	miR-49	0.90578				69	-0.440821	73	0.476741	72	0.035919
um	S	0	0931	-	-	Positive	6	525	4	099	9	574
Duoden	C	miR-14	0.91054				0.8	-0.036519	0.0	0.310216	0.0	0.273696
um	S	3	2659	-	-	Positive	08	735	47	62	70	885

							2		9		1	
Duodenum	CS	miR-139	0.912576763	-	-	Positive	0.3399	-0.225857941	0.2461	0.293820531	0.7206	0.06796259
Duodenum	CS	miR-214	0.92001051	-	-	Positive	0.9726	0.006614844	0.6617	0.084647826	0.5623	0.09126267
Duodenum	CS	miR-3432	0.983693559	-	-	Positive	0.6345	-0.095280503	0.212	0.307089586	0.1669	0.211809082
Duodenum	CS	miR-23b-3p	-	-	-0.904937141	Negative	0.1025	0.209836795	0.7126	-0.052327543	0.1602	0.157509251
Duodenum	CS	miR-34c	-	-	-0.900479892	Negative	0.7737	0.114642566	0.5924	-0.38766106	0.6809	-0.273018494
Duodenum	CS	miR-188	-	-	-0.885887602	Negative	0.4848	-0.185555653	0.2048	0.303392143	0.6338	0.11783649
Duodenum	CS	miR-133b	-	-	-0.872043763	Negative	0.4574	0.145850866	0.4307	-0.226770862	0.7358	-0.080919995
Duodenum	CS	let-7a-3p	-	-	-0.867998998	Negative	0.5922	-0.091895866	0.3908	0.133462359	0.8188	0.041566493
Duodenum	CS	miR-141	-	-	-0.861744789	Negative	0.1121	0.293516871	0.0067	-0.656822526	0.0167	-0.363305654

Duodenum	CS	miR-34b	-	-	-0.841914253	Negative	0.5694	-0.321928095	0.9228	-0.056583528	0.6127	-0.378511623
Duodenum	CS	miR-184	-	-	-0.837885162	Negative	0.0057	0.749593202	0.1248	-0.730610099	0.957	0.018983103
Duodenum	CS	miR-132	-	-	-0.831343372	Negative	0.4887	-0.162446822	0.359	0.220114251	0.7808	0.057667429
Duodenum	CS	miR-200c	-	-	-0.829433597	Negative	0.2802	0.229560939	0.0781	-0.286829281	0.7845	-0.057268342
Duodenum	CS	miR-345-5p	-	-	-0.827913803	Negative	0.5517	0.114770187	0.473	-0.131644006	0.9416	-0.016873819
Duodenum	CS	miR-409a	-	-	-0.820838419	Negative	0.1721	-0.425305835	0.323	0.38466385	0.8901	-0.040641984
Duodenum	CS	miR-2483-5p	-	-0.891608363	-	Negative	0.1791	-0.700439718	0.7616	-0.206450877	0.1395	-0.906890596
Duodenum	CS	miR-210	-	-0.88707325	-	Negative	0.2357	-0.224462285	0.0929	0.276124405	0.8121	0.05166212
Duodenum	CS	miR-221	-	-0.838889906	-	Negative	0.3819	0.117335281	0.5391	-0.053860648	0.6708	0.063474633
Duoden	C	miR-61	-	-0.838217	-	Negative	0.9	0	0.1	0.205318	0.1	0.205318

um	S	23		653			58		71	908	58	908
							2		5		9	
							0.1		0.2		0.6	
Duoden	C	miR-65		-0.830738			81	-0.204254	79	0.139886	51	-0.064368
um	S	29	-	17	-	Negative	8	994	1	175	5	819
							0.3				0.5	
Duoden	C	miR-45		-0.827241			84	0.118836	0.7	-0.035979	27	0.082856
um	S	4	-	102	-	Negative	6	23	5	297	7	932
							0.1				0.4	
Duoden	C	miR-22	-0.9778				82	0.130724	0.1	-0.246108	72	-0.115383
um	S	85f	3975	-	-	Negative	9	612	89	49	3	878
							0.4		0.0		0.0	
Duoden	C	miR-19	-0.9765				77	0.091989	33	-0.256006	91	-0.164016
um	S	1	08932	-	-	Negative	9	376	6	149	6	773
							0.2		0.0			
Duoden	C	miR-22	-0.9757				50	0.157322	60	-0.426412	0.1	-0.269089
um	S	85k	61705	-	-	Negative	2	491	4	289	8	798
							0.0		0.1		0.4	
Duoden	C	miR-36	-0.9518				06	0.401862	16	-0.644905	80	-0.243042
um	S	3	16349	-	-	Negative	2	111	1	041	9	931
							0.6		0.3			
Duoden	C	miR-14	-0.9426				93	0.058293	13	-0.255636	0.3	-0.197343
um	S	2-3p	45581	-	-	Negative	2	41	3	814	59	404
									0.2			
Duoden	C	miR-21	-0.9420				0.6	0.106915	20	-0.621488	0.3	-0.514573
um	S	9-5p	56003	-	-	Negative	84	204	5	377	11	173
Duoden	C	miR-18	-0.9358				0.2	-0.248467	0.1	0.323297	0.6	0.074829
um	S	1b	75534	-	-	Negative	19	977	51	662	53	685

							6				7	
Duodenum	CS	miR-378b	-0.912614161	-	-	Negative	0.114	-0.270010442	0.2959	0.176296007	0.3833	-0.093714435
Duodenum	CS	miR-20b	-0.91112175	-	-	Negative	0.1185	0.500428991	0.2901	-0.642447995	0.7848	-0.142019005
Duodenum	CS	miR-155	-0.909395251	-	-	Negative	0.0906	-0.167530486	0.3819	-0.369030357	0.2323	-0.536560843
Duodenum	CS	miR-181a	-0.909045251	-	-	Negative	0.2555	-0.218499491	0.2279	0.266045679	0.8022	0.047546188
Duodenum	CS	miR-2284w	-0.901073642	-	-	Negative	0.3373	-0.119739244	0.2972	0.212848649	0.6622	0.093109404
Duodenum	CS	miR-18b	-0.889941692	-	-	Negative	0.2651	0.353636955	0.3497	-0.353636955	0.9597	0
Duodenum	CS	let-7i	-0.888178447	-	-	Negative	0.5331	-0.089049621	0.0659	0.232968936	0.3466	0.143919315
Duodenum	CS	miR-1246	-0.887184852	-	-	Negative	0.5914	-0.391830108	0.3761	0.711202498	0.4041	0.31937239
Duodenum	CS	miR-150	-0.865459054	-	-	Negative	0.3529	-0.307011582	0.8159	-0.114130399	0.3974	-0.421141981

Duodenum	CS	miR-25	-0.857962749	-	-	Negative	0.2919	-0.081355299	0.4571	0.065326725	0.8853	-0.016028574
Duodenum	CS	miR-6522	-0.855853722	-	-	Negative	0.5892	0.112474729	0.6462	-0.182864057	0.844	-0.070389328
Duodenum	CS	miR-378	-0.851781109	-	-	Negative	0.2782	-0.170412607	0.2245	0.188122561	0.8516	0.017709954
Duodenum	CS	miR-93	-0.838439447	-	-	Negative	0.712	-0.034309144	0.726	0.024441238	0.9236	-0.009867906
Duodenum	CS	miR-140	-0.819726748	-	-	Negative	0.2677	-0.127758066	0.1487	0.179955841	0.3626	0.052197775
Duodenum	CS	miR-2284v	-0.817639903	-	-	Negative	0.7388	-0.117356951	0.5793	0.192645078	0.8827	0.075288127
Duodenum	CS	miR-151-3p	-0.810398263	-	-	Negative	0.184	-0.133756778	0.3181	0.099895011	0.4454	-0.033861767
Duodenum	CS	miR-338	-0.810093078	-	-	Negative	0.8537	-0.030879313	0.3747	-0.189530022	0.2788	-0.220409335
Duodenum	RS	miR-2419-3p	-	-	0.826636781	Positive	0.8876	-0.125530882	0.7979	-0.115477217	0.6959	-0.2410081
Duoden	R	miR-18	-	-	0.834685	Positive	0.6	0.118488	0.5	0.111581	0.3	0.230069

um	S	2			271		28	251	97	015	13	266
							7		7		5	
									0.0		0.1	
Duoden	R	miR-12			0.879591		0.6	-0.099535	34	0.584962	62	0.485426
um	S	24	-	-	001	Positive	98	674	4	501	6	827
							0.7		0.6		0.4	
Duoden	R	miR-20			0.910465		43	-0.046606	13	-0.075454	10	-0.122060
um	S	0b	-	-	118	Positive	5	768	8	01	5	778
							0.0		0.2		0.7	
Duoden	R	miR-12	0.81007				98	-0.284257	48	0.232164	54	-0.052092
um	S	5b	3861	-	-	Positive	6	121	2	885	4	236
							0.1		0.7		0.1	
Duoden	R	miR-24	0.81346				79	-0.700439	61	-0.206450	39	-0.906890
um	S	83-5p	4153	-	-	Positive	1	718	6	877	5	596
							0.0		0.1		0.6	
Duoden	R	miR-99	0.81884				89	-0.462081	53	0.397493	70	-0.064588
um	S	b	5542	-	-	Positive	7	738	1	363	4	375
							0.6		0.6		0.9	
Duoden	R	miR-18	0.82104				80	-0.036530	36	0.042357	38	0.005827
um	S	6	8788	-	-	Positive	8	37	7	565	7	195
							0.4		0.0		0.0	
Duoden	R	miR-19	0.83542				77	0.091989	33	-0.256006	91	-0.164016
um	S	1	0917	-	-	Positive	9	376	6	149	6	773
							0.3		0.2		0.7	
Duoden	R	miR-14	0.85053				02	0.109850	69	-0.153219	01	-0.043368
um	S	8b	7531	-	-	Positive	9	579	4	311	6	732
Duoden	R	miR-10	0.86335				0.2	-0.395545	0.1	0.521094	0.4	0.125549
um	S	0	2228	-	-	Positive	30	483	48	892	85	409

							9		5		1	
Duodenum	RS	miR-151-3p	0.867050253	-	-	Positive	0.184	-0.133756778	0.3181	0.099895011	0.4454	-0.033861767
Duodenum	RS	miR-129	0.870421816	-	-	Positive	0.8622	-0.019365325	0.3327	0.22881869	0.3449	0.209453366
Duodenum	RS	miR-129-5p	0.870421816	-	-	Positive	0.8622	-0.019365325	0.3327	0.22881869	0.3449	0.209453366
Duodenum	RS	miR-190b	0.892123522	-	-	Positive	0.1987	-0.280107919	0.7006	0.087462841	0.364	-0.192645078
Duodenum	RS	miR-1271	0.895115563	-	-	Positive	0.9821	-0.0030598	0.7196	-0.09177154	0.6268	-0.09483134
Duodenum	RS	miR-1468	-	-	-0.93369488	Negative	0.2714	-0.210268543	0.0957	0.328041219	0.4531	0.117772675
Duodenum	RS	miR-2285r	-	-	-0.915681141	Negative	0.3315	0.415037499	0.5013	-0.415037499	0.9144	0
Duodenum	RS	miR-27a-3p	-	-	-0.914900373	Negative	0.2728	-0.136131483	0.4074	0.114846232	0.8378	-0.021285251
Duodenum	RS	miR-6522	-	-	-0.911281805	Negative	0.5892	0.112474729	0.6462	-0.182864057	0.844	-0.070389328

Duodenum	RS	miR-2285c	-	-	-0.902855532	Negative	0.4037	0.157541277	0.3399	0.208586622	0.1355	0.366127899
Duodenum	RS	miR-130a	-	-	-0.892429669	Negative	0.2885	-0.200551257	0.5844	0.105169985	0.4061	-0.095381271
Duodenum	RS	miR-181c	-	-	-0.890653741	Negative	0.1345	-0.21353641	0.0517	0.291914505	0.2113	0.078378095
Duodenum	RS	miR-139	-	-	-0.854357235	Negative	0.3399	-0.225857941	0.2461	0.293820531	0.7206	0.06796259
Duodenum	RS	let-7a-5p	-	-	-0.846070421	Negative	0.547	-0.100447265	0.0288	0.340437417	0.0932	0.239990152
Duodenum	RS	miR-151-5p	-	-	-0.840218867	Negative	0.119	-0.256340838	0.6033	-0.087575398	0.0148	-0.343916236
Duodenum	RS	miR-28	-	-	-0.822889945	Negative	0.0578	-0.276754832	0.3722	0.145147488	0.3314	-0.131607345
Duodenum	RS	miR-124a	-	-0.927757833	-	Negative	0.8985	-0.079226691	0.6884	0.278535499	0.6602	0.199308808
Duodenum	RS	miR-124b	-	-0.927757833	-	Negative	0.8985	-0.079226691	0.6884	0.278535499	0.6602	0.199308808
Duoden	R	miR-37	-	-0.922507	-	Negative	0.0	0.684498	0.9	-0.050626	0.0	0.633872

um	S	6e			641			51	174	14	073	92	101
										3		2	
								0.4		0.4		0.9	
Duoden	R	miR-22	-0.8401					96	0.160464	25	-0.234465	69	-0.074000
um	S	85m	05085	-	-		Negative	2	672	7	254	2	581
								0.0		0.0		0.0	
	A	miR-30				0.814940		98	0.176934	00	0.559521	00	0.736456
Jejunum	L	a-5p	-	-		042	Positive	6	713	2	583	3	296
								0.2		0.4		0.8	
	A	miR-10				0.819364		57	0.554321	05	-0.434429	00	0.119892
Jejunum	L	b	-	-		879	Positive	9	342	9	127	4	216
										0.2		0.4	
	A	miR-24				0.825758		0.3	0.192645	37	-0.362570	72	-0.169925
Jejunum	L	43	-	-		95	Positive	85	078	2	079	1	001
								0.4		0.2			
	A					0.826365		19	0.164119	93	0.127111	0.1	0.291231
Jejunum	L	miR-24	-	-		661	Positive	5	38	8	918	37	298
								0.0		0.0		0.0	
	A	miR-34				0.834116		36	0.285908	11	0.267077	02	0.552986
Jejunum	L	2	-	-		902	Positive	6	872	4	202	6	074
								0.3		0.1		0.0	
	A	miR-24				0.851095		38	-0.412472	00	-0.544173	18	-0.956646
Jejunum	L	19-5p	-	-		501	Positive	1	707	3	819	2	526
								0.1		0.3		0.7	
	A	miR-15				0.855593		69	-0.143888	16	0.103988	14	-0.039900
Jejunum	L	b	-	-		41	Positive	1	927	7	844	8	083
	A	miR-12				0.860222		0.1	0.257792	0.1	0.425829	0.0	0.683622
Jejunum	L	7	-	-		091	Positive	81	931	48	284	27	215

							6		3			
Jejunum	AL	miR-214	-	-	0.875307874	Positive	0.0364	0.359867252	0.9971	0.00110509	0.0695	0.360972342
Jejunum	AL	miR-2419-3p	-	-	0.897181156	Positive	0.4943	-0.736965594	0.1294	-0.847996907	0.0125	-1.584962501
Jejunum	AL	miR-132	-	-	0.90316154	Positive	0.0122	0.286761781	0.021	-0.475137264	0.2541	-0.188375483
Jejunum	AL	miR-432	-	-	0.919000253	Positive	0.4789	0.2255597	0.7145	-0.127379306	0.7843	0.098180394
Jejunum	AL	miR-193b	-	-	0.922881436	Positive	0.1571	0.265382161	0.0193	-0.337817997	0.6892	-0.072435836
Jejunum	AL	miR-195	-	-	0.939297438	Positive	0.5326	0.127157494	0.0035	0.495880801	0.0197	0.623038295
Jejunum	AL	miR-154b	-	-	0.992885856	Positive	0.7278	-0.180572246	0.4162	0.280107919	0.4737	0.099535674
Jejunum	AL	miR-138	0.862405491	-	-	Positive	0.4644	-0.126276268	0.4985	0.142019005	0.9223	0.015742737
Jejunum	AL	miR-2285i	0.875241435	-	-	Positive	0.809	-0.137503524	0.0483	1.652076697	0.1308	1.514573173

Jejunum	AL	miR-2487	0.903780286	-	-	Positive	0.7734	0.094636874	0.037	-0.878908183	0.0601	-0.784271309
Jejunum	AL	miR-2887	0.96443156	-	-	Positive	0.93	0.038135129	0.0765	-0.95419631	0.1019	-0.916061182
Jejunum	AL	miR-2904	0.936445689	-	-	Positive	0.9065	-0.044854236	0.1043	-0.760329587	0.1085	-0.805183823
Jejunum	AL	miR-486	-	0.895804433	-	Positive	0.0007	0.933389437	0.2179	-0.724708128	0.6356	0.208681309
Jejunum	AL	miR-1277	-	-	-0.985296668	Negative	0.501	0.160464672	0.5636	0.180572246	0.1245	0.341036918
Jejunum	AL	miR-424-3p	-	-	-0.956302528	Negative	0.0926	-0.600392541	0.5997	-0.173331603	0.0505	-0.773724144
Jejunum	AL	miR-31	-	-	-0.909086226	Negative	0.0242	-0.437645914	0.1054	0.826495602	0.4957	0.388849687
Jejunum	AL	miR-375	-	-	-0.908469651	Negative	0.5217	-0.115605976	0.0577	-0.397559606	0.0257	-0.513165582
Jejunum	AL	miR-2376	-	-	-0.902493009	Negative	0.5426	-0.111031312	0.7324	-0.103093493	0.5021	-0.214124805
Jejunum	A	miR-22	-	-	-0.883949	Negative	0.0	-0.472068	<.0	1.519374	0.0	1.047305

	L	85p			042		88	444	00	159	1	715
							4		1			
							0.2		0.8		0.2	
	A	miR-37			-0.878195		14	-0.295775	97	-0.034765	27	-0.330541
Jejunum	L	8b	-	-	796	Negative	6	807	6	418	8	225
							0.6		0.3		0.5	
	A	miR-67			-0.855945		62	0.080919	43	-0.206450	30	-0.125530
Jejunum	L	1	-	-	545	Negative	6	995	8	877	5	882
							0.5		0.6		0.9	
	A	miR-20			-0.853711		25	-0.092362	04	0.104255	58	0.011893
Jejunum	L	0a	-	-	268	Negative	9	393	5	551	6	158
							0.7		<.0		0.0	
	A	miR-42			-0.847273		63	-0.037535	00	1.153805	00	1.116269
Jejunum	L	5-3p	-	-	115	Negative	3	647	1	336	1	689
							0.8		0.9		0.9	
	A	miR-34			-0.846621		16	-0.128007	87	0.013674	10	-0.114332
Jejunum	L	c	-	-	514	Negative	8	612	8	937	9	675
							0.3		0.3		0.8	
	A	miR-37			-0.839514		17	-0.183248	25	0.137088	22	-0.046160
Jejunum	L	8	-	-	175	Negative	3	997	2	035	7	961
							0.3		0.2		0.1	
	A	miR-19			-0.826128		23	-0.148989	82	-0.291937	40	-0.440926
Jejunum	L	4	-	-	247	Negative	5	428	7	278	4	706
							0.3		0.9		0.5	
	A	miR-37			-0.823218		53	-0.133330	13	0.018021	84	-0.115309
Jejunum	L	8c	-	-	078	Negative	1	723	3	408	2	316
	A	miR-33			-0.820084		0.3	0.222392	0.3	0.263034	0.0	0.485426
Jejunum	L	0	-	-	668	Negative	55	421	52	406	28	827

							6		1			
Jejunum	AL	miR-197	-	-0.858573428	-	Negative	0.0235	0.359842867	0.0007	-0.731232481	0.0114	-0.371389614
Jejunum	AL	miR-2285k	-	-0.904671491	-	Negative	0.1698	0.14478725	0.0004	0.73835715	0.0001	0.8831444
Jejunum	AL	miR-30b-3p	-0.893612299	-0.856331404	-	Negative	0.2247	0.387023123	0.172	-0.387023123	0.8335	0
Jejunum	AL	miR-505	-	-0.876935459	-	Negative	0.7755	0.044807795	0.2335	0.184217718	0.0976	0.229025514
Jejunum	AL	miR-652	-0.856354004	-0.907492091	-	Negative	0.0076	0.50478313	0.0002	-0.79088702	0.0689	-0.28610389
Jejunum	CS	miR-2443	-	-	0.890545174	Positive	0.385	0.192645078	0.2372	-0.362570079	0.4721	-0.169925001
Jejunum	CS	miR-106a	-	0.921121033	-	Positive	0.7752	-0.070389328	0.1161	0.422691072	0.2773	0.352301744
Jejunum	CS	miR-1296	-	0.970826485	-	Positive	0.3819	0.216317907	0.0964	-0.338801913	0.4937	-0.122484007
Jejunum	CS	miR-150	-	0.826561263	-	Positive	0.115	0.36583759	0.1795	-0.23578364	0.5383	0.130053949

Jejunum	CS	miR-15b	0.824639812	-	-	Positive	0.1691	-0.143888927	0.3167	0.103988844	0.7148	-0.039900083
Jejunum	CS	miR-181c	-	0.905534789	-	Positive	0.5174	0.101743865	0.0294	0.231780059	0.0496	0.333523924
Jejunum	CS	miR-2285r	0.852561063	-	-	Positive	0.6565	-0.115477217	0.0093	0.893084796	0.0281	0.777607579
Jejunum	CS	miR-2904	-	0.846121273	-	Positive	0.9065	-0.044854236	0.1043	-0.760329587	0.1085	-0.805183823
Jejunum	CS	miR-30c	0.875795253	-	-	Positive	0.4516	-0.072750249	0.0327	0.246327027	0.0821	0.173576778
Jejunum	CS	miR-320a	-	0.83771444	-	Positive	0.1967	0.148977355	0.0081	-0.491448308	0.0301	-0.342470954
Jejunum	CS	miR-323	-	0.826837534	-	Positive	0.2369	0.530514717	0.0735	-0.637429921	0.7994	-0.106915204
Jejunum	CS	miR-339a	0.892652517	-	-	Positive	0.5471	0.074478216	0.7101	-0.040719033	0.7686	0.033759183
Jejunum	CS	miR-363	-	0.980851135	-	Positive	0.0873	0.181547369	0.7415	0.050626073	0.061	0.232173442
Jejunum	C	miR-42	0.88639	-	-	Positive	0.7	-0.037535	<.0	1.153805	0.0	1.116269

	S	5-3p	7199				63	647	00	336	00	689
							3		1		1	
							0.2		0.5		0.1	
	C	miR-44		0.837440			69	0.332575	46	0.169925	16	0.502500
Jejunum	S	9a	-	284	-	Positive	2	339	8	001	8	341
							0.8		0.3		0.1	
	C	miR-48		0.816158			41	-0.028453	02	-0.192164	49	-0.220618
Jejunum	S	4	-	627	-	Positive	1	895	2	74	7	635
							0.2		0.9		0.2	
	C	miR-49			-0.888372		49	0.241008	65		46	0.241008
Jejunum	S	1	-	-	456	Negative	9	1	6	0	3	1
									0.4		0.0	
	C	miR-24			-0.872078		0.0	0.710493	48	0.459431	03	1.169925
Jejunum	S	83-5p	-	-	471	Negative	76	383	8	619	6	001
							0.2				0.3	
	C	miR-28			-0.852981		74	1.137503	0.0	-1.863938	22	-0.726434
Jejunum	S	85	-	-	029	Negative	7	524	41	45	5	927
							0.6		0.0		0.0	
	C	miR-22		-0.843506			69	-0.094859	07	0.571906	86	0.477047
Jejunum	S	85b	-	826	-	Negative	3	186	3	348	3	162
							0.7		0.0			
	C	miR-22		-0.839737			99	-0.051530	03	0.529109	0.0	0.477578
Jejunum	S	85u	-	001	-	Negative	3	301	7	266	77	965
							0.2				0.8	
	C	miR-30	-0.8735				24	0.387023	0.1	-0.387023	33	
Jejunum	S	b-3p	69472	-	-	Negative	7	123	72	123	5	0
	C	miR-65	-0.8843				0.6	0.142019	0.1	-0.389946	0.3	-0.247927
Jejunum	S	23	03747	-	-	Negative	11	005	51	518	75	513

							7		5		1	
Jejunum	RS	miR-345-5p	-	-	0.819140477	Positive	0.083	0.325240829	0.6368	0.050626073	0.0666	0.375866902
Jejunum	RS	miR-363	-	-	0.834989905	Positive	0.0873	0.181547369	0.7415	0.050626073	0.061	0.232173442
Jejunum	RS	miR-369-5p	-	-	0.845141528	Positive	0.8644	-0.028569152	0.9463	-0.028014376	0.8572	-0.056583528
Jejunum	RS	miR-301a	-	-	0.856198834	Positive	0.4089	-0.113739147	0.0073	0.308177507	0.2248	0.19443836
Jejunum	RS	miR-2284z	-	-	0.861179872	Positive	0.0102	-0.486538445	0.0085	0.413186704	0.7227	-0.073351742
Jejunum	RS	miR-2336	-	-	0.887445029	Positive	0.219	-0.216999163	0.002	0.491925464	0.2062	0.274926302
Jejunum	RS	miR-182	-	-	0.891087273	Positive	0.4159	-0.2125129	0.9889	-0.002829631	0.3302	-0.215342531
Jejunum	RS	miR-378c	-	-	0.931257192	Positive	0.3531	-0.133330723	0.9133	0.018021408	0.5842	-0.115309316
Jejunum	RS	let-7e	0.832691208	-	-	Positive	0.4102	0.190908001	0.0388	-0.467215552	0.1561	-0.276307551

Jejunum	RS	miR-100	0.828601842	-	-	Positive	0.1281	0.415971585	<.0001	1.322715163	0.0006	1.738686747
Jejunum	RS	miR-125b	0.896865876	0.858389021	-	Positive	0.1235	0.247779684	0.0041	0.423802	0.0039	0.671581684
Jejunum	RS	miR-1307	0.827824761	-	-	Positive	0.4648	0.177084793	0.0007	-0.918329268	0.0036	-0.741244475
Jejunum	RS	miR-17-3p	-	0.932595895	-	Positive	0.4346	-0.124071108	0.0199	-0.374303708	0.011	-0.498374816
Jejunum	RS	miR-18a	-	0.919277012	-	Positive	0.9932	0.00234294	0.0397	0.333705263	0.1701	0.336049203
Jejunum	RS	miR-199c	0.925788242	0.927416854	-	Positive	0.5421	0.099258871	0.9842	0.002077315	0.5677	0.101336186
Jejunum	RS	miR-19a	-	0.889886965	-	Positive	0.0951	-0.256405667	0.0122	0.333044127	0.6527	0.07663846
Jejunum	RS	miR-200a	-	0.921311491	-	Positive	0.5259	-0.092362393	0.6045	0.104255551	0.9586	0.011893158
Jejunum	RS	miR-20a	-	0.949795662	-	Positive	0.9832	0.003240452	0.0131	0.375872419	0.0469	0.379112871
Jejunum	R	miR-22	0.94549	0.956537	-	Positive	0.3	-0.104512	0.0	-0.173528	0.0	-0.278040

	S	1	4695	039			71	047	78	238	25	284
							3		8		3	
							0.2		0.2		0.8	
	R	miR-22	0.81430	0.813495			35	-0.280107	89	0.280107	95	
Jejunum	S	85c	81	695	-	Positive	5	919	2	919	8	0
							0.0				0.8	
	R	miR-32	0.85069	0.914004			51	0.352190	0.1	-0.320597	74	0.031593
Jejunum	S	4	7286	022	-	Positive	2	934	46	809	7	125
							0.8		0.4		0.3	
	R	miR-33	0.83069	0.844979			16	-0.048094	78	-0.198545	82	-0.246639
Jejunum	S	b	1593	119	-	Positive	1	288	7	679	7	968
							0.0				0.5	
	R	miR-34	0.96464				60	0.409112	0.0	-0.304479	54	0.104633
Jejunum	S	31	7613	-	-	Positive	8	665	25	124	3	541
							0.7		<.0		0.0	
	R	miR-42	0.84825				63	-0.037535	00	1.153805	00	1.116269
Jejunum	S	5-3p	9559	-	-	Positive	3	647	1	336	1	689
											0.0	
	R	miR-42		0.811187			0.3	-0.192732	0.1	-0.383028	30	-0.575761
Jejunum	S	9	-	93	-	Positive	41	153	33	936	7	088
							0.0		0.7		0.1	
	R	miR-45		0.833421			90	0.308429	04	0.055432	02	0.363862
Jejunum	S	5-3p	-	938	-	Positive	8	847	7	391	9	238
							0.2		0.9		0.2	
	R	miR-49	0.81043				49	0.241008	65		46	0.241008
Jejunum	S	1	2165	-	-	Positive	9	1	6	0	3	1
	R	miR-50		0.813948			0.4	-0.082462	0.0	-0.473931	0.0	-0.556393
Jejunum	S	2a	-	7	-	Positive	95	16	35	188	32	349

							2		7		2	
Jejunum	RS	miR-6119-3p	-	0.88475301	-	Positive	0.2354	-0.179108324	0.0053	0.534588978	0.0293	0.355480655
Jejunum	RS	miR-6523	0.910744842	-	-	Positive	0.6117	0.142019005	0.1515	-0.389946518	0.3751	-0.247927513
Jejunum	RS	miR-671	-	0.821990843	-	Positive	0.6626	0.080919995	0.3438	-0.206450877	0.5305	-0.125530882
Jejunum	RS	miR-760-3p	0.870159807	-	-	Positive	0.0397	0.493040011	0.0068	-0.704544116	0.418	-0.211504105
Jejunum	RS	miR-769	0.830557188	-	-	Positive	0.9463	0.009690057	0.0477	-0.485640496	0.0551	-0.475950439
Jejunum	RS	miR-99a-3p	-	0.862002969	-	Positive	0.9568	0.009277814	0.1154	0.393481356	0.0986	0.40275917
Jejunum	RS	miR-3432	-	-	-0.947368308	Negative	0.4755	0.167186919	0.0118	0.355553066	0.0649	0.522739986
Jejunum	RS	miR-486	-	-	-0.942842301	Negative	0.0007	0.933389437	0.2179	-0.724708128	0.6356	0.208681309
Jejunum	RS	miR-193b	-	-	-0.920400061	Negative	0.1571	0.265382161	0.0193	-0.337817997	0.6892	-0.072435836

Jejunum	RS	miR-185	-	-	-0.860130744	Negative	0.2058	0.252692462	0.0148	0.485426827	0.0076	0.738119289
Jejunum	RS	miR-1271	-	-	-0.821671013	Negative	0.1901	0.431157165	<.0001	2.258502715	0.0008	2.689659879
Jejunum	RS	miR-874	-	-	-0.810729811	Negative	0.0156	0.379848071	0.2892	0.185214872	0.0026	0.565062943
Jejunum	RS	miR-99a-5p	-	-	-0.810112821	Negative	0.1852	0.27908156	0.0001	1.055516051	0.0015	1.334597611
Jejunum	RS	miR-1246	-	-0.816652076	-	Negative	0.19	-1.08305574	0.3174	0.742018822	0.3014	-0.341036918
Jejunum	RS	miR-141	-0.933232058	-	-	Negative	0.3553	0.176052404	0.3731	0.16837722	0.1874	0.344429623
Jejunum	RS	miR-151-3p	-0.837759844	-	-	Negative	0.1749	0.080124748	0.0214	0.179269942	0.007	0.25939469
Jejunum	RS	miR-2285t	-0.874091758	-	-	Negative	0.8922	-0.033748791	0.0004	0.679740725	0.0627	0.645991934
Jejunum	RS	miR-2411-5p	-0.938856266	-	-	Negative	0.1003	-0.447458977	0.1863	0.321928095	0.6597	-0.125530882
Jejunum	R	miR-37	-0.8207	-	-	Negative	0.9	0.009202	0.0	0.695831	0.0	0.705033

	S	4a	43167				48	066	01	348	04	414
							1		3		1	
							0.2		0.8		0.2	
	R	miR-37	-0.8111	-0.866850			14	-0.295775	97	-0.034765	27	-0.330541
Jejunum	S	8b	77882	862	-	Negative	6	807	6	418	8	225
							0.7		<.0		0.0	
	R	miR-37	-0.8278				31	0.066233	00	1.727315	00	1.793549
Jejunum	S	9	67559	-	-	Negative	3	698	1	424	1	123
							0.6		0.1		0.1	
	R	miR-50	-0.8360				51	0.093109	15	0.736965	17	0.830074
Jejunum	S	3-3p	66203	-	-	Negative	9	404	4	594	6	999
							0.8				0.1	
	R	miR-65		-0.845051			24	0.034889	0.0	0.293435	09	0.328325
Jejunum	S	24	-	043	-	Negative	8	982	76	884	7	866
							0.1		0.0		0.4	
	A	miR-18			0.829409		10	-0.350876	64	0.489618	08	0.138741
Liver	L	1b	-	-	482	Positive	4	395	1	151	7	756
							0.1		0.5		0.2	
	A	miR-45			0.863725		63	-0.624104	27	0.282316	89	-0.341788
Liver	L	0a	-	-	68	Positive	1	756	8	239	2	517
											0.2	
	A	miR-24			0.864697		0.3	-0.573163	0.7	0.135136	84	-0.438027
Liver	L	19-5p	-	-	264	Positive	58	929	73	078	4	852
							0.4		0.1		0.2	
	A	miR-18			0.914106		31	-0.190885	11	0.457643	50	0.266758
Liver	L	1a	-	-	663	Positive	8	097	3	137	5	04
	A	miR-18	0.81498				0.6	-0.180572	0.2	0.502500	0.4	0.321928
Liver	L	14c	7831	-	-	Positive	40	246	32	341	88	095

							1		5		5	
Liver	AL	miR-199a-3p	0.941488439	0.831497583	-	Positive	0.3558	-0.179099018	0.4665	0.16536528	0.9278	-0.013733738
Liver	AL	miR-2310	0.814987831	-	-	Positive	0.6401	-0.180572246	0.2325	0.502500341	0.4885	0.321928095
Liver	AL	miR-2399-5p	0.983247736	0.924976527	-	Positive	0.5715	-0.160464672	0.5066	0.247927513	0.8028	0.087462841
Liver	AL	miR-2403	0.843962132	-	-	Positive	0.8109	0.125530882	0.7247	-0.2410081	0.8318	-0.115477217
Liver	AL	miR-449a	0.951967575	-	-	Positive	0.5151	0.592342031	0.941	-0.065095028	0.5458	0.527247003
Liver	AL	let-7g	-	-	-0.90919072	Negative	0.7042	-0.033981359	0.0802	0.170981465	0.0894	0.137000106
Liver	AL	let-7a-5p	-	-	-0.889641568	Negative	0.3414	-0.09057732	0.1155	0.147208395	0.4602	0.056631075
Liver	AL	miR-760-3p	-	-	-0.884390438	Negative	0.5347	0.347923303	0.8777	0	0.542	0.347923303
Liver	AL	miR-26b	-	-	-0.874418456	Negative	0.717	0.033611835	0.9912	0.000766283	0.7295	0.034378117

Liver	AL	miR-140	-	-	-0.851137476	Negative	0.3279	0.101664092	0.0284	-0.145459397	0.6585	-0.043795305
Liver	AL	miR-365-5p	-	-	-0.83647537	Negative	0.6232	0.096215315	0.5908	0.103093493	0.3489	0.199308808
Liver	AL	miR-17-3p	-	-	-0.816379337	Negative	0.2722	-0.138328157	0.1596	0.184424571	0.7882	0.046096415
Liver	AL	miR-1	-0.819833429	-0.848622469	-	Negative	0.3449	-0.514573173	0.0456	-0.722466024	0.0018	-1.237039197
Liver	AL	miR-133a	-	-0.900926683	-	Negative	0.9602	-0.013631866	0.6256	-0.145294137	0.577	-0.158926002
Liver	AL	miR-193a-5p	-0.811385098	-	-	Negative	0.6564	0.076235586	0.6512	0.092364018	0.3142	0.168599604
Liver	AL	miR-2285e	-0.831167515	-0.862600041	-	Negative	0.6311	-0.150416843	0.6198	0.158365596	0.9599	0.007948753
Liver	AL	miR-376e	-	-0.90993892	-	Negative	0.754	-0.140177658	0.9514	-0.05246742	0.6614	-0.192645078
Liver	AL	miR-421	-0.891190145	-0.89190145	-	Negative	0.6976	-0.050169596	0.2578	0.193166943	0.1955	0.142997346
Liver	A	miR-45	-0.8565	-0.895973	-	Negative	0.5	-0.347019	0.7	0.186364	0.5	-0.160654

	L	5-3p	73325	995			73	076	43	112	45	964
									3		1	
									0.8		0.8	
	A	miR-50		-0.867967				-0.078002	26	0.078002	26	
Liver	L	2a	-	164	-	Negative	0.6	512	8	512	3	0
							0.4		0.2			
	A	miR-67	-0.8381	-0.836160			48	0.131244	86	-0.251538	0.6	-0.120294
Liver	L	1	02807	122	-	Negative	1	533	8	767	26	234
							0.6		0.6		0.9	
	C	miR-22			0.846756		31	-0.150416	19	0.158365	59	0.007948
Liver	S	85e	-	-	138	Positive	1	843	8	596	9	753
							0.9		0.1		0.2	
	C	miR-50			0.857821		87		72	-0.253756	19	-0.253756
Liver	S	0	-	-	023	Positive	5	0	1	592	3	592
							0.6		0.7		0.8	
	C	miR-14			0.867948		22	-0.055360	42	0.029950	24	-0.025409
Liver	S	8a	-	-	99	Positive	8	386	2	813	1	573
									0.6		0.3	
	C	miR-20			0.939158		0.6	-0.094437	00	-0.081808	21	-0.176245
Liver	S	a	-	-	55	Positive	12	464	3	344	2	808
							0.6		0.8		0.5	
	C	miR-19			0.976418		89	0.070389	34	0.036525	40	0.106915
Liver	S	7	-	-	096	Positive	2	328	1	876	4	204
							0.6				0.1	
	C	miR-15		0.828357			47	-0.075546	0.4	-0.109391	14	-0.184938
Liver	S	2	-	734	-	Positive	7	932	83	934	2	867
	C	miR-20		0.821738			0.3	8.730753	<.0	-4.087462	0.3	4.643290
Liver	S	5	-	504	-	Positive	40	157	00	841	57	316

							4		1		9	
Liver	CS	miR-2284aa	-	0.94006448	-	Positive	0.2084	0.243454037	0.3306	0.144948336	0.1017	0.388402373
Liver	CS	miR-2284y	-	0.83044696	-	Positive	0.6639	-0.089267338	0.4918	0.184424571	0.6972	0.095157233
Liver	CS	miR-24	-	0.848127981	-	Positive	0.7062	0.071258683	0.7124	-0.053771256	0.9621	0.017487427
Liver	CS	miR-2419-5p	-	0.856740298	-	Positive	0.358	-0.573163929	0.773	0.135136078	0.2844	-0.438027852
Liver	CS	miR-30b-3p	-	0.838454144	-	Positive	0.8408	-0.106915204	0.9809	0	0.8597	-0.106915204
Liver	CS	miR-365-5p	-	0.87600964	-	Positive	0.6232	0.096215315	0.5908	0.103093493	0.3489	0.199308808
Liver	CS	miR-374b	0.862480894	-	-	Positive	0.1386	0.242430175	0.5157	-0.08171465	0.3157	0.160715525
Liver	CS	miR-375	0.87654676	-	-	Positive	0.5918	0.418879566	0.0469	0.548869498	0.3007	0.967749064
Liver	CS	miR-532	-	0.87792312	-	Positive	0.6403	0.07520882	0.586	-0.043779573	0.8315	0.031429247

Liver	CS	miR-9-5p	0.869794543	-	-	Positive	0.7338	-0.166897309	0.3347	0.451874267	0.4288	0.284976959	
Liver	CS	let-7c	-	-	-0.964571983	Negative	0.7395	0.05267403	0.9113	0.020126028	0.606	0.072800058	
Liver	CS	miR-27a-5p	-	-	-0.954168473	Negative	0.2747	0.612976877	0.9264	-0.08246216	0.311	0.530514717	
Liver	CS	let-7b	-	-	-0.953633969	Negative	0.2455	0.111351424	0.7148	-0.025479586	0.4322	0.085871838	
Liver	CS	miR-34c	-	-	-0.942897687	Negative	0.2881	-0.618129365	0.7471	-0.203533394	0.2225	-0.821662759	
Liver	CS	miR-199a-3p	-	-	-0.933047947	Negative	0.3558	-0.179099018	0.4665	0.16536528	0.9278	-0.013733738	
Liver	CS	miR-199c	-	-	-0.919264583	Negative	0.6417	0.100730632	0.4345	-0.263034406	0.6102	-0.162303774	
Liver	CS	miR-99a-3p	-	-	-0.91839811	Negative	0.8785	0.020536931	0.8823	-0.02816353	0.9619	-0.007626599	
Liver	CS	miR-6522	-	-	-0.900566816	Negative	0.1016	-0.559427409	0.578	0.222392421	0.2642	-0.337034987	
Liver	C	miR-18	-	-	-0.897647	Negative	0.9	0.030161	0.5	0.149747	0.4	0.179909	Liver(Down)

	S	1d			628		265	971	679	12	665	09
Liver	CS	miR-214	-	-	-0.888738917	Negative	0.785	0.035352978	0.2011	-0.315744223	0.2421	-0.280391245
Liver	CS	miR-155	-	-	-0.886560823	Negative	0.8129	-0.071083098	0.0314	0.595721847	0.1125	0.524638749
Liver	CS	miR-27a-3p	-	-	-0.881092605	Negative	0.6085	0.06790613	0.5421	0.043498889	0.4281	0.111405018
Liver	CS	miR-195	-	-	-0.869198668	Negative	0.7082	0.070962424	0.4008	-0.26034269	0.5532	-0.189380267
Liver	CS	miR-199b	-	-	-0.867222448	Negative	0.7145	0.159070418	0.8031	-0.126007885	0.9451	0.033062533
Liver	CS	miR-130a	-	-	-0.859998459	Negative	0.3926	-0.163249576	0.7226	0.070931623	0.5521	-0.092317953
Liver	CS	miR-504	-	-	-0.854587073	Negative	0.4794	0.519374159	0.3703	0.514573173	0.2452	1.033947332
Liver	CS	miR-218	-	-	-0.847169161	Negative	0.6111	0.11983977	0.2644	-0.565904165	0.3515	-0.446064395
Liver	CS	miR-199a-5p	-	-	-0.840498265	Negative	0.878	0.027005387	0.411	-0.215771823	0.464	-0.188766436

							6				8		
Liver	CS	miR-221	-	-	-0.835908207	Negative	0.2736	0.511500339	0.5327	-0.165750424	0.4523	0.345749915	Liver(Down)
Liver	CS	miR-34a	-	-	-0.823617385	Negative	0.1016	0.762641728	0.253	-1.035297052	0.6943	-0.272655324	
Liver	CS	miR-10a	-	-	-0.812608533	Negative	0.4929	0.255348808	0.2842	-0.505340191	0.6241	-0.249991383	
Liver	CS	miR-146b	-	-	-0.810918148	Negative	0.0163	1.857140726	0.2266	-1.317047709	0.4582	0.540093018	
Liver	CS	miR-92b	-	-	-0.810176298	Negative	0.229	0.224919033	0.4683	0.099219605	0.0913	0.324138638	
Liver	CS	miR-1249	-0.926991547	-	-	Negative	0.843	-0.062735755	0.4169	0.250808104	0.5823	0.188072348	
Liver	CS	miR-129-3p	-	-0.824927246	-	Negative	0.7507	-0.125530882	0.7096	0.125530882	0.9383	0	
Liver	CS	miR-1343-3p	-0.891047088	-	-	Negative	0.5286	0.169058258	0.1255	0.422857004	0.0146	0.591915261	
Liver	CS	miR-145	-	-0.857798364	-	Negative	0.5635	-0.175373813	0.4733	-0.172449787	0.1063	-0.3478236	

Liver	CS	miR-204	-	-0.857972711	-	Negative	0.5876	-0.177363299	0.6483	-0.117871058	0.2458	-0.295234356	
Liver	CS	miR-2285k	-0.823389091	-	-	Negative	0.2411	0.263668697	0.7998	-0.052331464	0.3069	0.211337233	
Liver	CS	miR-455-3p	-0.914749849	-	-	Negative	0.573	-0.347019076	0.7433	0.186364112	0.5451	-0.160654964	
Liver	CS	miR-483	-0.851789297	-	-	Negative	0.1465	0.38332864	0.958	-0.012490944	0.1256	0.370837695	
Liver	CS	miR-484	-0.863993079	-	-	Negative	0.4359	0.11937956	0.9735	0.005088877	0.2967	0.124468436	
Liver	CS	miR-502b	-0.886411461	-0.83688906	-	Negative	0.4529	0.190331212	0.5473	-0.123988717	0.7557	0.066342495	Liver(Up)
Liver	CS	miR-592	-	-0.842399345	-	Negative	0.1172	-0.652076697	0.0799	0.781359714	0.6683	0.129283017	
Liver	CS	miR-652	-0.811195195	-	-	Negative	0.2537	0.271302022	0.5565	0.099281879	0.1024	0.370583901	
Liver	CS	miR-6520	-0.811303571	-	-	Negative	0.8571	-0.041820176	0.0014	0.669851398	0.0124	0.628031223	
Liver	C	miR-87	-0.8593	-0.891354	-	Negative	0.8	-0.028929	0.8	-0.027557	0.7	-0.056487	

	S	4	81532	819			82	257	821	934	674	191	
Liver	RS	miR-25	-	-	0.815105136	Positive	0.3891	0.0805334	0.4804	0.052105824	0.0776	0.132639224	
Liver	RS	let-7g	-	-	0.823922696	Positive	0.7042	-0.033981359	0.0802	0.170981465	0.0894	0.137000106	Liver(Down)
Liver	RS	miR-2284z	-	-	0.824523768	Positive	0.8195	0.045514659	0.1176	0.308122295	0.1561	0.353636955	
Liver	RS	miR-28	-	-	0.8257032	Positive	0.0815	-0.286650568	0.0724	0.322019123	0.7664	0.035368555	
Liver	RS	miR-92a	-	-	0.826682752	Positive	0.1247	-0.230020436	0.0821	0.284401441	0.7634	0.054381005	
Liver	RS	miR-2887	-	-	0.828013487	Positive	0.3009	1.060153135	0.7607	-0.14093579	0.3601	0.919217345	
Liver	RS	miR-95	-	-	0.831421058	Positive	0.1256	-0.362570079	0.2228	0.231325546	0.6748	-0.131244533	
Liver	RS	miR-128	-	-	0.837754875	Positive	0.1335	-0.369456465	0.243	0.278691001	0.6522	-0.090765464	
Liver	RS	miR-2284aa	-	-	0.837971589	Positive	0.208	0.243454037	0.330	0.144948336	0.101	0.388402373	

							4		6		7		
Liver	RS	miR-2419-3p	-	-	0.841667453	Positive	0.473	-0.788495895	0.7641	-0.144389909	0.1399	-0.932885804	
Liver	RS	miR-33b	-	-	0.863618322	Positive	0.8786	-0.019003942	0.7088	-0.075101455	0.6597	-0.094105397	
Liver	RS	miR-375	-	-	0.869761557	Positive	0.5918	0.418879566	0.0469	0.548869498	0.3007	0.967749064	
Liver	RS	miR-6517	-	-	0.872084134	Positive	0.8114	-0.064130337	0.7638	0.064130337	0.9942	0	
Liver	RS	miR-497	-	-	0.901360068	Positive	0.025	-0.402978123	0.08	0.336227885	0.6808	-0.066750237	Liver(Specific)
Liver	RS	miR-2487	-	-	0.915160958	Positive	0.129	1.276383959	0.0698	-0.628369288	0.3453	0.648014671	
Liver	RS	miR-2478	-	-	0.948077657	Positive	0.3044	0.450265988	0.5245	0.215833276	0.1633	0.666099263	
Liver	RS	miR-1291	0.855485102	-	-	Positive	0.1606	1.03562391	0.4894	-0.263034406	0.2417	0.772589504	
Liver	RS	miR-129-3p	0.904947898	-	-	Positive	0.7507	-0.125530882	0.7096	0.125530882	0.9383	0	

Liver	RS	miR-1306	0.810196676	-	-	Positive	0.7698	-0.100928909	0.2226	0.30256277	0.5006	0.201633861	
Liver	RS	miR-219-5p	0.867460477	0.853782961	-	Positive	0.2864	-0.447458977	0.1209	0.584962501	0.7436	0.137503524	
Liver	RS	miR-2468	-	0.851184736	-	Positive	0.7524	-0.064130337	0.7591	-0.120294234	0.5378	-0.184424571	
Liver	RS	miR-30b-3p	0.938297434	-	-	Positive	0.8408	-0.106915204	0.9809	0	0.8597	-0.106915204	
Liver	RS	miR-3613	0.860562897	0.810548658	-	Positive	0.1509	-0.347923303	0.4322	0.129283017	0.2186	-0.218640286	
Liver	RS	miR-151-5p	-	-	-0.963597472	Negative	0.9475	0.008322578	0.4645	-0.104138841	0.5242	-0.095816264	
Liver	RS	miR-23a	-	-	-0.948770277	Negative	0.4989	0.141940945	0.3554	-0.148716445	0.977	-0.006775501	
Liver	RS	miR-7	-	-	-0.901553119	Negative	0.0746	0.592575685	0.0177	-0.652076697	0.8431	-0.059501012	
Liver	RS	miR-361	-	-	-0.899821158	Negative	0.4836	0.091565353	0.1465	-0.188848161	0.4387	-0.097282808	
Liver	R	miR-10	-	-	-0.897715	Negative	0.5	0.099553	0.2	-0.398690	0.3	-0.299137	Liver(Down)

	S	0			413		83	924	53	968	78	044
							2				4	
							0.2		0.2		0.5	
	R	miR-99			-0.887855		28	0.258908	36	-0.477121	75	-0.218213
Liver	S	a-5p	-	-	645	Negative	5	149	2	649	1	5
							0.0		0.0		0.9	
	R	miR-37			-0.870473		70	0.415695	62	-0.416353	97	-0.000657
Liver	S	4a	-	-	843	Negative	3	513	5	227	2	714
							0.1		0.0		0.6	
	R	miR-24			-0.848797		06	0.525356	47	-0.700513	18	-0.175156
Liver	S	-3p	-	-	89	Negative	9	985	2	618	6	634
									0.6		0.8	
	R	miR-19			-0.847180		0.6	0.071157	99	-0.056037	74	0.015120
Liver	S	2	-	-	199	Negative	01	845	4	034	7	811
							0.2		0.7		0.3	
	R	miR-22			-0.829806		41	0.263668	99	-0.052331	06	0.211337
Liver	S	85k	-	-	774	Negative	1	697	8	464	9	233
							0.1		0.1		0.6	
	R	miR-21			-0.824079		47	0.472170	71	-0.351531	88	0.120638
Liver	S	-3p	-	-	486	Negative	5	73	3	808	3	923
							0.6		0.2		0.4	
	R	miR-18			-0.820089		40	-0.180572	32	0.502500	88	0.321928
Liver	S	14c	-	-	163	Negative	1	246	5	341	5	095
							0.6		0.2		0.4	
	R	miR-23			-0.820089		40	-0.180572	32	0.502500	88	0.321928
Liver	S	10	-	-	163	Negative	1	246	5	341	5	095
	R	miR-18			-0.818464		0.9	-0.024045	0.3	-0.290590	0.5	-0.314635
Liver	S	3	-	-	279	Negative	77	474	17	128	78	602

							8		9		1	
							0.2		0.2		0.7	
	R	miR-19			-0.815577		75	0.215459	32	-0.268199	48	-0.052739
Liver	S	1	-	-	372	Negative	1	707	5	141	7	434
							0.3		0.5		0.5	
	R	miR-11	-0.8586				19	-0.522421	26	0.303780	42	-0.218640
Liver	S	85	19105	-	-	Negative	6	035	1	748	5	286
									0.4		0.1	
	R	miR-15	-0.8251	-0.829562			0.1	-0.784271	25	0.339486	47	-0.444784
Liver	S	4b	3831	645	-	Negative	43	309	2	466	1	843
							0.2		0.6		0.5	
	R	miR-18		-0.961828			87	-0.242570	26	0.102957	42	-0.139613
Liver	S	1c	-	734	-	Negative	5	303	8	19	1	113
							0.5		0.7		0.6	
	R	miR-18		-0.893541			13	-0.286304	52	0.100824	56	-0.185479
Liver	S	2	-	442	-	Negative	4	185	4	37	4	816
							0.3		0.8		0.1	
	R	miR-23	-0.8603				38	0.415037	06	0.125530	73	0.540568
Liver	S	76	81973	-	-	Negative	6	499	6	882	9	381
							0.8		0.7		0.8	
	R	miR-24	-0.8948	-0.984902			10	0.125530	24	-0.241008	31	-0.115477
Liver	S	03	83834	674	-	Negative	9	882	7	1	8	217
							0.8		0.3		0.4	
	R	miR-36		-0.831487			02	-0.099535	90	0.282399	85	0.182864
Liver	S	3	-	008	-	Negative	4	674	2	731	8	057
							0.8		0.7		0.9	
	R	miR-37	-0.8859				17	0.088536	32	-0.122484	22	-0.033947
Liver	S	6b	69555	-	-	Negative	4	675	8	007	1	332

Liver	RS	miR-493	-0.851837749	-0.851296791	-	Negative	0.6356	-0.232290882	0.7278	0.147898695	0.8259	-0.084392187	Liver(Down)
Liver	RS	miR-504	-0.84122364	-	-	Negative	0.4794	0.519374159	0.3703	0.514573173	0.2452	1.033947332	
Liver	RS	miR-6520	-0.860023909	-	-	Negative	0.8571	-0.041820176	0.014	0.669851398	0.0124	0.628031223	
Liver	RS	miR-655	-0.843048746	-	-	Negative	0.5303	-0.22881869	0.7282	-0.093109404	0.3581	-0.321928095	
Liver	RS	miR-660	-0.861859514	-	-	Negative	0.3788	-0.072712309	0.5786	0.03713079	0.6677	-0.035581519	
Liver	RS	miR-665	-	-0.820359326	-	Negative	0.4729	-0.288125387	0.4532	0.318822184	0.9614	0.030696797	
Mammary gland	RS	miR-451	-	-	-0.983938683	Negative	0.0193	1.106822752	0.0191	-0.934692561	0.5615	0.172130191	
Mammary gland	RS	miR-24	-	-	-0.973721556	Negative	0.0741	0.722466024	0.001	-1.137503524	0.0902	-0.415037499	
Mammary gland	CS	miR-486	-	-	-0.930389987	Negative	0.5726	-0.250943898	0.1221	0.716289755	0.2874	0.465345857	
Mamma	R	miR-14	-	-	-0.926406	Negative	0.0	0.957890	0.0	-0.934611	0.9	0.023279	Mammary

ry gland	S	1			063		34	725	11	225	22	5	Gland(Up)
							5		3		1		
							0.7		0.4		0.4		
Mamma	C	miR-92			-0.904329		35	-0.098795	99	-0.168048	27	-0.266843	
ry gland	S	a	-	-	474	Negative	7	059	7	441	4	5	
							0.2		0.0		0.0		
Mamma	C	miR-22			-0.901730		73	0.550363	25	-1.568919	68	-1.018555	
ry gland	S	4	-	-	372	Negative	5	839	5	658	6	819	
							0.3		0.9				
Mamma	C	miR-50			-0.875389		46	-0.412194	10	0.034557	0.2	-0.377637	
ry gland	S	4	-	-	897	Negative	8	75	8	222	51	527	
									0.2		0.0		
Mamma	C	miR-42			-0.872391		0.5	-0.377772	67	-0.548040	19	-0.925813	
ry gland	S	3-5p	-	-	262	Negative	34	368	2	736	7	105	
							0.0		0.0		0.1		
Mamma	R	miR-33			-0.857529		32	0.890051	02	-1.192183	80	-0.302131	
ry gland	S	8	-	-	839	Negative	8	533	8	391	8	858	
							0.0		0.0		0.3		
Mamma	R	miR-36			-0.843793		19	0.869530	03	-1.058459	69	-0.188929	
ry gland	S	2-5p	-	-	222	Negative	3	068	1	731	3	662	
									0.0		0.0		
Mamma	R	let-7a-			-0.840848		0.0	0.592364	02	-1.088510	26	-0.496145	
ry gland	S	3p	-	-	652	Negative	86	749	9	17	5	421	
							0.3				0.1		
Mamma	R	miR-13			-0.838025		96	0.689659	0.0	-1.446683	87	-0.757023	
ry gland	S	5a	-	-	018	Negative	4	879	13	126	4	247	
Mamma	C	miR-22			-0.833771		0.0	-1.688055	0.0	0.951090	0.1	-0.736965	
ry gland	S	84b	-	-	813	Negative	18	994	84	4	78	594	

							7		3		6	
Mammary gland	CS	miR-502b	-	-	-0.823799491	Negative	0.0336	-1.362570079	0.0313	1.476902755	0.8161	0.114332675
Mammary gland	RS	miR-144	-	-	-0.823582286	Negative	0.0055	1.481869008	0.0008	-1.741931847	0.2979	-0.260062839
Mammary gland	RS	miR-2887	-	-	0.810455549	Positive	0.3847	0.538623786	0.0765	-0.906890596	0.4677	-0.36826681
Mammary gland	CS	miR-30b-3p	-	-	0.810842931	Positive	0.6176	-0.387023123	0.6306	0.387023123	0.9002	0
Mammary gland	RS	miR-2339	-	-	0.811323731	Positive	0.1025	-1.547487795	0.2782	0.788495895	0.0683	-0.7589919
Mammary gland	RS	miR-196a	-	-	0.812221867	Positive	0.9657	0.021965331	0.7503	-0.152606605	0.7457	-0.130641274
Mammary gland	AL	miR-2443	-	-	0.813847281	Positive	0.1342	0.497499659	<.0001	-1.497499659	0.0003	-1
Mammary gland	RS	miR-2419-3p	-	-	0.821662618	Positive	0.3993	-1	0.6913	-0.201633861	0.0597	-1.201633861
Mammary gland	RS	miR-2284w	-	-	0.82383517	Positive	0.8893	0.064851144	0.1399	-0.56828376	0.0116	-0.503432615

Mammary gland	RS	miR-181a	-	-	0.824265196	Positive	0.40889	-0.475803575	0.91664	-0.048591264	0.0315	-0.524394839	
Mammary gland	AL	miR-1277	-	-	0.824853574	Positive	0.7804	0.169925001	0.1049	-1	0.1826	-0.830074999	
Mammary gland	RS	miR-2487	-	-	0.825228942	Positive	0.6201	0.256339753	0.4047	-0.397759508	0.7814	-0.141419755	
Mammary gland	CS	miR-1	-	-	0.825761698	Positive	0.3717	-3.668947171	0.379	3.435747994	0.6213	-0.233199176	
Mammary gland	AL	miR-190a	-	-	0.826361442	Positive	0.5285	0.804489588	0.8093	0.185031894	0.4277	0.989521482	Mammary Gland(Up)
Mammary gland	RS	miR-1249	-	-	0.8277963	Positive	0.1571	-0.730813367	0.2717	0.478047297	0.4605	-0.25276607	
Mammary gland	RS	miR-2419-5p	-	-	0.830833541	Positive	0.6273	-0.347355648	0.017	-1.073063462	0.0046	-1.420419111	
Mammary gland	CS	miR-299	-	-	0.832733289	Positive	0.7086	0.094636874	0.0862	0.560714954	0.0077	0.655351829	
Mammary gland	CS	miR-18a	-	-	0.83548617	Positive	0.4701	0.357956404	0.4526	-0.364411426	0.9914	-0.006455022	
Mamma	C	miR-37	-	-	0.837517	Positive	0.1	0.628780	0.1	-0.580506	0.8	0.048273	

ry gland	S	4a			716		469	461	311	609	358	852	
Mammary gland	CS	miR-6119-3p	-	-	0.837662566	Positive	0.0565	1	<.0001	-1.705256734	0.0043	-0.705256734	
Mammary gland	CS	miR-542-5p	-	-	0.840007105	Positive	0.9828	0	0.5374	0.552541023	0.1128	0.552541023	
Mammary gland	RS	miR-154c	-	-	0.842788343	Positive	0.7883	-0.145940361	0.469	-0.326128083	0.2348	-0.472068444	
Mammary gland	RS	miR-877	-	-	0.843363802	Positive	0.1727	-1.901221846	0.368	0.91186909	0.0014	-0.989352756	
Mammary gland	CS	miR-19b	-	-	0.843565154	Positive	0.4334	0.250413685	0.0586	-0.480506447	0.3722	-0.230092762	
Mammary gland	CS	miR-20a	-	-	0.845952788	Positive	0.165	0.735645049	0.0112	-1.100679169	0.2249	-0.365034119	
Mammary gland	CS	miR-6520	-	-	0.847021908	Positive	0.1981	-0.669851398	0.5141	0.222392421	0.1993	-0.447458977	
Mammary gland	CS	miR-454	-	-	0.853453682	Positive	0.2082	0.744742945	0.0257	-1.345135486	0.1345	-0.600392541	
Mammary gland	AL	miR-2285t	-	-	0.855384271	Positive	0.095	-1.325964917	0.426	0.447692687	0.026	-0.87827223	Mammary Gland(Up)

							2		2		3		
Mammary gland	CS	miR-17-5p	-	-	0.858428962	Positive	0.3934	0.455025349	0.0401	-0.872922502	0.1567	-0.417897153	
Mammary gland	CS	let-7a-3p	-	-	0.862651509	Positive	0.086	0.592364749	0.0029	-1.08851017	0.0265	-0.496145421	
Mammary gland	CS	miR-2285o	-	-	0.862954899	Positive	0.4651	0.299560282	0.2395	-0.547487795	0.5197	-0.247927513	
Mammary gland	CS	miR-190a	-	-	0.866018531	Positive	0.5285	0.804489588	0.8093	0.185031894	0.4277	0.989521482	Mammary Gland(Up)
Mammary gland	CS	miR-374b	-	-	0.869627464	Positive	0.1188	0.516556259	0.0284	-0.707292666	0.2094	-0.190736407	
Mammary gland	RS	miR-2285s	-	-	0.86988809	Positive	0.4363	-0.459431619	0.9251	0	0.4466	-0.459431619	
Mammary gland	CS	miR-429	-	-	0.871427343	Positive	0.0641	1.010327657	0.0002	-1.686638222	0.0162	-0.676310565	
Mammary gland	AL	miR-1388-5p	-	-	0.872590801	Positive	0.4088	-0.46872537	0.1128	0.819137152	0.5088	0.350411782	
Mammary gland	RS	let-7c	-	-	0.874157965	Positive	0.3283	-0.481822244	0.9725	0.013234807	0.0358	-0.468587437	Mammary Gland(Up)

Mammary gland	CS	miR-1296	-	-	0.878245831	Positive	0.7847	-0.099535674	0.017	-0.736965594	0.0109	-0.836501268	
Mammary gland	AL	miR-1388-3p	-	-	0.87988304	Positive	0.4256	0.397115591	0.0722	-0.729823525	0.4123	-0.332707934	
Mammary gland	RS	let-7i	-	-	0.88511502	Positive	0.2546	-0.354780371	0.9428	-0.018479857	0.0141	-0.373260228	
Mammary gland	CS	miR-26a	-	-	0.885697877	Positive	0.3684	0.365365871	0.0369	-0.720613955	0.1288	-0.355248085	
Mammary gland	CS	miR-200c	-	-	0.89069074	Positive	0.8287	0.106515234	0.7314	-0.150959026	0.8532	-0.044443792	Mammary Gland(Up)
Mammary gland	CS	miR-146a	-	-	0.911311087	Positive	0.9313	-0.046824712	0.6895	0.187025774	0.697	0.140201063	Mammary Gland(Down)
Mammary gland	CS	miR-24	-	-	0.919813858	Positive	0.0741	0.722466024	0.001	-1.137503524	0.0902	-0.415037499	
Mammary gland	CS	miR-126-3p	-	-	0.920598227	Positive	0.0595	0.985862336	0.003	-1.454957808	0.1407	-0.469095471	
Mammary gland	CS	miR-361	-	-	0.937044652	Positive	0.4513	0.326134206	0.09	-0.625628861	0.2016	-0.299494656	
Mamma	C	miR-15	-	-	0.945404	Positive	0.7	-0.180572	0.6	-0.197939	0.3	-0.378511	

ry gland	S	4b			38		931	246	787	378	889	623	
Mammary gland	RS	miR-381	-	-	0.946233339	Positive	0.2312	-0.328180627	0.3295	0.25403086	0.769	-0.074149767	
Mammary gland	CS	miR-503-3p	-	-	0.94646288	Positive	0.9798	0	0.4669	-0.530514717	0.3639	-0.530514717	
Mammary gland	CS	miR-19a	-	-	0.949207697	Positive	0.2258	0.508485843	0.0074	-0.92314203	0.1908	-0.414656187	
Mammary gland	CS	miR-376e	-	-	0.966833127	Positive	0.1018	0.790546634	0.0404	-0.919829651	0.663	-0.129283017	
Mammary gland	CS	miR-106b	-	-	0.983521504	Positive	0.0516	0.997729826	<.0001	-1.740137848	0.0136	-0.742408021	
Mammary gland	RS	let-7e	-	0.905704885	-	Positive	0.7273	0.132979992	0.0177	-0.715373837	0.0091	-0.582393845	
Mammary gland	RS	miR-1298	0.888841871	-	-	Positive	0.9982	0	0.802	0.38332864	0.7654	0.38332864	Mammary gland(Specific)
Mammary gland	RS	miR-2411-5p	-	0.866328523	-	Positive	0.8179	-0.09557766	0.4906	0.331205908	0.3539	0.235628248	
Mammary gland	RS	miR-2478	-	0.810006097	-	Positive	0.619	0.258160865	0.003	-1.225352471	0.012	-0.967191607	

								5		3		9
Mammary gland	RS	miR-375	-	0.846831164	-	Positive	0.6073	-0.172688424	0.2981	-0.332095854	0.093	-0.504784278
Mammary gland	CS	miR-1388-3p	-	0.815193271	-	Positive	0.4256	0.397115591	0.0722	-0.729823525	0.4123	-0.332707934
Mammary gland	CS	miR-15b	0.840518415	-	-	Positive	0.256	0.33493896	0.0004	-1.032309766	0.0012	-0.697370806
Mammary gland	CS	miR-17-3p	-	0.908967803	-	Positive	0.371	0.316672369	0.0005	-0.973527789	0.0084	-0.656855419
Mammary gland	CS	miR-2285c	-	0.836621997	-	Positive	0.6752	0.2410081	0.2208	-0.504042505	0.4844	-0.263034406
Mammary gland	CS	miR-2316	0.891567818	-	-	Positive	0.3906	-0.447458977	0.7479	0.206450877	0.4952	-0.2410081
Mammary gland	CS	miR-363	0.858247111	-	-	Positive	0.8509	0.097297201	0.0319	-0.975833689	0.0018	-0.878536488
Mammary gland	CS	miR-484	0.893590218	-	-	Positive	0.0333	0.68468675	<.0001	-1.30914194	0.025	-0.62445519
Mammary gland	AL	miR-101	-	0.974881971	-	Positive	0.6874	-0.129064275	0.9712	-0.009405395	0.4512	-0.13846967

							0.2		0.7		0.1	
Mamma	A	miR-12	0.88832	0.937224			47		17	0.293731	81	-0.706268
ry gland	L	47-5p	9363	327	-	Positive	2	-1	2	203	3	797
							0.7				0.0	
Mamma	A	miR-12		0.878621			84	-0.099535	0.0	-0.736965	10	-0.836501
ry gland	L	96	-	954	-	Positive	7	674	17	594	9	268
							0.7		0.2		0.0	
Mamma	A	miR-13	0.86493	0.897954			26	-0.171766	43	-0.451379	09	-0.623145
ry gland	L	06	053	729	-	Positive	7	348	3	346	4	695
							0.3				0.0	
Mamma	A	miR-14		0.886361			95	-0.650200	0.9	-0.009209	07	-0.659410
ry gland	L	68	-	706	-	Positive	3	437	88	718	9	156
							0.1		0.0		0.2	
Mamma	A	miR-14	0.84513	0.857176			55	-0.352848	49	0.519884	36	0.167035
ry gland	L	8a	6126	74	-	Positive	4	857	1	027	8	171
							0.9				0.0	
Mamma	A	miR-15	0.89494				72	-0.020157	0.0	-1.125232	08	-1.145390
ry gland	L	0	6955	-	-	Positive	1	744	24	971	4	714
							0.1		0.0			
Mamma	A	miR-16		0.835238			05	0.669139	04	-1.098308	0.0	-0.429168
ry gland	L	b	-	43	-	Positive	7	854	1	7	27	846
							0.4		0.9		0.0	
Mamma	A	miR-18	0.81701	0.916627			08	-0.475803	16	-0.048591	31	-0.524394
ry gland	L	1a	8451	147	-	Positive	9	575	4	264	5	839
							0.2		0.7		0.0	
Mamma	A	miR-18		0.942521			89	-0.846788	72	0.180701	18	-0.666087
ry gland	L	1b	-	794	-	Positive	9	872	7	738	5	134
Mamma	A	miR-19	0.99146	0.906234	-	Positive	0.9	0.021965	0.7	-0.152606	0.7	-0.130641

ry gland	L	6a	3631	743			657	331	503	605	457	274	
Mammary gland	AL	miR-196b	0.903129456	-	-	Positive	0.9149	-0.059172444	0.8549	0.087323484	0.9435	0.02815104	Mammary gland(Specific)
Mammary gland	AL	miR-205	0.812304593	0.91409554	-	Positive	0.2763	-0.659488929	0.964	-0.023327576	0.0866	-0.682816505	
Mammary gland	AL	miR-210	-	0.855113971	-	Positive	0.3127	-0.385772905	0.6153	-0.140725025	0.0101	-0.52649793	
Mammary gland	AL	miR-222	-	0.854600497	-	Positive	0.536	-0.241098616	0.0633	-0.608988775	0.0089	-0.850087391	
Mammary gland	AL	miR-2284k	0.898494917	0.984410359	-	Positive	0.1559	-0.662965013	0.966	0	0.1375	-0.662965013	
Mammary gland	AL	miR-2284n	-	0.983911273	-	Positive	0.2543	0.716207034	0.0107	-1.321928095	0.1	-0.605721061	
Mammary gland	AL	miR-2284w	0.849184661	0.829965361	-	Positive	0.8893	0.064851144	0.1399	-0.56828376	0.0116	-0.503432615	
Mammary gland	AL	miR-2284x	0.906512288	0.915377525	-	Positive	0.1223	-0.888042166	0.2037	0.656290539	0.1369	-0.231751627	
Mammary gland	AL	miR-2284y	-	0.814563556	-	Positive	0.156	-0.699295176	0.673	0.164781698	0.009	-0.534513478	

							6		6		6	
Mammary gland	AL	miR-2313-3p	0.89006282	-	-	Positive	0.4948	-0.637429921	0.7564	0.222392421	0.5546	-0.415037499
Mammary gland	AL	miR-2332	-	0.927052826	-	Positive	0.2849	-1.520832163	0.5061	0.722466024	0.0496	-0.798366139
Mammary gland	AL	miR-2339	-	0.827939239	-	Positive	0.1025	-1.547487795	0.2782	0.788495895	0.0683	-0.7589919
Mammary gland	AL	miR-25	-	0.902499792	-	Positive	0.119	-0.348422999	0.3356	0.174909437	0.1655	-0.173513562
Mammary gland	AL	miR-27b	-	0.921262387	-	Positive	0.0191	0.423176047	0.0001	-0.690582722	0.0254	-0.267406675
Mammary gland	AL	miR-296	0.87217731	-	-	Positive	0.1424	-1.006426269	0.3929	0.465053037	0.0264	-0.541373232
Mammary gland	AL	miR-31	0.934942573	-	-	Positive	0.5825	0.293692678	0.0202	-0.813302897	0.1497	-0.519610219
Mammary gland	AL	miR-320a	-	0.837440709	-	Positive	0.4592	-0.351069768	0.6775	-0.155479138	0.0186	-0.506548906
Mammary gland	AL	miR-328	-	0.948885107	-	Positive	0.3062	-0.430634354	0.2163	-0.358686645	0.0052	-0.789320999

Mammary gland	AL	miR-331	-	0.813005068	-	Positive	0.317	0.391234475	0.0014	-0.850908502	0.0947	-0.459674027	
Mammary gland	AL	miR-34b	0.958272004	0.813546103	-	Positive	0.2958	0.453717967	0.0336	-0.96829114	0.1085	-0.514573173	
Mammary gland	AL	miR-34c	0.869037462	-	-	Positive	0.0092	1.121015401	0.0007	-1.852284265	0.0671	-0.731268864	Mammary Gland(Up)
Mammary gland	AL	miR-363	-	0.951769778	-	Positive	0.8509	0.097297201	0.0319	-0.975833689	0.0018	-0.878536488	
Mammary gland	AL	miR-378b	0.825392403	-	-	Positive	0.2716	-0.459431619	0.8145	-0.073063462	0.0733	-0.532495081	
Mammary gland	AL	miR-450b	0.841494007	-	-	Positive	0.2568	0.576042122	0.0857	-0.733678335	0.5766	-0.157636213	
Mammary gland	AL	miR-504	0.863094876	-	-	Positive	0.3468	-0.41219475	0.9108	0.034557222	0.251	-0.377637527	
Mammary gland	AL	miR-592	-	0.899539891	-	Positive	0.9221	0.078002512	0.1242	-0.637429921	0.2262	-0.559427409	
Mammary gland	AL	miR-6119-5p	0.813972736	0.834502588	-	Positive	0.5475	0.204873299	0.0013	-0.851630901	0.0072	-0.646757602	
Mamma	A	miR-65	-	0.937734	-	Positive	0.1	-0.669851	0.5	0.222392	0.1	-0.447458	

ry gland	L	20		227			981	398	141	421	993	977
Mammary gland	AL	miR-744	0.872176912	-	-	Positive	0.2419	-0.703606997	0.6472	-0.195015982	0.0323	-0.89862298
Mammary gland	AL	miR-769	-	0.925622888	-	Positive	0.4808	-0.177573917	0.6223	-0.105127522	0.1542	-0.282701439
Mammary gland	AL	miR-92b	0.871037404	0.819980196	-	Positive	0.3003	0.250512486	0.0037	-0.593486573	0.1227	-0.342974087
Mammary gland	AL	miR-95	0.953757175	0.850683187	-	Positive	0.6158	-0.353636955	0.5881	-0.309328058	0.0629	-0.662965013
Mammary gland	AL	miR-9-5p	0.911839926	0.839179592	-	Positive	0.3954	-0.826668397	0.4299	0.721698838	0.8493	-0.10496956
Mammary gland	AL	miR-99a-3p	-	0.865219426	-	Positive	0.6567	-0.164786278	0.8139	-0.070328042	0.2688	-0.23511432
Mammary gland	RS	miR-127	-	-0.821070122	-	Negative	0.964	-0.019658431	0.2727	0.584484329	0.0145	0.564825899
Mammary gland	RS	miR-1271	-	-0.849925525	-	Negative	0.2173	-1.16549003	0.106	1.764469109	0.4405	0.598979079
Mammary gland	RS	miR-148b	-	-0.853224048	-	Negative	0.632	-0.193217323	0.112	0.591910797	0.251	0.398693474

							2		1		8	
Mammary gland	RS	miR-15b	-0.856055826	-	-	Negative	0.256	0.33493896	0.0004	-1.032309766	0.0012	-0.697370806
Mammary gland	RS	miR-183	-	-0.810880756	-	Negative	0.5989	-0.578125071	0.1733	1.715305519	0.3602	1.137180448
Mammary gland	RS	miR-18a	-	-0.838470924	-	Negative	0.4701	0.357956404	0.4526	-0.364411426	0.9914	-0.006455022
Mammary gland	RS	miR-191	-	-0.913615749	-	Negative	0.892	0.074002561	0.5992	0.272778268	0.3068	0.346780828
Mammary gland	RS	miR-192	-0.930353353	-0.969122217	-	Negative	0.098	0.223469962	0.1882	-0.159565951	0.5612	0.063904012
Mammary gland	RS	miR-21-3p	-	-0.934168356	-	Negative	0.6873	-0.285043027	0.2057	0.714155711	0.5732	0.429112684
Mammary gland	RS	miR-2285k	-	-0.874544796	-	Negative	0.3702	-0.363470357	0.2472	0.529348526	0.3638	0.165878168
Mammary gland	RS	miR-2313-3p	-	-0.859431057	-	Negative	0.4948	-0.637429921	0.7564	0.222392421	0.5546	-0.415037499
Mammary gland	RS	miR-29d	-0.842676149	-0.841986008	-	Negative	0.1518	0.876425315	0.0004	-1.46529227	0.0946	-0.588866955

Mammary gland	RS	miR-411c-3p	-0.900532844	-0.831536311	-	Negative	0.2797	0.757143476	0.7947	0.14974712	0.1568	0.906890596	
Mammary gland	RS	miR-449a	-0.883565605	-	-	Negative	0.2274	-0.36923381	0.3204	0.36923381	0.938	0	
Mammary gland	RS	miR-874	-	-0.816597489	-	Negative	0.9873	-0.008878151	0.7224	0.209176802	0.1144	0.20029865	
Mammary gland	RS	miR-96	-	-0.893378412	-	Negative	0.0866	0.878364348	0.001	-1.409327839	0.079	-0.53096349	Mammary Gland(Up)
Mammary gland	RS	miR-99a-5p	-	-0.929175073	-	Negative	0.8514	-0.132228905	0.5459	0.437150101	0.55	0.304921196	Mammary Gland(Up)
Mammary gland	CS	let-7b	-0.814585517	-0.828583347	-	Negative	0.86	-0.061446624	0.1137	-0.420345663	0.0334	-0.481792287	
Mammary gland	CS	let-7c	-	-0.851422284	-	Negative	0.3283	-0.481822244	0.9725	0.013234807	0.0358	-0.468587437	Mammary Gland(Up)
Mammary gland	CS	miR-1298	-0.974500864	-	-	Negative	0.9982	0	0.802	0.38332864	0.7654	0.38332864	Mammary gland(Specific)
Mammary gland	CS	miR-132	-0.850109916	-	-	Negative	0.8018	0.107484427	0.3729	-0.364340258	0.3441	-0.256855831	
Mamma	C	miR-14	-0.8699	-	-	Negative	0.3	-0.281598	0.9	-0.018264	0.1	-0.299862	

ry gland	S	0	21215				709	156	446	234	925	39
Mammary gland	CS	miR-149-5p	-0.858695374	-	-	Negative	0.5502	-0.394717515	0.231	-0.600229257	0.0215	-0.994946772
Mammary gland	CS	miR-188	-0.827355142	-	-	Negative	0.8948	0.048363022	0.0213	-0.769387072	0.0212	-0.72102405
Mammary gland	CS	miR-193a-5p	-0.827222944	-	-	Negative	0.7482	-0.201047519	0.8797	0.072149786	0.7466	-0.128897733
Mammary gland	CS	miR-199c	-0.845547182	-	-	Negative	0.4228	0.393622911	0.0766	-0.755496877	0.2277	-0.361873966
Mammary gland	CS	miR-378c	-0.839337869	-	-	Negative	0.4922	-0.304334035	0.5488	-0.190430657	0.054	-0.494764692
Mammary gland	CS	miR-382	-0.859898807	-	-	Negative	0.3812	-1.425305835	0.3927	1.38466385	0.8644	-0.040641984
Mammary gland	CS	miR-424-5p	-0.846397003	-	-	Negative	0.1698	0.703256233	0.3799	-0.468188978	0.4045	0.235067254
Mammary gland	CS	miR-432	-0.836374536	-	-	Negative	0.3868	-0.459431619	0.367	0.459431619	0.9982	0
Mammary gland	CS	miR-450a	-0.956437415	-	-	Negative	0.075	0.972920466	0.030	-1.069821449	0.780	-0.096900984

							5		3		4		
Mammary gland	CS	miR-450b	-0.944365241	-	-	Negative	0.2568	0.576042122	0.0857	-0.733678335	0.5766	-0.157636213	
Mammary gland	CS	miR-455-3p	-0.883139774	-	-	Negative	0.6589	0.229481846	0.261	-0.55987152	0.3591	-0.330389674	Mammary Gland(Down)
Mammary gland	CS	miR-483	-0.987576005	-	-	Negative	0.874	0.169925001	0.194	-0.700439718	0.3039	-0.530514717	
Mammary gland	CS	miR-582	-0.85271451	-	-	Negative	0.0509	0.839535328	0.005	-1.306661338	0.1396	-0.46712601	
Mammary gland	CS	miR-6531	-0.828498744	-	-	Negative	0.3437	0.584962501	0.5599	-0.485426827	0.8001	0.099535674	
Mammary gland	CS	miR-655	-0.857616788	-	-	Negative	0.1758	0.812914447	0.1724	-0.709409872	0.8025	0.103504576	
Mammary gland	CS	miR-760-3p	-0.898380811	-	-	Negative	0.5507	-0.398549376	0.5374	-0.311944006	0.1263	-0.710493383	
Mammary gland	CS	miR-885	-0.885721659	-	-	Negative	0.9918	0.007455805	0.0548	-0.823988656	0.0489	-0.816532851	
Mammary gland	AL	miR-10b	-	-0.8528438	-	Negative	0.7565	-0.127953009	0.0107	1.000285677	0.0845	0.872332668	Mammary Gland(Up)

Mammary gland	AL	miR-1271	-	-0.837195801	-	Negative	0.2173	-1.16549003	0.106	1.764469109	0.4405	0.598979079	
Mammary gland	AL	miR-146b	-	-0.884138619	-	Negative	0.1113	-1.247750918	0.0572	1.780410768	0.2624	0.53265985	
Mammary gland	AL	miR-99a-5p	-	-0.870173156	-	Negative	0.8514	-0.132228905	0.5459	0.437150101	0.55	0.304921196	Mammary Gland(Up)
Mammary gland	AL	miR-99b	-0.851284443	-0.923209297	-	Negative	0.4254	-0.538375624	0.5248	0.426524989	0.7471	-0.111850636	

Table S8. Amino acids related functions targeted by microRNAs associated with feed efficiency, nitrogen efficiency or milk urea nitrogen content in rumen, duodenum, jejunum, liver and mammary gland.

Amino acids related functions					
**Tissue**	**Diet**	**Correlation**	**Function**	**FDR**	**number of miRNA**
Rumen	Alfalfa hay	Positive	transport of amino acids	1.53E-04	13
Rumen	Alfalfa hay	Positive	metabolism of amino acids	5.15E-03	13
Rumen	Alfalfa hay	Negative	release of L-amino acid	4.51E-04	13
Rumen	Rice straw	Negative	phosphorylation of L-amino acid	2.72E-06	18
Rumen	Rice straw	Negative	transport of amino acids	8.01E-06	13
Rumen	Rice straw	Negative	release of amino acids	5.01E-05	17
Duodenum	Alfalfa hay	Positive	transport of amino acids	4.30E-02	12
Duodenum	Alfalfa hay	Negative	phosphorylation of L-amino acid	3.93E-09	68
Duodenum	Alfalfa hay	Negative	release of L-amino acid	8.89E-06	58
Duodenum	Corn stover	Positive	release of amino acids	9.01E-05	32
Duodenum	Corn stover	Negative	transport of amino acids	4.15E-05	22
Duodenum	Corn stover	Negative	phosphorylation of L-amino acid	5.30E-05	30
Duodenum	Corn stover	Negative	abnormal quantity of amino acids	2.55E-04	26
Jejunum	Alfalfa hay	Positive	transport of alpha-amino acid	2.45E-04	17
Jejunum	Alfalfa hay	Negative	phosphorylation of L-amino acid	5.45E-04	20
Jejunum	Alfalfa hay	Negative	transport of amino acids	7.96E-04	20
Jejunum	Corn stover	Positive	phosphorylation of L-amino acid	4.11E-04	17
Jejunum	Rice straw	Positive	phosphorylation of L-amino acid	8.20E-07	14

Jejunum	Rice straw	Positive	transport of amino acids	1.59E-05	15
Jejunum	Rice straw	Negative	transport of amino acids	1.36E-03	6
Liver	Alfalfa hay	Positive	phosphorylation of amino acids	2.36E-06	14
Liver	Alfalfa hay	Negative	phosphorylation of L-amino acid	2.62E-05	13
Liver	Corn stover	Positive	phosphorylation of L-amino acid	3.45E-06	36
Liver	Rice straw	Negative	phosphorylation of L-amino acid	2.26E-06	29
Mammary	Alfalfa hay	Positive	phosphorylation of amino acids	1.83E-08	44
Mammary	Alfalfa hay	Negative	transport of amino acids	9.23E-03	10
Mammary	Corn stover	Positive	phosphorylation of L-amino acid	7.76E-04	13
Mammary	Corn stover	Positive	release of amino acids	2.74E-03	14
Mammary	Corn stover	Negative	phosphorylation of L-amino acid	3.93E-08	49
Mammary	Rice straw	Negative	phosphorylation of L-amino acid	2.66E-06	31
Mammary	Rice straw	Negative	transport of amino acids	3.91E-04	28

All the Functions

Tissue	Diet	Correlation	Diseases or Functions Annotation	p-Value	# Molecules
Rumen	Alfalfa hay	Positive	organismal death	3.95E-22	643
Rumen	Alfalfa hay	Positive	morbidity or mortality	1.15E-21	647
Rumen	Alfalfa hay	Positive	cancer	1.51E-18	2319
Rumen	Alfalfa hay	Positive	proliferation of cells	4.61E-18	900
Rumen	Alfalfa hay	Positive	malignant solid tumor	7.81E-18	2290
Rumen	Alfalfa hay	Positive	cell death	4.53E-17	819
Rumen	Alfalfa hay	Positive	morphology of cells	5.03E-16	513
Rumen	Alfalfa	Positive	transport of molecule	8.27E-16	437

	hay				
Rumen	Alfalfa hay	Positive	necrosis	1.48E-14	642
Rumen	Alfalfa hay	Positive	apoptosis	6.68E-13	648
Rumen	Alfalfa hay	Positive	quantity of cells	1.96E-10	446
Rumen	Alfalfa hay	Positive	abnormal morphology of cells	1.51E-09	330
Rumen	Alfalfa hay	Positive	quantity of leukocytes	1.73E-09	250
Rumen	Alfalfa hay	Positive	cell death of connective tissue cells	1.74E-09	175
Rumen	Alfalfa hay	Positive	epithelial cancer	2.12E-09	1867
Rumen	Alfalfa hay	Positive	transcription	2.72E-09	488
Rumen	Alfalfa hay	Positive	expression of RNA	3.17E-09	520
Rumen	Alfalfa hay	Positive	tumorigenesis of tissue	4.81E-09	1909
Rumen	Alfalfa hay	Positive	neoplasia of epithelial tissue	4.81E-09	1880
Rumen	Alfalfa hay	Positive	quantity of blood cells	4.81E-09	273
Rumen	Alfalfa hay	Positive	development of body trunk	5.21E-09	293
Rumen	Alfalfa hay	Positive	organization of cytoplasm	5.63E-09	385
Rumen	Alfalfa hay	Positive	cellular homeostasis	7.29E-09	382
Rumen	Alfalfa hay	Positive	concentration of lipid	1.12E-08	221
Rumen	Alfalfa hay	Positive	cell movement	1.36E-08	511
Rumen	Alfalfa hay	Positive	perinatal death	2.36E-08	168
Rumen	Alfalfa hay	Positive	Movement Disorders	2.90E-08	276
Rumen	Alfalfa hay	Positive	digestive system cancer	5.13E-08	1604
Rumen	Alfalfa hay	Positive	digestive organ tumor	5.13E-08	1618
Rumen	Alfalfa	Positive	organization of cytoskeleton	5.28E-08	349

	hay				
Rumen	Alfalfa hay	Positive	Growth Failure	7.03E-08	167
Rumen	Alfalfa hay	Positive	transcription of RNA	8.10E-08	448
Rumen	Alfalfa hay	Positive	abdominal neoplasm	1.17E-07	1854
Rumen	Alfalfa hay	Positive	synthesis of lipid	1.56E-07	193
Rumen	Alfalfa hay	Positive	vasculogenesis	1.74E-07	198
Rumen	Alfalfa hay	Positive	development of lymphatic system	1.93E-07	106
Rumen	Alfalfa hay	Positive	abdominal cancer	2.97E-07	1830
Rumen	Alfalfa hay	Positive	Organ Degeneration	2.97E-07	139
Rumen	Alfalfa hay	Positive	development of blood cells	3.11E-07	177
Rumen	Alfalfa hay	Positive	morphology of cardiovascular system	3.39E-07	169
Rumen	Alfalfa hay	Positive	migration of cells	3.55E-07	453
Rumen	Alfalfa hay	Positive	quantity of mononuclear leukocytes	3.57E-07	196
Rumen	Alfalfa hay	Positive	uptake of monosaccharide	3.57E-07	83
Rumen	Alfalfa hay	Positive	microtubule dynamics	3.99E-07	297
Rumen	Alfalfa hay	Positive	differentiation of cells	4.03E-07	526
Rumen	Alfalfa hay	Positive	development of leukocytes	4.31E-07	161
Rumen	Alfalfa hay	Positive	size of body	4.56E-07	222
Rumen	Alfalfa hay	Positive	uptake of carbohydrate	4.66E-07	88
Rumen	Alfalfa hay	Positive	autosomal recessive disease	7.39E-07	237
Rumen	Alfalfa hay	Positive	development of lymphatic system component	7.39E-07	92
Rumen	Alfalfa hay	Positive	cell movement of tumor cell lines	7.48E-07	221
Rumen	Alfalfa	Positive	cell death of fibroblast cell lines	7.65E-07	121

	hay				
Rumen	Alfalfa hay	Positive	abnormal morphology of thoracic cavity	9.04E-07	159
Rumen	Alfalfa hay	Positive	cell survival	9.96E-07	340
Rumen	Alfalfa hay	Positive	formation of cytoskeleton	1.02E-06	108
Rumen	Alfalfa hay	Positive	quantity of lymphocytes	1.02E-06	187
Rumen	Alfalfa hay	Positive	formation of lymphatic system component	1.20E-06	87
Rumen	Alfalfa hay	Positive	cell death of tumor cell lines	1.23E-06	368
Rumen	Alfalfa hay	Positive	development of vasculature	1.25E-06	118
Rumen	Alfalfa hay	Positive	organization of organelle	1.26E-06	147
Rumen	Alfalfa hay	Positive	cell viability	1.26E-06	319
Rumen	Alfalfa hay	Positive	development of cytoplasm	1.69E-06	128
Rumen	Alfalfa hay	Positive	morphology of bone	1.75E-06	125
Rumen	Alfalfa hay	Positive	uptake of D-hexose	1.91E-06	69
Rumen	Alfalfa hay	Positive	angiogenesis	1.99E-06	231
Rumen	Alfalfa hay	Positive	formation of cells	2.07E-06	248
Rumen	Alfalfa hay	Positive	dyskinesia	2.38E-06	164
Rumen	Alfalfa hay	Positive	neuromuscular disease	2.55E-06	225
Rumen	Alfalfa hay	Positive	abnormal morphology of bone	2.55E-06	121
Rumen	Alfalfa hay	Positive	uptake of D-glucose	2.55E-06	68
Rumen	Alfalfa hay	Positive	development of connective tissue	2.80E-06	105
Rumen	Alfalfa hay	Positive	abnormal morphology of cardiovascular system	2.83E-06	152
Rumen	Alfalfa hay	Positive	growth of organism	2.86E-06	214
Rumen	Alfalfa	Positive	neurological signs	3.76E-06	171

	hay				
Rumen	Alfalfa hay	Positive	Huntington's Disease	3.94E-06	153
Rumen	Alfalfa hay	Positive	abnormal morphology of head	4.87E-06	212
Rumen	Alfalfa hay	Positive	formation of lymphoid organ	5.08E-06	78
Rumen	Alfalfa hay	Positive	neonatal death	6.65E-06	120
Rumen	Alfalfa hay	Positive	development of lymphocytes	6.65E-06	147
Rumen	Alfalfa hay	Positive	abnormal morphology of epithelial tissue	6.65E-06	115
Rumen	Alfalfa hay	Positive	fatty acid metabolism	6.65E-06	158
Rumen	Alfalfa hay	Positive	epilepsy	7.35E-06	88
Rumen	Alfalfa hay	Positive	epileptic seizure	8.72E-06	53
Rumen	Alfalfa hay	Positive	neuronal cell death	9.18E-06	176
Rumen	Alfalfa hay	Positive	Lymphocyte homeostasis	1.10E-05	144
Rumen	Alfalfa hay	Positive	transcription of DNA	1.10E-05	363
Rumen	Alfalfa hay	Positive	T cell development	1.10E-05	134
Rumen	Alfalfa hay	Positive	disorder of basal ganglia	1.13E-05	195
Rumen	Alfalfa hay	Positive	proliferation of blood cells	1.16E-05	212
Rumen	Alfalfa hay	Positive	homeostasis of leukocytes	1.23E-05	146
Rumen	Alfalfa hay	Positive	degeneration of cells	1.23E-05	96
Rumen	Alfalfa hay	Positive	accumulation of lipid	1.23E-05	85
Rumen	Alfalfa hay	Positive	degeneration of nervous system	1.33E-05	82
Rumen	Alfalfa hay	Positive	quantity of phagocytes	1.34E-05	115
Rumen	Alfalfa hay	Positive	morphology of body cavity	1.36E-05	289
Rumen	Alfalfa	Positive	morphology of head	1.36E-05	219

	hay				
Rumen	Alfalfa hay	Positive	formation of actin filaments	1.59E-05	86
Rumen	Alfalfa hay	Positive	apoptosis of tumor cell lines	1.60E-05	292
Rumen	Alfalfa hay	Positive	abnormal morphology of body cavity	1.64E-05	277
Rumen	Alfalfa hay	Positive	abnormal morphology of skull	1.76E-05	65
Rumen	Alfalfa hay	Positive	function of leukocytes	1.78E-05	141
Rumen	Alfalfa hay	Positive	proliferation of neuronal cells	1.79E-05	150
Rumen	Alfalfa hay	Positive	T cell homeostasis	1.79E-05	136
Rumen	Alfalfa hay	Positive	differentiation of connective tissue	2.02E-05	185
Rumen	Alfalfa hay	Positive	Viral Infection	2.03E-05	377
Rumen	Alfalfa hay	Positive	proliferation of immune cells	2.07E-05	199
Rumen	Alfalfa hay	Positive	transport of alpha-amino acid	2.15E-05	27
Rumen	Alfalfa hay	Positive	migration of blood cells	2.22E-05	217
Rumen	Alfalfa hay	Positive	apoptosis of fibroblast cell lines	2.27E-05	91
Rumen	Alfalfa hay	Positive	leukocyte migration	2.94E-05	216
Rumen	Alfalfa hay	Positive	abnormal morphology of abdomen	3.03E-05	217
Rumen	Alfalfa hay	Positive	seizure disorder	3.27E-05	124
Rumen	Alfalfa hay	Positive	concentration of phospholipid	3.91E-05	55
Rumen	Alfalfa hay	Positive	apoptosis of epithelial cell lines	3.91E-05	65
Rumen	Alfalfa hay	Positive	ubiquitination of protein	3.91E-05	91
Rumen	Alfalfa hay	Positive	morphology of vessel	3.98E-05	85
Rumen	Alfalfa hay	Positive	proliferation of connective tissue cells	4.21E-05	152
Rumen	Alfalfa	Positive	ubiquitination	4.41E-05	92

	hay				
Rumen	Alfalfa hay	Positive	cell death of cervical cancer cell lines	5.29E-05	95
Rumen	Alfalfa hay	Positive	proliferation of tumor cell lines	5.46E-05	360
Rumen	Alfalfa hay	Positive	Neurodegeneration	5.46E-05	85
Rumen	Alfalfa hay	Positive	quantity of reactive oxygen species	5.46E-05	53
Rumen	Alfalfa hay	Positive	formation of filaments	5.46E-05	106
Rumen	Alfalfa hay	Positive	abnormal morphology of reproductive system	5.46E-05	132
Rumen	Alfalfa hay	Positive	quantity of T lymphocytes	5.59E-05	138
Rumen	Alfalfa hay	Positive	cell death of epithelial cells	6.00E-05	129
Rumen	Alfalfa hay	Positive	fibrogenesis	6.00E-05	109
Rumen	Alfalfa hay	Positive	degeneration of neurons	6.00E-05	68
Rumen	Alfalfa hay	Positive	transport of inorganic cation	6.10E-05	95
Rumen	Alfalfa hay	Positive	transport of carbohydrate	6.15E-05	57
Rumen	Alfalfa hay	Positive	necrosis of epithelial tissue	6.34E-05	150
Rumen	Alfalfa hay	Positive	Edema	6.38E-05	93
Rumen	Alfalfa hay	Positive	formation of actin stress fibers	6.65E-05	68
Rumen	Alfalfa hay	Positive	activation of cells	6.78E-05	237
Rumen	Alfalfa hay	Positive	protein kinase cascade	7.00E-05	111
Rumen	Alfalfa hay	Positive	morphology of respiratory system	7.01E-05	85
Rumen	Alfalfa hay	Positive	differentiation of connective tissue cells	7.22E-05	162
Rumen	Alfalfa hay	Positive	metabolism of carbohydrate	7.32E-05	157
Rumen	Alfalfa hay	Positive	transport of metal	7.92E-05	90
Rumen	Alfalfa	Positive	transactivation	7.94E-05	147

	hay				
Rumen	Alfalfa hay	Positive	morphology of heart	8.02E-05	107
Rumen	Alfalfa hay	Positive	growth of connective tissue	8.12E-05	162
Rumen	Alfalfa hay	Positive	cellular degradation	8.62E-05	71
Rumen	Alfalfa hay	Positive	concentration of fatty acid	8.97E-05	77
Rumen	Alfalfa hay	Positive	development of abdomen	9.01E-05	147
Rumen	Alfalfa hay	Positive	transport of cation	9.41E-05	106
Rumen	Alfalfa hay	Positive	quantity of connective tissue	1.03E-04	152
Rumen	Alfalfa hay	Positive	cell movement of leukocytes	1.03E-04	189
Rumen	Alfalfa hay	Positive	generation of reactive oxygen species	1.06E-04	59
Rumen	Alfalfa hay	Positive	cell spreading	1.06E-04	76
Rumen	Alfalfa hay	Positive	oral squamous cell carcinoma	1.08E-04	52
Rumen	Alfalfa hay	Positive	transport of metal ion	1.15E-04	86
Rumen	Alfalfa hay	Positive	function of blood cells	1.15E-04	148
Rumen	Alfalfa hay	Positive	abnormal morphology of respiratory system	1.18E-04	83
Rumen	Alfalfa hay	Positive	morphology of reproductive system	1.28E-04	136
Rumen	Alfalfa hay	Positive	activation of blood cells	1.29E-04	184
Rumen	Alfalfa hay	Positive	cell death of breast cancer cell lines	1.34E-04	89
Rumen	Alfalfa hay	Positive	cell death of epithelial cell lines	1.34E-04	77
Rumen	Alfalfa hay	Positive	quantity of steroid	1.35E-04	120
Rumen	Alfalfa hay	Positive	differentiation of leukocytes	1.35E-04	167
Rumen	Alfalfa hay	Positive	transport of ion	1.35E-04	129
Rumen	Alfalfa	Positive	proliferation of fibroblast cell lines	1.39E-04	110

	hay				
Rumen	Alfalfa hay	Positive	differentiation of mononuclear leukocytes	1.41E-04	137
Rumen	Alfalfa hay	Positive	formation of cellular protrusions	1.45E-04	218
Rumen	Alfalfa hay	Positive	differentiation of lymphocytes	1.46E-04	127
Rumen	Alfalfa hay	Positive	accumulation of steroid	1.52E-04	28
Rumen	Alfalfa hay	Positive	transport of amino acids	1.53E-04	41
Rumen	Alfalfa hay	Positive	seizures	1.62E-04	104
Rumen	Alfalfa hay	Positive	transport of monosaccharide	1.64E-04	47
Rumen	Alfalfa hay	Positive	apoptosis of kidney cell lines	1.80E-04	66
Rumen	Alfalfa hay	Positive	proliferation of ovarian cancer cell lines	1.82E-04	41
Rumen	Alfalfa hay	Positive	quantity of antigen presenting cells	1.86E-04	75
Rumen	Alfalfa hay	Positive	demyelination of central nervous system	1.91E-04	18
Rumen	Alfalfa hay	Positive	morphology of endothelial tissue	1.91E-04	28
Rumen	Alfalfa hay	Positive	HIV infection	1.91E-04	175
Rumen	Alfalfa hay	Positive	chronic myeloid leukemia	1.92E-04	34
Rumen	Alfalfa hay	Positive	cell movement of myeloid cells	1.98E-04	136
Rumen	Alfalfa hay	Positive	homing of cells	1.99E-04	140
Rumen	Alfalfa hay	Positive	abnormal quantity of lipid	2.01E-04	44
Rumen	Alfalfa hay	Positive	morphology of digestive system	2.01E-04	143
Rumen	Alfalfa hay	Positive	morphology of cardiovascular tissue	2.08E-04	29
Rumen	Alfalfa hay	Positive	proliferation of lymphocytes	2.08E-04	178
Rumen	Alfalfa hay	Positive	synthesis of reactive oxygen species	2.14E-04	122
Rumen	Alfalfa	Positive	synthesis of fatty acid	2.18E-04	83

	hay				
Rumen	Alfalfa hay	Positive	abnormal morphology of body wall	2.18E-04	33
Rumen	Alfalfa hay	Positive	depolarization of mitochondrial membrane	2.25E-04	14
Rumen	Alfalfa hay	Positive	morphology of blood vessel	2.25E-04	76
Rumen	Alfalfa hay	Positive	infection by Retroviridae	2.27E-04	176
Rumen	Alfalfa hay	Positive	apoptosis of embryonic cell lines	2.28E-04	52
Rumen	Alfalfa hay	Positive	morphology of genital organ	2.28E-04	109
Rumen	Alfalfa hay	Positive	mass of organism	2.31E-04	88
Rumen	Alfalfa hay	Positive	transport of L-amino acid	2.31E-04	23
Rumen	Alfalfa hay	Positive	demyelination	2.37E-04	37
Rumen	Alfalfa hay	Positive	transport of sterol	2.39E-04	39
Rumen	Alfalfa hay	Positive	proliferation of mononuclear leukocytes	2.40E-04	180
Rumen	Alfalfa hay	Positive	binding of DNA	2.47E-04	131
Rumen	Alfalfa hay	Positive	apoptosis of breast cancer cell lines	2.47E-04	77
Rumen	Alfalfa hay	Positive	depolarization of cellular membrane	2.55E-04	27
Rumen	Alfalfa hay	Positive	metabolism of reactive oxygen species	2.62E-04	126
Rumen	Alfalfa hay	Positive	abnormal morphology of digestive system	2.82E-04	136
Rumen	Alfalfa hay	Positive	oral cavity carcinoma	2.85E-04	55
Rumen	Alfalfa hay	Positive	abnormal morphology of heart	2.88E-04	99
Rumen	Alfalfa hay	Positive	cellular infiltration by leukocytes	2.88E-04	100
Rumen	Alfalfa hay	Positive	concentration of sterol	2.95E-04	81
Rumen	Alfalfa hay	Positive	interphase	3.08E-04	161
Rumen	Alfalfa	Positive	growth of neurites	3.08E-04	117

	hay				
Rumen	Alfalfa hay	Positive	quantity of IgM	3.21E-04	41
Rumen	Alfalfa hay	Positive	targeting of protein	3.29E-04	39
Rumen	Alfalfa hay	Positive	urination disorder	3.29E-04	77
Rumen	Alfalfa hay	Positive	migration of tumor cell lines	3.39E-04	172
Rumen	Alfalfa hay	Positive	behavior	3.43E-04	234
Rumen	Alfalfa hay	Positive	abnormal morphology of rib	3.52E-04	24
Rumen	Alfalfa hay	Positive	secretory pathway	3.60E-04	55
Rumen	Alfalfa hay	Positive	concentration of cholesterol	3.60E-04	77
Rumen	Alfalfa hay	Positive	growth of embryo	3.67E-04	120
Rumen	Alfalfa hay	Positive	activation of leukocytes	3.70E-04	170
Rumen	Alfalfa hay	Positive	oral cancer	3.70E-04	62
Rumen	Alfalfa hay	Positive	abnormal morphology of rib cage	3.70E-04	29
Rumen	Alfalfa hay	Positive	cell cycle progression	3.80E-04	246
Rumen	Alfalfa hay	Positive	transport of carboxylic acid	3.81E-04	36
Rumen	Alfalfa hay	Positive	quantity of lymphatic system component	3.87E-04	97
Rumen	Alfalfa hay	Positive	dysgenesis	3.89E-04	134
Rumen	Alfalfa hay	Positive	metabolism of protein	3.92E-04	230
Rumen	Alfalfa hay	Positive	morphology of rib	3.92E-04	25
Rumen	Alfalfa hay	Positive	proliferation of embryonic cells	4.00E-04	67
Rumen	Alfalfa hay	Positive	abnormal morphology of genital organ	4.08E-04	104
Rumen	Alfalfa hay	Positive	transport of D-glucose	4.10E-04	42
Rumen	Alfalfa	Positive	colony formation of cells	4.24E-04	123

	hay				
Rumen	Alfalfa hay	Positive	attachment of cells	4.28E-04	40
Rumen	Alfalfa hay	Positive	colony formation	4.46E-04	133
Rumen	Alfalfa hay	Positive	transport of glutamine family amino acid	4.64E-04	20
Rumen	Alfalfa hay	Positive	cartilage development	4.78E-04	42
Rumen	Alfalfa hay	Positive	binding of lymphatic system cells	4.97E-04	12
Rumen	Alfalfa hay	Positive	cytopenia	5.23E-04	78
Rumen	Alfalfa hay	Positive	quantity of B lymphocytes	5.27E-04	89
Rumen	Alfalfa hay	Positive	morphology of vesicles	5.28E-04	14
Rumen	Alfalfa hay	Positive	invasion of tissue	5.33E-04	54
Rumen	Alfalfa hay	Positive	autosomal dominant disease	5.76E-04	155
Rumen	Alfalfa hay	Positive	apoptosis of cervical cancer cell lines	5.90E-04	74
Rumen	Alfalfa hay	Positive	transport of cholesterol	6.23E-04	37
Rumen	Alfalfa hay	Positive	Hypoplasia	6.23E-04	125
Rumen	Alfalfa hay	Positive	morphology of connective tissue	6.23E-04	115
Rumen	Alfalfa hay	Positive	long-term potentiation	6.27E-04	65
Rumen	Alfalfa hay	Positive	quantity of thymocytes	6.27E-04	62
Rumen	Alfalfa hay	Positive	quantity of carbohydrate	6.27E-04	128
Rumen	Alfalfa hay	Positive	morphology of axial skeleton	6.31E-04	55
Rumen	Alfalfa hay	Positive	mitosis	6.31E-04	123
Rumen	Alfalfa hay	Positive	synthesis of testosterone	6.44E-04	19
Rumen	Alfalfa hay	Positive	abnormal morphology of blood vessel	6.44E-04	70
Rumen	Alfalfa	Positive	transport of L-glutamic acid	6.62E-04	13

	hay				
Rumen	Alfalfa hay	Positive	development of cardiovascular tissue	6.63E-04	94
Rumen	Alfalfa hay	Positive	infection of cells	6.69E-04	190
Rumen	Alfalfa hay	Positive	retinal degeneration	6.72E-04	68
Rumen	Alfalfa hay	Positive	differentiation of blood cells	6.89E-04	200
Rumen	Alfalfa hay	Positive	length of cells	7.02E-04	31
Rumen	Alfalfa hay	Positive	proliferation of fibroblasts	7.16E-04	90
Rumen	Alfalfa hay	Positive	transmembrane potential of mitochondria	7.23E-04	58
Rumen	Alfalfa hay	Positive	growth of embryonic tissue	7.31E-04	72
Rumen	Alfalfa hay	Positive	phosphorylation of protein	7.34E-04	176
Rumen	Alfalfa hay	Positive	cognition	7.64E-04	113
Rumen	Alfalfa hay	Positive	function of lymphocytes	7.70E-04	87
Rumen	Alfalfa hay	Positive	accumulation of sterol	7.71E-04	23
Rumen	Alfalfa hay	Positive	recruitment of neutrophils	7.78E-04	51
Rumen	Alfalfa hay	Positive	abnormal morphology of axial skeleton	7.78E-04	53
Rumen	Alfalfa hay	Positive	dysmyelination	7.78E-04	39
Rumen	Alfalfa hay	Positive	dyspnea	7.78E-04	39
Rumen	Alfalfa hay	Positive	transactivation of RNA	8.13E-04	133
Rumen	Alfalfa hay	Positive	abnormal morphology of internal genitalia	8.28E-04	89
Rumen	Alfalfa hay	Positive	abnormal morphology of pulmonary alveolus	8.45E-04	34
Rumen	Alfalfa hay	Positive	hypersensitive reaction	8.67E-04	107
Rumen	Alfalfa hay	Positive	female genital tract serous cancer	8.72E-04	97
Rumen	Alfalfa	Positive	mammary tumor	8.72E-04	369

	hay				
Rumen	Alfalfa hay	Positive	transport of monovalent inorganic cation	8.81E-04	58
Rumen	Alfalfa hay	Positive	concentration of phosphatidic acid	9.00E-04	39
Rumen	Alfalfa hay	Positive	recruitment of phagocytes	9.01E-04	65
Rumen	Alfalfa hay	Positive	chemotaxis of cells	9.24E-04	129
Rumen	Alfalfa hay	Positive	infection by RNA virus	9.27E-04	204
Rumen	Alfalfa hay	Positive	quantity of lymphoid organ	9.32E-04	79
Rumen	Alfalfa hay	Positive	export of molecule	9.99E-04	76
Rumen	Alfalfa hay	Positive	synthesis of terpenoid	1.06E-03	73
Rumen	Alfalfa hay	Positive	congenital anomaly of musculoskeletal system	1.07E-03	175
Rumen	Alfalfa hay	Positive	apoptosis of connective tissue cells	1.10E-03	76
Rumen	Alfalfa hay	Positive	infection by HIV-1	1.10E-03	147
Rumen	Alfalfa hay	Positive	flux of lipid	1.10E-03	37
Rumen	Alfalfa hay	Positive	differentiation of T lymphocytes	1.10E-03	95
Rumen	Alfalfa hay	Positive	neurodegeneration of neurites	1.10E-03	28
Rumen	Alfalfa hay	Positive	morphology of muscle	1.13E-03	85
Rumen	Alfalfa hay	Positive	B-cell leukemia	1.16E-03	34
Rumen	Alfalfa hay	Positive	transport of steroid	1.17E-03	43
Rumen	Alfalfa hay	Positive	cell movement of macrophages	1.19E-03	69
Rumen	Alfalfa hay	Positive	proliferation of muscle cells	1.19E-03	93
Rumen	Alfalfa hay	Positive	formation of thymus gland	1.20E-03	45
Rumen	Alfalfa hay	Positive	transport of acidic amino acid	1.24E-03	16
Rumen	Alfalfa	Positive	hypoplasia of organ	1.25E-03	110

	hay				
Rumen	Alfalfa hay	Positive	formation of leukocytes	1.26E-03	44
Rumen	Alfalfa hay	Positive	quantity of macrophages	1.27E-03	54
Rumen	Alfalfa hay	Positive	formation of reactive oxygen species	1.27E-03	27
Rumen	Alfalfa hay	Positive	synthesis of nucleotide	1.29E-03	91
Rumen	Alfalfa hay	Positive	Hypertrophy	1.29E-03	116
Rumen	Alfalfa hay	Positive	synthesis of carbohydrate	1.32E-03	105
Rumen	Alfalfa hay	Positive	stress response of cells	1.37E-03	39
Rumen	Alfalfa hay	Positive	differentiation of epithelial tissue	1.40E-03	90
Rumen	Alfalfa hay	Positive	cytostasis of tumor cell lines	1.43E-03	44
Rumen	Alfalfa hay	Positive	neurotransmission	1.47E-03	102
Rumen	Alfalfa hay	Positive	relaxation of muscle	1.48E-03	26
Rumen	Alfalfa hay	Positive	chronic B-cell leukemia	1.50E-03	23
Rumen	Alfalfa hay	Positive	formation of lymphocytes	1.50E-03	36
Rumen	Alfalfa hay	Positive	cell death of kidney cell lines	1.52E-03	77
Rumen	Alfalfa hay	Positive	concentration of choline-phospholipid	1.53E-03	18
Rumen	Alfalfa hay	Positive	secretion of molecule	1.53E-03	133
Rumen	Alfalfa hay	Positive	serous neoplasm	1.53E-03	146
Rumen	Alfalfa hay	Positive	function of smooth muscle	1.57E-03	28
Rumen	Alfalfa hay	Positive	hepatocellular carcinoma	1.59E-03	892
Rumen	Alfalfa hay	Positive	function of cardiovascular system	1.59E-03	92
Rumen	Alfalfa hay	Positive	binding of endothelial cells	1.61E-03	31
Rumen	Alfalfa	Positive	repair of DNA	1.61E-03	74

	hay				
Rumen	Alfalfa hay	Positive	recruitment of cells	1.61E-03	89
Rumen	Alfalfa hay	Positive	congenital malformation of skeleton	1.61E-03	104
Rumen	Alfalfa hay	Positive	formation of mononuclear leukocytes	1.66E-03	37
Rumen	Alfalfa hay	Positive	necroptosis of fibroblast cell lines	1.67E-03	22
Rumen	Alfalfa hay	Positive	cell movement of granulocytes	1.70E-03	92
Rumen	Alfalfa hay	Positive	migration of muscle cells	1.70E-03	45
Rumen	Alfalfa hay	Positive	abnormal morphology of nervous system	1.70E-03	171
Rumen	Alfalfa hay	Positive	development of endothelial tissue	1.70E-03	91
Rumen	Alfalfa hay	Positive	bone mineral density	1.70E-03	47
Rumen	Alfalfa hay	Positive	efflux of lipid	1.74E-03	35
Rumen	Alfalfa hay	Positive	metabolism of nucleoside triphosphate	1.75E-03	39
Rumen	Alfalfa hay	Positive	dephosphorylation of protein	1.78E-03	50
Rumen	Alfalfa hay	Positive	cell movement of phagocytes	1.83E-03	132
Rumen	Alfalfa hay	Positive	survival of organism	1.83E-03	166
Rumen	Alfalfa hay	Positive	organization of nucleus	1.84E-03	32
Rumen	Alfalfa hay	Positive	abnormal morphology of jaw	1.85E-03	29
Rumen	Alfalfa hay	Positive	recruitment of macrophages	1.85E-03	31
Rumen	Alfalfa hay	Positive	synthesis of cholesterol	1.85E-03	21
Rumen	Alfalfa hay	Positive	cell death of kidney cells	1.85E-03	87
Rumen	Alfalfa hay	Positive	cellular infiltration	1.85E-03	107
Rumen	Alfalfa hay	Positive	transmigration of cells	1.85E-03	43
Rumen	Alfalfa	Positive	morphology of vertebral column	1.88E-03	40

	hay				
Rumen	Alfalfa hay	Positive	development of genitourinary system	1.88E-03	206
Rumen	Alfalfa hay	Positive	binding of blood vessel	1.88E-03	8
Rumen	Alfalfa hay	Positive	invasion of cells	1.92E-03	202
Rumen	Alfalfa hay	Positive	activation of lymphocytes	1.93E-03	112
Rumen	Alfalfa hay	Positive	locally advanced carcinoma	1.93E-03	9
Rumen	Alfalfa hay	Positive	morphology of skeleton	1.95E-03	71
Rumen	Alfalfa hay	Positive	development of hematopoietic system	1.96E-03	64
Rumen	Alfalfa hay	Positive	breast or ovarian cancer	1.99E-03	422
Rumen	Alfalfa hay	Positive	cell movement of neutrophils	1.99E-03	75
Rumen	Alfalfa hay	Positive	arrest in mitosis of tumor cell lines	1.99E-03	22
Rumen	Alfalfa hay	Positive	cell viability of cerebral cortex cells	1.99E-03	22
Rumen	Alfalfa hay	Positive	hepatobiliary system cancer	2.02E-03	922
Rumen	Alfalfa hay	Positive	quantity of epinephrine	2.04E-03	17
Rumen	Alfalfa hay	Positive	apoptosis of endothelial cells	2.04E-03	49
Rumen	Alfalfa hay	Positive	chronic myeloproliferative disorder	2.05E-03	43
Rumen	Alfalfa hay	Positive	abnormal morphology of skeleton	2.06E-03	69
Rumen	Alfalfa hay	Positive	apoptosis of dermal cells	2.07E-03	27
Rumen	Alfalfa hay	Positive	apoptosis of cerebral cortex cells	2.09E-03	31
Rumen	Alfalfa hay	Positive	apoptosis of skin	2.09E-03	28
Rumen	Alfalfa hay	Positive	cell death of hematopoietic progenitor cells	2.09E-03	59
Rumen	Alfalfa hay	Positive	morphology of nervous system	2.09E-03	183
Rumen	Alfalfa	Positive	stimulation of hepatoma cell lines	2.09E-03	6

	hay				
Rumen	Alfalfa hay	Positive	morphology of jaw	2.09E-03	30
Rumen	Alfalfa hay	Positive	attachment of tumor cell lines	2.11E-03	16
Rumen	Alfalfa hay	Positive	generation of lymphocytes	2.17E-03	39
Rumen	Alfalfa hay	Positive	learning	2.17E-03	102
Rumen	Alfalfa hay	Positive	apoptosis of neurons	2.19E-03	106
Rumen	Alfalfa hay	Positive	craniofacial abnormality	2.19E-03	88
Rumen	Alfalfa hay	Positive	quantity of natural killer T lymphocytes	2.21E-03	21
Rumen	Alfalfa hay	Positive	abnormal morphology of adipose tissue	2.27E-03	34
Rumen	Alfalfa hay	Positive	recruitment of myeloid cells	2.28E-03	59
Rumen	Alfalfa hay	Positive	internalization of lipid	2.29E-03	11
Rumen	Alfalfa hay	Positive	liver cancer	2.31E-03	917
Rumen	Alfalfa hay	Positive	cell death of embryonic cells	2.31E-03	33
Rumen	Alfalfa hay	Positive	metabolism of membrane lipid derivative	2.36E-03	95
Rumen	Alfalfa hay	Positive	cell death of hematopoietic cells	2.36E-03	61
Rumen	Alfalfa hay	Positive	function of muscle	2.37E-03	86
Rumen	Alfalfa hay	Positive	accumulation of triacylglycerol	2.42E-03	27
Rumen	Alfalfa hay	Positive	autophagy	2.42E-03	92
Rumen	Alfalfa hay	Positive	cell tethering or rolling	2.44E-03	29
Rumen	Alfalfa hay	Positive	morphology of endothelial cells	2.47E-03	20
Rumen	Alfalfa hay	Positive	quantity of subcutaneous fat	2.47E-03	20
Rumen	Alfalfa hay	Positive	metabolism of sphingolipid	2.54E-03	38
Rumen	Alfalfa	Positive	autophagy of cells	2.54E-03	67

	hay				
Rumen	Alfalfa hay	Positive	cell death of fibroblasts	2.54E-03	67
Rumen	Alfalfa hay	Positive	exocytosis	2.56E-03	50
Rumen	Alfalfa hay	Positive	proliferation of T lymphocytes	2.57E-03	143
Rumen	Alfalfa hay	Positive	differentiation of epithelial cells	2.63E-03	78
Rumen	Alfalfa hay	Positive	outgrowth of cells	2.65E-03	105
Rumen	Alfalfa hay	Positive	hyperplasia of tissue	2.66E-03	51
Rumen	Alfalfa hay	Positive	transport of long chain fatty acid	2.66E-03	12
Rumen	Alfalfa hay	Positive	G1 phase	2.66E-03	95
Rumen	Alfalfa hay	Positive	breast or colorectal cancer	2.66E-03	1047
Rumen	Alfalfa hay	Positive	synthesis of sphingolipid	2.71E-03	32
Rumen	Alfalfa hay	Positive	quantity of hematopoietic cells	2.71E-03	107
Rumen	Alfalfa hay	Positive	metabolism of peptide	2.71E-03	49
Rumen	Alfalfa hay	Positive	maturation of cells	2.73E-03	110
Rumen	Alfalfa hay	Positive	activation of mononuclear leukocytes	2.76E-03	116
Rumen	Alfalfa hay	Positive	length of neurons	2.78E-03	26
Rumen	Alfalfa hay	Positive	Dermatitis	2.78E-03	105
Rumen	Alfalfa hay	Positive	cell death of skin	2.78E-03	30
Rumen	Alfalfa hay	Positive	liver lesion	2.78E-03	922
Rumen	Alfalfa hay	Positive	accumulation of acylglycerol	2.81E-03	28
Rumen	Alfalfa hay	Positive	outgrowth of neurons	2.84E-03	99
Rumen	Alfalfa hay	Positive	abnormal morphology of lung	2.87E-03	48
Rumen	Alfalfa	Positive	apoptosis of hippocampus	2.89E-03	14

	hay				
Rumen	Alfalfa hay	Positive	synthesis of DNA	2.94E-03	101
Rumen	Alfalfa hay	Positive	neoplasia of lymphoid organ	2.94E-03	42
Rumen	Alfalfa hay	Positive	syndromic mental retardation	2.94E-03	42
Rumen	Alfalfa hay	Positive	morphology of mouth	2.95E-03	37
Rumen	Alfalfa hay	Positive	liver tumor	2.95E-03	921
Rumen	Alfalfa hay	Positive	morphology of capillary vessel	2.99E-03	18
Rumen	Alfalfa hay	Positive	accumulation of cholesterol	3.00E-03	20
Rumen	Alfalfa hay	Positive	outgrowth of neurites	3.05E-03	98
Rumen	Alfalfa hay	Positive	synthesis of steroid	3.15E-03	65
Rumen	Alfalfa hay	Positive	efflux of sterol	3.15E-03	31
Rumen	Alfalfa hay	Positive	necrosis of kidney	3.20E-03	89
Rumen	Alfalfa hay	Positive	psoriatic arthritis	3.22E-03	25
Rumen	Alfalfa hay	Positive	vascularization	3.23E-03	60
Rumen	Alfalfa hay	Positive	cardiogenesis	3.23E-03	113
Rumen	Alfalfa hay	Positive	cell death of embryonic cell lines	3.27E-03	61
Rumen	Alfalfa hay	Positive	proliferation of lymphatic system cells	3.27E-03	52
Rumen	Alfalfa hay	Positive	morphology of lung	3.27E-03	49
Rumen	Alfalfa hay	Positive	morphology of gonad	3.28E-03	92
Rumen	Alfalfa hay	Positive	apoptosis of neuroblastoma cell lines	3.29E-03	34
Rumen	Alfalfa hay	Positive	concentration of ATP	3.29E-03	34
Rumen	Alfalfa hay	Positive	abnormal morphology of vertebrae	3.31E-03	37
Rumen	Alfalfa	Positive	abnormal morphology of hepatobiliary system	3.33E-03	53

	hay				
Rumen	Alfalfa hay	Positive	morphology of vessel component	3.35E-03	19
Rumen	Alfalfa hay	Positive	morphology of heart ventricle	3.36E-03	50
Rumen	Alfalfa hay	Positive	ataxia	3.36E-03	65
Rumen	Alfalfa hay	Positive	quantity of hematopoietic progenitor cells	3.41E-03	106
Rumen	Alfalfa hay	Positive	development of digestive system	3.43E-03	93
Rumen	Alfalfa hay	Positive	degeneration of central nervous system	3.44E-03	36
Rumen	Alfalfa hay	Positive	cell death of endothelial cells	3.57E-03	52
Rumen	Alfalfa hay	Positive	abnormal morphology of vertebral column	3.57E-03	38
Rumen	Alfalfa hay	Positive	concentration of acylglycerol	3.58E-03	82
Rumen	Alfalfa hay	Positive	production of reactive oxygen species	3.58E-03	89
Rumen	Alfalfa hay	Positive	angiogenesis of lesion	3.58E-03	35
Rumen	Alfalfa hay	Positive	concentration of Ca2+	3.58E-03	35
Rumen	Alfalfa hay	Positive	coagulation	3.70E-03	50
Rumen	Alfalfa hay	Positive	apoptosis of hippocampal neurons	3.71E-03	12
Rumen	Alfalfa hay	Positive	demyelination of spinal cord	3.71E-03	12
Rumen	Alfalfa hay	Positive	cell death of dermal cells	3.78E-03	28
Rumen	Alfalfa hay	Positive	formation of blood cells	3.81E-03	51
Rumen	Alfalfa hay	Positive	recruitment of granulocytes	3.81E-03	55
Rumen	Alfalfa hay	Positive	transport of lipid	3.82E-03	61
Rumen	Alfalfa hay	Positive	granulation tissue	3.83E-03	14
Rumen	Alfalfa hay	Positive	cognitive impairment	3.84E-03	81
Rumen	Alfalfa	Positive	metabolism of nucleotide	3.87E-03	105

	hay				
Rumen	Alfalfa hay	Positive	activation of neuroglia	3.88E-03	36
Rumen	Alfalfa hay	Positive	metabolism of DNA	3.96E-03	96
Rumen	Alfalfa hay	Positive	morphology of lymphatic system component	3.96E-03	100
Rumen	Alfalfa hay	Positive	mental retardation	4.06E-03	59
Rumen	Alfalfa hay	Positive	cell movement of muscle cells	4.06E-03	47
Rumen	Alfalfa hay	Positive	abnormal morphology of embryonic tissue	4.17E-03	129
Rumen	Alfalfa hay	Positive	uptake of 2-deoxyglucose	4.24E-03	30
Rumen	Alfalfa hay	Positive	atelectasis	4.30E-03	23
Rumen	Alfalfa hay	Positive	midline defect	4.31E-03	66
Rumen	Alfalfa hay	Positive	remodeling of bone	4.37E-03	49
Rumen	Alfalfa hay	Positive	apoptosis of vascular endothelial cells	4.39E-03	28
Rumen	Alfalfa hay	Positive	mitosis of cervical cancer cell lines	4.39E-03	28
Rumen	Alfalfa hay	Positive	quantity of glycosphingolipid	4.39E-03	28
Rumen	Alfalfa hay	Positive	mitosis of tumor cell lines	4.40E-03	36
Rumen	Alfalfa hay	Positive	angiogenesis of tumor	4.40E-03	33
Rumen	Alfalfa hay	Positive	neurodegeneration of axons	4.41E-03	25
Rumen	Alfalfa hay	Positive	quantity of IL-6 in blood	4.41E-03	25
Rumen	Alfalfa hay	Positive	B-cell lymphoproliferative disorder	4.46E-03	96
Rumen	Alfalfa hay	Positive	abnormal morphology of endothelial tissue	4.46E-03	20
Rumen	Alfalfa hay	Positive	formation of T lymphocytes	4.55E-03	32
Rumen	Alfalfa hay	Positive	abnormal morphology of gonad	4.69E-03	88
Rumen	Alfalfa	Positive	development of reproductive system	4.69E-03	170

	hay				
Rumen	Alfalfa hay	Positive	cell movement of leukemia cell lines	4.73E-03	37
Rumen	Alfalfa hay	Positive	bleeding of brain	4.75E-03	21
Rumen	Alfalfa hay	Positive	recruitment of leukocytes	4.80E-03	79
Rumen	Alfalfa hay	Positive	concentration of phosphatidylcholine	4.83E-03	15
Rumen	Alfalfa hay	Positive	movement of mesenchymal stem cells	4.90E-03	11
Rumen	Alfalfa hay	Positive	branching of cells	4.94E-03	89
Rumen	Alfalfa hay	Positive	binding of guanosine 5'-O-(3-thiotriphosphate)	4.96E-03	22
Rumen	Alfalfa hay	Positive	delayed hypersensitive reaction	4.96E-03	36
Rumen	Alfalfa hay	Positive	recruitment of antigen presenting cells	5.01E-03	33
Rumen	Alfalfa hay	Positive	metabolism of nucleic acid component or derivative	5.09E-03	122
Rumen	Alfalfa hay	Positive	recruitment of blood cells	5.09E-03	80
Rumen	Alfalfa hay	Positive	I-kappaB kinase/NF-kappaB cascade	5.10E-03	45
Rumen	Alfalfa hay	Positive	formation of plasma membrane projections	5.13E-03	139
Rumen	Alfalfa hay	Positive	cell rolling of blood cells	5.13E-03	24
Rumen	Alfalfa hay	Positive	synthesis of sterol	5.13E-03	24
Rumen	Alfalfa hay	Positive	binding of hematopoietic progenitor cells	5.13E-03	9
Rumen	Alfalfa hay	Positive	gap junctional intercellular communication of cells	5.13E-03	9
Rumen	Alfalfa hay	Positive	abnormal morphology of capillary vessel	5.13E-03	17
Rumen	Alfalfa hay	Positive	cell death of pericytes	5.14E-03	12
Rumen	Alfalfa hay	Positive	apoptosis of epidermal cells	5.14E-03	25
Rumen	Alfalfa hay	Positive	Ovarian Cancer and Tumors	5.15E-03	174
Rumen	Alfalfa	Positive	metabolism of amino acids	5.15E-03	43

	hay				
Rumen	Alfalfa hay	Positive	apoptosis of heart cell lines	5.16E-03	13
Rumen	Alfalfa hay	Positive	synthesis of prostaglandin E2	5.23E-03	37
Rumen	Alfalfa hay	Positive	differentiation of embryonic tissue	5.26E-03	63
Rumen	Alfalfa hay	Positive	size of lesion	5.26E-03	63
Rumen	Alfalfa hay	Positive	steroid metabolism	5.26E-03	63
Rumen	Alfalfa hay	Positive	cell viability of central nervous system cells	5.27E-03	31
Rumen	Alfalfa hay	Positive	leukocytosis	5.27E-03	31
Rumen	Alfalfa hay	Positive	replication of Human cytomegalovirus	5.33E-03	6
Rumen	Alfalfa hay	Negative	proliferation of cells	5.24E-25	1072
Rumen	Alfalfa hay	Negative	cell death	2.81E-23	974
Rumen	Alfalfa hay	Negative	cancer	2.67E-22	2716
Rumen	Alfalfa hay	Negative	necrosis	5.55E-22	774
Rumen	Alfalfa hay	Negative	malignant solid tumor	3.41E-21	2681
Rumen	Alfalfa hay	Negative	organismal death	9.45E-21	722
Rumen	Alfalfa hay	Negative	apoptosis	9.45E-21	785
Rumen	Alfalfa hay	Negative	morbidity or mortality	9.45E-21	730
Rumen	Alfalfa hay	Negative	cellular homeostasis	3.19E-18	483
Rumen	Alfalfa hay	Negative	organization of cytoplasm	1.99E-16	478
Rumen	Alfalfa hay	Negative	morphology of cells	2.51E-16	585
Rumen	Alfalfa hay	Negative	cell movement	8.58E-16	629
Rumen	Alfalfa hay	Negative	cell death of tumor cell lines	8.91E-16	473
Rumen	Alfalfa	Negative	quantity of cells	2.68E-15	534

	hay				
Rumen	Alfalfa hay	Negative	morphology of cardiovascular system	2.91E-15	221
Rumen	Alfalfa hay	Negative	cell survival	5.52E-15	434
Rumen	Alfalfa hay	Negative	apoptosis of tumor cell lines	7.08E-15	385
Rumen	Alfalfa hay	Negative	expression of RNA	8.42E-15	628
Rumen	Alfalfa hay	Negative	cell viability	9.98E-15	408
Rumen	Alfalfa hay	Negative	migration of cells	1.09E-14	565
Rumen	Alfalfa hay	Negative	Movement Disorders	5.71E-14	340
Rumen	Alfalfa hay	Negative	transport of molecule	9.98E-14	485
Rumen	Alfalfa hay	Negative	transcription	1.12E-13	583
Rumen	Alfalfa hay	Negative	organization of cytoskeleton	1.82E-13	427
Rumen	Alfalfa hay	Negative	angiogenesis	1.88E-13	296
Rumen	Alfalfa hay	Negative	development of vasculature	3.99E-13	154
Rumen	Alfalfa hay	Negative	transcription of RNA	8.71E-13	543
Rumen	Alfalfa hay	Negative	abnormal morphology of cardiovascular system	1.01E-12	197
Rumen	Alfalfa hay	Negative	microtubule dynamics	1.35E-12	367
Rumen	Alfalfa hay	Negative	disorder of basal ganglia	1.85E-12	253
Rumen	Alfalfa hay	Negative	vasculogenesis	2.49E-12	244
Rumen	Alfalfa hay	Negative	quantity of blood cells	2.49E-12	324
Rumen	Alfalfa hay	Negative	neuromuscular disease	1.15E-11	282
Rumen	Alfalfa hay	Negative	cell viability of tumor cell lines	1.27E-11	253
Rumen	Alfalfa hay	Negative	dyskinesia	1.55E-11	207
Rumen	Alfalfa	Negative	cell spreading	1.79E-11	106

	hay				
Rumen	Alfalfa hay	Negative	quantity of leukocytes	1.79E-11	290
Rumen	Alfalfa hay	Negative	neurological signs	4.61E-11	215
Rumen	Alfalfa hay	Negative	size of body	6.01E-11	270
Rumen	Alfalfa hay	Negative	formation of cellular protrusions	6.25E-11	284
Rumen	Alfalfa hay	Negative	cell movement of tumor cell lines	6.38E-11	270
Rumen	Alfalfa hay	Negative	Growth Failure	8.31E-11	199
Rumen	Alfalfa hay	Negative	morphology of vessel	1.28E-10	113
Rumen	Alfalfa hay	Negative	migration of tumor cell lines	1.94E-10	228
Rumen	Alfalfa hay	Negative	tumorigenesis of tissue	2.09E-10	2229
Rumen	Alfalfa hay	Negative	epithelial cancer	2.43E-10	2175
Rumen	Alfalfa hay	Negative	development of body trunk	2.61E-10	338
Rumen	Alfalfa hay	Negative	neoplasia of epithelial tissue	2.61E-10	2194
Rumen	Alfalfa hay	Negative	Huntington's Disease	3.04E-10	190
Rumen	Alfalfa hay	Negative	proliferation of neuronal cells	3.87E-10	190
Rumen	Alfalfa hay	Negative	cell death of connective tissue cells	1.03E-09	196
Rumen	Alfalfa hay	Negative	small GTPase mediated signal transduction	1.04E-09	73
Rumen	Alfalfa hay	Negative	morphology of blood vessel	1.14E-09	103
Rumen	Alfalfa hay	Negative	ubiquitination	1.33E-09	119
Rumen	Alfalfa hay	Negative	neuronal cell death	1.69E-09	217
Rumen	Alfalfa hay	Negative	invasion of cells	2.06E-09	268
Rumen	Alfalfa hay	Negative	transcription of DNA	2.06E-09	441
Rumen	Alfalfa	Negative	abnormal morphology of thoracic cavity	2.26E-09	190

	hay				
Rumen	Alfalfa hay	Negative	growth of organism	3.23E-09	258
Rumen	Alfalfa hay	Negative	Viral Infection	3.59E-09	459
Rumen	Alfalfa hay	Negative	seizure disorder	4.03E-09	156
Rumen	Alfalfa hay	Negative	colony formation of cells	4.78E-09	162
Rumen	Alfalfa hay	Negative	necrosis of epithelial tissue	5.59E-09	189
Rumen	Alfalfa hay	Negative	synthesis of lipid	8.40E-09	223
Rumen	Alfalfa hay	Negative	ubiquitination of protein	8.46E-09	115
Rumen	Alfalfa hay	Negative	abnormal morphology of cells	9.35E-09	368
Rumen	Alfalfa hay	Negative	morphology of heart	1.11E-08	136
Rumen	Alfalfa hay	Negative	proliferation of muscle cells	1.11E-08	126
Rumen	Alfalfa hay	Negative	development of cytoplasm	1.19E-08	152
Rumen	Alfalfa hay	Negative	quantity of lymphocytes	1.40E-08	220
Rumen	Alfalfa hay	Negative	development of epithelial tissue	1.56E-08	167
Rumen	Alfalfa hay	Negative	cell movement of blood cells	1.61E-08	265
Rumen	Alfalfa hay	Negative	function of cardiovascular system	1.61E-08	125
Rumen	Alfalfa hay	Negative	morphology of body cavity	1.93E-08	348
Rumen	Alfalfa hay	Negative	migration of blood cells	1.93E-08	264
Rumen	Alfalfa hay	Negative	cell death of cervical cancer cell lines	2.35E-08	119
Rumen	Alfalfa hay	Negative	cell death of blood cells	2.44E-08	215
Rumen	Alfalfa hay	Negative	abnormal morphology of blood vessel	2.47E-08	94
Rumen	Alfalfa hay	Negative	leukocyte migration	2.51E-08	263
Rumen	Alfalfa	Negative	proliferation of tumor cell lines	2.59E-08	437

	hay				
Rumen	Alfalfa hay	Negative	transactivation	2.79E-08	183
Rumen	Alfalfa hay	Negative	quantity of mononuclear leukocytes	2.90E-08	226
Rumen	Alfalfa hay	Negative	abdominal neoplasm	3.50E-08	2159
Rumen	Alfalfa hay	Negative	cell death of heart cells	3.96E-08	80
Rumen	Alfalfa hay	Negative	cell death of heart	4.99E-08	82
Rumen	Alfalfa hay	Negative	apoptosis of neuroblastoma cell lines	5.48E-08	50
Rumen	Alfalfa hay	Negative	cell death of immune cells	5.90E-08	204
Rumen	Alfalfa hay	Negative	colony formation	6.06E-08	170
Rumen	Alfalfa hay	Negative	activation of cells	6.06E-08	289
Rumen	Alfalfa hay	Negative	inflammatory response	6.36E-08	236
Rumen	Alfalfa hay	Negative	epilepsy	6.36E-08	105
Rumen	Alfalfa hay	Negative	degeneration of nervous system	6.36E-08	99
Rumen	Alfalfa hay	Negative	autophagy	7.64E-08	124
Rumen	Alfalfa hay	Negative	Neurodegeneration	8.68E-08	105
Rumen	Alfalfa hay	Negative	cell death of neuroblastoma cell lines	9.09E-08	71
Rumen	Alfalfa hay	Negative	abdominal cancer	9.11E-08	2131
Rumen	Alfalfa hay	Negative	perinatal death	9.87E-08	185
Rumen	Alfalfa hay	Negative	abnormal morphology of epithelial tissue	9.93E-08	136
Rumen	Alfalfa hay	Negative	Organ Degeneration	9.93E-08	157
Rumen	Alfalfa hay	Negative	endocytosis	1.03E-07	115
Rumen	Alfalfa hay	Negative	epileptic seizure	1.14E-07	63
Rumen	Alfalfa	Negative	growth of embryo	1.27E-07	152

	hay				
Rumen	Alfalfa hay	Negative	seizures	1.35E-07	130
Rumen	Alfalfa hay	Negative	migration of breast cancer cell lines	1.35E-07	86
Rumen	Alfalfa hay	Negative	protein kinase cascade	1.60E-07	136
Rumen	Alfalfa hay	Negative	formation of cytoskeleton	1.82E-07	123
Rumen	Alfalfa hay	Negative	abnormal morphology of heart	1.86E-07	125
Rumen	Alfalfa hay	Negative	behavior	1.88E-07	289
Rumen	Alfalfa hay	Negative	homing	1.95E-07	178
Rumen	Alfalfa hay	Negative	development of cardiovascular tissue	1.96E-07	121
Rumen	Alfalfa hay	Negative	growth of neurites	1.97E-07	147
Rumen	Alfalfa hay	Negative	growth of plasma membrane projections	2.21E-07	148
Rumen	Alfalfa hay	Negative	cell viability of cervical cancer cell lines	2.29E-07	78
Rumen	Alfalfa hay	Negative	cell cycle progression	2.64E-07	303
Rumen	Alfalfa hay	Negative	abnormal morphology of body cavity	3.01E-07	327
Rumen	Alfalfa hay	Negative	quantity of connective tissue	3.02E-07	185
Rumen	Alfalfa hay	Negative	development of leukocytes	3.27E-07	181
Rumen	Alfalfa hay	Negative	cell movement of connective tissue cells	3.27E-07	78
Rumen	Alfalfa hay	Negative	cell movement of leukocytes	3.29E-07	229
Rumen	Alfalfa hay	Negative	proliferation of connective tissue cells	3.48E-07	182
Rumen	Alfalfa hay	Negative	development of endothelial tissue	3.53E-07	119
Rumen	Alfalfa hay	Negative	proliferation of smooth muscle cells	3.78E-07	96
Rumen	Alfalfa hay	Negative	differentiation of cells	3.82E-07	602
Rumen	Alfalfa	Negative	cell movement of breast cancer cell lines	3.90E-07	97

	hay				
Rumen	Alfalfa hay	Negative	cell death of epithelial cell lines	4.14E-07	95
Rumen	Alfalfa hay	Negative	morphology of artery	4.14E-07	58
Rumen	Alfalfa hay	Negative	phosphorylation of protein	4.14E-07	220
Rumen	Alfalfa hay	Negative	cell transformation	4.67E-07	145
Rumen	Alfalfa hay	Negative	cell movement of fibroblasts	4.77E-07	66
Rumen	Alfalfa hay	Negative	cell death of cardiomyocytes	4.87E-07	75
Rumen	Alfalfa hay	Negative	sprouting	5.24E-07	124
Rumen	Alfalfa hay	Negative	necrosis of cardiac muscle	5.30E-07	76
Rumen	Alfalfa hay	Negative	digestive system cancer	6.73E-07	1847
Rumen	Alfalfa hay	Negative	homing of cells	6.75E-07	171
Rumen	Alfalfa hay	Negative	cell death of fibroblast cell lines	6.77E-07	135
Rumen	Alfalfa hay	Negative	abnormal morphology of artery	7.05E-07	56
Rumen	Alfalfa hay	Negative	transactivation of RNA	7.17E-07	167
Rumen	Alfalfa hay	Negative	synthesis of DNA	7.56E-07	132
Rumen	Alfalfa hay	Negative	outgrowth of plasma membrane projections	8.56E-07	129
Rumen	Alfalfa hay	Negative	cell movement of endothelial cells	8.81E-07	114
Rumen	Alfalfa hay	Negative	digestive organ tumor	8.98E-07	1862
Rumen	Alfalfa hay	Negative	development of blood cells	9.20E-07	196
Rumen	Alfalfa hay	Negative	apoptosis of heart cells	9.20E-07	67
Rumen	Alfalfa hay	Negative	formation of muscle	9.20E-07	126
Rumen	Alfalfa hay	Negative	fibrogenesis	9.20E-07	130
Rumen	Alfalfa	Negative	outgrowth of neurons	9.20E-07	129

	hay				
Rumen	Alfalfa hay	Negative	outgrowth of neurites	9.20E-07	128
Rumen	Alfalfa hay	Negative	synthesis of nitric oxide	9.72E-07	92
Rumen	Alfalfa hay	Negative	growth of epithelial tissue	1.06E-06	203
Rumen	Alfalfa hay	Negative	metabolism of carbohydrate	1.14E-06	187
Rumen	Alfalfa hay	Negative	cell movement of myeloid cells	1.16E-06	165
Rumen	Alfalfa hay	Negative	cell death of epithelial cells	1.30E-06	153
Rumen	Alfalfa hay	Negative	cell death of kidney cells	1.38E-06	112
Rumen	Alfalfa hay	Negative	development of lymphatic system	1.39E-06	114
Rumen	Alfalfa hay	Negative	cell death of carcinoma cell lines	1.39E-06	94
Rumen	Alfalfa hay	Negative	concentration of lipid	1.44E-06	237
Rumen	Alfalfa hay	Negative	cell death of tumor	1.44E-06	121
Rumen	Alfalfa hay	Negative	endothelial cell development	1.55E-06	113
Rumen	Alfalfa hay	Negative	necrosis of kidney	1.58E-06	116
Rumen	Alfalfa hay	Negative	quantity of T lymphocytes	1.58E-06	163
Rumen	Alfalfa hay	Negative	outgrowth of cells	1.73E-06	135
Rumen	Alfalfa hay	Negative	apoptosis of blood cells	1.73E-06	152
Rumen	Alfalfa hay	Negative	survival of organism	1.73E-06	208
Rumen	Alfalfa hay	Negative	apoptosis of epithelial cell lines	1.95E-06	76
Rumen	Alfalfa hay	Negative	growth of connective tissue	1.96E-06	192
Rumen	Alfalfa hay	Negative	cell viability of cerebral cortex cells	1.98E-06	30
Rumen	Alfalfa hay	Negative	necrosis of tumor	1.99E-06	120
Rumen	Alfalfa	Negative	chemotaxis	2.20E-06	165

	hay				
Rumen	Alfalfa hay	Negative	fatty acid metabolism	2.23E-06	180
Rumen	Alfalfa hay	Negative	proliferation of embryonic stem cell lines	2.29E-06	15
Rumen	Alfalfa hay	Negative	proliferation of fibroblasts	2.44E-06	112
Rumen	Alfalfa hay	Negative	function of blood cells	2.44E-06	176
Rumen	Alfalfa hay	Negative	recruitment of cells	2.44E-06	113
Rumen	Alfalfa hay	Negative	development of lymphocytes	2.49E-06	167
Rumen	Alfalfa hay	Negative	apoptosis of cervical cancer cell lines	2.52E-06	92
Rumen	Alfalfa hay	Negative	development of mononuclear leukocytes	2.66E-06	168
Rumen	Alfalfa hay	Negative	formation of cells	2.71E-06	280
Rumen	Alfalfa hay	Negative	formation of actin stress fibers	2.79E-06	80
Rumen	Alfalfa hay	Negative	aggregation of cells	2.82E-06	104
Rumen	Alfalfa hay	Negative	secretion of molecule	3.03E-06	166
Rumen	Alfalfa hay	Negative	formation of filaments	3.05E-06	124
Rumen	Alfalfa hay	Negative	cellular infiltration	3.12E-06	135
Rumen	Alfalfa hay	Negative	cell death of breast cancer cell lines	3.26E-06	106
Rumen	Alfalfa hay	Negative	function of leukocytes	3.26E-06	162
Rumen	Alfalfa hay	Negative	cell death of muscle	3.31E-06	109
Rumen	Alfalfa hay	Negative	apoptosis of cardiomyocytes	3.31E-06	64
Rumen	Alfalfa hay	Negative	synthesis of fatty acid	3.36E-06	100
Rumen	Alfalfa hay	Negative	cellular infiltration by leukocytes	3.36E-06	121
Rumen	Alfalfa hay	Negative	differentiation of leukocytes	3.57E-06	198
Rumen	Alfalfa	Negative	invasion of tumor cell lines	3.78E-06	196

	hay				
Rumen	Alfalfa hay	Negative	quantity of lymphatic system component	3.78E-06	118
Rumen	Alfalfa hay	Negative	proliferation of embryonic cell lines	4.22E-06	56
Rumen	Alfalfa hay	Negative	migration of endothelial cells	4.58E-06	104
Rumen	Alfalfa hay	Negative	morphology of cardiovascular tissue	4.58E-06	35
Rumen	Alfalfa hay	Negative	cell death of muscle cells	4.63E-06	106
Rumen	Alfalfa hay	Negative	cellular infiltration of cells	4.64E-06	123
Rumen	Alfalfa hay	Negative	necrosis of muscle	4.65E-06	108
Rumen	Alfalfa hay	Negative	autosomal dominant disease	4.71E-06	188
Rumen	Alfalfa hay	Negative	quantity of lymphoid organ	5.42E-06	98
Rumen	Alfalfa hay	Negative	cell death of cerebral cortex cells	5.54E-06	76
Rumen	Alfalfa hay	Negative	cell death of T lymphocytes	5.54E-06	100
Rumen	Alfalfa hay	Negative	cellular degradation	5.55E-06	83
Rumen	Alfalfa hay	Negative	metabolism of membrane lipid derivative	5.83E-06	120
Rumen	Alfalfa hay	Negative	apoptosis of leukocytes	5.83E-06	139
Rumen	Alfalfa hay	Negative	degeneration of cells	5.83E-06	108
Rumen	Alfalfa hay	Negative	chemotaxis of cells	5.95E-06	158
Rumen	Alfalfa hay	Negative	quantity of hematopoietic cells	6.16E-06	135
Rumen	Alfalfa hay	Negative	migration of connective tissue cells	6.59E-06	62
Rumen	Alfalfa hay	Negative	T cell development	6.92E-06	151
Rumen	Alfalfa hay	Negative	apoptosis of carcinoma cell lines	7.66E-06	80
Rumen	Alfalfa hay	Negative	development of neurons	7.98E-06	225
Rumen	Alfalfa	Negative	growth of lesion	8.00E-06	229

	hay				
Rumen	Alfalfa hay	Negative	quantity of hematopoietic progenitor cells	8.52E-06	134
Rumen	Alfalfa hay	Negative	cell death of kidney cell lines	8.59E-06	96
Rumen	Alfalfa hay	Negative	size of animal	8.59E-06	61
Rumen	Alfalfa hay	Negative	degeneration of neurons	9.45E-06	78
Rumen	Alfalfa hay	Negative	differentiation of neuronal progenitor cells	9.76E-06	23
Rumen	Alfalfa hay	Negative	morphology of endothelial tissue	9.98E-06	33
Rumen	Alfalfa hay	Negative	cell death of pheochromocytoma cell lines	9.98E-06	44
Rumen	Alfalfa hay	Negative	growth of tumor	1.02E-05	228
Rumen	Alfalfa hay	Negative	recruitment of blood cells	1.03E-05	103
Rumen	Alfalfa hay	Negative	apoptosis of muscle	1.15E-05	84
Rumen	Alfalfa hay	Negative	quantity of thymus gland	1.16E-05	76
Rumen	Alfalfa hay	Negative	metabolism of protein	1.30E-05	273
Rumen	Alfalfa hay	Negative	cell death of mononuclear leukocytes	1.36E-05	121
Rumen	Alfalfa hay	Negative	cell death of tumor cells	1.44E-05	114
Rumen	Alfalfa hay	Negative	hydrolysis of lipid	1.44E-05	63
Rumen	Alfalfa hay	Negative	infection by Retroviridae	1.45E-05	207
Rumen	Alfalfa hay	Negative	apoptosis of fibroblast cell lines	1.47E-05	102
Rumen	Alfalfa hay	Negative	morphology of muscle	1.47E-05	104
Rumen	Alfalfa hay	Negative	proliferation of blood cells	1.47E-05	239
Rumen	Alfalfa hay	Negative	quantity of thymocytes	1.48E-05	75
Rumen	Alfalfa hay	Negative	cell death of central nervous system cells	1.51E-05	92
Rumen	Alfalfa	Negative	apoptosis of connective tissue cells	1.53E-05	93

	hay				
Rumen	Alfalfa hay	Negative	morphology of connective tissue	1.54E-05	138
Rumen	Alfalfa hay	Negative	cardiogenesis	1.60E-05	141
Rumen	Alfalfa hay	Negative	apoptosis of muscle cells	1.65E-05	83
Rumen	Alfalfa hay	Negative	congenital anomaly of musculoskeletal system	1.65E-05	211
Rumen	Alfalfa hay	Negative	activation of DNA endogenous promoter	1.65E-05	332
Rumen	Alfalfa hay	Negative	interphase	1.65E-05	190
Rumen	Alfalfa hay	Negative	migration of fibroblasts	1.65E-05	50
Rumen	Alfalfa hay	Negative	homeostasis of leukocytes	1.75E-05	163
Rumen	Alfalfa hay	Negative	morphology of respiratory system	1.76E-05	97
Rumen	Alfalfa hay	Negative	morphology of endothelial cells	1.83E-05	26
Rumen	Alfalfa hay	Negative	activation of enzyme	1.84E-05	118
Rumen	Alfalfa hay	Negative	autophagy of cells	1.89E-05	84
Rumen	Alfalfa hay	Negative	cell death of fibroblasts	1.89E-05	84
Rumen	Alfalfa hay	Negative	vascularization	2.00E-05	76
Rumen	Alfalfa hay	Negative	Lymphocyte homeostasis	2.05E-05	160
Rumen	Alfalfa hay	Negative	cell death of lymphocytes	2.05E-05	117
Rumen	Alfalfa hay	Negative	female genital tract serous cancer	2.05E-05	117
Rumen	Alfalfa hay	Negative	development of lymphatic system component	2.20E-05	96
Rumen	Alfalfa hay	Negative	T cell homeostasis	2.30E-05	152
Rumen	Alfalfa hay	Negative	consumption of oxygen	2.34E-05	53
Rumen	Alfalfa hay	Negative	differentiation of muscle cell lines	2.38E-05	55
Rumen	Alfalfa	Negative	Thrombosis	2.38E-05	57

	hay				
Rumen	Alfalfa hay	Negative	recruitment of leukocytes	2.50E-05	100
Rumen	Alfalfa hay	Negative	morphology of head	2.58E-05	246
Rumen	Alfalfa hay	Negative	Bleeding	2.67E-05	127
Rumen	Alfalfa hay	Negative	activation of leukocytes	2.68E-05	200
Rumen	Alfalfa hay	Negative	abnormal morphology of respiratory system	2.76E-05	95
Rumen	Alfalfa hay	Negative	cell death of hematopoietic cell lines	2.82E-05	74
Rumen	Alfalfa hay	Negative	apoptosis of hematopoietic cell lines	2.82E-05	67
Rumen	Alfalfa hay	Negative	morphogenesis of neurons	2.92E-05	127
Rumen	Alfalfa hay	Negative	cell movement of granulocytes	3.27E-05	112
Rumen	Alfalfa hay	Negative	infection by RNA virus	3.27E-05	243
Rumen	Alfalfa hay	Negative	neuritogenesis	3.31E-05	169
Rumen	Alfalfa hay	Negative	differentiation of blood cells	3.33E-05	237
Rumen	Alfalfa hay	Negative	quantity of myeloid cells	3.35E-05	108
Rumen	Alfalfa hay	Negative	activation of blood cells	3.35E-05	212
Rumen	Alfalfa hay	Negative	cell death of cortical neurons	3.62E-05	58
Rumen	Alfalfa hay	Negative	growth of lymphatic system component	3.62E-05	63
Rumen	Alfalfa hay	Negative	size of embryo	3.73E-05	108
Rumen	Alfalfa hay	Negative	organization of organelle	3.93E-05	157
Rumen	Alfalfa hay	Negative	proliferation of fibroblast cell lines	3.96E-05	126
Rumen	Alfalfa hay	Negative	infection by lentivirus	3.96E-05	203
Rumen	Alfalfa hay	Negative	cell movement of hepatoma cell lines	3.97E-05	34
Rumen	Alfalfa	Negative	neurodegeneration of neurites	3.97E-05	34

	hay				
Rumen	Alfalfa hay	Negative	cleavage of lipid	3.97E-05	65
Rumen	Alfalfa hay	Negative	formation of eye	3.97E-05	130
Rumen	Alfalfa hay	Negative	formation of plasma membrane projections	4.05E-05	172
Rumen	Alfalfa hay	Negative	transmembrane potential of mitochondria	4.08E-05	69
Rumen	Alfalfa hay	Negative	proliferation of immune cells	4.12E-05	223
Rumen	Alfalfa hay	Negative	proliferation of hematopoietic cells	4.23E-05	80
Rumen	Alfalfa hay	Negative	differentiation of muscle	4.26E-05	93
Rumen	Alfalfa hay	Negative	neonatal death	4.32E-05	130
Rumen	Alfalfa hay	Negative	ruffling	4.51E-05	39
Rumen	Alfalfa hay	Negative	proliferation of lymphatic system cells	4.58E-05	65
Rumen	Alfalfa hay	Negative	HIV infection	4.60E-05	202
Rumen	Alfalfa hay	Negative	development of body axis	4.61E-05	289
Rumen	Alfalfa hay	Negative	paraproteinemia	4.66E-05	102
Rumen	Alfalfa hay	Negative	cell death of myeloma cell lines	5.03E-05	35
Rumen	Alfalfa hay	Negative	formation of lamellipodia	5.07E-05	54
Rumen	Alfalfa hay	Negative	development of hematopoietic system	5.24E-05	78
Rumen	Alfalfa hay	Negative	cell death of endothelial cells	5.29E-05	65
Rumen	Alfalfa hay	Negative	advanced malignant tumor	5.57E-05	236
Rumen	Alfalfa hay	Negative	apoptosis of B-lymphocyte derived cell lines	5.65E-05	43
Rumen	Alfalfa hay	Negative	hypertrophy of heart	5.81E-05	102
Rumen	Alfalfa hay	Negative	mass of organism	5.83E-05	101
Rumen	Alfalfa	Negative	recruitment of macrophages	5.84E-05	38

	hay				
Rumen	Alfalfa hay	Negative	cell death of malignant tumor	5.94E-05	92
Rumen	Alfalfa hay	Negative	formation of actin filaments	5.94E-05	93
Rumen	Alfalfa hay	Negative	cell death of leukocyte cell lines	6.10E-05	65
Rumen	Alfalfa hay	Negative	aggregation of blood platelets	6.14E-05	62
Rumen	Alfalfa hay	Negative	cell viability of neuroblastoma cell lines	6.24E-05	25
Rumen	Alfalfa hay	Negative	I-kappaB kinase/NF-kappaB cascade	6.24E-05	57
Rumen	Alfalfa hay	Negative	quantity of B lymphocytes	6.24E-05	104
Rumen	Alfalfa hay	Negative	blood protein disorder	6.31E-05	112
Rumen	Alfalfa hay	Negative	concentration of Ca2+	6.35E-05	44
Rumen	Alfalfa hay	Negative	invasion of tissue	6.36E-05	63
Rumen	Alfalfa hay	Negative	proliferation of hematopoietic progenitor cells	6.36E-05	76
Rumen	Alfalfa hay	Negative	quantity of IL-6 in blood	6.42E-05	32
Rumen	Alfalfa hay	Negative	development of head	6.50E-05	270
Rumen	Alfalfa hay	Negative	apoptosis of leukocyte cell lines	6.55E-05	58
Rumen	Alfalfa hay	Negative	function of blood vessel	6.64E-05	23
Rumen	Alfalfa hay	Negative	autophagy of fibroblast cell lines	6.70E-05	18
Rumen	Alfalfa hay	Negative	transformation of fibroblast cell lines	6.74E-05	80
Rumen	Alfalfa hay	Negative	apoptosis of neurons	6.82E-05	128
Rumen	Alfalfa hay	Negative	apoptosis of fibroblasts	6.86E-05	69
Rumen	Alfalfa hay	Negative	plasma cell dyscrasia	6.92E-05	101
Rumen	Alfalfa hay	Negative	uptake of monosaccharide	6.92E-05	83
Rumen	Alfalfa	Negative	cell death of brain cells	6.97E-05	84

	hay				
Rumen	Alfalfa hay	Negative	apoptosis of T lymphocytes	7.05E-05	86
Rumen	Alfalfa hay	Negative	apoptosis of breast cancer cell lines	7.09E-05	88
Rumen	Alfalfa hay	Negative	mitosis	7.09E-05	144
Rumen	Alfalfa hay	Negative	cell death of brain	7.09E-05	89
Rumen	Alfalfa hay	Negative	metastasis	7.13E-05	213
Rumen	Alfalfa hay	Negative	cell movement of phagocytes	7.20E-05	158
Rumen	Alfalfa hay	Negative	cell death of B-lymphocyte derived cell lines	7.45E-05	44
Rumen	Alfalfa hay	Negative	catabolism of protein	7.45E-05	172
Rumen	Alfalfa hay	Negative	function of macrophages	7.45E-05	58
Rumen	Alfalfa hay	Negative	branching of cells	7.56E-05	110
Rumen	Alfalfa hay	Negative	cell viability of cortical neurons	7.70E-05	20
Rumen	Alfalfa hay	Negative	branching of neurites	7.74E-05	85
Rumen	Alfalfa hay	Negative	engulfment of cells	7.74E-05	120
Rumen	Alfalfa hay	Negative	formation of vascular lesion	7.74E-05	40
Rumen	Alfalfa hay	Negative	apoptosis of myeloma cell lines	8.00E-05	31
Rumen	Alfalfa hay	Negative	function of muscle	8.07E-05	104
Rumen	Alfalfa hay	Negative	congenital malformation of skeleton	8.63E-05	124
Rumen	Alfalfa hay	Negative	proliferation of endothelial cells	8.72E-05	94
Rumen	Alfalfa hay	Negative	necrosis of malignant tumor	8.77E-05	91
Rumen	Alfalfa hay	Negative	abnormal morphology of head	8.99E-05	231
Rumen	Alfalfa hay	Negative	transport of carboxylic acid	8.99E-05	41
Rumen	Alfalfa	Negative	synaptic transmission	9.22E-05	101

	hay				
Rumen	Alfalfa hay	Negative	aggregation of blood cells	9.67E-05	73
Rumen	Alfalfa hay	Negative	quantity of lymphatic system cells	9.72E-05	84
Rumen	Alfalfa hay	Negative	branching of neurons	9.80E-05	87
Rumen	Alfalfa hay	Negative	neurotransmission	9.99E-05	121
Rumen	Alfalfa hay	Negative	cell viability of connective tissue cells	1.00E-04	45
Rumen	Alfalfa hay	Negative	homo-oligomerization of protein	1.00E-04	45
Rumen	Alfalfa hay	Negative	development of phagocytes	1.03E-04	30
Rumen	Alfalfa hay	Negative	apoptosis of endothelial cells	1.05E-04	59
Rumen	Alfalfa hay	Negative	development of abdomen	1.05E-04	165
Rumen	Alfalfa hay	Negative	S phase	1.05E-04	80
Rumen	Alfalfa hay	Negative	Hypertrophy	1.06E-04	137
Rumen	Alfalfa hay	Negative	quantity of muscle	1.07E-04	44
Rumen	Alfalfa hay	Negative	migration of myeloid cells	1.07E-04	54
Rumen	Alfalfa hay	Negative	uptake of carbohydrate	1.08E-04	88
Rumen	Alfalfa hay	Negative	cell death of liver	1.10E-04	74
Rumen	Alfalfa hay	Negative	damage of epithelial tissue	1.10E-04	37
Rumen	Alfalfa hay	Negative	immune response of cells	1.11E-04	163
Rumen	Alfalfa hay	Negative	recruitment of phagocytes	1.13E-04	76
Rumen	Alfalfa hay	Negative	cell movement of muscle cells	1.15E-04	58
Rumen	Alfalfa hay	Negative	morphology of reproductive system	1.19E-04	153
Rumen	Alfalfa hay	Negative	cell death of cancer cells	1.20E-04	90
Rumen	Alfalfa	Negative	entry into S phase	1.21E-04	47

	hay				
Rumen	Alfalfa hay	Negative	cell viability of central nervous system cells	1.22E-04	39
Rumen	Alfalfa hay	Negative	abnormal morphology of reproductive system	1.24E-04	146
Rumen	Alfalfa hay	Negative	transport of synaptic vesicles	1.24E-04	27
Rumen	Alfalfa hay	Negative	morphogenesis of neurites	1.25E-04	122
Rumen	Alfalfa hay	Negative	assembly of protein-protein complex	1.25E-04	60
Rumen	Alfalfa hay	Negative	apoptosis of pheochromocytoma cell lines	1.26E-04	33
Rumen	Alfalfa hay	Negative	autophosphorylation of protein	1.28E-04	52
Rumen	Alfalfa hay	Negative	stress response of cells	1.30E-04	46
Rumen	Alfalfa hay	Negative	morphology of heart ventricle	1.30E-04	61
Rumen	Alfalfa hay	Negative	migration of hepatoma cell lines	1.30E-04	30
Rumen	Alfalfa hay	Negative	shape change of neurons	1.32E-04	88
Rumen	Alfalfa hay	Negative	replication of RNA virus	1.36E-04	145
Rumen	Alfalfa hay	Negative	quantity of muscle cells	1.39E-04	40
Rumen	Alfalfa hay	Negative	craniofacial abnormality	1.40E-04	105
Rumen	Alfalfa hay	Negative	astrocytosis of hippocampus	1.41E-04	9
Rumen	Alfalfa hay	Negative	ion homeostasis of cells	1.47E-04	158
Rumen	Alfalfa hay	Negative	permeability of cells	1.48E-04	35
Rumen	Alfalfa hay	Negative	cell movement of vascular smooth muscle cells	1.48E-04	39
Rumen	Alfalfa hay	Negative	abnormal morphology of endothelial tissue	1.49E-04	25
Rumen	Alfalfa hay	Negative	influx of cation	1.49E-04	61
Rumen	Alfalfa hay	Negative	degeneration of central nervous system	1.52E-04	44
Rumen	Alfalfa	Negative	necrosis of liver	1.54E-04	73

	hay				
Rumen	Alfalfa hay	Negative	sensory system development	1.56E-04	38
Rumen	Alfalfa hay	Negative	quantity of phagocytes	1.56E-04	123
Rumen	Alfalfa hay	Negative	recruitment of antigen presenting cells	1.57E-04	41
Rumen	Alfalfa hay	Negative	cell movement of neutrophils	1.63E-04	89
Rumen	Alfalfa hay	Negative	transmigration of cells	1.63E-04	51
Rumen	Alfalfa hay	Negative	growth of embryonic tissue	1.64E-04	83
Rumen	Alfalfa hay	Negative	apoptosis of cerebral cortex cells	1.64E-04	37
Rumen	Alfalfa hay	Negative	vascularization of body region	1.66E-04	45
Rumen	Alfalfa hay	Negative	synthesis of carbohydrate	1.69E-04	123
Rumen	Alfalfa hay	Negative	apoptosis of kidney cell lines	1.73E-04	73
Rumen	Alfalfa hay	Negative	concentration of phospholipid	1.75E-04	58
Rumen	Alfalfa hay	Negative	degeneration of brain	1.75E-04	42
Rumen	Alfalfa hay	Negative	shape change of tumor cell lines	1.78E-04	50
Rumen	Alfalfa hay	Negative	peripheral vascular disease	1.82E-04	111
Rumen	Alfalfa hay	Negative	function of heart	1.90E-04	71
Rumen	Alfalfa hay	Negative	binding of DNA	1.96E-04	148
Rumen	Alfalfa hay	Negative	hydrolysis of phospholipid	1.96E-04	43
Rumen	Alfalfa hay	Negative	benign neoplasia	2.02E-04	252
Rumen	Alfalfa hay	Negative	size of lesion	2.02E-04	77
Rumen	Alfalfa hay	Negative	morphogenesis of axons	2.15E-04	30
Rumen	Alfalfa hay	Negative	atrophy of muscle	2.15E-04	48
Rumen	Alfalfa	Negative	cell death of skin	2.15E-04	36

	hay				
Rumen	Alfalfa hay	Negative	formation of filopodia	2.15E-04	53
Rumen	Alfalfa hay	Negative	atherogenesis	2.16E-04	29
Rumen	Alfalfa hay	Negative	development of sensory organ	2.17E-04	159
Rumen	Alfalfa hay	Negative	hyperesthesia	2.17E-04	39
Rumen	Alfalfa hay	Negative	cell death of liver cells	2.24E-04	61
Rumen	Alfalfa hay	Negative	cell death of hepatoma cell lines	2.25E-04	54
Rumen	Alfalfa hay	Negative	length of cells	2.25E-04	35
Rumen	Alfalfa hay	Negative	apoptosis of hepatoma cell lines	2.29E-04	49
Rumen	Alfalfa hay	Negative	abnormal morphology of vascular endothelial cells	2.33E-04	18
Rumen	Alfalfa hay	Negative	infection of cells	2.38E-04	219
Rumen	Alfalfa hay	Negative	replication of virus	2.40E-04	158
Rumen	Alfalfa hay	Negative	proliferation of myeloid cells	2.45E-04	40
Rumen	Alfalfa hay	Negative	apoptosis of lymphoma cell lines	2.46E-04	56
Rumen	Alfalfa hay	Negative	cell death of embryonic cell lines	2.46E-04	73
Rumen	Alfalfa hay	Negative	apoptosis of cortical neurons	2.66E-04	31
Rumen	Alfalfa hay	Negative	morphology of vascular endothelial cells	2.72E-04	21
Rumen	Alfalfa hay	Negative	apoptosis of mononuclear leukocytes	2.72E-04	104
Rumen	Alfalfa hay	Negative	cell death of stem cells	2.72E-04	30
Rumen	Alfalfa hay	Negative	metabolism of phospholipid	2.73E-04	71
Rumen	Alfalfa hay	Negative	apoptosis of lymphocytes	2.86E-04	101
Rumen	Alfalfa hay	Negative	quantity of metal	3.02E-04	141
Rumen	Alfalfa	Negative	abnormal morphology of digestive system	3.03E-04	153

	hay				
Rumen	Alfalfa hay	Negative	cell death of sarcoma cell lines	3.03E-04	69
Rumen	Alfalfa hay	Negative	entry into interphase	3.18E-04	49
Rumen	Alfalfa hay	Negative	infection of embryonic cell lines	3.18E-04	82
Rumen	Alfalfa hay	Negative	infection of epithelial cell lines	3.18E-04	82
Rumen	Alfalfa hay	Negative	abnormal morphology of heart ventricle	3.18E-04	55
Rumen	Alfalfa hay	Negative	adhesion of tumor cell lines	3.51E-04	85
Rumen	Alfalfa hay	Negative	Edema	3.55E-04	100
Rumen	Alfalfa hay	Negative	cell movement of carcinoma cell lines	3.56E-04	63
Rumen	Alfalfa hay	Negative	apoptosis of microglia	3.56E-04	11
Rumen	Alfalfa hay	Negative	entrance of Ca2+	3.64E-04	28
Rumen	Alfalfa hay	Negative	contractility of heart	3.70E-04	55
Rumen	Alfalfa hay	Negative	proliferation of epithelial cells	3.71E-04	139
Rumen	Alfalfa hay	Negative	development of connective tissue	3.71E-04	107
Rumen	Alfalfa hay	Negative	synaptic transmission of nervous tissue	3.71E-04	37
Rumen	Alfalfa hay	Negative	migration of muscle cells	3.73E-04	52
Rumen	Alfalfa hay	Negative	connective or soft tissue tumor	3.78E-04	194
Rumen	Alfalfa hay	Negative	biosynthesis of polyunsaturated fatty acids	3.82E-04	68
Rumen	Alfalfa hay	Negative	cell viability of brain cells	3.84E-04	33
Rumen	Alfalfa hay	Negative	long-term potentiation	3.97E-04	73
Rumen	Alfalfa hay	Negative	activation of neuroglia	3.97E-04	43
Rumen	Alfalfa hay	Negative	neurodegeneration of cerebral cortex	4.15E-04	22
Rumen	Alfalfa	Negative	arrest in proliferation of cells	4.16E-04	65

	hay				
Rumen	Alfalfa hay	Negative	influx of inorganic cation	4.18E-04	59
Rumen	Alfalfa hay	Negative	abnormal morphology of embryonic tissue	4.33E-04	153
Rumen	Alfalfa hay	Negative	neurodegeneration of axons	4.39E-04	30
Rumen	Alfalfa hay	Negative	morphology of digestive system	4.39E-04	159
Rumen	Alfalfa hay	Negative	serous neoplasm	4.42E-04	169
Rumen	Alfalfa hay	Negative	branching of axons	4.51E-04	29
Rumen	Alfalfa hay	Negative	release of L-amino acid	4.51E-04	29
Rumen	Alfalfa hay	Negative	cognitive impairment	4.52E-04	96
Rumen	Alfalfa hay	Negative	abnormal morphology of abdomen	4.52E-04	237
Rumen	Alfalfa hay	Negative	formation of lung	4.57E-04	88
Rumen	Alfalfa hay	Negative	activation of central nervous system cells	4.60E-04	28
Rumen	Alfalfa hay	Negative	development of antigen presenting cells	4.64E-04	25
Rumen	Alfalfa hay	Negative	abnormal morphology of muscle	4.79E-04	92
Rumen	Alfalfa hay	Negative	cell spreading of tumor cell lines	4.80E-04	36
Rumen	Alfalfa hay	Negative	autosomal recessive disease	4.88E-04	248
Rumen	Alfalfa hay	Negative	function of antigen presenting cells	4.96E-04	73
Rumen	Alfalfa hay	Negative	release of neurotransmitter	5.01E-04	49
Rumen	Alfalfa hay	Negative	binding of filaments	5.11E-04	18
Rumen	Alfalfa hay	Negative	targeting of protein	5.12E-04	42
Rumen	Alfalfa hay	Negative	migration of vascular smooth muscle cells	5.12E-04	35
Rumen	Alfalfa hay	Negative	morphology of blood cells	5.16E-04	118
Rumen	Alfalfa	Negative	morphology of nervous system	5.20E-04	213

	hay				
Rumen	Alfalfa hay	Negative	apoptosis of dermal cells	5.21E-04	31
Rumen	Alfalfa hay	Negative	differentiation of connective tissue cells	5.23E-04	177
Rumen	Alfalfa hay	Negative	quantity of metal ion	5.45E-04	125
Rumen	Alfalfa hay	Negative	flux of cation	5.47E-04	89
Rumen	Alfalfa hay	Negative	migration of fibroblast cell lines	5.51E-04	43
Rumen	Alfalfa hay	Negative	transport of vesicles	5.56E-04	41
Rumen	Alfalfa hay	Negative	function of lymphocytes	5.72E-04	98
Rumen	Alfalfa hay	Negative	cell death of dermal cells	5.81E-04	33
Rumen	Alfalfa hay	Negative	infection of kidney cell lines	5.86E-04	83
Rumen	Alfalfa hay	Negative	function of synapse	5.92E-04	10
Rumen	Alfalfa hay	Negative	differentiation of T lymphocytes	6.05E-04	108
Rumen	Alfalfa hay	Negative	juvenile rheumatoid arthritis	6.06E-04	40
Rumen	Alfalfa hay	Negative	engulfment of leukocytes	6.06E-04	50
Rumen	Alfalfa hay	Negative	congenital anomaly of cardiovascular system	6.07E-04	68
Rumen	Alfalfa hay	Negative	differentiation of lymphocytes	6.09E-04	139
Rumen	Alfalfa hay	Negative	transport of carbohydrate	6.09E-04	59
Rumen	Alfalfa hay	Negative	apoptosis of skin	6.09E-04	32
Rumen	Alfalfa hay	Negative	invasion of tumor	6.11E-04	72
Rumen	Alfalfa hay	Negative	proliferation of myeloid progenitor cells	6.11E-04	17
Rumen	Alfalfa hay	Negative	sarcoma	6.14E-04	117
Rumen	Alfalfa hay	Negative	flux of ion	6.15E-04	93
Rumen	Alfalfa	Negative	formation of muscle cells	6.24E-04	55

	hay				
Rumen	Alfalfa hay	Negative	cell death of leukemia cell lines	6.24E-04	92
Rumen	Alfalfa hay	Negative	atherosclerotic lesion	6.31E-04	48
Rumen	Rice straw	Positive	proliferation of cells	1.63E-34	1224
Rumen	Rice straw	Positive	organismal death	1.06E-33	850
Rumen	Rice straw	Positive	morbidity or mortality	3.21E-33	857
Rumen	Rice straw	Positive	malignant solid tumor	5.86E-32	3030
Rumen	Rice straw	Positive	cancer	1.60E-31	3060
Rumen	Rice straw	Positive	cell death	4.08E-23	1068
Rumen	Rice straw	Positive	morphology of cells	8.90E-22	668
Rumen	Rice straw	Positive	necrosis	1.49E-21	846
Rumen	Rice straw	Positive	apoptosis	3.35E-21	864
Rumen	Rice straw	Positive	tumorigenesis of tissue	6.28E-21	2555
Rumen	Rice straw	Positive	epithelial cancer	1.30E-20	2494
Rumen	Rice straw	Positive	neoplasia of epithelial tissue	2.04E-20	2514
Rumen	Rice straw	Positive	expression of RNA	2.04E-20	719
Rumen	Rice straw	Positive	transcription	3.63E-20	674
Rumen	Rice straw	Positive	abdominal neoplasm	3.57E-19	2489
Rumen	Rice straw	Positive	transport of molecule	5.24E-19	557
Rumen	Rice straw	Positive	organization of cytoplasm	6.52E-19	533
Rumen	Rice straw	Positive	transcription of RNA	1.79E-18	626
Rumen	Rice straw	Positive	abdominal cancer	3.66E-18	2456
Rumen	Rice	Positive	cell movement	2.03E-17	698

	straw				
Rumen	Rice straw	Positive	migration of cells	4.88E-16	627
Rumen	Rice straw	Positive	cell death of tumor cell lines	8.04E-16	517
Rumen	Rice straw	Positive	organization of organelle	1.07E-15	214
Rumen	Rice straw	Positive	cell death of connective tissue cells	8.53E-15	232
Rumen	Rice straw	Positive	differentiation of cells	2.58E-14	716
Rumen	Rice straw	Positive	vasculogenesis	2.84E-14	273
Rumen	Rice straw	Positive	organization of cytoskeleton	3.14E-14	471
Rumen	Rice straw	Positive	apoptosis of tumor cell lines	3.14E-14	417
Rumen	Rice straw	Positive	angiogenesis	4.42E-14	325
Rumen	Rice straw	Positive	cell survival	5.73E-14	469
Rumen	Rice straw	Positive	quantity of cells	9.40E-14	576
Rumen	Rice straw	Positive	digestive system cancer	9.40E-14	2120
Rumen	Rice straw	Positive	cell viability	1.39E-13	440
Rumen	Rice straw	Positive	digestive organ tumor	1.85E-13	2136
Rumen	Rice straw	Positive	Movement Disorders	3.50E-13	367
Rumen	Rice straw	Positive	transcription of DNA	5.97E-13	505
Rumen	Rice straw	Positive	growth of organism	2.58E-12	296
Rumen	Rice straw	Positive	synthesis of lipid	5.90E-12	257
Rumen	Rice straw	Positive	Viral Infection	7.88E-12	521
Rumen	Rice straw	Positive	microtubule dynamics	9.44E-12	397
Rumen	Rice straw	Positive	invasion of cells	2.53E-11	302
Rumen	Rice	Positive	cellular homeostasis	2.77E-11	492

	straw				
Rumen	Rice straw	Positive	abnormal morphology of cells	3.15E-11	418
Rumen	Rice straw	Positive	growth of connective tissue	3.60E-11	232
Rumen	Rice straw	Positive	development of body trunk	4.55E-11	374
Rumen	Rice straw	Positive	congenital anomaly of musculoskeletal system	4.55E-11	260
Rumen	Rice straw	Positive	proliferation of tumor cell lines	4.85E-11	498
Rumen	Rice straw	Positive	proliferation of connective tissue cells	5.95E-11	215
Rumen	Rice straw	Positive	interphase	9.36E-11	234
Rumen	Rice straw	Positive	morphology of cardiovascular system	1.34E-10	222
Rumen	Rice straw	Positive	transactivation	1.34E-10	209
Rumen	Rice straw	Positive	size of body	2.06E-10	292
Rumen	Rice straw	Positive	cell death of fibroblasts	2.18E-10	107
Rumen	Rice straw	Positive	morphology of body cavity	2.19E-10	393
Rumen	Rice straw	Positive	growth of embryo	2.45E-10	176
Rumen	Rice straw	Positive	invasion of tumor cell lines	2.46E-10	235
Rumen	Rice straw	Positive	function of leukocytes	4.83E-10	194
Rumen	Rice straw	Positive	cell cycle progression	5.53E-10	348
Rumen	Rice straw	Positive	phosphorylation of protein	5.53E-10	255
Rumen	Rice straw	Positive	apoptosis of connective tissue cells	6.39E-10	116
Rumen	Rice straw	Positive	protein kinase cascade	1.09E-09	156
Rumen	Rice straw	Positive	perinatal death	1.40E-09	210
Rumen	Rice straw	Positive	formation of cytoskeleton	1.49E-09	141
Rumen	Rice	Positive	activation of DNA endogenous promoter	1.50E-09	393

	straw				
Rumen	Rice straw	Positive	quantity of leukocytes	1.61E-09	307
Rumen	Rice straw	Positive	function of blood cells	1.89E-09	207
Rumen	Rice straw	Positive	cell movement of tumor cell lines	4.02E-09	286
Rumen	Rice straw	Positive	formation of cellular protrusions	4.25E-09	301
Rumen	Rice straw	Positive	development of vasculature	4.25E-09	153
Rumen	Rice straw	Positive	migration of tumor cell lines	4.98E-09	242
Rumen	Rice straw	Positive	congenital malformation of skeleton	5.29E-09	154
Rumen	Rice straw	Positive	quantity of blood cells	5.29E-09	337
Rumen	Rice straw	Positive	concentration of lipid	5.84E-09	273
Rumen	Rice straw	Positive	growth of epithelial tissue	7.80E-09	233
Rumen	Rice straw	Positive	craniofacial abnormality	8.22E-09	132
Rumen	Rice straw	Positive	uptake of carbohydrate	8.31E-09	111
Rumen	Rice straw	Positive	transactivation of RNA	8.31E-09	191
Rumen	Rice straw	Positive	morphology of nervous system	8.41E-09	264
Rumen	Rice straw	Positive	metabolism of membrane lipid derivative	8.64E-09	142
Rumen	Rice straw	Positive	abnormal morphology of thoracic cavity	9.39E-09	204
Rumen	Rice straw	Positive	abnormal morphology of nervous system	9.68E-09	246
Rumen	Rice straw	Positive	secretory pathway	1.06E-08	80
Rumen	Rice straw	Positive	learning	1.21E-08	151
Rumen	Rice straw	Positive	necrosis of epithelial tissue	1.31E-08	204
Rumen	Rice straw	Positive	cell movement of fibroblasts	1.47E-08	75
Rumen	Rice	Positive	quantity of connective tissue	1.53E-08	208

	straw				
Rumen	Rice straw	Positive	cognition	1.56E-08	162
Rumen	Rice straw	Positive	abnormal morphology of body cavity	1.65E-08	367
Rumen	Rice straw	Positive	cell death of cervical cancer cell lines	1.94E-08	129
Rumen	Rice straw	Positive	abdominal adenocarcinoma	2.26E-08	1670
Rumen	Rice straw	Positive	proliferation of hematopoietic progenitor cells	2.50E-08	94
Rumen	Rice straw	Positive	mitosis	2.90E-08	174
Rumen	Rice straw	Positive	ubiquitination	2.90E-08	124
Rumen	Rice straw	Positive	apoptosis of fibroblast cell lines	3.37E-08	121
Rumen	Rice straw	Positive	quantity of lymphocytes	3.48E-08	238
Rumen	Rice straw	Positive	degeneration of cells	3.51E-08	126
Rumen	Rice straw	Positive	uptake of monosaccharide	3.51E-08	102
Rumen	Rice straw	Positive	development of cytoplasm	3.51E-08	163
Rumen	Rice straw	Positive	Growth Failure	3.51E-08	205
Rumen	Rice straw	Positive	ubiquitination of protein	3.55E-08	122
Rumen	Rice straw	Positive	neuromuscular disease	3.55E-08	290
Rumen	Rice straw	Positive	proliferation of neuronal cells	3.66E-08	198
Rumen	Rice straw	Positive	fibrogenesis	3.74E-08	147
Rumen	Rice straw	Positive	abnormal morphology of cardiovascular system	4.56E-08	195
Rumen	Rice straw	Positive	accumulation of lipid	4.94E-08	111
Rumen	Rice straw	Positive	adenocarcinoma	5.49E-08	1823
Rumen	Rice straw	Positive	formation of cells	5.80E-08	318
Rumen	Rice	Positive	quantity of mononuclear leukocytes	6.24E-08	245

	straw				
Rumen	Rice straw	Positive	morphology of heart	7.91E-08	144
Rumen	Rice straw	Positive	neurological signs	9.22E-08	219
Rumen	Rice straw	Positive	cell death of epithelial cells	9.32E-08	172
Rumen	Rice straw	Positive	cell viability of tumor cell lines	9.39E-08	257
Rumen	Rice straw	Positive	vascularization	9.72E-08	90
Rumen	Rice straw	Positive	degeneration of nervous system	1.19E-07	106
Rumen	Rice straw	Positive	development of cardiovascular tissue	1.23E-07	132
Rumen	Rice straw	Positive	cellular degradation	1.84E-07	95
Rumen	Rice straw	Positive	morphology of head	1.90E-07	284
Rumen	Rice straw	Positive	formation of filaments	2.02E-07	140
Rumen	Rice straw	Positive	S phase	2.03E-07	97
Rumen	Rice straw	Positive	autosomal recessive disease	2.06E-07	297
Rumen	Rice straw	Positive	degeneration of neurons	2.08E-07	90
Rumen	Rice straw	Positive	proliferation of hematopoietic cells	2.13E-07	95
Rumen	Rice straw	Positive	Huntington's Disease	2.25E-07	194
Rumen	Rice straw	Positive	Organ Degeneration	2.60E-07	169
Rumen	Rice straw	Positive	dyskinesia	3.28E-07	206
Rumen	Rice straw	Positive	binding of DNA	3.28E-07	177
Rumen	Rice straw	Positive	cell movement of connective tissue cells	3.51E-07	84
Rumen	Rice straw	Positive	cell death of epithelial cell lines	3.65E-07	103
Rumen	Rice straw	Positive	disorder of basal ganglia	4.33E-07	250
Rumen	Rice	Positive	development of blood cells	4.60E-07	216

	straw				
Rumen	Rice straw	Positive	seizures	4.66E-07	139
Rumen	Rice straw	Positive	development of epithelial tissue	4.69E-07	175
Rumen	Rice straw	Positive	abnormal morphology of head	4.80E-07	269
Rumen	Rice straw	Positive	apoptosis of fibroblasts	5.30E-07	82
Rumen	Rice straw	Positive	migration of connective tissue cells	5.95E-07	70
Rumen	Rice straw	Positive	exocytosis	5.95E-07	73
Rumen	Rice straw	Positive	synthesis of DNA	6.24E-07	144
Rumen	Rice straw	Positive	proliferation of fibroblasts	6.97E-07	124
Rumen	Rice straw	Positive	congenital encephalopathy	7.26E-07	108
Rumen	Rice straw	Positive	abdominal carcinoma	7.55E-07	1676
Rumen	Rice straw	Positive	genital tumor	8.13E-07	1154
Rumen	Rice straw	Positive	Neurodegeneration	8.81E-07	110
Rumen	Rice straw	Positive	congenital malformation of brain	8.84E-07	107
Rumen	Rice straw	Positive	uptake of D-hexose	8.91E-07	83
Rumen	Rice straw	Positive	replication of virus	9.48E-07	187
Rumen	Rice straw	Positive	female genital neoplasm	9.74E-07	1022
Rumen	Rice straw	Positive	development of leukocytes	1.06E-06	195
Rumen	Rice straw	Positive	uptake of D-glucose	1.09E-06	82
Rumen	Rice straw	Positive	cell death of fibroblast cell lines	1.11E-06	146
Rumen	Rice straw	Positive	growth of embryonic tissue	1.11E-06	99
Rumen	Rice straw	Positive	replication of RNA virus	1.11E-06	170
Rumen	Rice	Positive	seizure disorder	1.12E-06	159

	straw				
Rumen	Rice straw	Positive	apoptosis of cervical cancer cell lines	1.16E-06	101
Rumen	Rice straw	Positive	development of endothelial tissue	1.24E-06	127
Rumen	Rice straw	Positive	cell movement of endothelial cells	1.40E-06	123
Rumen	Rice straw	Positive	endothelial cell development	1.40E-06	123
Rumen	Rice straw	Positive	development of connective tissue	1.74E-06	128
Rumen	Rice straw	Positive	neuritogenesis	1.95E-06	192
Rumen	Rice straw	Positive	vascularization of body region	2.04E-06	54
Rumen	Rice straw	Positive	gastrointestinal tract cancer	2.04E-06	1422
Rumen	Rice straw	Positive	cell transformation	2.10E-06	155
Rumen	Rice straw	Positive	growth of lesion	2.15E-06	255
Rumen	Rice straw	Positive	cell movement of leukocytes	2.16E-06	246
Rumen	Rice straw	Positive	differentiation of epithelial tissue	2.22E-06	124
Rumen	Rice straw	Positive	hepatocellular carcinoma	2.24E-06	1188
Rumen	Rice straw	Positive	development of lymphatic system	2.35E-06	123
Rumen	Rice straw	Positive	gastrointestinal carcinoma	2.38E-06	1280
Rumen	Rice straw	Positive	morphology of respiratory system	2.43E-06	109
Rumen	Rice straw	Positive	formation of skin	2.48E-06	126
Rumen	Rice straw	Positive	growth of tumor	2.66E-06	254
Rumen	Rice straw	Positive	migration of fibroblasts	2.70E-06	56
Rumen	Rice straw	Positive	cell spreading	2.84E-06	98
Rumen	Rice straw	Positive	proliferation of epithelial cells	2.84E-06	164
Rumen	Rice	Positive	development of abdomen	2.88E-06	190

	straw				
Rumen	Rice straw	Positive	morphology of bone	2.99E-06	151
Rumen	Rice straw	Positive	proliferation of blood cells	3.03E-06	267
Rumen	Rice straw	Positive	metabolism of carbohydrate	3.34E-06	202
Rumen	Rice straw	Positive	abnormal morphology of respiratory system	3.57E-06	107
Rumen	Rice straw	Positive	cell movement of blood cells	3.66E-06	276
Rumen	Rice straw	Positive	differentiation of tumor cell lines	3.75E-06	129
Rumen	Rice straw	Positive	midline defect	4.02E-06	94
Rumen	Rice straw	Positive	migration of blood cells	4.24E-06	275
Rumen	Rice straw	Positive	growth of muscle tissue	4.27E-06	127
Rumen	Rice straw	Positive	formation of actin filaments	4.30E-06	106
Rumen	Rice straw	Positive	proliferation of endothelial cells	4.38E-06	108
Rumen	Rice straw	Positive	abnormal morphology of heart	4.41E-06	130
Rumen	Rice straw	Positive	formation of plasma membrane projections	4.47E-06	194
Rumen	Rice straw	Positive	transport of protein	4.49E-06	105
Rumen	Rice straw	Positive	arthritis	4.53E-06	293
Rumen	Rice straw	Positive	formation of lymphatic system component	4.60E-06	102
Rumen	Rice straw	Positive	quantity of glycosphingolipid	4.60E-06	41
Rumen	Rice straw	Positive	hepatobiliary system cancer	4.67E-06	1226
Rumen	Rice straw	Positive	migration of endothelial cells	4.70E-06	113
Rumen	Rice straw	Positive	proliferation of muscle cells	4.97E-06	126
Rumen	Rice straw	Positive	concentration of phospholipid	5.04E-06	68
Rumen	Rice	Positive	leukocyte migration	5.04E-06	274

	straw				
Rumen	Rice straw	Positive	development of lymphatic system component	5.12E-06	107
Rumen	Rice straw	Positive	arthropathy	5.48E-06	297
Rumen	Rice straw	Positive	morphogenesis of neurons	5.98E-06	142
Rumen	Rice straw	Positive	synthesis of carbohydrate	5.98E-06	142
Rumen	Rice straw	Positive	behavior	6.19E-06	307
Rumen	Rice straw	Positive	formation of lymphoid organ	6.32E-06	93
Rumen	Rice straw	Positive	T cell development	7.01E-06	165
Rumen	Rice straw	Positive	quantity of metal ion	7.30E-06	147
Rumen	Rice straw	Positive	abnormal morphology of bone	7.54E-06	145
Rumen	Rice straw	Positive	cell movement of myeloid cells	7.59E-06	176
Rumen	Rice straw	Positive	survival of organism	7.76E-06	224
Rumen	Rice straw	Positive	liver cancer	7.90E-06	1218
Rumen	Rice straw	Positive	formation of actin stress fibers	7.97E-06	85
Rumen	Rice straw	Positive	differentiation of bone cells	8.00E-06	136
Rumen	Rice straw	Positive	proliferation of smooth muscle cells	8.04E-06	99
Rumen	Rice straw	Positive	cell death of blood cells	8.05E-06	221
Rumen	Rice straw	Positive	cell death of T lymphocytes	8.08E-06	108
Rumen	Rice straw	Positive	Gastrointestinal Tract Cancer and Tumors	8.13E-06	1436
Rumen	Rice straw	Positive	abnormal morphology of abdomen	8.13E-06	274
Rumen	Rice straw	Positive	differentiation of bone	8.16E-06	137
Rumen	Rice straw	Positive	differentiation of connective tissue cells	8.64E-06	206
Rumen	Rice	Positive	quantity of carbohydrate	9.16E-06	169

	straw				
Rumen	Rice straw	Positive	synthesis of nitric oxide	9.35E-06	96
Rumen	Rice straw	Positive	quantity of metal	9.50E-06	163
Rumen	Rice straw	Positive	oxidation of palmitic acid	9.65E-06	24
Rumen	Rice straw	Positive	quantity of B lymphocytes	9.65E-06	117
Rumen	Rice straw	Positive	morphogenesis of neurites	9.81E-06	139
Rumen	Rice straw	Positive	growth of neurites	1.02E-05	152
Rumen	Rice straw	Positive	paraproteinemia	1.03E-05	114
Rumen	Rice straw	Positive	liver tumor	1.05E-05	1224
Rumen	Rice straw	Positive	development of neurons	1.08E-05	246
Rumen	Rice straw	Positive	differentiation of epithelial cells	1.08E-05	107
Rumen	Rice straw	Positive	attachment of cells	1.10E-05	52
Rumen	Rice straw	Positive	sprouting	1.10E-05	129
Rumen	Rice straw	Positive	epileptic seizure	1.12E-05	62
Rumen	Rice straw	Positive	morphology of central nervous system	1.13E-05	167
Rumen	Rice straw	Positive	arrest in interphase	1.15E-05	138
Rumen	Rice straw	Positive	plasticity of synapse	1.16E-05	47
Rumen	Rice straw	Positive	development of digestive system	1.16E-05	128
Rumen	Rice straw	Positive	small GTPase mediated signal transduction	1.22E-05	67
Rumen	Rice straw	Positive	peripheral vascular disease	1.24E-05	127
Rumen	Rice straw	Positive	Edema	1.28E-05	116
Rumen	Rice straw	Positive	secretion of molecule	1.29E-05	178
Rumen	Rice	Positive	export of molecule	1.34E-05	101

	straw				
Rumen	Rice straw	Positive	transport of carbohydrate	1.34E-05	70
Rumen	Rice straw	Positive	breast or colorectal cancer	1.36E-05	1387
Rumen	Rice straw	Positive	tubulation of endothelial cells	1.40E-05	46
Rumen	Rice straw	Positive	immune response of cells	1.42E-05	184
Rumen	Rice straw	Positive	quantity of sphingolipid	1.42E-05	42
Rumen	Rice straw	Positive	colony formation of cells	1.51E-05	160
Rumen	Rice straw	Positive	plasma cell dyscrasia	1.53E-05	113
Rumen	Rice straw	Positive	concentration of fatty acid	1.54E-05	96
Rumen	Rice straw	Positive	apoptosis of epithelial cell lines	1.54E-05	79
Rumen	Rice straw	Positive	homeostasis of blood cells	1.54E-05	179
Rumen	Rice straw	Positive	long-term potentiation	1.57E-05	85
Rumen	Rice straw	Positive	T cell homeostasis	1.59E-05	167
Rumen	Rice straw	Positive	development of lymphocytes	1.65E-05	178
Rumen	Rice straw	Positive	Rheumatic Disease	1.66E-05	329
Rumen	Rice straw	Positive	branching of cells	1.79E-05	123
Rumen	Rice straw	Positive	differentiation of embryonic tissue	1.80E-05	88
Rumen	Rice straw	Positive	development of mononuclear leukocytes	1.83E-05	179
Rumen	Rice straw	Positive	abnormal morphology of central nervous system	1.83E-05	157
Rumen	Rice straw	Positive	concentration of cholesterol	1.91E-05	99
Rumen	Rice straw	Positive	repair of DNA	1.91E-05	99
Rumen	Rice straw	Positive	homeostasis of leukocytes	1.96E-05	178
Rumen	Rice	Positive	function of myeloid cells	1.99E-05	83

	straw				
Rumen	Rice straw	Positive	stage 3 cancer	1.99E-05	51
Rumen	Rice straw	Positive	differentiation of connective tissue	1.99E-05	229
Rumen	Rice straw	Positive	function of lymphocytes	1.99E-05	114
Rumen	Rice straw	Positive	gonadal tumor	1.99E-05	255
Rumen	Rice straw	Positive	Lymphocyte homeostasis	2.00E-05	175
Rumen	Rice straw	Positive	benign neoplasia	2.08E-05	285
Rumen	Rice straw	Positive	outgrowth of neurites	2.12E-05	133
Rumen	Rice straw	Positive	development of reproductive system	2.16E-05	231
Rumen	Rice straw	Positive	metabolism of nucleic acid component or derivative	2.25E-05	167
Rumen	Rice straw	Positive	proliferation of embryonic cells	2.25E-05	86
Rumen	Rice straw	Positive	binding of protein binding site	2.26E-05	98
Rumen	Rice straw	Positive	infection by RNA virus	2.26E-05	268
Rumen	Rice straw	Positive	apoptosis of blood cells	2.31E-05	160
Rumen	Rice straw	Positive	abnormal morphology of epithelial tissue	2.43E-05	137
Rumen	Rice straw	Positive	cell death of tumor	2.46E-05	126
Rumen	Rice straw	Positive	multiple congenital anomalies	2.51E-05	138
Rumen	Rice straw	Positive	cell death of immune cells	2.52E-05	208
Rumen	Rice straw	Positive	cell death of lymphocytes	2.55E-05	127
Rumen	Rice straw	Positive	neonatal death	2.64E-05	143
Rumen	Rice straw	Positive	homing of cells	2.70E-05	178
Rumen	Rice straw	Positive	tumorigenesis of genital organ	2.72E-05	1101
Rumen	Rice	Positive	cell death of thymocytes	3.00E-05	55

	straw				
Rumen	Rice straw	Positive	metabolism of nucleoside triphosphate	3.01E-05	52
Rumen	Rice straw	Positive	development of genitourinary system	3.15E-05	273
Rumen	Rice straw	Positive	polyarticular juvenile rheumatoid arthritis	3.18E-05	36
Rumen	Rice straw	Positive	cell death of mononuclear leukocytes	3.18E-05	130
Rumen	Rice straw	Positive	differentiation of embryonic cells	3.18E-05	79
Rumen	Rice straw	Positive	metabolism of protein	3.29E-05	297
Rumen	Rice straw	Positive	neovascularization	3.42E-05	59
Rumen	Rice straw	Positive	recruitment of antigen presenting cells	3.42E-05	46
Rumen	Rice straw	Positive	fatty acid metabolism	3.42E-05	190
Rumen	Rice straw	Positive	peripheral arterial disease	3.42E-05	74
Rumen	Rice straw	Positive	synthesis of steroid	3.48E-05	88
Rumen	Rice straw	Positive	tubulation of cells	3.64E-05	54
Rumen	Rice straw	Positive	colon cancer	3.70E-05	1042
Rumen	Rice straw	Positive	colony formation	3.70E-05	171
Rumen	Rice straw	Positive	organization of filaments	3.71E-05	72
Rumen	Rice straw	Positive	proliferation of immune cells	3.89E-05	245
Rumen	Rice straw	Positive	Encephalitis	3.96E-05	99
Rumen	Rice straw	Positive	oxidation of long chain fatty acid	3.97E-05	29
Rumen	Rice straw	Positive	intestinal tumor	4.06E-05	1218
Rumen	Rice straw	Positive	neuronal cell death	4.08E-05	213
Rumen	Rice straw	Positive	blood protein disorder	4.08E-05	123
Rumen	Rice	Positive	tumorigenesis of reproductive tract	4.09E-05	965

	straw				
Rumen	Rice straw	Positive	cell death of neuroblastoma cell lines	4.35E-05	68
Rumen	Rice straw	Positive	cartilage development	4.42E-05	53
Rumen	Rice straw	Positive	inflammation of central nervous system	4.43E-05	101
Rumen	Rice straw	Positive	intestinal cancer	4.48E-05	1200
Rumen	Rice straw	Positive	quantity of T lymphocytes	4.68E-05	170
Rumen	Rice straw	Positive	concentration of sterol	4.69E-05	102
Rumen	Rice straw	Positive	tumorigenesis of gonad	4.72E-05	249
Rumen	Rice straw	Positive	cell movement of phagocytes	4.96E-05	174
Rumen	Rice straw	Positive	cell movement of muscle cells	5.00E-05	64
Rumen	Rice straw	Positive	cell death of breast cancer cell lines	5.01E-05	110
Rumen	Rice straw	Positive	proliferation of embryonic cell lines	5.03E-05	57
Rumen	Rice straw	Positive	colorectal neoplasia	5.12E-05	1213
Rumen	Rice straw	Positive	neoplasia of colon	5.12E-05	1043
Rumen	Rice straw	Positive	necrosis of tumor	5.18E-05	124
Rumen	Rice straw	Positive	apoptosis of neuroblastoma cell lines	5.19E-05	46
Rumen	Rice straw	Positive	colorectal cancer	5.29E-05	1197
Rumen	Rice straw	Positive	homing	5.33E-05	181
Rumen	Rice straw	Positive	memory	5.38E-05	87
Rumen	Rice straw	Positive	tumorigenesis of intestine	5.40E-05	1045
Rumen	Rice straw	Positive	quantity of Ca2+	5.47E-05	132
Rumen	Rice straw	Positive	branching of neurites	5.55E-05	93
Rumen	Rice	Positive	infection by Retroviridae	5.55E-05	223

	straw				
Rumen	Rice straw	Positive	biosynthesis of nucleoside triphosphate	5.58E-05	41
Rumen	Rice straw	Positive	movement of vascular endothelial cells	5.78E-05	64
Rumen	Rice straw	Positive	epilepsy	5.82E-05	102
Rumen	Rice straw	Positive	colorectal carcinoma	6.06E-05	1040
Rumen	Rice straw	Positive	activation of enzyme	6.07E-05	126
Rumen	Rice straw	Positive	apoptosis of embryonic cells	6.07E-05	38
Rumen	Rice straw	Positive	colon tumor	6.07E-05	1045
Rumen	Rice straw	Positive	abnormal morphology of embryonic tissue	6.07E-05	173
Rumen	Rice straw	Positive	chemotaxis of cells	6.07E-05	167
Rumen	Rice straw	Positive	concentration of acylglycerol	6.07E-05	110
Rumen	Rice straw	Positive	serous neoplasm	6.23E-05	191
Rumen	Rice straw	Positive	metabolism of triacylglycerol	6.40E-05	39
Rumen	Rice straw	Positive	infection of cells	6.41E-05	245
Rumen	Rice straw	Positive	activation of cells	6.53E-05	296
Rumen	Rice straw	Positive	apoptosis of epithelial cells	7.07E-05	95
Rumen	Rice straw	Positive	outgrowth of cells	7.25E-05	139
Rumen	Rice straw	Positive	cell death of kidney cells	7.26E-05	114
Rumen	Rice straw	Positive	ion homeostasis of cells	7.35E-05	175
Rumen	Rice straw	Positive	development of gastrointestinal tract	7.39E-05	88
Rumen	Rice straw	Positive	proliferation of central nervous system cells	7.47E-05	54
Rumen	Rice straw	Positive	concentration of Ca2+	7.90E-05	47
Rumen	Rice	Positive	Ovarian Cancer and Tumors	7.90E-05	233

	straw				
Rumen	Rice straw	Positive	cell movement of macrophages	7.90E-05	89
Rumen	Rice straw	Positive	morphology of skeleton	7.95E-05	93
Rumen	Rice straw	Positive	biosynthesis of purine ribonucleotide	8.17E-05	39
Rumen	Rice straw	Positive	cell death of kidney cell lines	8.27E-05	100
Rumen	Rice straw	Positive	size of bone	8.46E-05	92
Rumen	Rice straw	Positive	differentiation of smooth muscle	8.63E-05	28
Rumen	Rice straw	Positive	recruitment of phagocytes	8.63E-05	83
Rumen	Rice straw	Positive	morphology of vessel	8.69E-05	101
Rumen	Rice straw	Positive	urogenital cancer	8.78E-05	1239
Rumen	Rice straw	Positive	genital tract cancer	8.93E-05	1085
Rumen	Rice straw	Positive	apoptosis of T lymphocytes	8.93E-05	93
Rumen	Rice straw	Positive	epithelial-mesenchymal transition	9.27E-05	71
Rumen	Rice straw	Positive	cell movement of neutrophils	9.34E-05	98
Rumen	Rice straw	Positive	entry into S phase	9.34E-05	51
Rumen	Rice straw	Positive	encephalomyelitis	9.34E-05	86
Rumen	Rice straw	Positive	infection by lentivirus	9.47E-05	220
Rumen	Rice straw	Positive	cognitive impairment	9.55E-05	108
Rumen	Rice straw	Positive	chemotaxis	9.55E-05	171
Rumen	Rice straw	Positive	maturation of cells	9.78E-05	145
Rumen	Rice straw	Positive	cell movement of smooth muscle cells	9.84E-05	57
Rumen	Rice straw	Positive	cell death of tumor cells	9.85E-05	120
Rumen	Rice	Positive	morphology of reproductive system	9.96E-05	168

	straw				
Rumen	Rice straw	Positive	female genital tract cancer	1.00E-04	954
Rumen	Rice straw	Positive	invasion of prostate cancer cell lines	1.02E-04	39
Rumen	Rice straw	Positive	formation of focal adhesions	1.03E-04	55
Rumen	Rice straw	Positive	cell death of hematopoietic progenitor cells	1.03E-04	77
Rumen	Rice straw	Positive	recruitment of macrophages	1.05E-04	40
Rumen	Rice straw	Positive	differentiation of osteoblasts	1.05E-04	92
Rumen	Rice straw	Positive	abnormal morphology of skeleton	1.05E-04	90
Rumen	Rice straw	Positive	apoptosis of leukocytes	1.05E-04	145
Rumen	Rice straw	Positive	spatial learning	1.06E-04	64
Rumen	Rice straw	Positive	HIV infection	1.06E-04	219
Rumen	Rice straw	Positive	MAPKKK cascade	1.10E-04	70
Rumen	Rice straw	Positive	cell death of embryonic cells	1.12E-04	43
Rumen	Rice straw	Positive	proliferation of myeloid cells	1.13E-04	44
Rumen	Rice straw	Positive	homeostasis of ion	1.13E-04	80
Rumen	Rice straw	Positive	reorganization of cytoskeleton	1.13E-04	66
Rumen	Rice straw	Positive	function of cardiovascular system	1.15E-04	119
Rumen	Rice straw	Positive	transport of monosaccharide	1.18E-04	56
Rumen	Rice straw	Positive	engulfment of cells	1.18E-04	130
Rumen	Rice straw	Positive	Waldenstrom's macroglobulinemia	1.18E-04	65
Rumen	Rice straw	Positive	synthesis of nucleotide	1.18E-04	117
Rumen	Rice straw	Positive	differentiation of muscle	1.20E-04	99
Rumen	Rice	Positive	size of embryo	1.22E-04	115

	straw				
Rumen	Rice straw	Positive	synthesis of terpenoid	1.24E-04	93
Rumen	Rice straw	Positive	morphology of brain	1.30E-04	147
Rumen	Rice straw	Positive	chronic myeloid leukemia	1.30E-04	40
Rumen	Rice straw	Positive	differentiation of dermal cells	1.31E-04	58
Rumen	Rice straw	Positive	growth of lymphatic system component	1.31E-04	66
Rumen	Rice straw	Positive	metabolism of nucleotide	1.32E-04	139
Rumen	Rice straw	Positive	apoptosis of mononuclear leukocytes	1.35E-04	115
Rumen	Rice straw	Positive	organization of cells	1.38E-04	32
Rumen	Rice straw	Positive	cell death of pheochromocytoma cell lines	1.38E-04	44
Rumen	Rice straw	Positive	demyelination	1.38E-04	44
Rumen	Rice straw	Positive	experimental autoimmune encephalomyelitis	1.40E-04	85
Rumen	Rice straw	Positive	mass of organism	1.42E-04	108
Rumen	Rice straw	Positive	necrosis of kidney	1.44E-04	117
Rumen	Rice straw	Positive	development of head	1.53E-04	294
Rumen	Rice straw	Positive	quantity of connective tissue cells	1.55E-04	79
Rumen	Rice straw	Positive	oral cancer	1.61E-04	76
Rumen	Rice straw	Positive	neoplasia of cells	1.62E-04	140
Rumen	Rice straw	Positive	morphology of lymphatic system component	1.64E-04	132
Rumen	Rice straw	Positive	organization of cellular membrane	1.66E-04	48
Rumen	Rice straw	Positive	cell death of embryonic cell lines	1.67E-04	80
Rumen	Rice straw	Positive	I-kappaB kinase/NF-kappaB cascade	1.68E-04	60
Rumen	Rice	Positive	cell death of lymphoid organ	1.68E-04	60

	straw				
Rumen	Rice straw	Positive	vascularization of eye	1.68E-04	43
Rumen	Rice straw	Positive	exencephaly	1.68E-04	45
Rumen	Rice straw	Positive	function of T lymphocytes	1.68E-04	86
Rumen	Rice straw	Positive	proliferation of fibroblast cell lines	1.69E-04	134
Rumen	Rice straw	Positive	respiratory system development	1.69E-04	110
Rumen	Rice straw	Positive	cell death of hematopoietic cells	1.75E-04	79
Rumen	Rice straw	Positive	accumulation of steroid	1.78E-04	32
Rumen	Rice straw	Positive	differentiation of keratinocytes	1.78E-04	53
Rumen	Rice straw	Positive	development of body axis	1.83E-04	313
Rumen	Rice straw	Positive	mass of connective tissue	1.84E-04	68
Rumen	Rice straw	Positive	differentiation of epidermal cells	1.87E-04	57
Rumen	Rice straw	Positive	growth of skin	1.87E-04	73
Rumen	Rice straw	Positive	apoptosis of lymphocytes	1.95E-04	111
Rumen	Rice straw	Positive	migration of sarcoma cell lines	1.96E-04	30
Rumen	Rice straw	Positive	morphology of limb	1.96E-04	77
Rumen	Rice straw	Positive	dysmyelination	1.98E-04	48
Rumen	Rice straw	Positive	cell movement of granulocytes	1.98E-04	118
Rumen	Rice straw	Positive	apoptosis of kidney cell lines	1.98E-04	79
Rumen	Rice straw	Positive	intermediate disease stage peripheral arterial disease	1.98E-04	55
Rumen	Rice straw	Positive	replication of Influenza virus	2.05E-04	94
Rumen	Rice straw	Positive	Schizophrenia	2.05E-04	135
Rumen	Rice	Positive	cellular infiltration by leukocytes	2.05E-04	123

	straw				
Rumen	Rice straw	Positive	double-stranded DNA break repair of cells	2.05E-04	24
Rumen	Rice straw	Positive	abnormal morphology of brain	2.05E-04	140
Rumen	Rice straw	Positive	activation of endothelial cells	2.06E-04	28
Rumen	Rice straw	Positive	proliferation of tumor cells	2.11E-04	147
Rumen	Rice straw	Positive	development of hematopoietic system	2.11E-04	82
Rumen	Rice straw	Positive	long-term potentiation of synapse	2.15E-04	52
Rumen	Rice straw	Positive	migration of muscle cells	2.17E-04	57
Rumen	Rice straw	Positive	benign neoplasm of female genital organ	2.18E-04	134
Rumen	Rice straw	Positive	Fibrosis	2.21E-04	165
Rumen	Rice straw	Positive	autophagy	2.21E-04	119
Rumen	Rice straw	Positive	development of connective tissue cells	2.29E-04	74
Rumen	Rice straw	Positive	fusion of vesicles	2.31E-04	29
Rumen	Rice straw	Positive	cellular infiltration by macrophages	2.32E-04	55
Rumen	Rice straw	Positive	abnormal morphology of limb	2.32E-04	76
Rumen	Rice straw	Positive	gastrointestinal adenocarcinoma	2.34E-04	1183
Rumen	Rice straw	Positive	tumorigenesis of malignant tumor	2.35E-04	88
Rumen	Rice straw	Positive	neurotransmission	2.39E-04	130
Rumen	Rice straw	Positive	abnormal morphology of skull	2.40E-04	73
Rumen	Rice straw	Positive	abnormal morphology of reproductive system	2.40E-04	158
Rumen	Rice straw	Positive	schizophrenia spectrum disorder	2.41E-04	140
Rumen	Rice straw	Positive	G1 phase	2.44E-04	123
Rumen	Rice	Positive	proliferation of bone marrow cells	2.45E-04	43

	straw				
Rumen	Rice straw	Positive	quantity of bone	2.51E-04	81
Rumen	Rice straw	Positive	resorption of bone	2.52E-04	57
Rumen	Rice straw	Positive	formation of lung	2.57E-04	97
Rumen	Rice straw	Positive	cell movement of sarcoma cell lines	2.68E-04	35
Rumen	Rice straw	Positive	Dermatitis	2.68E-04	136
Rumen	Rice straw	Positive	development of phagocytes	2.74E-04	31
Rumen	Rice straw	Positive	morphology of muscle	2.76E-04	107
Rumen	Rice straw	Positive	morphology of genital organ	2.76E-04	133
Rumen	Rice straw	Positive	connective or soft tissue tumor	2.79E-04	214
Rumen	Rice straw	Positive	cell death of hepatoma cell lines	2.84E-04	58
Rumen	Rice straw	Positive	quantity of diacylglycerol	2.84E-04	24
Rumen	Rice straw	Positive	cell viability of connective tissue cells	2.84E-04	47
Rumen	Rice straw	Positive	entry into interphase	2.89E-04	53
Rumen	Rice straw	Positive	ossification of bone	2.89E-04	53
Rumen	Rice straw	Positive	organization of actin filaments	2.89E-04	38
Rumen	Rice straw	Positive	exocytosis by cells	2.92E-04	45
Rumen	Rice straw	Positive	migration of vascular endothelial cells	2.93E-04	57
Rumen	Rice straw	Positive	excitatory postsynaptic potential	2.94E-04	44
Rumen	Rice straw	Positive	juvenile rheumatoid arthritis	2.94E-04	44
Rumen	Rice straw	Positive	proliferation of epithelial cell lines	2.95E-04	76
Rumen	Rice straw	Positive	morphology of neuroglia	2.96E-04	42
Rumen	Rice	Positive	proliferation of mononuclear leukocytes	3.11E-04	223

	straw				
Rumen	Rice straw	Positive	cell death of heart	3.14E-04	75
Rumen	Rice straw	Positive	migration of cancer cells	3.17E-04	65
Rumen	Rice straw	Positive	quantity of lymphatic system component	3.18E-04	119
Rumen	Rice straw	Positive	synthesis of triacylglycerol	3.21E-04	27
Rumen	Rice straw	Positive	uterine tumor	3.27E-04	892
Rumen	Rice straw	Positive	morphology of skin	3.27E-04	88
Rumen	Rice straw	Positive	morphology of axial skeleton	3.28E-04	67
Rumen	Rice straw	Positive	function of phagocytes	3.28E-04	98
Rumen	Rice straw	Positive	apoptosis of neutrophils	3.31E-04	25
Rumen	Rice straw	Positive	inflammation of organ	3.32E-04	345
Rumen	Rice straw	Positive	differentiation of leukocytes	3.36E-04	204
Rumen	Rice straw	Positive	differentiation of nervous system	3.36E-04	154
Rumen	Rice straw	Positive	neural tube defect	3.36E-04	53
Rumen	Rice straw	Positive	death of embryo	3.49E-04	52
Rumen	Rice straw	Positive	synaptic transmission of cells	3.57E-04	81
Rumen	Rice straw	Positive	neovascularization of organ	3.62E-04	40
Rumen	Rice straw	Positive	abnormal morphology of cerebral cortex	3.66E-04	62
Rumen	Rice straw	Positive	cell movement of tumor cells	3.66E-04	62
Rumen	Rice straw	Negative	proliferation of cells	5.24E-46	1570
Rumen	Rice straw	Negative	cancer	1.70E-43	3953
Rumen	Rice straw	Negative	malignant solid tumor	3.99E-41	3902
Rumen	Rice	Negative	organismal death	7.25E-38	1060

	straw				
Rumen	Rice straw	Negative	morbidity or mortality	2.02E-37	1070
Rumen	Rice straw	Negative	cell death	4.96E-37	1400
Rumen	Rice straw	Negative	necrosis	5.04E-32	1100
Rumen	Rice straw	Negative	morphology of cells	3.19E-30	860
Rumen	Rice straw	Negative	apoptosis	2.69E-29	1113
Rumen	Rice straw	Negative	cell movement	2.97E-25	906
Rumen	Rice straw	Negative	organization of cytoplasm	1.01E-24	681
Rumen	Rice straw	Negative	transport of molecule	3.14E-24	709
Rumen	Rice straw	Negative	abdominal neoplasm	5.03E-24	3199
Rumen	Rice straw	Negative	tumorigenesis of tissue	4.61E-23	3264
Rumen	Rice straw	Negative	neoplasia of epithelial tissue	3.00E-22	3210
Rumen	Rice straw	Negative	abdominal cancer	3.82E-22	3153
Rumen	Rice straw	Negative	cell survival	1.74E-21	614
Rumen	Rice straw	Negative	epithelial cancer	3.42E-21	3175
Rumen	Rice straw	Negative	cell viability	4.24E-21	577
Rumen	Rice straw	Negative	cell death of tumor cell lines	4.86E-21	662
Rumen	Rice straw	Negative	migration of cells	8.13E-21	801
Rumen	Rice straw	Negative	organization of cytoskeleton	1.07E-20	611
Rumen	Rice straw	Negative	digestive organ tumor	1.91E-20	2771
Rumen	Rice straw	Negative	digestive system cancer	1.99E-20	2747
Rumen	Rice straw	Negative	cellular homeostasis	1.81E-19	653
Rumen	Rice	Negative	expression of RNA	6.31E-19	880

	straw				
Rumen	Rice straw	Negative	differentiation of cells	1.28E-18	916
Rumen	Rice straw	Negative	abnormal morphology of cells	1.77E-18	552
Rumen	Rice straw	Negative	angiogenesis	1.18E-17	411
Rumen	Rice straw	Negative	transcription	1.73E-17	817
Rumen	Rice straw	Negative	microtubule dynamics	2.54E-17	516
Rumen	Rice straw	Negative	transcription of RNA	3.97E-17	765
Rumen	Rice straw	Negative	apoptosis of tumor cell lines	4.92E-17	525
Rumen	Rice straw	Negative	morphology of cardiovascular system	7.51E-17	293
Rumen	Rice straw	Negative	vasculogenesis	3.43E-16	338
Rumen	Rice straw	Negative	synthesis of lipid	2.94E-14	322
Rumen	Rice straw	Negative	perinatal death	2.94E-14	274
Rumen	Rice straw	Negative	morphology of body cavity	4.98E-14	505
Rumen	Rice straw	Negative	formation of cellular protrusions	6.49E-14	394
Rumen	Rice straw	Negative	development of body trunk	7.14E-14	475
Rumen	Rice straw	Negative	concentration of lipid	8.06E-14	358
Rumen	Rice straw	Negative	quantity of cells	8.06E-14	714
Rumen	Rice straw	Negative	transcription of DNA	9.43E-14	629
Rumen	Rice straw	Negative	cell movement of tumor cell lines	1.17E-13	373
Rumen	Rice straw	Negative	size of body	1.70E-13	372
Rumen	Rice straw	Negative	phosphorylation of protein	1.70E-13	327
Rumen	Rice straw	Negative	proliferation of tumor cell lines	1.71E-13	632
Rumen	Rice	Negative	Movement Disorders	1.94E-13	451

	straw				
Rumen	Rice straw	Negative	abnormal morphology of epithelial tissue	2.34E-13	199
Rumen	Rice straw	Negative	cell viability of tumor cell lines	8.66E-13	341
Rumen	Rice straw	Negative	development of cytoplasm	1.88E-12	214
Rumen	Rice straw	Negative	abnormal morphology of cardiovascular system	2.53E-12	256
Rumen	Rice straw	Negative	cell death of connective tissue cells	2.53E-12	270
Rumen	Rice straw	Negative	growth of organism	2.66E-12	361
Rumen	Rice straw	Negative	abnormal morphology of body cavity	3.03E-12	476
Rumen	Rice straw	Negative	abnormal morphology of thoracic cavity	6.45E-12	262
Rumen	Rice straw	Negative	organization of organelle	7.21E-12	243
Rumen	Rice straw	Negative	interphase	8.16E-12	289
Rumen	Rice straw	Negative	neurological signs	8.37E-12	287
Rumen	Rice straw	Negative	morphology of heart	1.47E-11	188
Rumen	Rice straw	Negative	transactivation	1.66E-11	257
Rumen	Rice straw	Negative	necrosis of epithelial tissue	1.82E-11	261
Rumen	Rice straw	Negative	protein kinase cascade	1.88E-11	194
Rumen	Rice straw	Negative	quantity of blood cells	2.06E-11	429
Rumen	Rice straw	Negative	quantity of leukocytes	2.17E-11	387
Rumen	Rice straw	Negative	quantity of connective tissue	2.40E-11	266
Rumen	Rice straw	Negative	dyskinesia	3.20E-11	271
Rumen	Rice straw	Negative	development of vasculature	3.20E-11	192
Rumen	Rice straw	Negative	disorder of basal ganglia	3.69E-11	329
Rumen	Rice	Negative	abnormal morphology of head	4.02E-11	354

	straw				
Rumen	Rice straw	Negative	invasion of cells	6.92E-11	367
Rumen	Rice straw	Negative	cell death of epithelial cells	8.10E-11	222
Rumen	Rice straw	Negative	morphology of head	8.16E-11	369
Rumen	Rice straw	Negative	cell death of cervical cancer cell lines	8.90E-11	163
Rumen	Rice straw	Negative	chorea	1.11E-10	253
Rumen	Rice straw	Negative	Huntington's Disease	1.22E-10	252
Rumen	Rice straw	Negative	morphology of connective tissue	2.12E-10	206
Rumen	Rice straw	Negative	Growth Failure	2.67E-10	260
Rumen	Rice straw	Negative	transactivation of RNA	3.48E-10	238
Rumen	Rice straw	Negative	formation of cytoskeleton	4.47E-10	171
Rumen	Rice straw	Negative	abnormal morphology of abdomen	5.87E-10	365
Rumen	Rice straw	Negative	proliferation of blood cells	6.01E-10	352
Rumen	Rice straw	Negative	activation of DNA endogenous promoter	6.83E-10	488
Rumen	Rice straw	Negative	migration of tumor cell lines	6.84E-10	300
Rumen	Rice straw	Negative	proliferation of connective tissue cells	6.93E-10	256
Rumen	Rice straw	Negative	cell movement of blood cells	6.93E-10	364
Rumen	Rice straw	Negative	cell transformation	6.98E-10	204
Rumen	Rice straw	Negative	migration of blood cells	7.48E-10	363
Rumen	Rice straw	Negative	apoptosis of cervical cancer cell lines	7.54E-10	132
Rumen	Rice straw	Negative	metabolism of protein	8.92E-10	400
Rumen	Rice straw	Negative	leukocyte migration	9.06E-10	362
Rumen	Rice	Negative	cell death of epithelial cell lines	9.29E-10	132

	straw				
Rumen	Rice straw	Negative	growth of epithelial tissue	9.29E-10	289
Rumen	Rice straw	Negative	development of body axis	9.82E-10	429
Rumen	Rice straw	Negative	growth of connective tissue	1.02E-09	275
Rumen	Rice straw	Negative	Viral Infection	1.13E-09	631
Rumen	Rice straw	Negative	morphology of nervous system	1.20E-09	328
Rumen	Rice straw	Negative	proliferation of neuronal cells	1.36E-09	248
Rumen	Rice straw	Negative	concentration of fatty acid	1.67E-09	130
Rumen	Rice straw	Negative	formation of muscle	1.89E-09	177
Rumen	Rice straw	Negative	growth of muscle tissue	2.68E-09	167
Rumen	Rice straw	Negative	abnormal morphology of heart	2.70E-09	171
Rumen	Rice straw	Negative	activation of enzyme	2.70E-09	173
Rumen	Rice straw	Negative	proliferation of muscle cells	2.86E-09	166
Rumen	Rice straw	Negative	development of head	3.06E-09	400
Rumen	Rice straw	Negative	neuronal cell death	3.10E-09	287
Rumen	Rice straw	Negative	autophagy	3.78E-09	167
Rumen	Rice straw	Negative	growth of embryo	4.93E-09	207
Rumen	Rice straw	Negative	proliferation of immune cells	5.01E-09	328
Rumen	Rice straw	Negative	survival of organism	5.78E-09	294
Rumen	Rice straw	Negative	morphology of respiratory system	6.11E-09	141
Rumen	Rice straw	Negative	abnormal morphology of nervous system	6.45E-09	302
Rumen	Rice straw	Negative	ubiquitination	7.10E-09	151
Rumen	Rice	Negative	abnormal morphology of respiratory system	7.10E-09	139

	straw				
Rumen	Rice straw	Negative	invasion of tumor cell lines	7.37E-09	279
Rumen	Rice straw	Negative	neuromuscular disease	7.82E-09	361
Rumen	Rice straw	Negative	neonatal death	9.53E-09	191
Rumen	Rice straw	Negative	morphology of bone	1.07E-08	195
Rumen	Rice straw	Negative	function of cardiovascular system	1.34E-08	163
Rumen	Rice straw	Negative	ubiquitination of protein	1.34E-08	148
Rumen	Rice straw	Negative	autosomal recessive disease	1.34E-08	374
Rumen	Rice straw	Negative	breast or colorectal cancer	1.61E-08	1802
Rumen	Rice straw	Negative	cell death of blood cells	1.61E-08	288
Rumen	Rice straw	Negative	metabolism of carbohydrate	2.02E-08	260
Rumen	Rice straw	Negative	fatty acid metabolism	2.22E-08	252
Rumen	Rice straw	Negative	secretion of molecule	2.25E-08	233
Rumen	Rice straw	Negative	dysgenesis	2.75E-08	228
Rumen	Rice straw	Negative	abnormal morphology of bone	2.80E-08	188
Rumen	Rice straw	Negative	quantity of lymphocytes	2.92E-08	292
Rumen	Rice straw	Negative	degeneration of nervous system	2.93E-08	129
Rumen	Rice straw	Negative	Organ Degeneration	3.00E-08	210
Rumen	Rice straw	Negative	cell movement of leukocytes	3.48E-08	314
Rumen	Rice straw	Negative	quantity of mononuclear leukocytes	3.72E-08	302
Rumen	Rice straw	Negative	accumulation of lipid	4.78E-08	133
Rumen	Rice straw	Negative	morphology of digestive system	4.96E-08	239
Rumen	Rice	Negative	abnormal morphology of embryonic tissue	5.12E-08	230

	straw				
Rumen	Rice straw	Negative	apoptosis of epithelial cell lines	5.50E-08	103
Rumen	Rice straw	Negative	proliferation of mononuclear leukocytes	5.88E-08	302
Rumen	Rice straw	Negative	cell death of fibroblast cell lines	5.99E-08	183
Rumen	Rice straw	Negative	Hypoplasia	6.18E-08	213
Rumen	Rice straw	Negative	engulfment of cells	6.59E-08	175
Rumen	Rice straw	Negative	abnormal morphology of digestive system	6.94E-08	228
Rumen	Rice straw	Negative	formation of cells	8.09E-08	392
Rumen	Rice straw	Negative	proliferation of lymphocytes	8.15E-08	297
Rumen	Rice straw	Negative	development of abdomen	8.82E-08	241
Rumen	Rice straw	Negative	development of lymphatic system component	9.51E-08	136
Rumen	Rice straw	Negative	fibrogenesis	9.61E-08	176
Rumen	Rice straw	Negative	congenital anomaly of musculoskeletal system	9.62E-08	300
Rumen	Rice straw	Negative	cell cycle progression	1.28E-07	414
Rumen	Rice straw	Negative	cardiogenesis	1.28E-07	199
Rumen	Rice straw	Negative	autosomal dominant disease	1.35E-07	262
Rumen	Rice straw	Negative	adhesion of tumor cell lines	1.38E-07	127
Rumen	Rice straw	Negative	seizure disorder	1.38E-07	198
Rumen	Rice straw	Negative	growth of embryonic tissue	1.39E-07	122
Rumen	Rice straw	Negative	differentiation of connective tissue cells	1.40E-07	264
Rumen	Rice straw	Negative	cell death of immune cells	1.65E-07	270
Rumen	Rice straw	Negative	development of digestive system	1.77E-07	164
Rumen	Rice	Negative	Neurodegeneration	1.78E-07	135

	straw				
Rumen	Rice straw	Negative	differentiation of connective tissue	1.89E-07	296
Rumen	Rice straw	Negative	function of blood cells	1.89E-07	242
Rumen	Rice straw	Negative	proliferation of fibroblasts	1.90E-07	152
Rumen	Rice straw	Negative	morphology of vessel	1.90E-07	134
Rumen	Rice straw	Negative	function of leukocytes	2.05E-07	223
Rumen	Rice straw	Negative	cell death of muscle cells	2.12E-07	145
Rumen	Rice straw	Negative	quantity of lymphoid organ	2.28E-07	134
Rumen	Rice straw	Negative	formation of filaments	2.41E-07	169
Rumen	Rice straw	Negative	quantity of lymphatic system component	2.67E-07	161
Rumen	Rice straw	Negative	endocytosis	3.26E-07	148
Rumen	Rice straw	Negative	quantity of T lymphocytes	3.69E-07	221
Rumen	Rice straw	Negative	cell spreading	4.03E-07	121
Rumen	Rice straw	Negative	development of lymphatic system	4.46E-07	152
Rumen	Rice straw	Negative	proliferation of embryonic cells	4.48E-07	110
Rumen	Rice straw	Negative	metabolism of membrane lipid derivative	4.51E-07	164
Rumen	Rice straw	Negative	arrest in interphase	4.74E-07	175
Rumen	Rice straw	Negative	Bleeding	4.81E-07	178
Rumen	Rice straw	Negative	mass of connective tissue	4.82E-07	91
Rumen	Rice straw	Negative	binding of DNA	4.82E-07	215
Rumen	Rice straw	Negative	hepatocellular carcinoma	4.82E-07	1515
Rumen	Rice straw	Negative	cell death of muscle	4.82E-07	147
Rumen	Rice	Negative	catabolism of protein	4.82E-07	246

	straw				
Rumen	Rice straw	Negative	hepatobiliary system cancer	4.82E-07	1569
Rumen	Rice straw	Negative	formation of filopodia	5.06E-07	77
Rumen	Rice straw	Negative	proliferation of smooth muscle cells	5.26E-07	124
Rumen	Rice straw	Negative	synthesis of DNA	5.26E-07	175
Rumen	Rice straw	Negative	replication of virus	5.41E-07	230
Rumen	Rice straw	Negative	morphology of cardiovascular tissue	6.06E-07	45
Rumen	Rice straw	Negative	necrosis of muscle	6.16E-07	146
Rumen	Rice straw	Negative	gastrointestinal tract cancer	6.45E-07	1814
Rumen	Rice straw	Negative	activation of cells	7.05E-07	384
Rumen	Rice straw	Negative	abnormal morphology of skeleton	7.22E-07	118
Rumen	Rice straw	Negative	liver tumor	7.37E-07	1570
Rumen	Rice straw	Negative	liver cancer	7.39E-07	1560
Rumen	Rice straw	Negative	apoptosis of fibroblast cell lines	7.46E-07	140
Rumen	Rice straw	Negative	Gastrointestinal Tract Cancer and Tumors	8.02E-07	1841
Rumen	Rice straw	Negative	morphology of skeleton	8.04E-07	121
Rumen	Rice straw	Negative	cell death of kidney cells	8.22E-07	148
Rumen	Rice straw	Negative	proliferation of epithelial cells	8.22E-07	203
Rumen	Rice straw	Negative	replication of RNA virus	8.74E-07	208
Rumen	Rice straw	Negative	cell death of kidney cell lines	9.00E-07	130
Rumen	Rice straw	Negative	respiratory system development	9.09E-07	145
Rumen	Rice straw	Negative	activation of Protein kinase	9.26E-07	110
Rumen	Rice	Negative	formation of lung	9.76E-07	129

	straw				
Rumen	Rice straw	Negative	Hypertrophy	1.15E-06	195
Rumen	Rice straw	Negative	peripheral arterial disease	1.15E-06	94
Rumen	Rice straw	Negative	cell movement of endothelial cells	1.20E-06	149
Rumen	Rice straw	Negative	mass of adipose tissue	1.39E-06	85
Rumen	Rice straw	Negative	behavior	1.40E-06	386
Rumen	Rice straw	Negative	development of epithelial tissue	1.41E-06	211
Rumen	Rice straw	Negative	apoptosis of neurons	1.55E-06	180
Rumen	Rice straw	Negative	growth of neurites	1.59E-06	190
Rumen	Rice straw	Negative	formation of eye	1.61E-06	181
Rumen	Rice straw	Negative	morphology of skin	1.61E-06	117
Rumen	Rice straw	Negative	differentiation of muscle cell lines	1.62E-06	74
Rumen	Rice straw	Negative	vascularization	1.62E-06	103
Rumen	Rice straw	Negative	autophagy of cells	1.72E-06	114
Rumen	Rice straw	Negative	homing of cells	1.73E-06	226
Rumen	Rice straw	Negative	function of muscle	1.82E-06	146
Rumen	Rice straw	Negative	benign neoplasia	1.86E-06	362
Rumen	Rice straw	Negative	proliferation of hematopoietic cells	1.99E-06	110
Rumen	Rice straw	Negative	synthesis of fatty acid	2.06E-06	132
Rumen	Rice straw	Negative	function of smooth muscle	2.06E-06	46
Rumen	Rice straw	Negative	cell movement of fibroblasts	2.08E-06	82
Rumen	Rice straw	Negative	growth of plasma membrane projections	2.09E-06	191
Rumen	Rice	Negative	morphology of reproductive system	2.11E-06	217

	straw				
Rumen	Rice straw	Negative	neuritogenesis	2.15E-06	235
Rumen	Rice straw	Negative	transformation of fibroblast cell lines	2.16E-06	111
Rumen	Rice straw	Negative	migration of connective tissue cells	2.18E-06	81
Rumen	Rice straw	Negative	abnormal morphology of reproductive system	2.19E-06	207
Rumen	Rice straw	Negative	morphology of endothelial tissue	2.40E-06	42
Rumen	Rice straw	Negative	polymerization of protein	2.49E-06	154
Rumen	Rice straw	Negative	cognition	2.49E-06	186
Rumen	Rice straw	Negative	seizures	2.51E-06	165
Rumen	Rice straw	Negative	cell movement of connective tissue cells	2.52E-06	97
Rumen	Rice straw	Negative	small GTPase mediated signal transduction	2.55E-06	82
Rumen	Rice straw	Negative	phosphorylation of L-amino acid	2.72E-06	96
Rumen	Rice straw	Negative	morphology of central nervous system	2.87E-06	208
Rumen	Rice straw	Negative	differentiation of embryonic tissue	3.05E-06	109
Rumen	Rice straw	Negative	interphase of tumor cell lines	3.13E-06	153
Rumen	Rice straw	Negative	shape change of tumor cell lines	3.24E-06	70
Rumen	Rice straw	Negative	morphology of genital organ	3.32E-06	174
Rumen	Rice straw	Negative	synthesis of reactive oxygen species	3.46E-06	195
Rumen	Rice straw	Negative	transport of alpha-amino acid	3.55E-06	37
Rumen	Rice straw	Negative	cellular infiltration	3.57E-06	179
Rumen	Rice straw	Negative	transport of protein	3.65E-06	127
Rumen	Rice straw	Negative	abnormal morphology of endothelial tissue	3.77E-06	34
Rumen	Rice	Negative	peripheral vascular disease	3.85E-06	157

	straw				
Rumen	Rice straw	Negative	epileptic seizure	3.98E-06	75
Rumen	Rice straw	Negative	homing	4.07E-06	230
Rumen	Rice straw	Negative	quantity of carbohydrate	4.17E-06	209
Rumen	Rice straw	Negative	cell death of sarcoma cell lines	4.18E-06	98
Rumen	Rice straw	Negative	differentiation of muscle	4.25E-06	127
Rumen	Rice straw	Negative	neovascularization	4.46E-06	73
Rumen	Rice straw	Negative	proliferation of hematopoietic progenitor cells	4.54E-06	104
Rumen	Rice straw	Negative	proliferation of T lymphocytes	4.58E-06	241
Rumen	Rice straw	Negative	proliferation of fibroblast cell lines	4.63E-06	173
Rumen	Rice straw	Negative	development of blood cells	4.63E-06	259
Rumen	Rice straw	Negative	Dermatitis	4.75E-06	177
Rumen	Rice straw	Negative	formation of plasma membrane projections	4.75E-06	238
Rumen	Rice straw	Negative	cell death of embryonic cell lines	4.86E-06	103
Rumen	Rice straw	Negative	cell death of heart cells	5.23E-06	95
Rumen	Rice straw	Negative	glandular intraepithelial neoplasm	5.23E-06	58
Rumen	Rice straw	Negative	cellular infiltration by leukocytes	5.51E-06	159
Rumen	Rice straw	Negative	apoptosis of epithelial cells	5.57E-06	120
Rumen	Rice straw	Negative	disorder of lipid metabolism	5.65E-06	86
Rumen	Rice straw	Negative	stress response of cells	5.71E-06	63
Rumen	Rice straw	Negative	development of cardiovascular tissue	5.87E-06	152
Rumen	Rice straw	Negative	synthesis of carbohydrate	5.92E-06	173
Rumen	Rice	Negative	damage of epithelial tissue	5.98E-06	50

	straw				
Rumen	Rice straw	Negative	necrosis of kidney	6.05E-06	150
Rumen	Rice straw	Negative	growth of tumor	6.43E-06	311
Rumen	Rice straw	Negative	growth of lymphatic system component	6.49E-06	84
Rumen	Rice straw	Negative	breast or ovarian cancer	6.69E-06	709
Rumen	Rice straw	Negative	formation of actin filaments	6.69E-06	127
Rumen	Rice straw	Negative	morphology of brain	6.97E-06	188
Rumen	Rice straw	Negative	cellular infiltration of cells	7.13E-06	162
Rumen	Rice straw	Negative	adenocarcinoma	7.69E-06	2293
Rumen	Rice straw	Negative	apoptosis of blood cells	7.71E-06	199
Rumen	Rice straw	Negative	differentiation of muscle cells	7.71E-06	118
Rumen	Rice straw	Negative	S phase	7.81E-06	110
Rumen	Rice straw	Negative	cell death of cardiomyocytes	7.90E-06	92
Rumen	Rice straw	Negative	quantity of epithelial tissue	7.90E-06	78
Rumen	Rice straw	Negative	transport of amino acids	8.01E-06	61
Rumen	Rice straw	Negative	morphology of lymphatic system component	8.32E-06	169
Rumen	Rice straw	Negative	abnormal morphology of genital organ	8.36E-06	166
Rumen	Rice straw	Negative	apoptosis of heart	8.50E-06	83
Rumen	Rice straw	Negative	generation of blood cells	8.71E-06	73
Rumen	Rice straw	Negative	morphology of muscle cells	8.71E-06	73
Rumen	Rice straw	Negative	organization of actin cytoskeleton	9.21E-06	125
Rumen	Rice straw	Negative	cell death of heart	9.33E-06	97
Rumen	Rice	Negative	apoptosis of connective tissue cells	9.34E-06	123

	straw				
Rumen	Rice straw	Negative	death of perinatal stage organism	9.69E-06	40
Rumen	Rice straw	Negative	morphology of muscle	9.69E-06	138
Rumen	Rice straw	Negative	orientation of cells	9.71E-06	78
Rumen	Rice straw	Negative	long-term potentiation	9.71E-06	103
Rumen	Rice straw	Negative	cancer of secretory structure	9.77E-06	727
Rumen	Rice straw	Negative	prostatic intraepithelial neoplasia	9.77E-06	55
Rumen	Rice straw	Negative	cell death of fibroblasts	9.78E-06	111
Rumen	Rice straw	Negative	development of sensory organ	9.81E-06	224
Rumen	Rice straw	Negative	quantity of hematopoietic progenitor cells	9.95E-06	178
Rumen	Rice straw	Negative	ion homeostasis of cells	1.02E-05	221
Rumen	Rice straw	Negative	intermediate disease stage peripheral arterial disease	1.02E-05	70
Rumen	Rice straw	Negative	metabolism of nucleic acid component or derivative	1.02E-05	207
Rumen	Rice straw	Negative	learning	1.03E-05	169
Rumen	Rice straw	Negative	development of gastrointestinal tract	1.05E-05	110
Rumen	Rice straw	Negative	sprouting	1.06E-05	157
Rumen	Rice straw	Negative	secretory pathway	1.07E-05	85
Rumen	Rice straw	Negative	apoptosis of heart cells	1.09E-05	82
Rumen	Rice straw	Negative	Edema	1.11E-05	141
Rumen	Rice straw	Negative	midline defect	1.14E-05	111
Rumen	Rice straw	Negative	epilepsy	1.18E-05	127
Rumen	Rice straw	Negative	hypersensitive reaction	1.19E-05	173
Rumen	Rice	Negative	advanced stage peripheral arterial disease	1.21E-05	67

	straw				
Rumen	Rice straw	Negative	gastrointestinal carcinoma	1.21E-05	1615
Rumen	Rice straw	Negative	transport of L-amino acid	1.22E-05	33
Rumen	Rice straw	Negative	metabolism of monosaccharide	1.22E-05	56
Rumen	Rice straw	Negative	G2 phase	1.22E-05	112
Rumen	Rice straw	Negative	morphology of heart ventricle	1.22E-05	83
Rumen	Rice straw	Negative	apoptosis of cardiomyocytes	1.23E-05	80
Rumen	Rice straw	Negative	formation of lymphatic system component	1.23E-05	121
Rumen	Rice straw	Negative	organization of filaments	1.23E-05	88
Rumen	Rice straw	Negative	generation of T lymphocytes	1.33E-05	53
Rumen	Rice straw	Negative	release of L-amino acid	1.34E-05	40
Rumen	Rice straw	Negative	abnormal morphology of central nervous system	1.36E-05	193
Rumen	Rice straw	Negative	cell movement of mononuclear leukocytes	1.36E-05	185
Rumen	Rice straw	Negative	metabolism of reactive oxygen species	1.42E-05	199
Rumen	Rice straw	Negative	endothelial cell development	1.46E-05	144
Rumen	Rice straw	Negative	fusion of cellular membrane	1.46E-05	31
Rumen	Rice straw	Negative	abnormal morphology of skull	1.46E-05	93
Rumen	Rice straw	Negative	infection by RNA virus	1.47E-05	334
Rumen	Rice straw	Negative	generation of leukocytes	1.47E-05	71
Rumen	Rice straw	Negative	morphology of blood vessel	1.47E-05	117
Rumen	Rice straw	Negative	synthesis of terpenoid	1.47E-05	117
Rumen	Rice straw	Negative	morphology of skull	1.48E-05	95
Rumen	Rice	Negative	outgrowth of neurites	1.48E-05	163

	straw				
Rumen	Rice straw	Negative	apoptosis of muscle	1.53E-05	109
Rumen	Rice straw	Negative	outgrowth of plasma membrane projections	1.53E-05	164
Rumen	Rice straw	Negative	uptake of carbohydrate	1.54E-05	120
Rumen	Rice straw	Negative	development of neurons	1.57E-05	303
Rumen	Rice straw	Negative	formation of lymphoid organ	1.66E-05	110
Rumen	Rice straw	Negative	apoptosis of kidney cell lines	1.66E-05	100
Rumen	Rice straw	Negative	abnormal morphology of skin	1.66E-05	102
Rumen	Rice straw	Negative	cellular degradation	1.66E-05	106
Rumen	Rice straw	Negative	morphology of pulmonary alveolus	1.66E-05	54
Rumen	Rice straw	Negative	transmembrane potential of mitochondria	1.68E-05	91
Rumen	Rice straw	Negative	migration of endothelial cells	1.69E-05	134
Rumen	Rice straw	Negative	homeostasis of leukocytes	1.73E-05	219
Rumen	Rice straw	Negative	degeneration of cells	1.75E-05	140
Rumen	Rice straw	Negative	interphase of cervical cancer cell lines	1.88E-05	40
Rumen	Rice straw	Negative	modification of peptide	1.91E-05	49
Rumen	Rice straw	Negative	incidence of tumor	1.91E-05	131
Rumen	Rice straw	Negative	abnormal morphology of brain	1.91E-05	178
Rumen	Rice straw	Negative	abnormal morphology of body wall	1.93E-05	48
Rumen	Rice straw	Negative	apoptosis of muscle cells	1.93E-05	108
Rumen	Rice straw	Negative	development of endothelial tissue	1.93E-05	148
Rumen	Rice straw	Negative	cell death of bone cancer cell lines	1.95E-05	76
Rumen	Rice	Negative	inflammatory response	1.96E-05	301

	straw				
Rumen	Rice straw	Negative	quantity of adipose tissue	1.99E-05	115
Rumen	Rice straw	Negative	development of leukocytes	2.12E-05	231
Rumen	Rice straw	Negative	metabolism of DNA	2.14E-05	160
Rumen	Rice straw	Negative	infection by Retroviridae	2.15E-05	279
Rumen	Rice straw	Negative	oxidation of lipid	2.16E-05	90
Rumen	Rice straw	Negative	formation of skin	2.19E-05	148
Rumen	Rice straw	Negative	female genital tract serous cancer	2.26E-05	155
Rumen	Rice straw	Negative	branching of cells	2.29E-05	149
Rumen	Rice straw	Negative	chemotaxis of cells	2.31E-05	208
Rumen	Rice straw	Negative	concentration of sterol	2.39E-05	125
Rumen	Rice straw	Negative	release of L-glutamic acid	2.50E-05	38
Rumen	Rice straw	Negative	cell death of hematopoietic cells	2.50E-05	99
Rumen	Rice straw	Negative	differentiation of epithelial tissue	2.50E-05	145
Rumen	Rice straw	Negative	polarization of cells	2.58E-05	75
Rumen	Rice straw	Negative	relaxation of muscle	2.64E-05	40
Rumen	Rice straw	Negative	morphology of lung	2.67E-05	80
Rumen	Rice straw	Negative	adhesion of endothelial cell lines	2.70E-05	22
Rumen	Rice straw	Negative	colony formation	2.71E-05	211
Rumen	Rice straw	Negative	oxidation of fatty acid	2.76E-05	74
Rumen	Rice straw	Negative	cell death of neuroblastoma cell lines	2.77E-05	82
Rumen	Rice straw	Negative	plasticity of synapse	2.82E-05	54
Rumen	Rice	Negative	Lymphocyte homeostasis	2.86E-05	214

	straw				
Rumen	Rice straw	Negative	proliferation of vascular smooth muscle cells	2.88E-05	67
Rumen	Rice straw	Negative	size of lesion	2.89E-05	105
Rumen	Rice straw	Negative	quantity of thymus gland	2.92E-05	97
Rumen	Rice straw	Negative	development of connective tissue	2.92E-05	149
Rumen	Rice straw	Negative	cell death of brain	2.97E-05	119
Rumen	Rice straw	Negative	cell movement of myeloid cells	3.04E-05	212
Rumen	Rice straw	Negative	abnormal morphology of internal genitalia	3.07E-05	141
Rumen	Rice straw	Negative	generation of lymphocytes	3.09E-05	62
Rumen	Rice straw	Negative	colony formation of cells	3.18E-05	194
Rumen	Rice straw	Negative	transport of carbohydrate	3.35E-05	82
Rumen	Rice straw	Negative	cell death of leukemia cell lines	3.40E-05	129
Rumen	Rice straw	Negative	morphology of endothelial cells	3.43E-05	31
Rumen	Rice straw	Negative	HIV infection	3.46E-05	275
Rumen	Rice straw	Negative	congenital malformation of skeleton	3.51E-05	168
Rumen	Rice straw	Negative	MAPKKK cascade	3.54E-05	86
Rumen	Rice straw	Negative	accumulation of steroid	3.62E-05	39
Rumen	Rice straw	Negative	quantity of antigen presenting cells	3.70E-05	113
Rumen	Rice straw	Negative	synthesis of nitric oxide	3.70E-05	113
Rumen	Rice straw	Negative	cell viability of cervical cancer cell lines	3.70E-05	92
Rumen	Rice straw	Negative	degeneration of central nervous system	3.87E-05	58
Rumen	Rice straw	Negative	mammary tumor	3.89E-05	605
Rumen	Rice	Negative	uptake of monosaccharide	4.14E-05	110

	straw				
Rumen	Rice straw	Negative	I-kappaB kinase/NF-kappaB cascade	4.14E-05	74
Rumen	Rice straw	Negative	chemotaxis	4.18E-05	213
Rumen	Rice straw	Negative	mass of fat pad	4.29E-05	50
Rumen	Rice straw	Negative	cell movement of lymphocytes	4.46E-05	157
Rumen	Rice straw	Negative	concentration of triacylglycerol	4.59E-05	124
Rumen	Rice straw	Negative	morphology of mouth	4.70E-05	59
Rumen	Rice straw	Negative	exocytosis	4.75E-05	80
Rumen	Rice straw	Negative	transport of ion	4.78E-05	198
Rumen	Rice straw	Negative	cell death of liver cells	4.90E-05	82
Rumen	Rice straw	Negative	hypoplasia of organ	4.95E-05	176
Rumen	Rice straw	Negative	migration of fibroblasts	4.95E-05	62
Rumen	Rice straw	Negative	formation of vesicles	4.96E-05	53
Rumen	Rice straw	Negative	release of amino acids	5.01E-05	44
Rumen	Rice straw	Negative	metabolism of nucleotide	5.08E-05	173
Rumen	Rice straw	Negative	cell death of hematopoietic progenitor cells	5.27E-05	94
Rumen	Rice straw	Negative	metabolism of sphingolipid	5.63E-05	60
Rumen	Rice straw	Negative	concentration of acylglycerol	5.69E-05	134
Rumen	Rice straw	Negative	infection by HIV-1	5.83E-05	236
Rumen	Rice straw	Negative	cell death of central nervous system cells	5.83E-05	118
Rumen	Rice straw	Negative	transport of glutamine family amino acid	5.92E-05	28
Rumen	Rice straw	Negative	accumulation of fatty acid	5.94E-05	25
Rumen	Rice	Negative	vascularization of body region	5.94E-05	59

	straw				
Rumen	Rice straw	Negative	neural tube defect	5.94E-05	66
Rumen	Rice straw	Negative	arteriosclerosis	5.97E-05	184
Rumen	Rice straw	Negative	apoptosis of neuroblastoma cell lines	6.11E-05	54
Rumen	Rice straw	Negative	degeneration of neurons	6.14E-05	98
Rumen	Rice straw	Negative	cell viability of connective tissue cells	6.28E-05	58
Rumen	Rice straw	Negative	proliferation of endothelial cells	6.32E-05	125
Rumen	Rice straw	Negative	metabolism of glycolipid	6.33E-05	65
Rumen	Rice straw	Negative	development of lymphocytes	6.33E-05	215
Rumen	Rice straw	Negative	autophosphorylation of protein	6.33E-05	68
Rumen	Rice straw	Negative	morphology of gonad	6.33E-05	150
Rumen	Rice straw	Negative	cytopenia	6.38E-05	120
Rumen	Rice straw	Negative	quantity of protein in blood	6.38E-05	198
Rumen	Rice straw	Negative	function of phagocytes	6.49E-05	123
Rumen	Rice straw	Negative	uterine serous papillary cancer	6.66E-05	95
Rumen	Rice straw	Negative	quantity of phagocytes	6.68E-05	167
Rumen	Rice straw	Negative	high bone mass disease	6.71E-05	36
Rumen	Rice straw	Negative	T cell development	6.79E-05	196
Rumen	Rice straw	Negative	abdominal adenocarcinoma	6.80E-05	2075
Rumen	Rice straw	Negative	cell viability of fibroblast cell lines	6.93E-05	51
Rumen	Rice straw	Negative	activation of leukocytes	6.94E-05	268
Rumen	Rice straw	Negative	concentration of cholesterol	7.17E-05	117
Rumen	Rice	Negative	formation of thymus gland	7.19E-05	69

	straw				
Rumen	Rice straw	Negative	cell death of cerebral cortex cells	7.19E-05	94
Rumen	Rice straw	Negative	cell death of tumor	7.31E-05	151
Rumen	Rice straw	Negative	morphogenesis of neurons	7.43E-05	167
Rumen	Rice straw	Negative	atherosclerosis	7.54E-05	181
Rumen	Rice straw	Negative	production of reactive oxygen species	7.56E-05	145
Rumen	Rice straw	Negative	apoptosis of embryonic cell lines	7.64E-05	76
Rumen	Rice straw	Negative	development of mononuclear leukocytes	7.66E-05	216
Rumen	Rice straw	Negative	vaso-occlusion	7.68E-05	187
Rumen	Rice straw	Negative	morphology of epithelial cells	7.78E-05	93
Rumen	Rice straw	Negative	apoptosis of hematopoietic cells	7.78E-05	89
Rumen	Rice straw	Negative	outgrowth of cells	7.98E-05	170
Rumen	Rice straw	Negative	cell movement of kidney cell lines	8.07E-05	61
Rumen	Rice straw	Negative	formation of muscle cells	8.23E-05	75
Rumen	Rice straw	Negative	arrest in proliferation of cells	8.49E-05	88
Rumen	Rice straw	Negative	quantity of steroid	8.52E-05	182
Rumen	Rice straw	Negative	accumulation of sterol	8.58E-05	33
Rumen	Rice straw	Negative	intraepithelial neoplasia	8.58E-05	60
Rumen	Rice straw	Negative	quantity of interleukin	8.58E-05	60
Rumen	Rice straw	Negative	proliferation of lymphatic system cells	9.05E-05	83
Rumen	Rice straw	Negative	occlusion of artery	9.05E-05	185
Rumen	Rice straw	Negative	abnormal morphology of lung	9.19E-05	76
Rumen	Rice	Negative	proliferation of mesenchymal cells	9.22E-05	42

	straw				
Rumen	Rice straw	Negative	abnormal morphology of gonad	9.22E-05	144
Rumen	Rice straw	Negative	allergy	9.24E-05	159
Rumen	Rice straw	Negative	recruitment of cells	9.32E-05	141
Rumen	Rice straw	Negative	size of embryo	9.32E-05	141
Rumen	Rice straw	Negative	progression of tumor	9.33E-05	68
Rumen	Rice straw	Negative	serine phosphorylation of peptide	9.46E-05	39
Rumen	Rice straw	Negative	colon cancer	9.52E-05	1316
Rumen	Rice straw	Negative	influx of cation	9.54E-05	80
Rumen	Rice straw	Negative	T cell homeostasis	9.59E-05	200
Rumen	Rice straw	Negative	neovascularization of organ	9.59E-05	49
Rumen	Rice straw	Negative	G1 phase	9.64E-05	153
Rumen	Rice straw	Negative	quantity of thymocytes	9.72E-05	94
Rumen	Rice straw	Negative	morphology of lymphoid organ	9.91E-05	154
Rumen	Rice straw	Negative	transport of cation	9.91E-05	158
Rumen	Rice straw	Negative	apoptosis of liver	9.91E-05	67
Rumen	Rice straw	Negative	transport of monosaccharide	9.91E-05	67
Rumen	Rice straw	Negative	occlusion of blood vessel	1.02E-04	187
Rumen	Rice straw	Negative	size of cells	1.03E-04	168
Rumen	Rice straw	Negative	flux of ion	1.03E-04	128
Rumen	Rice straw	Negative	colon tumor	1.04E-04	1323
Rumen	Rice straw	Negative	apoptosis of leukocytes	1.04E-04	178
Rumen	Rice	Negative	neoplasia of colon	1.05E-04	1319

	straw				
Rumen	Rice straw	Negative	proliferation of bone marrow cells	1.06E-04	52
Rumen	Rice straw	Negative	long-term potentiation of synapse	1.06E-04	63
Rumen	Rice straw	Negative	migration of smooth muscle cells	1.06E-04	63
Rumen	Rice straw	Negative	transport of metal	1.06E-04	132
Rumen	Rice straw	Negative	modification of palmitic acid	1.07E-04	26
Rumen	Rice straw	Negative	accumulation of cells	1.08E-04	139
Rumen	Rice straw	Negative	activation of blood cells	1.09E-04	284
Rumen	Rice straw	Negative	colorectal neoplasia	1.09E-04	1537
Rumen	Rice straw	Negative	serous neoplasm	1.09E-04	234
Rumen	Rice straw	Negative	quantity of muscle cells	1.10E-04	51
Rumen	Rice straw	Negative	abnormal morphology of axial skeleton	1.11E-04	80
Rumen	Rice straw	Negative	quantity of metal	1.12E-04	193
Rumen	Corn stover	Positive	proliferation of cells	6.36E-51	1545
Rumen	Corn stover	Positive	morbidity or mortality	2.78E-41	1057
Rumen	Corn stover	Positive	organismal death	3.69E-41	1044
Rumen	Corn stover	Positive	cancer	1.75E-38	3805
Rumen	Corn stover	Positive	cell death	6.71E-38	1364
Rumen	Corn stover	Positive	malignant solid tumor	3.33E-37	3759
Rumen	Corn stover	Positive	necrosis	5.93E-33	1073
Rumen	Corn stover	Positive	organization of cytoplasm	2.20E-32	692
Rumen	Corn stover	Positive	apoptosis	2.39E-32	1095
Rumen	Corn	Positive	cell movement	1.77E-29	899

	stover				
Rumen	Corn stover	Positive	organization of cytoskeleton	2.34E-29	629
Rumen	Corn stover	Positive	morphology of cells	1.05E-26	821
Rumen	Corn stover	Positive	tumorigenesis of tissue	8.05E-25	3169
Rumen	Corn stover	Positive	neoplasia of epithelial tissue	7.91E-24	3116
Rumen	Corn stover	Positive	migration of cells	2.09E-23	791
Rumen	Corn stover	Positive	epithelial cancer	2.36E-23	3086
Rumen	Corn stover	Positive	abdominal neoplasm	3.68E-22	3084
Rumen	Corn stover	Positive	expression of RNA	8.02E-22	870
Rumen	Corn stover	Positive	microtubule dynamics	2.47E-21	519
Rumen	Corn stover	Positive	digestive organ tumor	1.79E-20	2683
Rumen	Corn stover	Positive	abdominal cancer	1.80E-20	3040
Rumen	Corn stover	Positive	quantity of cells	2.25E-20	728
Rumen	Corn stover	Positive	digestive system cancer	2.25E-20	2659
Rumen	Corn stover	Positive	cellular homeostasis	6.13E-20	637
Rumen	Corn stover	Positive	transcription	1.80E-19	804
Rumen	Corn stover	Positive	cell death of tumor cell lines	5.72E-19	634
Rumen	Corn stover	Positive	transcription of RNA	3.15E-18	749
Rumen	Corn stover	Positive	transport of molecule	1.59E-17	658
Rumen	Corn stover	Positive	differentiation of cells	6.10E-17	880
Rumen	Corn stover	Positive	cell survival	7.37E-17	576
Rumen	Corn stover	Positive	quantity of blood cells	8.83E-17	442
Rumen	Corn	Positive	cell viability	8.83E-17	542

	stover				
Rumen	Corn stover	Positive	quantity of leukocytes	1.67E-16	399
Rumen	Corn stover	Positive	Viral Infection	2.46E-16	652
Rumen	Corn stover	Positive	apoptosis of tumor cell lines	3.03E-16	507
Rumen	Corn stover	Positive	angiogenesis	3.85E-16	394
Rumen	Corn stover	Positive	formation of cellular protrusions	4.79E-16	392
Rumen	Corn stover	Positive	cell movement of tumor cell lines	6.88E-16	372
Rumen	Corn stover	Positive	vasculogenesis	1.30E-15	327
Rumen	Corn stover	Positive	size of body	1.08E-14	367
Rumen	Corn stover	Positive	morphology of body cavity	1.63E-14	493
Rumen	Corn stover	Positive	morphology of cardiovascular system	2.40E-14	277
Rumen	Corn stover	Positive	abnormal morphology of cells	2.99E-14	517
Rumen	Corn stover	Positive	proliferation of tumor cell lines	4.57E-14	617
Rumen	Corn stover	Positive	development of body trunk	9.52E-14	461
Rumen	Corn stover	Positive	abnormal morphology of body cavity	1.01E-13	470
Rumen	Corn stover	Positive	metabolism of carbohydrate	2.22E-13	274
Rumen	Corn stover	Positive	Movement Disorders	1.12E-12	435
Rumen	Corn stover	Positive	abnormal morphology of thoracic cavity	1.20E-12	258
Rumen	Corn stover	Positive	transcription of DNA	1.58E-12	604
Rumen	Corn stover	Positive	formation of cells	2.11E-12	406
Rumen	Corn stover	Positive	invasion of cells	2.30E-12	364
Rumen	Corn stover	Positive	function of blood cells	4.01E-12	256
Rumen	Corn	Positive	protein kinase cascade	5.61E-12	191

	stover				
Rumen	Corn stover	Positive	migration of tumor cell lines	6.33E-12	301
Rumen	Corn stover	Positive	homing of cells	6.56E-12	244
Rumen	Corn stover	Positive	function of leukocytes	6.78E-12	236
Rumen	Corn stover	Positive	homing	7.46E-12	250
Rumen	Corn stover	Positive	development of vasculature	9.79E-12	189
Rumen	Corn stover	Positive	cell movement of blood cells	1.01E-11	363
Rumen	Corn stover	Positive	migration of blood cells	1.11E-11	362
Rumen	Corn stover	Positive	leukocyte migration	1.41E-11	361
Rumen	Corn stover	Positive	cell death of blood cells	1.72E-11	294
Rumen	Corn stover	Positive	concentration of lipid	1.74E-11	338
Rumen	Corn stover	Positive	abnormal morphology of cardiovascular system	1.79E-11	246
Rumen	Corn stover	Positive	cell movement of leukocytes	2.30E-11	321
Rumen	Corn stover	Positive	quantity of lymphocytes	3.35E-11	298
Rumen	Corn stover	Positive	phosphorylation of protein	4.37E-11	308
Rumen	Corn stover	Positive	cell cycle progression	5.05E-11	422
Rumen	Corn stover	Positive	proliferation of neuronal cells	6.69E-11	247
Rumen	Corn stover	Positive	quantity of mononuclear leukocytes	7.23E-11	307
Rumen	Corn stover	Positive	organization of organelle	1.19E-10	232
Rumen	Corn stover	Positive	cell viability of tumor cell lines	1.30E-10	322
Rumen	Corn stover	Positive	degeneration of nervous system	1.55E-10	133
Rumen	Corn stover	Positive	transactivation	1.87E-10	246
Rumen	Corn	Positive	perinatal death	2.01E-10	252

	stover				
Rumen	Corn stover	Positive	activation of cells	2.03E-10	394
Rumen	Corn stover	Positive	cell movement of endothelial cells	2.06E-10	159
Rumen	Corn stover	Positive	growth of epithelial tissue	2.29E-10	284
Rumen	Corn stover	Positive	ubiquitination of protein	2.72E-10	150
Rumen	Corn stover	Positive	ubiquitination	2.72E-10	152
Rumen	Corn stover	Positive	Growth Failure	4.76E-10	252
Rumen	Corn stover	Positive	growth of organism	5.65E-10	340
Rumen	Corn stover	Positive	cell movement of myeloid cells	7.61E-10	229
Rumen	Corn stover	Positive	cell death of immune cells	7.84E-10	274
Rumen	Corn stover	Positive	morphology of nervous system	7.84E-10	320
Rumen	Corn stover	Positive	development of cytoplasm	8.35E-10	199
Rumen	Corn stover	Positive	development of lymphatic system	9.09E-10	158
Rumen	Corn stover	Positive	proliferation of blood cells	9.97E-10	341
Rumen	Corn stover	Positive	interphase	1.09E-09	272
Rumen	Corn stover	Positive	Neurodegeneration	1.22E-09	139
Rumen	Corn stover	Positive	abnormal morphology of embryonic tissue	1.23E-09	231
Rumen	Corn stover	Positive	invasion of tumor cell lines	1.31E-09	275
Rumen	Corn stover	Positive	development of epithelial tissue	1.35E-09	219
Rumen	Corn stover	Positive	cell death of cervical cancer cell lines	1.44E-09	155
Rumen	Corn stover	Positive	synthesis of lipid	1.44E-09	293
Rumen	Corn stover	Positive	development of blood cells	1.47E-09	270
Rumen	Corn	Positive	growth of tumor	1.55E-09	323

	stover				
Rumen	Corn stover	Positive	morphology of head	1.62E-09	352
Rumen	Corn stover	Positive	abnormal morphology of head	1.77E-09	336
Rumen	Corn stover	Positive	chemotaxis	1.92E-09	229
Rumen	Corn stover	Positive	infection of cells	1.92E-09	323
Rumen	Corn stover	Positive	infection by Retroviridae	2.04E-09	294
Rumen	Corn stover	Positive	organization of actin cytoskeleton	2.04E-09	135
Rumen	Corn stover	Positive	chemotaxis of cells	2.25E-09	222
Rumen	Corn stover	Positive	formation of cytoskeleton	2.38E-09	164
Rumen	Corn stover	Positive	development of leukocytes	2.39E-09	245
Rumen	Corn stover	Positive	cell movement of phagocytes	2.57E-09	230
Rumen	Corn stover	Positive	cell spreading	2.72E-09	125
Rumen	Corn stover	Positive	infection by RNA virus	3.23E-09	347
Rumen	Corn stover	Positive	dysmyelination	3.55E-09	68
Rumen	Corn stover	Positive	benign neoplasia	3.56E-09	368
Rumen	Corn stover	Positive	accumulation of cells	3.81E-09	154
Rumen	Corn stover	Positive	hepatobiliary system cancer	4.47E-09	1541
Rumen	Corn stover	Positive	development of connective tissue	4.57E-09	161
Rumen	Corn stover	Positive	infection by lentivirus	5.56E-09	290
Rumen	Corn stover	Positive	HIV infection	6.17E-09	289
Rumen	Corn stover	Positive	growth of plasma membrane projections	6.18E-09	197
Rumen	Corn stover	Positive	transactivation of RNA	6.84E-09	226
Rumen	Corn	Positive	differentiation of leukocytes	6.84E-09	274

	stover				
Rumen	Corn stover	Positive	liver tumor	7.10E-09	1542
Rumen	Corn stover	Positive	formation of lymphoid organ	7.21E-09	119
Rumen	Corn stover	Positive	growth of neurites	7.45E-09	195
Rumen	Corn stover	Positive	outgrowth of plasma membrane projections	7.45E-09	174
Rumen	Corn stover	Positive	liver cancer	7.45E-09	1532
Rumen	Corn stover	Positive	hepatocellular carcinoma	7.56E-09	1486
Rumen	Corn stover	Positive	development of neurons	7.91E-09	314
Rumen	Corn stover	Positive	development of abdomen	8.41E-09	239
Rumen	Corn stover	Positive	development of lymphatic system component	8.69E-09	136
Rumen	Corn stover	Positive	abnormal morphology of abdomen	8.81E-09	348
Rumen	Corn stover	Positive	demyelination	9.62E-09	61
Rumen	Corn stover	Positive	development of cardiovascular tissue	1.08E-08	159
Rumen	Corn stover	Positive	Organ Degeneration	1.14E-08	206
Rumen	Corn stover	Positive	cell death of connective tissue cells	1.14E-08	247
Rumen	Corn stover	Positive	formation of lymphatic system component	1.14E-08	129
Rumen	Corn stover	Positive	cell movement of neutrophils	1.14E-08	131
Rumen	Corn stover	Positive	abnormal morphology of nervous system	1.19E-08	292
Rumen	Corn stover	Positive	outgrowth of neurites	1.22E-08	172
Rumen	Corn stover	Positive	cell movement of granulocytes	1.24E-08	159
Rumen	Corn stover	Positive	differentiation of blood cells	1.36E-08	335
Rumen	Corn stover	Positive	colony formation of cells	1.43E-08	205
Rumen	Corn	Positive	accumulation of lipid	1.69E-08	131

	stover				
Rumen	Corn stover	Positive	autophagy	1.88E-08	160
Rumen	Corn stover	Positive	survival of organism	1.88E-08	283
Rumen	Corn stover	Positive	neuronal cell death	1.89E-08	275
Rumen	Corn stover	Positive	autosomal recessive disease	2.00E-08	362
Rumen	Corn stover	Positive	proliferation of connective tissue cells	2.15E-08	242
Rumen	Corn stover	Positive	accumulation of blood cells	2.47E-08	123
Rumen	Corn stover	Positive	concentration of fatty acid	2.47E-08	123
Rumen	Corn stover	Positive	development of hematopoietic system	2.70E-08	111
Rumen	Corn stover	Positive	cellular degradation	2.78E-08	113
Rumen	Corn stover	Positive	replication of RNA virus	2.88E-08	209
Rumen	Corn stover	Positive	morphology of heart	2.99E-08	171
Rumen	Corn stover	Positive	disorder of basal ganglia	3.25E-08	305
Rumen	Corn stover	Positive	fibrogenesis	3.31E-08	173
Rumen	Corn stover	Positive	colony formation	3.49E-08	220
Rumen	Corn stover	Positive	migration of endothelial cells	3.95E-08	141
Rumen	Corn stover	Positive	development of lymphocytes	4.09E-08	226
Rumen	Corn stover	Positive	necrosis of epithelial tissue	4.26E-08	239
Rumen	Corn stover	Positive	apoptosis of blood cells	4.90E-08	204
Rumen	Corn stover	Positive	growth of embryo	5.10E-08	197
Rumen	Corn stover	Positive	development of endothelial tissue	5.16E-08	155
Rumen	Corn stover	Positive	proliferation of immune cells	5.19E-08	313
Rumen	Corn	Positive	development of mononuclear leukocytes	5.38E-08	227

	stover				
Rumen	Corn stover	Positive	mitosis	5.53E-08	204
Rumen	Corn stover	Positive	infection by HIV-1	6.20E-08	246
Rumen	Corn stover	Positive	synthesis of carbohydrate	6.28E-08	177
Rumen	Corn stover	Positive	cell death of epithelial cells	6.31E-08	204
Rumen	Corn stover	Positive	degeneration of neurons	6.42E-08	106
Rumen	Corn stover	Positive	T cell development	6.67E-08	206
Rumen	Corn stover	Positive	neuromuscular disease	6.84E-08	345
Rumen	Corn stover	Positive	proliferation of hematopoietic cells	6.86E-08	112
Rumen	Corn stover	Positive	outgrowth of cells	7.00E-08	180
Rumen	Corn stover	Positive	neonatal death	7.63E-08	182
Rumen	Corn stover	Positive	cell movement of fibroblasts	7.67E-08	84
Rumen	Corn stover	Positive	degeneration of cells	7.68E-08	146
Rumen	Corn stover	Positive	formation of filaments	9.20E-08	166
Rumen	Corn stover	Positive	Lymphocyte homeostasis	9.88E-08	221
Rumen	Corn stover	Positive	neuritogenesis	1.03E-07	235
Rumen	Corn stover	Positive	metabolism of protein	1.05E-07	376
Rumen	Corn stover	Positive	neurological signs	1.06E-07	260
Rumen	Corn stover	Positive	endothelial cell development	1.10E-07	149
Rumen	Corn stover	Positive	uptake of carbohydrate	1.11E-07	125
Rumen	Corn stover	Positive	dyskinesia	1.25E-07	247
Rumen	Corn stover	Positive	growth of connective tissue	1.28E-07	257
Rumen	Corn	Positive	morphology of connective tissue	1.30E-07	189

	stover				
Rumen	Corn stover	Positive	homeostasis of leukocytes	1.33E-07	224
Rumen	Corn stover	Positive	formation of blood cells	1.40E-07	89
Rumen	Corn stover	Positive	development of body axis	1.44E-07	403
Rumen	Corn stover	Positive	replication of virus	1.58E-07	226
Rumen	Corn stover	Positive	proliferation of lymphatic system cells	1.63E-07	90
Rumen	Corn stover	Positive	T cell homeostasis	1.79E-07	209
Rumen	Corn stover	Positive	proliferation of hematopoietic progenitor cells	1.81E-07	106
Rumen	Corn stover	Positive	formation of thymus gland	1.83E-07	75
Rumen	Corn stover	Positive	quantity of T lymphocytes	1.84E-07	216
Rumen	Corn stover	Positive	uptake of monosaccharide	1.84E-07	116
Rumen	Corn stover	Positive	fatty acid metabolism	1.98E-07	240
Rumen	Corn stover	Positive	cell movement of connective tissue cells	1.98E-07	98
Rumen	Corn stover	Positive	activation of DNA endogenous promoter	2.03E-07	457
Rumen	Corn stover	Positive	proliferation of mononuclear leukocytes	2.07E-07	290
Rumen	Corn stover	Positive	cell transformation	2.14E-07	188
Rumen	Corn stover	Positive	chorea	2.14E-07	231
Rumen	Corn stover	Positive	abnormal morphology of heart	2.14E-07	159
Rumen	Corn stover	Positive	I-kappaB kinase/NF-kappaB cascade	2.30E-07	79
Rumen	Corn stover	Positive	morphology of vessel	2.30E-07	130
Rumen	Corn stover	Positive	vascularization	2.32E-07	103
Rumen	Corn stover	Positive	Huntington's Disease	2.34E-07	230
Rumen	Corn	Positive	formation of plasma membrane projections	2.36E-07	238

	stover				
Rumen	Corn stover	Positive	binding of DNA	2.58E-07	210
Rumen	Corn stover	Positive	formation of leukocytes	2.63E-07	73
Rumen	Corn stover	Positive	S phase	3.28E-07	112
Rumen	Corn stover	Positive	female genital tract serous cancer	3.40E-07	159
Rumen	Corn stover	Positive	congenital anomaly of musculoskeletal system	3.40E-07	288
Rumen	Corn stover	Positive	morphology of blood vessel	3.76E-07	120
Rumen	Corn stover	Positive	apoptosis of connective tissue cells	3.87E-07	125
Rumen	Corn stover	Positive	apoptosis of pheochromocytoma cell lines	4.21E-07	46
Rumen	Corn stover	Positive	development of head	4.21E-07	375
Rumen	Corn stover	Positive	proliferation of lymphocytes	4.57E-07	284
Rumen	Corn stover	Positive	small GTPase mediated signal transduction	4.99E-07	82
Rumen	Corn stover	Positive	migration of myeloid cells	5.20E-07	75
Rumen	Corn stover	Positive	midline defect	5.27E-07	113
Rumen	Corn stover	Positive	proliferation of epithelial cells	5.47E-07	198
Rumen	Corn stover	Positive	arrest in interphase	8.10E-07	169
Rumen	Corn stover	Positive	cell death of T lymphocytes	8.52E-07	131
Rumen	Corn stover	Positive	epileptic seizure	8.92E-07	75
Rumen	Corn stover	Positive	inflammatory response	8.92E-07	301
Rumen	Corn stover	Positive	mass of organism	9.20E-07	138
Rumen	Corn stover	Positive	accumulation of leukocytes	9.46E-07	113
Rumen	Corn stover	Positive	size of animal	1.06E-06	79
Rumen	Corn	Positive	apoptosis of cervical cancer cell lines	1.18E-06	118

	stover				
Rumen	Corn stover	Positive	recruitment of cells	1.19E-06	146
Rumen	Corn stover	Positive	cell death of mononuclear leukocytes	1.23E-06	161
Rumen	Corn stover	Positive	quantity of hematopoietic cells	1.23E-06	178
Rumen	Corn stover	Positive	proliferation of endothelial cells	1.23E-06	129
Rumen	Corn stover	Positive	autosomal dominant disease	1.34E-06	249
Rumen	Corn stover	Positive	cell death of epithelial cell lines	1.40E-06	118
Rumen	Corn stover	Positive	synthesis of fatty acid	1.46E-06	129
Rumen	Corn stover	Positive	abnormal morphology of reproductive system	1.46E-06	202
Rumen	Corn stover	Positive	quantity of hematopoietic progenitor cells	1.56E-06	177
Rumen	Corn stover	Positive	migration of phagocytes	1.59E-06	115
Rumen	Corn stover	Positive	cell death of pheochromocytoma cell lines	1.63E-06	56
Rumen	Corn stover	Positive	arthropathy	1.68E-06	361
Rumen	Corn stover	Positive	proliferation of fibroblasts	1.68E-06	144
Rumen	Corn stover	Positive	peripheral arterial disease	1.72E-06	91
Rumen	Corn stover	Positive	proliferation of embryonic cells	1.75E-06	105
Rumen	Corn stover	Positive	long-term potentiation	1.76E-06	103
Rumen	Corn stover	Positive	activation of enzyme	1.84E-06	157
Rumen	Corn stover	Positive	seizures	1.84E-06	161
Rumen	Corn stover	Positive	proliferation of fibroblast cell lines	1.86E-06	170
Rumen	Corn stover	Positive	morphology of reproductive system	1.87E-06	211
Rumen	Corn stover	Positive	abnormal morphology of epithelial tissue	2.00E-06	168
Rumen	Corn	Positive	migration of connective tissue cells	2.10E-06	79

	stover				
Rumen	Corn stover	Positive	Bleeding	2.12E-06	170
Rumen	Corn stover	Positive	morphology of lymphatic system component	2.22E-06	167
Rumen	Corn stover	Positive	function of phagocytes	2.29E-06	126
Rumen	Corn stover	Positive	reorganization of cytoskeleton	2.31E-06	83
Rumen	Corn stover	Positive	formation of T lymphocytes	2.35E-06	54
Rumen	Corn stover	Positive	cell movement of antigen presenting cells	2.36E-06	142
Rumen	Corn stover	Positive	quantity of connective tissue	2.45E-06	235
Rumen	Corn stover	Positive	metabolism of membrane lipid derivative	2.67E-06	156
Rumen	Corn stover	Positive	intermediate disease stage peripheral arterial disease	2.69E-06	70
Rumen	Corn stover	Positive	organization of filaments	2.76E-06	88
Rumen	Corn stover	Positive	growth of lymphatic system component	2.86E-06	83
Rumen	Corn stover	Positive	cell death of lymphocytes	2.96E-06	155
Rumen	Corn stover	Positive	Cytosis	2.98E-06	131
Rumen	Corn stover	Positive	proliferation of bone marrow cells	3.05E-06	55
Rumen	Corn stover	Positive	muscle tumor	3.23E-06	146
Rumen	Corn stover	Positive	blood protein disorder	3.39E-06	151
Rumen	Corn stover	Positive	formation of hematopoietic progenitor cells	3.54E-06	58
Rumen	Corn stover	Positive	connective or soft tissue tumor	3.55E-06	271
Rumen	Corn stover	Positive	activation of blood cells	3.57E-06	286
Rumen	Corn stover	Positive	arthritis	3.63E-06	353
Rumen	Corn stover	Positive	metabolism of monosaccharide	3.92E-06	56
Rumen	Corn	Positive	homing of blood cells	4.03E-06	152

	stover				
Rumen	Corn stover	Positive	concentration of phospholipid	4.07E-06	79
Rumen	Corn stover	Positive	behavior	4.08E-06	371
Rumen	Corn stover	Positive	migration of granulocytes	4.12E-06	64
Rumen	Corn stover	Positive	cell death of tumor	4.12E-06	153
Rumen	Corn stover	Positive	morphology of bone	4.25E-06	178
Rumen	Corn stover	Positive	abnormal morphology of bone	4.29E-06	173
Rumen	Corn stover	Positive	formation of mononuclear leukocytes	4.34E-06	59
Rumen	Corn stover	Positive	morphology of central nervous system	4.46E-06	201
Rumen	Corn stover	Positive	proliferation of T lymphocytes	4.49E-06	234
Rumen	Corn stover	Positive	engulfment of cells	4.73E-06	162
Rumen	Corn stover	Positive	formation of lymphocytes	4.87E-06	57
Rumen	Corn stover	Positive	necrosis of tumor	5.23E-06	152
Rumen	Corn stover	Positive	function of cardiovascular system	5.23E-06	148
Rumen	Corn stover	Positive	movement of vascular endothelial cells	5.49E-06	78
Rumen	Corn stover	Positive	cell death of fibroblasts	5.63E-06	109
Rumen	Corn stover	Positive	apoptosis of leukocytes	5.63E-06	180
Rumen	Corn stover	Positive	growth of muscle tissue	6.24E-06	149
Rumen	Corn stover	Positive	recruitment of leukocytes	6.31E-06	131
Rumen	Corn stover	Positive	quantity of carbohydrate	6.35E-06	202
Rumen	Corn stover	Positive	chemotaxis of myeloid cells	6.35E-06	119
Rumen	Corn stover	Positive	differentiation of T lymphocytes	6.62E-06	151
Rumen	Corn	Positive	chemotaxis of phagocytes	6.71E-06	120

	stover				
Rumen	Corn stover	Positive	ruffling	6.77E-06	50
Rumen	Corn stover	Positive	differentiation of mononuclear leukocytes	6.93E-06	210
Rumen	Corn stover	Positive	breast or colorectal cancer	7.23E-06	1711
Rumen	Corn stover	Positive	homing of leukocytes	7.30E-06	150
Rumen	Corn stover	Positive	abnormal morphology of central nervous system	7.39E-06	189
Rumen	Corn stover	Positive	entrance of Ca2+	7.44E-06	38
Rumen	Corn stover	Positive	function of mononuclear leukocytes	8.03E-06	137
Rumen	Corn stover	Positive	uptake of D-hexose	8.03E-06	93
Rumen	Corn stover	Positive	quantity of macrophages	8.03E-06	85
Rumen	Corn stover	Positive	neurotransmission	8.03E-06	163
Rumen	Corn stover	Positive	quantity of myeloid cells	8.36E-06	142
Rumen	Corn stover	Positive	transport of protein	8.61E-06	122
Rumen	Corn stover	Positive	branching of cells	8.61E-06	147
Rumen	Corn stover	Positive	development of digestive system	8.73E-06	152
Rumen	Corn stover	Positive	cell viability of cervical cancer cell lines	8.73E-06	92
Rumen	Corn stover	Positive	uptake of D-glucose	8.73E-06	92
Rumen	Corn stover	Positive	quantity of phagocytes	8.73E-06	167
Rumen	Corn stover	Positive	formation of muscle	8.76E-06	157
Rumen	Corn stover	Positive	concentration of phosphatidic acid	8.86E-06	60
Rumen	Corn stover	Positive	growth of embryonic tissue	9.23E-06	112
Rumen	Corn stover	Positive	differentiation of connective tissue cells	9.32E-06	246
Rumen	Corn	Positive	apoptosis of epithelial cell lines	9.45E-06	93

	stover				
Rumen	Corn stover	Positive	cell movement of lymphocytes	9.67E-06	156
Rumen	Corn stover	Positive	morphogenesis of neurons	9.80E-06	167
Rumen	Corn stover	Positive	serous neoplasm	9.80E-06	234
Rumen	Corn stover	Positive	recruitment of blood cells	9.80E-06	132
Rumen	Corn stover	Positive	autophagy of cells	9.90E-06	108
Rumen	Corn stover	Positive	function of lymphocytes	9.90E-06	136
Rumen	Corn stover	Positive	infection of embryonic cell lines	1.07E-05	112
Rumen	Corn stover	Positive	infection of epithelial cell lines	1.07E-05	112
Rumen	Corn stover	Positive	congenital malformation of skeleton	1.07E-05	166
Rumen	Corn stover	Positive	death of perinatal stage organism	1.08E-05	39
Rumen	Corn stover	Positive	cell death of stem cells	1.09E-05	40
Rumen	Corn stover	Positive	proliferation of muscle cells	1.09E-05	147
Rumen	Corn stover	Positive	benign neoplasm of female genital organ	1.09E-05	167
Rumen	Corn stover	Positive	proliferation of tumor cells	1.15E-05	183
Rumen	Corn stover	Positive	seizure disorder	1.15E-05	183
Rumen	Corn stover	Positive	abnormal morphology of blood vessel	1.15E-05	108
Rumen	Corn stover	Positive	homing of endothelial cells	1.15E-05	32
Rumen	Corn stover	Positive	differentiation of lymphocytes	1.17E-05	193
Rumen	Corn stover	Positive	paraproteinemia	1.20E-05	134
Rumen	Corn stover	Positive	skin abnormality	1.20E-05	94
Rumen	Corn stover	Positive	breast or ovarian cancer	1.20E-05	684
Rumen	Corn	Positive	cell movement of mononuclear leukocytes	1.21E-05	180

	stover				
Rumen	Corn stover	Positive	cell death of tumor cells	1.22E-05	147
Rumen	Corn stover	Positive	quantity of antigen presenting cells	1.22E-05	112
Rumen	Corn stover	Positive	synthesis of nitric oxide	1.22E-05	112
Rumen	Corn stover	Positive	formation of thymocytes	1.27E-05	48
Rumen	Corn stover	Positive	quantity of lymphatic system component	1.29E-05	149
Rumen	Corn stover	Positive	differentiation of connective tissue	1.29E-05	276
Rumen	Corn stover	Positive	formation of skin	1.30E-05	145
Rumen	Corn stover	Positive	craniofacial abnormality	1.31E-05	141
Rumen	Corn stover	Positive	Gastrointestinal Tract Cancer and Tumors	1.34E-05	1764
Rumen	Corn stover	Positive	abnormal morphology of lymphoid organ	1.47E-05	149
Rumen	Corn stover	Positive	formation of eye	1.47E-05	171
Rumen	Corn stover	Positive	Hypoplasia	1.47E-05	195
Rumen	Corn stover	Positive	formation of actin filaments	1.48E-05	122
Rumen	Corn stover	Positive	gastrointestinal carcinoma	1.50E-05	1561
Rumen	Corn stover	Positive	quantity of lymphoid organ	1.54E-05	123
Rumen	Corn stover	Positive	smooth muscle tumor	1.64E-05	121
Rumen	Corn stover	Positive	cell death of cerebral cortex cells	1.69E-05	94
Rumen	Corn stover	Positive	cell death of muscle cells	1.73E-05	133
Rumen	Corn stover	Positive	plasma cell dyscrasia	1.73E-05	133
Rumen	Corn stover	Positive	development of sensory organ	1.74E-05	216
Rumen	Corn stover	Positive	formation of lamellipodia	1.74E-05	69
Rumen	Corn	Positive	cell movement of muscle cells	1.75E-05	76

	stover				
Rumen	Corn stover	Positive	adhesion of immune cells	1.77E-05	138
Rumen	Corn stover	Positive	infection of kidney cell lines	1.84E-05	114
Rumen	Corn stover	Positive	length of cells	1.84E-05	46
Rumen	Corn stover	Positive	oral cancer	1.84E-05	93
Rumen	Corn stover	Positive	morphology of respiratory system	1.84E-05	124
Rumen	Corn stover	Positive	cellular infiltration of blood cells	2.03E-05	152
Rumen	Corn stover	Positive	transmigration of cells	2.07E-05	67
Rumen	Corn stover	Positive	morphology of brain	2.11E-05	180
Rumen	Corn stover	Positive	peripheral vascular disease	2.12E-05	149
Rumen	Corn stover	Positive	quantity of IL-6 in blood	2.12E-05	40
Rumen	Corn stover	Positive	morphology of digestive system	2.12E-05	218
Rumen	Corn stover	Positive	genital tumor	2.18E-05	1396
Rumen	Corn stover	Positive	adhesion of tumor cell lines	2.23E-05	115
Rumen	Corn stover	Positive	multiple congenital anomalies	2.30E-05	164
Rumen	Corn stover	Positive	morphology of lymphoid organ	2.34E-05	153
Rumen	Corn stover	Positive	formation of muscle cells	2.34E-05	75
Rumen	Corn stover	Positive	cell movement of macrophages	2.34E-05	107
Rumen	Corn stover	Positive	oral tumor	2.40E-05	120
Rumen	Corn stover	Positive	apoptosis of epithelial cells	2.47E-05	114
Rumen	Corn stover	Positive	synaptic transmission	2.57E-05	133
Rumen	Corn stover	Positive	oral squamous cell carcinoma	2.57E-05	74
Rumen	Corn	Positive	binding of protein binding site	2.58E-05	115

	stover				
Rumen	Corn stover	Positive	adenocarcinoma	2.65E-05	2209
Rumen	Corn stover	Positive	dysgenesis	2.68E-05	206
Rumen	Corn stover	Positive	colony formation of tumor cell lines	2.73E-05	110
Rumen	Corn stover	Positive	metabolism of DNA	2.73E-05	155
Rumen	Corn stover	Positive	Thrombosis	2.73E-05	71
Rumen	Corn stover	Positive	cellular infiltration by leukocytes	2.84E-05	151
Rumen	Corn stover	Positive	degeneration of central nervous system	2.84E-05	57
Rumen	Corn stover	Positive	apoptosis of muscle	2.85E-05	105
Rumen	Corn stover	Positive	gastrointestinal tract cancer	2.86E-05	1732
Rumen	Corn stover	Positive	cell movement of smooth muscle cells	2.88E-05	68
Rumen	Corn stover	Positive	abnormal morphology of brain	2.89E-05	172
Rumen	Corn stover	Positive	adhesion of blood cells	2.89E-05	147
Rumen	Corn stover	Positive	metabolism of hexose	2.97E-05	48
Rumen	Corn stover	Positive	cognition	3.01E-05	175
Rumen	Corn stover	Positive	invasion of tumor	3.03E-05	98
Rumen	Corn stover	Positive	cell death of muscle	3.06E-05	135
Rumen	Corn stover	Positive	cellular infiltration	3.08E-05	169
Rumen	Corn stover	Positive	morphogenesis of neurites	3.08E-05	162
Rumen	Corn stover	Positive	metabolism of polysaccharide	3.13E-05	96
Rumen	Corn stover	Positive	mammary tumor	3.19E-05	587
Rumen	Corn stover	Positive	fertility	3.26E-05	142
Rumen	Corn	Positive	advanced malignant tumor	3.31E-05	314

	stover				
Rumen	Corn stover	Positive	differentiation of muscle cell lines	3.55E-05	68
Rumen	Corn stover	Positive	abdominal adenocarcinoma	3.61E-05	2011
Rumen	Corn stover	Positive	apoptosis of muscle cells	3.66E-05	104
Rumen	Corn stover	Positive	activation of leukocytes	3.68E-05	262
Rumen	Corn stover	Positive	shape change of tumor cell lines	3.71E-05	65
Rumen	Corn stover	Positive	abnormal morphology of respiratory system	3.75E-05	121
Rumen	Corn stover	Positive	recruitment of phagocytes	3.77E-05	99
Rumen	Corn stover	Positive	necrosis of muscle	3.85E-05	134
Rumen	Corn stover	Positive	size of embryo	3.87E-05	139
Rumen	Corn stover	Positive	cell movement of leukemia cell lines	3.87E-05	59
Rumen	Corn stover	Positive	stress response of cells	3.87E-05	59
Rumen	Corn stover	Positive	quantity of B lymphocytes	3.94E-05	135
Rumen	Corn stover	Positive	migration of muscle cells	3.95E-05	69
Rumen	Corn stover	Positive	morphology of cardiovascular tissue	4.01E-05	40
Rumen	Corn stover	Positive	recruitment of macrophages	4.09E-05	47
Rumen	Corn stover	Positive	proliferation of smooth muscle cells	4.11E-05	113
Rumen	Corn stover	Positive	synthesis of polysaccharide	4.11E-05	75
Rumen	Corn stover	Positive	chronic psoriasis	4.11E-05	61
Rumen	Corn stover	Positive	Dermatitis	4.12E-05	167
Rumen	Corn stover	Positive	cell death of hematopoietic cell lines	4.17E-05	93
Rumen	Corn stover	Positive	learning	4.21E-05	161
Rumen	Corn	Positive	apoptosis of T lymphocytes	4.33E-05	111

	stover				
Rumen	Corn stover	Positive	apoptosis of mononuclear leukocytes	4.33E-05	139
Rumen	Corn stover	Positive	development of hematopoietic tissue	4.39E-05	54
Rumen	Corn stover	Positive	cell death of brain	4.44E-05	115
Rumen	Corn stover	Positive	oral cavity carcinoma	4.44E-05	80
Rumen	Corn stover	Positive	quantity of lymphatic system cells	4.57E-05	109
Rumen	Corn stover	Positive	Ovarian Cancer and Tumors	4.63E-05	282
Rumen	Corn stover	Positive	benign connective or soft tissue neoplasm	4.64E-05	142
Rumen	Corn stover	Positive	leiomyomatosis	4.70E-05	113
Rumen	Corn stover	Positive	formation of filopodia	4.77E-05	69
Rumen	Corn stover	Positive	function of antigen presenting cells	4.77E-05	98
Rumen	Corn stover	Positive	growth of skin	4.88E-05	88
Rumen	Corn stover	Positive	cell death of breast cancer cell lines	4.94E-05	130
Rumen	Corn stover	Positive	susceptibility to endotoxin shock	4.94E-05	25
Rumen	Corn stover	Positive	formation of actin stress fibers	4.97E-05	96
Rumen	Corn stover	Positive	chronic myeloproliferative disorder	5.07E-05	66
Rumen	Corn stover	Positive	apoptosis of neurons	5.08E-05	167
Rumen	Corn stover	Positive	chemotaxis of endothelial cells	5.11E-05	30
Rumen	Corn stover	Positive	cellular infiltration of cells	5.11E-05	153
Rumen	Corn stover	Positive	cell death of B-lymphocyte derived cell lines	5.14E-05	55
Rumen	Corn stover	Positive	immune response of cells	5.23E-05	216
Rumen	Corn stover	Positive	apoptosis of cerebral cortex cells	5.25E-05	47
Rumen	Corn	Positive	chronic myeloid leukemia	5.25E-05	47

	stover				
Rumen	Corn stover	Positive	sprouting	5.31E-05	149
Rumen	Corn stover	Positive	cell death of cortical neurons	5.31E-05	72
Rumen	Corn stover	Positive	G1 phase	5.40E-05	150
Rumen	Corn stover	Positive	migration of breast cancer cell lines	5.51E-05	98
Rumen	Corn stover	Positive	morphology of endothelial tissue	5.62E-05	38
Rumen	Corn stover	Positive	grade 1 lymphocytic cancer	5.67E-05	33
Rumen	Corn stover	Positive	synthesis of DNA	5.69E-05	160
Rumen	Corn stover	Positive	metastasis	5.74E-05	282
Rumen	Corn stover	Positive	migration of cancer cells	5.74E-05	79
Rumen	Corn stover	Positive	transport of carbohydrate	5.74E-05	79
Rumen	Corn stover	Positive	cell death of sarcoma cell lines	5.84E-05	91
Rumen	Corn stover	Positive	gonadal tumor	5.85E-05	303
Rumen	Corn stover	Positive	apoptosis of B-lymphocyte derived cell lines	5.87E-05	53
Rumen	Corn stover	Positive	quantity of interleukin	5.87E-05	59
Rumen	Corn stover	Positive	activation of Protein kinase	6.07E-05	100
Rumen	Corn stover	Positive	differentiation of epithelial tissue	6.09E-05	139
Rumen	Corn stover	Positive	secretory pathway	6.21E-05	80
Rumen	Corn stover	Positive	differentiation of tumor cell lines	6.26E-05	146
Rumen	Corn stover	Positive	abnormal morphology of skeleton	6.28E-05	107
Rumen	Corn stover	Positive	function of myeloid cells	6.28E-05	95
Rumen	Corn stover	Positive	accumulation of myeloid cells	6.36E-05	72
Rumen	Corn	Positive	formation of connective tissue cells	6.37E-05	85

	stover				
Rumen	Corn stover	Positive	migration of tumor cells	6.38E-05	90
Rumen	Corn stover	Positive	migration of fibroblasts	6.74E-05	60
Rumen	Corn stover	Positive	quantity of immunoglobulin	6.78E-05	116
Rumen	Corn stover	Positive	apoptosis of lymphocytes	6.90E-05	134
Rumen	Corn stover	Positive	development of genitourinary system	7.02E-05	326
Rumen	Corn stover	Positive	migration of smooth muscle cells	7.03E-05	62
Rumen	Corn stover	Positive	development of connective tissue cells	7.06E-05	89
Rumen	Corn stover	Positive	morphology of genital organ	7.20E-05	162
Rumen	Corn stover	Positive	cell death of embryonic cells	7.21E-05	50
Rumen	Corn stover	Positive	quantity of thymocytes	7.21E-05	92
Rumen	Corn stover	Positive	abnormal morphology of digestive system	7.22E-05	205
Rumen	Corn stover	Positive	development of bone marrow	7.42E-05	53
Rumen	Corn stover	Positive	hereditary neuropathy	7.57E-05	68
Rumen	Corn stover	Positive	migration of leukemia cell lines	7.81E-05	37
Rumen	Corn stover	Positive	replication of Influenza virus	7.98E-05	113
Rumen	Corn stover	Positive	invasion of tissue	8.14E-05	79
Rumen	Corn stover	Positive	aggregation of cells	8.14E-05	126
Rumen	Corn stover	Positive	neovascularization	8.33E-05	67
Rumen	Corn stover	Positive	cell movement of tumor cells	8.37E-05	75
Rumen	Corn stover	Positive	bone mineral density	8.44E-05	71
Rumen	Corn stover	Positive	uterine serous papillary cancer	8.45E-05	92
Rumen	Corn	Positive	attachment of cells	8.62E-05	57

	stover				
Rumen	Corn stover	Positive	chronic inflammatory disorder	8.64E-05	355
Rumen	Corn stover	Positive	accumulation of granulocytes	8.79E-05	47
Rumen	Corn stover	Positive	proliferation of cancer cells	8.84E-05	140
Rumen	Corn stover	Positive	entry into S phase	9.06E-05	59
Rumen	Corn stover	Positive	generation of lymphocytes	9.06E-05	59
Rumen	Corn stover	Positive	formation of lung	9.12E-05	117
Rumen	Corn stover	Negative	organismal death	2.27E-27	650
Rumen	Corn stover	Negative	proliferation of cells	2.27E-27	926
Rumen	Corn stover	Negative	morbidity or mortality	7.15E-27	654
Rumen	Corn stover	Negative	morphology of cells	3.69E-22	529
Rumen	Corn stover	Negative	cell death	2.28E-21	823
Rumen	Corn stover	Negative	malignant solid tumor	2.66E-20	2245
Rumen	Corn stover	Negative	necrosis	3.23E-20	656
Rumen	Corn stover	Negative	cancer	3.42E-20	2268
Rumen	Corn stover	Negative	apoptosis	5.31E-19	665
Rumen	Corn stover	Negative	cell movement	6.97E-19	554
Rumen	Corn stover	Negative	cell survival	6.42E-17	383
Rumen	Corn stover	Negative	organization of cytoplasm	7.28E-17	414
Rumen	Corn stover	Negative	quantity of cells	2.47E-16	464
Rumen	Corn stover	Negative	transport of molecule	1.51E-15	426
Rumen	Corn stover	Negative	cell viability	1.67E-15	356
Rumen	Corn	Negative	migration of cells	3.17E-15	488

	stover				
Rumen	Corn stover	Negative	organization of cytoskeleton	3.17E-15	376
Rumen	Corn stover	Negative	transcription	7.33E-15	506
Rumen	Corn stover	Negative	expression of RNA	1.46E-14	537
Rumen	Corn stover	Negative	transcription of RNA	3.39E-14	473
Rumen	Corn stover	Negative	vasculogenesis	1.53E-13	217
Rumen	Corn stover	Negative	cell movement of tumor cell lines	2.08E-13	244
Rumen	Corn stover	Negative	development of vasculature	3.68E-13	136
Rumen	Corn stover	Negative	morphology of cardiovascular system	7.31E-13	186
Rumen	Corn stover	Negative	proliferation of tumor cell lines	7.51E-13	399
Rumen	Corn stover	Negative	tumorigenesis of tissue	7.82E-13	1888
Rumen	Corn stover	Negative	transcription of DNA	1.30E-12	395
Rumen	Corn stover	Negative	invasion of cells	1.37E-12	245
Rumen	Corn stover	Negative	abnormal morphology of cells	1.42E-12	335
Rumen	Corn stover	Negative	proliferation of connective tissue cells	1.66E-12	178
Rumen	Corn stover	Negative	cell death of tumor cell lines	1.74E-12	392
Rumen	Corn stover	Negative	neoplasia of epithelial tissue	2.61E-12	1856
Rumen	Corn stover	Negative	angiogenesis	6.08E-12	251
Rumen	Corn stover	Negative	growth of connective tissue	9.27E-12	188
Rumen	Corn stover	Negative	microtubule dynamics	9.27E-12	313
Rumen	Corn stover	Negative	abnormal morphology of cardiovascular system	1.07E-11	169
Rumen	Corn stover	Negative	epithelial cancer	1.26E-11	1835
Rumen	Corn	Negative	differentiation of cells	2.12E-11	541

	stover				
Rumen	Corn stover	Negative	apoptosis of tumor cell lines	2.54E-11	317
Rumen	Corn stover	Negative	perinatal death	6.08E-11	173
Rumen	Corn stover	Negative	cellular homeostasis	1.69E-10	381
Rumen	Corn stover	Negative	morphology of body cavity	2.32E-10	309
Rumen	Corn stover	Negative	abnormal morphology of thoracic cavity	2.50E-10	169
Rumen	Corn stover	Negative	quantity of leukocytes	2.95E-10	246
Rumen	Corn stover	Negative	cell viability of tumor cell lines	3.53E-10	214
Rumen	Corn stover	Negative	quantity of blood cells	5.47E-10	270
Rumen	Corn stover	Negative	formation of cells	5.47E-10	260
Rumen	Corn stover	Negative	Movement Disorders	5.47E-10	277
Rumen	Corn stover	Negative	cell death of connective tissue cells	7.99E-10	171
Rumen	Corn stover	Negative	abdominal neoplasm	9.52E-10	1824
Rumen	Corn stover	Negative	abnormal morphology of body cavity	1.05E-09	294
Rumen	Corn stover	Negative	migration of tumor cell lines	1.12E-09	195
Rumen	Corn stover	Negative	ubiquitination	1.12E-09	105
Rumen	Corn stover	Negative	morphology of heart	1.37E-09	122
Rumen	Corn stover	Negative	ubiquitination of protein	1.99E-09	103
Rumen	Corn stover	Negative	invasion of tumor cell lines	2.32E-09	184
Rumen	Corn stover	Negative	cell transformation	4.32E-09	134
Rumen	Corn stover	Negative	transactivation	5.34E-09	162
Rumen	Corn stover	Negative	colony formation	5.42E-09	152
Rumen	Corn	Negative	digestive organ tumor	6.60E-09	1584

	stover				
Rumen	Corn stover	Negative	proliferation of fibroblast cell lines	7.97E-09	124
Rumen	Corn stover	Negative	abdominal cancer	9.57E-09	1796
Rumen	Corn stover	Negative	formation of cellular protrusions	9.57E-09	236
Rumen	Corn stover	Negative	organization of organelle	1.53E-08	151
Rumen	Corn stover	Negative	transactivation of RNA	1.53E-08	152
Rumen	Corn stover	Negative	size of body	1.83E-08	223
Rumen	Corn stover	Negative	digestive system cancer	1.97E-08	1566
Rumen	Corn stover	Negative	development of cytoplasm	1.97E-08	132
Rumen	Corn stover	Negative	colony formation of cells	2.22E-08	139
Rumen	Corn stover	Negative	development of connective tissue	3.49E-08	109
Rumen	Corn stover	Negative	morphology of vessel	3.81E-08	93
Rumen	Corn stover	Negative	quantity of mononuclear leukocytes	4.17E-08	195
Rumen	Corn stover	Negative	protein kinase cascade	4.17E-08	121
Rumen	Corn stover	Negative	disorder of basal ganglia	4.60E-08	202
Rumen	Corn stover	Negative	growth of epithelial tissue	4.64E-08	182
Rumen	Corn stover	Negative	proliferation of blood cells	4.87E-08	219
Rumen	Corn stover	Negative	neuromuscular disease	5.15E-08	228
Rumen	Corn stover	Negative	quantity of lymphocytes	5.33E-08	188
Rumen	Corn stover	Negative	abnormal morphology of heart	6.66E-08	111
Rumen	Corn stover	Negative	differentiation of epithelial tissue	7.50E-08	105
Rumen	Corn stover	Negative	activation of DNA endogenous promoter	7.70E-08	299
Rumen	Corn	Negative	formation of cytoskeleton	7.70E-08	109

	stover				
Rumen	Corn stover	Negative	proliferation of fibroblasts	9.87E-08	103
Rumen	Corn stover	Negative	proliferation of immune cells	1.03E-07	206
Rumen	Corn stover	Negative	apoptosis of heart cells	1.05E-07	62
Rumen	Corn stover	Negative	proliferation of neuronal cells	1.19E-07	156
Rumen	Corn stover	Negative	development of body trunk	1.50E-07	277
Rumen	Corn stover	Negative	morphology of reproductive system	1.50E-07	146
Rumen	Corn stover	Negative	abnormal morphology of abdomen	1.67E-07	224
Rumen	Corn stover	Negative	abnormal morphology of epithelial tissue	1.77E-07	118
Rumen	Corn stover	Negative	cell movement of endothelial cells	2.22E-07	102
Rumen	Corn stover	Negative	abnormal morphology of reproductive system	2.44E-07	139
Rumen	Corn stover	Negative	growth of organism	2.55E-07	214
Rumen	Corn stover	Negative	dyskinesia	2.67E-07	164
Rumen	Corn stover	Negative	cell cycle progression	3.26E-07	260
Rumen	Corn stover	Negative	morphology of blood vessel	3.40E-07	84
Rumen	Corn stover	Negative	cell death of fibroblast cell lines	3.44E-07	119
Rumen	Corn stover	Negative	Viral Infection	3.74E-07	380
Rumen	Corn stover	Negative	apoptosis of fibroblast cell lines	3.74E-07	95
Rumen	Corn stover	Negative	migration of endothelial cells	3.74E-07	95
Rumen	Corn stover	Negative	fibrogenesis	3.88E-07	115
Rumen	Corn stover	Negative	neurological signs	4.18E-07	171
Rumen	Corn stover	Negative	long-term potentiation	4.44E-07	74
Rumen	Corn	Negative	differentiation of epithelial cells	5.06E-07	91

	stover				
Rumen	Corn stover	Negative	apoptosis of cardiomyocytes	5.41E-07	59
Rumen	Corn stover	Negative	neonatal death	5.75E-07	121
Rumen	Corn stover	Negative	growth of muscle tissue	6.76E-07	105
Rumen	Corn stover	Negative	Edema	6.81E-07	98
Rumen	Corn stover	Negative	phosphorylation of protein	7.21E-07	189
Rumen	Corn stover	Negative	growth of plasma membrane projections	7.21E-07	127
Rumen	Corn stover	Negative	proliferation of lymphocytes	7.51E-07	187
Rumen	Corn stover	Negative	migration of vascular endothelial cells	7.78E-07	54
Rumen	Corn stover	Negative	morphology of cardiac muscle	7.78E-07	54
Rumen	Corn stover	Negative	formation of skin	8.70E-07	103
Rumen	Corn stover	Negative	proliferation of muscle cells	8.87E-07	104
Rumen	Corn stover	Negative	proliferation of mononuclear leukocytes	9.46E-07	189
Rumen	Corn stover	Negative	cell death of heart cells	1.11E-06	67
Rumen	Corn stover	Negative	formation of filaments	1.11E-06	110
Rumen	Corn stover	Negative	seizure disorder	1.14E-06	127
Rumen	Corn stover	Negative	interphase	1.15E-06	170
Rumen	Corn stover	Negative	morphology of muscle	1.19E-06	95
Rumen	Corn stover	Negative	development of lymphatic system	1.22E-06	100
Rumen	Corn stover	Negative	growth of neurites	1.22E-06	125
Rumen	Corn stover	Negative	apoptosis of muscle	1.27E-06	77
Rumen	Corn stover	Negative	accumulation of lipid	1.28E-06	86
Rumen	Corn	Negative	development of cardiovascular tissue	1.28E-06	103

	stover				
Rumen	Corn stover	Negative	fatty acid metabolism	1.28E-06	157
Rumen	Corn stover	Negative	morphology of respiratory system	1.28E-06	89
Rumen	Corn stover	Negative	Huntington's Disease	1.28E-06	151
Rumen	Corn stover	Negative	growth of tumor	1.42E-06	201
Rumen	Corn stover	Negative	organization of actin cytoskeleton	1.87E-06	86
Rumen	Corn stover	Negative	apoptosis of muscle cells	2.03E-06	76
Rumen	Corn stover	Negative	quantity of T lymphocytes	2.09E-06	141
Rumen	Corn stover	Negative	quantity of lymphatic system component	2.26E-06	104
Rumen	Corn stover	Negative	homing of cells	2.32E-06	146
Rumen	Corn stover	Negative	abnormal morphology of blood vessel	2.46E-06	77
Rumen	Corn stover	Negative	neuronal cell death	2.52E-06	174
Rumen	Corn stover	Negative	uptake of carbohydrate	2.63E-06	83
Rumen	Corn stover	Negative	morphology of head	2.93E-06	217
Rumen	Corn stover	Negative	proliferation of epidermal cells	3.24E-06	53
Rumen	Corn stover	Negative	cell death of blood cells	3.49E-06	176
Rumen	Corn stover	Negative	movement of vascular endothelial cells	3.59E-06	56
Rumen	Corn stover	Negative	cell movement of connective tissue cells	3.72E-06	66
Rumen	Corn stover	Negative	cell spreading	4.07E-06	79
Rumen	Corn stover	Negative	morphology of genital organ	4.07E-06	114
Rumen	Corn stover	Negative	proliferation of smooth muscle cells	4.44E-06	81
Rumen	Corn stover	Negative	function of cardiovascular system	4.44E-06	101
Rumen	Corn	Negative	morphogenesis of neurons	4.44E-06	114

	stover				
Rumen	Corn stover	Negative	uptake of monosaccharide	4.64E-06	77
Rumen	Corn stover	Negative	abnormal morphology of respiratory system	4.77E-06	86
Rumen	Corn stover	Negative	development of endothelial tissue	4.79E-06	100
Rumen	Corn stover	Negative	migration of blood cells	5.03E-06	215
Rumen	Corn stover	Negative	endothelial cell development	5.03E-06	97
Rumen	Corn stover	Negative	morphology of connective tissue	5.16E-06	122
Rumen	Corn stover	Negative	abnormal morphology of head	5.16E-06	206
Rumen	Corn stover	Negative	epilepsy	5.19E-06	86
Rumen	Corn stover	Negative	quantity of lymphoid organ	5.19E-06	86
Rumen	Corn stover	Negative	homing	5.19E-06	148
Rumen	Corn stover	Negative	morphogenesis of neurites	5.67E-06	112
Rumen	Corn stover	Negative	formation of actin filaments	6.36E-06	85
Rumen	Corn stover	Negative	differentiation of epidermal cells	6.40E-06	51
Rumen	Corn stover	Negative	leukocyte migration	6.48E-06	214
Rumen	Corn stover	Negative	development of lymphatic system component	6.48E-06	86
Rumen	Corn stover	Negative	differentiation of skin	6.71E-06	55
Rumen	Corn stover	Negative	outgrowth of plasma membrane projections	6.71E-06	109
Rumen	Corn stover	Negative	growth of skin	6.93E-06	64
Rumen	Corn stover	Negative	sprouting	7.18E-06	104
Rumen	Corn stover	Negative	morphology of skin	7.22E-06	77
Rumen	Corn stover	Negative	cell death of cardiomyocytes	7.22E-06	63
Rumen	Corn	Negative	outgrowth of neurons	7.22E-06	109

	stover				
Rumen	Corn stover	Negative	outgrowth of cells	7.22E-06	115
Rumen	Corn stover	Negative	morphology of muscle cells	7.27E-06	51
Rumen	Corn stover	Negative	development of blood cells	7.35E-06	165
Rumen	Corn stover	Negative	growth of embryo	7.36E-06	125
Rumen	Corn stover	Negative	development of genitourinary system	7.59E-06	217
Rumen	Corn stover	Negative	outgrowth of neurites	7.75E-06	108
Rumen	Corn stover	Negative	abnormal morphology of genital organ	7.75E-06	109
Rumen	Corn stover	Negative	development of epithelial tissue	7.75E-06	134
Rumen	Corn stover	Negative	morphology of nervous system	7.81E-06	194
Rumen	Corn stover	Negative	concentration of lipid	7.84E-06	200
Rumen	Corn stover	Negative	dysgenesis	8.41E-06	139
Rumen	Corn stover	Negative	S phase	8.57E-06	74
Rumen	Corn stover	Negative	cell death of immune cells	9.31E-06	166
Rumen	Corn stover	Negative	development of leukocytes	9.31E-06	150
Rumen	Corn stover	Negative	morphology of digestive system	9.31E-06	146
Rumen	Corn stover	Negative	chemotaxis of cells	9.45E-06	136
Rumen	Corn stover	Negative	cell movement of fibroblasts	1.01E-05	55
Rumen	Corn stover	Negative	Growth Failure	1.03E-05	152
Rumen	Corn stover	Negative	differentiation of keratinocytes	1.05E-05	47
Rumen	Corn stover	Negative	quantity of thymus gland	1.07E-05	67
Rumen	Corn stover	Negative	cell spreading of kidney cell lines	1.09E-05	14
Rumen	Corn	Negative	T cell development	1.11E-05	130

	stover				
Rumen	Corn stover	Negative	quantity of metal	1.12E-05	129
Rumen	Corn stover	Negative	differentiation of connective tissue	1.20E-05	181
Rumen	Corn stover	Negative	neuritogenesis	1.29E-05	148
Rumen	Corn stover	Negative	migration of connective tissue cells	1.44E-05	54
Rumen	Corn stover	Negative	quantity of thymocytes	1.50E-05	66
Rumen	Corn stover	Negative	differentiation of muscle	1.51E-05	83
Rumen	Corn stover	Negative	chemotaxis	1.55E-05	139
Rumen	Corn stover	Negative	apoptosis of leukocyte cell lines	1.76E-05	53
Rumen	Corn stover	Negative	synthesis of fatty acid	1.78E-05	85
Rumen	Corn stover	Negative	proliferation of T lymphocytes	1.85E-05	152
Rumen	Corn stover	Negative	T cell homeostasis	1.91E-05	132
Rumen	Corn stover	Negative	hyperplasia of tissue	1.96E-05	57
Rumen	Corn stover	Negative	cell movement of leukocytes	1.99E-05	188
Rumen	Corn stover	Negative	apoptosis of carcinoma cell lines	2.02E-05	69
Rumen	Corn stover	Negative	formation of plasma membrane projections	2.07E-05	150
Rumen	Corn stover	Negative	potentiation of synapse	2.15E-05	46
Rumen	Corn stover	Negative	formation of lymphatic system component	2.16E-05	80
Rumen	Corn stover	Negative	abnormal morphology of digestive system	2.31E-05	138
Rumen	Corn stover	Negative	necrosis of epithelial tissue	2.31E-05	148
Rumen	Corn stover	Negative	Hypoplasia	2.31E-05	129
Rumen	Corn stover	Negative	synthesis of lipid	2.32E-05	176
Rumen	Corn	Negative	cell movement of myeloid cells	2.32E-05	137

	stover				
Rumen	Corn stover	Negative	uptake of D-glucose	2.32E-05	63
Rumen	Corn stover	Negative	neoplasia of cells	2.32E-05	115
Rumen	Corn stover	Negative	metastasis	2.32E-05	186
Rumen	Corn stover	Negative	cell movement of phagocytes	2.37E-05	139
Rumen	Corn stover	Negative	development of reproductive system	2.37E-05	181
Rumen	Corn stover	Negative	Lymphocyte homeostasis	2.41E-05	138
Rumen	Corn stover	Negative	quantity of connective tissue	2.41E-05	151
Rumen	Corn stover	Negative	abnormal morphology of interalveolar septa	2.51E-05	18
Rumen	Corn stover	Negative	proliferation of epithelial cells	2.54E-05	126
Rumen	Corn stover	Negative	cell death of T lymphocytes	2.58E-05	85
Rumen	Corn stover	Negative	homeostasis of leukocytes	2.66E-05	140
Rumen	Corn stover	Negative	function of leukocytes	2.67E-05	136
Rumen	Corn stover	Negative	cell death of carcinoma cell lines	2.72E-05	78
Rumen	Corn stover	Negative	function of blood cells	2.82E-05	147
Rumen	Corn stover	Negative	abnormal morphology of internal genitalia	2.82E-05	93
Rumen	Corn stover	Negative	cell death of fibroblasts	2.88E-05	73
Rumen	Corn stover	Negative	formation of lymphoid organ	2.88E-05	73
Rumen	Corn stover	Negative	differentiation of muscle cells	2.93E-05	77
Rumen	Corn stover	Negative	cell viability of carcinoma cell lines	2.95E-05	44
Rumen	Corn stover	Negative	apoptosis of B-lymphocyte derived cell lines	2.96E-05	39
Rumen	Corn stover	Negative	transport of carbohydrate	3.46E-05	56
Rumen	Corn	Negative	long-term potentiation of synapse	3.46E-05	45

	stover				
Rumen	Corn stover	Negative	cell death of leukocyte cell lines	3.49E-05	58
Rumen	Corn stover	Negative	development of body axis	3.49E-05	248
Rumen	Corn stover	Negative	development of mononuclear leukocytes	3.49E-05	140
Rumen	Corn stover	Negative	differentiation of connective tissue cells	3.55E-05	159
Rumen	Corn stover	Negative	development of lymphocytes	3.55E-05	139
Rumen	Corn stover	Negative	metabolism of protein	3.62E-05	231
Rumen	Corn stover	Negative	proliferation of keratinocytes	3.95E-05	46
Rumen	Corn stover	Negative	survival of organism	4.13E-05	172
Rumen	Corn stover	Negative	formation of filopodia	4.17E-05	49
Rumen	Corn stover	Negative	generation of lymphocytes	4.22E-05	43
Rumen	Corn stover	Negative	proliferation of hematopoietic cells	4.60E-05	70
Rumen	Corn stover	Negative	morphology of bone	4.73E-05	115
Rumen	Corn stover	Negative	stress response of cells	5.17E-05	42
Rumen	Corn stover	Negative	G1/S phase transition	5.26E-05	60
Rumen	Corn stover	Negative	cell death of dermal cells	5.26E-05	32
Rumen	Corn stover	Negative	hypersensitive reaction	5.47E-05	110
Rumen	Corn stover	Negative	apoptosis of dermal cells	5.69E-05	30
Rumen	Corn stover	Negative	epileptic seizure	5.69E-05	49
Rumen	Corn stover	Negative	quantity of metal ion	5.70E-05	113
Rumen	Corn stover	Negative	seizures	5.78E-05	103
Rumen	Corn stover	Negative	Dermatitis	5.81E-05	111
Rumen	Corn	Negative	mitosis	5.91E-05	125

	stover				
Rumen	Corn stover	Negative	apoptosis of skin	6.09E-05	31
Rumen	Corn stover	Negative	transport of carboxylic acid	6.18E-05	37
Rumen	Corn stover	Negative	degeneration of nervous system	6.18E-05	77
Rumen	Corn stover	Negative	formation of actin stress fibers	6.18E-05	66
Rumen	Corn stover	Negative	formation of osteoclasts	6.18E-05	38
Rumen	Corn stover	Negative	morphology of gonad	6.37E-05	98
Rumen	Corn stover	Negative	advanced malignant tumor	6.37E-05	202
Rumen	Corn stover	Negative	branching of cells	6.39E-05	96
Rumen	Corn stover	Negative	replication of RNA virus	6.49E-05	127
Rumen	Corn stover	Negative	development of head	6.66E-05	231
Rumen	Corn stover	Negative	synthesis of reactive oxygen species	6.72E-05	121
Rumen	Corn stover	Negative	apoptosis of breast cancer cell lines	6.73E-05	77
Rumen	Corn stover	Negative	development of neurons	6.76E-05	188
Rumen	Corn stover	Negative	cell death of muscle cells	6.87E-05	88
Rumen	Corn stover	Negative	colony formation of tumor cell lines	6.98E-05	74
Rumen	Corn stover	Negative	formation of connective tissue cells	6.98E-05	59
Rumen	Corn stover	Negative	concentration of fatty acid	7.12E-05	75
Rumen	Corn stover	Negative	formation of vesicles	7.24E-05	37
Rumen	Corn stover	Negative	shape change of kidney cell lines	7.34E-05	23
Rumen	Corn stover	Negative	differentiation of blood cells	7.63E-05	201
Rumen	Corn stover	Negative	quantity of epithelial tissue	7.64E-05	51
Rumen	Corn	Negative	generation of blood cells	7.66E-05	48

	stover				
Rumen	Corn stover	Negative	cell death of melanoma cell lines	8.19E-05	68
Rumen	Corn stover	Negative	cancer of cells	8.45E-05	69
Rumen	Corn stover	Negative	apoptosis of blood cells	8.45E-05	124
Rumen	Corn stover	Negative	cell death of hepatoma cell lines	8.46E-05	49
Rumen	Corn stover	Negative	cell death of cerebral cortex cells	8.47E-05	63
Rumen	Corn stover	Negative	cell death of pheochromocytoma cell lines	8.64E-05	37
Rumen	Corn stover	Negative	cell death of breast cancer cell lines	8.97E-05	87
Rumen	Corn stover	Negative	synthesis of prostaglandin	9.27E-05	50
Rumen	Corn stover	Negative	congenital anomaly of musculoskeletal system	9.48E-05	177
Rumen	Corn stover	Negative	branching of neurites	9.48E-05	74
Rumen	Corn stover	Negative	proliferation of hematopoietic progenitor cells	9.49E-05	66
Rumen	Corn stover	Negative	differentiation of leukocytes	9.56E-05	163
Rumen	Corn stover	Negative	abnormal morphology of muscle	9.56E-05	83
Rumen	Corn stover	Negative	cell death of epithelial cells	9.61E-05	124
Rumen	Corn stover	Negative	inflammation of organ	9.61E-05	271
Rumen	Corn stover	Negative	abnormal morphology of gonad	9.62E-05	94
Rumen	Corn stover	Negative	branching of neurons	9.64E-05	76
Rumen	Corn stover	Negative	cell death of skin	9.64E-05	33
Rumen	Corn stover	Negative	activation of Protein kinase	9.95E-05	68
Rumen	Corn stover	Negative	cell death of epidermal cells	1.04E-04	30
Rumen	Corn stover	Negative	abnormal morphology of skin	1.05E-04	66
Rumen	Corn	Negative	development of connective tissue cells	1.09E-04	61

	stover				
Rumen	Corn stover	Negative	apoptosis of connective tissue cells	1.10E-04	78
Rumen	Corn stover	Negative	tumorigenesis of malignant tumor	1.10E-04	72
Rumen	Corn stover	Negative	formation of leukocytes	1.10E-04	46
Rumen	Corn stover	Negative	replication of virus	1.12E-04	138
Rumen	Corn stover	Negative	apoptosis of hematopoietic cell lines	1.12E-04	57
Rumen	Corn stover	Negative	organization of filaments	1.12E-04	57
Rumen	Corn stover	Negative	abnormal morphology of bone	1.12E-04	110
Rumen	Corn stover	Negative	abnormal morphology of nervous system	1.13E-04	174
Rumen	Corn stover	Negative	cognition	1.13E-04	114
Rumen	Corn stover	Negative	cell death of muscle	1.14E-04	89
Rumen	Corn stover	Negative	activation of enzyme	1.18E-04	99
Rumen	Corn stover	Negative	cell death of neuroblastoma cell lines	1.20E-04	54
Rumen	Corn stover	Negative	cell movement of antigen presenting cells	1.24E-04	90
Rumen	Corn stover	Negative	endocytosis	1.24E-04	89
Rumen	Corn stover	Negative	metabolism of reactive oxygen species	1.25E-04	124
Rumen	Corn stover	Negative	quantity of cellular protrusions	1.25E-04	46
Rumen	Corn stover	Negative	migration of fibroblasts	1.26E-04	42
Rumen	Corn stover	Negative	metabolism of prostaglandin	1.26E-04	51
Rumen	Corn stover	Negative	G1 phase	1.27E-04	99
Rumen	Corn stover	Negative	quantity of carbohydrate	1.30E-04	128
Rumen	Corn stover	Negative	quantity of B lymphocytes	1.35E-04	89
Rumen	Corn	Negative	formation of mononuclear leukocytes	1.37E-04	39

	stover				
Rumen	Corn stover	Negative	shape change of neurites	1.38E-04	75
Rumen	Corn stover	Negative	cell movement of breast cancer cell lines	1.40E-04	76
Rumen	Corn stover	Negative	apoptosis of neuroblastoma cell lines	1.40E-04	37
Rumen	Corn stover	Negative	cell movement of epithelial cells	1.41E-04	43
Rumen	Corn stover	Negative	behavior	1.49E-04	230
Rumen	Corn stover	Negative	infection by Retroviridae	1.49E-04	172
Rumen	Corn stover	Negative	aggregation of cells	1.63E-04	84
Rumen	Corn stover	Negative	I-kappaB kinase/NF-kappaB cascade	1.63E-04	49
Rumen	Corn stover	Negative	apoptosis of cervical cancer cell lines	1.67E-04	74
Rumen	Corn stover	Negative	development of abdomen	1.72E-04	141
Rumen	Corn stover	Negative	apoptosis of epidermal cells	1.72E-04	28
Rumen	Corn stover	Negative	binding of DNA	1.72E-04	128
Rumen	Corn stover	Negative	congenital malformation of skeleton	1.73E-04	106
Rumen	Corn stover	Negative	transmigration of cells	1.74E-04	45
Rumen	Corn stover	Negative	size of animal	1.74E-04	50
Rumen	Corn stover	Negative	function of muscle	1.74E-04	89
Rumen	Corn stover	Negative	differentiation of tumor cell lines	1.80E-04	96
Rumen	Corn stover	Negative	generation of leukocytes	1.92E-04	46
Rumen	Corn stover	Negative	Neurodegeneration	1.92E-04	80
Rumen	Corn stover	Negative	cell death of hematopoietic cell lines	1.95E-04	62
Rumen	Corn stover	Negative	hypoplasia of organ	1.99E-04	111
Rumen	Corn	Negative	accumulation of reactive oxygen species	1.99E-04	27

	stover				
Rumen	Corn stover	Negative	congenital heart disease	2.02E-04	57
Rumen	Corn stover	Negative	cell death of keratinocytes	2.13E-04	28
Rumen	Corn stover	Negative	morphology of cardiovascular tissue	2.13E-04	28
Rumen	Corn stover	Negative	transport of cation	2.17E-04	101
Rumen	Corn stover	Negative	development of hematopoietic system	2.17E-04	66
Rumen	Corn stover	Negative	generation of reactive oxygen species	2.17E-04	56
Rumen	Corn stover	Negative	dendritic growth/branching	2.19E-04	59
Rumen	Corn stover	Negative	maturation of cells	2.20E-04	113
Rumen	Corn stover	Negative	secretory pathway	2.21E-04	54
Rumen	Corn stover	Negative	apoptosis of pheochromocytoma cell lines	2.21E-04	29
Rumen	Corn stover	Negative	autosomal dominant disease	2.22E-04	153
Rumen	Corn stover	Negative	generation of cells	2.28E-04	69
Rumen	Corn stover	Negative	quantity of Ca2+	2.28E-04	102
Rumen	Corn stover	Negative	transport of monosaccharide	2.28E-04	45
Rumen	Corn stover	Negative	quantity of macrophages	2.30E-04	55
Rumen	Corn stover	Negative	congenital anomaly of cardiovascular system	2.31E-04	61
Rumen	Corn stover	Negative	apoptosis of leukocytes	2.34E-04	113
Rumen	Corn stover	Negative	metastasis of cells	2.36E-04	66
Rumen	Corn stover	Negative	morphology of epidermis	2.36E-04	41
Rumen	Corn stover	Negative	synthesis of DNA	2.36E-04	104
Rumen	Corn stover	Negative	migration of stomach cancer cell lines	2.41E-04	12
Rumen	Corn	Negative	relaxation of muscle	2.42E-04	27

	stover				
Rumen	Corn stover	Negative	proliferation of endothelial cells	2.46E-04	80
Rumen	Corn stover	Negative	apoptosis of epithelial cell lines	2.48E-04	60
Rumen	Corn stover	Negative	function of smooth muscle	2.69E-04	29
Rumen	Corn stover	Negative	morphology of cardiomyocytes	2.69E-04	29
Rumen	Corn stover	Negative	formation of lymphocytes	2.69E-04	37
Rumen	Corn stover	Negative	cardiogenesis	2.70E-04	116
Rumen	Corn stover	Negative	cell death of hematopoietic cells	2.71E-04	63
Rumen	Corn stover	Negative	transport of inorganic cation	2.85E-04	89
Rumen	Corn stover	Negative	development of digestive system	2.87E-04	96
Rumen	Corn stover	Negative	inflammatory response	2.87E-04	183
Rumen	Corn stover	Negative	apoptosis of hepatoma cell lines	2.88E-04	43
Rumen	Corn stover	Negative	abnormal morphology of endothelial tissue	2.97E-04	22
Rumen	Corn stover	Negative	morphology of stratum corneum	3.04E-04	16
Rumen	Corn stover	Negative	cell death of epithelial cell lines	3.18E-04	73
Rumen	Corn stover	Negative	cell movement of stomach cancer cell lines	3.26E-04	13
Rumen	Corn stover	Negative	replication of Hepatitis C virus	3.29E-04	25
Rumen	Corn stover	Negative	midline defect	3.38E-04	69
Rumen	Corn stover	Negative	biosynthesis of purine ribonucleotide	3.41E-04	31
Rumen	Corn stover	Negative	necrosis of tumor	3.41E-04	95
Rumen	Corn stover	Negative	cell death of tumor cells	3.50E-04	93
Rumen	Corn stover	Negative	arrest in interphase	3.50E-04	103
Rumen	Corn	Negative	apoptosis of keratinocytes	3.52E-04	26

	stover				
Rumen	Corn stover	Negative	HIV infection	3.62E-04	168
Rumen	Corn stover	Negative	cell death of lymphocytes	3.62E-04	96
Rumen	Corn stover	Negative	differentiation of lymphocytes	3.62E-04	121
Rumen	Corn stover	Negative	apoptosis of fibroblasts	3.63E-04	58
Rumen	Corn stover	Negative	abnormal morphology of embryonic tissue	3.77E-04	132
Rumen	Corn stover	Negative	metabolism of carbohydrate	3.86E-04	148
Rumen	Corn stover	Negative	mass of genitourinary system	3.86E-04	76
Rumen	Corn stover	Negative	morphology of lung	3.87E-04	51
Rumen	Corn stover	Negative	arrest in proliferation of cells	3.93E-04	57
Rumen	Corn stover	Negative	generation of T lymphocytes	3.98E-04	34
Rumen	Corn stover	Negative	cell death of thymocytes	4.02E-04	42
Rumen	Corn stover	Negative	learning	4.02E-04	103
Rumen	Corn stover	Negative	respiratory system development	4.02E-04	86
Rumen	Corn stover	Negative	morphology of heart ventricle	4.02E-04	52
Rumen	Corn stover	Negative	chronic myeloid leukemia	4.02E-04	32
Rumen	Corn stover	Negative	import of cation	4.02E-04	12
Rumen	Corn stover	Negative	apoptosis of T lymphocytes	4.12E-04	72
Rumen	Corn stover	Negative	allergy	4.23E-04	100
Rumen	Corn stover	Negative	oxidative stress response of cells	4.26E-04	21
Rumen	Corn stover	Negative	chemotaxis of antigen presenting cells	4.33E-04	43
Rumen	Corn stover	Negative	proliferation of vascular smooth muscle cells	4.33E-04	43
Rumen	Corn	Negative	shape change of tumor cell lines	4.33E-04	43

	stover				
Rumen	Corn stover	Negative	autosomal recessive disease	4.57E-04	212
Rumen	Corn stover	Negative	cell death of mononuclear leukocytes	4.73E-04	98
Rumen	Corn stover	Negative	formation of blood cells	4.74E-04	53
Rumen	Corn stover	Negative	cell movement of granulocytes	4.79E-04	92
Rumen	Corn stover	Negative	tyrosine phosphorylation of protein	4.89E-04	51
Rumen	Corn stover	Negative	cell movement of muscle cells	5.01E-04	49
Rumen	Corn stover	Negative	scattering of cells	5.06E-04	25
Rumen	Corn stover	Negative	secretion of molecule	5.10E-04	132
Rumen	Corn stover	Negative	infection by RNA virus	5.27E-04	200
Rumen	Corn stover	Negative	morphology of endothelial tissue	5.35E-04	26
Rumen	Corn stover	Negative	morphology of lymphatic system component	5.50E-04	102
Rumen	Corn stover	Negative	morphology of myocardium	5.53E-04	34
Rumen	Corn stover	Negative	ruffling	5.77E-04	32
Rumen	Corn stover	Negative	cell death of central nervous system cells	6.06E-04	74
Rumen	Corn stover	Negative	chemotaxis of myeloid cells	6.06E-04	74
Rumen	Corn stover	Negative	benign neoplasia	6.08E-04	212
Rumen	Corn stover	Negative	cell movement of prostate cancer cell lines	6.12E-04	36
Rumen	Corn stover	Negative	Thrombosis	6.19E-04	46
Rumen	Corn stover	Negative	synthesis of prostaglandin E2	6.24E-04	39
Rumen	Corn stover	Negative	differentiation of smooth muscle	6.27E-04	22
Rumen	Corn stover	Negative	atrophy of muscle	6.48E-04	41
Rumen	Corn	Negative	cell death of cervical cancer cell lines	6.52E-04	87

	stover				
Rumen	Corn stover	Negative	morphology of mouth	6.53E-04	38
Rumen	Corn stover	Negative	migration of muscle cells	6.60E-04	45
Rumen	Corn stover	Negative	differentiation of mononuclear leukocytes	6.69E-04	129
Rumen	Corn stover	Negative	dephosphorylation of protein	6.69E-04	50
Rumen	Corn stover	Negative	hypertrophy of heart	6.71E-04	84
Rumen	Corn stover	Negative	transport of D-glucose	6.79E-04	40
Rumen	Corn stover	Negative	reorganization of cytoskeleton	6.98E-04	51
Rumen	Corn stover	Negative	progression of tumor	7.01E-04	44
Rumen	Corn stover	Negative	transformation of fibroblast cell lines	7.01E-04	66
Rumen	Corn stover	Negative	development of gastrointestinal tract	7.05E-04	67
Rumen	Corn stover	Negative	expansion of cells	7.09E-04	76
Rumen	Corn stover	Negative	metabolism of nucleoside triphosphate	7.12E-04	39
Rumen	Corn stover	Negative	Hypertrophy	7.23E-04	114
Rumen	Corn stover	Negative	hepatobiliary system cancer	7.36E-04	904
Rumen	Corn stover	Negative	differentiation of T lymphocytes	7.43E-04	93
Rumen	Corn stover	Negative	size of embryo	7.43E-04	88
Rumen	Corn stover	Negative	angiogenesis of eye	7.43E-04	18
Rumen	Corn stover	Negative	senescence of cells	7.43E-04	63
Rumen	Corn stover	Negative	liver cancer	7.47E-04	900
Rumen	Corn stover	Negative	morphology of pulmonary alveolus	7.52E-04	34
Rumen	Corn stover	Negative	cell death of hematopoietic progenitor cells	7.72E-04	59
Rumen	Corn	Negative	urination disorder	7.72E-04	73

	stover				
Rumen	Corn stover	Negative	formation of vascular lesion	7.72E-04	33
Rumen	Corn stover	Negative	synthesis of nitric oxide	7.72E-04	70
Rumen	Corn stover	Negative	infection of cells	7.81E-04	184
Rumen	Corn stover	Negative	concentration of acylglycerol	7.83E-04	83
Rumen	Corn stover	Negative	quantity of hematopoietic cells	7.90E-04	107
Rumen	Corn stover	Negative	catabolism of protein	8.03E-04	142
Rumen	Corn stover	Negative	activation of cells	8.09E-04	222
Rumen	Corn stover	Negative	migration of breast cancer cell lines	8.11E-04	63
Rumen	Corn stover	Negative	mammary tumor	8.14E-04	359
Rumen	Corn stover	Negative	engulfment of cells	8.14E-04	99
Rumen	Corn stover	Negative	homeostasis of inorganic cation	8.24E-04	50
Rumen	Corn stover	Negative	Organ Degeneration	8.27E-04	118
Rumen	Corn stover	Negative	chemotaxis of phagocytes	8.27E-04	74
Rumen	Corn stover	Negative	export of molecule	8.27E-04	74
Rumen	Corn stover	Negative	liver tumor	8.27E-04	905
Rumen	Corn stover	Negative	G2 phase	8.27E-04	68
Rumen	Corn stover	Negative	repair of DNA	8.27E-04	73
Rumen	Corn stover	Negative	cell death of brain cells	8.28E-04	69
Rumen	Corn stover	Negative	peripheral vascular disease	8.38E-04	93
Rumen	Corn stover	Negative	tumorigenesis of epithelial neoplasm	8.40E-04	48
Rumen	Corn stover	Negative	necrosis of liver	8.61E-04	61
Rumen	Corn	Negative	progressive motor neuropathy	8.95E-04	124

	stover				
Rumen	Corn stover	Negative	cell death of brain	9.04E-04	73
Rumen	Corn stover	Negative	biosynthesis of polyunsaturated fatty acids	9.12E-04	58
Rumen	Corn stover	Negative	polyubiquitination	9.18E-04	25
Rumen	Corn stover	Negative	biosynthesis of nucleoside triphosphate	9.39E-04	31
Rumen	Corn stover	Negative	morphology of fibroblast cell lines	9.46E-04	26
Rumen	Corn stover	Negative	peripheral arterial disease	9.50E-04	55
Rumen	Corn stover	Negative	apoptosis of kidney cell lines	9.50E-04	61
Rumen	Corn stover	Negative	quantity of neurons	9.50E-04	101
Rumen	Corn stover	Negative	infection by HIV-1	9.55E-04	143
Rumen	Corn stover	Negative	formation of lung	9.70E-04	75
Rumen	Corn stover	Negative	hepatocellular carcinoma	9.73E-04	871
Rumen	Corn stover	Negative	synthesis of eicosanoid	9.86E-04	57
Rumen	Corn stover	Negative	breast or colorectal cancer	9.97E-04	1026
Rumen	Corn stover	Negative	development of exocrine gland	1.00E-03	58
Rumen	Corn stover	Negative	exocytosis	1.02E-03	50
Rumen	Corn stover	Negative	formation of T lymphocytes	1.04E-03	33
Rumen	Corn stover	Negative	quantity of hematopoietic progenitor cells	1.04E-03	106
Rumen	Corn stover	Negative	colony formation of lung cancer cell lines	1.04E-03	17
Rumen	Corn stover	Negative	abnormal morphology of artery	1.06E-03	41
Rumen	Corn stover	Negative	cell movement of neutrophils	1.06E-03	74
Rumen	Corn stover	Negative	apoptosis of melanoma cell lines	1.06E-03	36
Rumen	Corn	Negative	transport of metal	1.06E-03	82

	stover				
Duodenum	Alfalfa hay	Positive	proliferation of cells	5.46E-12	586
Duodenum	Alfalfa hay	Positive	necrosis	1.69E-11	429
Duodenum	Alfalfa hay	Positive	organization of cytoplasm	2.10E-11	279
Duodenum	Alfalfa hay	Positive	cell death	2.75E-10	526
Duodenum	Alfalfa hay	Positive	organismal death	5.65E-10	395
Duodenum	Alfalfa hay	Positive	morbidity or mortality	1.51E-09	397
Duodenum	Alfalfa hay	Positive	organization of cytoskeleton	2.22E-09	249
Duodenum	Alfalfa hay	Positive	apoptosis	3.76E-09	424
Duodenum	Alfalfa hay	Positive	transport of molecule	1.14E-08	277
Duodenum	Alfalfa hay	Positive	morphology of cells	2.02E-08	323
Duodenum	Alfalfa hay	Positive	cancer	3.21E-08	1463
Duodenum	Alfalfa hay	Positive	microtubule dynamics	3.21E-08	212
Duodenum	Alfalfa hay	Positive	malignant solid tumor	6.27E-08	1445
Duodenum	Alfalfa hay	Positive	cell death of tumor cell lines	1.21E-07	259
Duodenum	Alfalfa hay	Positive	dyskinesia	1.34E-07	122
Duodenum	Alfalfa hay	Positive	cellular homeostasis	2.08E-07	257
Duodenum	Alfalfa hay	Positive	cell movement	3.20E-07	341
Duodenum	Alfalfa hay	Positive	neurological signs	3.20E-07	126
Duodenum	Alfalfa hay	Positive	Huntington's Disease	4.87E-07	113
Duodenum	Alfalfa hay	Positive	apoptosis of tumor cell lines	4.87E-07	210
Duodenum	Alfalfa hay	Positive	transcription of DNA	6.52E-07	256
Duodenum	Alfalfa	Positive	expression of RNA	7.53E-07	341

	hay				
Duodenum	Alfalfa hay	Positive	transcription of RNA	1.07E-06	300
Duodenum	Alfalfa hay	Positive	disorder of basal ganglia	1.75E-06	141
Duodenum	Alfalfa hay	Positive	Movement Disorders	2.78E-06	184
Duodenum	Alfalfa hay	Positive	epileptic seizure	6.09E-06	41
Duodenum	Alfalfa hay	Positive	formation of cellular protrusions	6.79E-06	159
Duodenum	Alfalfa hay	Positive	transcription	6.79E-06	314
Duodenum	Alfalfa hay	Positive	differentiation of cells	9.42E-06	348
Duodenum	Alfalfa hay	Positive	proliferation of tumor cell lines	9.42E-06	251
Duodenum	Alfalfa hay	Positive	epithelial cancer	1.64E-05	1189
Duodenum	Alfalfa hay	Positive	proliferation of neuronal cells	1.90E-05	107
Duodenum	Alfalfa hay	Positive	size of body	1.90E-05	149
Duodenum	Alfalfa hay	Positive	transactivation	2.27E-05	107
Duodenum	Alfalfa hay	Positive	seizures	3.26E-05	78
Duodenum	Alfalfa hay	Positive	homing of cells	3.57E-05	103
Duodenum	Alfalfa hay	Positive	neoplasia of epithelial tissue	3.84E-05	1196
Duodenum	Alfalfa hay	Positive	migration of cells	3.87E-05	296
Duodenum	Alfalfa hay	Positive	chemotaxis of cells	3.87E-05	98
Duodenum	Alfalfa hay	Positive	proliferation of connective tissue cells	4.33E-05	108
Duodenum	Alfalfa hay	Positive	transactivation of RNA	5.16E-05	100
Duodenum	Alfalfa hay	Positive	apoptosis of cervical cancer cell lines	5.16E-05	58
Duodenum	Alfalfa hay	Positive	neuromuscular disease	6.19E-05	151
Duodenum	Alfalfa	Positive	invasion of cells	6.19E-05	149

	hay				
Duodenum	Alfalfa hay	Positive	activation of DNA endogenous promoter	6.74E-05	198
Duodenum	Alfalfa hay	Positive	angiogenesis	7.47E-05	154
Duodenum	Alfalfa hay	Positive	tumorigenesis of tissue	8.12E-05	1211
Duodenum	Alfalfa hay	Positive	development of body trunk	8.32E-05	184
Duodenum	Alfalfa hay	Positive	neuritogenesis	8.68E-05	105
Duodenum	Alfalfa hay	Positive	development of neurons	9.27E-05	135
Duodenum	Alfalfa hay	Positive	growth of connective tissue	1.03E-04	114
Duodenum	Alfalfa hay	Positive	seizure disorder	1.06E-04	87
Duodenum	Alfalfa hay	Positive	cell death of cervical cancer cell lines	1.11E-04	68
Duodenum	Alfalfa hay	Positive	formation of cytoskeleton	1.14E-04	72
Duodenum	Alfalfa hay	Positive	formation of plasma membrane projections	1.44E-04	106
Duodenum	Alfalfa hay	Positive	development of cytoplasm	1.64E-04	85
Duodenum	Alfalfa hay	Positive	degeneration of nervous system	1.77E-04	57
Duodenum	Alfalfa hay	Positive	quantity of cells	2.02E-04	272
Duodenum	Alfalfa hay	Positive	degeneration of cells	2.02E-04	66
Duodenum	Alfalfa hay	Positive	neuronal cell death	2.14E-04	118
Duodenum	Alfalfa hay	Positive	cell survival	2.15E-04	220
Duodenum	Alfalfa hay	Positive	cell death of connective tissue cells	2.23E-04	106
Duodenum	Alfalfa hay	Positive	apoptosis of connective tissue cells	2.27E-04	58
Duodenum	Alfalfa hay	Positive	cell spreading of fibroblast cell lines	2.31E-04	17
Duodenum	Alfalfa hay	Positive	Growth Failure	2.61E-04	105
Duodenum	Alfalfa	Positive	growth of neurites	2.65E-04	84

	hay				
Duodenum	Alfalfa hay	Positive	abnormal morphology of thoracic cavity	2.81E-04	103
Duodenum	Alfalfa hay	Positive	transport of carbohydrate	2.88E-04	41
Duodenum	Alfalfa hay	Positive	cell movement of tumor cell lines	3.37E-04	142
Duodenum	Alfalfa hay	Positive	branching of cells	3.56E-04	69
Duodenum	Alfalfa hay	Positive	shape change of fibroblast cell lines	3.89E-04	23
Duodenum	Alfalfa hay	Positive	epilepsy	3.95E-04	59
Duodenum	Alfalfa hay	Positive	metabolism of reactive oxygen species	3.97E-04	89
Duodenum	Alfalfa hay	Positive	Organ Degeneration	3.97E-04	88
Duodenum	Alfalfa hay	Positive	congenital malformation of skeleton	4.16E-04	77
Duodenum	Alfalfa hay	Positive	frequency of head and neck tumor	5.03E-04	6
Duodenum	Alfalfa hay	Positive	morphogenesis of neurons	5.03E-04	77
Duodenum	Alfalfa hay	Positive	vasculogenesis	5.27E-04	124
Duodenum	Alfalfa hay	Positive	synthesis of reactive oxygen species	6.53E-04	85
Duodenum	Alfalfa hay	Positive	morphology of connective tissue	6.61E-04	82
Duodenum	Alfalfa hay	Positive	organization of organelle	6.61E-04	94
Duodenum	Alfalfa hay	Positive	morphology of cardiovascular system	6.86E-04	106
Duodenum	Alfalfa hay	Positive	quantity of blood cells	7.73E-04	165
Duodenum	Alfalfa hay	Positive	degeneration of neurons	7.73E-04	47
Duodenum	Alfalfa hay	Positive	cell viability	7.82E-04	203
Duodenum	Alfalfa hay	Positive	morphogenesis of neurites	8.51E-04	75
Duodenum	Alfalfa hay	Positive	colony formation	8.51E-04	93
Duodenum	Alfalfa	Positive	size of animal	8.65E-04	37

	hay				
Duodenum	Alfalfa hay	Positive	colony formation of cells	9.55E-04	86
Duodenum	Alfalfa hay	Positive	craniofacial abnormality	9.75E-04	65
Duodenum	Alfalfa hay	Positive	outgrowth of cells	9.75E-04	77
Duodenum	Alfalfa hay	Positive	cellular degradation	9.75E-04	49
Duodenum	Alfalfa hay	Positive	adhesion of carcinoma cell lines	9.75E-04	13
Duodenum	Alfalfa hay	Positive	Edema	1.08E-03	63
Duodenum	Alfalfa hay	Positive	quantity of leukocytes	1.11E-03	148
Duodenum	Alfalfa hay	Positive	outgrowth of neurites	1.26E-03	72
Duodenum	Alfalfa hay	Positive	formation of filaments	1.26E-03	71
Duodenum	Alfalfa hay	Positive	abnormal morphology of vascular endothelial cells	1.29E-03	13
Duodenum	Alfalfa hay	Positive	shape change of kidney cell lines	1.29E-03	17
Duodenum	Alfalfa hay	Positive	fibrogenesis	1.30E-03	73
Duodenum	Alfalfa hay	Positive	cell spreading	1.34E-03	52
Duodenum	Alfalfa hay	Positive	abnormal morphology of epithelial tissue	1.34E-03	74
Duodenum	Alfalfa hay	Positive	development of vasculature	1.34E-03	74
Duodenum	Alfalfa hay	Positive	sprouting	1.36E-03	69
Duodenum	Alfalfa hay	Positive	transformation of fibroblast cell lines	1.36E-03	49
Duodenum	Alfalfa hay	Positive	digestive system cancer	1.36E-03	1008
Duodenum	Alfalfa hay	Positive	Neurodegeneration	1.37E-03	57
Duodenum	Alfalfa hay	Positive	abnormal morphology of cells	1.39E-03	196
Duodenum	Alfalfa hay	Positive	synthesis of lipid	1.43E-03	118
Duodenum	Alfalfa	Positive	transport of monosaccharide	1.46E-03	33

	hay				
Duodenum	Alfalfa hay	Positive	perinatal death	1.64E-03	100
Duodenum	Alfalfa hay	Positive	function of cardiovascular system	1.68E-03	66
Duodenum	Alfalfa hay	Positive	abnormal quantity of lipid	1.69E-03	31
Duodenum	Alfalfa hay	Positive	cell death of cerebral cortex cells	1.83E-03	44
Duodenum	Alfalfa hay	Positive	digestive organ tumor	1.95E-03	1015
Duodenum	Alfalfa hay	Positive	apoptosis of epithelial cell lines	2.05E-03	43
Duodenum	Alfalfa hay	Positive	production of reactive oxygen species	2.14E-03	65
Duodenum	Alfalfa hay	Positive	growth of lesion	2.18E-03	129
Duodenum	Alfalfa hay	Positive	cell death of embryonic stem cells	2.22E-03	14
Duodenum	Alfalfa hay	Positive	oxidation of palmitic acid	2.22E-03	14
Duodenum	Alfalfa hay	Positive	formation of cells	2.53E-03	155
Duodenum	Alfalfa hay	Positive	cell transformation	2.59E-03	79
Duodenum	Alfalfa hay	Positive	morphology of endothelial cells	2.79E-03	16
Duodenum	Alfalfa hay	Positive	oral cancer	2.82E-03	43
Duodenum	Alfalfa hay	Positive	cell death of brain	2.84E-03	53
Duodenum	Alfalfa hay	Positive	cell movement of phagocytes	2.85E-03	92
Duodenum	Alfalfa hay	Positive	growth of tumor	2.89E-03	128
Duodenum	Alfalfa hay	Positive	cell movement of macrophages	2.96E-03	49
Duodenum	Alfalfa hay	Positive	quantity of TNF in blood	3.00E-03	19
Duodenum	Alfalfa hay	Positive	cell death of brain cells	3.00E-03	50
Duodenum	Alfalfa hay	Positive	transport of water	3.09E-03	9
Duodenum	Alfalfa	Positive	adjuvant arthritis	3.26E-03	11

	hay				
Duodenum	Alfalfa hay	Positive	fatty acid oxidation disorder	3.35E-03	10
Duodenum	Alfalfa hay	Positive	inflammatory response	3.40E-03	125
Duodenum	Alfalfa hay	Positive	cell movement of leukocytes	3.81E-03	123
Duodenum	Alfalfa hay	Positive	abnormal morphology of cardiovascular system	3.81E-03	94
Duodenum	Alfalfa hay	Positive	necrosis of epithelial tissue	3.96E-03	97
Duodenum	Alfalfa hay	Positive	cell death of epithelial cell lines	3.96E-03	51
Duodenum	Alfalfa hay	Positive	congenital anomaly of digestive system	3.96E-03	43
Duodenum	Alfalfa hay	Positive	quantity of thymocytes	3.96E-03	43
Duodenum	Alfalfa hay	Positive	cycling of centrosome	4.10E-03	20
Duodenum	Alfalfa hay	Positive	morphology of cardiovascular tissue	4.10E-03	20
Duodenum	Alfalfa hay	Positive	morphology of vascular endothelial cells	4.12E-03	14
Duodenum	Alfalfa hay	Positive	branching of neurites	4.43E-03	50
Duodenum	Alfalfa hay	Positive	abnormal morphology of endothelial tissue	4.62E-03	16
Duodenum	Alfalfa hay	Positive	cell death of epithelial cells	4.71E-03	83
Duodenum	Alfalfa hay	Positive	shape change of neurites	4.77E-03	51
Duodenum	Alfalfa hay	Positive	abdominal neoplasm	4.78E-03	1163
Duodenum	Alfalfa hay	Positive	transport of D-glucose	4.78E-03	29
Duodenum	Alfalfa hay	Positive	midline defect	4.79E-03	48
Duodenum	Alfalfa hay	Positive	formation of actin filaments	4.80E-03	54
Duodenum	Alfalfa hay	Positive	formation of thymus gland	4.85E-03	32
Duodenum	Alfalfa hay	Positive	autosomal recessive disease	5.10E-03	144
Duodenum	Alfalfa	Positive	concentration of fatty acid	5.25E-03	50

	hay				
Duodenum	Alfalfa hay	Positive	osmotic water permeability of cells	5.34E-03	4
Duodenum	Alfalfa hay	Positive	cell movement of myeloid cells	5.34E-03	89
Duodenum	Alfalfa hay	Positive	inborn error of lipid metabolism	5.34E-03	11
Duodenum	Alfalfa hay	Positive	oral squamous cell carcinoma	5.69E-03	34
Duodenum	Alfalfa hay	Positive	development of head	5.82E-03	152
Duodenum	Alfalfa hay	Positive	migration of tumor cell lines	5.86E-03	113
Duodenum	Alfalfa hay	Positive	cell death of hippocampal cells	5.89E-03	21
Duodenum	Alfalfa hay	Positive	cell spreading of kidney cell lines	5.95E-03	9
Duodenum	Alfalfa hay	Positive	cell death of immune cells	6.36E-03	106
Duodenum	Alfalfa hay	Positive	morphology of body cavity	6.39E-03	181
Duodenum	Alfalfa hay	Positive	differentiation of connective tissue	6.54E-03	116
Duodenum	Alfalfa hay	Positive	multiple congenital anomalies	6.69E-03	71
Duodenum	Alfalfa hay	Positive	oxidation of fatty acid	6.75E-03	33
Duodenum	Alfalfa hay	Positive	Viral Infection	7.33E-03	237
Duodenum	Alfalfa hay	Positive	proliferation of fibroblast cell lines	7.40E-03	71
Duodenum	Alfalfa hay	Positive	cell movement of carcinoma cell lines	7.67E-03	38
Duodenum	Alfalfa hay	Positive	cell death of hippocampal neurons	7.93E-03	19
Duodenum	Alfalfa hay	Positive	abdominal cancer	7.93E-03	1147
Duodenum	Alfalfa hay	Positive	neutrophilia	8.04E-03	15
Duodenum	Alfalfa hay	Positive	attachment of cells	8.10E-03	27
Duodenum	Alfalfa hay	Positive	consumption of oxygen	8.21E-03	30
Duodenum	Alfalfa	Positive	morphology of nervous system	8.21E-03	123

	hay				
Duodenum	Alfalfa hay	Positive	quantity of connective tissue	8.32E-03	97
Duodenum	Alfalfa hay	Positive	axonogenesis	8.32E-03	41
Duodenum	Alfalfa hay	Positive	invasion of tumor cell lines	8.35E-03	106
Duodenum	Alfalfa hay	Positive	oxidation of long chain fatty acid	8.42E-03	16
Duodenum	Alfalfa hay	Positive	formation of actin stress fibers	8.69E-03	43
Duodenum	Alfalfa hay	Positive	familial dementia	8.94E-03	12
Duodenum	Alfalfa hay	Positive	cell death of breast cancer cell lines	9.24E-03	57
Duodenum	Alfalfa hay	Positive	leukocyte migration	9.41E-03	135
Duodenum	Alfalfa hay	Positive	mass of organism	9.77E-03	57
Duodenum	Alfalfa hay	Positive	transport of carboxylic acid	1.00E-02	24
Duodenum	Alfalfa hay	Positive	oral cavity carcinoma	1.00E-02	36
Duodenum	Alfalfa hay	Positive	mass of adipose tissue	1.03E-02	35
Duodenum	Alfalfa hay	Positive	fatty acid metabolism	1.08E-02	96
Duodenum	Alfalfa hay	Positive	concentration of lipid	1.08E-02	126
Duodenum	Alfalfa hay	Positive	familial Alzheimer's disease	1.08E-02	9
Duodenum	Alfalfa hay	Positive	abnormal morphology of head	1.09E-02	129
Duodenum	Alfalfa hay	Positive	differentiation of connective tissue cells	1.09E-02	102
Duodenum	Alfalfa hay	Positive	migration of carcinoma cell lines	1.10E-02	32
Duodenum	Alfalfa hay	Positive	morphology of head	1.12E-02	135
Duodenum	Alfalfa hay	Positive	hydrolysis of GTP	1.12E-02	16
Duodenum	Alfalfa hay	Positive	small GTPase mediated signal transduction	1.12E-02	34
Duodenum	Alfalfa	Positive	proliferation of embryonic cell lines	1.12E-02	30

	hay				
Duodenum	Alfalfa hay	Positive	size of embryo	1.13E-02	60
Duodenum	Alfalfa hay	Positive	Bleeding	1.14E-02	70
Duodenum	Alfalfa hay	Positive	autosomal dominant disease	1.14E-02	101
Duodenum	Alfalfa hay	Positive	quantity of mononuclear leukocytes	1.14E-02	115
Duodenum	Alfalfa hay	Positive	quantity of lymphocytes	1.14E-02	111
Duodenum	Alfalfa hay	Positive	cell death of blood cells	1.14E-02	109
Duodenum	Alfalfa hay	Positive	paralysis	1.18E-02	31
Duodenum	Alfalfa hay	Positive	cellular infiltration by macrophages	1.20E-02	30
Duodenum	Alfalfa hay	Positive	homo-oligomerization of protein	1.22E-02	26
Duodenum	Alfalfa hay	Positive	neonatal death	1.23E-02	72
Duodenum	Alfalfa hay	Positive	morphology of heart	1.24E-02	67
Duodenum	Alfalfa hay	Positive	morphology of respiratory system	1.24E-02	53
Duodenum	Alfalfa hay	Positive	cell death of fibroblasts	1.24E-02	46
Duodenum	Alfalfa hay	Positive	accumulation of reactive oxygen species	1.24E-02	18
Duodenum	Alfalfa hay	Positive	morphology of endothelial tissue	1.24E-02	18
Duodenum	Alfalfa hay	Positive	metabolism of protein	1.24E-02	148
Duodenum	Alfalfa hay	Positive	barrier function of skin	1.25E-02	11
Duodenum	Alfalfa hay	Positive	morphology of stratum corneum	1.25E-02	11
Duodenum	Alfalfa hay	Positive	ruffling	1.31E-02	22
Duodenum	Alfalfa hay	Positive	quantity of lipid peroxide	1.31E-02	7
Duodenum	Alfalfa hay	Positive	cell death of fibroblast cell lines	1.31E-02	70
Duodenum	Alfalfa	Positive	behavior	1.31E-02	150

	hay				
Duodenum	Alfalfa hay	Positive	proliferation of fibroblasts	1.33E-02	59
Duodenum	Alfalfa hay	Positive	formation of skin	1.36E-02	61
Duodenum	Alfalfa hay	Positive	secretion of molecule	1.36E-02	88
Duodenum	Alfalfa hay	Positive	thermogenesis	1.38E-02	15
Duodenum	Alfalfa hay	Positive	development of abdomen	1.40E-02	92
Duodenum	Alfalfa hay	Positive	abnormal morphology of respiratory system	1.43E-02	52
Duodenum	Alfalfa hay	Positive	autophagy	1.47E-02	62
Duodenum	Alfalfa hay	Positive	abnormal morphology of stratum corneum	1.48E-02	10
Duodenum	Alfalfa hay	Positive	arrest in proliferation of cells	1.50E-02	38
Duodenum	Alfalfa hay	Positive	quantity of lymphoid organ	1.51E-02	52
Duodenum	Alfalfa hay	Positive	mass of connective tissue	1.52E-02	36
Duodenum	Alfalfa hay	Positive	fusion of cellular membrane	1.52E-02	14
Duodenum	Alfalfa hay	Positive	apoptosis of melanoma cell lines	1.52E-02	25
Duodenum	Alfalfa hay	Positive	development of body axis	1.56E-02	158
Duodenum	Alfalfa hay	Positive	cell viability of tumor cell lines	1.56E-02	120
Duodenum	Alfalfa hay	Positive	cell movement of antigen presenting cells	1.61E-02	58
Duodenum	Alfalfa hay	Positive	abnormal morphology of body cavity	1.61E-02	170
Duodenum	Alfalfa hay	Positive	abnormal morphology of mandibular angle	1.61E-02	4
Duodenum	Alfalfa hay	Positive	frequency of brain tumor	1.61E-02	4
Duodenum	Alfalfa hay	Positive	targeting of protein	1.65E-02	25
Duodenum	Alfalfa hay	Positive	proliferation of epidermal cells	1.65E-02	31
Duodenum	Alfalfa	Positive	development of genitourinary system	1.68E-02	135

	hay				
Duodenum	Alfalfa hay	Positive	abnormal morphology of nervous system	1.77E-02	112
Duodenum	Alfalfa hay	Positive	cell death of hepatoma cell lines	1.80E-02	31
Duodenum	Alfalfa hay	Positive	beta-oxidation of fatty acid	1.80E-02	17
Duodenum	Alfalfa hay	Positive	cell movement of fibroblasts	1.81E-02	33
Duodenum	Alfalfa hay	Positive	morphology of gonad	1.81E-02	62
Duodenum	Alfalfa hay	Positive	internalization of lipid	1.83E-02	8
Duodenum	Alfalfa hay	Positive	exocytosis	1.86E-02	34
Duodenum	Alfalfa hay	Positive	secretory pathway	1.92E-02	35
Duodenum	Alfalfa hay	Positive	transport of glutamine family amino acid	1.94E-02	13
Duodenum	Alfalfa hay	Positive	ubiquitination of protein	1.94E-02	55
Duodenum	Alfalfa hay	Positive	phosphorylation of protein	2.06E-02	113
Duodenum	Alfalfa hay	Positive	angiosarcoma	2.08E-02	16
Duodenum	Alfalfa hay	Positive	apoptosis of hepatoma cell lines	2.08E-02	28
Duodenum	Alfalfa hay	Positive	cell cycle progression	2.17E-02	156
Duodenum	Alfalfa hay	Positive	formation of lymphocytes	2.17E-02	24
Duodenum	Alfalfa hay	Positive	morphology of bone	2.21E-02	72
Duodenum	Alfalfa hay	Positive	activation of JUN kinase	2.22E-02	11
Duodenum	Alfalfa hay	Positive	activation of enzyme	2.22E-02	63
Duodenum	Alfalfa hay	Positive	survival of vascular endothelial cells	2.24E-02	10
Duodenum	Alfalfa hay	Positive	congenital malformation of face	2.24E-02	28
Duodenum	Alfalfa hay	Positive	septic shock	2.24E-02	28
Duodenum	Alfalfa	Positive	morphology of vessel	2.25E-02	51

	hay				
Duodenum	Alfalfa hay	Positive	apoptosis of cerebral cortex cells	2.26E-02	21
Duodenum	Alfalfa hay	Positive	cyclopia	2.26E-02	6
Duodenum	Alfalfa hay	Positive	morphology of vasculature	2.27E-02	30
Duodenum	Alfalfa hay	Positive	cell death of smooth muscle cells	2.29E-02	22
Duodenum	Alfalfa hay	Positive	apoptosis of fibroblast cell lines	2.38E-02	54
Duodenum	Alfalfa hay	Positive	oxidation of lipid	2.39E-02	37
Duodenum	Alfalfa hay	Positive	proliferation of keratinocytes	2.39E-02	28
Duodenum	Alfalfa hay	Positive	morphology of genital organ	2.39E-02	68
Duodenum	Alfalfa hay	Positive	frequency of malignant tumor	2.41E-02	5
Duodenum	Alfalfa hay	Positive	apoptosis of breast cancer cell lines	2.41E-02	48
Duodenum	Alfalfa hay	Positive	development of digestive system	2.42E-02	62
Duodenum	Alfalfa hay	Positive	S phase	2.46E-02	44
Duodenum	Alfalfa hay	Positive	frequency of tumor	2.47E-02	7
Duodenum	Alfalfa hay	Positive	cell movement of lung cancer cell lines	2.50E-02	22
Duodenum	Alfalfa hay	Positive	quantity of lymphatic system component	2.59E-02	61
Duodenum	Alfalfa hay	Positive	S phase of fibroblast cell lines	2.59E-02	13
Duodenum	Alfalfa hay	Positive	arrest in proliferation of fibroblast cell lines	2.59E-02	11
Duodenum	Alfalfa hay	Positive	high bone mass disease	2.60E-02	16
Duodenum	Alfalfa hay	Positive	shape change of epithelial cell lines	2.62E-02	9
Duodenum	Alfalfa hay	Positive	polyarticular juvenile rheumatoid arthritis	2.69E-02	18
Duodenum	Alfalfa hay	Positive	cell death of muscle	2.73E-02	56
Duodenum	Alfalfa	Positive	apoptosis of embryonic cell lines	2.77E-02	32

	hay				
Duodenum	Alfalfa hay	Positive	proliferation of cervical cancer cell lines	2.77E-02	35
Duodenum	Alfalfa hay	Positive	congenital anomaly of musculoskeletal system	2.78E-02	112
Duodenum	Alfalfa hay	Positive	cell movement of connective tissue cells	2.88E-02	38
Duodenum	Alfalfa hay	Positive	cell movement of neutrophils	2.91E-02	49
Duodenum	Alfalfa hay	Positive	bleeding of tissue	3.01E-02	13
Duodenum	Alfalfa hay	Positive	formation of T lymphocytes	3.01E-02	22
Duodenum	Alfalfa hay	Positive	aggregation of cells	3.02E-02	53
Duodenum	Alfalfa hay	Positive	transport of L-amino acid	3.04E-02	14
Duodenum	Alfalfa hay	Positive	entry into S phase of fibroblast cell lines	3.04E-02	8
Duodenum	Alfalfa hay	Positive	apoptosis of neurons	3.05E-02	69
Duodenum	Alfalfa hay	Positive	cell death of melanoma cell lines	3.05E-02	42
Duodenum	Alfalfa hay	Positive	metabolism of membrane lipid derivative	3.10E-02	62
Duodenum	Alfalfa hay	Positive	sensitivity of cells	3.11E-02	26
Duodenum	Alfalfa hay	Positive	cell death of stem cells	3.14E-02	17
Duodenum	Alfalfa hay	Positive	abnormal morphology of bone	3.15E-02	69
Duodenum	Alfalfa hay	Positive	cell movement of endothelial cells	3.17E-02	57
Duodenum	Alfalfa hay	Positive	autophagy of cells	3.17E-02	44
Duodenum	Alfalfa hay	Positive	quantity of type II pneumocytes	3.20E-02	6
Duodenum	Alfalfa hay	Positive	frequency of thymic lymphoma	3.20E-02	3
Duodenum	Alfalfa hay	Positive	structural integrity of intercellular junctions	3.20E-02	3
Duodenum	Alfalfa hay	Positive	juvenile rheumatoid arthritis	3.20E-02	23
Duodenum	Alfalfa	Positive	cellular infiltration of phagocytes	3.20E-02	31

	hay				
Duodenum	Alfalfa hay	Positive	quantity of reactive oxygen species	3.20E-02	31
Duodenum	Alfalfa hay	Positive	abnormal morphology of second branchial arch	3.20E-02	7
Duodenum	Alfalfa hay	Positive	adhesion of sarcoma cell lines	3.20E-02	7
Duodenum	Alfalfa hay	Positive	formation of cerebral ventricles	3.20E-02	7
Duodenum	Alfalfa hay	Positive	fusion of vertebrae	3.20E-02	12
Duodenum	Alfalfa hay	Positive	congenital anomaly of mouth	3.20E-02	29
Duodenum	Alfalfa hay	Positive	neurodegeneration of neurites	3.20E-02	18
Duodenum	Alfalfa hay	Positive	morphology of skin	3.21E-02	45
Duodenum	Alfalfa hay	Positive	morphology of reproductive system	3.22E-02	83
Duodenum	Alfalfa hay	Positive	apoptosis of fibroblasts	3.25E-02	37
Duodenum	Alfalfa hay	Positive	transport of alpha-amino acid	3.25E-02	15
Duodenum	Alfalfa hay	Positive	apoptosis of kidney cell lines	3.25E-02	40
Duodenum	Alfalfa hay	Positive	fragmentation of DNA fragment	3.25E-02	13
Duodenum	Alfalfa hay	Positive	cell death of dermal cells	3.25E-02	19
Duodenum	Alfalfa hay	Positive	abnormal morphology of cervix	3.25E-02	4
Duodenum	Alfalfa hay	Positive	advanced angiosarcoma	3.25E-02	4
Duodenum	Alfalfa hay	Positive	elongation of fibroblast cell lines	3.25E-02	4
Duodenum	Alfalfa hay	Positive	extrahepatic bile duct cancer	3.25E-02	4
Duodenum	Alfalfa hay	Positive	import of L-glutamic acid	3.25E-02	4
Duodenum	Alfalfa hay	Positive	metastatic triple negative breast adenocarcinoma	3.25E-02	4
Duodenum	Alfalfa hay	Positive	resectable gallbladder cancer	3.25E-02	4
Duodenum	Alfalfa	Positive	stage 4 HER2 negative hormone receptor	3.25E-02	4

	hay		negative breast cancer		
Duodenum	Alfalfa hay	Positive	stage 4 triple negative breast adenocarcinoma	3.25E-02	4
Duodenum	Alfalfa hay	Positive	unresectable pancreatic cancer	3.25E-02	4
Duodenum	Alfalfa hay	Positive	cell death of skin	3.26E-02	20
Duodenum	Alfalfa hay	Positive	mammary tumor	3.26E-02	234
Duodenum	Alfalfa hay	Positive	differentiation of epidermal cells	3.29E-02	29
Duodenum	Alfalfa hay	Positive	dendritic growth/branching	3.33E-02	37
Duodenum	Alfalfa hay	Positive	growth of organism	3.35E-02	125
Duodenum	Alfalfa hay	Positive	differentiation of blood cells	3.41E-02	126
Duodenum	Alfalfa hay	Positive	intermediate disease stage peripheral arterial disease	3.41E-02	28
Duodenum	Alfalfa hay	Positive	cell death of muscle cells	3.41E-02	54
Duodenum	Alfalfa hay	Positive	breast or colorectal cancer	3.42E-02	670
Duodenum	Alfalfa hay	Positive	generation of reactive oxygen species	3.47E-02	35
Duodenum	Alfalfa hay	Positive	necrosis of muscle	3.49E-02	55
Duodenum	Alfalfa hay	Positive	abnormal morphology of skull	3.49E-02	37
Duodenum	Alfalfa hay	Positive	oligomerization of protein	3.49E-02	40
Duodenum	Alfalfa hay	Positive	grasping	3.49E-02	15
Duodenum	Alfalfa hay	Positive	abnormal quantity of fatty acid	3.49E-02	8
Duodenum	Alfalfa hay	Positive	hydrolysis of nucleotide	3.49E-02	23
Duodenum	Alfalfa hay	Positive	metabolism of carnitine	3.49E-02	5
Duodenum	Alfalfa hay	Positive	secretion of alpha granules	3.49E-02	5
Duodenum	Alfalfa hay	Positive	formation of thymocytes	3.49E-02	20
Duodenum	Alfalfa	Positive	internalization by tumor cell lines	3.50E-02	22

	hay				
Duodenum	Alfalfa hay	Positive	interphase of fibroblast cell lines	3.50E-02	22
Duodenum	Alfalfa hay	Positive	Shock Response	3.54E-02	30
Duodenum	Alfalfa hay	Positive	formation of leukocytes	3.57E-02	28
Duodenum	Alfalfa hay	Positive	abnormal morphology of gonad	3.64E-02	58
Duodenum	Alfalfa hay	Positive	catabolism of protein	3.69E-02	92
Duodenum	Alfalfa hay	Positive	internalization of cells	3.69E-02	29
Duodenum	Alfalfa hay	Positive	apoptosis of muscle	3.77E-02	43
Duodenum	Alfalfa hay	Positive	homing of blood cells	3.77E-02	60
Duodenum	Alfalfa hay	Positive	morphology of blood vessel	3.83E-02	46
Duodenum	Alfalfa hay	Positive	leukocytosis	3.83E-02	21
Duodenum	Alfalfa hay	Positive	cell viability of cervical cancer cell lines	3.86E-02	37
Duodenum	Alfalfa hay	Positive	protein kinase cascade	3.91E-02	66
Duodenum	Alfalfa hay	Positive	adhesion of lung cancer cell lines	3.93E-02	7
Duodenum	Alfalfa hay	Positive	dephosphorylation of protein	3.93E-02	32
Duodenum	Alfalfa hay	Positive	mitosis of tumor cell lines	4.01E-02	24
Duodenum	Alfalfa hay	Positive	differentiation of smooth muscle	4.01E-02	14
Duodenum	Alfalfa hay	Positive	dilation of heart ventricle	4.01E-02	12
Duodenum	Alfalfa hay	Positive	differentiation of epithelial tissue	4.03E-02	57
Duodenum	Alfalfa hay	Positive	mass of genitourinary system	4.05E-02	48
Duodenum	Alfalfa hay	Positive	quantity of D-sphingosine	4.05E-02	6
Duodenum	Alfalfa hay	Positive	tubulogenesis of endothelial cells	4.05E-02	6
Duodenum	Alfalfa	Positive	abnormal morphology of vasculature	4.06E-02	28

	hay				
Duodenum	Alfalfa hay	Positive	migration of connective tissue cells	4.07E-02	31
Duodenum	Alfalfa hay	Positive	polarization of tumor cell lines	4.12E-02	10
Duodenum	Alfalfa hay	Positive	interphase	4.27E-02	99
Duodenum	Alfalfa hay	Positive	transport of amino acids	4.30E-02	24
Duodenum	Alfalfa hay	Positive	morphology of second branchial arch	4.30E-02	8
Duodenum	Alfalfa hay	Positive	transport of L-glutamic acid	4.30E-02	8
Duodenum	Alfalfa hay	Positive	oxidative stress	4.32E-02	15
Duodenum	Alfalfa hay	Positive	metabolism of carbohydrate	4.33E-02	94
Duodenum	Alfalfa hay	Positive	movement of vascular endothelial cells	4.33E-02	31
Duodenum	Alfalfa hay	Positive	cell movement of epithelial cells	4.34E-02	26
Duodenum	Alfalfa hay	Positive	accumulation of lipid	4.41E-02	48
Duodenum	Alfalfa hay	Positive	apoptosis of retinal cells	4.43E-02	11
Duodenum	Alfalfa hay	Positive	chemotaxis of leukocytes	4.43E-02	56
Duodenum	Alfalfa hay	Positive	development of antigen presenting cells	4.45E-02	14
Duodenum	Alfalfa hay	Positive	abnormal morphology of pulmonary alveolus	4.45E-02	21
Duodenum	Alfalfa hay	Positive	chemotaxis of myeloid cells	4.45E-02	47
Duodenum	Alfalfa hay	Positive	formation of focal adhesions	4.46E-02	27
Duodenum	Alfalfa hay	Positive	benign neoplasia	4.47E-02	136
Duodenum	Alfalfa hay	Positive	differentiation of leukocytes	4.47E-02	101
Duodenum	Alfalfa hay	Positive	death of perinatal stage organism	4.47E-02	16
Duodenum	Alfalfa hay	Positive	formation of microtubules	4.47E-02	16
Duodenum	Alfalfa	Positive	oxidative stress response of cells	4.47E-02	13

	hay				
Duodenum	Alfalfa hay	Positive	quantity of subcutaneous fat	4.47E-02	13
Duodenum	Alfalfa hay	Positive	abnormal morphology of dorsal aorta	4.47E-02	12
Duodenum	Alfalfa hay	Positive	learning	4.51E-02	65
Duodenum	Alfalfa hay	Positive	abnormal morphology of heart	4.63E-02	60
Duodenum	Alfalfa hay	Positive	development of epithelial tissue	4.64E-02	79
Duodenum	Alfalfa hay	Positive	hypoplasia of organ	4.65E-02	69
Duodenum	Alfalfa hay	Positive	abnormal morphology of blood vessel	4.66E-02	43
Duodenum	Alfalfa hay	Positive	homing of leukocytes	4.73E-02	59
Duodenum	Alfalfa hay	Positive	abnormal quantity of leukocytes	4.73E-02	18
Duodenum	Alfalfa hay	Positive	transport of acidic amino acid	4.73E-02	10
Duodenum	Alfalfa hay	Positive	migration of endothelial cells	4.73E-02	52
Duodenum	Alfalfa hay	Positive	mitosis	4.73E-02	76
Duodenum	Alfalfa hay	Positive	cell viability of fibroblast cell lines	4.73E-02	21
Duodenum	Alfalfa hay	Positive	abnormal morphology of abdomen	4.74E-02	129
Duodenum	Alfalfa hay	Positive	apoptosis of smooth muscle cells	4.76E-02	20
Duodenum	Alfalfa hay	Positive	cognition	4.79E-02	70
Duodenum	Alfalfa hay	Positive	morphology of cerebral neocortex	4.81E-02	7
Duodenum	Alfalfa hay	Positive	proliferation of embryonic stem cell lines	4.81E-02	7
Duodenum	Alfalfa hay	Positive	transport of monocarboxylic acid	4.81E-02	7
Duodenum	Alfalfa hay	Positive	metabolism of DNA	4.82E-02	62
Duodenum	Alfalfa hay	Positive	recruitment of cells	4.82E-02	56
Duodenum	Alfalfa	Positive	growth of skin	4.82E-02	36

	hay				
Duodenum	Alfalfa hay	Positive	cell viability of cerebral cortex cells	4.82E-02	14
Duodenum	Alfalfa hay	Positive	organization of actin cytoskeleton	4.83E-02	48
Duodenum	Alfalfa hay	Positive	morphology of astrocytes	4.87E-02	11
Duodenum	Alfalfa hay	Positive	remodeling of embryonic tissue	4.87E-02	11
Duodenum	Alfalfa hay	Positive	cell spreading of dermal cells	4.87E-02	5
Duodenum	Alfalfa hay	Positive	import of lipid	4.87E-02	5
Duodenum	Alfalfa hay	Positive	locally advanced adenocarcinoma	4.87E-02	5
Duodenum	Alfalfa hay	Positive	quantity of carnitine	4.87E-02	5
Duodenum	Alfalfa hay	Positive	synthesis of GABA	4.87E-02	5
Duodenum	Alfalfa hay	Positive	vascularization of hindlimb	4.87E-02	5
Duodenum	Alfalfa hay	Positive	apoptosis of brain cells	4.87E-02	25
Duodenum	Alfalfa hay	Positive	migration of fibroblasts	4.87E-02	25
Duodenum	Alfalfa hay	Positive	contraction of muscle cells	4.91E-02	12
Duodenum	Alfalfa hay	Positive	development of face	4.91E-02	12
Duodenum	Alfalfa hay	Positive	morphology of right ventricle	4.91E-02	12
Duodenum	Alfalfa hay	Positive	quantity of lung cells	4.95E-02	8
Duodenum	Alfalfa hay	Positive	synthesis of heme	4.95E-02	8
Duodenum	Alfalfa hay	Positive	apoptosis of muscle cells	4.97E-02	42
Duodenum	Alfalfa hay	Positive	gluconeogenesis	4.98E-02	18
Duodenum	Alfalfa hay	Positive	formation of embryonic tissue	5.04E-02	47
Duodenum	Alfalfa hay	Positive	Zellweger syndrome	5.10E-02	6
Duodenum	Alfalfa	Positive	cell spreading of epithelial cells	5.10E-02	6

	hay				
Duodenum	Alfalfa hay	Positive	differentiation of muscle	5.17E-02	48
Duodenum	Alfalfa hay	Positive	aggregation of blood platelets	5.20E-02	32
Duodenum	Alfalfa hay	Positive	morphogenesis of axons	5.20E-02	16
Duodenum	Alfalfa hay	Positive	adhesion of fibrosarcoma cell lines	5.20E-02	4
Duodenum	Alfalfa hay	Positive	advanced stage biliary tract cancer	5.20E-02	4
Duodenum	Alfalfa hay	Positive	antineutrophil cytoplasmic antibody-associated vasculitis	5.20E-02	4
Duodenum	Alfalfa hay	Positive	generation of plasma membrane projections	5.20E-02	4
Duodenum	Alfalfa hay	Positive	locally advanced pancreatic adenocarcinoma	5.20E-02	4
Duodenum	Alfalfa hay	Positive	morphology of thyroid tumor cell lines	5.20E-02	4
Duodenum	Alfalfa hay	Positive	pyknosis	5.20E-02	4
Duodenum	Alfalfa hay	Positive	synthesis of pyrimidine ribonucleotide	5.20E-02	4
Duodenum	Alfalfa hay	Positive	unresectable pancreatic adenocarcinoma	5.20E-02	4
Duodenum	Alfalfa hay	Positive	cell death of kidney cancer cell lines	5.25E-02	10
Duodenum	Alfalfa hay	Positive	abnormal morphology of artery	5.27E-02	26
Duodenum	Alfalfa hay	Positive	differentiation of keratinocytes	5.27E-02	26
Duodenum	Alfalfa hay	Positive	mental retardation	5.36E-02	38
Duodenum	Alfalfa hay	Positive	synthesis of terpenoid	5.37E-02	45
Duodenum	Alfalfa hay	Positive	abnormal morphology of genital organ	5.39E-02	63
Duodenum	Alfalfa hay	Positive	spinocerebellar disease	5.44E-02	23
Duodenum	Alfalfa hay	Positive	formation of centrosome	5.45E-02	12
Duodenum	Alfalfa hay	Positive	involution	5.45E-02	12
Duodenum	Alfalfa	Positive	neurodegeneration of cerebral cortex	5.45E-02	12

	hay				
Duodenum	Alfalfa hay	Positive	tubulogenesis	5.45E-02	12
Duodenum	Alfalfa hay	Positive	uptake of monosaccharide	5.53E-02	43
Duodenum	Alfalfa hay	Positive	homing of tumor cell lines	5.63E-02	26
Duodenum	Alfalfa hay	Positive	contraction of cardiomyocytes	5.66E-02	9
Duodenum	Alfalfa hay	Positive	epithelial-mesenchymal transition	5.77E-02	34
Duodenum	Alfalfa hay	Positive	quantity of alveolar epithelium	5.79E-02	7
Duodenum	Alfalfa hay	Positive	quantity of pneumocytes	5.79E-02	7
Duodenum	Alfalfa hay	Positive	degeneration of central nervous system	5.86E-02	23
Duodenum	Alfalfa hay	Positive	endotoxemia	5.86E-02	18
Duodenum	Alfalfa hay	Positive	abnormal morphology of first branchial arch	5.88E-02	8
Duodenum	Alfalfa hay	Positive	permeability of cellular membrane	5.88E-02	8
Duodenum	Alfalfa hay	Positive	thrombosis of artery	5.88E-02	8
Duodenum	Alfalfa hay	Positive	development of central nervous system	5.88E-02	95
Duodenum	Alfalfa hay	Positive	osteopetrosis	6.03E-02	13
Duodenum	Alfalfa hay	Positive	transmembrane potential of mitochondria	6.03E-02	35
Duodenum	Alfalfa hay	Positive	function of muscle	6.15E-02	54
Duodenum	Alfalfa hay	Positive	migration of vascular endothelial cells	6.27E-02	28
Duodenum	Alfalfa hay	Positive	organization of fibrils	6.27E-02	11
Duodenum	Alfalfa hay	Positive	epithelial-mesenchymal transition of tumor cell lines	6.29E-02	20
Duodenum	Alfalfa hay	Positive	dysgenesis	6.33E-02	81
Duodenum	Alfalfa hay	Positive	morphology of testis	6.34E-02	43
Duodenum	Alfalfa	Positive	shape change of axons	6.34E-02	17

	hay				
Duodenum	Alfalfa hay	Positive	differentiation of skin	6.34E-02	30
Duodenum	Alfalfa hay	Positive	apoptosis of vascular endothelial cells	6.34E-02	18
Duodenum	Alfalfa hay	Positive	mitosis of cervical cancer cell lines	6.34E-02	18
Duodenum	Alfalfa hay	Positive	differentiation of epithelial cells	6.34E-02	49
Duodenum	Alfalfa hay	Positive	development of urinary tract	6.34E-02	45
Duodenum	Alfalfa hay	Positive	morphology of lung	6.34E-02	31
Duodenum	Alfalfa hay	Positive	abnormal morphology of skin	6.37E-02	39
Duodenum	Alfalfa hay	Positive	abnormal morphology of branchial arch	6.37E-02	14
Duodenum	Alfalfa hay	Positive	uptake of amino acids	6.37E-02	14
Duodenum	Alfalfa hay	Positive	Cytosis	6.44E-02	50
Duodenum	Alfalfa hay	Positive	respiratory system development	6.46E-02	53
Duodenum	Alfalfa hay	Positive	advanced soft tissue sarcoma	6.46E-02	6
Duodenum	Alfalfa hay	Positive	metabolism of ceramide	6.46E-02	6
Duodenum	Alfalfa hay	Positive	relapsed chronic lymphocytic leukemia	6.46E-02	6
Duodenum	Alfalfa hay	Positive	Hypoplasia	6.46E-02	76
Duodenum	Alfalfa hay	Positive	polymerization of protein	6.48E-02	57
Duodenum	Alfalfa hay	Negative	proliferation of cells	1.89E-60	1791
Duodenum	Alfalfa hay	Negative	cancer	2.67E-48	4440
Duodenum	Alfalfa hay	Negative	cell death	1.08E-47	1594
Duodenum	Alfalfa hay	Negative	organismal death	3.83E-47	1202
Duodenum	Alfalfa hay	Negative	malignant solid tumor	7.56E-47	4386
Duodenum	Alfalfa	Negative	morbidity or mortality	7.56E-47	1214

	hay				
Duodenum	Alfalfa hay	Negative	apoptosis	3.06E-38	1269
Duodenum	Alfalfa hay	Negative	morphology of cells	8.33E-37	972
Duodenum	Alfalfa hay	Negative	necrosis	1.60E-36	1232
Duodenum	Alfalfa hay	Negative	cell movement	3.82E-35	1042
Duodenum	Alfalfa hay	Negative	organization of cytoplasm	2.03E-33	782
Duodenum	Alfalfa hay	Negative	organization of cytoskeleton	6.84E-28	702
Duodenum	Alfalfa hay	Negative	transport of molecule	1.66E-27	793
Duodenum	Alfalfa hay	Negative	tumorigenesis of tissue	1.24E-26	3671
Duodenum	Alfalfa hay	Negative	differentiation of cells	2.19E-26	1054
Duodenum	Alfalfa hay	Negative	neoplasia of epithelial tissue	5.09E-26	3612
Duodenum	Alfalfa hay	Negative	migration of cells	2.74E-25	905
Duodenum	Alfalfa hay	Negative	epithelial cancer	5.56E-25	3574
Duodenum	Alfalfa hay	Negative	abdominal neoplasm	7.90E-24	3573
Duodenum	Alfalfa hay	Negative	quantity of cells	8.17E-24	842
Duodenum	Alfalfa hay	Negative	expression of RNA	3.44E-23	995
Duodenum	Alfalfa hay	Negative	abdominal cancer	1.55E-22	3526
Duodenum	Alfalfa hay	Negative	microtubule dynamics	5.96E-22	587
Duodenum	Alfalfa hay	Negative	transcription	7.18E-22	926
Duodenum	Alfalfa hay	Negative	cellular homeostasis	1.07E-21	729
Duodenum	Alfalfa hay	Negative	angiogenesis	9.39E-21	462
Duodenum	Alfalfa hay	Negative	cell movement of tumor cell lines	2.35E-20	436
Duodenum	Alfalfa	Negative	Movement Disorders	3.84E-20	526

	hay				
Duodenum	Alfalfa hay	Negative	concentration of lipid	3.97E-20	417
Duodenum	Alfalfa hay	Negative	Viral Infection	4.69E-20	759
Duodenum	Alfalfa hay	Negative	cell survival	5.21E-20	667
Duodenum	Alfalfa hay	Negative	transcription of RNA	7.33E-20	859
Duodenum	Alfalfa hay	Negative	abnormal morphology of cells	9.24E-20	612
Duodenum	Alfalfa hay	Negative	development of body trunk	1.53E-19	548
Duodenum	Alfalfa hay	Negative	organization of organelle	3.36E-19	290
Duodenum	Alfalfa hay	Negative	cell viability	6.28E-19	623
Duodenum	Alfalfa hay	Negative	cell death of tumor cell lines	6.98E-19	717
Duodenum	Alfalfa hay	Negative	digestive system cancer	8.66E-19	3054
Duodenum	Alfalfa hay	Negative	vasculogenesis	1.37E-18	378
Duodenum	Alfalfa hay	Negative	digestive organ tumor	1.44E-18	3079
Duodenum	Alfalfa hay	Negative	size of body	1.29E-17	425
Duodenum	Alfalfa hay	Negative	growth of organism	1.75E-17	418
Duodenum	Alfalfa hay	Negative	invasion of cells	2.64E-17	432
Duodenum	Alfalfa hay	Negative	transcription of DNA	4.20E-17	712
Duodenum	Alfalfa hay	Negative	quantity of blood cells	4.84E-17	499
Duodenum	Alfalfa hay	Negative	formation of cellular protrusions	5.42E-17	445
Duodenum	Alfalfa hay	Negative	quantity of leukocytes	8.29E-17	450
Duodenum	Alfalfa hay	Negative	proliferation of tumor cell lines	2.58E-16	713
Duodenum	Alfalfa hay	Negative	morphology of cardiovascular system	3.02E-16	317
Duodenum	Alfalfa	Negative	synthesis of lipid	7.01E-16	358

	hay				
Duodenum	Alfalfa hay	Negative	morphology of body cavity	8.11E-16	564
Duodenum	Alfalfa hay	Negative	apoptosis of tumor cell lines	1.15E-15	570
Duodenum	Alfalfa hay	Negative	abnormal morphology of body cavity	2.03E-15	540
Duodenum	Alfalfa hay	Negative	development of cytoplasm	2.97E-15	242
Duodenum	Alfalfa hay	Negative	cell death of connective tissue cells	5.27E-15	305
Duodenum	Alfalfa hay	Negative	migration of tumor cell lines	1.08E-14	350
Duodenum	Alfalfa hay	Negative	perinatal death	1.14E-14	300
Duodenum	Alfalfa hay	Negative	phosphorylation of protein	2.75E-14	361
Duodenum	Alfalfa hay	Negative	abnormal morphology of thoracic cavity	3.05E-14	295
Duodenum	Alfalfa hay	Negative	formation of cytoskeleton	3.79E-14	199
Duodenum	Alfalfa hay	Negative	disorder of basal ganglia	4.08E-14	374
Duodenum	Alfalfa hay	Negative	transactivation	6.49E-14	290
Duodenum	Alfalfa hay	Negative	protein kinase cascade	7.05E-14	219
Duodenum	Alfalfa hay	Negative	dyskinesia	1.37E-13	306
Duodenum	Alfalfa hay	Negative	Huntington's Disease	1.55E-13	287
Duodenum	Alfalfa hay	Negative	autosomal recessive disease	1.55E-13	438
Duodenum	Alfalfa hay	Negative	fibrogenesis	1.96E-13	213
Duodenum	Alfalfa hay	Negative	proliferation of connective tissue cells	3.12E-13	294
Duodenum	Alfalfa hay	Negative	neurological signs	4.87E-13	319
Duodenum	Alfalfa hay	Negative	abnormal morphology of cardiovascular system	4.88E-13	282
Duodenum	Alfalfa hay	Negative	growth of embryo	6.73E-13	241
Duodenum	Alfalfa	Negative	formation of filaments	7.07E-13	205

	hay				
Duodenum	Alfalfa hay	Negative	metabolism of carbohydrate	1.31E-12	304
Duodenum	Alfalfa hay	Negative	invasion of tumor cell lines	2.90E-12	322
Duodenum	Alfalfa hay	Negative	function of blood cells	3.89E-12	287
Duodenum	Alfalfa hay	Negative	ubiquitination	3.93E-12	175
Duodenum	Alfalfa hay	Negative	neuromuscular disease	3.96E-12	415
Duodenum	Alfalfa hay	Negative	degeneration of nervous system	4.06E-12	152
Duodenum	Alfalfa hay	Negative	function of leukocytes	4.17E-12	265
Duodenum	Alfalfa hay	Negative	homing of cells	4.27E-12	274
Duodenum	Alfalfa hay	Negative	morphology of heart	4.37E-12	206
Duodenum	Alfalfa hay	Negative	quantity of lymphocytes	5.41E-12	339
Duodenum	Alfalfa hay	Negative	ubiquitination of protein	6.18E-12	172
Duodenum	Alfalfa hay	Negative	transactivation of RNA	6.57E-12	267
Duodenum	Alfalfa hay	Negative	growth of connective tissue	7.25E-12	311
Duodenum	Alfalfa hay	Negative	cell viability of tumor cell lines	7.81E-12	369
Duodenum	Alfalfa hay	Negative	quantity of mononuclear leukocytes	9.31E-12	350
Duodenum	Alfalfa hay	Negative	Neurodegeneration	9.64E-12	161
Duodenum	Alfalfa hay	Negative	degeneration of cells	9.64E-12	177
Duodenum	Alfalfa hay	Negative	quantity of connective tissue	1.15E-11	292
Duodenum	Alfalfa hay	Negative	neuronal cell death	1.30E-11	326
Duodenum	Alfalfa hay	Negative	behavior	1.30E-11	457
Duodenum	Alfalfa hay	Negative	concentration of fatty acid	1.36E-11	147
Duodenum	Alfalfa	Negative	homing	1.36E-11	279

	hay				
Duodenum	Alfalfa hay	Negative	replication of RNA virus	1.43E-11	249
Duodenum	Alfalfa hay	Negative	growth of lesion	2.06E-11	375
Duodenum	Alfalfa hay	Negative	benign neoplasia	2.26E-11	429
Duodenum	Alfalfa hay	Negative	cell cycle progression	2.94E-11	479
Duodenum	Alfalfa hay	Negative	differentiation of connective tissue	3.28E-11	345
Duodenum	Alfalfa hay	Negative	growth of tumor	4.05E-11	373
Duodenum	Alfalfa hay	Negative	replication of virus	5.49E-11	271
Duodenum	Alfalfa hay	Negative	quantity of carbohydrate	5.75E-11	252
Duodenum	Alfalfa hay	Negative	chemotaxis of cells	5.85E-11	256
Duodenum	Alfalfa hay	Negative	differentiation of connective tissue cells	7.16E-11	306
Duodenum	Alfalfa hay	Negative	learning	8.11E-11	207
Duodenum	Alfalfa hay	Negative	cellular degradation	9.78E-11	133
Duodenum	Alfalfa hay	Negative	activation of DNA endogenous promoter	1.07E-10	543
Duodenum	Alfalfa hay	Negative	degeneration of neurons	1.07E-10	126
Duodenum	Alfalfa hay	Negative	Growth Failure	1.07E-10	286
Duodenum	Alfalfa hay	Negative	chemotaxis	1.57E-10	262
Duodenum	Alfalfa hay	Negative	cognition	1.68E-10	222
Duodenum	Alfalfa hay	Negative	development of vasculature	1.87E-10	206
Duodenum	Alfalfa hay	Negative	abnormal morphology of head	1.95E-10	385
Duodenum	Alfalfa hay	Negative	morphology of head	2.18E-10	403
Duodenum	Alfalfa hay	Negative	abnormal morphology of heart	2.30E-10	190
Duodenum	Alfalfa	Negative	seizures	2.36E-10	197

	hay				
Duodenum	Alfalfa hay	Negative	abnormal morphology of abdomen	2.44E-10	403
Duodenum	Alfalfa hay	Negative	accumulation of lipid	2.47E-10	152
Duodenum	Alfalfa hay	Negative	breast or colorectal cancer	2.54E-10	2031
Duodenum	Alfalfa hay	Negative	necrosis of epithelial tissue	2.60E-10	280
Duodenum	Alfalfa hay	Negative	seizure disorder	2.75E-10	228
Duodenum	Alfalfa hay	Negative	proliferation of fibroblasts	2.98E-10	176
Duodenum	Alfalfa hay	Negative	uptake of monosaccharide	3.94E-10	138
Duodenum	Alfalfa hay	Negative	proliferation of blood cells	4.88E-10	387
Duodenum	Alfalfa hay	Negative	quantity of T lymphocytes	6.73E-10	255
Duodenum	Alfalfa hay	Negative	uptake of carbohydrate	6.77E-10	147
Duodenum	Alfalfa hay	Negative	secretion of molecule	6.84E-10	262
Duodenum	Alfalfa hay	Negative	migration of blood cells	7.22E-10	399
Duodenum	Alfalfa hay	Negative	morphology of respiratory system	8.00E-10	156
Duodenum	Alfalfa hay	Negative	abnormal morphology of respiratory system	8.11E-10	154
Duodenum	Alfalfa hay	Negative	leukocyte migration	8.53E-10	398
Duodenum	Alfalfa hay	Negative	neuritogenesis	9.94E-10	275
Duodenum	Alfalfa hay	Negative	fatty acid metabolism	1.36E-09	282
Duodenum	Alfalfa hay	Negative	activation of cells	1.44E-09	441
Duodenum	Alfalfa hay	Negative	formation of plasma membrane projections	1.48E-09	280
Duodenum	Alfalfa hay	Negative	endocytosis	1.56E-09	170
Duodenum	Alfalfa hay	Negative	proliferation of neuronal cells	1.63E-09	271
Duodenum	Alfalfa	Negative	Organ Degeneration	1.78E-09	235

	hay				
Duodenum	Alfalfa hay	Negative	differentiation of leukocytes	1.86E-09	312
Duodenum	Alfalfa hay	Negative	abnormal morphology of digestive system	1.88E-09	257
Duodenum	Alfalfa hay	Negative	morphology of nervous system	2.01E-09	359
Duodenum	Alfalfa hay	Negative	development of leukocytes	2.02E-09	276
Duodenum	Alfalfa hay	Negative	concentration of phospholipid	2.15E-09	97
Duodenum	Alfalfa hay	Negative	proliferation of fibroblast cell lines	2.32E-09	204
Duodenum	Alfalfa hay	Negative	development of neurons	2.37E-09	358
Duodenum	Alfalfa hay	Negative	development of blood cells	2.44E-09	303
Duodenum	Alfalfa hay	Negative	synthesis of DNA	2.54E-09	201
Duodenum	Alfalfa hay	Negative	cell movement of leukocytes	2.95E-09	351
Duodenum	Alfalfa hay	Negative	binding of DNA	2.95E-09	246
Duodenum	Alfalfa hay	Negative	differentiation of blood cells	3.34E-09	383
Duodenum	Alfalfa hay	Negative	engulfment of cells	3.63E-09	196
Duodenum	Alfalfa hay	Negative	neonatal death	3.83E-09	210
Duodenum	Alfalfa hay	Negative	phosphorylation of L-amino acid	3.93E-09	113
Duodenum	Alfalfa hay	Negative	morphology of digestive system	4.05E-09	267
Duodenum	Alfalfa hay	Negative	infection by Retroviridae	4.37E-09	330
Duodenum	Alfalfa hay	Negative	survival of organism	4.75E-09	323
Duodenum	Alfalfa hay	Negative	female genital tract serous cancer	4.94E-09	186
Duodenum	Alfalfa hay	Negative	development of lymphocytes	4.95E-09	259
Duodenum	Alfalfa hay	Negative	growth of epithelial tissue	5.13E-09	313
Duodenum	Alfalfa	Negative	development of epithelial tissue	5.33E-09	243

	hay				
Duodenum	Alfalfa hay	Negative	cell death of fibroblast cell lines	5.57E-09	204
Duodenum	Alfalfa hay	Negative	activation of enzyme	5.69E-09	187
Duodenum	Alfalfa hay	Negative	development of lymphatic system	5.79E-09	173
Duodenum	Alfalfa hay	Negative	sprouting	5.82E-09	186
Duodenum	Alfalfa hay	Negative	metabolism of protein	6.28E-09	435
Duodenum	Alfalfa hay	Negative	quantity of lymphatic system component	6.58E-09	182
Duodenum	Alfalfa hay	Negative	breast or ovarian cancer	6.99E-09	815
Duodenum	Alfalfa hay	Negative	development of mononuclear leukocytes	7.28E-09	260
Duodenum	Alfalfa hay	Negative	cell transformation	7.65E-09	218
Duodenum	Alfalfa hay	Negative	serous neoplasm	8.47E-09	282
Duodenum	Alfalfa hay	Negative	proliferation of immune cells	8.50E-09	359
Duodenum	Alfalfa hay	Negative	cell movement of myeloid cells	8.93E-09	252
Duodenum	Alfalfa hay	Negative	HIV infection	1.04E-08	325
Duodenum	Alfalfa hay	Negative	gastrointestinal tract cancer	1.06E-08	2048
Duodenum	Alfalfa hay	Negative	cell death of epithelial cells	1.10E-08	233
Duodenum	Alfalfa hay	Negative	interphase	1.18E-08	301
Duodenum	Alfalfa hay	Negative	abnormal morphology of nervous system	1.18E-08	330
Duodenum	Alfalfa hay	Negative	infection by RNA virus	1.38E-08	389
Duodenum	Alfalfa hay	Negative	peripheral vascular disease	1.39E-08	182
Duodenum	Alfalfa hay	Negative	branching of cells	1.64E-08	177
Duodenum	Alfalfa hay	Negative	small GTPase mediated signal transduction	1.64E-08	95
Duodenum	Alfalfa	Negative	formation of lymphatic system component	1.67E-08	143

	hay				
Duodenum	Alfalfa hay	Negative	morphology of muscle	1.69E-08	162
Duodenum	Alfalfa hay	Negative	colony formation of cells	1.70E-08	230
Duodenum	Alfalfa hay	Negative	homeostasis of leukocytes	1.73E-08	257
Duodenum	Alfalfa hay	Negative	Gastrointestinal Tract Cancer and Tumors	1.82E-08	2077
Duodenum	Alfalfa hay	Negative	formation of cells	1.91E-08	436
Duodenum	Alfalfa hay	Negative	morphology of pulmonary alveolus	2.27E-08	65
Duodenum	Alfalfa hay	Negative	advanced malignant tumor	2.27E-08	379
Duodenum	Alfalfa hay	Negative	cell movement of phagocytes	2.30E-08	254
Duodenum	Alfalfa hay	Negative	formation of actin filaments	2.31E-08	148
Duodenum	Alfalfa hay	Negative	apoptosis of connective tissue cells	2.50E-08	144
Duodenum	Alfalfa hay	Negative	Lymphocyte homeostasis	2.50E-08	252
Duodenum	Alfalfa hay	Negative	epileptic seizure	2.78E-08	87
Duodenum	Alfalfa hay	Negative	quantity of lymphoid organ	2.78E-08	149
Duodenum	Alfalfa hay	Negative	autosomal dominant disease	2.79E-08	291
Duodenum	Alfalfa hay	Negative	development of cardiovascular tissue	2.89E-08	176
Duodenum	Alfalfa hay	Negative	synthesis of carbohydrate	2.96E-08	200
Duodenum	Alfalfa hay	Negative	colony formation	2.96E-08	248
Duodenum	Alfalfa hay	Negative	proliferation of embryonic cells	3.13E-08	123
Duodenum	Alfalfa hay	Negative	quantity of reactive oxygen species	3.31E-08	91
Duodenum	Alfalfa hay	Negative	adenocarcinoma	3.42E-08	2598
Duodenum	Alfalfa hay	Negative	proliferation of mononuclear leukocytes	3.42E-08	333
Duodenum	Alfalfa	Negative	metabolism of nucleic acid component or	3.65E-08	240

	hay		derivative		
Duodenum	Alfalfa hay	Negative	T cell development	3.67E-08	233
Duodenum	Alfalfa hay	Negative	proliferation of lymphocytes	3.87E-08	328
Duodenum	Alfalfa hay	Negative	development of lymphatic system component	4.12E-08	149
Duodenum	Alfalfa hay	Negative	gastrointestinal carcinoma	4.40E-08	1836
Duodenum	Alfalfa hay	Negative	metabolism of membrane lipid derivative	4.62E-08	183
Duodenum	Alfalfa hay	Negative	concentration of acylglycerol	4.62E-08	160
Duodenum	Alfalfa hay	Negative	arthropathy	4.93E-08	421
Duodenum	Alfalfa hay	Negative	replication of Influenza virus	4.95E-08	140
Duodenum	Alfalfa hay	Negative	apoptosis of fibroblast cell lines	4.99E-08	157
Duodenum	Alfalfa hay	Negative	development of abdomen	5.52E-08	265
Duodenum	Alfalfa hay	Negative	development of head	6.48E-08	432
Duodenum	Alfalfa hay	Negative	formation of actin stress fibers	6.49E-08	118
Duodenum	Alfalfa hay	Negative	mass of organism	7.05E-08	159
Duodenum	Alfalfa hay	Negative	infection of cells	7.06E-08	356
Duodenum	Alfalfa hay	Negative	arthritis	8.31E-08	413
Duodenum	Alfalfa hay	Negative	uterine serous papillary cancer	8.38E-08	114
Duodenum	Alfalfa hay	Negative	abnormal morphology of reproductive system	8.67E-08	234
Duodenum	Alfalfa hay	Negative	T cell homeostasis	8.73E-08	237
Duodenum	Alfalfa hay	Negative	dyspnea	9.25E-08	71
Duodenum	Alfalfa hay	Negative	synthesis of fatty acid	9.25E-08	149
Duodenum	Alfalfa hay	Negative	abnormal morphology of epithelial tissue	9.70E-08	195
Duodenum	Alfalfa	Negative	replication of Influenza A virus	9.73E-08	138

	hay				
Duodenum	Alfalfa hay	Negative	long-term potentiation	1.03E-07	119
Duodenum	Alfalfa hay	Negative	inflammatory response	1.11E-07	347
Duodenum	Alfalfa hay	Negative	abnormal morphology of embryonic tissue	1.12E-07	250
Duodenum	Alfalfa hay	Negative	dephosphorylation of protein	1.14E-07	94
Duodenum	Alfalfa hay	Negative	development of endothelial tissue	1.15E-07	172
Duodenum	Alfalfa hay	Negative	formation of lymphoid organ	1.15E-07	128
Duodenum	Alfalfa hay	Negative	development of body axis	1.17E-07	459
Duodenum	Alfalfa hay	Negative	accumulation of cells	1.17E-07	166
Duodenum	Alfalfa hay	Negative	infection by HIV-1	1.23E-07	276
Duodenum	Alfalfa hay	Negative	growth of plasma membrane projections	1.25E-07	215
Duodenum	Alfalfa hay	Negative	size of animal	1.35E-07	90
Duodenum	Alfalfa hay	Negative	export of molecule	1.35E-07	141
Duodenum	Alfalfa hay	Negative	morphology of reproductive system	1.46E-07	244
Duodenum	Alfalfa hay	Negative	S phase	1.47E-07	126
Duodenum	Alfalfa hay	Negative	function of cardiovascular system	1.57E-07	173
Duodenum	Alfalfa hay	Negative	growth of embryonic tissue	1.65E-07	132
Duodenum	Alfalfa hay	Negative	endothelial cell development	1.67E-07	166
Duodenum	Alfalfa hay	Negative	formation of leukocytes	1.73E-07	81
Duodenum	Alfalfa hay	Negative	cell movement of breast cancer cell lines	1.77E-07	138
Duodenum	Alfalfa hay	Negative	congenital anomaly of musculoskeletal system	1.77E-07	328
Duodenum	Alfalfa hay	Negative	formation of lung	1.91E-07	143
Duodenum	Alfalfa	Negative	organization of actin cytoskeleton	1.91E-07	143

	hay				
Duodenum	Alfalfa hay	Negative	abnormal morphology of pulmonary alveolus	2.02E-07	61
Duodenum	Alfalfa hay	Negative	uptake of D-hexose	2.15E-07	109
Duodenum	Alfalfa hay	Negative	cell viability of cervical cancer cell lines	2.15E-07	108
Duodenum	Alfalfa hay	Negative	metabolism of nucleotide	2.16E-07	202
Duodenum	Alfalfa hay	Negative	development of connective tissue	2.19E-07	173
Duodenum	Alfalfa hay	Negative	function of muscle	2.19E-07	163
Duodenum	Alfalfa hay	Negative	metabolism of reactive oxygen species	2.24E-07	228
Duodenum	Alfalfa hay	Negative	abdominal adenocarcinoma	2.24E-07	2359
Duodenum	Alfalfa hay	Negative	dysmyelination	2.30E-07	70
Duodenum	Alfalfa hay	Negative	morphogenesis of neurons	2.35E-07	196
Duodenum	Alfalfa hay	Negative	morphology of vessel	2.65E-07	145
Duodenum	Alfalfa hay	Negative	morphology of connective tissue	2.68E-07	211
Duodenum	Alfalfa hay	Negative	synthesis of reactive oxygen species	2.93E-07	219
Duodenum	Alfalfa hay	Negative	cytopenia	2.95E-07	141
Duodenum	Alfalfa hay	Negative	vascularization	3.30E-07	114
Duodenum	Alfalfa hay	Negative	morphology of bone	3.35E-07	206
Duodenum	Alfalfa hay	Negative	quantity of phagocytes	3.38E-07	195
Duodenum	Alfalfa hay	Negative	ion homeostasis of cells	3.51E-07	251
Duodenum	Alfalfa hay	Negative	growth of neurites	3.61E-07	211
Duodenum	Alfalfa hay	Negative	growth of muscle tissue	3.61E-07	173
Duodenum	Alfalfa hay	Negative	metastasis	3.61E-07	336
Duodenum	Alfalfa	Negative	abnormal morphology of bone	3.69E-07	200

	hay				
Duodenum	Alfalfa hay	Negative	proliferation of muscle cells	3.74E-07	172
Duodenum	Alfalfa hay	Negative	function of phagocytes	3.97E-07	144
Duodenum	Alfalfa hay	Negative	cardiogenesis	4.01E-07	215
Duodenum	Alfalfa hay	Negative	mammary tumor	4.18E-07	691
Duodenum	Alfalfa hay	Negative	uptake of D-glucose	4.32E-07	107
Duodenum	Alfalfa hay	Negative	invasion of prostate cancer cell lines	4.38E-07	55
Duodenum	Alfalfa hay	Negative	morphogenesis of neurites	4.42E-07	192
Duodenum	Alfalfa hay	Negative	quantity of adipose tissue	4.46E-07	132
Duodenum	Alfalfa hay	Negative	synthesis of nitric oxide	4.56E-07	131
Duodenum	Alfalfa hay	Negative	morphology of lymphatic system component	4.57E-07	191
Duodenum	Alfalfa hay	Negative	dysgenesis	5.05E-07	243
Duodenum	Alfalfa hay	Negative	quantity of steroid	5.28E-07	212
Duodenum	Alfalfa hay	Negative	quantity of hematopoietic cells	5.28E-07	202
Duodenum	Alfalfa hay	Negative	organization of filaments	5.55E-07	100
Duodenum	Alfalfa hay	Negative	cell death of muscle cells	5.57E-07	156
Duodenum	Alfalfa hay	Negative	activation of Protein kinase	5.73E-07	120
Duodenum	Alfalfa hay	Negative	hypersensitive reaction	5.88E-07	196
Duodenum	Alfalfa hay	Negative	cell death of blood cells	6.14E-07	307
Duodenum	Alfalfa hay	Negative	Dermatitis	6.27E-07	198
Duodenum	Alfalfa hay	Negative	quantity of hematopoietic progenitor cells	6.38E-07	201
Duodenum	Alfalfa hay	Negative	cell death of epithelial cell lines	6.43E-07	133
Duodenum	Alfalfa	Negative	colorectal neoplasia	6.45E-07	1750

	hay				
Duodenum	Alfalfa hay	Negative	differentiation of epithelial tissue	6.45E-07	166
Duodenum	Alfalfa hay	Negative	apoptosis of muscle	6.45E-07	124
Duodenum	Alfalfa hay	Negative	quantity of metal	6.70E-07	225
Duodenum	Alfalfa hay	Negative	formation of skin	6.77E-07	169
Duodenum	Alfalfa hay	Negative	secretory pathway	6.90E-07	96
Duodenum	Alfalfa hay	Negative	mitosis	6.98E-07	224
Duodenum	Alfalfa hay	Negative	morphology of muscle cells	7.37E-07	82
Duodenum	Alfalfa hay	Negative	cell death of cervical cancer cell lines	7.43E-07	162
Duodenum	Alfalfa hay	Negative	proliferation of smooth muscle cells	7.49E-07	134
Duodenum	Alfalfa hay	Negative	concentration of phosphatidic acid	7.79E-07	69
Duodenum	Alfalfa hay	Negative	intestinal tumor	7.92E-07	1754
Duodenum	Alfalfa hay	Negative	apoptosis of muscle cells	8.01E-07	123
Duodenum	Alfalfa hay	Negative	congenital anomaly of cardiovascular system	8.18E-07	109
Duodenum	Alfalfa hay	Negative	cell death of muscle	8.23E-07	159
Duodenum	Alfalfa hay	Negative	Hypertrophy	8.27E-07	214
Duodenum	Alfalfa hay	Negative	peripheral arterial disease	8.64E-07	102
Duodenum	Alfalfa hay	Negative	morphology of skin	8.66E-07	128
Duodenum	Alfalfa hay	Negative	invasion of carcinoma cell lines	8.66E-07	96
Duodenum	Alfalfa hay	Negative	MAPKKK cascade	8.66E-07	99
Duodenum	Alfalfa hay	Negative	neurodegeneration of neurites	8.79E-07	49
Duodenum	Alfalfa hay	Negative	Hypoplasia	8.97E-07	227
Duodenum	Alfalfa	Negative	plasticity of synapse	9.01E-07	62

	hay				
Duodenum	Alfalfa hay	Negative	differentiation of mononuclear leukocytes	9.80E-07	242
Duodenum	Alfalfa hay	Negative	quantity of thymus gland	9.81E-07	111
Duodenum	Alfalfa hay	Negative	quantity of thymocytes	9.93E-07	110
Duodenum	Alfalfa hay	Negative	necrosis of muscle	9.96E-07	158
Duodenum	Alfalfa hay	Negative	concentration of Ca2+	1.14E-06	65
Duodenum	Alfalfa hay	Negative	chemotaxis of myeloid cells	1.24E-06	136
Duodenum	Alfalfa hay	Negative	morphology of cardiac muscle	1.26E-06	82
Duodenum	Alfalfa hay	Negative	allergy	1.29E-06	184
Duodenum	Alfalfa hay	Negative	intestinal cancer	1.29E-06	1726
Duodenum	Alfalfa hay	Negative	cell movement of connective tissue cells	1.31E-06	106
Duodenum	Alfalfa hay	Negative	colorectal cancer	1.32E-06	1723
Duodenum	Alfalfa hay	Negative	apoptosis of neurons	1.32E-06	197
Duodenum	Alfalfa hay	Negative	gonadal tumor	1.37E-06	357
Duodenum	Alfalfa hay	Negative	quantity of glycosphingolipid	1.37E-06	52
Duodenum	Alfalfa hay	Negative	cell movement of fibroblasts	1.37E-06	89
Duodenum	Alfalfa hay	Negative	migration of breast cancer cell lines	1.37E-06	116
Duodenum	Alfalfa hay	Negative	colon cancer	1.37E-06	1495
Duodenum	Alfalfa hay	Negative	morphology of lung	1.38E-06	91
Duodenum	Alfalfa hay	Negative	synthesis of nucleotide	1.42E-06	166
Duodenum	Alfalfa hay	Negative	activation of leukocytes	1.43E-06	307
Duodenum	Alfalfa hay	Negative	cell spreading	1.48E-06	129
Duodenum	Alfalfa	Negative	quantity of antigen presenting cells	1.48E-06	129

	hay				
Duodenum	Alfalfa hay	Negative	quantity of protein in blood	1.50E-06	227
Duodenum	Alfalfa hay	Negative	colon tumor	1.53E-06	1503
Duodenum	Alfalfa hay	Negative	Ovarian Cancer and Tumors	1.57E-06	331
Duodenum	Alfalfa hay	Negative	epilepsy	1.74E-06	142
Duodenum	Alfalfa hay	Negative	formation of muscle	1.74E-06	180
Duodenum	Alfalfa hay	Negative	accumulation of blood cells	1.74E-06	130
Duodenum	Alfalfa hay	Negative	hepatocellular carcinoma	1.84E-06	1682
Duodenum	Alfalfa hay	Negative	quantity of B lymphocytes	1.94E-06	158
Duodenum	Alfalfa hay	Negative	apoptosis of epithelial cell lines	2.01E-06	106
Duodenum	Alfalfa hay	Negative	neoplasia of colon	2.05E-06	1497
Duodenum	Alfalfa hay	Negative	morphology of blood vessel	2.05E-06	131
Duodenum	Alfalfa hay	Negative	colorectal carcinoma	2.09E-06	1494
Duodenum	Alfalfa hay	Negative	transmembrane potential of mitochondria	2.10E-06	102
Duodenum	Alfalfa hay	Negative	ruffling	2.12E-06	56
Duodenum	Alfalfa hay	Negative	mass of connective tissue	2.21E-06	96
Duodenum	Alfalfa hay	Negative	function of myeloid cells	2.23E-06	112
Duodenum	Alfalfa hay	Negative	demyelination	2.27E-06	61
Duodenum	Alfalfa hay	Negative	transport of protein	2.28E-06	139
Duodenum	Alfalfa hay	Negative	proliferation of hematopoietic cells	2.33E-06	119
Duodenum	Alfalfa hay	Negative	Rheumatic Disease	2.39E-06	460
Duodenum	Alfalfa hay	Negative	hepatobiliary system cancer	2.42E-06	1741
Duodenum	Alfalfa	Negative	metabolism of monosaccharide	2.42E-06	62

	hay				
Duodenum	Alfalfa hay	Negative	inflammation of organ	2.48E-06	499
Duodenum	Alfalfa hay	Negative	tumorigenesis of intestine	2.62E-06	1499
Duodenum	Alfalfa hay	Negative	abnormal morphology of muscle	2.62E-06	145
Duodenum	Alfalfa hay	Negative	differentiation of muscle	2.71E-06	139
Duodenum	Alfalfa hay	Negative	proliferation of T lymphocytes	2.71E-06	266
Duodenum	Alfalfa hay	Negative	tumorigenesis of gonad	2.80E-06	350
Duodenum	Alfalfa hay	Negative	growth of lymphatic system component	2.81E-06	92
Duodenum	Alfalfa hay	Negative	branching of neurons	2.83E-06	132
Duodenum	Alfalfa hay	Negative	activation of blood cells	2.84E-06	325
Duodenum	Alfalfa hay	Negative	apoptosis of cervical cancer cell lines	3.02E-06	130
Duodenum	Alfalfa hay	Negative	activation of neuroglia	3.02E-06	66
Duodenum	Alfalfa hay	Negative	differentiation of bone cells	3.19E-06	183
Duodenum	Alfalfa hay	Negative	proliferation of epithelial cells	3.21E-06	219
Duodenum	Alfalfa hay	Negative	formation of thymus gland	3.37E-06	79
Duodenum	Alfalfa hay	Negative	apoptosis of blood cells	3.50E-06	220
Duodenum	Alfalfa hay	Negative	formation of blood cells	3.52E-06	94
Duodenum	Alfalfa hay	Negative	cell death of neuroblastoma cell lines	3.56E-06	92
Duodenum	Alfalfa hay	Negative	glucose metabolism disorder	3.62E-06	541
Duodenum	Alfalfa hay	Negative	autophagy	3.71E-06	169
Duodenum	Alfalfa hay	Negative	apoptosis of pheochromocytoma cell lines	3.81E-06	48
Duodenum	Alfalfa hay	Negative	function of antigen presenting cells	3.88E-06	114
Duodenum	Alfalfa	Negative	abnormal morphology of skull	3.95E-06	103

	hay				
Duodenum	Alfalfa hay	Negative	infection of embryonic cell lines	3.95E-06	127
Duodenum	Alfalfa hay	Negative	infection of epithelial cell lines	3.95E-06	127
Duodenum	Alfalfa hay	Negative	abdominal carcinoma	3.95E-06	2375
Duodenum	Alfalfa hay	Negative	differentiation of bone	4.03E-06	184
Duodenum	Alfalfa hay	Negative	respiratory system development	4.06E-06	155
Duodenum	Alfalfa hay	Negative	cell movement of neutrophils	4.22E-06	136
Duodenum	Alfalfa hay	Negative	morphology of blood cells	4.25E-06	186
Duodenum	Alfalfa hay	Negative	morphology of lymphoid organ	4.26E-06	176
Duodenum	Alfalfa hay	Negative	metabolism of phospholipid	4.29E-06	109
Duodenum	Alfalfa hay	Negative	concentration of triacylglycerol	4.32E-06	140
Duodenum	Alfalfa hay	Negative	chemotaxis of phagocytes	4.32E-06	135
Duodenum	Alfalfa hay	Negative	hypertrophy of heart	4.36E-06	153
Duodenum	Alfalfa hay	Negative	mass of adipose tissue	4.45E-06	90
Duodenum	Alfalfa hay	Negative	proliferation of prostate cancer cell lines	4.46E-06	139
Duodenum	Alfalfa hay	Negative	ovarian cancer	4.47E-06	309
Duodenum	Alfalfa hay	Negative	abnormal morphology of skin	4.76E-06	113
Duodenum	Alfalfa hay	Negative	atrophy of muscle	4.93E-06	72
Duodenum	Alfalfa hay	Negative	modification of peptide	4.96E-06	54
Duodenum	Alfalfa hay	Negative	outgrowth of plasma membrane projections	5.02E-06	182
Duodenum	Alfalfa hay	Negative	adenoma	5.03E-06	243
Duodenum	Alfalfa hay	Negative	infection of kidney cell lines	5.08E-06	130
Duodenum	Alfalfa	Negative	quantity of metal ion	5.10E-06	197

	hay				
Duodenum	Alfalfa hay	Negative	homing of blood cells	5.46E-06	170
Duodenum	Alfalfa hay	Negative	neurodegeneration of axons	5.46E-06	45
Duodenum	Alfalfa hay	Negative	cell death of pheochromocytoma cell lines	5.46E-06	60
Duodenum	Alfalfa hay	Negative	Bleeding	5.58E-06	189
Duodenum	Alfalfa hay	Negative	branching of neurites	5.58E-06	127
Duodenum	Alfalfa hay	Negative	cell death of immune cells	5.61E-06	287
Duodenum	Alfalfa hay	Negative	abnormal morphology of snout	5.76E-06	36
Duodenum	Alfalfa hay	Negative	liver cancer	5.96E-06	1728
Duodenum	Alfalfa hay	Negative	liver tumor	6.13E-06	1739
Duodenum	Alfalfa hay	Negative	liver lesion	6.16E-06	1740
Duodenum	Alfalfa hay	Negative	quantity of cytokine	6.26E-06	117
Duodenum	Alfalfa hay	Negative	cell movement of endothelial cells	6.36E-06	159
Duodenum	Alfalfa hay	Negative	shape change of neurons	6.49E-06	133
Duodenum	Alfalfa hay	Negative	differentiation of epithelial cells	6.57E-06	142
Duodenum	Alfalfa hay	Negative	function of mononuclear leukocytes	6.67E-06	154
Duodenum	Alfalfa hay	Negative	intermediate disease stage peripheral arterial disease	6.86E-06	76
Duodenum	Alfalfa hay	Negative	chronic myeloid leukemia	6.92E-06	54
Duodenum	Alfalfa hay	Negative	engulfment of leukocytes	6.92E-06	77
Duodenum	Alfalfa hay	Negative	hereditary neuropathy	7.04E-06	79
Duodenum	Alfalfa hay	Negative	size of embryo	7.07E-06	160
Duodenum	Alfalfa hay	Negative	proliferation of hematopoietic progenitor cells	7.10E-06	112
Duodenum	Alfalfa	Negative	chronic inflammatory disorder	7.44E-06	414

	hay				
Duodenum	Alfalfa hay	Negative	differentiation of lymphocytes	7.54E-06	219
Duodenum	Alfalfa hay	Negative	abnormal morphology of lymphoid organ	7.55E-06	169
Duodenum	Alfalfa hay	Negative	quantity of trophoblast giant cells	7.66E-06	23
Duodenum	Alfalfa hay	Negative	repair of DNA	7.94E-06	132
Duodenum	Alfalfa hay	Negative	production of antibody	7.97E-06	141
Duodenum	Alfalfa hay	Negative	autophagy of cells	7.97E-06	121
Duodenum	Alfalfa hay	Negative	cell death of fibroblasts	7.97E-06	121
Duodenum	Alfalfa hay	Negative	function of lymphocytes	8.02E-06	153
Duodenum	Alfalfa hay	Negative	cell movement of carcinoma cell lines	8.02E-06	96
Duodenum	Alfalfa hay	Negative	formation of lymphocytes	8.07E-06	62
Duodenum	Alfalfa hay	Negative	binding of protein binding site	8.12E-06	131
Duodenum	Alfalfa hay	Negative	production of protein	8.34E-06	148
Duodenum	Alfalfa hay	Negative	morphology of skull	8.50E-06	104
Duodenum	Alfalfa hay	Negative	Hypertension	8.52E-06	278
Duodenum	Alfalfa hay	Negative	morphology of genital organ	8.59E-06	188
Duodenum	Alfalfa hay	Negative	formation of mononuclear leukocytes	8.73E-06	64
Duodenum	Alfalfa hay	Negative	quantity of sphingolipid	8.80E-06	53
Duodenum	Alfalfa hay	Negative	abnormal morphology of lung	8.82E-06	86
Duodenum	Alfalfa hay	Negative	engulfment of blood cells	8.89E-06	81
Duodenum	Alfalfa hay	Negative	release of L-amino acid	8.89E-06	43
Duodenum	Alfalfa hay	Negative	differentiation of muscle cells	8.90E-06	128
Duodenum	Alfalfa	Negative	homing of leukocytes	8.90E-06	168

	hay				
Duodenum	Alfalfa hay	Negative	growth of skin	8.91E-06	101
Duodenum	Alfalfa hay	Negative	quantity of subcutaneous fat	9.08E-06	34
Duodenum	Alfalfa hay	Negative	metabolism of DNA	9.19E-06	177
Duodenum	Alfalfa hay	Negative	abnormal morphology of blood vessel	9.35E-06	121
Duodenum	Alfalfa hay	Negative	development of sensory organ	9.43E-06	246
Duodenum	Alfalfa hay	Negative	breast cancer	9.76E-06	634
Duodenum	Alfalfa hay	Negative	quantity of macrophages	1.01E-05	94
Duodenum	Alfalfa hay	Negative	quantity of immunoglobulin	1.01E-05	134
Duodenum	Alfalfa hay	Negative	concentration of sterol	1.03E-05	138
Duodenum	Alfalfa hay	Negative	abnormal morphology of type II pneumocytes	1.03E-05	19
Duodenum	Alfalfa hay	Negative	familial Alzheimer's disease	1.03E-05	19
Duodenum	Alfalfa hay	Negative	length of cells	1.03E-05	51
Duodenum	Alfalfa hay	Negative	morphology of heart ventricle	1.05E-05	90
Duodenum	Alfalfa hay	Negative	exocytosis	1.06E-05	89
Duodenum	Alfalfa hay	Negative	growth of liver	1.06E-05	89
Duodenum	Alfalfa hay	Negative	cell viability of central nervous system cells	1.06E-05	56
Duodenum	Alfalfa hay	Negative	migration of connective tissue cells	1.10E-05	85
Duodenum	Alfalfa hay	Negative	outgrowth of neurites	1.10E-05	179
Duodenum	Alfalfa hay	Negative	differentiation of muscle cell lines	1.10E-05	77
Duodenum	Alfalfa hay	Negative	function of macrophages	1.10E-05	84
Duodenum	Alfalfa hay	Negative	contractility of heart	1.10E-05	83
Duodenum	Alfalfa	Negative	phosphorylation of L-tyrosine	1.10E-05	83

	hay				
Duodenum	Alfalfa hay	Negative	proliferation of endothelial cells	1.10E-05	140
Duodenum	Alfalfa hay	Negative	urination disorder	1.10E-05	131
Duodenum	Alfalfa hay	Negative	cellular infiltration	1.13E-05	193
Duodenum	Alfalfa hay	Negative	arrest in interphase	1.14E-05	184
Duodenum	Alfalfa hay	Negative	congenital heart disease	1.16E-05	97
Duodenum	Alfalfa hay	Negative	Breast Cancer and Tumors	1.19E-05	635
Duodenum	Alfalfa hay	Negative	cleavage of lipid	1.24E-05	93
Duodenum	Alfalfa hay	Negative	abnormal morphology of internal genitalia	1.25E-05	156
Duodenum	Alfalfa hay	Negative	advanced stage solid tumor	1.26E-05	225
Duodenum	Alfalfa hay	Negative	cell death of liver cells	1.27E-05	91
Duodenum	Alfalfa hay	Negative	invasion of tissue	1.27E-05	91
Duodenum	Alfalfa hay	Negative	apoptosis of neuroblastoma cell lines	1.29E-05	60
Duodenum	Alfalfa hay	Negative	proliferation of liver cells	1.32E-05	88
Duodenum	Alfalfa hay	Negative	metabolism of peptide	1.34E-05	87
Duodenum	Alfalfa hay	Negative	multiple congenital anomalies	1.36E-05	186
Duodenum	Alfalfa hay	Negative	morphology of cardiomyocytes	1.42E-05	47
Duodenum	Alfalfa hay	Negative	release of L-glutamic acid	1.45E-05	41
Duodenum	Alfalfa hay	Negative	cell death of T lymphocytes	1.45E-05	141
Duodenum	Alfalfa hay	Negative	development of genitourinary system	1.46E-05	377
Duodenum	Alfalfa hay	Negative	quantity of IL-6 in blood	1.47E-05	44
Duodenum	Alfalfa hay	Negative	leukopenia	1.49E-05	64
Duodenum	Alfalfa	Negative	morphology of mouth	1.53E-05	65

	hay				
Duodenum	Alfalfa hay	Negative	stage 3 cancer	1.53E-05	65
Duodenum	Alfalfa hay	Negative	quantity of myeloid cells	1.54E-05	158
Duodenum	Alfalfa hay	Negative	metastatic gastrointestinal tract cancer	1.55E-05	102
Duodenum	Alfalfa hay	Negative	abnormal morphology of genital organ	1.55E-05	180
Duodenum	Alfalfa hay	Negative	experimentally-induced diabetes	1.55E-05	48
Duodenum	Alfalfa hay	Negative	metabolism of nucleoside triphosphate	1.60E-05	67
Duodenum	Alfalfa hay	Negative	biosynthesis of nucleoside triphosphate	1.64E-05	53
Duodenum	Alfalfa hay	Negative	development of hematopoietic system	1.68E-05	113
Duodenum	Alfalfa hay	Negative	shape change of neurites	1.71E-05	128
Duodenum	Alfalfa hay	Negative	production of reactive oxygen species	1.73E-05	162
Duodenum	Rice straw	Positive	cancer	1.42E-34	2894
Duodenum	Rice straw	Positive	malignant solid tumor	6.33E-34	2861
Duodenum	Rice straw	Positive	organismal death	9.10E-29	788
Duodenum	Rice straw	Positive	morbidity or mortality	6.12E-28	793
Duodenum	Rice straw	Positive	proliferation of cells	2.85E-25	1115
Duodenum	Rice straw	Positive	tumorigenesis of tissue	7.77E-22	2413
Duodenum	Rice straw	Positive	epithelial cancer	5.10E-21	2353
Duodenum	Rice straw	Positive	morphology of cells	5.10E-21	631
Duodenum	Rice straw	Positive	neoplasia of epithelial tissue	2.10E-20	2369
Duodenum	Rice straw	Positive	apoptosis	1.36E-19	811
Duodenum	Rice straw	Positive	cell death	1.08E-18	987
Duodenum	Rice	Positive	necrosis	1.98E-18	786

	straw				
Duodenum	Rice straw	Positive	expression of RNA	5.24E-18	671
Duodenum	Rice straw	Positive	transcription	8.28E-18	629
Duodenum	Rice straw	Positive	abdominal neoplasm	9.84E-18	2336
Duodenum	Rice straw	Positive	cell movement	9.84E-18	664
Duodenum	Rice straw	Positive	differentiation of cells	9.26E-17	692
Duodenum	Rice straw	Positive	angiogenesis	9.26E-17	320
Duodenum	Rice straw	Positive	quantity of cells	1.50E-16	561
Duodenum	Rice straw	Positive	abdominal cancer	4.94E-16	2300
Duodenum	Rice straw	Positive	transcription of RNA	6.40E-16	582
Duodenum	Rice straw	Positive	transport of molecule	6.67E-16	515
Duodenum	Rice straw	Positive	organization of cytoplasm	1.88E-15	491
Duodenum	Rice straw	Positive	migration of cells	4.00E-15	590
Duodenum	Rice straw	Positive	abnormal morphology of cells	5.24E-15	415
Duodenum	Rice straw	Positive	Growth Failure	4.24E-14	218
Duodenum	Rice straw	Positive	cellular homeostasis	2.31E-13	478
Duodenum	Rice straw	Positive	organization of cytoskeleton	4.41E-13	442
Duodenum	Rice straw	Positive	size of body	1.02E-12	288
Duodenum	Rice straw	Positive	morphology of cardiovascular system	1.67E-12	219
Duodenum	Rice straw	Positive	quantity of leukocytes	2.63E-12	305
Duodenum	Rice straw	Positive	vasculogenesis	2.65E-12	253
Duodenum	Rice straw	Positive	quantity of lymphocytes	3.17E-12	244
Duodenum	Rice	Positive	quantity of mononuclear leukocytes	3.60E-12	252

	straw				
Duodenum	Rice straw	Positive	cell movement of tumor cell lines	5.58E-12	285
Duodenum	Rice straw	Positive	cell survival	7.29E-12	435
Duodenum	Rice straw	Positive	cell death of tumor cell lines	7.29E-12	471
Duodenum	Rice straw	Positive	ubiquitination	1.25E-11	129
Duodenum	Rice straw	Positive	cell viability	1.59E-11	408
Duodenum	Rice straw	Positive	ubiquitination of protein	1.65E-11	127
Duodenum	Rice straw	Positive	protein kinase cascade	1.65E-11	155
Duodenum	Rice straw	Positive	abnormal morphology of cardiovascular system	2.36E-11	199
Duodenum	Rice straw	Positive	proliferation of connective tissue cells	3.41E-11	206
Duodenum	Rice straw	Positive	quantity of blood cells	3.63E-11	331
Duodenum	Rice straw	Positive	transcription of DNA	4.66E-11	469
Duodenum	Rice straw	Positive	apoptosis of tumor cell lines	4.75E-11	381
Duodenum	Rice straw	Positive	microtubule dynamics	5.82E-11	373
Duodenum	Rice straw	Positive	digestive organ tumor	5.82E-11	1992
Duodenum	Rice straw	Positive	digestive system cancer	9.28E-11	1973
Duodenum	Rice straw	Positive	growth of connective tissue	1.20E-10	219
Duodenum	Rice straw	Positive	Movement Disorders	2.12E-10	336
Duodenum	Rice straw	Positive	invasion of cells	2.12E-10	283
Duodenum	Rice straw	Positive	transactivation	2.95E-10	198
Duodenum	Rice straw	Positive	cell death of connective tissue cells	6.48E-10	204
Duodenum	Rice straw	Positive	growth of organism	7.63E-10	271
Duodenum	Rice	Positive	morphology of body cavity	8.59E-10	370

	straw				
Duodenum	Rice straw	Positive	cell transformation	1.03E-09	161
Duodenum	Rice straw	Positive	differentiation of connective tissue	1.03E-09	240
Duodenum	Rice straw	Positive	quantity of T lymphocytes	1.15E-09	183
Duodenum	Rice straw	Positive	migration of tumor cell lines	1.21E-09	233
Duodenum	Rice straw	Positive	abnormal morphology of thoracic cavity	1.25E-09	198
Duodenum	Rice straw	Positive	cell death of epithelial cells	1.68E-09	171
Duodenum	Rice straw	Positive	development of connective tissue	1.76E-09	133
Duodenum	Rice straw	Positive	necrosis of epithelial tissue	1.76E-09	198
Duodenum	Rice straw	Positive	binding of DNA	1.78E-09	178
Duodenum	Rice straw	Positive	Bleeding	1.86E-09	149
Duodenum	Rice straw	Positive	development of vasculature	2.24E-09	147
Duodenum	Rice straw	Positive	morphology of heart	2.38E-09	143
Duodenum	Rice straw	Positive	perinatal death	2.43E-09	199
Duodenum	Rice straw	Positive	differentiation of connective tissue cells	2.56E-09	213
Duodenum	Rice straw	Positive	abnormal morphology of body cavity	3.63E-09	352
Duodenum	Rice straw	Positive	degeneration of nervous system	4.08E-09	106
Duodenum	Rice straw	Positive	transactivation of RNA	4.59E-09	183
Duodenum	Rice straw	Positive	formation of cells	4.59E-09	308
Duodenum	Rice straw	Positive	Organ Degeneration	5.09E-09	168
Duodenum	Rice straw	Positive	invasion of tumor cell lines	5.60E-09	218
Duodenum	Rice straw	Positive	Neurodegeneration	7.50E-09	112
Duodenum	Rice	Positive	morphology of bone	1.04E-08	154

	straw				
Duodenum	Rice straw	Positive	colony formation of cells	1.36E-08	166
Duodenum	Rice straw	Positive	apoptosis of fibroblast cell lines	1.38E-08	117
Duodenum	Rice straw	Positive	development of body trunk	2.02E-08	341
Duodenum	Rice straw	Positive	colony formation	2.55E-08	178
Duodenum	Rice straw	Positive	abnormal morphology of nervous system	2.70E-08	232
Duodenum	Rice straw	Positive	apoptosis of epithelial cells	2.99E-08	103
Duodenum	Rice straw	Positive	metabolism of DNA	3.01E-08	137
Duodenum	Rice straw	Positive	development of blood cells	3.31E-08	211
Duodenum	Rice straw	Positive	quantity of B lymphocytes	3.41E-08	121
Duodenum	Rice straw	Positive	growth of embryo	3.42E-08	160
Duodenum	Rice straw	Positive	morphology of nervous system	3.42E-08	248
Duodenum	Rice straw	Positive	abnormal morphology of bone	3.44E-08	148
Duodenum	Rice straw	Positive	neuronal cell death	3.50E-08	219
Duodenum	Rice straw	Positive	formation of cytoskeleton	3.53E-08	130
Duodenum	Rice straw	Positive	function of cardiovascular system	3.63E-08	128
Duodenum	Rice straw	Positive	organization of organelle	3.67E-08	178
Duodenum	Rice straw	Positive	proliferation of tumor cell lines	3.76E-08	454
Duodenum	Rice straw	Positive	proliferation of blood cells	3.92E-08	264
Duodenum	Rice straw	Positive	S phase	3.92E-08	95
Duodenum	Rice straw	Positive	synthesis of lipid	3.92E-08	228
Duodenum	Rice straw	Positive	adenocarcinoma	4.44E-08	1718
Duodenum	Rice	Positive	development of epithelial tissue	4.45E-08	171

	straw				
Duodenum	Rice straw	Positive	proliferation of neuronal cells	4.94E-08	188
Duodenum	Rice straw	Positive	apoptosis of leukocytes	6.23E-08	153
Duodenum	Rice straw	Positive	proliferation of fibroblast cell lines	6.69E-08	143
Duodenum	Rice straw	Positive	development of leukocytes	6.97E-08	191
Duodenum	Rice straw	Positive	apoptosis of blood cells	6.97E-08	164
Duodenum	Rice straw	Positive	development of cytoplasm	8.05E-08	154
Duodenum	Rice straw	Positive	congenital anomaly of musculoskeletal system	1.08E-07	232
Duodenum	Rice straw	Positive	activation of cells	1.09E-07	299
Duodenum	Rice straw	Positive	abdominal adenocarcinoma	1.13E-07	1566
Duodenum	Rice straw	Positive	quantity of connective tissue	1.18E-07	194
Duodenum	Rice straw	Positive	generation of blood cells	1.31E-07	64
Duodenum	Rice straw	Positive	generation of leukocytes	1.31E-07	63
Duodenum	Rice straw	Positive	morphology of cardiovascular tissue	1.31E-07	39
Duodenum	Rice straw	Positive	development of lymphatic system	1.35E-07	122
Duodenum	Rice straw	Positive	cell cycle progression	1.37E-07	317
Duodenum	Rice straw	Positive	function of blood cells	1.37E-07	189
Duodenum	Rice straw	Positive	proliferation of lymphatic system cells	1.40E-07	75
Duodenum	Rice straw	Positive	quantity of lymphatic system component	1.53E-07	128
Duodenum	Rice straw	Positive	activation of DNA endogenous promoter	1.89E-07	360
Duodenum	Rice straw	Positive	morphology of respiratory system	1.89E-07	108
Duodenum	Rice straw	Positive	morphology of connective tissue	1.92E-07	152
Duodenum	Rice	Positive	apoptosis of muscle	2.03E-07	93

	straw				
Duodenum	Rice straw	Positive	generation of lymphocytes	2.03E-07	56
Duodenum	Rice straw	Positive	function of leukocytes	2.06E-07	174
Duodenum	Rice straw	Positive	demyelination of central nervous system	2.14E-07	24
Duodenum	Rice straw	Positive	morphology of skin	2.21E-07	96
Duodenum	Rice straw	Positive	mitosis	2.21E-07	162
Duodenum	Rice straw	Positive	differentiation of epithelial tissue	2.22E-07	122
Duodenum	Rice straw	Positive	abnormal morphology of heart	2.26E-07	129
Duodenum	Rice straw	Positive	Viral Infection	2.32E-07	465
Duodenum	Rice straw	Positive	female genital neoplasm	2.38E-07	970
Duodenum	Rice straw	Positive	quantity of thymus gland	2.54E-07	84
Duodenum	Rice straw	Positive	synthesis of nitric oxide	2.73E-07	97
Duodenum	Rice straw	Positive	apoptosis of neurons	2.73E-07	144
Duodenum	Rice straw	Positive	apoptosis of muscle cells	2.87E-07	92
Duodenum	Rice straw	Positive	degeneration of cells	2.95E-07	117
Duodenum	Rice straw	Positive	interphase	3.03E-07	207
Duodenum	Rice straw	Positive	hepatocellular carcinoma	3.03E-07	1129
Duodenum	Rice straw	Positive	proliferation of immune cells	3.05E-07	245
Duodenum	Rice straw	Positive	neuromuscular disease	3.17E-07	270
Duodenum	Rice straw	Positive	quantity of lymphoid organ	3.18E-07	106
Duodenum	Rice straw	Positive	abnormal morphology of abdomen	3.53E-07	268
Duodenum	Rice straw	Positive	sprouting	3.60E-07	129
Duodenum	Rice	Positive	morphology of reproductive system	3.95E-07	172

	straw				
Duodenum	Rice straw	Positive	growth of plasma membrane projections	4.42E-07	152
Duodenum	Rice straw	Positive	disorder of basal ganglia	4.68E-07	237
Duodenum	Rice straw	Positive	behavior	4.68E-07	298
Duodenum	Rice straw	Positive	cell movement of fibroblasts	4.75E-07	68
Duodenum	Rice straw	Positive	morphology of head	4.85E-07	267
Duodenum	Rice straw	Positive	cell death of hepatocytes	4.88E-07	59
Duodenum	Rice straw	Positive	abnormal morphology of head	4.96E-07	255
Duodenum	Rice straw	Positive	abnormal morphology of reproductive system	4.96E-07	164
Duodenum	Rice straw	Positive	activation of enzyme	5.12E-07	129
Duodenum	Rice straw	Positive	abnormal morphology of respiratory system	5.21E-07	105
Duodenum	Rice straw	Positive	cell death of blood cells	5.63E-07	216
Duodenum	Rice straw	Positive	migration of connective tissue cells	5.92E-07	67
Duodenum	Rice straw	Positive	cell death of fibroblast cell lines	6.02E-07	140
Duodenum	Rice straw	Positive	quantity of thymocytes	6.51E-07	82
Duodenum	Rice straw	Positive	differentiation of bone cells	6.61E-07	134
Duodenum	Rice straw	Positive	cell death of T lymphocytes	6.66E-07	107
Duodenum	Rice straw	Positive	differentiation of bone	6.72E-07	135
Duodenum	Rice straw	Positive	branching of cells	6.80E-07	123
Duodenum	Rice straw	Positive	skin abnormality	6.80E-07	81
Duodenum	Rice straw	Positive	cell death of fibroblasts	6.93E-07	92
Duodenum	Rice straw	Positive	genital tumor	7.20E-07	1088
Duodenum	Rice	Positive	binding of protein binding site	7.20E-07	99

	straw				
Duodenum	Rice straw	Positive	size of animal	7.27E-07	66
Duodenum	Rice straw	Positive	formation of skin	7.27E-07	122
Duodenum	Rice straw	Positive	phosphorylation of protein	7.36E-07	227
Duodenum	Rice straw	Positive	development of lymphatic system component	7.61E-07	105
Duodenum	Rice straw	Positive	neonatal death	7.63E-07	143
Duodenum	Rice straw	Positive	morphology of endothelial tissue	7.99E-07	36
Duodenum	Rice straw	Positive	cell death of liver	8.96E-07	84
Duodenum	Rice straw	Positive	cell movement of connective tissue cells	8.96E-07	79
Duodenum	Rice straw	Positive	cognition	9.08E-07	147
Duodenum	Rice straw	Positive	Edema	9.18E-07	115
Duodenum	Rice straw	Positive	apoptosis of heart cells	9.18E-07	69
Duodenum	Rice straw	Positive	growth of epithelial tissue	9.22E-07	211
Duodenum	Rice straw	Positive	cell death of lymphocytes	9.35E-07	127
Duodenum	Rice straw	Positive	growth of neurites	1.03E-06	149
Duodenum	Rice straw	Positive	apoptosis of T lymphocytes	1.03E-06	96
Duodenum	Rice straw	Positive	synthesis of DNA	1.03E-06	136
Duodenum	Rice straw	Positive	cell death of immune cells	1.05E-06	205
Duodenum	Rice straw	Positive	quantity of phagocytes	1.06E-06	138
Duodenum	Rice straw	Positive	development of lymphocytes	1.07E-06	175
Duodenum	Rice straw	Positive	development of cardiovascular tissue	1.15E-06	122
Duodenum	Rice straw	Positive	seizure disorder	1.15E-06	151
Duodenum	Rice	Positive	development of mononuclear leukocytes	1.17E-06	176

	straw				
Duodenum	Rice straw	Positive	cellular degradation	1.21E-06	88
Duodenum	Rice straw	Positive	proliferation of fibroblasts	1.24E-06	117
Duodenum	Rice straw	Positive	endometrium tumor	1.26E-06	791
Duodenum	Rice straw	Positive	necrosis of liver	1.27E-06	83
Duodenum	Rice straw	Positive	apoptosis of central nervous system cells	1.27E-06	60
Duodenum	Rice straw	Positive	apoptosis of lymphocytes	1.29E-06	115
Duodenum	Rice straw	Positive	T cell development	1.34E-06	160
Duodenum	Rice straw	Positive	proliferation of hematopoietic cells	1.39E-06	88
Duodenum	Rice straw	Positive	apoptosis of cardiomyocytes	1.42E-06	67
Duodenum	Rice straw	Positive	apoptosis of hepatocytes	1.45E-06	51
Duodenum	Rice straw	Positive	biosynthesis of nucleoside triphosphate	1.53E-06	43
Duodenum	Rice straw	Positive	proliferation of embryonic cells	1.62E-06	86
Duodenum	Rice straw	Positive	differentiation of epithelial cells	1.63E-06	105
Duodenum	Rice straw	Positive	cell death of dermal cells	1.66E-06	40
Duodenum	Rice straw	Positive	morphology of vessel	1.68E-06	103
Duodenum	Rice straw	Positive	outgrowth of plasma membrane projections	1.81E-06	132
Duodenum	Rice straw	Positive	cell viability of connective tissue cells	1.84E-06	51
Duodenum	Rice straw	Positive	growth of lymphatic system component	1.84E-06	69
Duodenum	Rice straw	Positive	proliferation of hematopoietic progenitor cells	1.86E-06	84
Duodenum	Rice straw	Positive	cell death of mononuclear leukocytes	1.88E-06	129
Duodenum	Rice straw	Positive	learning	1.88E-06	135
Duodenum	Rice	Positive	liver cancer	1.96E-06	1154

	straw				
Duodenum	Rice straw	Positive	growth of muscle tissue	2.01E-06	122
Duodenum	Rice straw	Positive	morphology of muscle	2.04E-06	111
Duodenum	Rice straw	Positive	abdominal carcinoma	2.04E-06	1572
Duodenum	Rice straw	Positive	abnormal morphology of epithelial tissue	2.09E-06	135
Duodenum	Rice straw	Positive	endometrial cancer	2.11E-06	787
Duodenum	Rice straw	Positive	tumorigenesis of reproductive tract	2.11E-06	924
Duodenum	Rice straw	Positive	autosomal recessive disease	2.22E-06	275
Duodenum	Rice straw	Positive	cell viability of tumor cell lines	2.30E-06	236
Duodenum	Rice straw	Positive	hepatobiliary system cancer	2.31E-06	1158
Duodenum	Rice straw	Positive	proliferation of muscle cells	2.44E-06	121
Duodenum	Rice straw	Positive	apoptosis of mononuclear leukocytes	2.46E-06	117
Duodenum	Rice straw	Positive	growth of embryonic tissue	2.52E-06	93
Duodenum	Rice straw	Positive	inflammation of central nervous system	2.53E-06	101
Duodenum	Rice straw	Positive	dysgenesis	2.58E-06	169
Duodenum	Rice straw	Positive	cell death of cervical cancer cell lines	2.59E-06	115
Duodenum	Rice straw	Positive	generation of T lymphocytes	2.70E-06	45
Duodenum	Rice straw	Positive	female genital tract adenocarcinoma	2.72E-06	786
Duodenum	Rice straw	Positive	morphology of endothelial cells	2.72E-06	28
Duodenum	Rice straw	Positive	differentiation of tumor cell lines	2.83E-06	123
Duodenum	Rice straw	Positive	migration of blood cells	3.08E-06	261
Duodenum	Rice straw	Positive	T cell homeostasis	3.09E-06	162
Duodenum	Rice	Positive	metabolism of protein	3.09E-06	288

	straw				
Duodenum	Rice straw	Positive	proliferation of mononuclear leukocytes	3.13E-06	224
Duodenum	Rice straw	Positive	aggregation of blood platelets	3.20E-06	68
Duodenum	Rice straw	Positive	development of endothelial tissue	3.22E-06	119
Duodenum	Rice straw	Positive	outgrowth of neurites	3.22E-06	130
Duodenum	Rice straw	Positive	degeneration of neurons	3.25E-06	82
Duodenum	Rice straw	Positive	outgrowth of neurons	3.25E-06	131
Duodenum	Rice straw	Positive	Lymphocyte homeostasis	3.31E-06	170
Duodenum	Rice straw	Positive	proliferation of myeloid cells	3.46E-06	46
Duodenum	Rice straw	Positive	female genital tract cancer	3.48E-06	916
Duodenum	Rice straw	Positive	abnormal morphology of skull	3.70E-06	76
Duodenum	Rice straw	Positive	size of embryo	3.73E-06	116
Duodenum	Rice straw	Positive	leukocyte migration	3.76E-06	260
Duodenum	Rice straw	Positive	formation of actin stress fibers	3.76E-06	82
Duodenum	Rice straw	Positive	proliferation of epidermal cells	3.76E-06	61
Duodenum	Rice straw	Positive	apoptosis of cerebral cortex cells	4.03E-06	42
Duodenum	Rice straw	Positive	abnormal morphology of skin	4.04E-06	83
Duodenum	Rice straw	Positive	endothelial cell development	4.04E-06	115
Duodenum	Rice straw	Positive	migration of fibroblasts	4.08E-06	53
Duodenum	Rice straw	Positive	autophagy	4.15E-06	121
Duodenum	Rice straw	Positive	metabolism of nucleoside triphosphate	4.15E-06	52
Duodenum	Rice straw	Positive	formation of cellular protrusions	4.18E-06	268
Duodenum	Rice	Positive	liver tumor	4.26E-06	1157

	straw				
Duodenum	Rice straw	Positive	cell movement of leukocytes	4.27E-06	231
Duodenum	Rice straw	Positive	cell death of pheochromocytoma cell lines	4.27E-06	46
Duodenum	Rice straw	Positive	Hypoplasia	4.27E-06	158
Duodenum	Rice straw	Positive	metabolism of nucleic acid component or derivative	4.27E-06	162
Duodenum	Rice straw	Positive	proliferation of lymphocytes	4.27E-06	220
Duodenum	Rice straw	Positive	formation of lymphoid organ	4.33E-06	89
Duodenum	Rice straw	Positive	formation of lymphatic system component	4.36E-06	97
Duodenum	Rice straw	Positive	ossification of bone	4.46E-06	56
Duodenum	Rice straw	Positive	proliferation of smooth muscle cells	4.51E-06	95
Duodenum	Rice straw	Positive	homeostasis of leukocytes	4.51E-06	172
Duodenum	Rice straw	Positive	abnormal morphology of vascular endothelial cells	4.54E-06	21
Duodenum	Rice straw	Positive	apoptosis of brain	4.57E-06	55
Duodenum	Rice straw	Positive	proliferation of dermal cells	4.75E-06	63
Duodenum	Rice straw	Positive	cell death of skin	4.82E-06	41
Duodenum	Rice straw	Positive	fibrogenesis	5.33E-06	131
Duodenum	Rice straw	Positive	generation of cells	5.38E-06	88
Duodenum	Rice straw	Positive	hypersensitive reaction	5.64E-06	136
Duodenum	Rice straw	Positive	size of bone	5.85E-06	92
Duodenum	Rice straw	Positive	demyelination of spinal cord	6.10E-06	17
Duodenum	Rice straw	Positive	uptake of carbohydrate	6.19E-06	96
Duodenum	Rice straw	Positive	concentration of lipid	6.22E-06	242
Duodenum	Rice	Positive	dysmyelination	6.52E-06	50

	straw				
Duodenum	Rice straw	Positive	degeneration of central nervous system	6.63E-06	49
Duodenum	Rice straw	Positive	vascularization	6.64E-06	80
Duodenum	Rice straw	Positive	cell death of epidermal cells	6.82E-06	37
Duodenum	Rice straw	Positive	development of myeloid cells	6.82E-06	37
Duodenum	Rice straw	Positive	immune response of cells	6.82E-06	176
Duodenum	Rice straw	Positive	autosomal dominant disease	6.85E-06	194
Duodenum	Rice straw	Positive	seizures	6.87E-06	127
Duodenum	Rice straw	Positive	cell death of liver cells	7.10E-06	68
Duodenum	Rice straw	Positive	Encephalitis	7.26E-06	97
Duodenum	Rice straw	Positive	differentiation of muscle cell lines	7.32E-06	58
Duodenum	Rice straw	Positive	advanced malignant tumor	7.35E-06	251
Duodenum	Rice straw	Positive	apoptosis of liver	7.66E-06	57
Duodenum	Rice straw	Positive	uterine tumor	7.72E-06	860
Duodenum	Rice straw	Positive	maturation of cells	7.83E-06	143
Duodenum	Rice straw	Positive	cell death of central nervous system cells	7.83E-06	96
Duodenum	Rice straw	Positive	cell death of muscle	7.83E-06	111
Duodenum	Rice straw	Positive	attachment of cells	7.88E-06	50
Duodenum	Rice straw	Positive	autophagy of cells	7.97E-06	88
Duodenum	Rice straw	Positive	abnormal morphology of endothelial tissue	8.01E-06	28
Duodenum	Rice straw	Positive	morphogenesis of neurons	8.07E-06	134
Duodenum	Rice straw	Positive	apoptosis of connective tissue cells	8.07E-06	97
Duodenum	Rice	Positive	apoptosis of dermal cells	8.25E-06	36

	straw				
Duodenum	Rice straw	Positive	metabolism of carbohydrate	8.26E-06	189
Duodenum	Rice straw	Positive	proliferation of bone marrow cells	8.26E-06	45
Duodenum	Rice straw	Positive	differentiation of leukocytes	8.91E-06	203
Duodenum	Rice straw	Positive	apoptosis of brain cells	9.13E-06	52
Duodenum	Rice straw	Positive	congenital anomaly of cardiovascular system	9.44E-06	77
Duodenum	Rice straw	Positive	adhesion of immune cells	1.01E-05	112
Duodenum	Rice straw	Positive	outgrowth of cells	1.01E-05	136
Duodenum	Rice straw	Positive	cell death of muscle cells	1.02E-05	108
Duodenum	Rice straw	Positive	epilepsy	1.02E-05	100
Duodenum	Rice straw	Positive	cell death of keratinocytes	1.02E-05	35
Duodenum	Rice straw	Positive	cell death of smooth muscle cells	1.04E-05	43
Duodenum	Rice straw	Positive	demyelination	1.04E-05	45
Duodenum	Rice straw	Positive	necrosis of muscle	1.06E-05	110
Duodenum	Rice straw	Positive	survival of organism	1.07E-05	211
Duodenum	Rice straw	Positive	production of protein	1.08E-05	106
Duodenum	Rice straw	Positive	binding of T lymphocytes	1.12E-05	30
Duodenum	Rice straw	Positive	cytopenia	1.17E-05	97
Duodenum	Rice straw	Positive	tumorigenesis of genital organ	1.18E-05	1041
Duodenum	Rice straw	Positive	metabolism of nucleotide	1.22E-05	137
Duodenum	Rice straw	Positive	cell death of gonadal cell lines	1.29E-05	31
Duodenum	Rice straw	Positive	quantity of hematopoietic cells	1.37E-05	138
Duodenum	Rice	Positive	hypoplasia of organ	1.37E-05	139

	straw				
Duodenum	Rice straw	Positive	formation of muscle	1.37E-05	125
Duodenum	Rice straw	Positive	morphogenesis of neurites	1.45E-05	131
Duodenum	Rice straw	Positive	homing of cells	1.50E-05	170
Duodenum	Rice straw	Positive	morphology of mouth	1.52E-05	49
Duodenum	Rice straw	Positive	formation of filaments	1.53E-05	125
Duodenum	Rice straw	Positive	morphology of heart ventricle	1.54E-05	66
Duodenum	Rice straw	Positive	quantity of glycosphingolipid	1.58E-05	38
Duodenum	Rice straw	Positive	morphology of blood vessel	1.62E-05	92
Duodenum	Rice straw	Positive	activation of blood cells	1.62E-05	222
Duodenum	Rice straw	Positive	development of head	1.68E-05	285
Duodenum	Rice straw	Positive	morphology of skeleton	1.77E-05	91
Duodenum	Rice straw	Positive	dyskinesia	1.77E-05	186
Duodenum	Rice straw	Positive	uptake of monosaccharide	1.77E-05	88
Duodenum	Rice straw	Positive	aggregation of blood cells	1.80E-05	78
Duodenum	Rice straw	Positive	quantity of hematopoietic progenitor cells	1.80E-05	137
Duodenum	Rice straw	Positive	polyarticular juvenile rheumatoid arthritis	1.80E-05	35
Duodenum	Rice straw	Positive	growth of tumor	1.80E-05	235
Duodenum	Rice straw	Positive	coagulation	1.80E-05	66
Duodenum	Rice straw	Positive	aggregation of cells	1.83E-05	104
Duodenum	Rice straw	Positive	arrest in proliferation of cells	1.85E-05	72
Duodenum	Rice straw	Positive	metastasis	1.87E-05	225
Duodenum	Rice	Positive	function of myeloid cells	1.89E-05	79

	straw				
Duodenum	Rice straw	Positive	cell movement of endothelial cells	1.93E-05	112
Duodenum	Rice straw	Positive	cell death of leukemia cell lines	1.95E-05	102
Duodenum	Rice straw	Positive	formation of actin filaments	1.95E-05	98
Duodenum	Rice straw	Positive	growth of skin	1.97E-05	73
Duodenum	Rice straw	Positive	morphology of digestive system	1.99E-05	173
Duodenum	Rice straw	Positive	apoptosis of neuroblastoma cell lines	2.02E-05	45
Duodenum	Rice straw	Positive	engulfment of cells	2.06E-05	127
Duodenum	Rice straw	Positive	apoptosis of liver cells	2.08E-05	55
Duodenum	Rice straw	Positive	endometrial carcinoma	2.09E-05	727
Duodenum	Rice straw	Positive	migration of vascular endothelial cells	2.11E-05	58
Duodenum	Rice straw	Positive	cell death of brain	2.18E-05	94
Duodenum	Rice straw	Positive	apoptosis of epidermal cells	2.21E-05	34
Duodenum	Rice straw	Positive	arthritis	2.33E-05	272
Duodenum	Rice straw	Positive	interphase of connective tissue cells	2.36E-05	31
Duodenum	Rice straw	Positive	quantity of granulocytes	2.38E-05	101
Duodenum	Rice straw	Positive	differentiation of blood cells	2.38E-05	247
Duodenum	Rice straw	Positive	abnormal morphology of digestive system	2.52E-05	165
Duodenum	Rice straw	Positive	epileptic seizure	2.53E-05	58
Duodenum	Rice straw	Positive	abnormal morphology of skeleton	2.54E-05	88
Duodenum	Rice straw	Positive	endometrioid carcinoma	2.54E-05	720
Duodenum	Rice straw	Positive	development of phagocytes	2.55E-05	32
Duodenum	Rice	Positive	adhesion of blood cells	2.55E-05	118

	straw				
Duodenum	Rice straw	Positive	interphase of fibroblasts	2.56E-05	29
Duodenum	Rice straw	Positive	homing	2.73E-05	173
Duodenum	Rice straw	Positive	movement of vascular endothelial cells	2.73E-05	62
Duodenum	Rice straw	Positive	morphology of lymphatic system component	2.77E-05	129
Duodenum	Rice straw	Positive	proliferation of hematopoietic cell lines	2.80E-05	74
Duodenum	Rice straw	Positive	development of body axis	2.82E-05	302
Duodenum	Rice straw	Positive	migration of endothelial cells	2.83E-05	104
Duodenum	Rice straw	Positive	quantity of immunoglobulin	2.84E-05	95
Duodenum	Rice straw	Positive	congenital heart disease	2.84E-05	70
Duodenum	Rice straw	Positive	Huntington's Disease	2.94E-05	173
Duodenum	Rice straw	Positive	abnormal morphology of blood vessel	3.00E-05	86
Duodenum	Rice straw	Positive	morphology of genital organ	3.06E-05	131
Duodenum	Rice straw	Positive	proliferation of T lymphocytes	3.14E-05	181
Duodenum	Rice straw	Positive	genital tract cancer	3.25E-05	1027
Duodenum	Rice straw	Positive	proliferation of epithelial cells	3.27E-05	150
Duodenum	Rice straw	Positive	metabolism of sphingolipid	3.29E-05	49
Duodenum	Rice straw	Positive	quantity of antigen presenting cells	3.37E-05	89
Duodenum	Rice straw	Positive	apoptosis of keratinocytes	3.39E-05	32
Duodenum	Rice straw	Positive	quantity of carbohydrate	3.39E-05	157
Duodenum	Rice straw	Positive	uptake of D-glucose	3.48E-05	73
Duodenum	Rice straw	Positive	arthropathy	3.48E-05	275
Duodenum	Rice	Positive	synthesis of ceramide	3.50E-05	29

	straw				
Duodenum	Rice straw	Positive	cell death of neuroblastoma cell lines	3.53E-05	65
Duodenum	Rice straw	Positive	development of hematopoietic system	3.53E-05	81
Duodenum	Rice straw	Positive	multiple congenital anomalies	3.67E-05	130
Duodenum	Rice straw	Positive	apoptosis of fibroblasts	3.76E-05	72
Duodenum	Rice straw	Positive	abnormal morphology of snout	3.76E-05	27
Duodenum	Rice straw	Positive	growth of liver	3.80E-05	64
Duodenum	Rice straw	Positive	development of neurons	3.81E-05	229
Duodenum	Rice straw	Positive	apoptosis of pheochromocytoma cell lines	3.88E-05	35
Duodenum	Rice straw	Positive	recruitment of myeloid cells	3.88E-05	75
Duodenum	Rice straw	Positive	quantity of myeloid cells	4.39E-05	111
Duodenum	Rice straw	Positive	recruitment of phagocytes	4.43E-05	80
Duodenum	Rice straw	Positive	apoptosis of cervical cancer cell lines	4.44E-05	90
Duodenum	Rice straw	Positive	neurological signs	4.46E-05	193
Duodenum	Rice straw	Positive	activation of leukocytes	4.46E-05	206
Duodenum	Rice straw	Positive	cell death of cerebral cortex cells	4.48E-05	75
Duodenum	Rice straw	Positive	I-kappaB kinase/NF-kappaB cascade	4.60E-05	59
Duodenum	Rice straw	Positive	transformation of fibroblast cell lines	5.06E-05	83
Duodenum	Rice straw	Positive	branching of neurons	5.20E-05	91
Duodenum	Rice straw	Positive	transmigration of cells	5.41E-05	54
Duodenum	Rice straw	Positive	infection by Retroviridae	5.46E-05	211
Duodenum	Rice straw	Positive	paraproteinemia	5.61E-05	105
Duodenum	Rice	Positive	proliferation of liver cells	5.68E-05	63

	straw				
Duodenum	Rice straw	Positive	cell death of hematopoietic cells	5.78E-05	77
Duodenum	Rice straw	Positive	chronic inflammatory disorder	5.79E-05	279
Duodenum	Rice straw	Positive	cell death of heart cells	5.82E-05	72
Duodenum	Rice straw	Positive	uterine cancer	5.83E-05	810
Duodenum	Rice straw	Positive	cell death of brain cells	5.83E-05	87
Duodenum	Rice straw	Positive	cell spreading	6.03E-05	88
Duodenum	Rice straw	Positive	development of genitourinary system	6.05E-05	256
Duodenum	Rice straw	Positive	recruitment of neutrophils	6.07E-05	62
Duodenum	Rice straw	Positive	apoptosis of epithelial cell lines	6.11E-05	73
Duodenum	Rice straw	Positive	production of antibody	6.11E-05	98
Duodenum	Rice straw	Positive	function of lymphocytes	6.26E-05	106
Duodenum	Rice straw	Positive	adenocarcinoma in endometrium	6.26E-05	713
Duodenum	Rice straw	Positive	adjuvant arthritis	6.39E-05	17
Duodenum	Rice straw	Positive	cell death of heart	6.39E-05	74
Duodenum	Rice straw	Positive	cell death of hematopoietic progenitor cells	6.39E-05	74
Duodenum	Rice straw	Positive	abnormal morphology of genital organ	6.39E-05	125
Duodenum	Rice straw	Positive	quantity of metal	6.39E-05	150
Duodenum	Rice straw	Positive	congenital malformation of skeleton	6.39E-05	129
Duodenum	Rice straw	Positive	adhesion of tumor cell lines	6.45E-05	91
Duodenum	Rice straw	Positive	abnormal morphology of body wall	6.56E-05	38
Duodenum	Rice straw	Positive	branching of neurites	6.68E-05	88
Duodenum	Rice	Positive	consumption of oxygen	6.68E-05	53

	straw				
Duodenum	Rice straw	Positive	growth of lymphoid organ	6.76E-05	58
Duodenum	Rice straw	Positive	export of molecule	6.76E-05	93
Duodenum	Rice straw	Positive	cellular infiltration by leukocytes	6.86E-05	119
Duodenum	Rice straw	Positive	fatty acid metabolism	6.86E-05	178
Duodenum	Rice straw	Positive	cellular infiltration	6.91E-05	133
Duodenum	Rice straw	Positive	function of phagocytes	7.22E-05	96
Duodenum	Rice straw	Positive	fragmentation of DNA	7.22E-05	57
Duodenum	Rice straw	Positive	synthesis of nucleotide	7.24E-05	112
Duodenum	Rice straw	Positive	quantity of IgM	7.27E-05	48
Duodenum	Rice straw	Positive	recruitment of antigen presenting cells	7.33E-05	43
Duodenum	Rice straw	Positive	development of abdomen	7.42E-05	172
Duodenum	Rice straw	Positive	cell death of stem cells	7.59E-05	32
Duodenum	Rice straw	Positive	migration of myeloid cells	7.70E-05	56
Duodenum	Rice straw	Positive	encephalomyelitis	7.85E-05	82
Duodenum	Rice straw	Positive	gonadal tumor	7.86E-05	237
Duodenum	Rice straw	Positive	activation of lymphocytes	8.05E-05	139
Duodenum	Rice straw	Positive	binding of lymphocytes	8.17E-05	35
Duodenum	Rice straw	Positive	plasma cell dyscrasia	8.17E-05	104
Duodenum	Rice straw	Positive	secretion of molecule	8.18E-05	164
Duodenum	Rice straw	Positive	length of cells	8.18E-05	37
Duodenum	Rice straw	Positive	DNA replication	8.19E-05	80
Duodenum	Rice	Positive	cell death of hepatoma cell lines	8.42E-05	57

	straw				
Duodenum	Rice straw	Positive	cell death of epithelial cell lines	8.57E-05	89
Duodenum	Rice straw	Positive	long-term potentiation	8.60E-05	78
Duodenum	Rice straw	Positive	Inflammatory Bowel Disease	8.83E-05	82
Duodenum	Rice straw	Positive	blood protein disorder	8.93E-05	115
Duodenum	Rice straw	Positive	differentiation of T lymphocytes	8.96E-05	116
Duodenum	Rice straw	Positive	proliferation of cervical cancer cell lines	9.16E-05	66
Duodenum	Rice straw	Positive	binding of leukocytes	9.32E-05	60
Duodenum	Rice straw	Positive	adhesion of mononuclear leukocytes	9.36E-05	50
Duodenum	Rice straw	Positive	death of perinatal stage organism	9.45E-05	31
Duodenum	Rice straw	Positive	infection by lentivirus	9.52E-05	208
Duodenum	Rice straw	Positive	chemotaxis of cells	9.55E-05	157
Duodenum	Rice straw	Positive	interphase of tumor cell lines	9.69E-05	113
Duodenum	Rice straw	Positive	cell movement of carcinoma cell lines	9.69E-05	67
Duodenum	Rice straw	Positive	quantity of epithelial tissue	9.98E-05	59
Duodenum	Rice straw	Positive	apoptosis of hematopoietic cells	9.98E-05	70
Duodenum	Rice straw	Positive	release of metal	9.98E-05	70
Duodenum	Rice straw	Positive	replication of RNA virus	1.01E-04	151
Duodenum	Rice straw	Positive	biosynthesis of purine ribonucleotide	1.02E-04	37
Duodenum	Rice straw	Positive	cell death of myeloma cell lines	1.02E-04	35
Duodenum	Rice straw	Positive	metabolism of glycosphingolipid	1.04E-04	44
Duodenum	Rice straw	Positive	apoptosis of neutrophils	1.04E-04	25
Duodenum	Rice	Positive	hemorrhagic disease	1.04E-04	80

	straw				
Duodenum	Rice straw	Positive	proliferation of leukocyte cell lines	1.04E-04	66
Duodenum	Rice straw	Positive	neuritogenesis	1.06E-04	172
Duodenum	Rice straw	Positive	apoptosis of myeloid cells	1.07E-04	62
Duodenum	Rice straw	Positive	peripheral vascular disease	1.07E-04	116
Duodenum	Rice straw	Positive	cell movement of lymphocytes	1.08E-04	120
Duodenum	Rice straw	Positive	HIV infection	1.09E-04	207
Duodenum	Rice straw	Positive	apoptosis of hematopoietic progenitor cells	1.10E-04	67
Duodenum	Rice straw	Positive	cell death of embryonic cells	1.16E-04	41
Duodenum	Rice straw	Positive	accumulation of lipid	1.16E-04	93
Duodenum	Rice straw	Positive	migration of granulocytes	1.17E-04	49
Duodenum	Rice straw	Positive	urogenital cancer	1.18E-04	1164
Duodenum	Rice straw	Positive	morphology of lymphoid organ	1.18E-04	119
Duodenum	Rice straw	Positive	quantity of double-positive thymocyte	1.19E-04	40
Duodenum	Rice straw	Positive	function of heart	1.19E-04	74
Duodenum	Rice straw	Positive	targeting of protein	1.19E-04	45
Duodenum	Rice straw	Positive	experimental autoimmune encephalomyelitis	1.20E-04	81
Duodenum	Rice straw	Positive	cell death of cardiomyocytes	1.23E-04	69
Duodenum	Rice straw	Positive	recruitment of macrophages	1.24E-04	38
Duodenum	Rice straw	Positive	damage of nervous system	1.24E-04	72
Duodenum	Rice straw	Positive	morphology of vessel component	1.25E-04	24
Duodenum	Rice straw	Positive	bleeding of brain	1.27E-04	27
Duodenum	Rice	Positive	condensation of chromatin	1.27E-04	27

	straw				
Duodenum	Rice straw	Positive	morphology of vascular endothelial cells	1.27E-04	22
Duodenum	Rice straw	Positive	cell death of granulocytes	1.27E-04	34
Duodenum	Rice straw	Positive	synthesis of ATP	1.27E-04	34
Duodenum	Rice straw	Positive	female genital tract serous cancer	1.27E-04	117
Duodenum	Rice straw	Negative	malignant solid tumor	4.51E-16	1605
Duodenum	Rice straw	Negative	cell movement	1.83E-15	409
Duodenum	Rice straw	Negative	cancer	2.00E-15	1617
Duodenum	Rice straw	Negative	proliferation of cells	2.00E-15	643
Duodenum	Rice straw	Negative	morphology of cells	2.71E-15	378
Duodenum	Rice straw	Negative	cell death	7.97E-15	587
Duodenum	Rice straw	Negative	organization of cytoskeleton	1.62E-14	286
Duodenum	Rice straw	Negative	epithelial cancer	5.77E-14	1342
Duodenum	Rice straw	Negative	migration of cells	8.80E-14	364
Duodenum	Rice straw	Negative	organization of cytoplasm	8.80E-14	305
Duodenum	Rice straw	Negative	abdominal neoplasm	1.12E-13	1342
Duodenum	Rice straw	Negative	tumorigenesis of tissue	1.25E-13	1370
Duodenum	Rice straw	Negative	neoplasia of epithelial tissue	1.41E-13	1350
Duodenum	Rice straw	Negative	apoptosis	2.28E-12	470
Duodenum	Rice straw	Negative	microtubule dynamics	2.59E-12	242
Duodenum	Rice straw	Negative	organismal death	4.30E-12	431
Duodenum	Rice straw	Negative	abdominal cancer	8.49E-12	1317
Duodenum	Rice	Negative	necrosis	8.51E-12	456

	straw				
Duodenum	Rice straw	Negative	morbidity or mortality	2.30E-11	432
Duodenum	Rice straw	Negative	development of neurons	1.23E-10	168
Duodenum	Rice straw	Negative	digestive organ tumor	1.72E-10	1159
Duodenum	Rice straw	Negative	formation of cellular protrusions	1.72E-10	188
Duodenum	Rice straw	Negative	digestive system cancer	1.85E-10	1149
Duodenum	Rice straw	Negative	perinatal death	3.96E-10	133
Duodenum	Rice straw	Negative	transcription of RNA	9.58E-10	337
Duodenum	Rice straw	Negative	transcription	9.58E-10	358
Duodenum	Rice straw	Negative	differentiation of cells	1.31E-09	396
Duodenum	Rice straw	Negative	Movement Disorders	1.61E-09	210
Duodenum	Rice straw	Negative	transcription of DNA	2.47E-09	285
Duodenum	Rice straw	Negative	abnormal morphology of cells	4.85E-09	241
Duodenum	Rice straw	Negative	expression of RNA	4.85E-09	377
Duodenum	Rice straw	Negative	size of body	6.57E-09	173
Duodenum	Rice straw	Negative	angiogenesis	7.92E-09	182
Duodenum	Rice straw	Negative	disorder of basal ganglia	1.04E-08	158
Duodenum	Rice straw	Negative	cell movement of tumor cell lines	1.21E-08	172
Duodenum	Rice straw	Negative	transport of molecule	1.30E-08	293
Duodenum	Rice straw	Negative	development of vasculature	1.56E-08	96
Duodenum	Rice straw	Negative	abnormal morphology of thoracic cavity	1.68E-08	126
Duodenum	Rice straw	Negative	dyskinesia	2.01E-08	131
Duodenum	Rice	Negative	morphology of nervous system	2.87E-08	159

	straw				
Duodenum	Rice straw	Negative	cell movement of fibroblasts	4.85E-08	50
Duodenum	Rice straw	Negative	development of epithelial tissue	5.19E-08	112
Duodenum	Rice straw	Negative	neuritogenesis	8.07E-08	123
Duodenum	Rice straw	Negative	formation of plasma membrane projections	9.87E-08	125
Duodenum	Rice straw	Negative	cell movement of connective tissue cells	9.93E-08	57
Duodenum	Rice straw	Negative	neurological signs	1.27E-07	134
Duodenum	Rice straw	Negative	Huntington's Disease	2.14E-07	120
Duodenum	Rice straw	Negative	cell death of tumor cell lines	2.26E-07	273
Duodenum	Rice straw	Negative	neonatal death	2.43E-07	96
Duodenum	Rice straw	Negative	cellular homeostasis	4.00E-07	271
Duodenum	Rice straw	Negative	neuronal cell death	4.48E-07	137
Duodenum	Rice straw	Negative	congenital anomaly of musculoskeletal system	4.88E-07	146
Duodenum	Rice straw	Negative	migration of tumor cell lines	5.01E-07	141
Duodenum	Rice straw	Negative	vasculogenesis	5.04E-07	146
Duodenum	Rice straw	Negative	abnormal morphology of nervous system	9.12E-07	143
Duodenum	Rice straw	Negative	morphology of respiratory system	1.04E-06	71
Duodenum	Rice straw	Negative	abnormal morphology of respiratory system	1.21E-06	70
Duodenum	Rice straw	Negative	activation of DNA endogenous promoter	1.41E-06	220
Duodenum	Rice straw	Negative	apoptosis of tumor cell lines	1.41E-06	220
Duodenum	Rice straw	Negative	neuromuscular disease	1.46E-06	168
Duodenum	Rice straw	Negative	proliferation of neuronal cells	1.46E-06	117
Duodenum	Rice	Negative	morphology of cardiovascular system	1.84E-06	124

	straw				
Duodenum	Rice straw	Negative	migration of connective tissue cells	1.84E-06	46
Duodenum	Rice straw	Negative	proliferation of connective tissue cells	2.44E-06	119
Duodenum	Rice straw	Negative	morphology of muscle	2.84E-06	74
Duodenum	Rice straw	Negative	transport of carbohydrate	3.04E-06	48
Duodenum	Rice straw	Negative	small GTPase mediated signal transduction	3.30E-06	46
Duodenum	Rice straw	Negative	abnormal morphology of cardiovascular system	3.60E-06	114
Duodenum	Rice straw	Negative	development of body trunk	3.67E-06	203
Duodenum	Rice straw	Negative	chemotaxis of cells	3.87E-06	107
Duodenum	Rice straw	Negative	transport of monosaccharide	3.90E-06	41
Duodenum	Rice straw	Negative	transport of D-glucose	4.18E-06	38
Duodenum	Rice straw	Negative	cognition	4.99E-06	94
Duodenum	Rice straw	Negative	formation of muscle	5.04E-06	84
Duodenum	Rice straw	Negative	protein kinase cascade	6.02E-06	88
Duodenum	Rice straw	Negative	differentiation of connective tissue	6.07E-06	140
Duodenum	Rice straw	Negative	binding of DNA	6.35E-06	105
Duodenum	Rice straw	Negative	cell survival	6.35E-06	244
Duodenum	Rice straw	Negative	chemotaxis	6.53E-06	109
Duodenum	Rice straw	Negative	development of connective tissue	6.53E-06	79
Duodenum	Rice straw	Negative	homing of cells	7.07E-06	111
Duodenum	Rice straw	Negative	cell movement of carcinoma cell lines	8.02E-06	49
Duodenum	Rice straw	Negative	branching of cells	1.10E-05	78
Duodenum	Rice	Negative	development of cytoplasm	1.10E-05	94

	straw				
Duodenum	Rice straw	Negative	cell viability	1.31E-05	228
Duodenum	Rice straw	Negative	differentiation of connective tissue cells	1.39E-05	124
Duodenum	Rice straw	Negative	learning	1.39E-05	86
Duodenum	Rice straw	Negative	formation of skin	1.52E-05	77
Duodenum	Rice straw	Negative	growth of connective tissue	1.55E-05	124
Duodenum	Rice straw	Negative	transactivation	1.55E-05	113
Duodenum	Rice straw	Negative	homing	1.83E-05	112
Duodenum	Rice straw	Negative	colony formation of cells	2.07E-05	98
Duodenum	Rice straw	Negative	multiple congenital anomalies	2.19E-05	86
Duodenum	Rice straw	Negative	formation of cytoskeleton	2.27E-05	78
Duodenum	Rice straw	Negative	phosphorylation of protein	2.42E-05	138
Duodenum	Rice straw	Negative	differentiation of epithelial tissue	2.42E-05	75
Duodenum	Rice straw	Negative	migration of fibroblasts	2.67E-05	36
Duodenum	Rice straw	Negative	development of head	2.69E-05	177
Duodenum	Rice straw	Negative	development of body axis	2.72E-05	188
Duodenum	Rice straw	Negative	morphogenesis of neurons	2.86E-05	86
Duodenum	Rice straw	Negative	cell transformation	2.98E-05	92
Duodenum	Rice straw	Negative	invasion of cells	3.00E-05	159
Duodenum	Rice straw	Negative	morphology of head	3.00E-05	160
Duodenum	Rice straw	Negative	migration of carcinoma cell lines	3.05E-05	41
Duodenum	Rice straw	Negative	morphology of bone	3.24E-05	90
Duodenum	Rice	Negative	apoptosis of fibroblast cell lines	3.33E-05	69

	straw				
Duodenum	Rice straw	Negative	morphology of heart	4.28E-05	82
Duodenum	Rice straw	Negative	behavior	4.28E-05	177
Duodenum	Rice straw	Negative	proliferation of muscle cells	4.43E-05	76
Duodenum	Rice straw	Negative	morphology of central nervous system	4.70E-05	100
Duodenum	Rice straw	Negative	abnormal morphology of head	4.80E-05	152
Duodenum	Rice straw	Negative	transactivation of RNA	4.82E-05	105
Duodenum	Rice straw	Negative	abnormal morphology of body cavity	5.17E-05	200
Duodenum	Rice straw	Negative	formation of actin filaments	6.27E-05	64
Duodenum	Rice straw	Negative	sprouting	6.41E-05	78
Duodenum	Rice straw	Negative	midline defect	6.89E-05	57
Duodenum	Rice straw	Negative	binding of protein binding site	6.99E-05	61
Duodenum	Rice straw	Negative	breast or colorectal cancer	6.99E-05	758
Duodenum	Rice straw	Negative	cell death of connective tissue cells	7.11E-05	114
Duodenum	Rice straw	Negative	colony formation	7.37E-05	103
Duodenum	Rice straw	Negative	morphology of fibroblast cell lines	7.48E-05	24
Duodenum	Rice straw	Negative	synthesis of lipid	7.48E-05	132
Duodenum	Rice straw	Negative	proliferation of hepatoma cell lines	7.60E-05	45
Duodenum	Rice straw	Negative	formation of lung	7.63E-05	63
Duodenum	Rice straw	Negative	proliferation of tumor cell lines	7.69E-05	260
Duodenum	Rice straw	Negative	epithelial-mesenchymal transition of tumor cell lines	7.79E-05	29
Duodenum	Rice straw	Negative	respiratory system development	7.87E-05	70
Duodenum	Rice	Negative	abnormal morphology of vasculature	7.87E-05	38

	straw				
Duodenum	Rice straw	Negative	morphology of vasculature	7.87E-05	39
Duodenum	Rice straw	Negative	abnormal morphology of epithelial tissue	7.87E-05	83
Duodenum	Rice straw	Negative	morphogenesis of neurites	7.87E-05	83
Duodenum	Rice straw	Negative	differentiation of epithelial cells	8.13E-05	65
Duodenum	Rice straw	Negative	cartilage development	8.24E-05	35
Duodenum	Rice straw	Negative	formation of actin stress fibers	9.20E-05	52
Duodenum	Rice straw	Negative	growth of neurites	9.44E-05	90
Duodenum	Rice straw	Negative	morphology of body cavity	1.01E-04	206
Duodenum	Rice straw	Negative	growth of embryonic tissue	1.02E-04	58
Duodenum	Rice straw	Negative	transformation of fibroblast cell lines	1.02E-04	55
Duodenum	Rice straw	Negative	abnormal morphology of muscle	1.13E-04	65
Duodenum	Rice straw	Negative	function of cardiovascular system	1.19E-04	74
Duodenum	Rice straw	Negative	abnormal morphology of pulmonary alveolus	1.23E-04	29
Duodenum	Rice straw	Negative	Growth Failure	1.24E-04	112
Duodenum	Rice straw	Negative	growth of epithelial tissue	1.37E-04	125
Duodenum	Rice straw	Negative	abnormal morphology of heart	1.44E-04	76
Duodenum	Rice straw	Negative	abdominal adenocarcinoma	1.55E-04	886
Duodenum	Rice straw	Negative	morphology of lung	1.74E-04	42
Duodenum	Rice straw	Negative	abnormal morphology of lung	1.78E-04	41
Duodenum	Rice straw	Negative	proliferation of dermal cells	1.81E-04	40
Duodenum	Rice straw	Negative	formation of filaments	1.85E-04	78
Duodenum	Rice	Negative	outgrowth of neurites	1.95E-04	79

	straw				
Duodenum	Rice straw	Negative	cell movement of phagocytes	1.96E-04	103
Duodenum	Rice straw	Negative	morphology of neuroglia	1.96E-04	29
Duodenum	Rice straw	Negative	colony formation of tumor cell lines	2.01E-04	57
Duodenum	Rice straw	Negative	oxidation of fatty acid	2.04E-04	39
Duodenum	Rice straw	Negative	cell movement of fibroblast cell lines	2.05E-04	38
Duodenum	Rice straw	Negative	apoptosis of neuroblastoma cell lines	2.05E-04	30
Duodenum	Rice straw	Negative	fibrogenesis	2.12E-04	80
Duodenum	Rice straw	Negative	abnormal morphology of bone	2.36E-04	84
Duodenum	Rice straw	Negative	cell spreading	2.36E-04	57
Duodenum	Rice straw	Negative	proliferation of cervical cancer cell lines	2.41E-04	44
Duodenum	Rice straw	Negative	cell movement of breast cancer cell lines	2.60E-04	59
Duodenum	Rice straw	Negative	quantity of connective tissue cells	2.60E-04	50
Duodenum	Rice straw	Negative	formation of embryonic tissue	2.60E-04	60
Duodenum	Rice straw	Negative	metabolism of carbohydrate	2.60E-04	114
Duodenum	Rice straw	Negative	organization of actin cytoskeleton	2.60E-04	61
Duodenum	Rice straw	Negative	gastrointestinal tract cancer	2.61E-04	764
Duodenum	Rice straw	Negative	transport of protein	2.82E-04	61
Duodenum	Rice straw	Negative	axonogenesis	2.89E-04	48
Duodenum	Rice straw	Negative	Gastrointestinal Tract Cancer and Tumors	2.94E-04	775
Duodenum	Rice straw	Negative	morphology of right ventricle	3.00E-04	17
Duodenum	Rice straw	Negative	differentiation of muscle	3.00E-04	61
Duodenum	Rice	Negative	outgrowth of cells	3.00E-04	83

	straw				
Duodenum	Rice straw	Negative	degeneration of cells	3.11E-04	68
Duodenum	Rice straw	Negative	retraction of cellular protrusions	3.16E-04	22
Duodenum	Rice straw	Negative	concentration of fatty acid	3.16E-04	57
Duodenum	Rice straw	Negative	adenocarcinoma	3.20E-04	965
Duodenum	Rice straw	Negative	development of lymphatic system	3.20E-04	70
Duodenum	Rice straw	Negative	development of embryonic cells	3.33E-04	18
Duodenum	Rice straw	Negative	abnormal morphology of interalveolar septa	3.38E-04	14
Duodenum	Rice straw	Negative	cell death of neuroblastoma cell lines	3.38E-04	42
Duodenum	Rice straw	Negative	morphology of brain	3.43E-04	88
Duodenum	Rice straw	Negative	development of cardiovascular tissue	3.53E-04	72
Duodenum	Rice straw	Negative	cell death of epithelial cells	3.66E-04	93
Duodenum	Rice straw	Negative	Bleeding	3.71E-04	81
Duodenum	Rice straw	Negative	cell movement of myeloid cells	3.71E-04	100
Duodenum	Rice straw	Negative	proliferation of cancer cells	3.82E-04	71
Duodenum	Rice straw	Negative	growth of lesion	3.90E-04	141
Duodenum	Rice straw	Negative	gastrointestinal carcinoma	4.03E-04	687
Duodenum	Rice straw	Negative	growth of skin	4.08E-04	46
Duodenum	Rice straw	Negative	development of digestive system	4.08E-04	74
Duodenum	Rice straw	Negative	endothelial cell development	4.19E-04	69
Duodenum	Rice straw	Negative	morphology of blood vessel	4.21E-04	57
Duodenum	Rice straw	Negative	Organ Degeneration	4.42E-04	92
Duodenum	Rice	Negative	morphology of vessel	4.56E-04	61

	straw				
Duodenum	Rice straw	Negative	exocytosis	4.71E-04	41
Duodenum	Rice straw	Negative	congenital anomaly of digestive system	4.73E-04	48
Duodenum	Rice straw	Negative	hyperplasia of skin	5.17E-04	31
Duodenum	Rice straw	Negative	growth of embryo	5.20E-04	89
Duodenum	Rice straw	Negative	growth of tumor	5.24E-04	140
Duodenum	Rice straw	Negative	Rho protein signal transduction	5.25E-04	22
Duodenum	Rice straw	Negative	cell viability of cerebral cortex cells	5.29E-04	19
Duodenum	Rice straw	Negative	differentiation of bone	5.37E-04	78
Duodenum	Rice straw	Negative	congenital malformation of skeleton	5.38E-04	80
Duodenum	Rice straw	Negative	secretory pathway	5.40E-04	42
Duodenum	Rice straw	Negative	mitosis	5.40E-04	92
Duodenum	Rice straw	Negative	proliferation of smooth muscle cells	5.67E-04	57
Duodenum	Rice straw	Negative	proliferation of fibroblast cell lines	5.67E-04	80
Duodenum	Rice straw	Negative	colon cancer	5.73E-04	567
Duodenum	Rice straw	Negative	proliferation of epidermal cells	5.87E-04	37
Duodenum	Rice straw	Negative	invasion of carcinoma cell lines	5.87E-04	42
Duodenum	Rice straw	Negative	morphology of connective tissue	5.87E-04	86
Duodenum	Rice straw	Negative	colon tumor	5.87E-04	570
Duodenum	Rice straw	Negative	abnormal morphology of neuroglia	6.05E-04	22
Duodenum	Rice straw	Negative	cellularity	6.05E-04	18
Duodenum	Rice straw	Negative	proliferation of embryonic cell lines	6.11E-04	35
Duodenum	Rice	Negative	cell cycle progression	6.22E-04	178

	straw				
Duodenum	Rice straw	Negative	abnormal morphology of embryonic tissue	6.25E-04	100
Duodenum	Rice straw	Negative	tubulation of cells	6.25E-04	33
Duodenum	Rice straw	Negative	scattering	6.28E-04	24
Duodenum	Rice straw	Negative	differentiation of bone cells	6.34E-04	77
Duodenum	Rice straw	Negative	proliferation of epithelial cells	6.35E-04	91
Duodenum	Rice straw	Negative	proliferation of fibroblasts	6.44E-04	68
Duodenum	Rice straw	Negative	abdominal carcinoma	6.58E-04	890
Duodenum	Rice straw	Negative	colorectal neoplasia	6.61E-04	658
Duodenum	Rice straw	Negative	quantity of carbohydrate	6.73E-04	95
Duodenum	Rice straw	Negative	proliferation of keratinocytes	6.80E-04	34
Duodenum	Rice straw	Negative	abnormal morphology of right ventricle	6.89E-04	15
Duodenum	Rice straw	Negative	development of endothelial tissue	6.98E-04	70
Duodenum	Rice straw	Negative	proliferation of tumor cells	7.25E-04	87
Duodenum	Rice straw	Negative	cell death of fibroblast cell lines	7.25E-04	80
Duodenum	Rice straw	Negative	cell death of epithelial cell lines	7.27E-04	56
Duodenum	Rice straw	Negative	differentiation of neural precursor cells	7.29E-04	12
Duodenum	Rice straw	Negative	cell movement of lung cancer cell lines	7.33E-04	27
Duodenum	Rice straw	Negative	fatty acid metabolism	7.45E-04	108
Duodenum	Rice straw	Negative	differentiation of tumor cell lines	7.45E-04	72
Duodenum	Rice straw	Negative	abnormal morphology of body wall	7.63E-04	25
Duodenum	Rice straw	Negative	necrosis of epithelial tissue	7.67E-04	106
Duodenum	Rice	Negative	cell movement of blood cells	7.67E-04	151

	straw				
Duodenum	Rice straw	Negative	abnormal morphology of blood vessel	7.67E-04	53
Duodenum	Rice straw	Negative	abnormal morphology of central nervous system	7.73E-04	89
Duodenum	Rice straw	Negative	morphology of cardiovascular tissue	7.99E-04	22
Duodenum	Rice straw	Negative	mass of connective tissue	8.02E-04	42
Duodenum	Rice straw	Negative	craniofacial abnormality	8.04E-04	68
Duodenum	Rice straw	Negative	differentiation of embryonic tissue	8.15E-04	51
Duodenum	Rice straw	Negative	formation of focal adhesions	8.18E-04	34
Duodenum	Rice straw	Negative	apoptosis of neurons	8.60E-04	81
Duodenum	Rice straw	Negative	proliferation of embryonic cells	8.77E-04	50
Duodenum	Rice straw	Negative	maturation of chondrocytes	9.06E-04	11
Duodenum	Rice straw	Negative	metabolism of membrane lipid derivative	9.16E-04	73
Duodenum	Rice straw	Negative	cell viability of cortical neurons	9.35E-04	14
Duodenum	Rice straw	Negative	quantity of cells	9.44E-04	283
Duodenum	Rice straw	Negative	development of abdomen	9.51E-04	104
Duodenum	Rice straw	Negative	migration of blood cells	9.63E-04	150
Duodenum	Rice straw	Negative	formation of muscle cells	9.70E-04	38
Duodenum	Rice straw	Negative	morphology of endothelial tissue	9.76E-04	21
Duodenum	Rice straw	Negative	quantity of bone cells	9.80E-04	35
Duodenum	Rice straw	Negative	morphology of endothelial cells	1.01E-03	17
Duodenum	Rice straw	Negative	I-kappaB kinase/NF-kappaB cascade	1.01E-03	37
Duodenum	Rice straw	Negative	colorectal cancer	1.05E-03	647
Duodenum	Rice	Negative	migration of fibroblast cell lines	1.09E-03	30

	straw				
Duodenum	Rice straw	Negative	cell viability of tumor cell lines	1.10E-03	135
Duodenum	Rice straw	Negative	scattering of cells	1.22E-03	20
Duodenum	Rice straw	Negative	invasion of tumor cell lines	1.22E-03	117
Duodenum	Rice straw	Negative	hypoglycemia	1.22E-03	27
Duodenum	Rice straw	Negative	tubulation of endothelial cells	1.22E-03	27
Duodenum	Rice straw	Negative	growth of malignant tumor	1.24E-03	80
Duodenum	Rice straw	Negative	leukocyte migration	1.27E-03	149
Duodenum	Rice straw	Negative	quantity of connective tissue	1.27E-03	107
Duodenum	Rice straw	Negative	congenital malformation of face	1.29E-03	33
Duodenum	Rice straw	Negative	synthesis of polysaccharide	1.29E-03	38
Duodenum	Rice straw	Negative	Viral Infection	1.32E-03	259
Duodenum	Rice straw	Negative	cytostasis	1.37E-03	55
Duodenum	Rice straw	Negative	branching of neurites	1.37E-03	54
Duodenum	Rice straw	Negative	heart rate	1.37E-03	54
Duodenum	Rice straw	Negative	quantity of muscle	1.38E-03	29
Duodenum	Rice straw	Negative	apoptosis of connective tissue cells	1.43E-03	57
Duodenum	Rice straw	Negative	apoptosis of epithelial cell lines	1.44E-03	45
Duodenum	Rice straw	Negative	quantity of diacylglycerol	1.45E-03	16
Duodenum	Rice straw	Negative	cell movement of hepatoma cell lines	1.45E-03	22
Duodenum	Rice straw	Negative	shape change of fibroblast cell lines	1.45E-03	22
Duodenum	Rice straw	Negative	vascularization	1.45E-03	47
Duodenum	Rice	Negative	uptake of fatty acid	1.45E-03	19

	straw				
Duodenum	Rice straw	Negative	migration of muscle cells	1.45E-03	35
Duodenum	Rice straw	Negative	morphology of muscle cells	1.45E-03	35
Duodenum	Rice straw	Negative	growth of organism	1.47E-03	143
Duodenum	Rice straw	Negative	bleeding of brain	1.47E-03	18
Duodenum	Rice straw	Negative	uptake of carbohydrate	1.51E-03	56
Duodenum	Rice straw	Negative	synthesis of carbohydrate	1.51E-03	78
Duodenum	Rice straw	Negative	colorectal carcinoma	1.52E-03	562
Duodenum	Rice straw	Negative	shape change of neurites	1.52E-03	55
Duodenum	Rice straw	Negative	development of lymphatic system component	1.54E-03	59
Duodenum	Rice straw	Negative	cell death of brain	1.62E-03	56
Duodenum	Rice straw	Negative	development of urinary tract	1.64E-03	55
Duodenum	Rice straw	Negative	damage of central nervous system	1.74E-03	39
Duodenum	Rice straw	Negative	shape change of neurons	1.74E-03	56
Duodenum	Rice straw	Negative	branching of neurons	1.76E-03	55
Duodenum	Rice straw	Negative	retraction of plasma membrane projections	1.76E-03	18
Duodenum	Rice straw	Negative	abnormal morphology of endothelial tissue	1.77E-03	17
Duodenum	Rice straw	Negative	abnormal morphology of brain	1.78E-03	81
Duodenum	Rice straw	Negative	abnormality of respiratory system	1.79E-03	21
Duodenum	Rice straw	Negative	differentiation of neurons	1.79E-03	76
Duodenum	Rice straw	Negative	development of gastrointestinal tract	1.80E-03	51
Duodenum	Rice straw	Negative	exocytosis by cells	1.92E-03	28
Duodenum	Rice	Negative	morphology of skeletal muscle	1.92E-03	28

	straw				
Duodenum	Rice straw	Negative	keratosis	1.92E-03	27
Duodenum	Rice straw	Negative	schizophrenia spectrum disorder	1.97E-03	81
Duodenum	Rice straw	Negative	Schizophrenia	1.97E-03	78
Duodenum	Rice straw	Negative	differentiation of muscle cell lines	2.00E-03	34
Duodenum	Rice straw	Negative	formation of lamellipodia	2.00E-03	34
Duodenum	Rice straw	Negative	differentiation of muscle cells	2.02E-03	54
Duodenum	Rice straw	Negative	morphology of neurons	2.03E-03	77
Duodenum	Rice straw	Negative	reorganization of cytoskeleton	2.03E-03	39
Duodenum	Rice straw	Negative	cycling of centrosome	2.06E-03	21
Duodenum	Rice straw	Negative	strength of muscle	2.06E-03	18
Duodenum	Rice straw	Negative	migration of breast cancer cell lines	2.08E-03	48
Duodenum	Rice straw	Negative	iris coloboma	2.08E-03	5
Duodenum	Rice straw	Negative	lack of salivary gland	2.08E-03	5
Duodenum	Rice straw	Negative	concentration of lipid	2.10E-03	138
Duodenum	Rice straw	Negative	differentiation of nervous system	2.12E-03	89
Duodenum	Rice straw	Negative	quantity of bone	2.25E-03	48
Duodenum	Rice straw	Negative	scattering of tumor cell lines	2.26E-03	11
Duodenum	Rice straw	Negative	cell movement of muscle cells	2.26E-03	37
Duodenum	Rice straw	Negative	morphology of nervous tissue	2.31E-03	79
Duodenum	Rice straw	Negative	hypertrophy of cardiomyocytes	2.33E-03	29
Duodenum	Rice straw	Negative	cell death of B-lymphocyte derived cell lines	2.38E-03	28
Duodenum	Rice	Negative	cell death of cerebral cortex cells	2.40E-03	45

	straw				
Duodenum	Rice straw	Negative	quantity of muscle cells	2.40E-03	26
Duodenum	Rice straw	Negative	maturation of cells	2.40E-03	82
Duodenum	Rice straw	Negative	apoptosis of skin	2.51E-03	22
Duodenum	Rice straw	Negative	morphology of central nervous system cells	2.53E-03	30
Duodenum	Rice straw	Negative	migration of embryonic cell lines	2.56E-03	20
Duodenum	Rice straw	Negative	morphology of mouth	2.60E-03	29
Duodenum	Rice straw	Negative	abnormal morphology of axial skeleton	2.61E-03	39
Duodenum	Rice straw	Negative	dysgenesis	2.64E-03	95
Duodenum	Rice straw	Negative	cell movement of neutrophils	2.70E-03	56
Duodenum	Rice straw	Negative	damage of brain	2.70E-03	37
Duodenum	Rice straw	Negative	gastrointestinal adenocarcinoma	2.77E-03	639
Duodenum	Rice straw	Negative	dendritic growth/branching	2.77E-03	43
Duodenum	Rice straw	Negative	apoptosis of tumor cells	2.78E-03	54
Duodenum	Rice straw	Negative	proliferation of hematopoietic progenitor cells	2.78E-03	47
Duodenum	Rice straw	Negative	formation of kidney	2.81E-03	53
Duodenum	Rice straw	Negative	morphology of skeleton	2.81E-03	53
Duodenum	Rice straw	Negative	quantity of centrosome	2.84E-03	14
Duodenum	Rice straw	Negative	morphology of axial skeleton	2.87E-03	40
Duodenum	Rice straw	Negative	epithelial-mesenchymal transition	2.90E-03	41
Duodenum	Rice straw	Negative	size of head	2.90E-03	41
Duodenum	Rice straw	Negative	morphology of second branchial arch	2.92E-03	10
Duodenum	Rice	Negative	cell death of stem cells	2.92E-03	20

	straw				
Duodenum	Rice straw	Negative	metabolism of polysaccharide	2.97E-03	46
Duodenum	Rice straw	Negative	apoptosis of cerebral cortex cells	2.98E-03	24
Duodenum	Rice straw	Negative	oscillation of Ca2+	2.98E-03	16
Duodenum	Rice straw	Negative	hyperplasia of epidermis	2.99E-03	25
Duodenum	Rice straw	Negative	weight gain	3.08E-03	58
Duodenum	Rice straw	Negative	cell movement of leukocytes	3.12E-03	130
Duodenum	Rice straw	Negative	shape change of tumor cell lines	3.12E-03	32
Duodenum	Rice straw	Negative	cell death of heart cells	3.17E-03	43
Duodenum	Rice straw	Negative	Hypoplasia	3.17E-03	89
Duodenum	Rice straw	Negative	uptake of monosaccharide	3.27E-03	51
Duodenum	Rice straw	Negative	ubiquitination	3.34E-03	62
Duodenum	Rice straw	Negative	cell death of neuroglia	3.35E-03	26
Duodenum	Rice straw	Negative	anoikis	3.37E-03	25
Duodenum	Rice straw	Negative	peripheral vascular disease	3.37E-03	69
Duodenum	Rice straw	Negative	binding of DNA fragment	3.48E-03	17
Duodenum	Rice straw	Negative	separation of mitotic centrosome	3.56E-03	4
Duodenum	Rice straw	Negative	apoptosis of neuroglia	3.56E-03	21
Duodenum	Rice straw	Negative	neurodegeneration of cerebral cortex	3.57E-03	15
Duodenum	Rice straw	Negative	ubiquitination of protein	3.63E-03	61
Duodenum	Rice straw	Negative	colorectal adenocarcinoma	3.67E-03	542
Duodenum	Rice straw	Negative	development of sensory organ	3.67E-03	98
Duodenum	Rice	Negative	cell death of heart	3.67E-03	44

	straw				
Duodenum	Rice straw	Negative	skin abnormality	3.67E-03	44
Duodenum	Rice straw	Negative	morphogenesis of epithelial tissue	3.77E-03	32
Duodenum	Rice straw	Negative	morphology of rib	3.88E-03	18
Duodenum	Rice straw	Negative	growth of cartilage tissue	3.88E-03	20
Duodenum	Rice straw	Negative	muscular hypertrophy	3.91E-03	40
Duodenum	Rice straw	Negative	abnormal morphology of dilated right ventricle	3.94E-03	10
Duodenum	Rice straw	Negative	limb development	3.94E-03	45
Duodenum	Rice straw	Negative	abnormal morphology of skeleton	3.96E-03	51
Duodenum	Rice straw	Negative	closure of embryonic tissue	4.12E-03	30
Duodenum	Rice straw	Negative	hypertrophy of heart cells	4.14E-03	32
Duodenum	Rice straw	Negative	cell viability of fibroblast cell lines	4.25E-03	25
Duodenum	Rice straw	Negative	secretion of alpha granules	4.31E-03	6
Duodenum	Rice straw	Negative	abnormal morphology of neurons	4.31E-03	68
Duodenum	Rice straw	Negative	formation of thymus gland	4.31E-03	33
Duodenum	Rice straw	Negative	autosomal dominant disease	4.31E-03	109
Duodenum	Rice straw	Negative	abnormal morphology of artery	4.31E-03	31
Duodenum	Rice straw	Negative	differentiation of keratinocytes	4.31E-03	31
Duodenum	Rice straw	Negative	closure of neural tube	4.40E-03	28
Duodenum	Rice straw	Negative	cardiogenesis	4.41E-03	83
Duodenum	Rice straw	Negative	cell death of tumor cells	4.41E-03	67
Duodenum	Rice straw	Negative	tubulation of vascular endothelial cells	4.41E-03	20
Duodenum	Rice	Negative	metastatic colorectal cancer	4.42E-03	38

	straw				
Duodenum	Rice straw	Negative	cell death of brain cells	4.48E-03	51
Duodenum	Rice straw	Negative	abnormal morphology of vascular endothelial cells	4.48E-03	12
Duodenum	Rice straw	Negative	concentration of Ca2+	4.48E-03	27
Duodenum	Rice straw	Negative	hepatocellular carcinoma	4.54E-03	624
Duodenum	Rice straw	Negative	sensory system development	4.73E-03	24
Duodenum	Rice straw	Negative	cell death of skin	4.73E-03	23
Duodenum	Rice straw	Negative	abnormal morphology of rib	4.74E-03	17
Duodenum	Rice straw	Negative	cell death of muscle cells	4.74E-03	61
Duodenum	Rice straw	Negative	GATA2 deficiency	4.74E-03	7
Duodenum	Rice straw	Negative	cell death of central nervous system cells	4.74E-03	54
Duodenum	Rice straw	Negative	migration of hepatoma cell lines	4.74E-03	19
Duodenum	Rice straw	Negative	proliferation of chondrocytes	4.74E-03	19
Duodenum	Rice straw	Negative	cell death of hepatoma cell lines	4.74E-03	34
Duodenum	Rice straw	Negative	proliferation of epithelial cell lines	4.80E-03	44
Duodenum	Rice straw	Negative	damage of nervous system	4.80E-03	43
Duodenum	Rice straw	Negative	morphology of skull	4.80E-03	43
Duodenum	Rice straw	Negative	function of endothelial cells	4.81E-03	13
Duodenum	Rice straw	Negative	synthesis of phospholipid	4.84E-03	35
Duodenum	Rice straw	Negative	differentiation of epidermal cells	5.01E-03	33
Duodenum	Rice straw	Negative	hyperplasia of tissue	5.12E-03	38
Duodenum	Rice straw	Negative	frequency of head and neck tumor	5.14E-03	5
Duodenum	Rice	Negative	long-term potentiation of granule cells	5.14E-03	5

	straw				
Duodenum	Rice straw	Negative	necrosis of tumor	5.16E-03	68
Duodenum	Rice straw	Negative	abnormal morphology of skull	5.16E-03	42
Duodenum	Rice straw	Negative	oxidation of lipid	5.16E-03	41
Duodenum	Rice straw	Negative	binding of endothelial cells	5.22E-03	23
Duodenum	Rice straw	Negative	formation of cells	5.22E-03	161
Duodenum	Rice straw	Negative	dyspnea	5.22E-03	28
Duodenum	Rice straw	Negative	autophagy	5.31E-03	67
Duodenum	Rice straw	Negative	mass of adipose tissue	5.43E-03	37
Duodenum	Rice straw	Negative	apoptosis of B-lymphocyte derived cell lines	5.60E-03	26
Duodenum	Rice straw	Negative	papilloma	5.60E-03	26
Duodenum	Rice straw	Negative	Edema	5.64E-03	62
Duodenum	Rice straw	Negative	cell death of muscle	5.64E-03	62
Duodenum	Rice straw	Negative	quantity of epithelial tissue	5.71E-03	35
Duodenum	Rice straw	Negative	engulfment of cells	5.72E-03	72
Duodenum	Rice straw	Negative	grip strength	5.72E-03	16
Duodenum	Rice straw	Negative	cytostasis of tumor cell lines	5.73E-03	32
Duodenum	Rice straw	Negative	migration of lung cancer cell lines	5.77E-03	21
Duodenum	Rice straw	Negative	cell movement of cancer cells	5.80E-03	30
Duodenum	Rice straw	Negative	benign neoplasia	5.84E-03	152
Duodenum	Rice straw	Negative	atelectasis	5.84E-03	18
Duodenum	Rice straw	Negative	oral cancer	5.87E-03	43
Duodenum	Rice	Negative	fusion of cellular membrane	5.88E-03	15

	straw				
Duodenum	Rice straw	Negative	chemotaxis of phagocytes	5.90E-03	54
Duodenum	Rice straw	Negative	targeting of protein	5.92E-03	27
Duodenum	Rice straw	Negative	dilation of heart ventricle	5.92E-03	14
Duodenum	Rice straw	Negative	abnormal morphology of skeletal muscle	6.14E-03	26
Duodenum	Rice straw	Negative	hepatobiliary system cancer	6.20E-03	644
Duodenum	Rice straw	Negative	cell movement of tumor cells	6.20E-03	36
Duodenum	Rice straw	Negative	formation of epidermis	6.20E-03	36
Duodenum	Rice straw	Negative	metabolism of phospholipid	6.20E-03	44
Duodenum	Rice straw	Negative	respiratory failure	6.28E-03	25
Duodenum	Rice straw	Negative	migration of smooth muscle cells	6.30E-03	30
Duodenum	Rice straw	Negative	cell viability of central nervous system cells	6.41E-03	24
Duodenum	Rice straw	Negative	smooth muscle tumor	6.45E-03	55
Duodenum	Rice straw	Negative	length of cells	6.52E-03	22
Duodenum	Rice straw	Negative	mass of organism	6.61E-03	60
Duodenum	Rice straw	Negative	long-term memory	6.62E-03	16
Duodenum	Rice straw	Negative	retraction of neurites	6.62E-03	16
Duodenum	Rice straw	Negative	Neurodegeneration	6.62E-03	56
Duodenum	Rice straw	Negative	differentiation of brain	6.67E-03	18
Duodenum	Rice straw	Negative	cell movement of smooth muscle cells	6.73E-03	32
Duodenum	Rice straw	Negative	morphology of skin	6.83E-03	50
Duodenum	Rice straw	Negative	morphology of astrocytes	7.09E-03	13
Duodenum	Rice	Negative	cell viability of osteoblasts	7.19E-03	7

	straw				
Duodenum	Rice straw	Negative	apoptosis of dermal cells	7.24E-03	20
Duodenum	Rice straw	Negative	abnormal morphology of fornix	7.25E-03	6
Duodenum	Rice straw	Negative	metabolism of nucleic acid component or derivative	7.25E-03	89
Duodenum	Rice straw	Negative	cell movement of embryonic cell lines	7.25E-03	23
Duodenum	Rice straw	Negative	cell viability of brain cells	7.30E-03	21
Duodenum	Rice straw	Negative	MAPKKK cascade	7.32E-03	39
Duodenum	Rice straw	Negative	liver tumor	7.67E-03	644
Duodenum	Rice straw	Negative	cell death of cortical neurons	7.69E-03	34
Duodenum	Rice straw	Negative	chondrogenesis	7.73E-03	11
Duodenum	Rice straw	Negative	cell death of pheochromocytoma cell lines	7.73E-03	25
Duodenum	Rice straw	Negative	fusion of muscle cells	7.73E-03	16
Duodenum	Rice straw	Negative	invasion of fibroblast cell lines	7.73E-03	16
Duodenum	Rice straw	Negative	differentiation of skin	7.73E-03	35
Duodenum	Rice straw	Negative	proliferation of endothelial cells	7.85E-03	56
Duodenum	Rice straw	Negative	muscle tumor	7.90E-03	64
Duodenum	Rice straw	Negative	neovascularization	7.98E-03	32
Duodenum	Rice straw	Negative	apoptosis of muscle	8.05E-03	48
Duodenum	Rice straw	Negative	ruffling	8.08E-03	23
Duodenum	Rice straw	Negative	seizure disorder	8.22E-03	81
Duodenum	Rice straw	Negative	degeneration of nervous system	8.28E-03	52
Duodenum	Rice straw	Negative	proliferation of myoblasts	8.30E-03	12
Duodenum	Rice	Negative	differentiation of brain cells	8.35E-03	17

	straw				
Duodenum	Rice straw	Negative	apoptosis of renal glomerulus	8.42E-03	14
Duodenum	Rice straw	Negative	cell spreading of fibroblast cell lines	8.42E-03	14
Duodenum	Rice straw	Negative	migration of cancer cells	8.42E-03	37
Duodenum	Rice straw	Negative	hypertrophy of cardiac muscle	8.42E-03	31
Duodenum	Rice straw	Negative	colon carcinoma	8.45E-03	523
Duodenum	Rice straw	Negative	apoptosis of glomerular cells	8.45E-03	13
Duodenum	Rice straw	Negative	hypoactivity of mice	8.62E-03	32
Duodenum	Rice straw	Negative	hypertrophy of tissue	8.68E-03	47
Duodenum	Rice straw	Negative	liver cancer	8.75E-03	639
Duodenum	Rice straw	Negative	abnormal morphology of type II pneumocytes	8.86E-03	9
Duodenum	Rice straw	Negative	apoptosis of hepatoma cell lines	8.92E-03	30
Duodenum	Rice straw	Negative	development of antigen presenting cells	8.92E-03	16
Duodenum	Rice straw	Negative	morphology of joint	8.92E-03	16
Duodenum	Rice straw	Negative	assembly of protein-protein complex	9.02E-03	36
Duodenum	Rice straw	Negative	morphology of tumor cell lines	9.02E-03	38
Duodenum	Rice straw	Negative	cell death of dermal cells	9.16E-03	21
Duodenum	Rice straw	Negative	export of molecule	9.55E-03	53
Duodenum	Rice straw	Negative	cell death of cardiomyocytes	9.67E-03	40
Duodenum	Rice straw	Negative	formation of T lymphocytes	9.76E-03	24
Duodenum	Rice straw	Negative	apoptosis of heart cells	9.78E-03	36
Duodenum	Corn stover	Positive	organismal death	2.21E-42	1009
Duodenum	Corn	Positive	proliferation of cells	3.67E-42	1445

	stover				
Duodenum	Corn stover	Positive	morbidity or mortality	5.90E-42	1017
Duodenum	Corn stover	Positive	cancer	6.24E-34	3608
Duodenum	Corn stover	Positive	cell death	9.18E-34	1289
Duodenum	Corn stover	Positive	malignant solid tumor	1.64E-32	3563
Duodenum	Corn stover	Positive	necrosis	1.83E-27	1005
Duodenum	Corn stover	Positive	morphology of cells	9.13E-27	789
Duodenum	Corn stover	Positive	apoptosis	1.45E-26	1024
Duodenum	Corn stover	Positive	organization of cytoplasm	1.36E-25	640
Duodenum	Corn stover	Positive	cell movement	5.46E-23	834
Duodenum	Corn stover	Positive	transport of molecule	8.55E-22	652
Duodenum	Corn stover	Positive	organization of cytoskeleton	1.64E-21	575
Duodenum	Corn stover	Positive	expression of RNA	6.98E-21	830
Duodenum	Corn stover	Positive	cellular homeostasis	8.55E-21	616
Duodenum	Corn stover	Positive	migration of cells	6.04E-19	738
Duodenum	Corn stover	Positive	transcription	2.31E-18	766
Duodenum	Corn stover	Positive	transcription of RNA	7.64E-18	717
Duodenum	Corn stover	Positive	differentiation of cells	1.01E-17	848
Duodenum	Corn stover	Positive	size of body	1.17E-17	365
Duodenum	Corn stover	Positive	abdominal neoplasm	1.43E-17	2910
Duodenum	Corn stover	Positive	abnormal morphology of cells	2.73E-17	511
Duodenum	Corn stover	Positive	cell survival	5.06E-17	554
Duodenum	Corn	Positive	microtubule dynamics	5.74E-17	481

	stover				
Duodenum	Corn stover	Positive	neoplasia of epithelial tissue	1.06E-16	2924
Duodenum	Corn stover	Positive	cell movement of tumor cell lines	1.34E-16	361
Duodenum	Corn stover	Positive	tumorigenesis of tissue	1.38E-16	2968
Duodenum	Corn stover	Positive	perinatal death	1.42E-16	265
Duodenum	Corn stover	Positive	epithelial cancer	1.43E-16	2897
Duodenum	Corn stover	Positive	Movement Disorders	2.78E-16	434
Duodenum	Corn stover	Positive	abdominal cancer	6.58E-16	2866
Duodenum	Corn stover	Positive	morphology of cardiovascular system	8.09E-16	272
Duodenum	Corn stover	Positive	angiogenesis	2.20E-15	376
Duodenum	Corn stover	Positive	transcription of DNA	2.61E-15	594
Duodenum	Corn stover	Positive	Viral Infection	2.62E-15	620
Duodenum	Corn stover	Positive	cell death of tumor cell lines	3.37E-15	590
Duodenum	Corn stover	Positive	cell viability	8.25E-15	511
Duodenum	Corn stover	Positive	migration of tumor cell lines	1.33E-14	300
Duodenum	Corn stover	Positive	growth of organism	1.50E-14	347
Duodenum	Corn stover	Positive	quantity of cells	3.79E-14	666
Duodenum	Corn stover	Positive	abnormal morphology of cardiovascular system	5.72E-14	246
Duodenum	Corn stover	Positive	development of body trunk	7.80E-14	443
Duodenum	Corn stover	Positive	vasculogenesis	1.47E-13	307
Duodenum	Corn stover	Positive	apoptosis of tumor cell lines	2.54E-13	473
Duodenum	Corn stover	Positive	proliferation of tumor cell lines	3.11E-13	587
Duodenum	Corn	Positive	development of vasculature	3.15E-13	187

	stover				
Duodenum	Corn stover	Positive	transactivation	3.78E-13	247
Duodenum	Corn stover	Positive	abnormal morphology of thoracic cavity	7.91E-13	249
Duodenum	Corn stover	Positive	disorder of basal ganglia	1.31E-12	314
Duodenum	Corn stover	Positive	transactivation of RNA	1.51E-12	232
Duodenum	Corn stover	Positive	abnormal morphology of body cavity	1.80E-12	445
Duodenum	Corn stover	Positive	digestive system cancer	1.84E-12	2475
Duodenum	Corn stover	Positive	invasion of cells	1.85E-12	350
Duodenum	Corn stover	Positive	formation of cellular protrusions	2.55E-12	361
Duodenum	Corn stover	Positive	digestive organ tumor	3.81E-12	2494
Duodenum	Corn stover	Positive	small GTPase mediated signal transduction	4.13E-12	92
Duodenum	Corn stover	Positive	morphology of body cavity	4.22E-12	461
Duodenum	Corn stover	Positive	neuromuscular disease	4.22E-12	353
Duodenum	Corn stover	Positive	cell death of connective tissue cells	4.43E-12	252
Duodenum	Corn stover	Positive	ubiquitination	4.61E-12	152
Duodenum	Corn stover	Positive	ubiquitination of protein	4.66E-12	150
Duodenum	Corn stover	Positive	protein kinase cascade	5.48E-12	184
Duodenum	Corn stover	Positive	neonatal death	1.95E-11	189
Duodenum	Corn stover	Positive	proliferation of connective tissue cells	3.43E-11	245
Duodenum	Corn stover	Positive	neurological signs	3.82E-11	266
Duodenum	Corn stover	Positive	degeneration of cells	4.18E-11	152
Duodenum	Corn stover	Positive	development of cytoplasm	5.40E-11	196
Duodenum	Corn	Positive	cell viability of tumor cell lines	5.46E-11	311

	stover				
Duodenum	Corn stover	Positive	morphology of nervous system	1.36E-10	311
Duodenum	Corn stover	Positive	growth of connective tissue	1.50E-10	261
Duodenum	Corn stover	Positive	dyskinesia	1.58E-10	251
Duodenum	Corn stover	Positive	cell cycle progression	1.78E-10	402
Duodenum	Corn stover	Positive	neuronal cell death	1.78E-10	274
Duodenum	Corn stover	Positive	morphology of heart	1.93E-10	173
Duodenum	Corn stover	Positive	growth of embryo	2.69E-10	199
Duodenum	Corn stover	Positive	Growth Failure	3.35E-10	243
Duodenum	Corn stover	Positive	formation of cytoskeleton	3.75E-10	161
Duodenum	Corn stover	Positive	behavior	4.74E-10	380
Duodenum	Corn stover	Positive	activation of DNA endogenous promoter	5.02E-10	455
Duodenum	Corn stover	Positive	size of embryo	6.01E-10	153
Duodenum	Corn stover	Positive	Huntington's Disease	6.97E-10	233
Duodenum	Corn stover	Positive	quantity of connective tissue	7.59E-10	243
Duodenum	Corn stover	Positive	fibrogenesis	7.86E-10	173
Duodenum	Corn stover	Positive	abnormal morphology of nervous system	1.03E-09	286
Duodenum	Corn stover	Positive	degeneration of nervous system	1.07E-09	126
Duodenum	Corn stover	Positive	abnormal morphology of heart	1.15E-09	162
Duodenum	Corn stover	Positive	concentration of lipid	1.16E-09	316
Duodenum	Corn stover	Positive	development of connective tissue	1.54E-09	157
Duodenum	Corn stover	Positive	proliferation of neuronal cells	1.55E-09	232
Duodenum	Corn	Positive	metabolism of carbohydrate	1.69E-09	248

	stover				
Duodenum	Corn stover	Positive	benign neoplasia	1.69E-09	355
Duodenum	Corn stover	Positive	seizures	1.99E-09	167
Duodenum	Corn stover	Positive	development of epithelial tissue	2.05E-09	210
Duodenum	Corn stover	Positive	organization of organelle	2.29E-09	218
Duodenum	Corn stover	Positive	differentiation of muscle cell lines	2.73E-09	77
Duodenum	Corn stover	Positive	metabolism of protein	2.98E-09	370
Duodenum	Corn stover	Positive	quantity of blood cells	3.56E-09	388
Duodenum	Corn stover	Positive	morphology of head	3.70E-09	336
Duodenum	Corn stover	Positive	growth of epithelial tissue	4.54E-09	267
Duodenum	Corn stover	Positive	Neurodegeneration	5.81E-09	132
Duodenum	Corn stover	Positive	colony formation of cells	6.46E-09	199
Duodenum	Corn stover	Positive	replication of RNA virus	6.93E-09	204
Duodenum	Corn stover	Positive	development of blood cells	8.75E-09	256
Duodenum	Corn stover	Positive	morphology of connective tissue	9.41E-09	187
Duodenum	Corn stover	Positive	formation of filaments	9.49E-09	164
Duodenum	Corn stover	Positive	invasion of tumor cell lines	1.08E-08	260
Duodenum	Corn stover	Positive	infection by Retroviridae	1.08E-08	279
Duodenum	Corn stover	Positive	seizure disorder	1.13E-08	190
Duodenum	Corn stover	Positive	colony formation	2.03E-08	213
Duodenum	Corn stover	Positive	growth of lesion	2.03E-08	305
Duodenum	Corn stover	Positive	abnormal morphology of head	2.12E-08	317
Duodenum	Corn	Positive	development of body axis	2.15E-08	392

	stover				
Duodenum	Corn stover	Positive	epileptic seizure	2.19E-08	77
Duodenum	Corn stover	Positive	differentiation of connective tissue	2.34E-08	281
Duodenum	Corn stover	Positive	development of abdomen	2.36E-08	228
Duodenum	Corn stover	Positive	growth of tumor	2.45E-08	304
Duodenum	Corn stover	Positive	apoptosis of connective tissue cells	2.45E-08	125
Duodenum	Corn stover	Positive	proliferation of fibroblasts	2.49E-08	146
Duodenum	Corn stover	Positive	Organ Degeneration	2.49E-08	197
Duodenum	Corn stover	Positive	cell death of epithelial cell lines	2.51E-08	120
Duodenum	Corn stover	Positive	quantity of leukocytes	2.56E-08	345
Duodenum	Corn stover	Positive	necrosis of epithelial tissue	2.56E-08	231
Duodenum	Corn stover	Positive	differentiation of tumor cell lines	3.44E-08	155
Duodenum	Corn stover	Positive	morphology of muscle	3.59E-08	139
Duodenum	Corn stover	Positive	metabolism of monosaccharide	4.15E-08	59
Duodenum	Corn stover	Positive	infection by RNA virus	4.25E-08	327
Duodenum	Corn stover	Positive	infection by lentivirus	4.64E-08	274
Duodenum	Corn stover	Positive	concentration of fatty acid	4.72E-08	118
Duodenum	Corn stover	Positive	replication of virus	4.72E-08	220
Duodenum	Corn stover	Positive	degeneration of neurons	4.72E-08	103
Duodenum	Corn stover	Positive	morphology of vessel	4.91E-08	128
Duodenum	Corn stover	Positive	uptake of carbohydrate	4.98E-08	122
Duodenum	Corn stover	Positive	HIV infection	5.05E-08	273
Duodenum	Corn	Positive	growth of muscle tissue	5.74E-08	152

	stover				
Duodenum	Corn stover	Positive	growth of embryonic tissue	5.92E-08	116
Duodenum	Corn stover	Positive	proliferation of muscle cells	6.51E-08	151
Duodenum	Corn stover	Positive	cell death of muscle cells	6.57E-08	138
Duodenum	Corn stover	Positive	infection of cells	6.57E-08	302
Duodenum	Corn stover	Positive	development of leukocytes	6.58E-08	229
Duodenum	Corn stover	Positive	differentiation of connective tissue cells	6.79E-08	248
Duodenum	Corn stover	Positive	mitosis	6.84E-08	196
Duodenum	Corn stover	Positive	cellular degradation	6.98E-08	108
Duodenum	Corn stover	Positive	survival of organism	7.01E-08	269
Duodenum	Corn stover	Positive	function of blood cells	7.26E-08	228
Duodenum	Corn stover	Positive	quantity of lymphocytes	8.66E-08	270
Duodenum	Corn stover	Positive	development of head	9.03E-08	364
Duodenum	Corn stover	Positive	function of leukocytes	9.36E-08	210
Duodenum	Corn stover	Positive	uptake of monosaccharide	1.01E-07	113
Duodenum	Corn stover	Positive	quantity of carbohydrate	1.04E-07	203
Duodenum	Corn stover	Positive	neuritogenesis	1.04E-07	226
Duodenum	Corn stover	Positive	autosomal recessive disease	1.12E-07	343
Duodenum	Corn stover	Positive	outgrowth of plasma membrane projections	1.14E-07	163
Duodenum	Corn stover	Positive	development of neurons	1.28E-07	295
Duodenum	Corn stover	Positive	congenital anomaly of musculoskeletal system	1.30E-07	279
Duodenum	Corn stover	Positive	cell transformation	1.31E-07	182
Duodenum	Corn	Positive	cell death of muscle	1.31E-07	140

	stover				
Duodenum	Corn stover	Positive	growth of plasma membrane projections	1.33E-07	184
Duodenum	Corn stover	Positive	morphology of bone	1.43E-07	178
Duodenum	Corn stover	Positive	secretion of molecule	1.49E-07	214
Duodenum	Corn stover	Positive	long-term potentiation	1.52E-07	103
Duodenum	Corn stover	Positive	necrosis of muscle	1.75E-07	139
Duodenum	Corn stover	Positive	morphology of blood vessel	1.83E-07	117
Duodenum	Corn stover	Positive	learning	1.83E-07	166
Duodenum	Corn stover	Positive	metabolism of nucleic acid component or derivative	1.89E-07	202
Duodenum	Corn stover	Positive	outgrowth of neurites	1.93E-07	161
Duodenum	Corn stover	Positive	cell movement of smooth muscle cells	2.05E-07	72
Duodenum	Corn stover	Positive	abnormal morphology of embryonic tissue	2.12E-07	212
Duodenum	Corn stover	Positive	cell death of fibroblast cell lines	2.18E-07	169
Duodenum	Corn stover	Positive	formation of plasma membrane projections	2.24E-07	229
Duodenum	Corn stover	Positive	synthesis of lipid	2.35E-07	270
Duodenum	Corn stover	Positive	formation of muscle	2.41E-07	158
Duodenum	Corn stover	Positive	growth of neurites	2.85E-07	181
Duodenum	Corn stover	Positive	mass of organism	2.85E-07	135
Duodenum	Corn stover	Positive	development of genitourinary system	2.89E-07	329
Duodenum	Corn stover	Positive	cognition	3.16E-07	178
Duodenum	Corn stover	Positive	accumulation of lipid	3.31E-07	122
Duodenum	Corn stover	Positive	cell death of epithelial cells	3.41E-07	193
Duodenum	Corn	Positive	activation of cells	3.50E-07	359

	stover				
Duodenum	Corn stover	Positive	quantity of mononuclear leukocytes	3.98E-07	276
Duodenum	Corn stover	Positive	cell death of heart cells	4.10E-07	93
Duodenum	Corn stover	Positive	development of mononuclear leukocytes	4.26E-07	214
Duodenum	Corn stover	Positive	quantity of T lymphocytes	4.35E-07	206
Duodenum	Corn stover	Positive	abnormal morphology of abdomen	4.53E-07	324
Duodenum	Corn stover	Positive	apoptosis of muscle	4.58E-07	108
Duodenum	Corn stover	Positive	morphology of respiratory system	4.58E-07	126
Duodenum	Corn stover	Positive	cell death of fibroblasts	4.84E-07	109
Duodenum	Corn stover	Positive	development of lymphocytes	5.38E-07	212
Duodenum	Corn stover	Positive	proliferation of fibroblast cell lines	5.43E-07	166
Duodenum	Corn stover	Positive	abnormal morphology of respiratory system	5.93E-07	124
Duodenum	Corn stover	Positive	migration of smooth muscle cells	6.10E-07	66
Duodenum	Corn stover	Positive	cardiogenesis	6.10E-07	183
Duodenum	Corn stover	Positive	cell movement of muscle cells	6.10E-07	78
Duodenum	Corn stover	Positive	apoptosis of muscle cells	6.15E-07	107
Duodenum	Corn stover	Positive	abnormal morphology of reproductive system	6.16E-07	196
Duodenum	Corn stover	Positive	homing of cells	6.62E-07	213
Duodenum	Corn stover	Positive	morphology of reproductive system	6.83E-07	205
Duodenum	Corn stover	Positive	outgrowth of cells	7.20E-07	169
Duodenum	Corn stover	Positive	infection by HIV-1	7.21E-07	231
Duodenum	Corn stover	Positive	vascularization	7.21E-07	98
Duodenum	Corn	Positive	peripheral vascular disease	7.36E-07	150

	stover				
Duodenum	Corn stover	Positive	development of digestive system	7.46E-07	151
Duodenum	Corn stover	Positive	development of cardiovascular tissue	7.87E-07	146
Duodenum	Corn stover	Positive	dysgenesis	8.49E-07	206
Duodenum	Corn stover	Positive	apoptosis of fibroblast cell lines	8.56E-07	131
Duodenum	Corn stover	Positive	apoptosis of fibroblasts	9.31E-07	91
Duodenum	Corn stover	Positive	formation of actin stress fibers	9.31E-07	99
Duodenum	Corn stover	Positive	differentiation of leukocytes	9.57E-07	252
Duodenum	Corn stover	Positive	abnormal morphology of epithelial tissue	9.99E-07	163
Duodenum	Corn stover	Positive	cell viability of cervical cancer cell lines	9.99E-07	92
Duodenum	Corn stover	Positive	metabolism of nucleotide	1.05E-06	170
Duodenum	Corn stover	Positive	abnormal morphology of blood vessel	1.09E-06	108
Duodenum	Corn stover	Positive	synthesis of carbohydrate	1.16E-06	165
Duodenum	Corn stover	Positive	cell death of sarcoma cell lines	1.16E-06	94
Duodenum	Corn stover	Positive	abnormal morphology of bone	1.18E-06	169
Duodenum	Corn stover	Positive	formation of cells	1.20E-06	357
Duodenum	Corn stover	Positive	proliferation of hematopoietic cells	1.25E-06	104
Duodenum	Corn stover	Positive	function of cardiovascular system	1.36E-06	145
Duodenum	Corn stover	Positive	homing	1.37E-06	217
Duodenum	Corn stover	Positive	cell death of heart	1.40E-06	94
Duodenum	Corn stover	Positive	expression of protein	1.44E-06	134
Duodenum	Corn stover	Positive	apoptosis of heart cells	1.45E-06	80
Duodenum	Corn	Positive	cell death of cervical cancer cell lines	1.58E-06	138

	stover				
Duodenum	Corn stover	Positive	migration of muscle cells	1.58E-06	71
Duodenum	Corn stover	Positive	morphology of muscle cells	1.58E-06	71
Duodenum	Corn stover	Positive	morphology of heart ventricle	1.58E-06	81
Duodenum	Corn stover	Positive	peripheral arterial disease	1.58E-06	88
Duodenum	Corn stover	Positive	endothelial cell development	1.58E-06	139
Duodenum	Corn stover	Positive	development of lymphatic system	1.61E-06	140
Duodenum	Corn stover	Positive	Bleeding	1.64E-06	164
Duodenum	Corn stover	Positive	cell movement of leukocytes	1.65E-06	283
Duodenum	Corn stover	Positive	differentiation of blood cells	1.75E-06	309
Duodenum	Corn stover	Positive	growth of skin	1.83E-06	90
Duodenum	Corn stover	Positive	activation of enzyme	1.90E-06	151
Duodenum	Corn stover	Positive	synthesis of nucleotide	1.91E-06	142
Duodenum	Corn stover	Positive	development of endothelial tissue	1.94E-06	143
Duodenum	Corn stover	Positive	cell movement of myeloid cells	2.28E-06	204
Duodenum	Corn stover	Positive	proliferation of smooth muscle cells	2.30E-06	114
Duodenum	Corn stover	Positive	homeostasis of leukocytes	2.46E-06	209
Duodenum	Corn stover	Positive	synthesis of protein	2.48E-06	171
Duodenum	Corn stover	Positive	morphology of cardiac muscle	2.51E-06	71
Duodenum	Corn stover	Positive	morphology of skin	2.62E-06	109
Duodenum	Corn stover	Positive	secretory pathway	2.62E-06	82
Duodenum	Corn stover	Positive	abnormal morphology of heart ventricle	2.83E-06	74
Duodenum	Corn	Positive	cell death of cardiomyocytes	2.85E-06	88

	stover				
Duodenum	Corn stover	Positive	export of molecule	3.00E-06	117
Duodenum	Corn stover	Positive	Hypoplasia	3.05E-06	191
Duodenum	Corn stover	Positive	Hypertrophy	3.15E-06	180
Duodenum	Corn stover	Positive	advanced malignant tumor	3.25E-06	308
Duodenum	Corn stover	Positive	growth of lymphatic system component	3.32E-06	80
Duodenum	Corn stover	Positive	autophagy	3.48E-06	145
Duodenum	Corn stover	Positive	quantity of lymphatic system component	3.51E-06	146
Duodenum	Corn stover	Positive	sprouting	3.62E-06	149
Duodenum	Corn stover	Positive	proliferation of hematopoietic progenitor cells	3.65E-06	98
Duodenum	Corn stover	Positive	differentiation of embryonic tissue	3.75E-06	102
Duodenum	Corn stover	Positive	cell spreading	3.75E-06	110
Duodenum	Corn stover	Positive	apoptosis of cardiomyocytes	3.79E-06	77
Duodenum	Corn stover	Positive	apoptosis of epithelial cell lines	4.20E-06	91
Duodenum	Corn stover	Positive	abnormal morphology of muscle	4.21E-06	124
Duodenum	Corn stover	Positive	proliferation of epithelial cells	4.34E-06	186
Duodenum	Corn stover	Positive	formation of eye	4.48E-06	167
Duodenum	Corn stover	Positive	cell death of tumor	4.60E-06	147
Duodenum	Corn stover	Positive	Lymphocyte homeostasis	4.80E-06	204
Duodenum	Corn stover	Positive	cell death of cortical neurons	4.87E-06	73
Duodenum	Corn stover	Positive	cell movement of breast cancer cell lines	5.05E-06	114
Duodenum	Corn stover	Positive	cartilage development	5.11E-06	62
Duodenum	Corn	Positive	development of lymphatic system component	5.26E-06	121

	stover				
Duodenum	Corn stover	Positive	T cell development	5.32E-06	189
Duodenum	Corn stover	Positive	interphase	5.50E-06	242
Duodenum	Corn stover	Positive	synthesis of reactive oxygen species	5.51E-06	181
Duodenum	Corn stover	Positive	phosphorylation of protein	5.57E-06	269
Duodenum	Corn stover	Positive	organization of actin cytoskeleton	5.60E-06	118
Duodenum	Corn stover	Positive	necrosis of tumor	5.86E-06	146
Duodenum	Corn stover	Positive	G1 phase	5.99E-06	149
Duodenum	Corn stover	Positive	proliferation of blood cells	6.02E-06	305
Duodenum	Corn stover	Positive	development of sensory organ	6.09E-06	210
Duodenum	Corn stover	Positive	quantity of interleukin	6.18E-06	60
Duodenum	Corn stover	Positive	binding of DNA	6.26E-06	195
Duodenum	Corn stover	Positive	Edema	6.55E-06	133
Duodenum	Corn stover	Positive	quantity of IL-6 in blood	6.57E-06	40
Duodenum	Corn stover	Positive	formation of actin filaments	6.60E-06	119
Duodenum	Corn stover	Positive	abnormal morphology of internal genitalia	6.69E-06	135
Duodenum	Corn stover	Positive	abnormal morphology of left ventricle	6.71E-06	39
Duodenum	Corn stover	Positive	quantity of lymphoid organ	6.71E-06	120
Duodenum	Corn stover	Positive	engulfment of cells	6.86E-06	155
Duodenum	Corn stover	Positive	dephosphorylation of protein	6.86E-06	77
Duodenum	Corn stover	Positive	muscle tumor	6.93E-06	139
Duodenum	Corn stover	Positive	morphology of genital organ	6.93E-06	161
Duodenum	Corn	Positive	migration of breast cancer cell lines	7.00E-06	98

	stover				
Duodenum	Corn stover	Positive	proliferation of embryonic cells	7.33E-06	99
Duodenum	Corn stover	Positive	differentiation of muscle	7.33E-06	118
Duodenum	Corn stover	Positive	metabolism of reactive oxygen species	8.10E-06	187
Duodenum	Corn stover	Positive	cell movement of blood cells	8.43E-06	315
Duodenum	Corn stover	Positive	replication of Influenza virus	8.79E-06	113
Duodenum	Corn stover	Positive	benign neoplasm of female genital organ	8.86E-06	161
Duodenum	Corn stover	Positive	targeting of protein	9.01E-06	56
Duodenum	Corn stover	Positive	cell movement of vascular smooth muscle cells	9.01E-06	50
Duodenum	Corn stover	Positive	production of reactive oxygen species	9.09E-06	140
Duodenum	Corn stover	Positive	morphology of left ventricle	9.12E-06	40
Duodenum	Corn stover	Positive	quantity of thymus gland	9.18E-06	93
Duodenum	Corn stover	Positive	migration of blood cells	9.18E-06	314
Duodenum	Corn stover	Positive	damage of nervous system	9.32E-06	90
Duodenum	Corn stover	Positive	replication of Influenza A virus	9.81E-06	112
Duodenum	Corn stover	Positive	chemotaxis of cells	1.03E-05	196
Duodenum	Corn stover	Positive	homo-oligomerization of protein	1.04E-05	57
Duodenum	Corn stover	Positive	morphology of central nervous system	1.07E-05	191
Duodenum	Corn stover	Positive	quantity of protein in blood	1.09E-05	189
Duodenum	Corn stover	Positive	leukocyte migration	1.10E-05	313
Duodenum	Corn stover	Positive	Oral Cancer and Tumors	1.18E-05	91
Duodenum	Corn stover	Positive	uptake of D-hexose	1.27E-05	89
Duodenum	Corn	Positive	atrophy of muscle	1.28E-05	62

	stover				
Duodenum	Corn stover	Positive	transmembrane potential of mitochondria	1.28E-05	86
Duodenum	Corn stover	Positive	morphology of cardiovascular tissue	1.28E-05	40
Duodenum	Corn stover	Positive	gliosis of brain	1.28E-05	22
Duodenum	Corn stover	Positive	adenocarcinoma	1.28E-05	2109
Duodenum	Corn stover	Positive	development of urinary tract	1.28E-05	111
Duodenum	Corn stover	Positive	memory	1.28E-05	101
Duodenum	Corn stover	Positive	differentiation of epithelial tissue	1.28E-05	137
Duodenum	Corn stover	Positive	arthritis	1.37E-05	334
Duodenum	Corn stover	Positive	release of L-amino acid	1.37E-05	38
Duodenum	Corn stover	Positive	autosomal dominant disease	1.38E-05	233
Duodenum	Corn stover	Positive	contractility of heart	1.49E-05	72
Duodenum	Corn stover	Positive	biosynthesis of nucleoside triphosphate	1.50E-05	47
Duodenum	Corn stover	Positive	cell death of tumor cells	1.50E-05	141
Duodenum	Corn stover	Positive	arthropathy	1.52E-05	339
Duodenum	Corn stover	Positive	migration of carcinoma cell lines	1.52E-05	70
Duodenum	Corn stover	Positive	oral cancer	1.56E-05	90
Duodenum	Corn stover	Positive	cell death of neuroblastoma cell lines	1.59E-05	78
Duodenum	Corn stover	Positive	occlusion of blood vessel	1.59E-05	179
Duodenum	Corn stover	Positive	chemotaxis	1.59E-05	201
Duodenum	Corn stover	Positive	T cell homeostasis	1.59E-05	191
Duodenum	Corn stover	Positive	migration of vascular smooth muscle cells	1.60E-05	46
Duodenum	Corn	Positive	formation of lung	1.63E-05	116

	stover				
Duodenum	Corn stover	Positive	cell viability of central nervous system cells	1.66E-05	49
Duodenum	Corn stover	Positive	dyspnea	1.66E-05	57
Duodenum	Corn stover	Positive	MAPKKK cascade	1.69E-05	82
Duodenum	Corn stover	Positive	quantity of hematopoietic progenitor cells	1.69E-05	165
Duodenum	Corn stover	Positive	apoptosis of neurons	1.69E-05	163
Duodenum	Corn stover	Positive	transmigration of cells	1.73E-05	65
Duodenum	Corn stover	Positive	vaso-occlusion	1.75E-05	178
Duodenum	Corn stover	Positive	proliferation of dermal cells	1.77E-05	72
Duodenum	Corn stover	Positive	differentiation of muscle cells	1.85E-05	109
Duodenum	Corn stover	Positive	cell movement of phagocytes	1.88E-05	202
Duodenum	Corn stover	Positive	morphology of endothelial tissue	1.88E-05	38
Duodenum	Corn stover	Positive	quantity of thymocytes	1.88E-05	91
Duodenum	Corn stover	Positive	morphogenesis of neurons	1.90E-05	159
Duodenum	Corn stover	Positive	epilepsy	1.92E-05	118
Duodenum	Corn stover	Positive	gliosis of central nervous system	1.92E-05	29
Duodenum	Corn stover	Positive	metabolism of hexose	1.92E-05	47
Duodenum	Corn stover	Positive	endocytosis	1.98E-05	131
Duodenum	Corn stover	Positive	cell movement of carcinoma cell lines	1.98E-05	82
Duodenum	Corn stover	Positive	cell death of blood cells	2.00E-05	251
Duodenum	Corn stover	Positive	adenoma	2.02E-05	203
Duodenum	Corn stover	Positive	cell movement of fibroblasts	2.04E-05	74
Duodenum	Corn	Positive	formation of kidney	2.05E-05	108

	stover				
Duodenum	Corn stover	Positive	generation of lymphocytes	2.11E-05	59
Duodenum	Corn stover	Positive	abnormal morphology of genital organ	2.12E-05	153
Duodenum	Corn stover	Positive	occlusion of artery	2.15E-05	176
Duodenum	Corn stover	Positive	homeostasis of neurons	2.18E-05	11
Duodenum	Corn stover	Positive	morphology of nerves	2.35E-05	58
Duodenum	Corn stover	Positive	fatty acid metabolism	2.37E-05	219
Duodenum	Corn stover	Positive	activation of Protein kinase	2.43E-05	98
Duodenum	Corn stover	Positive	cytostasis	2.43E-05	109
Duodenum	Corn stover	Positive	hypertrophy of heart	2.47E-05	128
Duodenum	Corn stover	Positive	uptake of D-glucose	2.56E-05	87
Duodenum	Corn stover	Positive	abnormal morphology of skin	2.56E-05	95
Duodenum	Corn stover	Positive	cell death of central nervous system cells	2.58E-05	112
Duodenum	Corn stover	Positive	fertility	2.62E-05	137
Duodenum	Corn stover	Positive	branching of cells	2.64E-05	139
Duodenum	Corn stover	Positive	muscular hypertrophy	2.65E-05	81
Duodenum	Corn stover	Positive	apoptosis of neuroblastoma cell lines	2.66E-05	52
Duodenum	Corn stover	Positive	movement of vascular endothelial cells	2.76E-05	73
Duodenum	Corn stover	Positive	maturation of cells	2.78E-05	169
Duodenum	Corn stover	Positive	exocytosis	2.81E-05	76
Duodenum	Corn stover	Positive	cell death of pheochromocytoma cell lines	2.94E-05	51
Duodenum	Corn stover	Positive	morphology of cardiomyocytes	2.98E-05	41
Duodenum	Corn	Positive	morphology of skull	3.16E-05	88

	stover				
Duodenum	Corn stover	Positive	apoptosis of cervical cancer cell lines	3.16E-05	108
Duodenum	Corn stover	Positive	metabolism of D-glucose	3.18E-05	40
Duodenum	Corn stover	Positive	quantity of cellular protrusions	3.19E-05	65
Duodenum	Corn stover	Positive	degeneration of central nervous system	3.19E-05	55
Duodenum	Corn stover	Positive	length of cells	3.19E-05	44
Duodenum	Corn stover	Positive	function of muscle	3.22E-05	131
Duodenum	Corn stover	Positive	I-kappaB kinase/NF-kappaB cascade	3.22E-05	70
Duodenum	Corn stover	Positive	adhesion of tumor cell lines	3.24E-05	110
Duodenum	Corn stover	Positive	congenital anomaly of cardiovascular system	3.24E-05	89
Duodenum	Corn stover	Positive	congenital malformation of skeleton	3.25E-05	157
Duodenum	Corn stover	Positive	synthesis of DNA	3.27E-05	155
Duodenum	Corn stover	Positive	female genital tract serous cancer	3.32E-05	144
Duodenum	Corn stover	Positive	abnormal morphology of skull	3.35E-05	86
Duodenum	Corn stover	Positive	generation of T lymphocytes	3.51E-05	49
Duodenum	Corn stover	Positive	hypersensitive reaction	3.61E-05	159
Duodenum	Corn stover	Positive	proliferation of endothelial cells	3.68E-05	118
Duodenum	Corn stover	Positive	binding of protein binding site	3.73E-05	110
Duodenum	Corn stover	Positive	cell death of kidney cell lines	4.07E-05	115
Duodenum	Corn stover	Positive	proliferation of epidermal cells	4.09E-05	68
Duodenum	Corn stover	Positive	formation of skin	4.23E-05	137
Duodenum	Corn stover	Positive	intermediate disease stage peripheral arterial disease	4.39E-05	64
Duodenum	Corn	Positive	formation of lymphatic system component	4.40E-05	111

	stover				
Duodenum	Corn stover	Positive	cell death of cerebral cortex cells	4.52E-05	89
Duodenum	Corn stover	Positive	colony formation of tumor cell lines	4.52E-05	105
Duodenum	Corn stover	Positive	invasion of carcinoma cell lines	4.52E-05	78
Duodenum	Corn stover	Positive	metastasis	4.54E-05	271
Duodenum	Corn stover	Positive	morphology of lymphatic system component	4.61E-05	154
Duodenum	Corn stover	Positive	cellular infiltration by leukocytes	4.75E-05	144
Duodenum	Corn stover	Positive	abdominal adenocarcinoma	4.79E-05	1914
Duodenum	Corn stover	Positive	abnormal morphology of central nervous system	4.79E-05	177
Duodenum	Corn stover	Positive	morphology of digestive system	4.80E-05	207
Duodenum	Corn stover	Positive	oral cavity carcinoma	5.10E-05	77
Duodenum	Corn stover	Positive	cell movement of endothelial cells	5.31E-05	132
Duodenum	Corn stover	Positive	hypoplasia of organ	5.41E-05	164
Duodenum	Corn stover	Positive	Rheumatic Disease	5.42E-05	376
Duodenum	Corn stover	Positive	degeneration of brain	5.52E-05	52
Duodenum	Corn stover	Positive	invasion of prostate cancer cell lines	5.57E-05	44
Duodenum	Corn stover	Positive	connective or soft tissue tumor	5.66E-05	252
Duodenum	Corn stover	Positive	apoptosis of pheochromocytoma cell lines	5.78E-05	40
Duodenum	Corn stover	Positive	growth of brain	5.87E-05	49
Duodenum	Corn stover	Positive	gastrointestinal tract cancer	5.90E-05	1646
Duodenum	Corn stover	Positive	differentiation of embryonic cells	5.95E-05	88
Duodenum	Corn stover	Positive	skin abnormality	5.95E-05	88
Duodenum	Corn	Positive	smooth muscle tumor	5.95E-05	114

	stover				
Duodenum	Corn stover	Positive	cell viability of embryonic cells	5.96E-05	26
Duodenum	Corn stover	Positive	morphology of skeleton	6.05E-05	106
Duodenum	Corn stover	Positive	generation of cells	6.19E-05	100
Duodenum	Corn stover	Positive	transmigration of blood cells	6.21E-05	53
Duodenum	Corn stover	Positive	contractility of ventricular myocardium	6.22E-05	27
Duodenum	Corn stover	Positive	metabolism of nucleoside triphosphate	6.28E-05	57
Duodenum	Corn stover	Positive	midline defect	6.28E-05	101
Duodenum	Corn stover	Positive	morphogenesis of neurites	6.31E-05	154
Duodenum	Corn stover	Positive	bone mineral density	6.36E-05	69
Duodenum	Corn stover	Positive	cell death of brain	6.38E-05	110
Duodenum	Corn stover	Positive	contractility of myocardium	6.41E-05	29
Duodenum	Corn stover	Positive	morphology of endothelial cells	6.41E-05	29
Duodenum	Corn stover	Positive	gastrointestinal carcinoma	6.42E-05	1479
Duodenum	Corn stover	Positive	gliosis of cerebral cortex	6.45E-05	14
Duodenum	Corn stover	Positive	abnormal morphology of uterus	6.45E-05	38
Duodenum	Corn stover	Positive	abnormal morphology of skeleton	6.45E-05	103
Duodenum	Corn stover	Positive	cell death of kidney cells	6.50E-05	130
Duodenum	Corn stover	Positive	quantity of cytokine	6.51E-05	97
Duodenum	Corn stover	Positive	feeding	6.62E-05	114
Duodenum	Corn stover	Positive	function of phagocytes	6.68E-05	115
Duodenum	Corn stover	Positive	morphology of brain	6.75E-05	170
Duodenum	Corn	Positive	cell death of immune cells	6.81E-05	236

	stover				
Duodenum	Corn stover	Positive	relaxation of muscle	6.82E-05	37
Duodenum	Corn stover	Positive	metabolism of D-hexose	6.89E-05	41
Duodenum	Corn stover	Positive	differentiation of bone	7.00E-05	151
Duodenum	Corn stover	Positive	abnormal morphology of body wall	7.02E-05	44
Duodenum	Corn stover	Positive	hypertrophy of cardiac muscle	7.07E-05	63
Duodenum	Corn stover	Positive	development of striated muscle	7.07E-05	71
Duodenum	Corn stover	Positive	development of reproductive system	7.14E-05	262
Duodenum	Corn stover	Positive	quantity of metal ion	7.17E-05	162
Duodenum	Corn stover	Positive	death of perinatal stage organism	7.17E-05	36
Duodenum	Corn stover	Positive	apoptosis of blood cells	7.29E-05	180
Duodenum	Corn stover	Positive	formation of filopodia	7.30E-05	66
Duodenum	Corn stover	Positive	Dermatitis	7.31E-05	159
Duodenum	Corn stover	Positive	concentration of phospholipid	7.46E-05	72
Duodenum	Corn stover	Positive	Gastrointestinal Tract Cancer and Tumors	7.47E-05	1670
Duodenum	Corn stover	Positive	release of L-glutamic acid	7.53E-05	35
Duodenum	Corn stover	Positive	morphology of mouth	7.53E-05	55
Duodenum	Corn stover	Positive	infection of tumor cell lines	8.31E-05	185
Duodenum	Corn stover	Positive	apoptosis of sarcoma cell lines	8.39E-05	74
Duodenum	Corn stover	Positive	cell death of brain cells	8.39E-05	103
Duodenum	Corn stover	Positive	growth of lymphoid organ	8.39E-05	68
Duodenum	Corn stover	Positive	stress response of cells	8.41E-05	56
Duodenum	Corn	Positive	homeostasis of ion	8.54E-05	91

	stover				
Duodenum	Corn stover	Positive	differentiation of epithelial cells	8.66E-05	117
Duodenum	Corn stover	Positive	migration of vascular endothelial cells	8.82E-05	66
Duodenum	Corn stover	Positive	cell viability of brain cells	9.01E-05	41
Duodenum	Corn stover	Positive	release of amino acids	9.01E-05	41
Duodenum	Corn stover	Positive	abnormal morphology of snout	9.05E-05	30
Duodenum	Corn stover	Positive	autophagy of cells	9.05E-05	100
Duodenum	Corn stover	Positive	formation of lymphoid organ	9.05E-05	100
Duodenum	Corn stover	Positive	progression of tumor	9.19E-05	64
Duodenum	Corn stover	Positive	abnormal morphology of digestive system	9.28E-05	196
Duodenum	Corn stover	Positive	vascularization of body region	9.34E-05	55
Duodenum	Corn stover	Positive	ion homeostasis of cells	9.55E-05	200
Duodenum	Corn stover	Positive	differentiation of bone cells	9.60E-05	149
Duodenum	Corn stover	Positive	morphology of uterus	9.72E-05	40
Duodenum	Corn stover	Positive	hypertrophy of tissue	9.79E-05	97
Duodenum	Corn stover	Positive	infection of cervical cancer cell lines	9.79E-05	160
Duodenum	Corn stover	Positive	biosynthesis of purine ribonucleotide	9.80E-05	43
Duodenum	Corn stover	Positive	potentiation of synapse	9.82E-05	60
Duodenum	Corn stover	Positive	morphology of gonad	9.97E-05	139
Duodenum	Corn stover	Positive	G1/S phase transition	9.97E-05	82
Duodenum	Corn stover	Positive	transport of inorganic cation	1.01E-04	130
Duodenum	Corn stover	Positive	formation of leukocytes	1.02E-04	63
Duodenum	Corn	Positive	transmigration of leukocytes	1.03E-04	52

	stover				
Duodenum	Corn stover	Positive	proliferation of immune cells	1.03E-04	279
Duodenum	Corn stover	Positive	neurodegeneration of neurites	1.04E-04	39
Duodenum	Corn stover	Positive	cell death of bone cancer cell lines	1.05E-04	69
Duodenum	Corn stover	Positive	morphology of testis	1.06E-04	102
Duodenum	Corn stover	Positive	generation of leukocytes	1.10E-04	64
Duodenum	Corn stover	Positive	Cytosis	1.10E-04	119
Duodenum	Corn stover	Positive	hepatobiliary system cancer	1.11E-04	1416
Duodenum	Corn stover	Positive	necrosis of kidney	1.12E-04	134
Duodenum	Corn stover	Positive	craniofacial abnormality	1.13E-04	131
Duodenum	Corn stover	Positive	oxidative stress	1.13E-04	33
Duodenum	Corn stover	Positive	leiomyomatosis	1.13E-04	107
Duodenum	Corn stover	Positive	checkpoint control	1.14E-04	44
Duodenum	Corn stover	Positive	hypertrophy of cells	1.14E-04	108
Duodenum	Corn stover	Positive	generation of blood cells	1.17E-04	65
Duodenum	Corn stover	Negative	malignant solid tumor	1.25E-45	4008
Duodenum	Corn stover	Negative	cancer	1.25E-45	4050
Duodenum	Corn stover	Negative	proliferation of cells	9.35E-37	1558
Duodenum	Corn stover	Negative	cell death	3.18E-33	1411
Duodenum	Corn stover	Negative	organismal death	3.24E-31	1052
Duodenum	Corn stover	Negative	morbidity or mortality	5.35E-31	1063
Duodenum	Corn stover	Negative	necrosis	2.07E-28	1105
Duodenum	Corn	Negative	tumorigenesis of tissue	1.42E-27	3365

	stover				
Duodenum	Corn stover	Negative	organization of cytoplasm	1.97E-27	705
Duodenum	Corn stover	Negative	neoplasia of epithelial tissue	6.33E-27	3311
Duodenum	Corn stover	Negative	morphology of cells	9.84E-27	861
Duodenum	Corn stover	Negative	epithelial cancer	2.23E-26	3279
Duodenum	Corn stover	Negative	apoptosis	3.96E-25	1114
Duodenum	Corn stover	Negative	abdominal neoplasm	2.49E-22	3260
Duodenum	Corn stover	Negative	organization of cytoskeleton	4.71E-22	629
Duodenum	Corn stover	Negative	cell movement	2.34E-21	904
Duodenum	Corn stover	Negative	abdominal cancer	1.63E-20	3213
Duodenum	Corn stover	Negative	expression of RNA	2.27E-20	906
Duodenum	Corn stover	Negative	transcription	3.93E-19	843
Duodenum	Corn stover	Negative	cell death of tumor cell lines	4.10E-19	666
Duodenum	Corn stover	Negative	cell survival	5.29E-18	610
Duodenum	Corn stover	Negative	transcription of RNA	6.97E-18	785
Duodenum	Corn stover	Negative	transport of molecule	9.08E-18	692
Duodenum	Corn stover	Negative	digestive organ tumor	1.06E-17	2813
Duodenum	Corn stover	Negative	microtubule dynamics	1.63E-17	527
Duodenum	Corn stover	Negative	digestive system cancer	2.24E-17	2786
Duodenum	Corn stover	Negative	migration of cells	7.58E-17	796
Duodenum	Corn stover	Negative	cell death of connective tissue cells	1.92E-16	290
Duodenum	Corn stover	Negative	vasculogenesis	2.25E-16	345
Duodenum	Corn	Negative	abnormal morphology of cells	2.25E-16	553

	stover				
Duodenum	Corn stover	Negative	development of cytoplasm	2.65E-16	230
Duodenum	Corn stover	Negative	angiogenesis	2.84E-16	413
Duodenum	Corn stover	Negative	cell viability	4.17E-16	565
Duodenum	Corn stover	Negative	differentiation of cells	3.87E-15	914
Duodenum	Corn stover	Negative	morphology of cardiovascular system	4.92E-15	292
Duodenum	Corn stover	Negative	apoptosis of tumor cell lines	1.18E-14	524
Duodenum	Corn stover	Negative	autosomal recessive disease	1.19E-14	412
Duodenum	Corn stover	Negative	Movement Disorders	2.70E-14	464
Duodenum	Corn stover	Negative	cell cycle progression	1.48E-13	456
Duodenum	Corn stover	Negative	invasion of cells	2.40E-13	386
Duodenum	Corn stover	Negative	transcription of DNA	4.39E-13	638
Duodenum	Corn stover	Negative	quantity of cells	1.02E-12	722
Duodenum	Corn stover	Negative	Growth Failure	1.81E-12	274
Duodenum	Corn stover	Negative	formation of cytoskeleton	1.81E-12	182
Duodenum	Corn stover	Negative	growth of organism	2.85E-12	368
Duodenum	Corn stover	Negative	abnormal morphology of thoracic cavity	3.51E-12	268
Duodenum	Corn stover	Negative	growth of connective tissue	4.13E-12	291
Duodenum	Corn stover	Negative	size of body	6.77E-12	372
Duodenum	Corn stover	Negative	proliferation of connective tissue cells	8.79E-12	269
Duodenum	Corn stover	Negative	proliferation of tumor cell lines	2.74E-11	632
Duodenum	Corn stover	Negative	formation of cellular protrusions	3.16E-11	389
Duodenum	Corn	Negative	organization of organelle	3.51E-11	245

	stover				
Duodenum	Corn stover	Negative	morphology of body cavity	4.89E-11	499
Duodenum	Corn stover	Negative	congenital anomaly of musculoskeletal system	4.89E-11	323
Duodenum	Corn stover	Negative	cell movement of tumor cell lines	5.32E-11	368
Duodenum	Corn stover	Negative	cellular homeostasis	5.76E-11	620
Duodenum	Corn stover	Negative	abnormal morphology of cardiovascular system	7.60E-11	255
Duodenum	Corn stover	Negative	morphology of heart	7.60E-11	189
Duodenum	Corn stover	Negative	development of body trunk	9.21E-11	468
Duodenum	Corn stover	Negative	development of vasculature	9.21E-11	194
Duodenum	Corn stover	Negative	proliferation of fibroblast cell lines	9.45E-11	196
Duodenum	Corn stover	Negative	perinatal death	1.10E-10	265
Duodenum	Corn stover	Negative	interphase	1.57E-10	289
Duodenum	Corn stover	Negative	cell death of fibroblasts	1.93E-10	129
Duodenum	Corn stover	Negative	invasion of tumor cell lines	2.14E-10	292
Duodenum	Corn stover	Negative	formation of cells	2.55E-10	415
Duodenum	Corn stover	Negative	Viral Infection	2.58E-10	649
Duodenum	Corn stover	Negative	disorder of basal ganglia	5.56E-10	330
Duodenum	Corn stover	Negative	fibrogenesis	6.75E-10	188
Duodenum	Corn stover	Negative	phosphorylation of protein	7.77E-10	317
Duodenum	Corn stover	Negative	abnormal morphology of body cavity	8.20E-10	472
Duodenum	Corn stover	Negative	mitosis	8.30E-10	222
Duodenum	Corn stover	Negative	dyskinesia	9.12E-10	270
Duodenum	Corn	Negative	epileptic seizure	9.46E-10	86

	stover				
Duodenum	Corn stover	Negative	growth of embryo	9.46E-10	214
Duodenum	Corn stover	Negative	cell transformation	1.20E-09	207
Duodenum	Corn stover	Negative	neurological signs	1.20E-09	283
Duodenum	Corn stover	Negative	necrosis of epithelial tissue	1.37E-09	258
Duodenum	Corn stover	Negative	cell death of cervical cancer cell lines	1.38E-09	162
Duodenum	Corn stover	Negative	benign neoplasia	1.38E-09	389
Duodenum	Corn stover	Negative	formation of filaments	1.54E-09	181
Duodenum	Corn stover	Negative	function of cardiovascular system	2.22E-09	169
Duodenum	Corn stover	Negative	apoptosis of fibroblast cell lines	2.27E-09	152
Duodenum	Corn stover	Negative	cell death of fibroblast cell lines	2.76E-09	192
Duodenum	Corn stover	Negative	concentration of lipid	3.31E-09	343
Duodenum	Corn stover	Negative	ubiquitination	3.39E-09	155
Duodenum	Corn stover	Negative	apoptosis of connective tissue cells	3.79E-09	138
Duodenum	Corn stover	Negative	protein kinase cascade	4.13E-09	189
Duodenum	Corn stover	Negative	migration of tumor cell lines	4.22E-09	302
Duodenum	Corn stover	Negative	chorea	4.22E-09	251
Duodenum	Corn stover	Negative	Huntington's Disease	4.58E-09	250
Duodenum	Corn stover	Negative	colony formation of cells	4.86E-09	217
Duodenum	Corn stover	Negative	formation of actin filaments	5.76E-09	141
Duodenum	Corn stover	Negative	activation of DNA endogenous promoter	6.00E-09	492
Duodenum	Corn stover	Negative	size of embryo	6.27E-09	162
Duodenum	Corn	Negative	cell viability of tumor cell lines	6.27E-09	329

	stover				
Duodenum	Corn stover	Negative	synthesis of lipid	6.42E-09	304
Duodenum	Corn stover	Negative	cell death of epithelial cells	8.19E-09	218
Duodenum	Corn stover	Negative	abnormal morphology of embryonic tissue	9.20E-09	238
Duodenum	Corn stover	Negative	abnormal morphology of heart	1.06E-08	172
Duodenum	Corn stover	Negative	adenocarcinoma	1.67E-08	2381
Duodenum	Corn stover	Negative	quantity of leukocytes	1.76E-08	379
Duodenum	Corn stover	Negative	growth of muscle tissue	1.91E-08	167
Duodenum	Corn stover	Negative	proliferation of muscle cells	2.05E-08	166
Duodenum	Corn stover	Negative	colony formation	2.05E-08	232
Duodenum	Corn stover	Negative	metabolism of carbohydrate	2.24E-08	265
Duodenum	Corn stover	Negative	ubiquitination of protein	2.24E-08	150
Duodenum	Corn stover	Negative	movement of vascular endothelial cells	3.61E-08	88
Duodenum	Corn stover	Negative	apoptosis of cervical cancer cell lines	3.71E-08	129
Duodenum	Corn stover	Negative	development of connective tissue	3.80E-08	165
Duodenum	Corn stover	Negative	degeneration of nervous system	3.95E-08	131
Duodenum	Corn stover	Negative	migration of muscle cells	4.06E-08	81
Duodenum	Corn stover	Negative	congenital malformation of skeleton	5.80E-08	185
Duodenum	Corn stover	Negative	abnormal morphology of nervous system	6.31E-08	303
Duodenum	Corn stover	Negative	size of animal	6.65E-08	86
Duodenum	Corn stover	Negative	neuronal cell death	7.00E-08	286
Duodenum	Corn stover	Negative	cell movement of endothelial cells	7.04E-08	157
Duodenum	Corn	Negative	formation of actin stress fibers	7.33E-08	111

	stover				
Duodenum	Corn stover	Negative	morphology of nervous system	7.73E-08	325
Duodenum	Corn stover	Negative	formation of skin	7.96E-08	162
Duodenum	Corn stover	Negative	proliferation of smooth muscle cells	1.03E-07	129
Duodenum	Corn stover	Negative	migration of smooth muscle cells	1.10E-07	73
Duodenum	Corn stover	Negative	craniofacial abnormality	1.19E-07	157
Duodenum	Corn stover	Negative	morphology of respiratory system	1.33E-07	139
Duodenum	Corn stover	Negative	seizures	1.39E-07	174
Duodenum	Corn stover	Negative	cell movement of fibroblasts	1.44E-07	87
Duodenum	Corn stover	Negative	cell movement of muscle cells	1.49E-07	86
Duodenum	Corn stover	Negative	abnormal morphology of respiratory system	1.50E-07	137
Duodenum	Corn stover	Negative	quantity of blood cells	1.50E-07	415
Duodenum	Corn stover	Negative	transactivation	1.65E-07	244
Duodenum	Corn stover	Negative	cell death of embryonic cells	1.66E-07	59
Duodenum	Corn stover	Negative	abdominal adenocarcinoma	1.68E-07	2161
Duodenum	Corn stover	Negative	growth of epithelial tissue	1.97E-07	283
Duodenum	Corn stover	Negative	genital tumor	2.25E-07	1502
Duodenum	Corn stover	Negative	apoptosis of epithelial cell lines	2.25E-07	103
Duodenum	Corn stover	Negative	seizure disorder	2.25E-07	201
Duodenum	Corn stover	Negative	morphology of head	2.47E-07	357
Duodenum	Corn stover	Negative	Neurodegeneration	2.59E-07	137
Duodenum	Corn stover	Negative	breast or colorectal cancer	3.07E-07	1828
Duodenum	Corn	Negative	neuromuscular disease	3.13E-07	359

	stover				
Duodenum	Corn stover	Negative	abnormal morphology of head	3.13E-07	340
Duodenum	Corn stover	Negative	hepatobiliary system cancer	3.17E-07	1607
Duodenum	Corn stover	Negative	cell death of epithelial cell lines	3.23E-07	126
Duodenum	Corn stover	Negative	cell movement of smooth muscle cells	3.23E-07	77
Duodenum	Corn stover	Negative	metabolism of DNA	3.40E-07	172
Duodenum	Corn stover	Negative	neonatal death	3.59E-07	188
Duodenum	Corn stover	Negative	Organ Degeneration	4.00E-07	209
Duodenum	Corn stover	Negative	female genital neoplasm	4.54E-07	1326
Duodenum	Corn stover	Negative	differentiation of epithelial tissue	4.70E-07	156
Duodenum	Corn stover	Negative	development of cardiovascular tissue	4.70E-07	160
Duodenum	Corn stover	Negative	liver cancer	4.70E-07	1598
Duodenum	Corn stover	Negative	liver tumor	4.88E-07	1608
Duodenum	Corn stover	Negative	hepatocellular carcinoma	5.22E-07	1549
Duodenum	Corn stover	Negative	metabolism of protein	5.75E-07	391
Duodenum	Corn stover	Negative	development of head	5.99E-07	394
Duodenum	Corn stover	Negative	survival of organism	6.22E-07	289
Duodenum	Corn stover	Negative	development of body axis	6.41E-07	420
Duodenum	Corn stover	Negative	synthesis of DNA	6.93E-07	178
Duodenum	Corn stover	Negative	transactivation of RNA	8.29E-07	227
Duodenum	Corn stover	Negative	cell death of stem cells	8.59E-07	44
Duodenum	Corn stover	Negative	differentiation of connective tissue cells	9.20E-07	265
Duodenum	Corn	Negative	cell death of pheochromocytoma cell lines	9.21E-07	59

	stover				
Duodenum	Corn stover	Negative	migration of vascular endothelial cells	9.21E-07	78
Duodenum	Corn stover	Negative	cellular degradation	9.63E-07	113
Duodenum	Corn stover	Negative	differentiation of connective tissue	9.69E-07	298
Duodenum	Corn stover	Negative	development of leukocytes	9.80E-07	244
Duodenum	Corn stover	Negative	proliferation of fibroblasts	1.02E-06	152
Duodenum	Corn stover	Negative	differentiation of tumor cell lines	1.19E-06	162
Duodenum	Corn stover	Negative	development of blood cells	1.24E-06	268
Duodenum	Corn stover	Negative	apoptosis of fibroblasts	1.39E-06	98
Duodenum	Corn stover	Negative	necrosis of kidney	1.41E-06	156
Duodenum	Corn stover	Negative	invasion of tissue	1.49E-06	89
Duodenum	Corn stover	Negative	S phase	1.50E-06	115
Duodenum	Corn stover	Negative	apoptosis of kidney cell lines	1.54E-06	106
Duodenum	Corn stover	Negative	cell spreading	1.70E-06	121
Duodenum	Corn stover	Negative	Bleeding	1.72E-06	179
Duodenum	Corn stover	Negative	quantity of lymphocytes	1.72E-06	288
Duodenum	Corn stover	Negative	morphology of bone	1.73E-06	189
Duodenum	Corn stover	Negative	uptake of monosaccharide	1.73E-06	118
Duodenum	Corn stover	Negative	concentration of fatty acid	1.87E-06	122
Duodenum	Corn stover	Negative	epilepsy	1.96E-06	133
Duodenum	Corn stover	Negative	Gastrointestinal Tract Cancer and Tumors	2.03E-06	1878
Duodenum	Corn stover	Negative	migration of endothelial cells	2.04E-06	141
Duodenum	Corn	Negative	gastrointestinal tract cancer	2.06E-06	1849

	stover				
Duodenum	Corn stover	Negative	behavior	2.12E-06	393
Duodenum	Corn stover	Negative	quantity of mononuclear leukocytes	2.13E-06	298
Duodenum	Corn stover	Negative	cardiogenesis	2.24E-06	197
Duodenum	Corn stover	Negative	differentiation of epithelial cells	2.31E-06	135
Duodenum	Corn stover	Negative	uptake of carbohydrate	2.31E-06	126
Duodenum	Corn stover	Negative	cell death of kidney cells	2.36E-06	149
Duodenum	Corn stover	Negative	abnormal morphology of bone	2.39E-06	183
Duodenum	Corn stover	Negative	formation of focal adhesions	2.39E-06	72
Duodenum	Corn stover	Negative	abnormal morphology of epithelial tissue	2.41E-06	176
Duodenum	Corn stover	Negative	morphology of digestive system	2.43E-06	235
Duodenum	Corn stover	Negative	gastrointestinal carcinoma	2.44E-06	1662
Duodenum	Corn stover	Negative	breast or ovarian cancer	2.59E-06	729
Duodenum	Corn stover	Negative	mitosis of cervical cancer cell lines	2.59E-06	49
Duodenum	Corn stover	Negative	migration of fibroblasts	2.73E-06	67
Duodenum	Corn stover	Negative	Hypertrophy	2.81E-06	197
Duodenum	Corn stover	Negative	cell viability of connective tissue cells	2.95E-06	63
Duodenum	Corn stover	Negative	mitosis of tumor cell lines	2.95E-06	63
Duodenum	Corn stover	Negative	synthesis of carbohydrate	3.03E-06	178
Duodenum	Corn stover	Negative	development of endothelial tissue	3.03E-06	155
Duodenum	Corn stover	Negative	cell death of neuroblastoma cell lines	3.04E-06	87
Duodenum	Corn stover	Negative	neuritogenesis	3.04E-06	239
Duodenum	Corn	Negative	formation of muscle	3.17E-06	167

	stover				
Duodenum	Corn stover	Negative	proliferation of immune cells	3.35E-06	318
Duodenum	Corn stover	Negative	degeneration of neurons	3.36E-06	105
Duodenum	Corn stover	Negative	transport of D-glucose	3.52E-06	67
Duodenum	Corn stover	Negative	Lymphocyte homeostasis	3.57E-06	224
Duodenum	Corn stover	Negative	transport of carbohydrate	3.76E-06	87
Duodenum	Corn stover	Negative	development of epithelial tissue	3.76E-06	213
Duodenum	Corn stover	Negative	cell death of kidney cell lines	3.78E-06	130
Duodenum	Corn stover	Negative	migration of vascular smooth muscle cells	3.83E-06	51
Duodenum	Corn stover	Negative	development of lymphocytes	3.93E-06	227
Duodenum	Corn stover	Negative	growth of tumor	4.03E-06	319
Duodenum	Corn stover	Negative	female genital tract serous cancer	4.03E-06	162
Duodenum	Corn stover	Negative	abnormal morphology of abdomen	4.09E-06	349
Duodenum	Corn stover	Negative	cell movement of connective tissue cells	4.35E-06	98
Duodenum	Corn stover	Negative	morphology of connective tissue	4.36E-06	191
Duodenum	Corn stover	Negative	proliferation of blood cells	4.39E-06	336
Duodenum	Corn stover	Negative	abnormal morphology of digestive system	4.78E-06	223
Duodenum	Corn stover	Negative	homeostasis of leukocytes	4.89E-06	227
Duodenum	Corn stover	Negative	T cell homeostasis	5.06E-06	212
Duodenum	Corn stover	Negative	proliferation of neuronal cells	5.06E-06	235
Duodenum	Corn stover	Negative	cell death of muscle cells	5.20E-06	142
Duodenum	Corn stover	Negative	cell death of embryonic stem cells	5.34E-06	28
Duodenum	Corn	Negative	invasion of carcinoma cell lines	5.34E-06	88

	stover				
Duodenum	Corn stover	Negative	abdominal carcinoma	5.36E-06	2171
Duodenum	Corn stover	Negative	endothelial cell development	5.39E-06	149
Duodenum	Corn stover	Negative	replication of RNA virus	5.40E-06	208
Duodenum	Corn stover	Negative	infection by Retroviridae	5.74E-06	289
Duodenum	Corn stover	Negative	development of lymphatic system	5.74E-06	150
Duodenum	Corn stover	Negative	formation of lymphoid organ	6.19E-06	114
Duodenum	Corn stover	Negative	migration of connective tissue cells	6.35E-06	81
Duodenum	Corn stover	Negative	cell death of muscle	6.35E-06	145
Duodenum	Corn stover	Negative	growth of embryonic tissue	6.46E-06	118
Duodenum	Corn stover	Negative	HIV infection	6.50E-06	286
Duodenum	Corn stover	Negative	activation of cells	6.61E-06	385
Duodenum	Corn stover	Negative	death of perinatal stage organism	6.79E-06	41
Duodenum	Corn stover	Negative	activation of enzyme	6.80E-06	162
Duodenum	Corn stover	Negative	infection by HIV-1	7.00E-06	247
Duodenum	Corn stover	Negative	T cell development	7.02E-06	206
Duodenum	Corn stover	Negative	cell movement of vascular smooth muscle cells	7.27E-06	54
Duodenum	Corn stover	Negative	development of lymphatic system component	7.46E-06	131
Duodenum	Corn stover	Negative	necrosis of muscle	7.86E-06	144
Duodenum	Corn stover	Negative	morphology of vessel	8.08E-06	130
Duodenum	Corn stover	Negative	transmembrane potential of mitochondria	8.11E-06	94
Duodenum	Corn stover	Negative	autosomal dominant disease	8.11E-06	257
Duodenum	Corn	Negative	learning	8.14E-06	173

	stover				
Duodenum	Corn stover	Negative	cognition	8.36E-06	187
Duodenum	Corn stover	Negative	apoptosis of pheochromocytoma cell lines	8.46E-06	45
Duodenum	Corn stover	Negative	formation of lymphatic system component	9.26E-06	124
Duodenum	Corn stover	Negative	apoptosis of stem cells	9.37E-06	36
Duodenum	Corn stover	Negative	apoptosis of muscle	9.94E-06	112
Duodenum	Corn stover	Negative	development of neurons	1.00E-05	311
Duodenum	Corn stover	Negative	Edema	1.05E-05	144
Duodenum	Corn stover	Negative	function of blood cells	1.10E-05	237
Duodenum	Corn stover	Negative	multiple congenital anomalies	1.11E-05	174
Duodenum	Corn stover	Negative	development of digestive system	1.12E-05	159
Duodenum	Corn stover	Negative	morphology of mouth	1.12E-05	62
Duodenum	Corn stover	Negative	morphogenesis of neurons	1.16E-05	175
Duodenum	Corn stover	Negative	tumorigenesis of genital organ	1.17E-05	1435
Duodenum	Corn stover	Negative	cell movement of blood cells	1.23E-05	345
Duodenum	Corn stover	Negative	apoptosis of muscle cells	1.26E-05	111
Duodenum	Corn stover	Negative	apoptosis of neuroblastoma cell lines	1.26E-05	57
Duodenum	Corn stover	Negative	apoptosis of embryonic cells	1.27E-05	47
Duodenum	Corn stover	Negative	migration of blood cells	1.31E-05	344
Duodenum	Corn stover	Negative	formation of plasma membrane projections	1.46E-05	240
Duodenum	Corn stover	Negative	leukocyte migration	1.55E-05	343
Duodenum	Corn stover	Negative	transport of monosaccharide	1.67E-05	71
Duodenum	Corn	Negative	cell death of lymphocytes	1.69E-05	159

	stover				
Duodenum	Corn stover	Negative	proliferation of epidermal cells	1.70E-05	75
Duodenum	Corn stover	Negative	quantity of connective tissue	1.71E-05	242
Duodenum	Corn stover	Negative	proliferation of dermal cells	1.78E-05	78
Duodenum	Corn stover	Negative	quantity of reactive oxygen species	1.78E-05	78
Duodenum	Corn stover	Negative	function of leukocytes	1.78E-05	217
Duodenum	Corn stover	Negative	mass of organism	1.78E-05	139
Duodenum	Corn stover	Negative	cell death of blood cells	1.78E-05	276
Duodenum	Corn stover	Negative	infection of cells	1.78E-05	315
Duodenum	Corn stover	Negative	ruffling	1.80E-05	51
Duodenum	Corn stover	Negative	morphology of skin	1.81E-05	115
Duodenum	Corn stover	Negative	differentiation of epidermal cells	1.85E-05	73
Duodenum	Corn stover	Negative	sprouting	1.88E-05	159
Duodenum	Corn stover	Negative	Hypertension	1.90E-05	256
Duodenum	Corn stover	Negative	cell viability of cervical cancer cell lines	1.93E-05	95
Duodenum	Corn stover	Negative	cell death of immune cells	2.00E-05	263
Duodenum	Corn stover	Negative	cell death of mononuclear leukocytes	2.01E-05	163
Duodenum	Corn stover	Negative	morphology of limb	2.10E-05	99
Duodenum	Corn stover	Negative	serous neoplasm	2.14E-05	244
Duodenum	Corn stover	Negative	transport of alpha-amino acid	2.14E-05	36
Duodenum	Corn stover	Negative	cell death of tumor	2.18E-05	157
Duodenum	Corn stover	Negative	abnormal morphology of limb	2.25E-05	98
Duodenum	Corn	Negative	synthesis of fatty acid	2.28E-05	130

	stover				
Duodenum	Corn stover	Negative	genital tract cancer	2.32E-05	1419
Duodenum	Corn stover	Negative	transformation of fibroblast cell lines	2.38E-05	109
Duodenum	Corn stover	Negative	invasion of lung cancer cell lines	2.41E-05	49
Duodenum	Corn stover	Negative	formation of thymus gland	2.41E-05	72
Duodenum	Corn stover	Negative	arrest in interphase	2.44E-05	170
Duodenum	Corn stover	Negative	leukopenia	2.57E-05	60
Duodenum	Corn stover	Negative	growth of plasma membrane projections	2.58E-05	189
Duodenum	Corn stover	Negative	proliferation of epithelial cells	2.60E-05	199
Duodenum	Corn stover	Negative	differentiation of muscle	2.66E-05	126
Duodenum	Corn stover	Negative	tumorigenesis of reproductive tract	2.75E-05	1254
Duodenum	Corn stover	Negative	degeneration of cells	2.76E-05	142
Duodenum	Corn stover	Negative	sensitivity of cells	2.81E-05	64
Duodenum	Corn stover	Negative	cell death of heart	2.86E-05	97
Duodenum	Corn stover	Negative	small GTPase mediated signal transduction	2.86E-05	80
Duodenum	Corn stover	Negative	outgrowth of plasma membrane projections	2.92E-05	166
Duodenum	Corn stover	Negative	secretion of molecule	2.92E-05	221
Duodenum	Corn stover	Negative	cell death of heart cells	2.93E-05	94
Duodenum	Corn stover	Negative	I-kappaB kinase/NF-kappaB cascade	2.94E-05	76
Duodenum	Corn stover	Negative	hypertrophy of tissue	2.96E-05	108
Duodenum	Corn stover	Negative	stress response of cells	3.01E-05	62
Duodenum	Corn stover	Negative	cytopenia	3.05E-05	124
Duodenum	Corn	Negative	concentration of phosphatidic acid	3.13E-05	61

	stover				
Duodenum	Corn stover	Negative	proliferation of mononuclear leukocytes	3.15E-05	291
Duodenum	Corn stover	Negative	activation of Protein kinase	3.46E-05	106
Duodenum	Corn stover	Negative	morphogenesis of neurites	3.46E-05	170
Duodenum	Corn stover	Negative	cell movement of fibroblast cell lines	3.48E-05	73
Duodenum	Corn stover	Negative	growth of lymphatic system component	3.48E-05	83
Duodenum	Corn stover	Negative	binding of DNA	3.48E-05	209
Duodenum	Corn stover	Negative	accumulation of lipid	3.53E-05	124
Duodenum	Corn stover	Negative	differentiation of leukocytes	3.56E-05	266
Duodenum	Corn stover	Negative	mammary tumor	3.63E-05	619
Duodenum	Corn stover	Negative	proliferation of vascular smooth muscle cells	3.63E-05	68
Duodenum	Corn stover	Negative	growth of lymphoid organ	3.81E-05	75
Duodenum	Corn stover	Negative	replication of virus	3.81E-05	224
Duodenum	Corn stover	Negative	female genital tract cancer	4.04E-05	1244
Duodenum	Corn stover	Negative	necrosis of tumor	4.15E-05	155
Duodenum	Corn stover	Negative	transport of amino acids	4.15E-05	60
Duodenum	Corn stover	Negative	paraproteinemia	4.24E-05	138
Duodenum	Corn stover	Negative	DNA replication	4.34E-05	105
Duodenum	Corn stover	Negative	cartilage development	4.44E-05	64
Duodenum	Corn stover	Negative	abnormal morphology of skull	4.44E-05	93
Duodenum	Corn stover	Negative	growth of neurites	4.44E-05	186
Duodenum	Corn stover	Negative	incidence of tumor	4.45E-05	132
Duodenum	Corn	Negative	muscular hypertrophy	4.69E-05	87

	stover				
Duodenum	Corn stover	Negative	outgrowth of cells	4.69E-05	175
Duodenum	Corn stover	Negative	quantity of carbohydrate	4.99E-05	207
Duodenum	Corn stover	Negative	dysgenesis	5.03E-05	215
Duodenum	Corn stover	Negative	morphology of blood vessel	5.22E-05	117
Duodenum	Corn stover	Negative	dysmyelination	5.30E-05	60
Duodenum	Corn stover	Negative	phosphorylation of L-amino acid	5.30E-05	93
Duodenum	Corn stover	Negative	apoptosis of epithelial cells	5.62E-05	118
Duodenum	Corn stover	Negative	quantity of thymocytes	5.62E-05	97
Duodenum	Corn stover	Negative	colon cancer	5.63E-05	1350
Duodenum	Corn stover	Negative	cell death of tumor cells	5.64E-05	151
Duodenum	Corn stover	Negative	proliferation of lymphocytes	5.69E-05	285
Duodenum	Corn stover	Negative	vascularization	5.69E-05	99
Duodenum	Corn stover	Negative	bone mineral density	5.72E-05	75
Duodenum	Corn stover	Negative	metabolism of membrane lipid derivative	5.76E-05	157
Duodenum	Corn stover	Negative	apoptosis of heart cells	5.76E-05	81
Duodenum	Corn stover	Negative	quantity of T lymphocytes	5.76E-05	213
Duodenum	Corn stover	Negative	uptake of D-hexose	5.78E-05	94
Duodenum	Corn stover	Negative	plasma cell dyscrasia	5.89E-05	137
Duodenum	Corn stover	Negative	differentiation of blood cells	6.13E-05	328
Duodenum	Corn stover	Negative	uptake of D-glucose	6.22E-05	93
Duodenum	Corn stover	Negative	differentiation of keratinocytes	6.23E-05	66
Duodenum	Corn	Negative	endometrium tumor	6.29E-05	1061

	stover				
Duodenum	Corn stover	Negative	colon tumor	6.34E-05	1357
Duodenum	Corn stover	Negative	cell death of breast cancer cell lines	6.34E-05	136
Duodenum	Corn stover	Negative	Thrombosis	6.37E-05	73
Duodenum	Corn stover	Negative	midline defect	6.38E-05	110
Duodenum	Corn stover	Negative	oxidative stress response of cells	6.38E-05	31
Duodenum	Corn stover	Negative	outgrowth of neurites	6.47E-05	163
Duodenum	Corn stover	Negative	quantity of hematopoietic progenitor cells	6.97E-05	177
Duodenum	Corn stover	Negative	quantity of lymphoid organ	6.97E-05	126
Duodenum	Corn stover	Negative	fatty acid metabolism	7.36E-05	237
Duodenum	Corn stover	Negative	neoplasia of colon	7.40E-05	1352
Duodenum	Corn stover	Negative	uterine tumor	7.47E-05	1168
Duodenum	Corn stover	Negative	outgrowth of neurons	7.59E-05	164
Duodenum	Corn stover	Negative	formation of lamellipodia	7.59E-05	70
Duodenum	Corn stover	Negative	formation of reactive oxygen species	7.59E-05	41
Duodenum	Corn stover	Negative	morphology of cardiovascular tissue	7.59E-05	41
Duodenum	Corn stover	Negative	cell death of cardiomyocytes	7.59E-05	90
Duodenum	Corn stover	Negative	colony formation of fibroblast cell lines	7.69E-05	43
Duodenum	Corn stover	Negative	cell death of myeloma cell lines	7.69E-05	44
Duodenum	Corn stover	Negative	experimentally-induced diabetes	7.69E-05	44
Duodenum	Corn stover	Negative	metabolism of nucleic acid component or derivative	7.80E-05	206
Duodenum	Corn stover	Negative	morphology of skull	7.97E-05	94
Duodenum	Corn	Negative	interphase of tumor cell lines	8.19E-05	149

	stover				
Duodenum	Corn stover	Negative	recruitment of antigen presenting cells	8.28E-05	54
Duodenum	Corn stover	Negative	concentration of phospholipid	8.31E-05	78
Duodenum	Corn stover	Negative	gastrointestinal adenocarcinoma	8.33E-05	1548
Duodenum	Corn stover	Negative	development of genitourinary system	8.51E-05	343
Duodenum	Corn stover	Negative	migration of fibroblast cell lines	8.61E-05	59
Duodenum	Corn stover	Negative	tumorigenesis of intestine	8.62E-05	1354
Duodenum	Corn stover	Negative	skin abnormality	8.71E-05	95
Duodenum	Corn stover	Negative	urogenital cancer	8.73E-05	1613
Duodenum	Corn stover	Negative	proliferation of embryonic cells	9.17E-05	103
Duodenum	Corn stover	Negative	branching of cells	9.20E-05	149
Duodenum	Corn stover	Negative	morphology of muscle	9.20E-05	136
Duodenum	Corn stover	Negative	infection by RNA virus	9.23E-05	335
Duodenum	Corn stover	Negative	recruitment of cells	9.31E-05	144
Duodenum	Corn stover	Negative	cell movement of leukocytes	9.65E-05	298
Duodenum	Corn stover	Negative	peripheral vascular disease	9.65E-05	153
Duodenum	Corn stover	Negative	recruitment of macrophages	9.70E-05	48
Duodenum	Corn stover	Negative	homing of cells	9.71E-05	220
Duodenum	Corn stover	Negative	growth of skin	9.74E-05	91
Duodenum	Corn stover	Negative	necrosis of cardiac muscle	9.74E-05	91
Duodenum	Corn stover	Negative	development of gastrointestinal tract	9.76E-05	108
Duodenum	Corn stover	Negative	metabolism of nucleotide	9.76E-05	175
Duodenum	Corn	Negative	Hypoplasia	9.76E-05	200

	stover				
Duodenum	Corn stover	Negative	apoptosis of endothelial cells	9.81E-05	78
Duodenum	Corn stover	Negative	endometrial cancer	9.86E-05	1056
Duodenum	Corn stover	Negative	advanced malignant tumor	9.87E-05	327
Duodenum	Corn stover	Negative	arrest in mitosis	1.01E-04	42
Duodenum	Corn stover	Negative	cell movement of cancer cells	1.03E-04	64
Duodenum	Corn stover	Negative	cell movement of epithelial cells	1.03E-04	64
Duodenum	Corn stover	Negative	formation of filopodia	1.04E-04	71
Duodenum	Corn stover	Negative	G1 phase	1.07E-04	156
Duodenum	Corn stover	Negative	metastasis	1.07E-04	295
Duodenum	Corn stover	Negative	hypertrophy of cells	1.08E-04	118
Duodenum	Corn stover	Negative	morphology of heart ventricle	1.16E-04	81
Duodenum	Corn stover	Negative	differentiation of muscle cells	1.18E-04	115
Duodenum	Corn stover	Negative	apoptosis of cardiomyocytes	1.18E-04	78
Duodenum	Corn stover	Negative	colorectal neoplasia	1.22E-04	1572
Duodenum	Corn stover	Negative	formation of leukocytes	1.26E-04	68
Duodenum	Corn stover	Negative	proliferation of endothelial cells	1.34E-04	126
Duodenum	Corn stover	Negative	hypertrophy of heart	1.34E-04	136
Duodenum	Corn stover	Negative	attachment of cells	1.35E-04	59
Duodenum	Corn stover	Negative	connective or soft tissue tumor	1.39E-04	274
Duodenum	Corn stover	Negative	metastatic colorectal cancer	1.40E-04	81
Duodenum	Corn stover	Negative	quantity of lymphatic system component	1.42E-04	151
Duodenum	Corn	Negative	shape change of tumor cell lines	1.42E-04	66

	stover				
Duodenum	Corn stover	Negative	apoptosis of neurons	1.42E-04	173
Duodenum	Corn stover	Negative	cell death of astrocytes	1.42E-04	22
Duodenum	Corn stover	Negative	cell death of hepatoma cell lines	1.46E-04	72
Duodenum	Corn stover	Negative	formation of eye	1.46E-04	174
Duodenum	Corn stover	Negative	apoptosis of hepatoma cell lines	1.50E-04	65
Duodenum	Corn stover	Negative	tumorigenesis of malignant tumor	1.51E-04	110
Duodenum	Corn stover	Negative	generation of lymphocytes	1.51E-04	61
Duodenum	Corn stover	Negative	binding of protein binding site	1.55E-04	117
Duodenum	Corn stover	Negative	development of sensory organ	1.57E-04	221
Duodenum	Corn stover	Negative	polymerization of protein	1.58E-04	148
Duodenum	Corn stover	Negative	arrest in mitosis of tumor cell lines	1.59E-04	33
Duodenum	Corn stover	Negative	cell death of T lymphocytes	1.59E-04	127
Duodenum	Corn stover	Negative	differentiation of skin	1.63E-04	76
Duodenum	Corn stover	Negative	morphology of reproductive system	1.65E-04	210
Duodenum	Corn stover	Negative	expression of protein	1.71E-04	136
Duodenum	Corn stover	Negative	apoptosis of blood cells	1.73E-04	195
Duodenum	Corn stover	Negative	differentiation of bone	1.75E-04	163
Duodenum	Corn stover	Negative	differentiation of T lymphocytes	1.78E-04	151
Duodenum	Corn stover	Negative	intestinal tumor	1.82E-04	1574
Duodenum	Corn stover	Negative	development of reproductive system	1.83E-04	285
Duodenum	Corn stover	Negative	cell movement of neutrophils	1.84E-04	120
Duodenum	Corn	Negative	secretory pathway	1.84E-04	82

	stover				
Duodenum	Corn stover	Negative	cycling of centrosome	1.86E-04	40
Duodenum	Corn stover	Negative	contractility of myocardium	1.90E-04	30
Duodenum	Corn stover	Negative	morphology of endothelial cells	1.90E-04	30
Duodenum	Corn stover	Negative	function of heart	1.90E-04	95
Duodenum	Corn stover	Negative	uterine serous papillary cancer	1.90E-04	95
Duodenum	Corn stover	Negative	homing	1.98E-04	224
Duodenum	Corn stover	Negative	formation of embryonic tissue	1.98E-04	119
Duodenum	Corn stover	Negative	abnormal morphology of skeleton	1.99E-04	110
Duodenum	Corn stover	Negative	female genital tract adenocarcinoma	2.01E-04	1052
Duodenum	Corn stover	Negative	adenoma	2.04E-04	216
Duodenum	Corn stover	Negative	cell death of cerebral cortex cells	2.04E-04	94
Duodenum	Corn stover	Negative	pelvic tumor	2.05E-04	1575
Duodenum	Corn stover	Negative	pelvic cancer	2.08E-04	1565
Duodenum	Corn stover	Negative	morphology of skeleton	2.08E-04	113
Duodenum	Corn stover	Negative	cell movement of tumor cells	2.18E-04	77
Duodenum	Corn stover	Negative	cell death of sarcoma cell lines	2.20E-04	93
Duodenum	Corn stover	Negative	organization of actin cytoskeleton	2.20E-04	121
Duodenum	Corn stover	Negative	colorectal carcinoma	2.22E-04	1342
Duodenum	Corn stover	Negative	abnormal morphology of body wall	2.24E-04	46
Duodenum	Corn stover	Negative	differentiation of bone cells	2.25E-04	161
Duodenum	Corn stover	Negative	activation of neuroglia	2.30E-04	57
Duodenum	Corn	Negative	central nervous system tumor	2.32E-04	349

	stover				
Duodenum	Corn stover	Negative	migration of breast cancer cell lines	2.39E-04	100
Duodenum	Corn stover	Negative	morphology of lung	2.43E-04	78
Duodenum	Corn stover	Negative	targeting of protein	2.43E-04	56
Duodenum	Corn stover	Negative	shape change of fibroblast cell lines	2.47E-04	41
Duodenum	Corn stover	Negative	peripheral arterial disease	2.49E-04	87
Duodenum	Corn stover	Negative	accumulation of reactive oxygen species	2.49E-04	38
Duodenum	Corn stover	Negative	morphology of endothelial tissue	2.49E-04	38
Duodenum	Corn stover	Negative	abnormal morphology of reproductive system	2.49E-04	199
Duodenum	Corn stover	Negative	transport of protein	2.49E-04	121
Duodenum	Corn stover	Negative	formation of vascular lesion	2.50E-04	50
Duodenum	Corn stover	Negative	development of connective tissue cells	2.53E-04	91
Duodenum	Corn stover	Negative	abnormal quantity of amino acids	2.55E-04	29
Duodenum	Corn stover	Negative	cell death of brain	2.61E-04	117
Duodenum	Corn stover	Negative	hydrolysis of nucleotide	2.66E-04	54
Duodenum	Corn stover	Negative	Ovarian Cancer and Tumors	2.80E-04	291
Duodenum	Corn stover	Negative	apoptosis of cerebral cortex cells	2.81E-04	47
Duodenum	Corn stover	Negative	endocytosis	2.86E-04	137
Duodenum	Corn stover	Negative	abnormal morphology of blood vessel	2.86E-04	107
Duodenum	Corn stover	Negative	size of cells	2.87E-04	169
Duodenum	Corn stover	Negative	melanoma	2.88E-04	2368
Duodenum	Corn stover	Negative	arrest in growth of organism	2.89E-04	70
Duodenum	Corn	Negative	demyelination	2.92E-04	52

	stover				
Duodenum	Corn stover	Negative	Breast Cancer and Tumors	2.93E-04	571
Duodenum	Corn stover	Negative	migration of cancer cells	2.94E-04	80
Duodenum	Corn stover	Negative	function of muscle	2.95E-04	138
Duodenum	Corn stover	Negative	colorectal cancer	2.96E-04	1545
Duodenum	Corn stover	Negative	synthesis of protein	3.03E-04	175
Duodenum	Corn stover	Negative	tubulation of endothelial cells	3.03E-04	51
Duodenum	Corn stover	Negative	permeability of cells	3.03E-04	44
Duodenum	Corn stover	Negative	breast cancer	3.08E-04	569
Jejunum	Alfalfa hay	Positive	organismal death	1.35E-32	898
Jejunum	Alfalfa hay	Positive	morbidity or mortality	2.38E-32	906
Jejunum	Alfalfa hay	Positive	malignant solid tumor	4.12E-32	3249
Jejunum	Alfalfa hay	Positive	cancer	8.39E-32	3282
Jejunum	Alfalfa hay	Positive	proliferation of cells	1.08E-28	1271
Jejunum	Alfalfa hay	Positive	organization of cytoplasm	1.56E-24	590
Jejunum	Alfalfa hay	Positive	cell death	9.09E-24	1140
Jejunum	Alfalfa hay	Positive	necrosis	2.88E-21	898
Jejunum	Alfalfa hay	Positive	organization of cytoskeleton	2.00E-20	529
Jejunum	Alfalfa hay	Positive	morphology of cells	1.47E-19	698
Jejunum	Alfalfa hay	Positive	epithelial cancer	2.05E-19	2664
Jejunum	Alfalfa hay	Positive	neoplasia of epithelial tissue	8.72E-19	2683
Jejunum	Alfalfa hay	Positive	tumorigenesis of tissue	1.46E-18	2722
Jejunum	Alfalfa	Positive	apoptosis	3.41E-18	902

	hay				
Jejunum	Alfalfa hay	Positive	abdominal neoplasm	2.17E-17	2655
Jejunum	Alfalfa hay	Positive	transport of molecule	4.55E-17	582
Jejunum	Alfalfa hay	Positive	microtubule dynamics	5.95E-17	446
Jejunum	Alfalfa hay	Positive	abdominal cancer	1.32E-16	2621
Jejunum	Alfalfa hay	Positive	cellular homeostasis	4.21E-16	549
Jejunum	Alfalfa hay	Positive	cell movement	1.59E-15	732
Jejunum	Alfalfa hay	Positive	cell death of tumor cell lines	3.13E-15	546
Jejunum	Alfalfa hay	Positive	expression of RNA	1.70E-14	732
Jejunum	Alfalfa hay	Positive	digestive system cancer	3.38E-14	2275
Jejunum	Alfalfa hay	Positive	differentiation of cells	6.09E-14	760
Jejunum	Alfalfa hay	Positive	migration of cells	8.82E-14	654
Jejunum	Alfalfa hay	Positive	cell movement of tumor cell lines	1.41E-13	324
Jejunum	Alfalfa hay	Positive	digestive organ tumor	1.67E-13	2289
Jejunum	Alfalfa hay	Positive	formation of cellular protrusions	3.53E-13	339
Jejunum	Alfalfa hay	Positive	transcription	5.55E-13	677
Jejunum	Alfalfa hay	Positive	apoptosis of tumor cell lines	6.51E-13	436
Jejunum	Alfalfa hay	Positive	quantity of cells	1.40E-12	606
Jejunum	Alfalfa hay	Positive	morphology of cardiovascular system	2.07E-12	242
Jejunum	Alfalfa hay	Positive	development of body trunk	3.21E-12	403
Jejunum	Alfalfa hay	Positive	transcription of RNA	6.61E-12	629
Jejunum	Alfalfa hay	Positive	angiogenesis	9.48E-12	334
Jejunum	Alfalfa	Positive	vasculogenesis	1.26E-11	278

	hay				
Jejunum	Alfalfa hay	Positive	Movement Disorders	3.18E-11	380
Jejunum	Alfalfa hay	Positive	Growth Failure	4.12E-11	230
Jejunum	Alfalfa hay	Positive	organization of organelle	8.01E-11	209
Jejunum	Alfalfa hay	Positive	morphology of body cavity	9.00E-11	420
Jejunum	Alfalfa hay	Positive	abnormal morphology of cardiovascular system	1.06E-10	218
Jejunum	Alfalfa hay	Positive	size of body	1.11E-10	311
Jejunum	Alfalfa hay	Positive	abnormal morphology of thoracic cavity	1.28E-10	224
Jejunum	Alfalfa hay	Positive	cell death of connective tissue cells	1.62E-10	229
Jejunum	Alfalfa hay	Positive	migration of tumor cell lines	1.94E-10	263
Jejunum	Alfalfa hay	Positive	development of vasculature	2.44E-10	166
Jejunum	Alfalfa hay	Positive	abnormal morphology of cells	2.56E-10	439
Jejunum	Alfalfa hay	Positive	abnormal morphology of body cavity	3.98E-10	400
Jejunum	Alfalfa hay	Positive	neurological signs	5.01E-10	243
Jejunum	Alfalfa hay	Positive	dyskinesia	7.17E-10	231
Jejunum	Alfalfa hay	Positive	cell survival	8.71E-10	475
Jejunum	Alfalfa hay	Positive	development of lymphatic system	1.62E-09	142
Jejunum	Alfalfa hay	Positive	disorder of basal ganglia	2.03E-09	278
Jejunum	Alfalfa hay	Positive	chorea	3.40E-09	215
Jejunum	Alfalfa hay	Positive	Bleeding	3.40E-09	164
Jejunum	Alfalfa hay	Positive	neuromuscular disease	3.42E-09	314
Jejunum	Alfalfa hay	Positive	concentration of lipid	3.44E-09	291
Jejunum	Alfalfa	Positive	Huntington's Disease	3.80E-09	214

	hay				
Jejunum	Alfalfa hay	Positive	quantity of blood cells	3.83E-09	359
Jejunum	Alfalfa hay	Positive	quantity of leukocytes	3.89E-09	324
Jejunum	Alfalfa hay	Positive	behavior	4.02E-09	347
Jejunum	Alfalfa hay	Positive	formation of cytoskeleton	4.78E-09	147
Jejunum	Alfalfa hay	Positive	development of lymphatic system component	4.87E-09	124
Jejunum	Alfalfa hay	Positive	development of blood cells	4.87E-09	239
Jejunum	Alfalfa hay	Positive	invasion of cells	7.02E-09	308
Jejunum	Alfalfa hay	Positive	proliferation of tumor cell lines	7.18E-09	516
Jejunum	Alfalfa hay	Positive	Viral Infection	7.94E-09	535
Jejunum	Alfalfa hay	Positive	growth of organism	7.94E-09	297
Jejunum	Alfalfa hay	Positive	development of leukocytes	8.33E-09	217
Jejunum	Alfalfa hay	Positive	development of cytoplasm	8.48E-09	175
Jejunum	Alfalfa hay	Positive	cell viability	1.00E-08	440
Jejunum	Alfalfa hay	Positive	growth of embryo	1.54E-08	179
Jejunum	Alfalfa hay	Positive	quantity of lymphatic system component	2.40E-08	145
Jejunum	Alfalfa hay	Positive	size of embryo	4.47E-08	137
Jejunum	Alfalfa hay	Positive	autosomal recessive disease	4.47E-08	320
Jejunum	Alfalfa hay	Positive	growth of lesion	4.47E-08	281
Jejunum	Alfalfa hay	Positive	formation of lymphatic system component	5.02E-08	115
Jejunum	Alfalfa hay	Positive	fibrogenesis	5.59E-08	155
Jejunum	Alfalfa hay	Positive	growth of tumor	5.62E-08	280
Jejunum	Alfalfa	Positive	morphology of heart	5.73E-08	153

	hay				
Jejunum	Alfalfa hay	Positive	transcription of DNA	5.73E-08	506
Jejunum	Alfalfa hay	Positive	quantity of lymphoid organ	5.76E-08	120
Jejunum	Alfalfa hay	Positive	formation of muscle	6.01E-08	150
Jejunum	Alfalfa hay	Positive	protein kinase cascade	6.49E-08	158
Jejunum	Alfalfa hay	Positive	formation of filaments	6.89E-08	150
Jejunum	Alfalfa hay	Positive	development of connective tissue	7.17E-08	141
Jejunum	Alfalfa hay	Positive	development of lymphocytes	8.48E-08	201
Jejunum	Alfalfa hay	Positive	development of mononuclear leukocytes	1.04E-07	202
Jejunum	Alfalfa hay	Positive	development of hematopoietic system	1.21E-07	99
Jejunum	Alfalfa hay	Positive	cell cycle progression	1.29E-07	356
Jejunum	Alfalfa hay	Positive	cell movement of endothelial cells	1.53E-07	134
Jejunum	Alfalfa hay	Positive	phosphorylation of protein	1.76E-07	258
Jejunum	Alfalfa hay	Positive	homeostasis of leukocytes	1.79E-07	200
Jejunum	Alfalfa hay	Positive	migration of endothelial cells	2.06E-07	125
Jejunum	Alfalfa hay	Positive	colony formation of cells	2.31E-07	179
Jejunum	Alfalfa hay	Positive	formation of T lymphocytes	2.55E-07	52
Jejunum	Alfalfa hay	Positive	T cell development	2.97E-07	182
Jejunum	Alfalfa hay	Positive	colony formation	3.02E-07	193
Jejunum	Alfalfa hay	Positive	formation of cells	3.16E-07	334
Jejunum	Alfalfa hay	Positive	concentration of fatty acid	3.20E-07	108
Jejunum	Alfalfa hay	Positive	function of leukocytes	3.33E-07	193
Jejunum	Alfalfa	Positive	formation of lymphocytes	4.28E-07	55

	hay				
Jejunum	Alfalfa hay	Positive	Lymphocyte homeostasis	4.33E-07	195
Jejunum	Alfalfa hay	Positive	cell viability of tumor cell lines	5.56E-07	269
Jejunum	Alfalfa hay	Positive	differentiation of blood cells	6.47E-07	289
Jejunum	Alfalfa hay	Positive	formation of lymphoid organ	6.47E-07	102
Jejunum	Alfalfa hay	Positive	development of epithelial tissue	6.63E-07	185
Jejunum	Alfalfa hay	Positive	migration of blood cells	6.68E-07	298
Jejunum	Alfalfa hay	Positive	formation of leukocytes	6.68E-07	66
Jejunum	Alfalfa hay	Positive	activation of DNA endogenous promoter	6.68E-07	401
Jejunum	Alfalfa hay	Positive	congenital anomaly of musculoskeletal system	6.68E-07	255
Jejunum	Alfalfa hay	Positive	morphology of vessel	6.68E-07	116
Jejunum	Alfalfa hay	Positive	abnormal morphology of heart	8.08E-07	141
Jejunum	Alfalfa hay	Positive	leukocyte migration	8.08E-07	297
Jejunum	Alfalfa hay	Positive	accumulation of lipid	8.08E-07	113
Jejunum	Alfalfa hay	Positive	proliferation of connective tissue cells	8.20E-07	209
Jejunum	Alfalfa hay	Positive	formation of mononuclear leukocytes	8.24E-07	56
Jejunum	Alfalfa hay	Positive	formation of thymocytes	8.31E-07	47
Jejunum	Alfalfa hay	Positive	formation of thymus gland	8.78E-07	67
Jejunum	Alfalfa hay	Positive	T cell homeostasis	9.04E-07	184
Jejunum	Alfalfa hay	Positive	differentiation of leukocytes	9.15E-07	234
Jejunum	Alfalfa hay	Positive	neuritogenesis	1.20E-06	205
Jejunum	Alfalfa hay	Positive	apoptosis of fibroblast cell lines	1.26E-06	122
Jejunum	Alfalfa	Positive	function of blood cells	1.26E-06	206

	hay				
Jejunum	Alfalfa hay	Positive	learning	1.63E-06	151
Jejunum	Alfalfa hay	Positive	proliferation of neuronal cells	1.70E-06	202
Jejunum	Alfalfa hay	Positive	quantity of thymocytes	1.99E-06	89
Jejunum	Alfalfa hay	Positive	invasion of tumor cell lines	2.02E-06	230
Jejunum	Alfalfa hay	Positive	quantity of mononuclear leukocytes	2.07E-06	252
Jejunum	Alfalfa hay	Positive	cell movement of myeloid cells	2.11E-06	190
Jejunum	Alfalfa hay	Positive	development of hematopoietic progenitor cells	2.19E-06	76
Jejunum	Alfalfa hay	Positive	growth of connective tissue	2.30E-06	223
Jejunum	Alfalfa hay	Positive	perinatal death	2.46E-06	207
Jejunum	Alfalfa hay	Positive	cognition	2.48E-06	162
Jejunum	Alfalfa hay	Positive	autophagy	2.63E-06	136
Jejunum	Alfalfa hay	Positive	cell death of fibroblast cell lines	2.75E-06	153
Jejunum	Alfalfa hay	Positive	quantity of hematopoietic cells	2.88E-06	158
Jejunum	Alfalfa hay	Positive	abnormal morphology of abdomen	2.93E-06	295
Jejunum	Alfalfa hay	Positive	metabolism of carbohydrate	2.99E-06	215
Jejunum	Alfalfa hay	Positive	mitosis	3.18E-06	175
Jejunum	Alfalfa hay	Positive	formation of plasma membrane projections	3.22E-06	207
Jejunum	Alfalfa hay	Positive	quantity of carbohydrate	3.22E-06	182
Jejunum	Alfalfa hay	Positive	quantity of lymphocytes	3.23E-06	242
Jejunum	Alfalfa hay	Positive	ion homeostasis of cells	3.52E-06	194
Jejunum	Alfalfa hay	Positive	quantity of hematopoietic progenitor cells	3.70E-06	157
Jejunum	Alfalfa	Positive	formation of actin stress fibers	3.84E-06	91

	hay				
Jejunum	Alfalfa hay	Positive	formation of blood cells	3.93E-06	77
Jejunum	Alfalfa hay	Positive	morphology of blood vessel	4.03E-06	105
Jejunum	Alfalfa hay	Positive	cell transformation	4.03E-06	163
Jejunum	Alfalfa hay	Positive	morphology of connective tissue	4.03E-06	163
Jejunum	Alfalfa hay	Positive	activation of cells	4.19E-06	325
Jejunum	Alfalfa hay	Positive	homing of cells	4.19E-06	194
Jejunum	Alfalfa hay	Positive	mitosis of tumor cell lines	4.41E-06	55
Jejunum	Alfalfa hay	Positive	degeneration of cells	4.45E-06	125
Jejunum	Alfalfa hay	Positive	homing	4.45E-06	199
Jejunum	Alfalfa hay	Positive	cell death of cervical cancer cell lines	4.81E-06	127
Jejunum	Alfalfa hay	Positive	synthesis of carbohydrate	5.04E-06	151
Jejunum	Alfalfa hay	Positive	inflammatory response	5.13E-06	263
Jejunum	Alfalfa hay	Positive	development of cardiovascular tissue	5.13E-06	133
Jejunum	Alfalfa hay	Positive	growth of muscle tissue	5.67E-06	134
Jejunum	Alfalfa hay	Positive	cell movement of leukocytes	5.76E-06	259
Jejunum	Alfalfa hay	Positive	abnormal morphology of embryonic tissue	6.03E-06	190
Jejunum	Alfalfa hay	Positive	cell spreading	7.60E-06	102
Jejunum	Alfalfa hay	Positive	morphology of head	7.60E-06	292
Jejunum	Alfalfa hay	Positive	development of neurons	7.81E-06	263
Jejunum	Alfalfa hay	Positive	proliferation of blood cells	9.85E-06	281
Jejunum	Alfalfa hay	Positive	growth of embryonic tissue	1.06E-05	101
Jejunum	Alfalfa	Positive	uptake of monosaccharide	1.10E-05	99

	hay				
Jejunum	Alfalfa hay	Positive	sprouting	1.10E-05	137
Jejunum	Alfalfa hay	Positive	synthesis of lipid	1.11E-05	241
Jejunum	Alfalfa hay	Positive	proliferation of muscle cells	1.15E-05	132
Jejunum	Alfalfa hay	Positive	formation of hematopoietic progenitor cells	1.17E-05	52
Jejunum	Alfalfa hay	Positive	immune response of cells	1.38E-05	196
Jejunum	Alfalfa hay	Positive	cell death of muscle	1.38E-05	123
Jejunum	Alfalfa hay	Positive	development of endothelial tissue	1.40E-05	130
Jejunum	Alfalfa hay	Positive	proliferation of embryonic cell lines	1.44E-05	62
Jejunum	Alfalfa hay	Positive	cell death of muscle cells	1.51E-05	120
Jejunum	Alfalfa hay	Positive	formation of actin filaments	1.53E-05	110
Jejunum	Alfalfa hay	Positive	differentiation of T lymphocytes	1.54E-05	134
Jejunum	Alfalfa hay	Positive	autosomal dominant disease	1.56E-05	216
Jejunum	Alfalfa hay	Positive	adenocarcinoma	1.57E-05	1924
Jejunum	Alfalfa hay	Positive	seizure disorder	1.63E-05	163
Jejunum	Alfalfa hay	Positive	morphology of respiratory system	1.63E-05	112
Jejunum	Alfalfa hay	Positive	cell movement of granulocytes	1.69E-05	131
Jejunum	Alfalfa hay	Positive	abnormal morphology of blood vessel	1.72E-05	97
Jejunum	Alfalfa hay	Positive	necrosis of muscle	1.78E-05	122
Jejunum	Alfalfa hay	Positive	abnormal morphology of head	1.93E-05	276
Jejunum	Alfalfa hay	Positive	seizures	1.93E-05	140
Jejunum	Alfalfa hay	Positive	uptake of carbohydrate	1.93E-05	105
Jejunum	Alfalfa	Positive	differentiation of connective tissue cells	2.07E-05	217

	hay				
Jejunum	Alfalfa hay	Positive	cell movement of neutrophils	2.12E-05	107
Jejunum	Alfalfa hay	Positive	abnormal morphology of respiratory system	2.24E-05	110
Jejunum	Alfalfa hay	Positive	chemotaxis	2.32E-05	186
Jejunum	Alfalfa hay	Positive	proliferation of smooth muscle cells	2.32E-05	103
Jejunum	Alfalfa hay	Positive	endothelial cell development	2.32E-05	125
Jejunum	Alfalfa hay	Positive	cell death of epithelial cell lines	2.36E-05	102
Jejunum	Alfalfa hay	Positive	quantity of connective tissue	2.46E-05	204
Jejunum	Alfalfa hay	Positive	proliferation of embryonic cells	2.51E-05	91
Jejunum	Alfalfa hay	Positive	growth of epithelial tissue	2.57E-05	228
Jejunum	Alfalfa hay	Positive	chemotaxis of cells	2.74E-05	180
Jejunum	Alfalfa hay	Positive	proliferation of endothelial cells	2.80E-05	111
Jejunum	Alfalfa hay	Positive	apoptosis of connective tissue cells	2.80E-05	106
Jejunum	Alfalfa hay	Positive	degeneration of nervous system	3.04E-05	103
Jejunum	Alfalfa hay	Positive	gastrointestinal carcinoma	3.14E-05	1357
Jejunum	Alfalfa hay	Positive	mass of organism	3.31E-05	118
Jejunum	Alfalfa hay	Positive	epileptic seizure	3.34E-05	64
Jejunum	Alfalfa hay	Positive	morphogenesis of neurons	3.47E-05	147
Jejunum	Alfalfa hay	Positive	abdominal adenocarcinoma	3.47E-05	1750
Jejunum	Alfalfa hay	Positive	gastrointestinal tract cancer	3.93E-05	1507
Jejunum	Alfalfa hay	Positive	cell movement of phagocytes	4.00E-05	186
Jejunum	Alfalfa hay	Positive	organization of actin cytoskeleton	4.08E-05	107
Jejunum	Alfalfa	Positive	proliferation of central nervous system cells	4.08E-05	58

	hay				
Jejunum	Alfalfa hay	Positive	benign neoplasia	4.16E-05	302
Jejunum	Alfalfa hay	Positive	small GTPase mediated signal transduction	4.40E-05	69
Jejunum	Alfalfa hay	Positive	apoptosis of epithelial cell lines	4.40E-05	82
Jejunum	Alfalfa hay	Positive	apoptosis of B-lymphocyte derived cell lines	4.75E-05	49
Jejunum	Alfalfa hay	Positive	skin abnormality	5.15E-05	83
Jejunum	Alfalfa hay	Positive	ubiquitination	5.43E-05	118
Jejunum	Alfalfa hay	Positive	differentiation of lymphocytes	5.48E-05	169
Jejunum	Alfalfa hay	Positive	infection by Retroviridae	5.69E-05	238
Jejunum	Alfalfa hay	Positive	proliferation of fibroblasts	5.73E-05	123
Jejunum	Alfalfa hay	Positive	dysgenesis	5.74E-05	182
Jejunum	Alfalfa hay	Positive	differentiation of connective tissue	6.16E-05	241
Jejunum	Alfalfa hay	Positive	fatty acid metabolism	6.16E-05	201
Jejunum	Alfalfa hay	Positive	engulfment of cells	6.38E-05	140
Jejunum	Alfalfa hay	Positive	angiogenesis of lesion	6.38E-05	50
Jejunum	Alfalfa hay	Positive	Hypertrophy	6.39E-05	161
Jejunum	Alfalfa hay	Positive	ubiquitination of protein	6.45E-05	116
Jejunum	Alfalfa hay	Positive	branching of cells	6.60E-05	128
Jejunum	Alfalfa hay	Positive	apoptosis of pheochromocytoma cell lines	6.83E-05	38
Jejunum	Alfalfa hay	Positive	quantity of double-positive thymocyte	6.92E-05	45
Jejunum	Alfalfa hay	Positive	Organ Degeneration	7.12E-05	167
Jejunum	Alfalfa hay	Positive	Hypertension	7.21E-05	213
Jejunum	Alfalfa	Positive	quantity of T lymphocytes	7.44E-05	180

	hay				
Jejunum	Alfalfa hay	Positive	cell death of B-lymphocyte derived cell lines	7.92E-05	50
Jejunum	Alfalfa hay	Positive	peripheral vascular disease	8.18E-05	131
Jejunum	Alfalfa hay	Positive	cell viability of cervical cancer cell lines	8.32E-05	80
Jejunum	Alfalfa hay	Positive	morphogenesis of neurites	8.44E-05	143
Jejunum	Alfalfa hay	Positive	transmembrane potential of mitochondria	8.45E-05	78
Jejunum	Alfalfa hay	Positive	cell death of fibroblasts	8.45E-05	94
Jejunum	Alfalfa hay	Positive	proliferation of hematopoietic progenitor cells	8.55E-05	87
Jejunum	Alfalfa hay	Positive	morphology of blood cells	8.68E-05	142
Jejunum	Alfalfa hay	Positive	proliferation of immune cells	8.68E-05	259
Jejunum	Alfalfa hay	Positive	mitosis of cervical cancer cell lines	8.68E-05	40
Jejunum	Alfalfa hay	Positive	transactivation	8.68E-05	193
Jejunum	Alfalfa hay	Positive	Hypoplasia	8.78E-05	170
Jejunum	Alfalfa hay	Positive	breast or colorectal cancer	8.84E-05	1476
Jejunum	Alfalfa hay	Positive	Neurodegeneration	9.27E-05	108
Jejunum	Alfalfa hay	Positive	breast or ovarian cancer	9.42E-05	592
Jejunum	Alfalfa hay	Positive	Thrombosis	9.68E-05	63
Jejunum	Alfalfa hay	Positive	cartilage development	9.68E-05	55
Jejunum	Alfalfa hay	Positive	cell death of tumor	9.81E-05	131
Jejunum	Alfalfa hay	Positive	morphology of nervous system	9.82E-05	256
Jejunum	Alfalfa hay	Positive	angiogenesis of tumor	9.82E-05	47
Jejunum	Alfalfa hay	Positive	plasticity of synapse	9.82E-05	47
Jejunum	Alfalfa	Positive	movement of vascular endothelial cells	9.88E-05	67

	hay				
Jejunum	Alfalfa hay	Positive	Edema	9.88E-05	119
Jejunum	Alfalfa hay	Positive	development of abdomen	9.99E-05	193
Jejunum	Alfalfa hay	Positive	shape change of tumor cell lines	1.04E-04	58
Jejunum	Alfalfa hay	Positive	Gastrointestinal Tract Cancer and Tumors	1.06E-04	1524
Jejunum	Alfalfa hay	Positive	morphology of muscle	1.10E-04	116
Jejunum	Alfalfa hay	Positive	formation of lung	1.13E-04	105
Jejunum	Alfalfa hay	Positive	polymerization of protein	1.13E-04	127
Jejunum	Alfalfa hay	Positive	mass of connective tissue	1.15E-04	73
Jejunum	Alfalfa hay	Positive	neuronal cell death	1.21E-04	224
Jejunum	Alfalfa hay	Positive	abnormal morphology of epithelial tissue	1.26E-04	142
Jejunum	Alfalfa hay	Positive	bleeding of liver	1.26E-04	18
Jejunum	Alfalfa hay	Positive	proliferation of hematopoietic cells	1.35E-04	90
Jejunum	Alfalfa hay	Positive	abnormal morphology of nervous system	1.36E-04	237
Jejunum	Alfalfa hay	Positive	I-kappaB kinase/NF-kappaB cascade	1.36E-04	64
Jejunum	Alfalfa hay	Positive	HIV infection	1.43E-04	233
Jejunum	Alfalfa hay	Positive	differentiation of muscle cell lines	1.58E-04	60
Jejunum	Alfalfa hay	Positive	function of cardiovascular system	1.65E-04	126
Jejunum	Alfalfa hay	Positive	development of head	1.75E-04	314
Jejunum	Alfalfa hay	Positive	outgrowth of cells	1.76E-04	146
Jejunum	Alfalfa hay	Positive	endocytosis	1.83E-04	118
Jejunum	Alfalfa hay	Positive	apoptosis of cervical cancer cell lines	1.83E-04	98
Jejunum	Alfalfa	Positive	activation of blood cells	1.83E-04	243

	hay				
Jejunum	Alfalfa hay	Positive	migration of cancer cells	1.93E-04	70
Jejunum	Alfalfa hay	Positive	cell movement of tumor cells	1.95E-04	67
Jejunum	Alfalfa hay	Positive	hepatocellular carcinoma	1.95E-04	1247
Jejunum	Alfalfa hay	Positive	necrosis of tumor	1.99E-04	129
Jejunum	Alfalfa hay	Positive	binding of DNA	1.99E-04	173
Jejunum	Alfalfa hay	Positive	metabolism of protein	1.99E-04	311
Jejunum	Alfalfa hay	Positive	genital tumor	2.04E-04	1205
Jejunum	Alfalfa hay	Positive	cellular degradation	2.07E-04	89
Jejunum	Alfalfa hay	Positive	differentiation of mononuclear leukocytes	2.09E-04	179
Jejunum	Alfalfa hay	Positive	cell death of breast cancer cell lines	2.09E-04	114
Jejunum	Alfalfa hay	Positive	synthesis of reactive oxygen species	2.12E-04	160
Jejunum	Alfalfa hay	Positive	cell death of pheochromocytoma cell lines	2.23E-04	46
Jejunum	Alfalfa hay	Positive	cell death of heart cells	2.32E-04	78
Jejunum	Alfalfa hay	Positive	outgrowth of plasma membrane projections	2.32E-04	137
Jejunum	Alfalfa hay	Positive	metastasis	2.38E-04	246
Jejunum	Alfalfa hay	Positive	vascularization	2.39E-04	83
Jejunum	Alfalfa hay	Positive	outgrowth of neurites	2.42E-04	136
Jejunum	Alfalfa hay	Positive	autophagy of cells	2.45E-04	92
Jejunum	Alfalfa hay	Positive	transport of alpha-amino acid	2.45E-04	30
Jejunum	Alfalfa hay	Positive	synthesis of DNA	2.45E-04	140
Jejunum	Alfalfa hay	Positive	apoptosis of muscle	2.51E-04	91
Jejunum	Alfalfa	Positive	cell death of stem cells	2.59E-04	34

	hay				
Jejunum	Alfalfa hay	Positive	mass of adipose tissue	2.61E-04	68
Jejunum	Alfalfa hay	Positive	hypoplasia of organ	2.62E-04	149
Jejunum	Alfalfa hay	Positive	cardiogenesis	2.62E-04	157
Jejunum	Alfalfa hay	Positive	proliferation of mononuclear leukocytes	2.65E-04	239
Jejunum	Alfalfa hay	Positive	infection by RNA virus	2.65E-04	278
Jejunum	Alfalfa hay	Positive	ruffling	2.66E-04	42
Jejunum	Alfalfa hay	Positive	quantity of metal	2.81E-04	165
Jejunum	Alfalfa hay	Positive	proliferation of epithelial cell lines	2.82E-04	81
Jejunum	Alfalfa hay	Positive	liver tumor	2.83E-04	1292
Jejunum	Alfalfa hay	Positive	organization of filaments	2.83E-04	73
Jejunum	Alfalfa hay	Positive	neurodegeneration of cerebral cortex	2.89E-04	25
Jejunum	Alfalfa hay	Positive	morphology of cardiovascular tissue	2.89E-04	35
Jejunum	Alfalfa hay	Positive	cell death of neuroblastoma cell lines	2.97E-04	69
Jejunum	Alfalfa hay	Positive	development of body axis	2.99E-04	333
Jejunum	Alfalfa hay	Positive	cell death of melanoma cell lines	3.15E-04	87
Jejunum	Alfalfa hay	Positive	liver cancer	3.16E-04	1283
Jejunum	Alfalfa hay	Positive	abdominal carcinoma	3.16E-04	1760
Jejunum	Alfalfa hay	Positive	proliferation of lymphocytes	3.16E-04	235
Jejunum	Alfalfa hay	Positive	advanced malignant tumor	3.20E-04	271
Jejunum	Alfalfa hay	Positive	apoptosis of muscle cells	3.25E-04	90
Jejunum	Alfalfa hay	Positive	export of molecule	3.26E-04	101
Jejunum	Alfalfa	Positive	metabolism of reactive oxygen species	3.26E-04	165

	hay				
Jejunum	Alfalfa hay	Positive	intracranial hemorrhage	3.27E-04	52
Jejunum	Alfalfa hay	Positive	morphology of lymphatic system component	3.30E-04	139
Jejunum	Alfalfa hay	Positive	congenital malformation of skeleton	3.31E-04	141
Jejunum	Alfalfa hay	Positive	memory	3.31E-04	89
Jejunum	Alfalfa hay	Positive	cell death of tumor cells	3.33E-04	125
Jejunum	Alfalfa hay	Positive	secretory pathway	3.34E-04	70
Jejunum	Alfalfa hay	Positive	transport of carbohydrate	3.38E-04	69
Jejunum	Alfalfa hay	Positive	homeostasis of ion	3.41E-04	83
Jejunum	Alfalfa hay	Positive	proliferation of T lymphocytes	3.57E-04	197
Jejunum	Alfalfa hay	Positive	cell death of embryonic cells	3.64E-04	44
Jejunum	Alfalfa hay	Positive	apoptosis of carcinoma cell lines	3.69E-04	85
Jejunum	Alfalfa hay	Positive	differentiation of tumor cell lines	3.73E-04	127
Jejunum	Alfalfa hay	Positive	female genital tract serous cancer	3.78E-04	129
Jejunum	Alfalfa hay	Positive	necrosis of epithelial tissue	3.87E-04	192
Jejunum	Alfalfa hay	Positive	degeneration of neurons	3.89E-04	83
Jejunum	Alfalfa hay	Positive	hepatobiliary system cancer	3.91E-04	1287
Jejunum	Alfalfa hay	Positive	survival of organism	3.91E-04	227
Jejunum	Alfalfa hay	Positive	reorganization of cytoskeleton	3.95E-04	68
Jejunum	Alfalfa hay	Positive	gastrointestinal adenocarcinoma	4.04E-04	1266
Jejunum	Alfalfa hay	Positive	cleavage of cells	4.08E-04	56
Jejunum	Alfalfa hay	Positive	congenital anomaly of skin	4.08E-04	64
Jejunum	Alfalfa	Positive	formation of muscle cells	4.08E-04	64

	hay				
Jejunum	Alfalfa hay	Positive	migration of vascular endothelial cells	4.13E-04	60
Jejunum	Alfalfa hay	Positive	mammary tumor	4.14E-04	505
Jejunum	Alfalfa hay	Positive	synthesis of glycosaminoglycan	4.17E-04	34
Jejunum	Alfalfa hay	Positive	cancer of secretory structure	4.17E-04	601
Jejunum	Alfalfa hay	Positive	abnormal morphology of digestive system	4.25E-04	178
Jejunum	Alfalfa hay	Positive	apoptosis of breast cancer cell lines	4.42E-04	98
Jejunum	Alfalfa hay	Positive	concentration of phosphatidic acid	4.42E-04	50
Jejunum	Alfalfa hay	Positive	uptake of D-glucose	4.42E-04	77
Jejunum	Alfalfa hay	Positive	respiratory system development	4.42E-04	115
Jejunum	Alfalfa hay	Positive	serous neoplasm	4.47E-04	198
Jejunum	Alfalfa hay	Positive	growth of lymphatic system component	4.50E-04	68
Jejunum	Alfalfa hay	Positive	melanoma	4.70E-04	1940
Jejunum	Alfalfa hay	Positive	interphase	4.77E-04	212
Jejunum	Alfalfa hay	Positive	consumption of oxygen	4.77E-04	56
Jejunum	Alfalfa hay	Positive	long-term potentiation	4.77E-04	84
Jejunum	Alfalfa hay	Positive	cell death of heart	4.78E-04	79
Jejunum	Alfalfa hay	Positive	activation of leukocytes	4.79E-04	225
Jejunum	Alfalfa hay	Positive	cell movement of carcinoma cell lines	4.85E-04	72
Jejunum	Alfalfa hay	Positive	migration of tumor cells	4.88E-04	78
Jejunum	Alfalfa hay	Positive	cytopenia	4.89E-04	101
Jejunum	Alfalfa hay	Positive	proliferation of embryoblast	5.02E-04	13
Jejunum	Alfalfa	Positive	cell death of carcinoma cell lines	5.04E-04	97

	hay				
Jejunum	Alfalfa hay	Positive	formation of vascular lesion	5.07E-04	43
Jejunum	Alfalfa hay	Positive	occlusion of artery	5.09E-04	156
Jejunum	Alfalfa hay	Positive	metastatic colorectal cancer	5.15E-04	68
Jejunum	Alfalfa hay	Positive	differentiation of bone	5.16E-04	136
Jejunum	Alfalfa hay	Positive	prostatic disease	5.16E-04	312
Jejunum	Alfalfa hay	Positive	quantity of metal ion	5.16E-04	146
Jejunum	Alfalfa hay	Positive	cell death of blood cells	5.16E-04	223
Jejunum	Alfalfa hay	Positive	exocytosis	5.16E-04	67
Jejunum	Alfalfa hay	Positive	differentiation of epithelial tissue	5.26E-04	120
Jejunum	Alfalfa hay	Positive	morphology of leukocytes	5.28E-04	115
Jejunum	Alfalfa hay	Positive	Oral Cancer and Tumors	5.32E-04	79
Jejunum	Alfalfa hay	Positive	synthesis of protein	5.35E-04	147
Jejunum	Alfalfa hay	Positive	size of lesion	5.45E-04	87
Jejunum	Alfalfa hay	Positive	transport of monosaccharide	5.50E-04	57
Jejunum	Alfalfa hay	Positive	function of myeloid cells	5.57E-04	82
Jejunum	Alfalfa hay	Positive	vaso-occlusion	5.65E-04	157
Jejunum	Alfalfa hay	Positive	growth of plasma membrane projections	5.73E-04	153
Jejunum	Alfalfa hay	Positive	concentration of Ca2+	5.75E-04	47
Jejunum	Alfalfa hay	Positive	apoptosis of muscle cell lines	5.84E-04	27
Jejunum	Alfalfa hay	Positive	cell spreading of fibroblast cell lines	5.84E-04	24
Jejunum	Alfalfa hay	Positive	transport of ion	5.84E-04	165
Jejunum	Alfalfa	Positive	male genital neoplasm	5.84E-04	326

	hay				
Jejunum	Alfalfa hay	Positive	function of phagocytes	6.16E-04	103
Jejunum	Alfalfa hay	Positive	size of animal	6.24E-04	63
Jejunum	Alfalfa hay	Positive	morphology of airway	6.26E-04	14
Jejunum	Alfalfa hay	Positive	cell viability of carcinoma cell lines	6.27E-04	52
Jejunum	Alfalfa hay	Positive	transport of D-glucose	6.27E-04	52
Jejunum	Alfalfa hay	Positive	morphology of digestive system	6.28E-04	185
Jejunum	Alfalfa hay	Positive	morphology of pulmonary alveolus	6.28E-04	44
Jejunum	Alfalfa hay	Positive	cytostasis of tumor cell lines	6.40E-04	57
Jejunum	Alfalfa hay	Positive	metabolism of nucleotide	6.60E-04	144
Jejunum	Alfalfa hay	Positive	growth of thymus gland	6.66E-04	37
Jejunum	Alfalfa hay	Positive	cell death of epithelial cells	6.67E-04	162
Jejunum	Alfalfa hay	Positive	abnormal morphology of muscle	6.71E-04	106
Jejunum	Alfalfa hay	Positive	bleeding of brain	6.89E-04	28
Jejunum	Alfalfa hay	Positive	muscle tumor	6.91E-04	120
Jejunum	Alfalfa hay	Positive	arthropathy	6.91E-04	300
Jejunum	Alfalfa hay	Positive	oral cancer	6.95E-04	78
Jejunum	Alfalfa hay	Positive	abnormal morphology of conducting airway	7.05E-04	12
Jejunum	Alfalfa hay	Positive	accumulation of cells	7.07E-04	117
Jejunum	Alfalfa hay	Positive	occlusion of blood vessel	7.07E-04	157
Jejunum	Alfalfa hay	Positive	dysmyelination	7.09E-04	49
Jejunum	Alfalfa hay	Positive	differentiation of bone cells	7.15E-04	134
Jejunum	Alfalfa	Positive	growth of neurites	7.24E-04	151

	hay				
Jejunum	Alfalfa hay	Positive	arthritis	7.24E-04	295
Jejunum	Alfalfa hay	Positive	Prostate Cancer and Tumors	7.28E-04	306
Jejunum	Alfalfa hay	Positive	cell movement of cancer cells	7.37E-04	53
Jejunum	Alfalfa hay	Positive	abnormal morphology of artery	7.38E-04	54
Jejunum	Alfalfa hay	Positive	death of embryo	7.38E-04	54
Jejunum	Alfalfa hay	Positive	proliferation of hepatoma cell lines	7.38E-04	69
Jejunum	Alfalfa hay	Positive	epilepsy	7.38E-04	103
Jejunum	Alfalfa hay	Positive	transactivation of RNA	7.43E-04	176
Jejunum	Alfalfa hay	Positive	morphology of heart ventricle	7.67E-04	67
Jejunum	Alfalfa hay	Positive	peripheral arterial disease	7.76E-04	73
Jejunum	Alfalfa hay	Positive	apoptosis of heart cells	7.79E-04	66
Jejunum	Alfalfa hay	Positive	neurodegeneration of hippocampus	7.93E-04	19
Jejunum	Alfalfa hay	Positive	oral tumor	7.97E-04	101
Jejunum	Alfalfa hay	Positive	concentration of phospholipid	8.02E-04	64
Jejunum	Alfalfa hay	Positive	morphology of lymphoid organ	8.02E-04	129
Jejunum	Alfalfa hay	Positive	accumulation of blood cells	8.32E-04	94
Jejunum	Alfalfa hay	Positive	morphology of spleen	8.32E-04	94
Jejunum	Alfalfa hay	Positive	proliferation of tumor cells	8.32E-04	153
Jejunum	Alfalfa hay	Positive	attachment of cells	8.32E-04	49
Jejunum	Alfalfa hay	Positive	migration of carcinoma cell lines	8.37E-04	60
Jejunum	Alfalfa hay	Positive	replication of virus	8.49E-04	181
Jejunum	Alfalfa	Positive	hypertrophy of heart	8.50E-04	112

	hay				
Jejunum	Alfalfa hay	Positive	branching of neurons	8.57E-04	96
Jejunum	Alfalfa hay	Positive	replication of RNA virus	8.74E-04	164
Jejunum	Alfalfa hay	Positive	differentiation of endothelial cells	8.85E-04	30
Jejunum	Alfalfa hay	Positive	demyelination	9.01E-04	44
Jejunum	Alfalfa hay	Positive	multiple congenital anomalies	9.06E-04	138
Jejunum	Alfalfa hay	Positive	malignant gallbladder neoplasia	9.06E-04	13
Jejunum	Alfalfa hay	Positive	proliferation of embryonic stem cell lines	9.06E-04	13
Jejunum	Alfalfa hay	Positive	flux of ion	9.40E-04	107
Jejunum	Alfalfa hay	Positive	transformation of fibroblast cell lines	9.40E-04	87
Jejunum	Alfalfa hay	Positive	cell death of myeloma cell lines	9.40E-04	36
Jejunum	Alfalfa hay	Positive	benign neoplasm of female genital organ	9.42E-04	139
Jejunum	Alfalfa hay	Positive	branching of neurites	9.52E-04	93
Jejunum	Alfalfa hay	Positive	hereditary neoplastic syndrome	9.67E-04	60
Jejunum	Alfalfa hay	Positive	morphology of artery	1.01E-03	55
Jejunum	Alfalfa hay	Positive	neonatal death	1.02E-03	143
Jejunum	Alfalfa hay	Positive	abnormal morphology of pulmonary alveolus	1.02E-03	42
Jejunum	Alfalfa hay	Positive	morphology of lung	1.04E-03	65
Jejunum	Alfalfa hay	Positive	abnormal morphology of bone	1.07E-03	142
Jejunum	Alfalfa hay	Positive	size of cells	1.07E-03	140
Jejunum	Alfalfa hay	Positive	G2 phase of fibroblasts	1.07E-03	14
Jejunum	Alfalfa hay	Positive	abnormal morphology of lymphoid organ	1.08E-03	124
Jejunum	Alfalfa	Positive	apoptosis of myeloma cell lines	1.09E-03	32

	hay				
Jejunum	Alfalfa hay	Positive	morphology of endothelial tissue	1.09E-03	32
Jejunum	Alfalfa hay	Positive	morphology of axial skeleton	1.09E-03	69
Jejunum	Alfalfa hay	Positive	quantity of IgG2a	1.11E-03	39
Jejunum	Alfalfa hay	Positive	cell death of cardiomyocytes	1.13E-03	73
Jejunum	Alfalfa hay	Positive	morphology of bone	1.13E-03	146
Jejunum	Alfalfa hay	Positive	anemia	1.14E-03	114
Jejunum	Alfalfa hay	Positive	Emery-Dreifuss muscular dystrophy	1.14E-03	26
Jejunum	Alfalfa hay	Positive	transport of L-amino acid	1.14E-03	26
Jejunum	Alfalfa hay	Positive	formation of filopodia	1.14E-03	58
Jejunum	Alfalfa hay	Positive	autophosphorylation of protein	1.16E-03	56
Jejunum	Alfalfa hay	Positive	influx of inorganic cation	1.16E-03	66
Jejunum	Alfalfa hay	Positive	secretion of molecule	1.16E-03	177
Jejunum	Alfalfa hay	Positive	metabolism of DNA	1.17E-03	130
Jejunum	Alfalfa hay	Positive	autosomal dominant myopathy	1.17E-03	33
Jejunum	Alfalfa hay	Positive	MAPKKK cascade	1.21E-03	70
Jejunum	Alfalfa hay	Positive	medullary carcinoma	1.22E-03	16
Jejunum	Alfalfa hay	Positive	cell viability of lung cancer cell lines	1.22E-03	42
Jejunum	Alfalfa hay	Positive	flux of inorganic cation	1.22E-03	100
Jejunum	Alfalfa hay	Positive	apoptosis of fibroblasts	1.23E-03	74
Jejunum	Alfalfa hay	Positive	necrosis of cardiac muscle	1.23E-03	74
Jejunum	Alfalfa hay	Positive	malignant neoplasm of male genital organ	1.23E-03	310
Jejunum	Alfalfa	Positive	shape change of neurons	1.23E-03	97

	hay				
Jejunum	Alfalfa hay	Positive	transport of carboxylic acid	1.25E-03	43
Jejunum	Alfalfa hay	Positive	formation of skin	1.26E-03	120
Jejunum	Alfalfa hay	Positive	invasion of tissue	1.28E-03	67
Jejunum	Alfalfa hay	Positive	oral cavity carcinoma	1.28E-03	67
Jejunum	Alfalfa hay	Positive	cell death of fibrosarcoma cell lines	1.29E-03	29
Jejunum	Alfalfa hay	Positive	morphology of aorta	1.30E-03	27
Jejunum	Alfalfa hay	Positive	fertility	1.31E-03	119
Jejunum	Alfalfa hay	Positive	synthesis of fatty acid	1.32E-03	103
Jejunum	Alfalfa hay	Positive	synthesis of nitric oxide	1.34E-03	92
Jejunum	Alfalfa hay	Positive	mixed papillary and follicular thyroid cancer	1.34E-03	10
Jejunum	Alfalfa hay	Positive	abnormal morphology of spleen	1.34E-03	89
Jejunum	Alfalfa hay	Positive	smooth muscle tumor	1.34E-03	100
Jejunum	Alfalfa hay	Positive	function of muscle	1.34E-03	114
Jejunum	Alfalfa hay	Positive	ploidy of cells	1.34E-03	49
Jejunum	Alfalfa hay	Positive	cell death of embryonic stem cells	1.42E-03	21
Jejunum	Alfalfa hay	Positive	flux of cation	1.42E-03	101
Jejunum	Alfalfa hay	Positive	connective or soft tissue tumor	1.42E-03	223
Jejunum	Alfalfa hay	Positive	oral squamous cell carcinoma	1.44E-03	61
Jejunum	Alfalfa hay	Positive	quantity of immunoglobulin	1.45E-03	98
Jejunum	Alfalfa hay	Positive	breast cancer	1.45E-03	466
Jejunum	Alfalfa hay	Positive	laminopathy	1.48E-03	28
Jejunum	Alfalfa	Positive	morphology of lymphocytes	1.49E-03	66

	hay				
Jejunum	Alfalfa hay	Positive	malignant neoplasm of endocrine gland	1.51E-03	102
Jejunum	Alfalfa hay	Positive	metastatic gastrointestinal tract cancer	1.51E-03	75
Jejunum	Alfalfa hay	Negative	proliferation of cells	2.10E-29	1198
Jejunum	Alfalfa hay	Negative	cell death	7.03E-28	1091
Jejunum	Alfalfa hay	Negative	malignant solid tumor	5.07E-25	2994
Jejunum	Alfalfa hay	Negative	cancer	2.48E-24	3023
Jejunum	Alfalfa hay	Negative	necrosis	1.31E-22	850
Jejunum	Alfalfa hay	Negative	organization of cytoplasm	6.17E-22	546
Jejunum	Alfalfa hay	Negative	transport of molecule	1.39E-21	568
Jejunum	Alfalfa hay	Negative	cell movement	3.91E-21	716
Jejunum	Alfalfa hay	Negative	morphology of cells	4.18E-21	663
Jejunum	Alfalfa hay	Negative	apoptosis	7.30E-21	860
Jejunum	Alfalfa hay	Negative	organismal death	7.28E-20	785
Jejunum	Alfalfa hay	Negative	morbidity or mortality	1.87E-19	792
Jejunum	Alfalfa hay	Negative	cell survival	3.50E-18	488
Jejunum	Alfalfa hay	Negative	organization of cytoskeleton	7.97E-18	487
Jejunum	Alfalfa hay	Negative	cell viability	1.22E-17	458
Jejunum	Alfalfa hay	Negative	migration of cells	4.82E-17	631
Jejunum	Alfalfa hay	Negative	cellular homeostasis	8.29E-16	515
Jejunum	Alfalfa hay	Negative	differentiation of cells	2.09E-15	721
Jejunum	Alfalfa hay	Negative	neoplasia of epithelial tissue	3.13E-14	2467
Jejunum	Alfalfa	Negative	tumorigenesis of tissue	5.42E-14	2503

	hay				
Jejunum	Alfalfa hay	Negative	microtubule dynamics	1.16E-13	406
Jejunum	Alfalfa hay	Negative	expression of RNA	1.54E-13	681
Jejunum	Alfalfa hay	Negative	epithelial cancer	2.13E-13	2439
Jejunum	Alfalfa hay	Negative	transcription	3.87E-13	636
Jejunum	Alfalfa hay	Negative	transcription of RNA	4.23E-13	597
Jejunum	Alfalfa hay	Negative	quantity of cells	5.54E-13	571
Jejunum	Alfalfa hay	Negative	abdominal neoplasm	3.43E-12	2434
Jejunum	Alfalfa hay	Negative	development of cytoplasm	5.01E-12	177
Jejunum	Alfalfa hay	Negative	angiogenesis	5.01E-12	316
Jejunum	Alfalfa hay	Negative	vasculogenesis	5.01E-12	264
Jejunum	Alfalfa hay	Negative	concentration of lipid	7.87E-12	286
Jejunum	Alfalfa hay	Negative	abdominal cancer	2.88E-11	2400
Jejunum	Alfalfa hay	Negative	formation of cytoskeleton	4.39E-11	146
Jejunum	Alfalfa hay	Negative	transcription of DNA	4.75E-11	494
Jejunum	Alfalfa hay	Negative	organization of organelle	6.36E-11	198
Jejunum	Alfalfa hay	Negative	cell movement of tumor cell lines	1.33E-10	293
Jejunum	Alfalfa hay	Negative	invasion of cells	1.65E-10	298
Jejunum	Alfalfa hay	Negative	Movement Disorders	1.65E-10	354
Jejunum	Alfalfa hay	Negative	proliferation of tumor cell lines	1.86E-10	494
Jejunum	Alfalfa hay	Negative	quantity of leukocytes	2.35E-10	311
Jejunum	Alfalfa hay	Negative	cell death of tumor cell lines	3.54E-10	486
Jejunum	Alfalfa	Negative	formation of filaments	3.73E-10	150

	hay				
Jejunum	Alfalfa hay	Negative	fibrogenesis	4.89E-10	154
Jejunum	Alfalfa hay	Negative	quantity of blood cells	5.48E-10	342
Jejunum	Alfalfa hay	Negative	cell viability of tumor cell lines	1.67E-09	266
Jejunum	Alfalfa hay	Negative	neuronal cell death	2.13E-09	236
Jejunum	Alfalfa hay	Negative	synthesis of lipid	2.13E-09	246
Jejunum	Alfalfa hay	Negative	size of body	2.13E-09	287
Jejunum	Alfalfa hay	Negative	protein kinase cascade	2.15E-09	155
Jejunum	Alfalfa hay	Negative	transactivation	2.32E-09	204
Jejunum	Alfalfa hay	Negative	abnormal morphology of cells	2.32E-09	407
Jejunum	Alfalfa hay	Negative	formation of cellular protrusions	2.37E-09	302
Jejunum	Alfalfa hay	Negative	dyskinesia	6.94E-09	214
Jejunum	Alfalfa hay	Negative	growth of connective tissue	8.29E-09	222
Jejunum	Alfalfa hay	Negative	cell movement of myeloid cells	8.29E-09	190
Jejunum	Alfalfa hay	Negative	disorder of basal ganglia	8.29E-09	259
Jejunum	Alfalfa hay	Negative	neurological signs	8.61E-09	224
Jejunum	Alfalfa hay	Negative	migration of blood cells	1.10E-08	290
Jejunum	Alfalfa hay	Negative	cell movement of phagocytes	1.29E-08	192
Jejunum	Alfalfa hay	Negative	leukocyte migration	1.41E-08	289
Jejunum	Alfalfa hay	Negative	quantity of carbohydrate	1.55E-08	182
Jejunum	Alfalfa hay	Negative	neuromuscular disease	1.55E-08	292
Jejunum	Alfalfa hay	Negative	proliferation of connective tissue cells	1.55E-08	205
Jejunum	Alfalfa	Negative	homing of cells	1.72E-08	194

	hay				
Jejunum	Alfalfa hay	Negative	Viral Infection	1.89E-08	499
Jejunum	Alfalfa hay	Negative	morphology of body cavity	2.29E-08	381
Jejunum	Alfalfa hay	Negative	transactivation of RNA	2.44E-08	189
Jejunum	Alfalfa hay	Negative	concentration of fatty acid	2.44E-08	106
Jejunum	Alfalfa hay	Negative	formation of actin filaments	2.54E-08	114
Jejunum	Alfalfa hay	Negative	digestive organ tumor	3.02E-08	2084
Jejunum	Alfalfa hay	Negative	development of cardiovascular tissue	3.94E-08	134
Jejunum	Alfalfa hay	Negative	chemotaxis of cells	4.15E-08	183
Jejunum	Alfalfa hay	Negative	homing	4.77E-08	197
Jejunum	Alfalfa hay	Negative	cell movement of leukocytes	4.88E-08	255
Jejunum	Alfalfa hay	Negative	digestive system cancer	5.27E-08	2063
Jejunum	Alfalfa hay	Negative	Organ Degeneration	5.44E-08	172
Jejunum	Alfalfa hay	Negative	formation of actin stress fibers	6.05E-08	92
Jejunum	Alfalfa hay	Negative	phosphorylation of protein	6.36E-08	245
Jejunum	Alfalfa hay	Negative	cell death of connective tissue cells	6.83E-08	205
Jejunum	Alfalfa hay	Negative	apoptosis of tumor cell lines	8.24E-08	382
Jejunum	Alfalfa hay	Negative	chemotaxis	8.48E-08	187
Jejunum	Alfalfa hay	Negative	Huntington's Disease	8.49E-08	196
Jejunum	Alfalfa hay	Negative	metabolism of carbohydrate	8.71E-08	210
Jejunum	Alfalfa hay	Negative	abnormal morphology of body cavity	9.59E-08	362
Jejunum	Alfalfa hay	Negative	migration of tumor cell lines	1.21E-07	235
Jejunum	Alfalfa	Negative	development of endothelial tissue	1.21E-07	131

	hay				
Jejunum	Alfalfa hay	Negative	development of body trunk	1.30E-07	354
Jejunum	Alfalfa hay	Negative	endothelial cell development	1.31E-07	127
Jejunum	Alfalfa hay	Negative	invasion of tumor cell lines	1.62E-07	222
Jejunum	Alfalfa hay	Negative	morphology of cardiovascular system	1.88E-07	208
Jejunum	Alfalfa hay	Negative	engulfment of cells	1.97E-07	143
Jejunum	Alfalfa hay	Negative	inflammatory response	2.21E-07	255
Jejunum	Alfalfa hay	Negative	seizure disorder	2.45E-07	162
Jejunum	Alfalfa hay	Negative	development of epithelial tissue	2.91E-07	176
Jejunum	Alfalfa hay	Negative	endocytosis	2.97E-07	123
Jejunum	Alfalfa hay	Negative	secretory pathway	3.58E-07	76
Jejunum	Alfalfa hay	Negative	cell cycle progression	4.38E-07	331
Jejunum	Alfalfa hay	Negative	abnormal morphology of thoracic cavity	5.22E-07	196
Jejunum	Alfalfa hay	Negative	exocytosis	7.02E-07	73
Jejunum	Alfalfa hay	Negative	uptake of monosaccharide	7.15E-07	98
Jejunum	Alfalfa hay	Negative	development of vasculature	8.95E-07	144
Jejunum	Alfalfa hay	Negative	seizures	9.16E-07	138
Jejunum	Alfalfa hay	Negative	quantity of mononuclear leukocytes	9.17E-07	239
Jejunum	Alfalfa hay	Negative	activation of DNA endogenous promoter	9.76E-07	375
Jejunum	Alfalfa hay	Negative	degeneration of nervous system	1.06E-06	103
Jejunum	Alfalfa hay	Negative	growth of organism	1.09E-06	268
Jejunum	Alfalfa hay	Negative	development of connective tissue	1.09E-06	129
Jejunum	Alfalfa	Negative	necrosis of epithelial tissue	1.12E-06	195

	hay				
Jejunum	Alfalfa hay	Negative	behavior	1.14E-06	312
Jejunum	Alfalfa hay	Negative	long-term potentiation	1.43E-06	89
Jejunum	Alfalfa hay	Negative	proliferation of blood cells	1.51E-06	269
Jejunum	Alfalfa hay	Negative	ion homeostasis of cells	2.15E-06	184
Jejunum	Alfalfa hay	Negative	invasion of carcinoma cell lines	2.28E-06	74
Jejunum	Alfalfa hay	Negative	uptake of carbohydrate	2.28E-06	103
Jejunum	Alfalfa hay	Negative	learning	2.34E-06	142
Jejunum	Alfalfa hay	Negative	cognition	2.40E-06	153
Jejunum	Alfalfa hay	Negative	function of leukocytes	2.40E-06	178
Jejunum	Alfalfa hay	Negative	perinatal death	2.45E-06	195
Jejunum	Alfalfa hay	Negative	interphase	2.84E-06	213
Jejunum	Alfalfa hay	Negative	quantity of lymphocytes	2.90E-06	228
Jejunum	Alfalfa hay	Negative	cell movement of fibroblasts	2.90E-06	69
Jejunum	Alfalfa hay	Negative	hypersensitive reaction	2.95E-06	145
Jejunum	Alfalfa hay	Negative	formation of plasma membrane projections	3.23E-06	195
Jejunum	Alfalfa hay	Negative	neuritogenesis	3.24E-06	191
Jejunum	Alfalfa hay	Negative	cell movement of connective tissue cells	3.76E-06	81
Jejunum	Alfalfa hay	Negative	cell death of blood cells	4.18E-06	223
Jejunum	Alfalfa hay	Negative	activation of cells	4.50E-06	305
Jejunum	Alfalfa hay	Negative	quantity of metal	4.50E-06	165
Jejunum	Alfalfa hay	Negative	morphology of heart	4.55E-06	137
Jejunum	Alfalfa	Negative	organization of actin cytoskeleton	4.65E-06	105

	hay				
Jejunum	Alfalfa hay	Negative	proliferation of neuronal cells	4.86E-06	188
Jejunum	Alfalfa hay	Negative	reorganization of cytoskeleton	4.91E-06	71
Jejunum	Alfalfa hay	Negative	chemotaxis of phagocytes	5.17E-06	103
Jejunum	Alfalfa hay	Negative	degeneration of cells	5.30E-06	118
Jejunum	Alfalfa hay	Negative	proliferation of immune cells	5.48E-06	251
Jejunum	Alfalfa hay	Negative	cell death of epithelial cells	5.78E-06	164
Jejunum	Alfalfa hay	Negative	abnormal morphology of cardiovascular system	5.96E-06	185
Jejunum	Alfalfa hay	Negative	cell movement of carcinoma cell lines	6.29E-06	75
Jejunum	Alfalfa hay	Negative	Growth Failure	6.37E-06	194
Jejunum	Alfalfa hay	Negative	cell death of immune cells	6.37E-06	212
Jejunum	Alfalfa hay	Negative	differentiation of connective tissue cells	6.45E-06	207
Jejunum	Alfalfa hay	Negative	uterine serous papillary cancer	6.45E-06	83
Jejunum	Alfalfa hay	Negative	synthesis of carbohydrate	6.81E-06	142
Jejunum	Alfalfa hay	Negative	migration of connective tissue cells	7.52E-06	67
Jejunum	Alfalfa hay	Negative	function of blood cells	7.72E-06	190
Jejunum	Alfalfa hay	Negative	colony formation	8.31E-06	175
Jejunum	Alfalfa hay	Negative	cell movement of granulocytes	8.76E-06	125
Jejunum	Alfalfa hay	Negative	proliferation of endothelial cells	8.99E-06	107
Jejunum	Alfalfa hay	Negative	cell death of fibroblast cell lines	9.48E-06	142
Jejunum	Alfalfa hay	Negative	chemotaxis of myeloid cells	9.80E-06	101
Jejunum	Alfalfa hay	Negative	differentiation of connective tissue	1.14E-05	231
Jejunum	Alfalfa	Negative	colony formation of cells	1.16E-05	161

	hay				
Jejunum	Alfalfa hay	Negative	metabolism of nucleotide	1.18E-05	145
Jejunum	Alfalfa hay	Negative	morphology of head	1.18E-05	273
Jejunum	Alfalfa hay	Negative	influx of Ca2+	1.28E-05	68
Jejunum	Alfalfa hay	Negative	cell movement of neutrophils	1.40E-05	102
Jejunum	Alfalfa hay	Negative	influx of cation	1.46E-05	70
Jejunum	Alfalfa hay	Negative	long-term potentiation of synapse	1.49E-05	56
Jejunum	Alfalfa hay	Negative	plasticity of synapse	1.53E-05	47
Jejunum	Alfalfa hay	Negative	dephosphorylation of protein	1.53E-05	68
Jejunum	Alfalfa hay	Negative	quantity of connective tissue	1.64E-05	193
Jejunum	Alfalfa hay	Negative	cell movement of macrophages	1.90E-05	92
Jejunum	Alfalfa hay	Negative	fatty acid metabolism	1.93E-05	192
Jejunum	Alfalfa hay	Negative	Neurodegeneration	1.95E-05	105
Jejunum	Alfalfa hay	Negative	proliferation of mononuclear leukocytes	1.95E-05	232
Jejunum	Alfalfa hay	Negative	accumulation of cells	1.97E-05	118
Jejunum	Alfalfa hay	Negative	female genital tract serous cancer	1.99E-05	128
Jejunum	Alfalfa hay	Negative	growth of lesion	2.03E-05	249
Jejunum	Alfalfa hay	Negative	cell movement of antigen presenting cells	2.08E-05	117
Jejunum	Alfalfa hay	Negative	influx of inorganic cation	2.08E-05	69
Jejunum	Alfalfa hay	Negative	quantity of metal ion	2.15E-05	145
Jejunum	Alfalfa hay	Negative	branching of cells	2.20E-05	123
Jejunum	Alfalfa hay	Negative	synthesis of nucleotide	2.24E-05	121
Jejunum	Alfalfa	Negative	secretion of molecule	2.31E-05	177

	hay				
Jejunum	Alfalfa hay	Negative	binding of DNA	2.32E-05	168
Jejunum	Alfalfa hay	Negative	formation of skin	2.32E-05	122
Jejunum	Alfalfa hay	Negative	activation of antigen presenting cells	2.41E-05	107
Jejunum	Alfalfa hay	Negative	growth of tumor	2.45E-05	248
Jejunum	Alfalfa hay	Negative	proliferation of lymphocytes	2.49E-05	228
Jejunum	Alfalfa hay	Negative	Dermatitis	2.60E-05	142
Jejunum	Alfalfa hay	Negative	metabolism of membrane lipid derivative	2.67E-05	128
Jejunum	Alfalfa hay	Negative	killing of macrophages	2.76E-05	12
Jejunum	Alfalfa hay	Negative	concentration of acylglycerol	2.81E-05	112
Jejunum	Alfalfa hay	Negative	growth of epithelial tissue	2.87E-05	214
Jejunum	Alfalfa hay	Negative	allergy	2.87E-05	133
Jejunum	Alfalfa hay	Negative	flux of ion	3.25E-05	108
Jejunum	Alfalfa hay	Negative	transport of carbohydrate	3.39E-05	69
Jejunum	Alfalfa hay	Negative	morphology of lymphatic system component	3.58E-05	136
Jejunum	Alfalfa hay	Negative	demyelination	3.64E-05	46
Jejunum	Alfalfa hay	Negative	differentiation of muscle cell lines	3.75E-05	59
Jejunum	Alfalfa hay	Negative	abnormal morphology of heart	3.80E-05	126
Jejunum	Alfalfa hay	Negative	migration of fibroblasts	3.97E-05	53
Jejunum	Alfalfa hay	Negative	cell movement of endothelial cells	4.04E-05	117
Jejunum	Alfalfa hay	Negative	quantity of Ca2+	4.42E-05	133
Jejunum	Alfalfa hay	Negative	autophagy	4.71E-05	123
Jejunum	Alfalfa	Negative	formation of cells	4.81E-05	299

	hay				
Jejunum	Alfalfa hay	Negative	organization of filaments	4.86E-05	72
Jejunum	Alfalfa hay	Negative	growth of lymphatic system component	4.86E-05	68
Jejunum	Alfalfa hay	Negative	morphology of heart ventricle	4.86E-05	68
Jejunum	Alfalfa hay	Negative	transport of protein	5.06E-05	101
Jejunum	Alfalfa hay	Negative	morphology of muscle	5.38E-05	111
Jejunum	Alfalfa hay	Negative	proliferation of smooth muscle cells	5.38E-05	96
Jejunum	Alfalfa hay	Negative	permeability of cells	5.41E-05	39
Jejunum	Alfalfa hay	Negative	abnormal morphology of head	5.54E-05	256
Jejunum	Alfalfa hay	Negative	quantity of myeloid cells	5.59E-05	117
Jejunum	Alfalfa hay	Negative	dysmyelination	5.92E-05	50
Jejunum	Alfalfa hay	Negative	flux of cation	6.01E-05	102
Jejunum	Alfalfa hay	Negative	accumulation of blood cells	6.02E-05	94
Jejunum	Alfalfa hay	Negative	growth of muscle tissue	6.09E-05	122
Jejunum	Alfalfa hay	Negative	synthesis of DNA	6.21E-05	135
Jejunum	Alfalfa hay	Negative	metabolism of nucleic acid component or derivative	6.24E-05	165
Jejunum	Alfalfa hay	Negative	transport of alpha-amino acid	6.36E-05	30
Jejunum	Alfalfa hay	Negative	abnormal morphology of abdomen	6.44E-05	268
Jejunum	Alfalfa hay	Negative	cell movement of muscle cells	6.46E-05	64
Jejunum	Alfalfa hay	Negative	quantity of phagocytes	6.48E-05	137
Jejunum	Alfalfa hay	Negative	ubiquitination	6.53E-05	111
Jejunum	Alfalfa hay	Negative	development of neurons	6.70E-05	241
Jejunum	Alfalfa	Negative	quantity of lymphatic system component	6.88E-05	123

	hay				
Jejunum	Alfalfa hay	Negative	activation of leukocytes	6.96E-05	217
Jejunum	Alfalfa hay	Negative	hydrolysis of lipid	6.99E-05	66
Jejunum	Alfalfa hay	Negative	concentration of phosphatidic acid	6.99E-05	50
Jejunum	Alfalfa hay	Negative	proliferation of muscle cells	7.01E-05	121
Jejunum	Alfalfa hay	Negative	cellular infiltration by macrophages	7.78E-05	57
Jejunum	Alfalfa hay	Negative	transport of monosaccharide	7.78E-05	57
Jejunum	Alfalfa hay	Negative	cell death of brain	7.97E-05	97
Jejunum	Alfalfa hay	Negative	flux of Ca2+	7.97E-05	95
Jejunum	Alfalfa hay	Negative	benign neoplasia	8.18E-05	281
Jejunum	Alfalfa hay	Negative	ubiquitination of protein	8.22E-05	109
Jejunum	Alfalfa hay	Negative	receptor-mediated endocytosis	8.26E-05	53
Jejunum	Alfalfa hay	Negative	flux of inorganic cation	8.26E-05	100
Jejunum	Alfalfa hay	Negative	homing of blood cells	8.26E-05	123
Jejunum	Alfalfa hay	Negative	export of molecule	8.27E-05	98
Jejunum	Alfalfa hay	Negative	neurotransmission	8.27E-05	133
Jejunum	Alfalfa hay	Negative	release of fatty acid	8.31E-05	60
Jejunum	Alfalfa hay	Negative	transport of cation	8.31E-05	130
Jejunum	Alfalfa hay	Negative	sprouting	8.73E-05	125
Jejunum	Alfalfa hay	Negative	adhesion of immune cells	8.91E-05	114
Jejunum	Alfalfa hay	Negative	development of body axis	9.12E-05	316
Jejunum	Alfalfa hay	Negative	degeneration of neurons	9.30E-05	81
Jejunum	Alfalfa	Negative	cell death of epithelial cell lines	9.30E-05	94

	hay				
Jejunum	Alfalfa hay	Negative	experimentally-induced diabetes	9.55E-05	37
Jejunum	Alfalfa hay	Negative	cell death of hippocampal neurons	9.91E-05	34
Jejunum	Alfalfa hay	Negative	concentration of phospholipid	1.01E-04	64
Jejunum	Alfalfa hay	Negative	metastasis of cells	1.02E-04	84
Jejunum	Alfalfa hay	Negative	morphology of nervous system	1.03E-04	240
Jejunum	Alfalfa hay	Negative	cellular infiltration by leukocytes	1.06E-04	125
Jejunum	Alfalfa hay	Negative	transmigration of cells	1.10E-04	56
Jejunum	Alfalfa hay	Negative	cognitive impairment	1.20E-04	108
Jejunum	Alfalfa hay	Negative	metabolism of protein	1.20E-04	293
Jejunum	Alfalfa hay	Negative	epileptic seizure	1.20E-04	59
Jejunum	Alfalfa hay	Negative	epilepsy	1.20E-04	101
Jejunum	Alfalfa hay	Negative	release of eicosanoid	1.20E-04	51
Jejunum	Alfalfa hay	Negative	cell death of hippocampal cells	1.22E-04	37
Jejunum	Alfalfa hay	Negative	hydrolysis of phosphoinositide	1.23E-04	25
Jejunum	Alfalfa hay	Negative	infection by RNA virus	1.23E-04	263
Jejunum	Alfalfa hay	Negative	cellular degradation	1.23E-04	85
Jejunum	Alfalfa hay	Negative	morphology of bone	1.23E-04	143
Jejunum	Alfalfa hay	Negative	cell death of lymphocytes	1.23E-04	124
Jejunum	Alfalfa hay	Negative	abnormal morphology of bone	1.23E-04	139
Jejunum	Alfalfa hay	Negative	apoptosis of fibroblast cell lines	1.23E-04	107
Jejunum	Alfalfa hay	Negative	migration of endothelial cells	1.23E-04	107
Jejunum	Alfalfa	Negative	activation of blood cells	1.23E-04	229

	hay				
Jejunum	Alfalfa hay	Negative	uptake of D-glucose	1.23E-04	75
Jejunum	Alfalfa hay	Negative	cell death of heart	1.28E-04	77
Jejunum	Alfalfa hay	Negative	cell death of T lymphocytes	1.28E-04	103
Jejunum	Alfalfa hay	Negative	transport of ion	1.33E-04	159
Jejunum	Alfalfa hay	Negative	cancer of cells	1.37E-04	85
Jejunum	Alfalfa hay	Negative	apoptosis of blood cells	1.38E-04	156
Jejunum	Alfalfa hay	Negative	migration of muscle cells	1.41E-04	58
Jejunum	Alfalfa hay	Negative	differentiation of bone cells	1.42E-04	130
Jejunum	Alfalfa hay	Negative	cell death of central nervous system cells	1.43E-04	96
Jejunum	Alfalfa hay	Negative	replication of RNA virus	1.43E-04	159
Jejunum	Alfalfa hay	Negative	cell death of mononuclear leukocytes	1.43E-04	127
Jejunum	Alfalfa hay	Negative	formation of muscle	1.43E-04	127
Jejunum	Alfalfa hay	Negative	homing of leukocytes	1.47E-04	121
Jejunum	Alfalfa hay	Negative	abnormal morphology of heart ventricle	1.48E-04	61
Jejunum	Alfalfa hay	Negative	release of neurotransmitter	1.52E-04	55
Jejunum	Alfalfa hay	Negative	transport of metal	1.56E-04	108
Jejunum	Alfalfa hay	Negative	arthropathy	1.70E-04	286
Jejunum	Alfalfa hay	Negative	development of head	1.70E-04	294
Jejunum	Alfalfa hay	Negative	infection by Retroviridae	1.70E-04	220
Jejunum	Alfalfa hay	Negative	HIV infection	1.70E-04	218
Jejunum	Alfalfa hay	Negative	chemotaxis of leukocytes	1.75E-04	114
Jejunum	Alfalfa	Negative	morphology of lymphoid organ	1.81E-04	125

	hay				
Jejunum	Alfalfa hay	Negative	syndromic mental retardation	1.81E-04	55
Jejunum	Alfalfa hay	Negative	morphology of connective tissue	1.93E-04	145
Jejunum	Alfalfa hay	Negative	cell death of heart cells	1.96E-04	74
Jejunum	Alfalfa hay	Negative	arthritis	1.96E-04	281
Jejunum	Alfalfa hay	Negative	abnormal morphology of nervous system	2.09E-04	221
Jejunum	Alfalfa hay	Negative	transport of inorganic cation	2.09E-04	113
Jejunum	Alfalfa hay	Negative	S phase	2.10E-04	86
Jejunum	Alfalfa hay	Negative	neutropenia	2.10E-04	19
Jejunum	Alfalfa hay	Negative	apoptosis of epithelial cell lines	2.10E-04	75
Jejunum	Alfalfa hay	Negative	serous neoplasm	2.10E-04	188
Jejunum	Alfalfa hay	Negative	I-kappaB kinase/NF-kappaB cascade	2.10E-04	60
Jejunum	Alfalfa hay	Negative	production of reactive oxygen species	2.11E-04	117
Jejunum	Alfalfa hay	Negative	concentration of eicosanoid	2.11E-04	42
Jejunum	Alfalfa hay	Negative	quantity of B lymphocytes	2.11E-04	111
Jejunum	Alfalfa hay	Negative	concentration of Ca2+	2.11E-04	46
Jejunum	Alfalfa hay	Negative	small GTPase mediated signal transduction	2.14E-04	63
Jejunum	Alfalfa hay	Negative	release of arachidonic acid	2.14E-04	35
Jejunum	Alfalfa hay	Negative	mitosis	2.17E-04	155
Jejunum	Alfalfa hay	Negative	cell viability of cervical cancer cell lines	2.17E-04	74
Jejunum	Alfalfa hay	Negative	synthesis of fatty acid	2.18E-04	101
Jejunum	Alfalfa hay	Negative	proliferation of fibroblasts	2.23E-04	113
Jejunum	Alfalfa	Negative	mass of connective tissue	2.24E-04	68

	hay				
Jejunum	Alfalfa hay	Negative	proliferation of lymphatic system cells	2.24E-04	68
Jejunum	Alfalfa hay	Negative	accumulation of lipid	2.26E-04	97
Jejunum	Alfalfa hay	Negative	development of blood cells	2.28E-04	200
Jejunum	Alfalfa hay	Negative	development of lymphatic system component	2.31E-04	100
Jejunum	Alfalfa hay	Negative	proliferation of T lymphocytes	2.34E-04	186
Jejunum	Alfalfa hay	Negative	homeostasis of ion	2.40E-04	79
Jejunum	Alfalfa hay	Negative	quantity of steroid	2.43E-04	146
Jejunum	Alfalfa hay	Negative	binding of endothelial cells	2.43E-04	39
Jejunum	Alfalfa hay	Negative	infection of cells	2.46E-04	241
Jejunum	Alfalfa hay	Negative	degeneration of central nervous system	2.47E-04	47
Jejunum	Alfalfa hay	Negative	apoptosis of neurons	2.47E-04	137
Jejunum	Alfalfa hay	Negative	maturation of cells	2.51E-04	143
Jejunum	Alfalfa hay	Negative	growth of embryo	2.51E-04	149
Jejunum	Alfalfa hay	Negative	quantity of hematopoietic cells	2.55E-04	139
Jejunum	Alfalfa hay	Negative	experimentally-induced arthritis	2.57E-04	53
Jejunum	Alfalfa hay	Negative	neurodegeneration of cerebral cortex	2.58E-04	24
Jejunum	Alfalfa hay	Negative	proliferation of glomerular cells	2.58E-04	24
Jejunum	Alfalfa hay	Negative	proliferation of mesangial cells	2.59E-04	21
Jejunum	Alfalfa hay	Negative	size of animal	2.62E-04	61
Jejunum	Alfalfa hay	Negative	cell death of brain cells	2.63E-04	89
Jejunum	Alfalfa hay	Negative	homeostasis of inorganic cation	2.65E-04	64
Jejunum	Alfalfa	Negative	apoptosis of leukocytes	2.68E-04	143

	hay				
Jejunum	Alfalfa hay	Negative	synaptic transmission	2.74E-04	108
Jejunum	Alfalfa hay	Negative	development of leukocytes	2.77E-04	181
Jejunum	Alfalfa hay	Negative	cell death of cerebral cortex cells	2.77E-04	76
Jejunum	Alfalfa hay	Negative	formation of leukocytes	2.81E-04	55
Jejunum	Alfalfa hay	Negative	activation of neuroglia	2.92E-04	47
Jejunum	Alfalfa hay	Negative	proliferation of fibroblast cell lines	2.93E-04	133
Jejunum	Alfalfa hay	Negative	modification of peptide	2.95E-04	39
Jejunum	Alfalfa hay	Negative	abnormal morphology of embryonic tissue	2.99E-04	169
Jejunum	Alfalfa hay	Negative	muscle contraction	2.99E-04	81
Jejunum	Alfalfa hay	Negative	quantity of bone	2.99E-04	81
Jejunum	Alfalfa hay	Negative	metabolism of monosaccharide	2.99E-04	44
Jejunum	Alfalfa hay	Negative	activation of phagocytes	2.99E-04	118
Jejunum	Alfalfa hay	Negative	cellular infiltration	3.12E-04	137
Jejunum	Alfalfa hay	Negative	neovascularization	3.16E-04	56
Jejunum	Alfalfa hay	Negative	quantity of hematopoietic progenitor cells	3.16E-04	138
Jejunum	Alfalfa hay	Negative	Bleeding	3.16E-04	133
Jejunum	Alfalfa hay	Negative	aggregation of cells	3.19E-04	104
Jejunum	Alfalfa hay	Negative	function of cardiovascular system	3.19E-04	117
Jejunum	Alfalfa hay	Negative	accumulation of leukocytes	3.29E-04	87
Jejunum	Alfalfa hay	Negative	quantity of T lymphocytes	3.35E-04	165
Jejunum	Alfalfa hay	Negative	growth of renal glomerulus	3.36E-04	28
Jejunum	Alfalfa	Negative	cleavage of lipid	3.43E-04	67

	hay				
Jejunum	Alfalfa hay	Negative	autophagy of cells	3.50E-04	86
Jejunum	Alfalfa hay	Negative	transport of L-amino acid	3.52E-04	26
Jejunum	Alfalfa hay	Negative	excitatory postsynaptic potential	3.60E-04	44
Jejunum	Alfalfa hay	Negative	transport of metal ion	3.64E-04	102
Jejunum	Alfalfa hay	Negative	differentiation of muscle	3.72E-04	97
Jejunum	Alfalfa hay	Negative	development of lymphocytes	3.72E-04	170
Jejunum	Alfalfa hay	Negative	advanced malignant tumor	3.82E-04	253
Jejunum	Alfalfa hay	Negative	chronic psoriasis	3.82E-04	50
Jejunum	Alfalfa hay	Negative	concentration of sterol	3.85E-04	98
Jejunum	Alfalfa hay	Negative	quantity of granulocytes	3.88E-04	101
Jejunum	Alfalfa hay	Negative	intracranial hemorrhage	3.91E-04	49
Jejunum	Alfalfa hay	Negative	migration of carcinoma cell lines	3.93E-04	58
Jejunum	Alfalfa hay	Negative	cell transformation	3.96E-04	143
Jejunum	Alfalfa hay	Negative	apoptosis of kidney cell lines	3.97E-04	78
Jejunum	Alfalfa hay	Negative	development of mononuclear leukocytes	3.97E-04	171
Jejunum	Alfalfa hay	Negative	entrance of Ca2+	3.97E-04	30
Jejunum	Alfalfa hay	Negative	abnormal morphology of lymphoid organ	3.97E-04	119
Jejunum	Alfalfa hay	Negative	replication of virus	3.97E-04	172
Jejunum	Alfalfa hay	Negative	cell death of tumor	4.05E-04	120
Jejunum	Alfalfa hay	Negative	oxidative stress response of cells	4.05E-04	25
Jejunum	Alfalfa hay	Negative	concentration of triacylglycerol	4.22E-04	98
Jejunum	Alfalfa	Negative	proliferation of leukemia cell lines	4.30E-04	70

	hay				
Jejunum	Alfalfa hay	Negative	abnormal quantity of lipid	4.32E-04	51
Jejunum	Alfalfa hay	Negative	homeostasis of metal ion	4.33E-04	62
Jejunum	Alfalfa hay	Negative	development of abdomen	4.34E-04	177
Jejunum	Alfalfa hay	Negative	neurodegeneration of brain	4.34E-04	40
Jejunum	Alfalfa hay	Negative	inflammation of organ	4.40E-04	344
Jejunum	Alfalfa hay	Negative	serine phosphorylation of peptide	4.48E-04	32
Jejunum	Alfalfa hay	Negative	cell movement of mononuclear leukocytes	4.59E-04	143
Jejunum	Alfalfa hay	Negative	growth of neurites	4.59E-04	143
Jejunum	Alfalfa hay	Negative	phagocytosis	4.59E-04	95
Jejunum	Alfalfa hay	Negative	vascularization	4.70E-04	77
Jejunum	Alfalfa hay	Negative	consumption of oxygen	4.70E-04	53
Jejunum	Alfalfa hay	Negative	concentration of cholesterol	4.70E-04	93
Jejunum	Alfalfa hay	Negative	assembly of protein-protein complex	4.70E-04	63
Jejunum	Alfalfa hay	Negative	morphology of respiratory system	4.78E-04	99
Jejunum	Alfalfa hay	Negative	abnormal morphology of skull	4.90E-04	72
Jejunum	Alfalfa hay	Negative	differentiation of epithelial tissue	4.90E-04	113
Jejunum	Alfalfa hay	Negative	branching of neurites	4.90E-04	89
Jejunum	Alfalfa hay	Negative	serine phosphorylation	4.96E-04	35
Jejunum	Alfalfa hay	Negative	cell death of kidney cells	4.96E-04	110
Jejunum	Alfalfa hay	Negative	growth of plasma membrane projections	4.96E-04	144
Jejunum	Alfalfa hay	Negative	cell viability of blood cells	4.96E-04	92
Jejunum	Alfalfa	Negative	adhesion of blood cells	4.98E-04	118

	hay				
Jejunum	Alfalfa hay	Negative	degeneration of brain	5.01E-04	44
Jejunum	Alfalfa hay	Negative	necrosis of tumor	5.07E-04	119
Jejunum	Alfalfa hay	Negative	peripheral vascular disease	5.07E-04	119
Jejunum	Alfalfa hay	Negative	abnormal morphology of right ventricle	5.09E-04	21
Jejunum	Alfalfa hay	Negative	homeostasis of divalent cations	5.11E-04	50
Jejunum	Alfalfa hay	Negative	proliferation of hematopoietic cell lines	5.15E-04	73
Jejunum	Alfalfa hay	Negative	development of lymphatic system	5.15E-04	112
Jejunum	Alfalfa hay	Negative	neonatal death	5.21E-04	136
Jejunum	Alfalfa hay	Negative	mass of adipose tissue	5.30E-04	63
Jejunum	Alfalfa hay	Negative	morphology of right ventricle	5.37E-04	23
Jejunum	Alfalfa hay	Negative	phosphorylation of L-amino acid	5.45E-04	72
Jejunum	Alfalfa hay	Negative	cell movement of lymphocytes	5.45E-04	123
Jejunum	Alfalfa hay	Negative	mobilization of cells	5.54E-04	32
Jejunum	Alfalfa hay	Negative	vascularization of body region	5.55E-04	47
Jejunum	Alfalfa hay	Negative	synthesis of phospholipid	5.58E-04	59
Jejunum	Alfalfa hay	Negative	homeostasis of leukocytes	5.86E-04	169
Jejunum	Alfalfa hay	Negative	necrosis of kidney	5.93E-04	114
Jejunum	Alfalfa hay	Negative	autophosphorylation of protein	6.06E-04	54
Jejunum	Alfalfa hay	Negative	quantity of polyunsaturated fatty acids	6.07E-04	43
Jejunum	Alfalfa hay	Negative	morphology of skin	6.11E-04	87
Jejunum	Alfalfa hay	Negative	metastasis	6.28E-04	227
Jejunum	Alfalfa	Negative	cell death of kidney cell lines	6.35E-04	96

	hay				
Jejunum	Alfalfa hay	Negative	muscular hypertrophy	6.39E-04	67
Jejunum	Alfalfa hay	Negative	activation of Protein kinase	6.43E-04	81
Jejunum	Alfalfa hay	Negative	cellular infiltration of phagocytes	6.46E-04	59
Jejunum	Alfalfa hay	Negative	branching of neurons	6.46E-04	91
Jejunum	Alfalfa hay	Negative	necrosis of cardiac muscle	6.50E-04	71
Jejunum	Alfalfa hay	Negative	activation of macrophages	6.50E-04	77
Jejunum	Alfalfa hay	Negative	abnormal morphology of respiratory system	6.50E-04	97
Jejunum	Alfalfa hay	Negative	autosomal recessive disease	6.50E-04	272
Jejunum	Alfalfa hay	Negative	synthesis of purine nucleotide	6.75E-04	58
Jejunum	Alfalfa hay	Negative	recruitment of leukocytes	6.92E-04	102
Jejunum	Alfalfa hay	Negative	quantity of antigen presenting cells	7.08E-04	88
Jejunum	Alfalfa hay	Negative	exocytosis by cells	7.09E-04	44
Jejunum	Alfalfa hay	Negative	cell viability of mononuclear leukocytes	7.09E-04	65
Jejunum	Alfalfa hay	Negative	outgrowth of neurites	7.27E-04	125
Jejunum	Alfalfa hay	Negative	transport of D-glucose	7.27E-04	49
Jejunum	Alfalfa hay	Negative	morphogenesis of neurons	7.29E-04	131
Jejunum	Alfalfa hay	Negative	migration of mesenchymal cells	7.61E-04	17
Jejunum	Alfalfa hay	Negative	chemotaxis of antigen presenting cells	7.61E-04	52
Jejunum	Alfalfa hay	Negative	chronic myeloid leukemia	7.62E-04	38
Jejunum	Alfalfa hay	Negative	exocytosis of granules	7.62E-04	13
Jejunum	Alfalfa hay	Negative	internalization of lipid	7.62E-04	13
Jejunum	Alfalfa	Negative	necrosis of muscle	7.90E-04	107

	hay				
Jejunum	Alfalfa hay	Negative	size of embryo	7.90E-04	111
Jejunum	Alfalfa hay	Negative	hypertrophy of cardiomyocytes	7.96E-04	46
Jejunum	Alfalfa hay	Negative	transport of amino acids	7.96E-04	46
Jejunum	Alfalfa hay	Negative	cell movement of smooth muscle cells	8.09E-04	54
Jejunum	Alfalfa hay	Negative	hydrolysis of carbohydrate	8.19E-04	45
Jejunum	Alfalfa hay	Negative	T cell development	8.27E-04	153
Jejunum	Alfalfa hay	Negative	neoplasia of cells	8.36E-04	136
Jejunum	Alfalfa hay	Negative	miniature excitatory postsynaptic currents	8.36E-04	24
Jejunum	Alfalfa hay	Negative	relaxation of muscle	8.38E-04	31
Jejunum	Alfalfa hay	Negative	shape change of neurites	8.40E-04	90
Jejunum	Alfalfa hay	Negative	transport of synaptic vesicles	8.53E-04	27
Jejunum	Alfalfa hay	Negative	activation of enzyme	8.55E-04	120
Jejunum	Alfalfa hay	Negative	immediate hypersensitivity	8.61E-04	100
Jejunum	Alfalfa hay	Negative	synthesis of progesterone	8.63E-04	32
Jejunum	Alfalfa hay	Negative	Lymphocyte homeostasis	8.63E-04	165
Jejunum	Alfalfa hay	Negative	outgrowth of cells	8.66E-04	133
Jejunum	Alfalfa hay	Negative	keratosis	8.67E-04	42
Jejunum	Alfalfa hay	Negative	abnormal morphology of skeleton	8.78E-04	86
Jejunum	Alfalfa hay	Negative	phagocytosis of cells	8.78E-04	86
Jejunum	Alfalfa hay	Negative	differentiation of muscle cells	8.89E-04	89
Jejunum	Alfalfa hay	Negative	binding of mononuclear leukocytes	8.94E-04	40
Jejunum	Alfalfa	Negative	binding of lymphocytes	8.95E-04	34

	hay				
Jejunum	Alfalfa hay	Negative	uptake of fatty acid	9.04E-04	28
Jejunum	Alfalfa hay	Negative	mental retardation	9.07E-04	75
Jejunum	Alfalfa hay	Negative	metabolism of phospholipid	9.07E-04	75
Jejunum	Alfalfa hay	Negative	response of mononuclear leukocytes	9.07E-04	75
Jejunum	Alfalfa hay	Negative	cell death of tumor cells	9.14E-04	115
Jejunum	Alfalfa hay	Negative	cellularity	9.15E-04	25
Jejunum	Alfalfa hay	Negative	apoptosis of heart cells	9.26E-04	62
Jejunum	Alfalfa hay	Negative	tyrosine phosphorylation of protein	9.26E-04	62
Jejunum	Alfalfa hay	Negative	function of muscle	9.43E-04	108
Jejunum	Alfalfa hay	Negative	transmigration of blood cells	9.78E-04	44
Jejunum	Alfalfa hay	Negative	mobilization of Ca2+	1.02E-03	107
Jejunum	Alfalfa hay	Negative	formation of filopodia	1.02E-03	55
Jejunum	Alfalfa hay	Negative	cell death of cardiomyocytes	1.04E-03	69
Jejunum	Alfalfa hay	Negative	morphology of skeleton	1.05E-03	88
Jejunum	Alfalfa hay	Negative	metabolism of acylglycerol	1.05E-03	47
Jejunum	Alfalfa hay	Negative	hydrolysis of phosphatidylinositol	1.09E-03	33
Jejunum	Alfalfa hay	Negative	degranulation of cells	1.10E-03	72
Jejunum	Alfalfa hay	Negative	contraction of heart	1.10E-03	59
Jejunum	Alfalfa hay	Negative	formation of connective tissue cells	1.10E-03	68
Jejunum	Alfalfa hay	Negative	quantity of lymphoid organ	1.11E-03	96
Jejunum	Alfalfa hay	Negative	morphogenesis of neurites	1.11E-03	128
Jejunum	Alfalfa	Negative	quantity of cellular protrusions	1.11E-03	53

	hay				
Jejunum	Alfalfa hay	Negative	synthesis of nitric oxide	1.12E-03	87
Jejunum	Alfalfa hay	Negative	contraction of striated muscle	1.12E-03	45
Jejunum	Alfalfa hay	Negative	cell viability of leukocytes	1.13E-03	82
Jejunum	Alfalfa hay	Negative	cell death of muscle cells	1.13E-03	104
Jejunum	Alfalfa hay	Negative	abnormal morphology of axial skeleton	1.14E-03	63
Jejunum	Alfalfa hay	Negative	trafficking of cells	1.15E-03	28
Jejunum	Alfalfa hay	Negative	recruitment of cells	1.18E-03	110
Jejunum	Alfalfa hay	Negative	formation of osteoclasts	1.23E-03	42
Jejunum	Alfalfa hay	Negative	differentiation of leukocytes	1.23E-03	200
Jejunum	Alfalfa hay	Negative	MAPKKK cascade	1.24E-03	66
Jejunum	Alfalfa hay	Negative	differentiation of blood cells	1.24E-03	248
Jejunum	Alfalfa hay	Negative	adhesion of tumor cell lines	1.24E-03	90
Jejunum	Alfalfa hay	Negative	autosomal dominant disease	1.27E-03	190
Jejunum	Alfalfa hay	Negative	glucose metabolism disorder	1.27E-03	369
Jejunum	Alfalfa hay	Negative	release of granules	1.28E-03	15
Jejunum	Alfalfa hay	Negative	binding of cells	1.28E-03	145
Jejunum	Alfalfa hay	Negative	development of connective tissue cells	1.30E-03	71
Jejunum	Alfalfa hay	Negative	quantity of macrophages	1.30E-03	65
Jejunum	Rice straw	Positive	malignant solid tumor	1.37E-32	3361
Jejunum	Rice straw	Positive	cancer	1.37E-32	3396
Jejunum	Rice straw	Positive	morbidity or mortality	9.66E-30	920
Jejunum	Rice	Positive	organismal death	2.85E-29	907

	straw				
Jejunum	Rice straw	Positive	proliferation of cells	2.77E-27	1302
Jejunum	Rice straw	Positive	organization of cytoplasm	5.22E-21	592
Jejunum	Rice straw	Positive	tumorigenesis of tissue	6.79E-21	2828
Jejunum	Rice straw	Positive	epithelial cancer	6.79E-21	2762
Jejunum	Rice straw	Positive	neoplasia of epithelial tissue	8.59E-21	2785
Jejunum	Rice straw	Positive	cell death	7.99E-19	1146
Jejunum	Rice straw	Positive	morphology of cells	2.51E-18	712
Jejunum	Rice straw	Positive	abdominal neoplasm	9.61E-18	2746
Jejunum	Rice straw	Positive	necrosis	4.26E-17	901
Jejunum	Rice straw	Positive	abdominal cancer	4.26E-17	2712
Jejunum	Rice straw	Positive	organization of cytoskeleton	1.64E-16	526
Jejunum	Rice straw	Positive	transport of molecule	1.09E-15	592
Jejunum	Rice straw	Positive	digestive system cancer	1.09E-15	2362
Jejunum	Rice straw	Positive	digestive organ tumor	7.92E-15	2376
Jejunum	Rice straw	Positive	vasculogenesis	1.13E-14	298
Jejunum	Rice straw	Positive	cell movement	1.14E-14	749
Jejunum	Rice straw	Positive	abnormal morphology of cells	1.71E-14	474
Jejunum	Rice straw	Positive	cellular homeostasis	5.84E-14	554
Jejunum	Rice straw	Positive	angiogenesis	6.70E-14	353
Jejunum	Rice straw	Positive	expression of RNA	7.36E-14	750
Jejunum	Rice straw	Positive	transcription	1.42E-13	701
Jejunum	Rice	Positive	apoptosis	2.24E-13	899

	straw				
Jejunum	Rice straw	Positive	Movement Disorders	4.21E-13	400
Jejunum	Rice straw	Positive	microtubule dynamics	5.54E-13	440
Jejunum	Rice straw	Positive	transcription of RNA	9.69E-13	653
Jejunum	Rice straw	Positive	differentiation of cells	3.05E-12	772
Jejunum	Rice straw	Positive	cell survival	3.69E-12	503
Jejunum	Rice straw	Positive	cell viability	2.65E-11	469
Jejunum	Rice straw	Positive	development of cytoplasm	3.02E-11	189
Jejunum	Rice straw	Positive	morphology of cardiovascular system	3.14E-11	244
Jejunum	Rice straw	Positive	disorder of basal ganglia	3.52E-11	294
Jejunum	Rice straw	Positive	neurological signs	5.06E-11	254
Jejunum	Rice straw	Positive	migration of cells	5.85E-11	656
Jejunum	Rice straw	Positive	dyskinesia	9.86E-11	241
Jejunum	Rice straw	Positive	cell death of connective tissue cells	1.06E-10	236
Jejunum	Rice straw	Positive	abnormal morphology of thoracic cavity	1.35E-10	230
Jejunum	Rice straw	Positive	formation of cellular protrusions	4.76E-10	334
Jejunum	Rice straw	Positive	quantity of cells	7.82E-10	607
Jejunum	Rice straw	Positive	chorea	1.15E-09	223
Jejunum	Rice straw	Positive	Huntington's Disease	1.33E-09	222
Jejunum	Rice straw	Positive	neuromuscular disease	2.52E-09	324
Jejunum	Rice straw	Positive	growth of organism	3.31E-09	308
Jejunum	Rice straw	Positive	size of body	7.96E-09	311
Jejunum	Rice	Positive	proliferation of tumor cell lines	1.73E-08	530

	straw				
Jejunum	Rice straw	Positive	cell death of tumor cell lines	1.73E-08	523
Jejunum	Rice straw	Positive	abnormal morphology of cardiovascular system	1.77E-08	215
Jejunum	Rice straw	Positive	concentration of lipid	3.03E-08	295
Jejunum	Rice straw	Positive	synthesis of lipid	3.22E-08	263
Jejunum	Rice straw	Positive	development of body trunk	3.41E-08	393
Jejunum	Rice straw	Positive	formation of cells	4.18E-08	350
Jejunum	Rice straw	Positive	peripheral arterial disease	4.21E-08	90
Jejunum	Rice straw	Positive	benign neoplasia	5.07E-08	331
Jejunum	Rice straw	Positive	protein kinase cascade	5.58E-08	163
Jejunum	Rice straw	Positive	Viral Infection	5.77E-08	546
Jejunum	Rice straw	Positive	transcription of DNA	6.19E-08	522
Jejunum	Rice straw	Positive	invasion of cells	7.08E-08	312
Jejunum	Rice straw	Positive	development of connective tissue	7.97E-08	145
Jejunum	Rice straw	Positive	morphology of body cavity	1.23E-07	414
Jejunum	Rice straw	Positive	adenocarcinoma	1.25E-07	2017
Jejunum	Rice straw	Positive	formation of cytoskeleton	1.26E-07	146
Jejunum	Rice straw	Positive	perinatal death	1.27E-07	220
Jejunum	Rice straw	Positive	degeneration of nervous system	1.30E-07	115
Jejunum	Rice straw	Positive	fibrogenesis	1.30E-07	158
Jejunum	Rice straw	Positive	morphology of heart	1.31E-07	156
Jejunum	Rice straw	Positive	development of vasculature	1.68E-07	160
Jejunum	Rice	Positive	congenital anomaly of musculoskeletal system	1.88E-07	266

	straw				
Jejunum	Rice straw	Positive	organization of organelle	2.77E-07	200
Jejunum	Rice straw	Positive	autosomal recessive disease	3.02E-07	325
Jejunum	Rice straw	Positive	apoptosis of tumor cell lines	3.37E-07	416
Jejunum	Rice straw	Positive	Organ Degeneration	3.60E-07	184
Jejunum	Rice straw	Positive	behavior	3.87E-07	346
Jejunum	Rice straw	Positive	formation of filaments	5.09E-07	151
Jejunum	Rice straw	Positive	small GTPase mediated signal transduction	5.35E-07	77
Jejunum	Rice straw	Positive	abdominal adenocarcinoma	5.62E-07	1834
Jejunum	Rice straw	Positive	ubiquitination	5.70E-07	130
Jejunum	Rice straw	Positive	metabolism of nucleic acid component or derivative	7.97E-07	191
Jejunum	Rice straw	Positive	breast or colorectal cancer	1.01E-06	1553
Jejunum	Rice straw	Positive	abnormal morphology of body cavity	1.13E-06	391
Jejunum	Rice straw	Positive	sprouting	1.73E-06	145
Jejunum	Rice straw	Positive	cell transformation	1.78E-06	170
Jejunum	Rice straw	Positive	cell movement of tumor cell lines	1.79E-06	298
Jejunum	Rice straw	Positive	ubiquitination of protein	2.39E-06	126
Jejunum	Rice straw	Positive	concentration of fatty acid	2.71E-06	108
Jejunum	Rice straw	Positive	neuronal cell death	2.72E-06	242
Jejunum	Rice straw	Positive	abnormal morphology of embryonic tissue	2.90E-06	198
Jejunum	Rice straw	Positive	abnormal morphology of heart	3.00E-06	143
Jejunum	Rice straw	Positive	gastrointestinal tract cancer	3.10E-06	1574
Jejunum	Rice	Positive	morphology of head	3.53E-06	304

	straw				
Jejunum	Rice straw	Positive	invasion of tumor cell lines	3.63E-06	236
Jejunum	Rice straw	Positive	apoptosis of connective tissue cells	3.71E-06	113
Jejunum	Rice straw	Positive	gastrointestinal carcinoma	3.73E-06	1416
Jejunum	Rice straw	Positive	growth of connective tissue	3.75E-06	229
Jejunum	Rice straw	Positive	metabolism of nucleoside triphosphate	4.22E-06	59
Jejunum	Rice straw	Positive	migration of tumor cell lines	4.22E-06	249
Jejunum	Rice straw	Positive	infection by Retroviridae	4.34E-06	253
Jejunum	Rice straw	Positive	metabolism of carbohydrate	4.40E-06	221
Jejunum	Rice straw	Positive	phosphorylation of protein	4.66E-06	258
Jejunum	Rice straw	Positive	cell cycle progression	4.66E-06	357
Jejunum	Rice straw	Positive	growth of epithelial tissue	4.72E-06	240
Jejunum	Rice straw	Positive	cell movement of endothelial cells	4.84E-06	132
Jejunum	Rice straw	Positive	abnormal morphology of head	4.98E-06	289
Jejunum	Rice straw	Positive	quantity of leukocytes	5.99E-06	315
Jejunum	Rice straw	Positive	proliferation of connective tissue cells	5.99E-06	211
Jejunum	Rice straw	Positive	proliferation of fibroblast cell lines	5.99E-06	155
Jejunum	Rice straw	Positive	apoptosis of fibroblast cell lines	5.99E-06	123
Jejunum	Rice straw	Positive	Gastrointestinal Tract Cancer and Tumors	6.33E-06	1594
Jejunum	Rice straw	Positive	formation of leukocytes	7.23E-06	65
Jejunum	Rice straw	Positive	interphase	7.70E-06	231
Jejunum	Rice straw	Positive	HIV infection	8.17E-06	249
Jejunum	Rice	Positive	metabolism of protein	8.55E-06	332

	straw				
Jejunum	Rice straw	Positive	growth of embryo	8.61E-06	172
Jejunum	Rice straw	Positive	cell viability of tumor cell lines	9.37E-06	270
Jejunum	Rice straw	Positive	metabolism of nucleotide	9.70E-06	159
Jejunum	Rice straw	Positive	branching of cells	9.74E-06	136
Jejunum	Rice straw	Positive	colon tumor	1.05E-05	1169
Jejunum	Rice straw	Positive	cell death of fibroblast cell lines	1.06E-05	155
Jejunum	Rice straw	Positive	cellular degradation	1.07E-05	97
Jejunum	Rice straw	Positive	tumorigenesis of intestine	1.07E-05	1168
Jejunum	Rice straw	Positive	colon cancer	1.10E-05	1162
Jejunum	Rice straw	Positive	abnormal morphology of epithelial tissue	1.11E-05	152
Jejunum	Rice straw	Positive	neoplasia of colon	1.14E-05	1165
Jejunum	Rice straw	Positive	function of leukocytes	1.20E-05	191
Jejunum	Rice straw	Positive	function of blood cells	1.28E-05	207
Jejunum	Rice straw	Positive	abdominal carcinoma	1.29E-05	1842
Jejunum	Rice straw	Positive	apoptosis of blood cells	1.35E-05	177
Jejunum	Rice straw	Positive	death of perinatal stage organism	1.38E-05	37
Jejunum	Rice straw	Positive	Neurodegeneration	1.39E-05	115
Jejunum	Rice straw	Positive	biosynthesis of nucleoside triphosphate	1.47E-05	46
Jejunum	Rice straw	Positive	metabolism of membrane lipid derivative	1.57E-05	141
Jejunum	Rice straw	Positive	cognition	1.62E-05	163
Jejunum	Rice straw	Positive	I-kappaB kinase/NF-kappaB cascade	1.66E-05	69
Jejunum	Rice	Positive	synthesis of carbohydrate	1.90E-05	153

	straw				
Jejunum	Rice straw	Positive	transactivation	2.13E-05	203
Jejunum	Rice straw	Positive	morphology of nervous system	2.26E-05	269
Jejunum	Rice straw	Positive	cell movement of muscle cells	2.26E-05	71
Jejunum	Rice straw	Positive	colorectal carcinoma	2.28E-05	1159
Jejunum	Rice straw	Positive	learning	2.58E-05	150
Jejunum	Rice straw	Positive	growth of lesion	2.63E-05	273
Jejunum	Rice straw	Positive	cell death of fibroblasts	2.66E-05	99
Jejunum	Rice straw	Positive	movement of vascular endothelial cells	2.72E-05	71
Jejunum	Rice straw	Positive	cell movement of smooth muscle cells	2.85E-05	64
Jejunum	Rice straw	Positive	morphogenesis of neurons	3.03E-05	152
Jejunum	Rice straw	Positive	branching of neurites	3.04E-05	103
Jejunum	Rice straw	Positive	growth of tumor	3.14E-05	272
Jejunum	Rice straw	Positive	melanoma	3.24E-05	2026
Jejunum	Rice straw	Positive	accumulation of lipid	3.30E-05	110
Jejunum	Rice straw	Positive	benign neoplasm of female genital organ	3.33E-05	152
Jejunum	Rice straw	Positive	fatty acid metabolism	3.49E-05	209
Jejunum	Rice straw	Positive	infection by RNA virus	3.49E-05	294
Jejunum	Rice straw	Positive	migration of muscle cells	3.57E-05	65
Jejunum	Rice straw	Positive	morphology of muscle	3.57E-05	122
Jejunum	Rice straw	Positive	female genital neoplasm	3.57E-05	1112
Jejunum	Rice straw	Positive	quantity of blood cells	3.62E-05	344
Jejunum	Rice	Positive	neonatal death	4.05E-05	156

	straw				
Jejunum	Rice straw	Positive	intestinal tumor	4.05E-05	1351
Jejunum	Rice straw	Positive	colorectal neoplasia	4.05E-05	1347
Jejunum	Rice straw	Positive	formation of muscle	4.19E-05	142
Jejunum	Rice straw	Positive	cell death of embryonic cells	4.19E-05	48
Jejunum	Rice straw	Positive	migration of smooth muscle cells	4.60E-05	59
Jejunum	Rice straw	Positive	development of epithelial tissue	4.60E-05	181
Jejunum	Rice straw	Positive	development of head	4.61E-05	329
Jejunum	Rice straw	Positive	morphology of vessel	4.62E-05	112
Jejunum	Rice straw	Positive	outgrowth of plasma membrane projections	4.79E-05	145
Jejunum	Rice straw	Positive	colorectal cancer	5.11E-05	1328
Jejunum	Rice straw	Positive	metabolism of DNA	5.18E-05	142
Jejunum	Rice straw	Positive	peripheral vascular disease	5.37E-05	136
Jejunum	Rice straw	Positive	intermediate disease stage peripheral arterial disease	5.40E-05	62
Jejunum	Rice straw	Positive	neuritogenesis	5.55E-05	202
Jejunum	Rice straw	Positive	transmembrane potential of mitochondria	5.63E-05	81
Jejunum	Rice straw	Positive	gastrointestinal adenocarcinoma	6.01E-05	1322
Jejunum	Rice straw	Positive	degeneration of neurons	6.01E-05	89
Jejunum	Rice straw	Positive	synthesis of nucleotide	6.08E-05	130
Jejunum	Rice straw	Positive	intestinal cancer	6.13E-05	1329
Jejunum	Rice straw	Positive	cell death of blood cells	6.38E-05	237
Jejunum	Rice straw	Positive	abnormal morphology of nervous system	6.45E-05	247
Jejunum	Rice	Positive	development of cardiovascular tissue	6.45E-05	132

	straw				
Jejunum	Rice straw	Positive	synthesis of fatty acid	6.51E-05	113
Jejunum	Rice straw	Positive	genital tumor	6.76E-05	1253
Jejunum	Rice straw	Positive	female genital tract serous cancer	7.16E-05	137
Jejunum	Rice straw	Positive	cartilage development	7.22E-05	57
Jejunum	Rice straw	Positive	attachment of cells	7.22E-05	54
Jejunum	Rice straw	Positive	abnormal morphology of respiratory system	7.61E-05	111
Jejunum	Rice straw	Positive	morphology of heart ventricle	7.87E-05	73
Jejunum	Rice straw	Positive	long-term potentiation	7.92E-05	90
Jejunum	Rice straw	Positive	cell viability of connective tissue cells	8.31E-05	53
Jejunum	Rice straw	Positive	branching of neurons	8.31E-05	104
Jejunum	Rice straw	Positive	degeneration of cells	8.77E-05	123
Jejunum	Rice straw	Positive	survival of organism	9.33E-05	239
Jejunum	Rice straw	Positive	morphology of respiratory system	9.62E-05	112
Jejunum	Rice straw	Positive	cell death of stem cells	9.62E-05	36
Jejunum	Rice straw	Positive	Bleeding	9.67E-05	149
Jejunum	Rice straw	Positive	infection by HIV-1	9.84E-05	209
Jejunum	Rice straw	Positive	function of cardiovascular system	1.02E-04	131
Jejunum	Rice straw	Positive	morphogenesis of neurites	1.02E-04	147
Jejunum	Rice straw	Positive	development of body axis	1.04E-04	348
Jejunum	Rice straw	Positive	necrosis of epithelial tissue	1.06E-04	202
Jejunum	Rice straw	Positive	homing of cells	1.07E-04	192
Jejunum	Rice	Positive	biosynthesis of purine ribonucleotide	1.10E-04	42

	straw				
Jejunum	Rice straw	Positive	Growth Failure	1.12E-04	205
Jejunum	Rice straw	Positive	cell movement of neutrophils	1.16E-04	107
Jejunum	Rice straw	Positive	outgrowth of neurites	1.16E-04	142
Jejunum	Rice straw	Positive	abnormal morphology of reproductive system	1.17E-04	176
Jejunum	Rice straw	Positive	migration of granulocytes	1.23E-04	56
Jejunum	Rice straw	Positive	morphology of blood vessel	1.23E-04	102
Jejunum	Rice straw	Positive	formation of thymocytes	1.23E-04	43
Jejunum	Rice straw	Positive	formation of plasma membrane projections	1.25E-04	204
Jejunum	Rice straw	Positive	cell spreading	1.25E-04	100
Jejunum	Rice straw	Positive	formation of blood cells	1.27E-04	74
Jejunum	Rice straw	Positive	colorectal adenocarcinoma	1.31E-04	1117
Jejunum	Rice straw	Positive	morphology of connective tissue	1.34E-04	160
Jejunum	Rice straw	Positive	accumulation of steroid	1.38E-04	35
Jejunum	Rice straw	Positive	formation of actin filaments	1.40E-04	109
Jejunum	Rice straw	Positive	colony formation of cells	1.40E-04	170
Jejunum	Rice straw	Positive	transport of protein	1.42E-04	108
Jejunum	Rice straw	Positive	differentiation of connective tissue	1.44E-04	246
Jejunum	Rice straw	Positive	activation of DNA endogenous promoter	1.44E-04	395
Jejunum	Rice straw	Positive	proliferation of endothelial cells	1.52E-04	111
Jejunum	Rice straw	Positive	peroxisomal disorder	1.57E-04	16
Jejunum	Rice straw	Positive	homing	1.63E-04	196
Jejunum	Rice	Positive	formation of filopodia	1.63E-04	63

	straw				
Jejunum	Rice straw	Positive	differentiation of connective tissue cells	1.64E-04	218
Jejunum	Rice straw	Positive	development of blood cells	1.71E-04	221
Jejunum	Rice straw	Positive	polyneuropathy	1.71E-04	44
Jejunum	Rice straw	Positive	development of neurons	1.71E-04	262
Jejunum	Rice straw	Positive	memory	1.71E-04	93
Jejunum	Rice straw	Positive	concentration of Ca2+	1.85E-04	50
Jejunum	Rice straw	Positive	growth of plasma membrane projections	1.86E-04	161
Jejunum	Rice straw	Positive	apoptosis of leukocytes	1.86E-04	158
Jejunum	Rice straw	Positive	migration of endothelial cells	1.93E-04	116
Jejunum	Rice straw	Positive	immune response of cells	1.97E-04	195
Jejunum	Rice straw	Positive	morphology of reproductive system	1.97E-04	183
Jejunum	Rice straw	Positive	serous ovarian carcinoma	1.99E-04	74
Jejunum	Rice straw	Positive	development of hematopoietic system	1.99E-04	90
Jejunum	Rice straw	Positive	benign ovarian tumor	2.00E-04	75
Jejunum	Rice straw	Positive	proliferation of neuronal cells	2.03E-04	196
Jejunum	Rice straw	Positive	autophagy	2.03E-04	131
Jejunum	Rice straw	Positive	formation of T lymphocytes	2.03E-04	46
Jejunum	Rice straw	Positive	development of leukocytes	2.06E-04	200
Jejunum	Rice straw	Positive	morphology of genital organ	2.11E-04	147
Jejunum	Rice straw	Positive	fibrosis of heart	2.11E-04	68
Jejunum	Rice straw	Positive	cell death of central nervous system cells	2.11E-04	104
Jejunum	Rice	Positive	apoptosis of muscle	2.15E-04	94

	straw				
Jejunum	Rice straw	Positive	endothelial cell development	2.15E-04	124
Jejunum	Rice straw	Positive	quantity of lymphoid organ	2.15E-04	109
Jejunum	Rice straw	Positive	development of endothelial tissue	2.15E-04	128
Jejunum	Rice straw	Positive	cell movement of vascular smooth muscle cells	2.30E-04	45
Jejunum	Rice straw	Positive	cell movement of blood cells	2.33E-04	290
Jejunum	Rice straw	Positive	T cell development	2.33E-04	172
Jejunum	Rice straw	Positive	multiple congenital anomalies	2.36E-04	146
Jejunum	Rice straw	Positive	breast or ovarian cancer	2.37E-04	607
Jejunum	Rice straw	Positive	Ras protein signal transduction	2.40E-04	28
Jejunum	Rice straw	Positive	adenoma	2.40E-04	188
Jejunum	Rice straw	Positive	expression of protein	2.40E-04	119
Jejunum	Rice straw	Positive	cell movement of fibroblasts	2.43E-04	68
Jejunum	Rice straw	Positive	cell death of immune cells	2.45E-04	222
Jejunum	Rice straw	Positive	hereditary neuropathy	2.52E-04	62
Jejunum	Rice straw	Positive	cell movement of granulocytes	2.54E-04	129
Jejunum	Rice straw	Positive	potentiation of synapse	2.54E-04	57
Jejunum	Rice straw	Positive	congenital malformation of skeleton	2.55E-04	146
Jejunum	Rice straw	Positive	proliferation of cervical cancer cell lines	2.57E-04	74
Jejunum	Rice straw	Positive	cell death of heart cells	2.61E-04	80
Jejunum	Rice straw	Positive	apoptosis of muscle cells	2.73E-04	93
Jejunum	Rice straw	Positive	migration of connective tissue cells	2.79E-04	67
Jejunum	Rice	Positive	formation of vascular lesion	2.80E-04	45

	straw				
Jejunum	Rice straw	Positive	infection of cells	2.83E-04	265
Jejunum	Rice straw	Positive	secretion of molecule	2.88E-04	187
Jejunum	Rice straw	Positive	ruffling	2.92E-04	43
Jejunum	Rice straw	Positive	concentration of phosphatidic acid	2.92E-04	52
Jejunum	Rice straw	Positive	development of genitourinary system	2.92E-04	293
Jejunum	Rice straw	Positive	abnormal morphology of genital organ	2.92E-04	141
Jejunum	Rice straw	Positive	migration of myeloid cells	2.93E-04	62
Jejunum	Rice straw	Positive	advanced stage peripheral arterial disease	3.00E-04	57
Jejunum	Rice straw	Positive	cell viability of kidney cell lines	3.09E-04	32
Jejunum	Rice straw	Positive	shape change of tumor cell lines	3.10E-04	58
Jejunum	Rice straw	Positive	colony formation	3.12E-04	182
Jejunum	Rice straw	Positive	recruitment of cells	3.15E-04	124
Jejunum	Rice straw	Positive	chemotaxis of cells	3.18E-04	179
Jejunum	Rice straw	Positive	morphology of gonad	3.18E-04	131
Jejunum	Rice straw	Positive	Emery-Dreifuss muscular dystrophy	3.22E-04	28
Jejunum	Rice straw	Positive	spatial learning	3.22E-04	68
Jejunum	Rice straw	Positive	growth of neurites	3.22E-04	158
Jejunum	Rice straw	Positive	migration of blood cells	3.24E-04	288
Jejunum	Rice straw	Positive	cell death of heart	3.33E-04	82
Jejunum	Rice straw	Positive	hereditary polyneuropathy	3.34E-04	40
Jejunum	Rice straw	Positive	transactivation of RNA	3.36E-04	184
Jejunum	Rice	Positive	replication of RNA virus	3.36E-04	172

	straw				
Jejunum	Rice straw	Positive	long-term potentiation of synapse	3.38E-04	56
Jejunum	Rice straw	Positive	cell death of epithelial cells	3.40E-04	169
Jejunum	Rice straw	Positive	proliferation of blood cells	3.66E-04	278
Jejunum	Rice straw	Positive	morphology of central nervous system	3.72E-04	174
Jejunum	Rice straw	Positive	formation of mononuclear leukocytes	3.79E-04	50
Jejunum	Rice straw	Positive	leukocyte migration	3.79E-04	287
Jejunum	Rice straw	Positive	development of connective tissue cells	3.83E-04	80
Jejunum	Rice straw	Positive	development of reproductive system	3.83E-04	245
Jejunum	Rice straw	Positive	chemotaxis	3.93E-04	184
Jejunum	Rice straw	Positive	cell viability of embryonic cell lines	4.02E-04	29
Jejunum	Rice straw	Positive	female genital tract adenocarcinoma	4.04E-04	895
Jejunum	Rice straw	Positive	uptake of carbohydrate	4.16E-04	102
Jejunum	Rice straw	Positive	function of phagocytes	4.19E-04	107
Jejunum	Rice straw	Positive	concentration of phospholipid	4.31E-04	67
Jejunum	Rice straw	Positive	cell death of cerebral cortex cells	4.33E-04	82
Jejunum	Rice straw	Positive	outgrowth of cells	4.40E-04	148
Jejunum	Rice straw	Positive	proliferation of fibroblasts	4.41E-04	122
Jejunum	Rice straw	Positive	cell death of cardiomyocytes	4.41E-04	77
Jejunum	Rice straw	Positive	proliferation of hepatoma cell lines	4.41E-04	72
Jejunum	Rice straw	Positive	transmembrane potential	4.44E-04	98
Jejunum	Rice straw	Positive	chronic psoriasis	4.46E-04	54
Jejunum	Rice	Positive	migration of vascular smooth muscle cells	4.58E-04	41

	straw				
Jejunum	Rice straw	Positive	cell death of brain	4.61E-04	102
Jejunum	Rice straw	Positive	size of embryo	4.73E-04	123
Jejunum	Rice straw	Positive	cell death of mononuclear leukocytes	4.76E-04	136
Jejunum	Rice straw	Positive	apoptosis of epithelial cells	4.76E-04	100
Jejunum	Rice straw	Positive	proliferation of smooth muscle cells	4.76E-04	100
Jejunum	Rice straw	Positive	cell death of cortical neurons	4.76E-04	64
Jejunum	Rice straw	Positive	synthesis of ATP	4.77E-04	37
Jejunum	Rice straw	Positive	organization of actin cytoskeleton	4.78E-04	105
Jejunum	Rice straw	Positive	laminopathy	4.79E-04	30
Jejunum	Rice straw	Positive	transport of alpha-amino acid	4.79E-04	30
Jejunum	Rice straw	Positive	formation of lymphocytes	4.86E-04	48
Jejunum	Rice straw	Positive	dephosphorylation of protein	4.90E-04	68
Jejunum	Rice straw	Positive	apoptosis of fibroblasts	4.90E-04	78
Jejunum	Rice straw	Positive	necrosis of cardiac muscle	4.90E-04	78
Jejunum	Rice straw	Positive	cell death of neuroblastoma cell lines	4.93E-04	70
Jejunum	Rice straw	Positive	proliferation of immune cells	5.11E-04	261
Jejunum	Rice straw	Positive	cell movement of leukocytes	5.34E-04	253
Jejunum	Rice straw	Positive	internalization of lipid	5.47E-04	14
Jejunum	Rice straw	Positive	development of mononuclear leukocytes	5.47E-04	187
Jejunum	Rice straw	Positive	differentiation of bone	5.48E-04	140
Jejunum	Rice straw	Positive	quantity of metal	5.63E-04	168
Jejunum	Rice	Positive	dendritic growth/branching	5.63E-04	78

	straw				
Jejunum	Rice straw	Positive	cell movement of tumor cells	5.64E-04	67
Jejunum	Rice straw	Positive	migration of cancer cells	5.71E-04	70
Jejunum	Rice straw	Positive	recruitment of phagocytes	5.84E-04	87
Jejunum	Rice straw	Positive	abnormal morphology of gonad	5.87E-04	125
Jejunum	Rice straw	Positive	migration of vascular endothelial cells	6.20E-04	61
Jejunum	Rice straw	Positive	morphology of limb	6.23E-04	82
Jejunum	Rice straw	Positive	cell movement of myeloid cells	6.24E-04	181
Jejunum	Rice straw	Positive	quantity of lymphatic system component	6.35E-04	129
Jejunum	Rice straw	Positive	craniofacial abnormality	6.40E-04	122
Jejunum	Rice straw	Positive	DNA replication	6.52E-04	88
Jejunum	Rice straw	Positive	ion homeostasis of cells	6.57E-04	186
Jejunum	Rice straw	Positive	colon carcinoma	6.57E-04	1077
Jejunum	Rice straw	Positive	uptake of monosaccharide	6.61E-04	94
Jejunum	Rice straw	Positive	development of lymphocytes	6.80E-04	185
Jejunum	Rice straw	Positive	mammary tumor	6.80E-04	519
Jejunum	Rice straw	Positive	recruitment of macrophages	6.91E-04	41
Jejunum	Rice straw	Positive	T cell homeostasis	7.03E-04	173
Jejunum	Rice straw	Positive	migration of fibroblasts	7.17E-04	53
Jejunum	Rice straw	Positive	arrest in growth of organism	7.18E-04	61
Jejunum	Rice straw	Positive	epileptic seizure	7.18E-04	61
Jejunum	Rice straw	Positive	size of lesion	7.18E-04	89
Jejunum	Rice	Positive	synthesis of purine nucleotide	7.32E-04	63

	straw				
Jejunum	Rice straw	Positive	abnormal morphology of muscle	7.32E-04	109
Jejunum	Rice straw	Positive	cell death of cervical cancer cell lines	7.39E-04	120
Jejunum	Rice straw	Positive	apoptosis of heart cells	7.53E-04	68
Jejunum	Rice straw	Positive	development of hematopoietic progenitor cells	7.53E-04	69
Jejunum	Rice straw	Positive	differentiation of bone cells	7.53E-04	138
Jejunum	Rice straw	Positive	urothelial cancer	7.63E-04	50
Jejunum	Rice straw	Positive	cell death of muscle cells	7.64E-04	115
Jejunum	Rice straw	Positive	transport of mitochondria	7.64E-04	13
Jejunum	Rice straw	Positive	morphology of artery	7.81E-04	57
Jejunum	Rice straw	Positive	fertility	7.95E-04	124
Jejunum	Rice straw	Positive	proliferation of hematopoietic cells	8.02E-04	89
Jejunum	Rice straw	Positive	Wound	8.04E-04	80
Jejunum	Rice straw	Positive	arthropathy	8.16E-04	309
Jejunum	Rice straw	Positive	cell viability of cervical cancer cell lines	8.19E-04	78
Jejunum	Rice straw	Positive	tumorigenesis of reproductive tract	8.33E-04	1050
Jejunum	Rice straw	Positive	cell viability of carcinoma cell lines	8.36E-04	53
Jejunum	Rice straw	Positive	binding of DNA	8.39E-04	174
Jejunum	Rice straw	Positive	formation of lymphoid organ	8.49E-04	92
Jejunum	Rice straw	Positive	quantity of carbohydrate	8.71E-04	173
Jejunum	Rice straw	Positive	quantity of metal ion	8.82E-04	149
Jejunum	Rice straw	Positive	seizures	9.00E-04	135
Jejunum	Rice	Positive	female genital tract cancer	9.07E-04	1043

	straw				
Jejunum	Rice straw	Positive	abnormal morphology of skeleton	9.17E-04	94
Jejunum	Rice straw	Positive	migration of tumor cells	9.20E-04	79
Jejunum	Rice straw	Positive	experimentally-induced diabetes	9.28E-04	37
Jejunum	Rice straw	Positive	metabolism of D-hexose	9.28E-04	37
Jejunum	Rice straw	Positive	hepatobiliary system cancer	9.33E-04	1324
Jejunum	Rice straw	Positive	formation of thymus gland	9.36E-04	59
Jejunum	Rice straw	Positive	Thrombosis	9.59E-04	61
Jejunum	Rice straw	Positive	quantity of mononuclear leukocytes	9.65E-04	241
Jejunum	Rice straw	Positive	degeneration of eye	9.65E-04	91
Jejunum	Rice straw	Positive	Breast Cancer and Tumors	9.69E-04	485
Jejunum	Rice straw	Positive	cognitive impairment	9.69E-04	113
Jejunum	Rice straw	Positive	size of animal	9.69E-04	64
Jejunum	Rice straw	Positive	development of hematopoietic cells	9.69E-04	70
Jejunum	Rice straw	Positive	transport of carbohydrate	9.69E-04	69
Jejunum	Rice straw	Positive	apoptosis of cardiomyocytes	9.69E-04	66
Jejunum	Rice straw	Positive	import of lipid	9.69E-04	9
Jejunum	Rice straw	Positive	cell death of renal glomerulus	9.75E-04	25
Jejunum	Rice straw	Positive	psoriasis	9.81E-04	194
Jejunum	Rice straw	Positive	cellular infiltration of cells	9.87E-04	134
Jejunum	Rice straw	Positive	cell movement of cancer cells	9.87E-04	54
Jejunum	Rice straw	Positive	morphology of joint	9.87E-04	28
Jejunum	Rice	Positive	growth of yeast	9.87E-04	38

	straw				
Jejunum	Rice straw	Positive	cellular infiltration	9.92E-04	147
Jejunum	Rice straw	Positive	Lymphocyte homeostasis	9.99E-04	181
Jejunum	Rice straw	Positive	arthritis	1.03E-03	303
Jejunum	Rice straw	Positive	cell death of muscle	1.03E-03	117
Jejunum	Rice straw	Positive	abnormal morphology of skull	1.03E-03	77
Jejunum	Rice straw	Positive	cell death of fibrosarcoma cell lines	1.03E-03	30
Jejunum	Rice straw	Positive	tumorigenesis of genital organ	1.03E-03	1196
Jejunum	Rice straw	Positive	homeostasis of leukocytes	1.03E-03	184
Jejunum	Rice straw	Positive	replication of virus	1.03E-03	186
Jejunum	Rice straw	Positive	morphology of right ventricle	1.03E-03	24
Jejunum	Rice straw	Positive	autosomal dominant myopathy	1.04E-03	34
Jejunum	Rice straw	Positive	urothelial neoplasm	1.07E-03	51
Jejunum	Rice straw	Positive	abnormal morphology of joint	1.08E-03	27
Jejunum	Rice straw	Positive	hypersensitive reaction	1.08E-03	144
Jejunum	Rice straw	Positive	retinal degeneration	1.08E-03	89
Jejunum	Rice straw	Positive	litter size	1.09E-03	66
Jejunum	Rice straw	Positive	cell death of brain cells	1.09E-03	94
Jejunum	Rice straw	Positive	apoptosis of neurons	1.10E-03	146
Jejunum	Rice straw	Positive	necrosis of renal glomerulus	1.10E-03	23
Jejunum	Rice straw	Positive	abnormal morphology of limb	1.11E-03	80
Jejunum	Rice straw	Positive	growth of muscle tissue	1.13E-03	126
Jejunum	Rice	Positive	serous neoplasm	1.13E-03	201

	straw				
Jejunum	Rice straw	Positive	liver tumor	1.14E-03	1325
Jejunum	Rice straw	Positive	targeting of protein	1.14E-03	48
Jejunum	Rice straw	Positive	abnormal morphology of abdomen	1.14E-03	284
Jejunum	Rice straw	Positive	hereditary motor and sensory neuropathy	1.14E-03	35
Jejunum	Rice straw	Positive	metabolism of D-glucose	1.14E-03	35
Jejunum	Rice straw	Positive	G1 phase	1.14E-03	131
Jejunum	Rice straw	Positive	morphology of skeleton	1.15E-03	96
Jejunum	Rice straw	Positive	consumption of oxygen	1.16E-03	56
Jejunum	Rice straw	Positive	formation of lymphatic system component	1.16E-03	100
Jejunum	Rice straw	Positive	autosomal dominant Emery-Dreifuss muscular dystrophy	1.16E-03	26
Jejunum	Rice straw	Positive	proliferation of mononuclear leukocytes	1.16E-03	241
Jejunum	Rice straw	Positive	permeability of cells	1.18E-03	38
Jejunum	Rice straw	Positive	proliferation of T lymphocytes	1.19E-03	199
Jejunum	Rice straw	Positive	arrest in growth of embryo	1.20E-03	59
Jejunum	Rice straw	Positive	necrosis of muscle	1.25E-03	116
Jejunum	Rice straw	Positive	synthesis of protein	1.25E-03	149
Jejunum	Rice straw	Positive	breast cancer	1.25E-03	482
Jejunum	Rice straw	Positive	oral cancer	1.25E-03	79
Jejunum	Rice straw	Positive	proliferation of muscle cells	1.25E-03	125
Jejunum	Rice straw	Positive	apoptosis of epithelial cell lines	1.27E-03	78
Jejunum	Rice straw	Positive	juvenile dermatomyositis	1.27E-03	25
Jejunum	Rice	Positive	morphology of cardiovascular tissue	1.28E-03	34

	straw				
Jejunum	Rice straw	Positive	abnormal morphology of vascular endothelial cells	1.28E-03	19
Jejunum	Rice straw	Positive	morphology of brain	1.28E-03	155
Jejunum	Rice straw	Positive	quantity of lymphocytes	1.29E-03	231
Jejunum	Rice straw	Positive	proliferation of hematopoietic progenitor cells	1.29E-03	84
Jejunum	Rice straw	Positive	morphology of bone	1.29E-03	150
Jejunum	Rice straw	Positive	formation of actin stress fibers	1.31E-03	83
Jejunum	Rice straw	Positive	genital tract cancer	1.32E-03	1184
Jejunum	Rice straw	Positive	proliferation of lymphocytes	1.33E-03	237
Jejunum	Rice straw	Positive	liver cancer	1.35E-03	1315
Jejunum	Rice straw	Positive	mental retardation	1.35E-03	81
Jejunum	Rice straw	Positive	transitional cell bladder cancer	1.36E-03	44
Jejunum	Rice straw	Positive	transport of amino acids	1.36E-03	49
Jejunum	Rice straw	Positive	development of lymphatic system component	1.36E-03	105
Jejunum	Rice straw	Positive	morphology of muscle cells	1.37E-03	59
Jejunum	Rice straw	Positive	abnormal morphology of blood vessel	1.40E-03	91
Jejunum	Rice straw	Positive	morphology of skull	1.41E-03	78
Jejunum	Rice straw	Positive	seizure disorder	1.44E-03	156
Jejunum	Rice straw	Positive	cellular infiltration of blood cells	1.52E-03	130
Jejunum	Rice straw	Positive	differentiation of tumor cell lines	1.53E-03	127
Jejunum	Rice straw	Positive	abnormal morphology of body wall	1.55E-03	39
Jejunum	Rice straw	Positive	cell death of lymphocytes	1.57E-03	129
Jejunum	Rice	Positive	secretory pathway	1.57E-03	69

	straw				
Jejunum	Rice straw	Positive	quantity of cellular protrusions	1.58E-03	57
Jejunum	Rice straw	Positive	formation of skin	1.58E-03	123
Jejunum	Rice straw	Positive	degeneration of central nervous system	1.58E-03	48
Jejunum	Rice straw	Positive	abnormal morphology of central nervous system	1.58E-03	160
Jejunum	Rice straw	Positive	branching of vasculature	1.59E-03	14
Jejunum	Rice straw	Positive	internalization of cells	1.59E-03	59
Jejunum	Rice straw	Positive	cell movement of connective tissue cells	1.62E-03	77
Jejunum	Rice straw	Positive	endometrium tumor	1.63E-03	887
Jejunum	Rice straw	Positive	proliferation of bone marrow cells	1.64E-03	44
Jejunum	Rice straw	Positive	cell viability of epithelial cell lines	1.64E-03	37
Jejunum	Rice straw	Positive	glioma cancer	1.66E-03	105
Jejunum	Rice straw	Positive	function of myeloid cells	1.68E-03	82
Jejunum	Rice straw	Positive	apoptosis of neuroblastoma cell lines	1.70E-03	45
Jejunum	Rice straw	Positive	metabolism of monosaccharide	1.70E-03	45
Jejunum	Rice straw	Positive	development of digestive system	1.75E-03	128
Jejunum	Rice straw	Positive	cell movement of epithelial cells	1.75E-03	53
Jejunum	Rice straw	Positive	morphology of mitochondria	1.76E-03	38
Jejunum	Rice straw	Positive	inflammatory response	1.76E-03	252
Jejunum	Rice straw	Positive	abnormal morphology of artery	1.76E-03	54
Jejunum	Rice straw	Positive	colon adenocarcinoma	1.84E-03	1049
Jejunum	Rice straw	Positive	hereditary spastic paraplegia	1.87E-03	19
Jejunum	Rice	Positive	catabolism of protein	1.88E-03	197

	straw				
Jejunum	Rice straw	Positive	quantity of connective tissue	1.88E-03	197
Jejunum	Rice straw	Positive	adhesion of immune cells	1.88E-03	117
Jejunum	Rice straw	Positive	gliosis of brain	1.91E-03	18
Jejunum	Rice straw	Positive	organization of peroxisomes	1.92E-03	12
Jejunum	Rice straw	Positive	formation of connective tissue cells	1.93E-03	73
Jejunum	Rice straw	Positive	quantity of thymus gland	1.95E-03	80
Jejunum	Rice straw	Positive	metabolism of hexose	1.98E-03	40
Jejunum	Rice straw	Positive	disorder of pregnancy	1.98E-03	162
Jejunum	Rice straw	Positive	cellular infiltration by leukocytes	2.00E-03	129
Jejunum	Rice straw	Positive	cell movement of phagocytes	2.00E-03	180
Jejunum	Rice straw	Positive	abnormal morphology of endothelial tissue	2.01E-03	26
Jejunum	Rice straw	Positive	transport of L-amino acid	2.01E-03	26
Jejunum	Rice straw	Negative	proliferation of cells	6.06E-47	1645
Jejunum	Rice straw	Negative	malignant solid tumor	6.16E-42	4107
Jejunum	Rice straw	Negative	cancer	6.16E-42	4151
Jejunum	Rice straw	Negative	morbidity or mortality	1.21E-38	1122
Jejunum	Rice straw	Negative	organismal death	1.21E-38	1109
Jejunum	Rice straw	Negative	cell death	9.65E-38	1467
Jejunum	Rice straw	Negative	morphology of cells	5.05E-33	909
Jejunum	Rice straw	Negative	apoptosis	9.13E-32	1175
Jejunum	Rice straw	Negative	organization of cytoplasm	6.12E-31	735
Jejunum	Rice	Negative	cell movement	1.34E-29	966

	straw				
Jejunum	Rice straw	Negative	necrosis	2.43E-29	1136
Jejunum	Rice straw	Negative	tumorigenesis of tissue	1.15E-28	3465
Jejunum	Rice straw	Negative	neoplasia of epithelial tissue	1.42E-28	3413
Jejunum	Rice straw	Negative	epithelial cancer	4.50E-27	3375
Jejunum	Rice straw	Negative	organization of cytoskeleton	4.85E-26	660
Jejunum	Rice straw	Negative	abdominal neoplasm	6.11E-25	3368
Jejunum	Rice straw	Negative	migration of cells	2.46E-24	854
Jejunum	Rice straw	Negative	differentiation of cells	5.51E-23	982
Jejunum	Rice straw	Negative	abdominal cancer	7.13E-23	3319
Jejunum	Rice straw	Negative	microtubule dynamics	6.85E-21	554
Jejunum	Rice straw	Negative	transport of molecule	6.91E-21	724
Jejunum	Rice straw	Negative	cellular homeostasis	8.70E-21	688
Jejunum	Rice straw	Negative	expression of RNA	9.42E-20	925
Jejunum	Rice straw	Negative	angiogenesis	4.00E-19	434
Jejunum	Rice straw	Negative	transcription of RNA	5.52E-19	810
Jejunum	Rice straw	Negative	cell death of tumor cell lines	5.80E-19	681
Jejunum	Rice straw	Negative	transcription	7.13E-19	862
Jejunum	Rice straw	Negative	cell survival	1.30E-18	627
Jejunum	Rice straw	Negative	quantity of cells	6.01E-18	770
Jejunum	Rice straw	Negative	cell viability	2.01E-17	585
Jejunum	Rice straw	Negative	vasculogenesis	5.10E-17	355
Jejunum	Rice	Negative	digestive organ tumor	1.79E-16	2882

	straw				
Jejunum	Rice straw	Negative	abnormal morphology of cells	4.10E-16	565
Jejunum	Rice straw	Negative	formation of cellular protrusions	4.50E-16	420
Jejunum	Rice straw	Negative	digestive system cancer	9.36E-16	2851
Jejunum	Rice straw	Negative	Viral Infection	1.03E-15	698
Jejunum	Rice straw	Negative	apoptosis of tumor cell lines	2.34E-15	540
Jejunum	Rice straw	Negative	organization of organelle	2.77E-15	265
Jejunum	Rice straw	Negative	cell movement of tumor cell lines	4.22E-15	395
Jejunum	Rice straw	Negative	Movement Disorders	5.94E-15	478
Jejunum	Rice straw	Negative	proliferation of tumor cell lines	9.02E-15	668
Jejunum	Rice straw	Negative	cell death of connective tissue cells	1.03E-14	290
Jejunum	Rice straw	Negative	proliferation of connective tissue cells	1.75E-14	285
Jejunum	Rice straw	Negative	size of body	3.10E-14	391
Jejunum	Rice straw	Negative	morphology of cardiovascular system	3.88E-14	295
Jejunum	Rice straw	Negative	homing of cells	6.72E-14	268
Jejunum	Rice straw	Negative	autosomal recessive disease	7.04E-14	418
Jejunum	Rice straw	Negative	growth of connective tissue	8.37E-14	304
Jejunum	Rice straw	Negative	migration of blood cells	8.87E-14	398
Jejunum	Rice straw	Negative	leukocyte migration	1.10E-13	397
Jejunum	Rice straw	Negative	quantity of blood cells	1.25E-13	459
Jejunum	Rice straw	Negative	phosphorylation of protein	1.25E-13	341
Jejunum	Rice straw	Negative	function of cardiovascular system	1.34E-13	186
Jejunum	Rice	Negative	transcription of DNA	1.49E-13	656

	straw				
Jejunum	Rice straw	Negative	homing	1.94E-13	273
Jejunum	Rice straw	Negative	quantity of leukocytes	2.54E-13	413
Jejunum	Rice straw	Negative	concentration of lipid	5.97E-13	369
Jejunum	Rice straw	Negative	cell spreading	8.04E-13	143
Jejunum	Rice straw	Negative	chemotaxis of cells	8.72E-13	251
Jejunum	Rice straw	Negative	development of cytoplasm	1.08E-12	223
Jejunum	Rice straw	Negative	Growth Failure	1.48E-12	280
Jejunum	Rice straw	Negative	chemotaxis	2.14E-12	257
Jejunum	Rice straw	Negative	cell movement of leukocytes	2.96E-12	348
Jejunum	Rice straw	Negative	formation of cytoskeleton	5.22E-12	184
Jejunum	Rice straw	Negative	proliferation of fibroblasts	5.24E-12	174
Jejunum	Rice straw	Negative	metabolism of carbohydrate	5.74E-12	287
Jejunum	Rice straw	Negative	invasion of cells	6.14E-12	388
Jejunum	Rice straw	Negative	protein kinase cascade	6.51E-12	203
Jejunum	Rice straw	Negative	synthesis of lipid	6.63E-12	325
Jejunum	Rice straw	Negative	migration of tumor cell lines	1.84E-11	320
Jejunum	Rice straw	Negative	inflammatory response	2.06E-11	349
Jejunum	Rice straw	Negative	development of body trunk	2.66E-11	482
Jejunum	Rice straw	Negative	growth of organism	3.43E-11	371
Jejunum	Rice straw	Negative	growth of embryo	3.43E-11	224
Jejunum	Rice straw	Negative	cell movement of myeloid cells	4.13E-11	250
Jejunum	Rice	Negative	fibrogenesis	4.20E-11	196

	straw				
Jejunum	Rice straw	Negative	necrosis of epithelial tissue	4.36E-11	270
Jejunum	Rice straw	Negative	ubiquitination	5.20E-11	164
Jejunum	Rice straw	Negative	function of blood cells	8.24E-11	268
Jejunum	Rice straw	Negative	development of vasculature	8.24E-11	198
Jejunum	Rice straw	Negative	morphology of body cavity	9.20E-11	509
Jejunum	Rice straw	Negative	formation of filaments	9.27E-11	189
Jejunum	Rice straw	Negative	ubiquitination of protein	9.27E-11	161
Jejunum	Rice straw	Negative	disorder of basal ganglia	9.36E-11	341
Jejunum	Rice straw	Negative	transactivation	1.05E-10	264
Jejunum	Rice straw	Negative	morphology of heart	1.13E-10	192
Jejunum	Rice straw	Negative	cell cycle progression	1.30E-10	451
Jejunum	Rice straw	Negative	abnormal morphology of thoracic cavity	1.35E-10	267
Jejunum	Rice straw	Negative	cell death of fibroblast cell lines	2.25E-10	200
Jejunum	Rice straw	Negative	colony formation of cells	2.26E-10	227
Jejunum	Rice straw	Negative	degeneration of cells	2.26E-10	165
Jejunum	Rice straw	Negative	Huntington's Disease	2.43E-10	261
Jejunum	Rice straw	Negative	apoptosis of fibroblast cell lines	2.53E-10	158
Jejunum	Rice straw	Negative	abnormal morphology of cardiovascular system	2.53E-10	258
Jejunum	Rice straw	Negative	activation of cells	3.19E-10	422
Jejunum	Rice straw	Negative	abnormal morphology of body cavity	3.74E-10	485
Jejunum	Rice straw	Negative	differentiation of connective tissue cells	3.74E-10	288
Jejunum	Rice	Negative	invasion of tumor cell lines	3.89E-10	297

	straw				
Jejunum	Rice straw	Negative	proliferation of blood cells	3.89E-10	368
Jejunum	Rice straw	Negative	dyskinesia	4.59E-10	277
Jejunum	Rice straw	Negative	formation of muscle	5.07E-10	186
Jejunum	Rice straw	Negative	metabolism of protein	7.57E-10	418
Jejunum	Rice straw	Negative	neurological signs	7.60E-10	290
Jejunum	Rice straw	Negative	cell viability of tumor cell lines	8.40E-10	341
Jejunum	Rice straw	Negative	activation of DNA endogenous promoter	9.12E-10	509
Jejunum	Rice straw	Negative	proliferation of neuronal cells	9.32E-10	259
Jejunum	Rice straw	Negative	development of neurons	9.91E-10	342
Jejunum	Rice straw	Negative	differentiation of connective tissue	9.97E-10	321
Jejunum	Rice straw	Negative	cell movement of fibroblasts	1.03E-09	94
Jejunum	Rice straw	Negative	mitosis	1.04E-09	226
Jejunum	Rice straw	Negative	morphology of muscle	1.04E-09	159
Jejunum	Rice straw	Negative	Neurodegeneration	2.02E-09	147
Jejunum	Rice straw	Negative	neuromuscular disease	2.24E-09	380
Jejunum	Rice straw	Negative	cell transformation	2.29E-09	210
Jejunum	Rice straw	Negative	colony formation	2.50E-09	241
Jejunum	Rice straw	Negative	infection of cells	2.54E-09	346
Jejunum	Rice straw	Negative	cell movement of phagocytes	2.74E-09	246
Jejunum	Rice straw	Negative	function of leukocytes	2.88E-09	241
Jejunum	Rice straw	Negative	quantity of connective tissue	4.07E-09	267
Jejunum	Rice	Negative	neuritogenesis	4.08E-09	259

	straw				
Jejunum	Rice straw	Negative	development of blood cells	4.25E-09	287
Jejunum	Rice straw	Negative	vascularization	4.62E-09	115
Jejunum	Rice straw	Negative	cell death of muscle	6.48E-09	160
Jejunum	Rice straw	Negative	degeneration of nervous system	6.63E-09	136
Jejunum	Rice straw	Negative	synthesis of reactive oxygen species	6.63E-09	216
Jejunum	Rice straw	Negative	transactivation of RNA	6.63E-09	242
Jejunum	Rice straw	Negative	growth of epithelial tissue	6.63E-09	297
Jejunum	Rice straw	Negative	engulfment of cells	6.63E-09	186
Jejunum	Rice straw	Negative	replication of virus	7.10E-09	249
Jejunum	Rice straw	Negative	length of cells	7.23E-09	56
Jejunum	Rice straw	Negative	cell death of muscle cells	7.83E-09	156
Jejunum	Rice straw	Negative	Hypertrophy	7.91E-09	213
Jejunum	Rice straw	Negative	necrosis of muscle	7.91E-09	159
Jejunum	Rice straw	Negative	proliferation of immune cells	8.15E-09	341
Jejunum	Rice straw	Negative	behavior	8.54E-09	418
Jejunum	Rice straw	Negative	replication of RNA virus	9.37E-09	226
Jejunum	Rice straw	Negative	cell movement of endothelial cells	1.11E-08	163
Jejunum	Rice straw	Negative	development of epithelial tissue	1.11E-08	230
Jejunum	Rice straw	Negative	metabolism of membrane lipid derivative	1.15E-08	177
Jejunum	Rice straw	Negative	cell movement of connective tissue cells	1.15E-08	108
Jejunum	Rice straw	Negative	degeneration of neurons	1.19E-08	115
Jejunum	Rice	Negative	quantity of phagocytes	1.20E-08	192

	straw				
Jejunum	Rice straw	Negative	recruitment of cells	1.23E-08	164
Jejunum	Rice straw	Negative	formation of plasma membrane projections	1.24E-08	262
Jejunum	Rice straw	Negative	abnormal morphology of heart	1.27E-08	175
Jejunum	Rice straw	Negative	secretory pathway	1.41E-08	97
Jejunum	Rice straw	Negative	quantity of lymphocytes	1.47E-08	306
Jejunum	Rice straw	Negative	quantity of mononuclear leukocytes	1.55E-08	317
Jejunum	Rice straw	Negative	cell death of epithelial cells	1.75E-08	221
Jejunum	Rice straw	Negative	proliferation of fibroblast cell lines	1.76E-08	191
Jejunum	Rice straw	Negative	metabolism of reactive oxygen species	1.89E-08	222
Jejunum	Rice straw	Negative	morphology of connective tissue	1.96E-08	206
Jejunum	Rice straw	Negative	organization of actin cytoskeleton	2.21E-08	140
Jejunum	Rice straw	Negative	development of connective tissue	2.21E-08	169
Jejunum	Rice straw	Negative	vascularization of body region	2.62E-08	70
Jejunum	Rice straw	Negative	function of muscle	2.90E-08	159
Jejunum	Rice straw	Negative	cell movement of neutrophils	3.03E-08	138
Jejunum	Rice straw	Negative	development of leukocytes	3.03E-08	257
Jejunum	Rice straw	Negative	neuronal cell death	3.06E-08	294
Jejunum	Rice straw	Negative	cellular degradation	3.06E-08	120
Jejunum	Rice straw	Negative	exocytosis	3.38E-08	93
Jejunum	Rice straw	Negative	uptake of monosaccharide	3.63E-08	126
Jejunum	Rice straw	Negative	morphology of nervous system	3.63E-08	334
Jejunum	Rice	Negative	growth of plasma membrane projections	3.94E-08	207

	straw				
Jejunum	Rice straw	Negative	congenital anomaly of musculoskeletal system	4.03E-08	315
Jejunum	Rice straw	Negative	development of lymphatic system	4.06E-08	162
Jejunum	Rice straw	Negative	abnormal morphology of abdomen	4.20E-08	370
Jejunum	Rice straw	Negative	learning	4.35E-08	187
Jejunum	Rice straw	Negative	formation of cells	4.45E-08	411
Jejunum	Rice straw	Negative	long-term potentiation	4.68E-08	115
Jejunum	Rice straw	Negative	chemotaxis of myeloid cells	5.22E-08	135
Jejunum	Rice straw	Negative	abnormal morphology of respiratory system	5.58E-08	141
Jejunum	Rice straw	Negative	apoptosis of heart cells	5.82E-08	92
Jejunum	Rice straw	Negative	apoptosis of cardiomyocytes	5.98E-08	90
Jejunum	Rice straw	Negative	cellular infiltration by leukocytes	6.28E-08	174
Jejunum	Rice straw	Negative	cell death of blood cells	6.34E-08	297
Jejunum	Rice straw	Negative	uptake of carbohydrate	6.70E-08	134
Jejunum	Rice straw	Negative	activation of blood cells	7.14E-08	318
Jejunum	Rice straw	Negative	cardiogenesis	7.62E-08	208
Jejunum	Rice straw	Negative	proliferation of hematopoietic cells	7.62E-08	119
Jejunum	Rice straw	Negative	migration of connective tissue cells	8.02E-08	88
Jejunum	Rice straw	Negative	cellular infiltration	8.07E-08	194
Jejunum	Rice straw	Negative	accumulation of lipid	8.36E-08	137
Jejunum	Rice straw	Negative	abnormal morphology of epithelial tissue	8.66E-08	186
Jejunum	Rice straw	Negative	benign neoplasia	8.66E-08	387
Jejunum	Rice	Negative	morphology of respiratory system	9.18E-08	142

	straw				
Jejunum	Rice straw	Negative	cell death of epithelial cell lines	1.01E-07	130
Jejunum	Rice straw	Negative	outgrowth of plasma membrane projections	1.04E-07	181
Jejunum	Rice straw	Negative	formation of actin filaments	1.10E-07	139
Jejunum	Rice straw	Negative	cell death of heart cells	1.12E-07	104
Jejunum	Rice straw	Negative	cognition	1.13E-07	200
Jejunum	Rice straw	Negative	growth of muscle tissue	1.14E-07	167
Jejunum	Rice straw	Negative	proliferation of muscle cells	1.22E-07	166
Jejunum	Rice straw	Negative	growth of neurites	1.22E-07	203
Jejunum	Rice straw	Negative	cell death of heart	1.25E-07	107
Jejunum	Rice straw	Negative	cell death of immune cells	1.26E-07	282
Jejunum	Rice straw	Negative	seizure disorder	1.32E-07	206
Jejunum	Rice straw	Negative	apoptosis of connective tissue cells	1.35E-07	135
Jejunum	Rice straw	Negative	infection by RNA virus	1.35E-07	363
Jejunum	Rice straw	Negative	muscular hypertrophy	1.38E-07	97
Jejunum	Rice straw	Negative	T cell development	1.41E-07	219
Jejunum	Rice straw	Negative	quantity of T lymphocytes	1.46E-07	232
Jejunum	Rice straw	Negative	proliferation of hematopoietic progenitor cells	1.49E-07	113
Jejunum	Rice straw	Negative	development of cardiovascular tissue	1.50E-07	165
Jejunum	Rice straw	Negative	activation of leukocytes	1.52E-07	297
Jejunum	Rice straw	Negative	cell death of cardiomyocytes	1.52E-07	101
Jejunum	Rice straw	Negative	migration of endothelial cells	1.54E-07	148
Jejunum	Rice	Negative	breast or colorectal cancer	1.54E-07	1881

	straw				
Jejunum	Rice straw	Negative	morphology of heart ventricle	1.54E-07	92
Jejunum	Rice straw	Negative	interphase	1.56E-07	280
Jejunum	Rice straw	Negative	cellular infiltration of cells	1.60E-07	176
Jejunum	Rice straw	Negative	apoptosis of epithelial cell lines	1.62E-07	105
Jejunum	Rice straw	Negative	development of lymphocytes	1.66E-07	239
Jejunum	Rice straw	Negative	cell movement of granulocytes	1.72E-07	165
Jejunum	Rice straw	Negative	migration of fibroblasts	1.77E-07	71
Jejunum	Rice straw	Negative	morphology of vessel	1.80E-07	139
Jejunum	Rice straw	Negative	differentiation of muscle cell lines	1.85E-07	79
Jejunum	Rice straw	Negative	survival of organism	1.93E-07	298
Jejunum	Rice straw	Negative	development of head	2.00E-07	406
Jejunum	Rice straw	Negative	generation of reactive oxygen species	2.16E-07	97
Jejunum	Rice straw	Negative	development of mononuclear leukocytes	2.28E-07	240
Jejunum	Rice straw	Negative	necrosis of cardiac muscle	2.28E-07	102
Jejunum	Rice straw	Negative	homing of blood cells	2.29E-07	168
Jejunum	Rice straw	Negative	proliferation of embryonic cell lines	2.47E-07	77
Jejunum	Rice straw	Negative	demyelination	2.47E-07	61
Jejunum	Rice straw	Negative	quantity of metal	2.51E-07	216
Jejunum	Rice straw	Negative	synthesis of nitric oxide	2.55E-07	126
Jejunum	Rice straw	Negative	outgrowth of neurites	2.56E-07	178
Jejunum	Rice straw	Negative	T cell homeostasis	2.61E-07	223
Jejunum	Rice	Negative	function of phagocytes	2.71E-07	138

	straw				
Jejunum	Rice straw	Negative	cell death of fibroblasts	2.71E-07	121
Jejunum	Rice straw	Negative	abnormal morphology of muscle	3.08E-07	142
Jejunum	Rice straw	Negative	development of lymphatic system component	3.08E-07	139
Jejunum	Rice straw	Negative	function of myeloid cells	3.11E-07	110
Jejunum	Rice straw	Negative	synthesis of carbohydrate	3.15E-07	186
Jejunum	Rice straw	Negative	morphology of head	3.18E-07	364
Jejunum	Rice straw	Negative	infection by Retroviridae	3.20E-07	303
Jejunum	Rice straw	Negative	outgrowth of neurons	3.20E-07	179
Jejunum	Rice straw	Negative	shape change of tumor cell lines	3.22E-07	75
Jejunum	Rice straw	Negative	homeostasis of leukocytes	3.25E-07	238
Jejunum	Rice straw	Negative	endothelial cell development	3.26E-07	157
Jejunum	Rice straw	Negative	development of endothelial tissue	3.26E-07	162
Jejunum	Rice straw	Negative	mass of organism	3.26E-07	149
Jejunum	Rice straw	Negative	Lymphocyte homeostasis	3.30E-07	234
Jejunum	Rice straw	Negative	abnormal morphology of nervous system	3.38E-07	305
Jejunum	Rice straw	Negative	chemotaxis of phagocytes	3.54E-07	133
Jejunum	Rice straw	Negative	proliferation of mononuclear leukocytes	3.67E-07	310
Jejunum	Rice straw	Negative	chemotaxis of leukocytes	3.83E-07	157
Jejunum	Rice straw	Negative	differentiation of epithelial tissue	3.96E-07	159
Jejunum	Rice straw	Negative	recruitment of phagocytes	3.97E-07	113
Jejunum	Rice straw	Negative	morphology of lymphatic system component	4.00E-07	182
Jejunum	Rice	Negative	homing of leukocytes	4.00E-07	166

	straw				
Jejunum	Rice straw	Negative	formation of vascular lesion	4.05E-07	58
Jejunum	Rice straw	Negative	seizures	4.05E-07	175
Jejunum	Rice straw	Negative	apoptosis of muscle	4.33E-07	119
Jejunum	Rice straw	Negative	small GTPase mediated signal transduction	4.48E-07	87
Jejunum	Rice straw	Negative	formation of actin stress fibers	4.55E-07	110
Jejunum	Rice straw	Negative	infection by lentivirus	4.74E-07	300
Jejunum	Rice straw	Negative	HIV infection	5.10E-07	299
Jejunum	Rice straw	Negative	fatty acid metabolism	5.33E-07	255
Jejunum	Rice straw	Negative	arthropathy	5.33E-07	392
Jejunum	Rice straw	Negative	differentiation of blood cells	5.39E-07	350
Jejunum	Rice straw	Negative	apoptosis of muscle cells	5.57E-07	118
Jejunum	Rice straw	Negative	differentiation of bone	5.64E-07	179
Jejunum	Rice straw	Negative	apoptosis of pheochromocytoma cell lines	5.73E-07	48
Jejunum	Rice straw	Negative	differentiation of leukocytes	5.78E-07	283
Jejunum	Rice straw	Negative	size of embryo	6.14E-07	157
Jejunum	Rice straw	Negative	recruitment of leukocytes	6.16E-07	144
Jejunum	Rice straw	Negative	binding of DNA	6.16E-07	223
Jejunum	Rice straw	Negative	outgrowth of cells	6.23E-07	188
Jejunum	Rice straw	Negative	ion homeostasis of cells	6.25E-07	237
Jejunum	Rice straw	Negative	secretion of molecule	6.35E-07	235
Jejunum	Rice straw	Negative	recruitment of blood cells	6.36E-07	146
Jejunum	Rice	Negative	S phase	6.63E-07	118

	straw				
Jejunum	Rice straw	Negative	proliferation of lymphocytes	6.63E-07	304
Jejunum	Rice straw	Negative	function of heart	6.63E-07	106
Jejunum	Rice straw	Negative	uterine serous papillary cancer	6.63E-07	106
Jejunum	Rice straw	Negative	infection by HIV-1	6.65E-07	258
Jejunum	Rice straw	Negative	length of neurons	6.68E-07	46
Jejunum	Rice straw	Negative	development of body axis	6.81E-07	429
Jejunum	Rice straw	Negative	arthritis	6.81E-07	385
Jejunum	Rice straw	Negative	dysmyelination	6.96E-07	66
Jejunum	Rice straw	Negative	proliferation of embryonic cells	6.96E-07	113
Jejunum	Rice straw	Negative	quantity of interleukin	7.07E-07	68
Jejunum	Rice straw	Negative	differentiation of bone cells	7.17E-07	177
Jejunum	Rice straw	Negative	cell movement of muscle cells	7.44E-07	85
Jejunum	Rice straw	Negative	cell movement of mononuclear leukocytes	7.44E-07	199
Jejunum	Rice straw	Negative	quantity of antigen presenting cells	8.20E-07	124
Jejunum	Rice straw	Negative	proliferation of epithelial cells	8.20E-07	211
Jejunum	Rice straw	Negative	phosphorylation of L-amino acid	8.20E-07	101
Jejunum	Rice straw	Negative	cell death of cervical cancer cell lines	8.40E-07	154
Jejunum	Rice straw	Negative	quantity of metal ion	8.57E-07	191
Jejunum	Rice straw	Negative	quantity of myeloid cells	8.61E-07	156
Jejunum	Rice straw	Negative	quantity of carbohydrate	8.74E-07	221
Jejunum	Rice straw	Negative	hypertrophy of cells	8.74E-07	129
Jejunum	Rice	Negative	metabolism of peptide	8.88E-07	87

	straw				
Jejunum	Rice straw	Negative	Organ Degeneration	9.26E-07	211
Jejunum	Rice straw	Negative	abnormal morphology of head	9.27E-07	344
Jejunum	Rice straw	Negative	function of antigen presenting cells	9.27E-07	111
Jejunum	Rice straw	Negative	heart rate	9.75E-07	124
Jejunum	Rice straw	Negative	abnormal morphology of digestive system	1.03E-06	231
Jejunum	Rice straw	Negative	formation of skin	1.05E-06	160
Jejunum	Rice straw	Negative	growth of lesion	1.11E-06	330
Jejunum	Rice straw	Negative	migration of muscle cells	1.13E-06	78
Jejunum	Rice straw	Negative	cell death of endothelial cells	1.14E-06	93
Jejunum	Rice straw	Negative	proliferation of smooth muscle cells	1.18E-06	127
Jejunum	Rice straw	Negative	synthesis of DNA	1.19E-06	180
Jejunum	Rice straw	Negative	hypertrophy of heart	1.23E-06	148
Jejunum	Rice straw	Negative	size of cells	1.29E-06	185
Jejunum	Rice straw	Negative	growth of tumor	1.30E-06	329
Jejunum	Rice straw	Negative	hypertrophy of tissue	1.35E-06	115
Jejunum	Rice straw	Negative	quantity of cytokine	1.44E-06	114
Jejunum	Rice straw	Negative	hyperesthesia	1.45E-06	57
Jejunum	Rice straw	Negative	cell movement of smooth muscle cells	1.53E-06	76
Jejunum	Rice straw	Negative	metabolism of monosaccharide	1.58E-06	60
Jejunum	Rice straw	Negative	maturation of cells	1.68E-06	195
Jejunum	Rice straw	Negative	synthesis of phospholipid	1.70E-06	82
Jejunum	Rice	Negative	modification of peptide	1.73E-06	53

	straw				
Jejunum	Rice straw	Negative	Edema	1.76E-06	150
Jejunum	Rice straw	Negative	hypersensitive reaction	1.83E-06	184
Jejunum	Rice straw	Negative	Bleeding	1.83E-06	182
Jejunum	Rice straw	Negative	growth of embryonic tissue	1.92E-06	122
Jejunum	Rice straw	Negative	apoptosis of endothelial cells	1.96E-06	85
Jejunum	Rice straw	Negative	autophagy	1.97E-06	162
Jejunum	Rice straw	Negative	proliferation of epithelial cell lines	2.01E-06	104
Jejunum	Rice straw	Negative	transport of carbohydrate	2.13E-06	89
Jejunum	Rice straw	Negative	attachment of cells	2.27E-06	65
Jejunum	Rice straw	Negative	metabolism of phospholipid	2.36E-06	105
Jejunum	Rice straw	Negative	quantity of IL-6 in blood	2.80E-06	44
Jejunum	Rice straw	Negative	proliferation of kidney cell lines	2.91E-06	81
Jejunum	Rice straw	Negative	migration of smooth muscle cells	2.94E-06	70
Jejunum	Rice straw	Negative	exocytosis by cells	3.04E-06	61
Jejunum	Rice straw	Negative	breast or ovarian cancer	3.08E-06	746
Jejunum	Rice straw	Negative	morphology of digestive system	3.15E-06	239
Jejunum	Rice straw	Negative	cell death of central nervous system cells	3.28E-06	128
Jejunum	Rice straw	Negative	morphology of bone	3.37E-06	191
Jejunum	Rice straw	Negative	cell death of liver cells	3.40E-06	89
Jejunum	Rice straw	Negative	skin abnormality	3.42E-06	102
Jejunum	Rice straw	Negative	morphology of blood vessel	3.52E-06	124
Jejunum	Rice	Negative	organization of filaments	3.52E-06	93

	straw				
Jejunum	Rice straw	Negative	connective or soft tissue tumor	3.64E-06	291
Jejunum	Rice straw	Negative	congenital malformation of skeleton	3.70E-06	180
Jejunum	Rice straw	Negative	apoptosis of epithelial cells	3.95E-06	125
Jejunum	Rice straw	Negative	cell death of pheochromocytoma cell lines	3.97E-06	58
Jejunum	Rice straw	Negative	quantity of muscle	4.10E-06	62
Jejunum	Rice straw	Negative	cytostasis	4.20E-06	124
Jejunum	Rice straw	Negative	development of digestive system	4.21E-06	164
Jejunum	Rice straw	Negative	production of reactive oxygen species	4.41E-06	157
Jejunum	Rice straw	Negative	perinatal death	4.98E-06	247
Jejunum	Rice straw	Negative	potentiation of synapse	5.04E-06	70
Jejunum	Rice straw	Negative	atrophy of muscle	5.13E-06	69
Jejunum	Rice straw	Negative	cell spreading of fibroblast cell lines	5.19E-06	31
Jejunum	Rice straw	Negative	activation of Protein kinase	5.47E-06	111
Jejunum	Rice straw	Negative	concentration of phosphatidic acid	5.47E-06	64
Jejunum	Rice straw	Negative	cell viability of connective tissue cells	5.49E-06	63
Jejunum	Rice straw	Negative	concentration of fatty acid	5.69E-06	122
Jejunum	Rice straw	Negative	contraction of heart	5.69E-06	82
Jejunum	Rice straw	Negative	neovascularization	5.69E-06	75
Jejunum	Rice straw	Negative	concentration of acylglycerol	5.83E-06	144
Jejunum	Rice straw	Negative	quantity of Ca2+	6.04E-06	173
Jejunum	Rice straw	Negative	Rheumatic Disease	6.05E-06	432
Jejunum	Rice	Negative	abnormal morphology of heart ventricle	6.10E-06	80

	straw				
Jejunum	Rice straw	Negative	morphology of cardiovascular tissue	6.10E-06	44
Jejunum	Rice straw	Negative	female genital tract serous cancer	6.46E-06	164
Jejunum	Rice straw	Negative	formation of eye	6.60E-06	185
Jejunum	Rice straw	Negative	long-term potentiation of synapse	6.60E-06	69
Jejunum	Rice straw	Negative	dilation of heart ventricle	6.80E-06	30
Jejunum	Rice straw	Negative	concentration of phospholipid	6.81E-06	83
Jejunum	Rice straw	Negative	midline defect	6.85E-06	116
Jejunum	Rice straw	Negative	activation of enzyme	6.88E-06	165
Jejunum	Rice straw	Negative	quantity of muscle cells	7.11E-06	56
Jejunum	Rice straw	Negative	function of macrophages	7.37E-06	81
Jejunum	Rice straw	Negative	sprouting	7.37E-06	164
Jejunum	Rice straw	Negative	serine phosphorylation	7.41E-06	47
Jejunum	Rice straw	Negative	apoptosis of liver	7.62E-06	73
Jejunum	Rice straw	Negative	proliferation of lymphatic system cells	7.62E-06	90
Jejunum	Rice straw	Negative	differentiation of epithelial cells	7.65E-06	135
Jejunum	Rice straw	Negative	I-kappaB kinase/NF-kappaB cascade	7.90E-06	79
Jejunum	Rice straw	Negative	apoptosis of blood cells	8.00E-06	207
Jejunum	Rice straw	Negative	catabolism of protein	8.07E-06	249
Jejunum	Rice straw	Negative	cell movement of macrophages	8.07E-06	116
Jejunum	Rice straw	Negative	quantity of granulocytes	8.35E-06	136
Jejunum	Rice straw	Negative	cell death of tumor	8.35E-06	162
Jejunum	Rice	Negative	morphology of lymphoid organ	8.35E-06	166

	straw				
Jejunum	Rice straw	Negative	function of endothelial cells	8.43E-06	27
Jejunum	Rice straw	Negative	hepatocellular carcinoma	8.55E-06	1574
Jejunum	Rice straw	Negative	adhesion of immune cells	8.58E-06	149
Jejunum	Rice straw	Negative	morphogenesis of neurons	8.58E-06	179
Jejunum	Rice straw	Negative	movement of vascular endothelial cells	8.67E-06	82
Jejunum	Rice straw	Negative	allodynia	8.70E-06	29
Jejunum	Rice straw	Negative	function of endothelial tissue	8.70E-06	29
Jejunum	Rice straw	Negative	branching of cells	8.77E-06	157
Jejunum	Rice straw	Negative	peripheral arterial disease	9.23E-06	94
Jejunum	Rice straw	Negative	synthesis of fatty acid	9.40E-06	134
Jejunum	Rice straw	Negative	mammary tumor	9.66E-06	640
Jejunum	Rice straw	Negative	formation of lymphatic system component	9.83E-06	126
Jejunum	Rice straw	Negative	quantity of steroid	9.90E-06	195
Jejunum	Rice straw	Negative	necrosis of tumor	1.01E-05	161
Jejunum	Rice straw	Negative	memory	1.01E-05	112
Jejunum	Rice straw	Negative	differentiation of muscle	1.04E-05	130
Jejunum	Rice straw	Negative	abnormal morphology of lymphoid organ	1.09E-05	160
Jejunum	Rice straw	Negative	craniofacial abnormality	1.16E-05	151
Jejunum	Rice straw	Negative	proliferation of tumor cells	1.18E-05	196
Jejunum	Rice straw	Negative	recruitment of macrophages	1.19E-05	51
Jejunum	Rice straw	Negative	engulfment of leukocytes	1.22E-05	73
Jejunum	Rice	Negative	neovascularization of organ	1.24E-05	53

	straw				
Jejunum	Rice straw	Negative	immune response of cells	1.27E-05	236
Jejunum	Rice straw	Negative	recruitment of antigen presenting cells	1.27E-05	57
Jejunum	Rice straw	Negative	paraproteinemia	1.28E-05	143
Jejunum	Rice straw	Negative	formation of lung	1.31E-05	129
Jejunum	Rice straw	Negative	engulfment of blood cells	1.33E-05	77
Jejunum	Rice straw	Negative	uptake of D-hexose	1.33E-05	98
Jejunum	Rice straw	Negative	adhesion of blood cells	1.33E-05	159
Jejunum	Rice straw	Negative	cell viability of cervical cancer cell lines	1.41E-05	97
Jejunum	Rice straw	Negative	uptake of D-glucose	1.41E-05	97
Jejunum	Rice straw	Negative	dilation of heart chamber	1.41E-05	31
Jejunum	Rice straw	Negative	formation of filopodia	1.42E-05	75
Jejunum	Rice straw	Negative	size of animal	1.46E-05	80
Jejunum	Rice straw	Negative	apoptosis of neurons	1.48E-05	182
Jejunum	Rice straw	Negative	morphology of lymph node	1.53E-05	73
Jejunum	Rice straw	Negative	release of metal	1.57E-05	95
Jejunum	Rice straw	Negative	length of plasma membrane projections	1.57E-05	42
Jejunum	Rice straw	Negative	serine phosphorylation of peptide	1.57E-05	42
Jejunum	Rice straw	Negative	transport of monosaccharide	1.58E-05	72
Jejunum	Rice straw	Negative	transport of amino acids	1.59E-05	62
Jejunum	Rice straw	Negative	quantity of protein in blood	1.60E-05	210
Jejunum	Rice straw	Negative	peripheral vascular disease	1.62E-05	160
Jejunum	Rice	Negative	apoptosis of liver cells	1.62E-05	71

	straw				
Jejunum	Rice straw	Negative	cell death of liver	1.62E-05	104
Jejunum	Rice straw	Negative	abnormal morphology of bone	1.66E-05	182
Jejunum	Rice straw	Negative	Thrombosis	1.71E-05	76
Jejunum	Rice straw	Negative	inflammation of central nervous system	1.76E-05	129
Jejunum	Rice straw	Negative	smooth muscle tumor	1.76E-05	129
Jejunum	Rice straw	Negative	quantity of macrophages	1.76E-05	89
Jejunum	Rice straw	Negative	plasma cell dyscrasia	1.80E-05	142
Jejunum	Rice straw	Negative	cell death of sarcoma cell lines	1.81E-05	99
Jejunum	Rice straw	Negative	adenocarcinoma	1.89E-05	2405
Jejunum	Rice straw	Negative	experimentally-induced diabetes	1.92E-05	46
Jejunum	Rice straw	Negative	differentiation of tumor cell lines	1.98E-05	159
Jejunum	Rice straw	Negative	MAPKKK cascade	2.04E-05	90
Jejunum	Rice straw	Negative	proliferation of T lymphocytes	2.06E-05	247
Jejunum	Rice straw	Negative	hypertrophy of cardiac muscle	2.06E-05	71
Jejunum	Rice straw	Negative	necrosis of liver	2.06E-05	103
Jejunum	Rice straw	Negative	morphology of endothelial tissue	2.10E-05	41
Jejunum	Rice straw	Negative	abnormal morphology of embryonic tissue	2.13E-05	225
Jejunum	Rice straw	Negative	growth of lymphatic system component	2.15E-05	85
Jejunum	Rice straw	Negative	angiogenesis of lesion	2.15E-05	59
Jejunum	Rice straw	Negative	cell spreading of tumor cell lines	2.16E-05	51
Jejunum	Rice straw	Negative	apoptosis of neuroblastoma cell lines	2.19E-05	57
Jejunum	Rice	Negative	cell movement of lymphocytes	2.19E-05	165

	straw				
Jejunum	Rice straw	Negative	angiogenesis of tumor	2.19E-05	56
Jejunum	Rice straw	Negative	epileptic seizure	2.19E-05	75
Jejunum	Rice straw	Negative	remodeling of bone	2.24E-05	84
Jejunum	Rice straw	Negative	apoptosis of fibroblasts	2.24E-05	95
Jejunum	Rice straw	Negative	invasion of carcinoma cell lines	2.36E-05	87
Jejunum	Rice straw	Negative	morphology of blood cells	2.38E-05	173
Jejunum	Rice straw	Negative	cell death of brain	2.41E-05	124
Jejunum	Rice straw	Negative	liver cancer	2.50E-05	1617
Jejunum	Rice straw	Negative	blood protein disorder	2.57E-05	157
Jejunum	Rice straw	Negative	mass of heart	2.65E-05	62
Jejunum	Rice straw	Negative	contractility of myocardium	2.68E-05	32
Jejunum	Rice straw	Negative	degranulation	2.75E-05	98
Jejunum	Rice straw	Negative	cell death of B-lymphocyte derived cell lines	2.81E-05	59
Jejunum	Rice straw	Negative	differentiation of embryonic tissue	2.84E-05	109
Jejunum	Rice straw	Negative	resorption of bone	2.87E-05	74
Jejunum	Rice straw	Negative	hepatobiliary system cancer	2.97E-05	1623
Jejunum	Rice straw	Negative	abdominal adenocarcinoma	3.00E-05	2188
Jejunum	Rice straw	Negative	contractility of heart	3.00E-05	78
Jejunum	Rice straw	Negative	Encephalitis	3.09E-05	125
Jejunum	Rice straw	Negative	differentiation of T lymphocytes	3.12E-05	158
Jejunum	Rice straw	Negative	serous neoplasm	3.13E-05	248
Jejunum	Rice	Negative	liver tumor	3.15E-05	1626

	straw				
Jejunum	Rice straw	Negative	muscle tumor	3.41E-05	151
Jejunum	Rice straw	Negative	colon cancer	3.48E-05	1389
Jejunum	Rice straw	Negative	degranulation of cells	3.51E-05	97
Jejunum	Rice straw	Negative	formation of muscle cells	3.55E-05	79
Jejunum	Rice straw	Negative	differentiation of osteoblasts	3.60E-05	118
Jejunum	Rice straw	Negative	abnormal morphology of blood vessel	3.61E-05	113
Jejunum	Rice straw	Negative	abnormal morphology of lymph node	3.62E-05	68
Jejunum	Rice straw	Negative	proliferation of endothelial cells	3.76E-05	131
Jejunum	Rice straw	Negative	permeability of cells	3.76E-05	47
Jejunum	Rice straw	Negative	atherogenesis	3.80E-05	39
Jejunum	Rice straw	Negative	hyperplasia of tissue	3.92E-05	85
Jejunum	Rice straw	Negative	morphogenesis of neurites	3.92E-05	173
Jejunum	Rice straw	Negative	leiomyomatosis	4.03E-05	121
Jejunum	Rice straw	Negative	function of blood vessel	4.03E-05	29
Jejunum	Rice straw	Negative	release of L-amino acid	4.03E-05	40
Jejunum	Rice straw	Negative	apoptosis of leukocytes	4.03E-05	188
Jejunum	Rice straw	Negative	quantity of cellular protrusions	4.04E-05	71
Jejunum	Rice straw	Negative	polymerization of protein	4.08E-05	154
Jejunum	Rice straw	Negative	engulfment of myeloid cells	4.11E-05	64
Jejunum	Rice straw	Negative	endocytosis	4.15E-05	144
Jejunum	Rice straw	Negative	release of Ca2+	4.34E-05	90
Jejunum	Rice	Negative	cell movement of breast cancer cell lines	4.35E-05	122

	straw				
Jejunum	Rice straw	Negative	replication of Influenza virus	4.35E-05	122
Jejunum	Rice straw	Negative	degranulation of phagocytes	4.36E-05	69
Jejunum	Rice straw	Negative	chemotaxis of neutrophils	4.41E-05	74
Jejunum	Rice straw	Negative	differentiation of muscle cells	4.55E-05	119
Jejunum	Rice straw	Negative	proliferation of prostate cancer cell lines	4.55E-05	128
Jejunum	Rice straw	Negative	recruitment of neutrophils	4.79E-05	81
Jejunum	Corn stover	Positive	proliferation of cells	3.57E-33	1344
Jejunum	Corn stover	Positive	cancer	3.57E-33	3430
Jejunum	Corn stover	Positive	malignant solid tumor	1.00E-30	3383
Jejunum	Corn stover	Positive	cell death	3.08E-28	1208
Jejunum	Corn stover	Positive	necrosis	7.59E-27	960
Jejunum	Corn stover	Positive	organismal death	4.15E-25	895
Jejunum	Corn stover	Positive	morbidity or mortality	4.48E-25	905
Jejunum	Corn stover	Positive	apoptosis	3.51E-24	969
Jejunum	Corn stover	Positive	morphology of cells	3.78E-24	745
Jejunum	Corn stover	Positive	cell movement	8.24E-23	798
Jejunum	Corn stover	Positive	organization of cytoplasm	5.11E-19	587
Jejunum	Corn stover	Positive	migration of cells	1.05E-18	706
Jejunum	Corn stover	Positive	cellular homeostasis	2.75E-18	580
Jejunum	Corn stover	Positive	expression of RNA	1.38E-17	778
Jejunum	Corn stover	Positive	epithelial cancer	7.48E-17	2760
Jejunum	Corn	Positive	quantity of cells	7.61E-17	653

	stover				
Jejunum	Corn stover	Positive	transcription	9.30E-17	725
Jejunum	Corn stover	Positive	neoplasia of epithelial tissue	1.36E-16	2782
Jejunum	Corn stover	Positive	transcription of RNA	2.15E-16	679
Jejunum	Corn stover	Positive	tumorigenesis of tissue	2.15E-16	2823
Jejunum	Corn stover	Positive	cell death of tumor cell lines	2.17E-15	567
Jejunum	Corn stover	Positive	organization of cytoskeleton	2.51E-15	524
Jejunum	Corn stover	Positive	cell survival	2.81E-15	523
Jejunum	Corn stover	Positive	cell death of connective tissue cells	3.46E-15	254
Jejunum	Corn stover	Positive	differentiation of cells	6.47E-15	795
Jejunum	Corn stover	Positive	angiogenesis	8.85E-15	359
Jejunum	Corn stover	Positive	transport of molecule	9.87E-15	591
Jejunum	Corn stover	Positive	morphology of cardiovascular system	1.26E-14	258
Jejunum	Corn stover	Positive	size of body	2.30E-14	338
Jejunum	Corn stover	Positive	vasculogenesis	1.04E-13	296
Jejunum	Corn stover	Positive	Viral Infection	1.40E-13	585
Jejunum	Corn stover	Positive	cell viability	2.41E-13	483
Jejunum	Corn stover	Positive	abdominal neoplasm	2.79E-13	2737
Jejunum	Corn stover	Positive	abnormal morphology of cells	2.99E-13	471
Jejunum	Corn stover	Positive	microtubule dynamics	1.52E-12	441
Jejunum	Corn stover	Positive	abnormal morphology of cardiovascular system	1.64E-12	232
Jejunum	Corn stover	Positive	apoptosis of tumor cell lines	3.55E-12	448
Jejunum	Corn	Positive	cell movement of tumor cell lines	3.55E-12	329

	stover				
Jejunum	Corn stover	Positive	abdominal cancer	3.64E-12	2698
Jejunum	Corn stover	Positive	digestive organ tumor	3.97E-12	2374
Jejunum	Corn stover	Positive	abnormal morphology of thoracic cavity	4.53E-12	237
Jejunum	Corn stover	Positive	cell death of epithelial cells	4.98E-12	205
Jejunum	Corn stover	Positive	transcription of DNA	5.94E-12	551
Jejunum	Corn stover	Positive	digestive system cancer	6.70E-12	2351
Jejunum	Corn stover	Positive	necrosis of epithelial tissue	6.70E-12	237
Jejunum	Corn stover	Positive	quantity of blood cells	2.05E-11	384
Jejunum	Corn stover	Positive	morphology of heart	2.23E-11	170
Jejunum	Corn stover	Positive	concentration of lipid	2.93E-11	311
Jejunum	Corn stover	Positive	organization of organelle	3.46E-11	217
Jejunum	Corn stover	Positive	quantity of connective tissue	3.83E-11	239
Jejunum	Corn stover	Positive	morphology of body cavity	3.83E-11	437
Jejunum	Corn stover	Positive	abnormal morphology of body cavity	5.75E-11	419
Jejunum	Corn stover	Positive	development of vasculature	6.79E-11	173
Jejunum	Corn stover	Positive	protein kinase cascade	6.80E-11	174
Jejunum	Corn stover	Positive	quantity of leukocytes	8.17E-11	344
Jejunum	Corn stover	Positive	transactivation	8.42E-11	229
Jejunum	Corn stover	Positive	Growth Failure	1.63E-10	235
Jejunum	Corn stover	Positive	abnormal morphology of heart	1.72E-10	159
Jejunum	Corn stover	Positive	colony formation of cells	5.00E-10	196
Jejunum	Corn	Positive	growth of organism	7.07E-10	313

	stover				
Jejunum	Corn stover	Positive	metabolism of carbohydrate	7.89E-10	240
Jejunum	Corn stover	Positive	proliferation of tumor cell lines	1.17E-09	541
Jejunum	Corn stover	Positive	Organ Degeneration	1.24E-09	195
Jejunum	Corn stover	Positive	colony formation	1.24E-09	210
Jejunum	Corn stover	Positive	function of leukocytes	1.42E-09	210
Jejunum	Corn stover	Positive	growth of embryo	1.43E-09	189
Jejunum	Corn stover	Positive	proliferation of connective tissue cells	1.57E-09	229
Jejunum	Corn stover	Positive	morphology of connective tissue	1.66E-09	183
Jejunum	Corn stover	Positive	formation of cellular protrusions	1.70E-09	333
Jejunum	Corn stover	Positive	cell death of fibroblast cell lines	1.87E-09	171
Jejunum	Corn stover	Positive	differentiation of connective tissue cells	2.73E-09	245
Jejunum	Corn stover	Positive	autosomal recessive disease	2.91E-09	338
Jejunum	Corn stover	Positive	growth of connective tissue	3.47E-09	245
Jejunum	Corn stover	Positive	interphase	3.52E-09	249
Jejunum	Corn stover	Positive	migration of tumor cell lines	3.61E-09	266
Jejunum	Corn stover	Positive	Movement Disorders	3.88E-09	382
Jejunum	Corn stover	Positive	development of cytoplasm	3.93E-09	182
Jejunum	Corn stover	Positive	function of blood cells	3.93E-09	225
Jejunum	Corn stover	Positive	development of body trunk	4.73E-09	400
Jejunum	Corn stover	Positive	cell death of epithelial cell lines	5.43E-09	118
Jejunum	Corn stover	Positive	cell viability of tumor cell lines	6.11E-09	289
Jejunum	Corn	Positive	quantity of lymphocytes	1.07E-08	264

	stover				
Jejunum	Corn stover	Positive	quantity of mononuclear leukocytes	1.28E-08	273
Jejunum	Corn stover	Positive	cell death of cervical cancer cell lines	1.37E-08	141
Jejunum	Corn stover	Positive	apoptosis of fibroblast cell lines	1.37E-08	133
Jejunum	Corn stover	Positive	invasion of cells	1.58E-08	317
Jejunum	Corn stover	Positive	differentiation of connective tissue	2.16E-08	270
Jejunum	Corn stover	Positive	transactivation of RNA	2.33E-08	207
Jejunum	Corn stover	Positive	ubiquitination of protein	3.40E-08	133
Jejunum	Corn stover	Positive	function of cardiovascular system	4.00E-08	146
Jejunum	Corn stover	Positive	phosphorylation of protein	5.23E-08	270
Jejunum	Corn stover	Positive	ubiquitination	5.84E-08	134
Jejunum	Corn stover	Positive	proliferation of blood cells	5.99E-08	305
Jejunum	Corn stover	Positive	apoptosis of epithelial cell lines	5.99E-08	94
Jejunum	Corn stover	Positive	activation of cells	6.37E-08	349
Jejunum	Corn stover	Positive	growth of epithelial tissue	6.56E-08	251
Jejunum	Corn stover	Positive	development of leukocytes	7.74E-08	220
Jejunum	Corn stover	Positive	autosomal dominant disease	9.08E-08	236
Jejunum	Corn stover	Positive	apoptosis of heart cells	9.86E-08	81
Jejunum	Corn stover	Positive	development of connective tissue	1.00E-07	145
Jejunum	Corn stover	Positive	concentration of fatty acid	1.01E-07	113
Jejunum	Corn stover	Positive	quantity of T lymphocytes	1.18E-07	201
Jejunum	Corn stover	Positive	abnormal morphology of reproductive system	1.25E-07	192
Jejunum	Corn	Positive	migration of blood cells	1.36E-07	313

	stover				
Jejunum	Corn stover	Positive	development of blood cells	1.54E-07	240
Jejunum	Corn stover	Positive	cell transformation	1.65E-07	175
Jejunum	Corn stover	Positive	apoptosis of muscle	1.65E-07	106
Jejunum	Corn stover	Positive	leukocyte migration	1.66E-07	312
Jejunum	Corn stover	Positive	proliferation of fibroblast cell lines	1.90E-07	162
Jejunum	Corn stover	Positive	synthesis of nitric oxide	1.93E-07	111
Jejunum	Corn stover	Positive	cell cycle progression	1.94E-07	368
Jejunum	Corn stover	Positive	homing	1.98E-07	213
Jejunum	Corn stover	Positive	development of lymphocytes	2.08E-07	206
Jejunum	Corn stover	Positive	quantity of carbohydrate	2.13E-07	194
Jejunum	Corn stover	Positive	apoptosis of muscle cells	2.23E-07	105
Jejunum	Corn stover	Positive	formation of cells	2.34E-07	347
Jejunum	Corn stover	Positive	dyskinesia	2.34E-07	227
Jejunum	Corn stover	Positive	development of mononuclear leukocytes	2.51E-07	207
Jejunum	Corn stover	Positive	morphology of nervous system	2.58E-07	282
Jejunum	Corn stover	Positive	activation of blood cells	2.61E-07	270
Jejunum	Corn stover	Positive	proliferation of neuronal cells	2.67E-07	213
Jejunum	Corn stover	Positive	synthesis of lipid	2.75E-07	259
Jejunum	Corn stover	Positive	apoptosis of cardiomyocytes	2.76E-07	78
Jejunum	Corn stover	Positive	perinatal death	2.81E-07	219
Jejunum	Corn stover	Positive	abnormal morphology of abdomen	2.87E-07	312
Jejunum	Corn	Positive	function of muscle	2.98E-07	135

	stover				
Jejunum	Corn stover	Positive	cell movement of leukocytes	3.03E-07	276
Jejunum	Corn stover	Positive	behavior	3.23E-07	348
Jejunum	Corn stover	Positive	proliferation of immune cells	3.32E-07	284
Jejunum	Corn stover	Positive	activation of DNA endogenous promoter	3.32E-07	418
Jejunum	Corn stover	Positive	transport of carbohydrate	3.44E-07	81
Jejunum	Corn stover	Positive	morphology of blood vessel	3.62E-07	112
Jejunum	Corn stover	Positive	homing of cells	3.80E-07	206
Jejunum	Corn stover	Positive	cell death of immune cells	3.86E-07	240
Jejunum	Corn stover	Positive	cell movement of myeloid cells	3.88E-07	200
Jejunum	Corn stover	Positive	cell death of kidney cell lines	4.04E-07	119
Jejunum	Corn stover	Positive	cell death of blood cells	4.05E-07	251
Jejunum	Corn stover	Positive	congenital anomaly of musculoskeletal system	4.18E-07	265
Jejunum	Corn stover	Positive	proliferation of embryonic cells	4.24E-07	100
Jejunum	Corn stover	Positive	uptake of monosaccharide	4.40E-07	107
Jejunum	Corn stover	Positive	morphology of reproductive system	4.45E-07	198
Jejunum	Corn stover	Positive	morphology of vessel	4.71E-07	120
Jejunum	Corn stover	Positive	degeneration of nervous system	5.00E-07	113
Jejunum	Corn stover	Positive	abnormal morphology of head	5.18E-07	296
Jejunum	Corn stover	Positive	activation of leukocytes	5.72E-07	252
Jejunum	Corn stover	Positive	neuromuscular disease	5.87E-07	312
Jejunum	Corn stover	Positive	neuronal cell death	6.26E-07	246
Jejunum	Corn	Positive	neurological signs	6.29E-07	236

	stover				
Jejunum	Corn stover	Positive	growth of lesion	6.54E-07	284
Jejunum	Corn stover	Positive	apoptosis of epithelial cells	6.79E-07	112
Jejunum	Corn stover	Positive	chorea	6.85E-07	211
Jejunum	Corn stover	Positive	metabolism of membrane lipid derivative	7.31E-07	147
Jejunum	Corn stover	Positive	Neurodegeneration	7.46E-07	120
Jejunum	Corn stover	Positive	morphology of head	7.58E-07	309
Jejunum	Corn stover	Positive	fibrogenesis	7.58E-07	155
Jejunum	Corn stover	Positive	Huntington's Disease	7.71E-07	210
Jejunum	Corn stover	Positive	consumption of oxygen	7.83E-07	66
Jejunum	Corn stover	Positive	concentration of phospholipid	8.09E-07	76
Jejunum	Corn stover	Positive	morphology of skin	8.40E-07	107
Jejunum	Corn stover	Positive	inflammatory response	8.83E-07	277
Jejunum	Corn stover	Positive	accumulation of lipid	9.69E-07	116
Jejunum	Corn stover	Positive	mass of connective tissue	9.95E-07	82
Jejunum	Corn stover	Positive	uptake of carbohydrate	1.10E-06	113
Jejunum	Corn stover	Positive	infection by Retroviridae	1.10E-06	257
Jejunum	Corn stover	Positive	growth of tumor	1.16E-06	282
Jejunum	Corn stover	Positive	metabolism of monosaccharide	1.17E-06	54
Jejunum	Corn stover	Positive	chemotaxis	1.27E-06	199
Jejunum	Corn stover	Positive	MAPKKK cascade	1.28E-06	83
Jejunum	Corn stover	Positive	growth of muscle tissue	1.28E-06	141
Jejunum	Corn	Positive	cell movement of neutrophils	1.29E-06	115

	stover				
Jejunum	Corn stover	Positive	proliferation of fibroblasts	1.34E-06	134
Jejunum	Corn stover	Positive	synthesis of reactive oxygen species	1.35E-06	177
Jejunum	Corn stover	Positive	differentiation of leukocytes	1.36E-06	241
Jejunum	Corn stover	Positive	HIV infection	1.43E-06	254
Jejunum	Corn stover	Positive	proliferation of muscle cells	1.46E-06	140
Jejunum	Corn stover	Positive	development of lymphatic system	1.50E-06	135
Jejunum	Corn stover	Positive	formation of filaments	1.50E-06	149
Jejunum	Corn stover	Positive	disorder of lipid metabolism	1.58E-06	80
Jejunum	Corn stover	Positive	cell death of heart cells	1.59E-06	88
Jejunum	Corn stover	Positive	neuritogenesis	1.67E-06	211
Jejunum	Corn stover	Positive	proliferation of mononuclear leukocytes	1.69E-06	262
Jejunum	Corn stover	Positive	apoptosis of connective tissue cells	1.70E-06	114
Jejunum	Corn stover	Positive	differentiation of bone	1.83E-06	153
Jejunum	Corn stover	Positive	blood protein disorder	1.86E-06	141
Jejunum	Corn stover	Positive	development of cardiovascular tissue	1.89E-06	139
Jejunum	Corn stover	Positive	proliferation of lymphocytes	1.92E-06	258
Jejunum	Corn stover	Positive	apoptosis of kidney cell lines	1.94E-06	94
Jejunum	Corn stover	Positive	cell death of fibroblasts	2.02E-06	103
Jejunum	Corn stover	Positive	metabolism of hexose	2.09E-06	48
Jejunum	Corn stover	Positive	cell movement of granulocytes	2.14E-06	139
Jejunum	Corn stover	Positive	cell death of lymphocytes	2.32E-06	144
Jejunum	Corn	Positive	differentiation of mononuclear leukocytes	2.50E-06	196

	stover				
Jejunum	Corn stover	Positive	differentiation of bone cells	2.74E-06	151
Jejunum	Corn stover	Positive	autophagy	2.79E-06	140
Jejunum	Corn stover	Positive	infection of cells	2.79E-06	280
Jejunum	Corn stover	Positive	development of endothelial tissue	2.87E-06	137
Jejunum	Corn stover	Positive	formation of plasma membrane projections	3.06E-06	214
Jejunum	Corn stover	Positive	infection by RNA virus	3.14E-06	302
Jejunum	Corn stover	Positive	chemotaxis of cells	3.16E-06	191
Jejunum	Corn stover	Positive	abnormal morphology of nervous system	3.19E-06	256
Jejunum	Corn stover	Positive	apoptosis of cervical cancer cell lines	3.51E-06	108
Jejunum	Corn stover	Positive	fatty acid metabolism	3.59E-06	215
Jejunum	Corn stover	Positive	formation of cytoskeleton	3.59E-06	140
Jejunum	Corn stover	Positive	cell death of mononuclear leukocytes	3.69E-06	147
Jejunum	Corn stover	Positive	proliferation of T lymphocytes	3.83E-06	216
Jejunum	Corn stover	Positive	development of epithelial tissue	3.88E-06	187
Jejunum	Corn stover	Positive	disorder of basal ganglia	4.06E-06	269
Jejunum	Corn stover	Positive	mass of adipose tissue	4.11E-06	76
Jejunum	Corn stover	Positive	abnormal morphology of epithelial tissue	4.13E-06	154
Jejunum	Corn stover	Positive	synthesis of fatty acid	4.13E-06	118
Jejunum	Corn stover	Positive	development of lymphatic system component	4.13E-06	117
Jejunum	Corn stover	Positive	endothelial cell development	4.41E-06	132
Jejunum	Corn stover	Positive	abnormal morphology of embryonic tissue	4.42E-06	197
Jejunum	Corn	Positive	quantity of hematopoietic cells	4.44E-06	162

	stover				
Jejunum	Corn stover	Positive	morphogenesis of neurons	4.44E-06	156
Jejunum	Corn stover	Positive	Lymphocyte homeostasis	4.50E-06	196
Jejunum	Corn stover	Positive	cell death of heart	4.70E-06	89
Jejunum	Corn stover	Positive	production of reactive oxygen species	4.74E-06	136
Jejunum	Corn stover	Positive	homeostasis of leukocytes	5.07E-06	199
Jejunum	Corn stover	Positive	cell movement of phagocytes	5.39E-06	197
Jejunum	Corn stover	Positive	quantity of hematopoietic progenitor cells	5.73E-06	161
Jejunum	Corn stover	Positive	cell death of cardiomyocytes	5.76E-06	84
Jejunum	Corn stover	Positive	cell death of kidney cells	5.79E-06	130
Jejunum	Corn stover	Positive	cell death of embryonic cell lines	6.18E-06	93
Jejunum	Corn stover	Positive	ion homeostasis of cells	6.20E-06	199
Jejunum	Corn stover	Positive	necrosis of kidney	6.21E-06	135
Jejunum	Corn stover	Positive	secretory pathway	6.57E-06	78
Jejunum	Corn stover	Positive	mass of fat pad	6.58E-06	48
Jejunum	Corn stover	Positive	degeneration of cells	6.70E-06	128
Jejunum	Corn stover	Positive	cell death of muscle cells	6.85E-06	125
Jejunum	Corn stover	Positive	morphology of muscle	6.85E-06	125
Jejunum	Corn stover	Positive	metabolism of reactive oxygen species	6.87E-06	180
Jejunum	Corn stover	Positive	G1 phase	7.02E-06	143
Jejunum	Corn stover	Positive	cell viability of cervical cancer cell lines	7.20E-06	86
Jejunum	Corn stover	Positive	morphology of lymphatic system component	7.56E-06	152
Jejunum	Corn	Positive	endocytosis	7.56E-06	128

	stover				
Jejunum	Corn stover	Positive	paraproteinemia	7.77E-06	125
Jejunum	Corn stover	Positive	serous neoplasm	7.83E-06	216
Jejunum	Corn stover	Positive	abnormal morphology of blood vessel	7.93E-06	101
Jejunum	Corn stover	Positive	synthesis of DNA	8.49E-06	152
Jejunum	Corn stover	Positive	apoptosis of blood cells	8.92E-06	178
Jejunum	Corn stover	Positive	mass of organism	8.95E-06	124
Jejunum	Corn stover	Positive	small GTPase mediated signal transduction	9.02E-06	73
Jejunum	Corn stover	Positive	cellular infiltration by leukocytes	9.02E-06	142
Jejunum	Corn stover	Positive	survival of organism	9.27E-06	246
Jejunum	Corn stover	Positive	size of animal	9.88E-06	71
Jejunum	Corn stover	Positive	apoptosis of leukocytes	9.92E-06	165
Jejunum	Corn stover	Positive	female genital tract serous cancer	1.04E-05	141
Jejunum	Corn stover	Positive	quantity of adipose tissue	1.10E-05	105
Jejunum	Corn stover	Positive	cell death of muscle	1.13E-05	127
Jejunum	Corn stover	Positive	plasma cell dyscrasia	1.17E-05	124
Jejunum	Corn stover	Positive	infection by HIV-1	1.17E-05	215
Jejunum	Corn stover	Positive	transport of monosaccharide	1.17E-05	64
Jejunum	Corn stover	Positive	differentiation of lymphocytes	1.20E-05	178
Jejunum	Corn stover	Positive	invasion of tumor cell lines	1.20E-05	233
Jejunum	Corn stover	Positive	function of phagocytes	1.24E-05	114
Jejunum	Corn stover	Positive	T cell homeostasis	1.24E-05	184
Jejunum	Corn	Positive	proliferation of smooth muscle cells	1.26E-05	107

	stover				
Jejunum	Corn stover	Positive	growth of neurites	1.33E-05	166
Jejunum	Corn stover	Positive	proliferation of epithelial cells	1.41E-05	176
Jejunum	Corn stover	Positive	necrosis of muscle	1.47E-05	126
Jejunum	Corn stover	Positive	T cell development	1.61E-05	179
Jejunum	Corn stover	Positive	accumulation of cells	1.61E-05	129
Jejunum	Corn stover	Positive	growth of plasma membrane projections	1.62E-05	167
Jejunum	Corn stover	Positive	morphology of genital organ	1.64E-05	153
Jejunum	Corn stover	Positive	apoptosis of lymphocytes	1.64E-05	127
Jejunum	Corn stover	Positive	growth of embryonic tissue	1.69E-05	103
Jejunum	Corn stover	Positive	morphogenesis of neurites	1.70E-05	151
Jejunum	Corn stover	Positive	proliferation of hematopoietic cells	1.92E-05	96
Jejunum	Corn stover	Positive	sprouting	1.92E-05	140
Jejunum	Corn stover	Positive	plasticity of synapse	1.93E-05	50
Jejunum	Corn stover	Positive	arrest in interphase	1.96E-05	150
Jejunum	Corn stover	Positive	function of heart	2.05E-05	88
Jejunum	Corn stover	Positive	differentiation of blood cells	2.05E-05	289
Jejunum	Corn stover	Positive	abnormal morphology of skin	2.16E-05	92
Jejunum	Corn stover	Positive	formation of lymphoid organ	2.27E-05	99
Jejunum	Corn stover	Positive	development of neurons	2.29E-05	269
Jejunum	Corn stover	Positive	abnormal morphology of genital organ	2.35E-05	147
Jejunum	Corn stover	Positive	activation of neuroglia	2.37E-05	54
Jejunum	Corn	Positive	apoptosis of hepatocytes	2.37E-05	54

	stover				
Jejunum	Corn stover	Positive	cardiogenesis	2.38E-05	168
Jejunum	Corn stover	Positive	function of myeloid cells	2.46E-05	90
Jejunum	Corn stover	Positive	cellular infiltration of cells	2.54E-05	143
Jejunum	Corn stover	Positive	attachment of cells	2.61E-05	55
Jejunum	Corn stover	Positive	polymerization of protein	2.71E-05	134
Jejunum	Corn stover	Positive	bone mineral density	2.72E-05	68
Jejunum	Corn stover	Positive	Waldenstrom's macroglobulinemia	2.78E-05	73
Jejunum	Corn stover	Positive	quantity of thymus gland	2.81E-05	88
Jejunum	Corn stover	Positive	aggregation of blood platelets	2.88E-05	74
Jejunum	Corn stover	Positive	synthesis of carbohydrate	2.88E-05	152
Jejunum	Corn stover	Positive	atrophy of muscle	2.89E-05	59
Jejunum	Corn stover	Positive	hereditary motor and sensory neuropathy	2.95E-05	39
Jejunum	Corn stover	Positive	abnormal morphology of digestive system	3.24E-05	191
Jejunum	Corn stover	Positive	cellular infiltration	3.24E-05	156
Jejunum	Corn stover	Positive	quantity of thymocytes	3.24E-05	87
Jejunum	Corn stover	Positive	formation of thymus gland	3.25E-05	64
Jejunum	Corn stover	Positive	morphology of heart ventricle	3.44E-05	74
Jejunum	Corn stover	Positive	G1/S phase transition	3.45E-05	81
Jejunum	Corn stover	Positive	cell death of T lymphocytes	3.59E-05	115
Jejunum	Corn stover	Positive	apoptosis of mononuclear leukocytes	3.75E-05	129
Jejunum	Corn stover	Positive	exocytosis	4.01E-05	73
Jejunum	Corn	Positive	autophagy of cells	4.02E-05	98

	stover				
Jejunum	Corn stover	Positive	apoptosis of neuroblastoma cell lines	4.02E-05	50
Jejunum	Corn stover	Positive	degeneration of central nervous system	4.32E-05	53
Jejunum	Corn stover	Positive	organization of actin cytoskeleton	4.34E-05	110
Jejunum	Corn stover	Positive	genital tumor	4.51E-05	1264
Jejunum	Corn stover	Positive	dysmyelination	4.73E-05	54
Jejunum	Corn stover	Positive	transport of D-glucose	4.81E-05	57
Jejunum	Corn stover	Positive	quantity of protein in blood	4.84E-05	178
Jejunum	Corn stover	Positive	quantity of lymphoid organ	4.84E-05	112
Jejunum	Corn stover	Positive	abnormal morphology of internal genitalia	5.05E-05	126
Jejunum	Corn stover	Positive	morphology of bone	5.07E-05	159
Jejunum	Corn stover	Positive	hereditary polyneuropathy	5.10E-05	42
Jejunum	Corn stover	Positive	benign neoplasia	5.22E-05	312
Jejunum	Corn stover	Positive	vascularization	5.22E-05	88
Jejunum	Corn stover	Positive	quantity of lymphatic system component	5.29E-05	135
Jejunum	Corn stover	Positive	abnormal morphology of membrane tissue	5.29E-05	66
Jejunum	Corn stover	Positive	cellular degradation	5.31E-05	94
Jejunum	Corn stover	Positive	proliferation of endothelial cells	5.37E-05	113
Jejunum	Corn stover	Positive	hypersensitive reaction	5.43E-05	152
Jejunum	Corn stover	Positive	I-kappaB kinase/NF-kappaB cascade	5.48E-05	67
Jejunum	Corn stover	Positive	polyneuropathy	5.97E-05	45
Jejunum	Corn stover	Positive	proliferation of lymphatic system cells	6.07E-05	76
Jejunum	Corn	Positive	proliferation of hematopoietic progenitor cells	6.10E-05	90

	stover				
Jejunum	Corn stover	Positive	targeting of protein	6.10E-05	52
Jejunum	Corn stover	Positive	S phase	6.10E-05	96
Jejunum	Corn stover	Positive	abnormal morphology of bone	6.32E-05	154
Jejunum	Corn stover	Positive	metabolism of D-hexose	6.32E-05	40
Jejunum	Corn stover	Positive	morphology of digestive system	6.32E-05	198
Jejunum	Corn stover	Positive	formation of muscle	6.36E-05	141
Jejunum	Corn stover	Positive	function of blood vessel	6.74E-05	26
Jejunum	Corn stover	Positive	accumulation of blood cells	6.79E-05	102
Jejunum	Corn stover	Positive	cell death of liver cells	6.79E-05	74
Jejunum	Corn stover	Positive	size of bone	6.81E-05	101
Jejunum	Corn stover	Positive	cell death of hepatocytes	6.95E-05	60
Jejunum	Corn stover	Positive	long-term potentiation	6.96E-05	90
Jejunum	Corn stover	Positive	recruitment of phagocytes	6.96E-05	91
Jejunum	Corn stover	Positive	function of smooth muscle	6.96E-05	39
Jejunum	Corn stover	Positive	lysosomal storage disease	6.96E-05	39
Jejunum	Corn stover	Positive	adhesion of immune cells	7.08E-05	125
Jejunum	Corn stover	Positive	abnormal morphology of gonad	7.18E-05	130
Jejunum	Corn stover	Positive	formation of lymphatic system component	7.38E-05	106
Jejunum	Corn stover	Positive	abnormal morphology of muscle	7.57E-05	114
Jejunum	Corn stover	Positive	quantity of interleukin	7.57E-05	55
Jejunum	Corn stover	Positive	concentration of acylglycerol	7.73E-05	120
Jejunum	Corn	Positive	metabolism of D-glucose	7.73E-05	38

	stover				
Jejunum	Corn stover	Positive	mitosis	7.90E-05	173
Jejunum	Corn stover	Positive	apoptosis of embryonic cell lines	8.28E-05	69
Jejunum	Corn stover	Positive	size of embryo	8.59E-05	127
Jejunum	Corn stover	Positive	hydrolysis of lipid	8.77E-05	71
Jejunum	Corn stover	Positive	differentiation of muscle	9.23E-05	109
Jejunum	Corn stover	Positive	morphology of gonad	9.23E-05	134
Jejunum	Corn stover	Positive	cell death of liver	9.23E-05	88
Jejunum	Corn stover	Positive	neonatal death	9.30E-05	154
Jejunum	Corn stover	Positive	activation of lymphocytes	9.30E-05	161
Jejunum	Corn stover	Positive	familial dementia	9.35E-05	22
Jejunum	Corn stover	Positive	peripheral neuropathy	9.63E-05	59
Jejunum	Corn stover	Positive	morphology of central nervous system	9.79E-05	178
Jejunum	Corn stover	Positive	branching of cells	9.79E-05	131
Jejunum	Corn stover	Positive	differentiation of muscle cells	9.90E-05	102
Jejunum	Corn stover	Positive	demyelination	1.03E-04	48
Jejunum	Corn stover	Positive	hepatic steatosis	1.03E-04	97
Jejunum	Corn stover	Positive	quantity of B lymphocytes	1.03E-04	123
Jejunum	Corn stover	Positive	morphology of blood cells	1.03E-04	146
Jejunum	Corn stover	Positive	morphology of skull	1.03E-04	83
Jejunum	Corn stover	Positive	transformation of fibroblast cell lines	1.04E-04	94
Jejunum	Corn stover	Positive	apoptosis of liver	1.05E-04	61
Jejunum	Corn	Positive	formation of vascular lesion	1.05E-04	46

	stover				
Jejunum	Corn stover	Positive	quantity of IgM	1.05E-04	54
Jejunum	Corn stover	Positive	differentiation of muscle cell lines	1.09E-04	62
Jejunum	Corn stover	Positive	activation of mononuclear leukocytes	1.09E-04	168
Jejunum	Corn stover	Positive	function of vascular smooth muscle	1.09E-04	16
Jejunum	Corn stover	Positive	engulfment of cells	1.10E-04	143
Jejunum	Corn stover	Positive	morphology of lymphoid organ	1.13E-04	138
Jejunum	Corn stover	Positive	cell movement of fibroblasts	1.14E-04	69
Jejunum	Corn stover	Positive	accumulation of leukocytes	1.16E-04	97
Jejunum	Corn stover	Positive	abnormal morphology of skull	1.17E-04	81
Jejunum	Corn stover	Positive	metabolism of protein	1.18E-04	324
Jejunum	Corn stover	Positive	binding of DNA	1.18E-04	180
Jejunum	Corn stover	Positive	necrosis of liver	1.19E-04	87
Jejunum	Corn stover	Positive	export of molecule	1.19E-04	106
Jejunum	Corn stover	Positive	dyslipidemia	1.19E-04	43
Jejunum	Corn stover	Positive	concentration of phosphatidic acid	1.19E-04	53
Jejunum	Corn stover	Positive	growth of lymphatic system component	1.20E-04	72
Jejunum	Corn stover	Positive	outgrowth of neurons	1.20E-04	143
Jejunum	Corn stover	Positive	death of embryo	1.31E-04	58
Jejunum	Corn stover	Positive	transport of protein	1.31E-04	108
Jejunum	Corn stover	Positive	migration of connective tissue cells	1.31E-04	68
Jejunum	Corn stover	Positive	arrest in proliferation of cells	1.33E-04	79
Jejunum	Corn	Positive	internalization of cells	1.34E-04	63

	stover				
Jejunum	Corn stover	Positive	uptake of D-hexose	1.35E-04	82
Jejunum	Corn stover	Positive	seizure disorder	1.36E-04	163
Jejunum	Corn stover	Positive	abnormal morphology of left ventricle	1.37E-04	35
Jejunum	Corn stover	Positive	abnormal morphology of lymphoid organ	1.39E-04	133
Jejunum	Corn stover	Positive	transmigration of cells	1.43E-04	60
Jejunum	Corn stover	Positive	abnormal morphology of heart ventricle	1.47E-04	66
Jejunum	Corn stover	Positive	expansion of cells	1.47E-04	108
Jejunum	Corn stover	Positive	neurodegeneration of central nervous system	1.48E-04	45
Jejunum	Corn stover	Positive	abnormal morphology of hepatobiliary system	1.50E-04	76
Jejunum	Corn stover	Positive	biosynthesis of nucleoside triphosphate	1.51E-04	43
Jejunum	Corn stover	Positive	quantity of cytokine	1.54E-04	92
Jejunum	Corn stover	Positive	apoptosis of fibroblasts	1.54E-04	80
Jejunum	Corn stover	Positive	uptake of D-glucose	1.55E-04	81
Jejunum	Corn stover	Positive	activation of enzyme	1.59E-04	136
Jejunum	Corn stover	Positive	morphology of left ventricle	1.63E-04	36
Jejunum	Corn stover	Positive	migration of fibroblasts	1.64E-04	55
Jejunum	Corn stover	Positive	degeneration of brain	1.68E-04	49
Jejunum	Corn stover	Positive	abnormal morphology of pericardium	1.70E-04	38
Jejunum	Corn stover	Positive	cleavage of lipid	1.70E-04	74
Jejunum	Corn stover	Positive	outgrowth of neurites	1.71E-04	141
Jejunum	Corn stover	Positive	skin abnormality	1.81E-04	83
Jejunum	Corn	Positive	metabolism of DNA	1.81E-04	139

	stover				
Jejunum	Corn stover	Positive	demyelinating peripheral neuropathy	1.84E-04	28
Jejunum	Corn stover	Positive	formation of eye	1.87E-04	152
Jejunum	Corn stover	Positive	adenoma	1.88E-04	189
Jejunum	Corn stover	Positive	abnormal morphology of liver	1.92E-04	71
Jejunum	Corn stover	Positive	concentration of ATP	1.92E-04	48
Jejunum	Corn stover	Positive	migration of carcinoma cell lines	1.94E-04	64
Jejunum	Corn stover	Positive	cognition	1.94E-04	157
Jejunum	Corn stover	Positive	biosynthesis of purine ribonucleotide	1.94E-04	41
Jejunum	Corn stover	Positive	chemotaxis of myeloid cells	1.97E-04	104
Jejunum	Corn stover	Positive	seizures	1.99E-04	139
Jejunum	Corn stover	Positive	branching of neurons	2.03E-04	102
Jejunum	Corn stover	Positive	hydrolysis of phosphoinositide	2.04E-04	26
Jejunum	Corn stover	Positive	learning	2.05E-04	145
Jejunum	Corn stover	Positive	uterine serous papillary cancer	2.06E-04	84
Jejunum	Corn stover	Positive	neurodegeneration of brain	2.10E-04	44
Jejunum	Corn stover	Positive	cell movement of endothelial cells	2.10E-04	124
Jejunum	Corn stover	Positive	Hypertrophy	2.12E-04	163
Jejunum	Corn stover	Positive	Bleeding	2.13E-04	147
Jejunum	Corn stover	Positive	activation of antigen presenting cells	2.16E-04	112
Jejunum	Corn stover	Positive	permeability of cells	2.19E-04	40
Jejunum	Corn stover	Positive	secretion of molecule	2.27E-04	188
Jejunum	Corn	Positive	cell death of tumor	2.32E-04	133

	stover				
Jejunum	Corn stover	Positive	apoptosis of liver cells	2.34E-04	59
Jejunum	Corn stover	Positive	Cytosis	2.39E-04	113
Jejunum	Corn stover	Positive	replication of virus	2.42E-04	191
Jejunum	Corn stover	Positive	growth of liver	2.59E-04	70
Jejunum	Corn stover	Positive	concentration of sterol	2.65E-04	108
Jejunum	Corn stover	Positive	replication of RNA virus	2.66E-04	173
Jejunum	Corn stover	Positive	hepatocellular carcinoma	2.69E-04	1296
Jejunum	Corn stover	Positive	degeneration of neurons	2.71E-04	86
Jejunum	Corn stover	Positive	recruitment of macrophages	2.76E-04	42
Jejunum	Corn stover	Positive	morphology of epidermis	2.77E-04	54
Jejunum	Corn stover	Positive	cell viability of connective tissue cells	2.88E-04	51
Jejunum	Corn stover	Positive	glycolysis	2.88E-04	51
Jejunum	Corn stover	Positive	abnormal morphology of testis	2.93E-04	93
Jejunum	Corn stover	Positive	necrosis of tumor	2.93E-04	132
Jejunum	Corn stover	Positive	function of lymphocytes	2.94E-04	119
Jejunum	Corn stover	Positive	proliferation of tumor cells	2.97E-04	161
Jejunum	Corn stover	Positive	atherogenesis	2.97E-04	33
Jejunum	Corn stover	Positive	development of hematopoietic system	3.03E-04	89
Jejunum	Corn stover	Positive	differentiation of osteoclasts	3.04E-04	71
Jejunum	Corn stover	Positive	quantity of antigen presenting cells	3.10E-04	98
Jejunum	Corn stover	Positive	concentration of Ca2+	3.10E-04	49
Jejunum	Corn	Positive	quantity of connective tissue cells	3.10E-04	85

	stover				
Jejunum	Corn stover	Positive	abnormal morphology of lymph node	3.10E-04	57
Jejunum	Corn stover	Positive	metabolism of glycolipid	3.10E-04	57
Jejunum	Corn stover	Positive	chemotaxis of phagocytes	3.15E-04	104
Jejunum	Corn stover	Positive	metabolism of nucleoside triphosphate	3.15E-04	53
Jejunum	Corn stover	Positive	production of protein	3.18E-04	115
Jejunum	Corn stover	Positive	quantity of phagocytes	3.19E-04	146
Jejunum	Corn stover	Positive	shape change of neurons	3.19E-04	103
Jejunum	Corn stover	Positive	quantity of double-positive thymocyte	3.26E-04	44
Jejunum	Corn stover	Positive	thickness of connective tissue	3.28E-04	23
Jejunum	Corn stover	Positive	formation of actin filaments	3.34E-04	107
Jejunum	Corn stover	Positive	morphology of lymph node	3.40E-04	60
Jejunum	Corn stover	Positive	apoptosis of T lymphocytes	3.40E-04	99
Jejunum	Corn stover	Positive	proliferation of liver cells	3.42E-04	69
Jejunum	Corn stover	Positive	branching of neurites	3.45E-04	98
Jejunum	Corn stover	Positive	Dermatitis	3.50E-04	149
Jejunum	Corn stover	Positive	length of cells	3.51E-04	40
Jejunum	Corn stover	Positive	adenocarcinoma	3.52E-04	1984
Jejunum	Corn stover	Positive	abnormal morphology of skeleton	3.54E-04	96
Jejunum	Corn stover	Positive	cell movement of connective tissue cells	3.56E-04	80
Jejunum	Corn stover	Positive	weight gain	3.56E-04	109
Jejunum	Corn stover	Positive	proliferation of myeloid cells	3.79E-04	46
Jejunum	Corn	Positive	cell death of breast cancer cell lines	3.84E-04	116

	stover				
Jejunum	Corn stover	Positive	function of cardiac muscle	3.91E-04	67
Jejunum	Corn stover	Positive	formation of leukocytes	3.92E-04	59
Jejunum	Corn stover	Positive	breast or colorectal cancer	3.93E-04	1527
Jejunum	Corn stover	Positive	quantity of polyunsaturated fatty acids	4.06E-04	47
Jejunum	Corn stover	Positive	female genital neoplasm	4.07E-04	1104
Jejunum	Corn stover	Positive	adhesion of blood cells	4.09E-04	130
Jejunum	Corn stover	Positive	hereditary neuropathy	4.11E-04	61
Jejunum	Corn stover	Positive	phosphorylation of L-amino acid	4.11E-04	79
Jejunum	Corn stover	Positive	dyspnea	4.15E-04	51
Jejunum	Corn stover	Positive	development of digestive system	4.27E-04	132
Jejunum	Corn stover	Positive	metabolism of glycosphingolipid	4.30E-04	48
Jejunum	Corn stover	Positive	entrance of Ca2+	4.30E-04	32
Jejunum	Corn stover	Positive	quantity of embryonic cells	4.30E-04	32
Jejunum	Corn stover	Positive	length of long bones	4.31E-04	25
Jejunum	Corn stover	Positive	metabolism of sphingolipid	4.32E-04	52
Jejunum	Corn stover	Positive	stress response of cells	4.32E-04	52
Jejunum	Corn stover	Positive	accumulation of sterol	4.33E-04	29
Jejunum	Corn stover	Positive	cell viability of cerebral cortex cells	4.33E-04	29
Jejunum	Corn stover	Positive	infection of embryonic cell lines	4.38E-04	97
Jejunum	Corn stover	Positive	infection of epithelial cell lines	4.38E-04	97
Jejunum	Corn stover	Positive	function of antigen presenting cells	4.41E-04	87
Jejunum	Corn	Positive	development of abdomen	4.54E-04	195

	stover				
Jejunum	Corn stover	Negative	morbidity or mortality	6.17E-16	514
Jejunum	Corn stover	Negative	organismal death	6.17E-16	507
Jejunum	Corn stover	Negative	cell death	2.90E-15	665
Jejunum	Corn stover	Negative	malignant solid tumor	3.30E-15	1827
Jejunum	Corn stover	Negative	cancer	4.90E-14	1841
Jejunum	Corn stover	Negative	apoptosis	1.32E-13	537
Jejunum	Corn stover	Negative	differentiation of cells	6.05E-12	461
Jejunum	Corn stover	Negative	proliferation of cells	6.65E-12	704
Jejunum	Corn stover	Negative	necrosis	1.18E-11	514
Jejunum	Corn stover	Negative	organization of cytoplasm	1.63E-11	331
Jejunum	Corn stover	Negative	organization of cytoskeleton	2.27E-10	301
Jejunum	Corn stover	Negative	morphology of cells	4.66E-10	397
Jejunum	Corn stover	Negative	cell movement	5.92E-10	428
Jejunum	Corn stover	Negative	transcription	3.27E-09	401
Jejunum	Corn stover	Negative	transport of molecule	4.78E-09	333
Jejunum	Corn stover	Negative	quantity of cells	8.82E-09	359
Jejunum	Corn stover	Negative	expression of RNA	1.03E-08	424
Jejunum	Corn stover	Negative	microtubule dynamics	1.31E-08	254
Jejunum	Corn stover	Negative	vasculogenesis	1.74E-08	170
Jejunum	Corn stover	Negative	transcription of RNA	2.14E-08	372
Jejunum	Corn stover	Negative	migration of cells	6.73E-08	377
Jejunum	Corn	Negative	seizure disorder	3.14E-07	113

	stover				
Jejunum	Corn stover	Negative	proliferation of connective tissue cells	3.69E-07	137
Jejunum	Corn stover	Negative	angiogenesis	4.85E-07	196
Jejunum	Corn stover	Negative	growth of connective tissue	6.15E-07	146
Jejunum	Corn stover	Negative	tumorigenesis of tissue	7.19E-07	1516
Jejunum	Corn stover	Negative	phosphorylation of protein	7.21E-07	163
Jejunum	Corn stover	Negative	formation of cellular protrusions	7.97E-07	194
Jejunum	Corn stover	Negative	organization of organelle	7.99E-07	125
Jejunum	Corn stover	Negative	organization of actin cytoskeleton	8.46E-07	77
Jejunum	Corn stover	Negative	growth of organism	1.04E-06	181
Jejunum	Corn stover	Negative	Viral Infection	1.10E-06	319
Jejunum	Corn stover	Negative	abdominal neoplasm	1.19E-06	1480
Jejunum	Corn stover	Negative	transcription of DNA	1.36E-06	305
Jejunum	Corn stover	Negative	seizures	1.58E-06	96
Jejunum	Corn stover	Negative	cellular homeostasis	1.62E-06	302
Jejunum	Corn stover	Negative	neoplasia of epithelial tissue	1.62E-06	1489
Jejunum	Corn stover	Negative	cell death of tumor cell lines	2.08E-06	302
Jejunum	Corn stover	Negative	epithelial cancer	3.48E-06	1473
Jejunum	Corn stover	Negative	quantity of blood cells	5.73E-06	212
Jejunum	Corn stover	Negative	growth of embryo	7.12E-06	109
Jejunum	Corn stover	Negative	cell death of connective tissue cells	7.84E-06	133
Jejunum	Corn stover	Negative	morphology of cardiovascular system	8.63E-06	136
Jejunum	Corn	Negative	proteinuria	1.10E-05	45

	stover				
Jejunum	Corn stover	Negative	quantity of leukocytes	1.28E-05	190
Jejunum	Corn stover	Negative	fibrogenesis	1.40E-05	95
Jejunum	Corn stover	Negative	formation of filaments	1.72E-05	92
Jejunum	Corn stover	Negative	synthesis of lipid	2.05E-05	152
Jejunum	Corn stover	Negative	organization of filaments	2.53E-05	53
Jejunum	Corn stover	Negative	invasion of cells	2.53E-05	180
Jejunum	Corn stover	Negative	abdominal cancer	2.72E-05	1451
Jejunum	Corn stover	Negative	development of epithelial tissue	3.01E-05	114
Jejunum	Corn stover	Negative	cell movement of tumor cell lines	3.34E-05	176
Jejunum	Corn stover	Negative	morphology of urinary system	3.80E-05	65
Jejunum	Corn stover	Negative	endothelial cell development	3.84E-05	82
Jejunum	Corn stover	Negative	neuritogenesis	3.92E-05	126
Jejunum	Corn stover	Negative	development of cardiovascular tissue	4.00E-05	85
Jejunum	Corn stover	Negative	growth of epithelial tissue	4.00E-05	144
Jejunum	Corn stover	Negative	metabolism of carbohydrate	4.00E-05	133
Jejunum	Corn stover	Negative	apoptosis of tumor cell lines	4.00E-05	239
Jejunum	Corn stover	Negative	neuromuscular disease	4.04E-05	181
Jejunum	Corn stover	Negative	abnormal morphology of urinary system	4.82E-05	62
Jejunum	Corn stover	Negative	cell survival	6.65E-05	269
Jejunum	Corn stover	Negative	Movement Disorders	6.93E-05	211
Jejunum	Corn stover	Negative	protein kinase cascade	7.14E-05	94
Jejunum	Corn	Negative	formation of plasma membrane projections	7.64E-05	127

	stover				
Jejunum	Corn stover	Negative	morphology of kidney	7.64E-05	60
Jejunum	Corn stover	Negative	assembly of protein-protein complex	7.69E-05	48
Jejunum	Corn stover	Negative	formation of cytoskeleton	7.99E-05	85
Jejunum	Corn stover	Negative	development of endothelial tissue	8.09E-05	83
Jejunum	Corn stover	Negative	concentration of lipid	8.77E-05	166
Jejunum	Corn stover	Negative	Growth Failure	8.77E-05	127
Jejunum	Corn stover	Negative	abnormal morphology of cardiovascular system	9.74E-05	121
Jejunum	Corn stover	Negative	abnormal morphology of kidney	1.00E-04	57
Jejunum	Corn stover	Negative	differentiation of epithelial tissue	1.25E-04	81
Jejunum	Corn stover	Negative	development of body trunk	1.38E-04	219
Jejunum	Corn stover	Negative	digestive organ tumor	1.44E-04	1268
Jejunum	Corn stover	Negative	ion homeostasis of cells	1.49E-04	118
Jejunum	Corn stover	Negative	digestive system cancer	1.68E-04	1256
Jejunum	Corn stover	Negative	homing of cells	1.68E-04	118
Jejunum	Corn stover	Negative	development of cytoplasm	1.71E-04	100
Jejunum	Corn stover	Negative	autosomal recessive disease	1.75E-04	184
Jejunum	Corn stover	Negative	proliferation of endothelial cells	1.82E-04	71
Jejunum	Corn stover	Negative	formation of actin stress fibers	1.85E-04	57
Jejunum	Corn stover	Negative	morphology of heart	1.93E-04	89
Jejunum	Corn stover	Negative	apoptosis of fibroblast cell lines	2.11E-04	74
Jejunum	Corn stover	Negative	degeneration of nervous system	2.11E-04	66
Jejunum	Corn	Negative	differentiation of connective tissue cells	2.26E-04	133

	stover				
Jejunum	Corn stover	Negative	morphology of nervous system	2.95E-04	158
Jejunum	Corn stover	Negative	migration of blood cells	2.95E-04	174
Jejunum	Corn stover	Negative	abnormal morphology of thoracic cavity	3.06E-04	122
Jejunum	Corn stover	Negative	chemotaxis of cells	3.06E-04	111
Jejunum	Corn stover	Negative	cell viability	3.06E-04	248
Jejunum	Corn stover	Negative	cell death of fibroblast cell lines	3.40E-04	92
Jejunum	Corn stover	Negative	cell movement of endothelial cells	3.49E-04	78
Jejunum	Corn stover	Negative	transactivation	3.65E-04	120
Jejunum	Corn stover	Negative	leukocyte migration	3.79E-04	173
Jejunum	Corn stover	Negative	epilepsy	3.84E-04	69
Jejunum	Corn stover	Negative	quantity of metal	3.93E-04	105
Jejunum	Corn stover	Negative	homing	3.93E-04	119
Jejunum	Corn stover	Negative	endocytosis	4.09E-04	76
Jejunum	Corn stover	Negative	exocytosis	4.18E-04	46
Jejunum	Corn stover	Negative	Neurodegeneration	4.45E-04	69
Jejunum	Corn stover	Negative	differentiation of connective tissue	4.59E-04	147
Jejunum	Corn stover	Negative	migration of connective tissue cells	4.66E-04	44
Jejunum	Corn stover	Negative	morphology of head	4.72E-04	173
Jejunum	Corn stover	Negative	congenital anomaly of musculoskeletal system	4.72E-04	148
Jejunum	Corn stover	Negative	formation of actin filaments	4.72E-04	68
Jejunum	Corn stover	Negative	chemotaxis	5.05E-04	113
Jejunum	Corn	Negative	invasion of tumor cell lines	5.05E-04	135

	stover				
Jejunum	Corn stover	Negative	ubiquitination	5.05E-04	74
Jejunum	Corn stover	Negative	cell death of macrophages	5.05E-04	33
Jejunum	Corn stover	Negative	urination disorder	5.05E-04	65
Jejunum	Corn stover	Negative	ubiquitination of protein	5.05E-04	73
Jejunum	Corn stover	Negative	morphology of body cavity	5.11E-04	228
Jejunum	Corn stover	Negative	synthesis of DNA	5.32E-04	89
Jejunum	Corn stover	Negative	cell death of antigen presenting cells	5.32E-04	40
Jejunum	Corn stover	Negative	function of cardiovascular system	5.44E-04	80
Jejunum	Corn stover	Negative	transmigration of cells	5.51E-04	39
Jejunum	Corn stover	Negative	cell death of myeloid cells	5.56E-04	50
Jejunum	Corn stover	Negative	learning	5.57E-04	89
Jejunum	Corn stover	Negative	migration of tumor cell lines	5.75E-04	142
Jejunum	Corn stover	Negative	quantity of lymphocytes	6.05E-04	142
Jejunum	Corn stover	Negative	proliferation of neuronal cells	6.45E-04	118
Jejunum	Corn stover	Negative	abnormal morphology of abdomen	6.49E-04	172
Jejunum	Corn stover	Negative	quantity of lymphatic system component	6.95E-04	81
Jejunum	Corn stover	Negative	cell death of phagocytes	7.30E-04	49
Jejunum	Corn stover	Negative	quantity of carbohydrate	7.56E-04	107
Jejunum	Corn stover	Negative	cognition	8.28E-04	95
Jejunum	Corn stover	Negative	proliferation of fibroblasts	8.40E-04	76
Jejunum	Corn stover	Negative	proliferation of lymphatic system cells	8.40E-04	47
Jejunum	Corn	Negative	quantity of mononuclear leukocytes	8.92E-04	146

	stover				
Jejunum	Corn stover	Negative	morphology of connective tissue	8.92E-04	96
Jejunum	Corn stover	Negative	infection of cells	9.25E-04	157
Jejunum	Corn stover	Negative	binding of DNA	9.35E-04	107
Jejunum	Corn stover	Negative	function of blood cells	9.35E-04	119
Jejunum	Corn stover	Negative	development of vasculature	9.53E-04	88
Jejunum	Corn stover	Negative	proliferation of embryonic cells	9.53E-04	56
Jejunum	Corn stover	Negative	migration of endothelial cells	9.72E-04	71
Jejunum	Corn stover	Negative	transport of protein	9.72E-04	66
Jejunum	Corn stover	Negative	transactivation of RNA	9.72E-04	111
Jejunum	Corn stover	Negative	quantity of lymphoid organ	1.04E-03	67
Jejunum	Corn stover	Negative	disorder of basal ganglia	1.09E-03	151
Jejunum	Corn stover	Negative	concentration of acylglycerol	1.09E-03	72
Jejunum	Corn stover	Negative	activation of DNA endogenous promoter	1.09E-03	228
Jejunum	Corn stover	Negative	behavior	1.10E-03	190
Jejunum	Corn stover	Negative	dyskinesia	1.10E-03	124
Jejunum	Corn stover	Negative	S phase	1.10E-03	58
Jejunum	Corn stover	Negative	metabolism of fructose-1,6-diphosphate	1.11E-03	6
Jejunum	Corn stover	Negative	epileptic seizure	1.11E-03	40
Jejunum	Corn stover	Negative	quantity of metal ion	1.16E-03	92
Jejunum	Corn stover	Negative	abnormal morphology of nervous system	1.28E-03	143
Jejunum	Corn stover	Negative	growth of vessel	1.30E-03	29
Jejunum	Corn	Negative	infection of kidney cell lines	1.36E-03	62

	stover				
Jejunum	Corn stover	Negative	proliferation of tumor cell lines	1.36E-03	284
Jejunum	Corn stover	Negative	proliferation of fibroblast cell lines	1.42E-03	88
Jejunum	Corn stover	Negative	outgrowth of neurons	1.48E-03	85
Jejunum	Corn stover	Negative	accumulation of blood cells	1.49E-03	61
Jejunum	Corn stover	Negative	activation of antigen presenting cells	1.55E-03	68
Jejunum	Corn stover	Negative	abnormal morphology of body cavity	1.55E-03	215
Jejunum	Corn stover	Negative	quantity of cellular protrusions	1.56E-03	38
Jejunum	Corn stover	Negative	degeneration of cells	1.58E-03	73
Jejunum	Corn stover	Negative	synthesis of carbohydrate	1.58E-03	88
Jejunum	Corn stover	Negative	skin abnormality	1.64E-03	51
Jejunum	Corn stover	Negative	outgrowth of neurites	1.70E-03	84
Jejunum	Corn stover	Negative	quantity of T lymphocytes	1.71E-03	108
Jejunum	Corn stover	Negative	influx of Ca2+	1.71E-03	43
Jejunum	Corn stover	Negative	influx of inorganic cation	1.76E-03	44
Jejunum	Corn stover	Negative	proliferation of hematopoietic cell lines	1.76E-03	50
Jejunum	Corn stover	Negative	degeneration of neurons	1.76E-03	53
Jejunum	Corn stover	Negative	differentiation of epithelial cells	1.76E-03	67
Jejunum	Corn stover	Negative	abnormal morphology of heart	1.76E-03	80
Jejunum	Corn stover	Negative	abnormal morphology of head	1.77E-03	161
Jejunum	Corn stover	Negative	development of lymphatic system	1.77E-03	75
Jejunum	Corn stover	Negative	development of neurons	1.84E-03	152
Jejunum	Corn	Negative	abnormal morphology of thoracic vertebra	1.84E-03	12

	stover				
Jejunum	Corn stover	Negative	abnormal morphology of Schwann cells	1.87E-03	9
Jejunum	Corn stover	Negative	growth of lymphatic system component	1.87E-03	44
Jejunum	Corn stover	Negative	morphology of right ventricle	1.92E-03	17
Jejunum	Corn stover	Negative	morphology of fibroblast cell lines	2.06E-03	23
Jejunum	Corn stover	Negative	necrosis of epithelial tissue	2.20E-03	117
Jejunum	Corn stover	Negative	quantity of Ca2+	2.24E-03	84
Jejunum	Corn stover	Negative	quantity of phagocytes	2.26E-03	87
Jejunum	Corn stover	Negative	abnormal morphology of cells	2.52E-03	234
Jejunum	Corn stover	Negative	size of body	2.63E-03	163
Jejunum	Corn stover	Negative	abnormal morphology of respiratory system	2.63E-03	65
Jejunum	Corn stover	Negative	concentration of triacylglycerol	2.63E-03	65
Jejunum	Corn stover	Negative	migration of fibroblasts	2.63E-03	34
Jejunum	Corn stover	Negative	growth of embryonic tissue	2.72E-03	59
Jejunum	Corn stover	Negative	metabolism of monosaccharide	2.76E-03	30
Jejunum	Corn stover	Negative	cellular degradation	2.86E-03	55
Jejunum	Corn stover	Negative	generation of superoxide	2.86E-03	18
Jejunum	Corn stover	Negative	function of leukocytes	2.86E-03	107
Jejunum	Corn stover	Negative	homeostasis of ion	2.86E-03	52
Jejunum	Corn stover	Negative	cell movement of connective tissue cells	2.86E-03	49
Jejunum	Corn stover	Negative	infection of embryonic cell lines	2.86E-03	59
Jejunum	Corn stover	Negative	infection of epithelial cell lines	2.86E-03	59
Jejunum	Corn	Negative	inflammatory response	2.87E-03	150

	stover				
Jejunum	Corn stover	Negative	abnormal morphology of renal tubule	2.88E-03	27
Jejunum	Corn stover	Negative	proliferative vitreoretinopathy	3.00E-03	6
Jejunum	Corn stover	Negative	development of lymphatic system component	3.16E-03	65
Jejunum	Corn stover	Negative	dysgenesis	3.32E-03	107
Jejunum	Corn stover	Negative	oligomerization of protein	3.34E-03	52
Jejunum	Corn stover	Negative	extension of cellular protrusions	3.34E-03	46
Jejunum	Corn stover	Negative	neurological signs	3.34E-03	127
Jejunum	Corn stover	Negative	formation of cells	3.35E-03	185
Jejunum	Corn stover	Negative	apoptosis of blood cells	3.35E-03	99
Jejunum	Corn stover	Negative	formation of filopodia	3.44E-03	38
Jejunum	Corn stover	Negative	formation of vascular lesion	3.71E-03	28
Jejunum	Corn stover	Negative	Huntington's Disease	3.74E-03	113
Jejunum	Corn stover	Negative	quantity of granulocytes	3.74E-03	66
Jejunum	Corn stover	Negative	abnormal morphology of bone	3.74E-03	88
Jejunum	Corn stover	Negative	neuronal cell death	3.74E-03	132
Jejunum	Corn stover	Negative	quantity of connective tissue	3.74E-03	118
Jejunum	Corn stover	Negative	development of connective tissue	3.80E-03	76
Jejunum	Corn stover	Negative	cell death of blood cells	3.86E-03	134
Jejunum	Corn stover	Negative	abnormal morphology of right ventricle	3.93E-03	15
Jejunum	Corn stover	Negative	formation of lung	3.98E-03	63
Jejunum	Corn stover	Negative	concentration of fatty acid	4.04E-03	59
Jejunum	Corn	Negative	apoptosis of myeloid cells	4.10E-03	42

	stover				
Jejunum	Corn stover	Negative	apoptosis of phagocytes	4.10E-03	42
Jejunum	Corn stover	Negative	quantity of myeloid cells	4.14E-03	73
Jejunum	Corn stover	Negative	uptake of monosaccharide	4.15E-03	57
Jejunum	Corn stover	Negative	MAPKKK cascade	4.19E-03	45
Jejunum	Corn stover	Negative	fusion of cellular membrane	4.39E-03	17
Jejunum	Corn stover	Negative	interphase	4.40E-03	127
Jejunum	Corn stover	Negative	formation of testis	4.46E-03	29
Jejunum	Corn stover	Negative	growth of muscle tissue	4.46E-03	76
Jejunum	Corn stover	Negative	outgrowth of cells	4.46E-03	87
Jejunum	Corn stover	Negative	differentiation of muscle	4.46E-03	63
Jejunum	Corn stover	Negative	chemotaxis of myeloid cells	4.71E-03	61
Jejunum	Corn stover	Negative	congenital anomaly of skin	4.72E-03	40
Jejunum	Corn stover	Negative	accumulation of cells	4.77E-03	72
Jejunum	Corn stover	Negative	quantity of thymus gland	4.86E-03	50
Jejunum	Corn stover	Negative	differentiation of embryonic cells	4.88E-03	49
Jejunum	Corn stover	Negative	neurodegeneration of axons	4.95E-03	22
Jejunum	Corn stover	Negative	neurodegeneration of neurites	4.99E-03	23
Jejunum	Corn stover	Negative	formation of vessel component	5.21E-03	19
Jejunum	Corn stover	Negative	cell viability of tumor cell lines	5.24E-03	148
Jejunum	Corn stover	Negative	cell movement of leukemia cell lines	5.42E-03	32
Jejunum	Corn stover	Negative	development of body axis	5.46E-03	195
Jejunum	Corn	Negative	proliferation of vascular smooth muscle cells	5.51E-03	35

	stover				
Jejunum	Corn stover	Negative	iris coloboma	5.51E-03	5
Jejunum	Corn stover	Negative	metabolism of glucose-6-phosphate	5.51E-03	5
Jejunum	Corn stover	Negative	abnormal morphology of embryonic tissue	5.55E-03	107
Jejunum	Corn stover	Negative	hypoplasia of kidney	5.63E-03	16
Jejunum	Corn stover	Negative	homo-oligomerization of protein	5.64E-03	31
Jejunum	Corn stover	Negative	proliferation of muscle cells	5.74E-03	75
Jejunum	Corn stover	Negative	proliferation of leukocyte cell lines	5.74E-03	44
Jejunum	Corn stover	Negative	adhesion of immune cells	5.80E-03	71
Jejunum	Corn stover	Negative	accumulation of leukocytes	5.86E-03	56
Jejunum	Corn stover	Negative	abnormal morphology of membrane tissue	5.86E-03	38
Jejunum	Corn stover	Negative	organization of fibrils	5.86E-03	15
Jejunum	Corn stover	Negative	apoptosis of antigen presenting cells	5.86E-03	32
Jejunum	Corn stover	Negative	cell death of muscle	5.90E-03	70
Jejunum	Corn stover	Negative	glycolysis of cells	5.90E-03	27
Jejunum	Corn stover	Negative	Hypoplasia	6.00E-03	99
Jejunum	Corn stover	Negative	perinatal death	6.00E-03	115
Jejunum	Corn stover	Negative	morphology of heart ventricle	6.08E-03	42
Jejunum	Corn stover	Negative	replication of virus	6.24E-03	109
Jejunum	Corn stover	Negative	growth of plasma membrane projections	6.24E-03	92
Jejunum	Corn stover	Negative	proliferation of adipoblasts	6.24E-03	6
Jejunum	Corn stover	Negative	multiple congenital anomalies	6.24E-03	84
Jejunum	Corn	Negative	proliferation of central nervous system cells	6.26E-03	34

	stover				
Jejunum	Corn stover	Negative	morphology of bone	6.29E-03	89
Jejunum	Corn stover	Negative	cell death of cervical cancer cell lines	6.32E-03	71
Jejunum	Corn stover	Negative	quantity of thymocytes	6.34E-03	49
Jejunum	Corn stover	Negative	cell movement of leukocytes	6.67E-03	145
Jejunum	Corn stover	Negative	cell movement of myeloid cells	6.68E-03	105
Jejunum	Corn stover	Negative	secretion of molecule	6.70E-03	107
Jejunum	Corn stover	Negative	growth of neurites	6.73E-03	91
Jejunum	Corn stover	Negative	organization of actin filaments	7.04E-03	25
Jejunum	Corn stover	Negative	homeostasis of inorganic cation	7.11E-03	41
Jejunum	Corn stover	Negative	morphology of central nervous system	7.19E-03	100
Jejunum	Corn stover	Negative	cell death of central nervous system cells	7.31E-03	60
Jejunum	Corn stover	Negative	size of cells	7.36E-03	85
Jejunum	Corn stover	Negative	quantity of cytokine	7.47E-03	53
Jejunum	Corn stover	Negative	vascularization of body region	7.53E-03	31
Jejunum	Corn stover	Negative	benign neoplasia	7.53E-03	172
Jejunum	Corn stover	Negative	branching of cells	7.58E-03	74
Jejunum	Corn stover	Negative	morphogenesis of neurons	7.59E-03	84
Jejunum	Corn stover	Negative	apoptosis of cervical cancer cell lines	7.60E-03	58
Jejunum	Corn stover	Negative	anxiety	7.63E-03	41
Jejunum	Corn stover	Negative	function of muscle	7.69E-03	70
Jejunum	Corn stover	Negative	activation of macrophages	7.74E-03	50
Jejunum	Corn	Negative	formation of thymus gland	7.88E-03	36

	stover				
Jejunum	Corn stover	Negative	quantity of kidney	7.88E-03	16
Jejunum	Corn stover	Negative	necrosis of muscle	7.91E-03	69
Jejunum	Corn stover	Negative	proliferation of epithelial cells	7.92E-03	96
Jejunum	Corn stover	Negative	proliferation of mitochondria	8.04E-03	4
Jejunum	Corn stover	Negative	cell movement of phagocytes	8.18E-03	106
Jejunum	Corn stover	Negative	homeostasis of metal ion	8.18E-03	40
Jejunum	Corn stover	Negative	activation of central nervous system cells	8.18E-03	20
Jejunum	Corn stover	Negative	fatty acid metabolism	8.51E-03	115
Jejunum	Corn stover	Negative	cell cycle progression	8.80E-03	192
Jejunum	Corn stover	Negative	contractility of muscle	9.01E-03	41
Jejunum	Corn stover	Negative	oxidation of D-glucose	9.05E-03	14
Jejunum	Corn stover	Negative	atherosclerotic lesion	9.05E-03	34
Jejunum	Corn stover	Negative	formation of neointima	9.05E-03	18
Jejunum	Corn stover	Negative	chemotaxis of phagocytes	9.24E-03	60
Jejunum	Corn stover	Negative	growth of kidney	9.57E-03	22
Jejunum	Corn stover	Negative	atherogenesis	9.58E-03	20
Jejunum	Corn stover	Negative	cell death of immune cells	9.66E-03	125
Jejunum	Corn stover	Negative	flux of Ca2+	9.77E-03	58
Jejunum	Corn stover	Negative	size of embryo	9.81E-03	71
Jejunum	Corn stover	Negative	chemotaxis of antigen presenting cells	9.97E-03	34
Jejunum	Corn stover	Negative	apoptosis of macrophages	9.97E-03	25
Jejunum	Corn	Negative	apoptosis of leukocytes	1.01E-02	89

	stover				
Jejunum	Corn stover	Negative	uptake of carbohydrate	1.01E-02	59
Jejunum	Corn stover	Negative	hypersensitive reaction	1.01E-02	84
Jejunum	Corn stover	Negative	flux of inorganic cation	1.01E-02	61
Jejunum	Corn stover	Negative	morphology of neuroglia	1.01E-02	27
Jejunum	Corn stover	Negative	concentration of Ca2+	1.01E-02	29
Jejunum	Corn stover	Negative	proliferation of smooth muscle cells	1.02E-02	58
Jejunum	Corn stover	Negative	infection by HIV-1	1.02E-02	116
Jejunum	Corn stover	Negative	hypoplasia of organ	1.02E-02	87
Jejunum	Corn stover	Negative	morphogenesis of neurites	1.02E-02	82
Jejunum	Corn stover	Negative	invasion of carcinoma cell lines	1.03E-02	42
Jejunum	Corn stover	Negative	cell death of muscle cells	1.04E-02	67
Jejunum	Corn stover	Negative	trafficking of cells	1.07E-02	19
Jejunum	Corn stover	Negative	abnormal morphology of neuroglia	1.08E-02	21
Jejunum	Corn stover	Negative	oxidation of palmitic acid	1.09E-02	14
Jejunum	Corn stover	Negative	resorption of bone	1.10E-02	36
Jejunum	Corn stover	Negative	sprouting	1.10E-02	76
Jejunum	Corn stover	Negative	juvenile dermatomyositis	1.10E-02	16
Jejunum	Corn stover	Negative	uterine serous papillary cancer	1.11E-02	48
Jejunum	Corn stover	Negative	respiratory system development	1.12E-02	68
Jejunum	Corn stover	Negative	formation of lymphoid organ	1.12E-02	54
Jejunum	Corn stover	Negative	cell death of stromal cells	1.13E-02	6
Jejunum	Corn	Negative	synthesis of porphyrin	1.14E-02	11

	stover				
Jejunum	Corn stover	Negative	proliferation of blood cells	1.15E-02	156
Jejunum	Corn stover	Negative	Organ Degeneration	1.16E-02	95
Jejunum	Corn stover	Negative	polymerization of protein	1.16E-02	73
Jejunum	Corn stover	Negative	apoptosis of muscle cell lines	1.16E-02	17
Jejunum	Corn stover	Negative	abnormal morphology of axial skeleton	1.20E-02	41
Jejunum	Corn stover	Negative	growth of blood vessel	1.20E-02	22
Jejunum	Corn stover	Negative	activation of cells	1.20E-02	179
Jejunum	Corn stover	Negative	growth of renal glomerulus	1.20E-02	18
Jejunum	Corn stover	Negative	infection by RNA virus	1.21E-02	160
Jejunum	Corn stover	Negative	long-term potentiation	1.21E-02	50
Jejunum	Corn stover	Negative	damage of liver cells	1.21E-02	9
Jejunum	Corn stover	Negative	collapse of growth cone	1.21E-02	19
Jejunum	Corn stover	Negative	migration of leukemia cell lines	1.22E-02	20
Jejunum	Corn stover	Negative	abnormal morphology of skeleton	1.22E-02	55
Jejunum	Corn stover	Negative	adhesion of blood cells	1.22E-02	74
Jejunum	Corn stover	Negative	proliferation of hematopoietic cells	1.23E-02	52
Jejunum	Corn stover	Negative	development of abdomen	1.23E-02	110
Jejunum	Corn stover	Negative	allergy	1.23E-02	79
Jejunum	Corn stover	Negative	exit from S phase of colon cancer cell lines	1.23E-02	5
Jejunum	Corn stover	Negative	proliferation of immune cells	1.23E-02	147
Jejunum	Corn stover	Negative	replication of RNA virus	1.23E-02	97
Jejunum	Corn	Negative	autosomal dominant disease	1.27E-02	120

	stover				
Jejunum	Corn stover	Negative	homeostasis of transition metal ion	1.27E-02	16
Jejunum	Corn stover	Negative	size of animal	1.27E-02	38
Jejunum	Corn stover	Negative	abnormal morphology of nephron	1.27E-02	31
Jejunum	Corn stover	Negative	development of connective tissue cells	1.27E-02	46
Jejunum	Corn stover	Negative	formation of skin	1.29E-02	72
Jejunum	Corn stover	Negative	flux of ion	1.31E-02	64
Jejunum	Corn stover	Negative	differentiation of leukocytes	1.31E-02	126
Jejunum	Corn stover	Negative	cell death of melanoma cell lines	1.32E-02	51
Jejunum	Corn stover	Negative	homeostasis of Cu2+	1.32E-02	7
Jejunum	Corn stover	Negative	number of caudal vertebra	1.32E-02	7
Jejunum	Corn stover	Negative	Dermatitis	1.32E-02	84
Jejunum	Corn stover	Negative	mitosis	1.32E-02	95
Jejunum	Corn stover	Negative	mass of connective tissue	1.32E-02	42
Jejunum	Corn stover	Negative	morphology of axial skeleton	1.32E-02	42
Jejunum	Corn stover	Negative	function of neutrophils	1.32E-02	21
Jejunum	Corn stover	Negative	morphology of cardiovascular tissue	1.32E-02	21
Jejunum	Corn stover	Negative	migration of stem cells	1.32E-02	13
Jejunum	Corn stover	Negative	synthesis of heme	1.32E-02	10
Jejunum	Corn stover	Negative	abnormal morphology of vertebrae	1.32E-02	30
Jejunum	Corn stover	Negative	concentration of phosphatidic acid	1.32E-02	30
Jejunum	Corn stover	Negative	function of phagocytes	1.32E-02	61
Jejunum	Corn	Negative	remodeling of bone	1.32E-02	40

	stover				
Jejunum	Corn stover	Negative	infection by Retroviridae	1.33E-02	134
Jejunum	Corn stover	Negative	proliferation of kidney cells	1.33E-02	19
Jejunum	Corn stover	Negative	glucose metabolism disorder	1.33E-02	230
Jejunum	Corn stover	Negative	cell death of epithelial cells	1.34E-02	95
Jejunum	Corn stover	Negative	synthesis of fatty acid	1.34E-02	62
Jejunum	Corn stover	Negative	abnormal morphology of epithelial tissue	1.39E-02	81
Jejunum	Corn stover	Negative	growth of yeast	1.40E-02	23
Jejunum	Corn stover	Negative	shape change of neurites	1.40E-02	57
Jejunum	Corn stover	Negative	quantity of hematopoietic progenitor cells	1.42E-02	85
Jejunum	Corn stover	Negative	synthesis of nucleotide	1.44E-02	71
Jejunum	Corn stover	Negative	contractility of myocardium	1.44E-02	16
Jejunum	Corn stover	Negative	cell movement of sarcoma cell lines	1.44E-02	22
Jejunum	Corn stover	Negative	activation of Protein kinase	1.45E-02	51
Jejunum	Corn stover	Negative	morphology of muscle	1.47E-02	66
Jejunum	Corn stover	Negative	fertility	1.52E-02	71
Jejunum	Corn stover	Negative	morphology of endothelial tissue	1.52E-02	20
Jejunum	Corn stover	Negative	smooth muscle tumor	1.52E-02	60
Jejunum	Corn stover	Negative	stage 2-3 breast cancer	1.52E-02	18
Jejunum	Corn stover	Negative	cell death of brain	1.53E-02	58
Jejunum	Corn stover	Negative	reorganization of cytoskeleton	1.53E-02	40
Jejunum	Corn stover	Negative	accumulation of myeloid cells	1.55E-02	37
Jejunum	Corn	Negative	vascularization	1.55E-02	48

	stover				
Jejunum	Corn stover	Negative	migration of mesenchymal stem cells	1.56E-02	9
Jejunum	Corn stover	Negative	leiomyomatosis	1.57E-02	57
Jejunum	Corn stover	Negative	apoptosis of bone marrow cells	1.60E-02	15
Jejunum	Corn stover	Negative	congenital disorders of glycosylation	1.60E-02	15
Jejunum	Corn stover	Negative	proliferation of glomerular cells	1.60E-02	15
Jejunum	Corn stover	Negative	formation of lymphatic system component	1.61E-02	58
Jejunum	Corn stover	Negative	long-term potentiation of synapse	1.64E-02	32
Jejunum	Corn stover	Negative	exocytosis by cells	1.64E-02	28
Jejunum	Corn stover	Negative	HIV infection	1.65E-02	132
Jejunum	Corn stover	Negative	development of head	1.66E-02	178
Jejunum	Corn stover	Negative	synthesis of polysaccharide	1.66E-02	38
Jejunum	Corn stover	Negative	migration of muscle cells	1.67E-02	35
Jejunum	Corn stover	Negative	autosomal dominant Emery-Dreifuss muscular dystrophy	1.67E-02	16
Jejunum	Corn stover	Negative	release of ATP	1.67E-02	12
Jejunum	Corn stover	Negative	activation of neuroglia	1.67E-02	29
Jejunum	Corn stover	Negative	function of heart	1.67E-02	47
Jejunum	Corn stover	Negative	genodermatosis	1.73E-02	19
Jejunum	Corn stover	Negative	benign connective or soft tissue neoplasm	1.74E-02	71
Jejunum	Corn stover	Negative	quantity of antigen presenting cells	1.75E-02	55
Jejunum	Corn stover	Negative	synthesis of nitric oxide	1.75E-02	55
Jejunum	Corn stover	Negative	coagulation	1.75E-02	40
Jejunum	Corn	Negative	proliferation of lymphatic endothelial cells	1.76E-02	8

	stover				
Jejunum	Corn stover	Negative	uptake of thymidine	1.76E-02	8
Jejunum	Corn stover	Negative	proliferation of prostate cancer cell lines	1.76E-02	60
Jejunum	Corn stover	Negative	abnormal morphology of zona fasciculata	1.76E-02	6
Jejunum	Corn stover	Negative	exit from S phase	1.76E-02	6
Jejunum	Corn stover	Negative	growth of Candida albicans ATCC 90028	1.76E-02	6
Jejunum	Corn stover	Negative	import of lipid	1.76E-02	6
Jejunum	Corn stover	Negative	shape change of neuroblastoma cell lines	1.76E-02	6
Jejunum	Corn stover	Negative	glomerulosclerosis	1.76E-02	28
Jejunum	Corn stover	Negative	synthesis of terpenoid	1.77E-02	56
Jejunum	Corn stover	Negative	growth of brain	1.81E-02	26
Jejunum	Corn stover	Negative	quantity of muscle cells	1.81E-02	26
Jejunum	Corn stover	Negative	glycolysis	1.81E-02	29
Jejunum	Corn stover	Negative	activation of phagocytes	1.81E-02	72
Jejunum	Corn stover	Negative	binding of protein binding site	1.81E-02	57
Jejunum	Corn stover	Negative	cell movement of macrophages	1.81E-02	53
Jejunum	Corn stover	Negative	branching of neurites	1.81E-02	55
Jejunum	Corn stover	Negative	apoptosis of kidney cell lines	1.82E-02	48
Jejunum	Corn stover	Negative	production of HIV	1.82E-02	15
Jejunum	Corn stover	Negative	morphology of vertebral column	1.85E-02	31
Jejunum	Corn stover	Negative	activation of hematopoietic cells	1.85E-02	13
Jejunum	Corn stover	Negative	metabolism of porphyrin	1.85E-02	13
Jejunum	Corn	Negative	proliferation of mesangial cells	1.85E-02	13

	stover				
Jejunum	Corn stover	Negative	cell movement of fibroblasts	1.87E-02	38
Jejunum	Corn stover	Negative	morphology of lymph node	1.90E-02	34
Jejunum	Corn stover	Negative	homeostasis of leukocytes	1.91E-02	104
Jejunum	Corn stover	Negative	quantity of marginal-zone B lymphocytes	1.91E-02	19
Jejunum	Corn stover	Negative	transmigration of blood cells	1.91E-02	28
Jejunum	Corn stover	Negative	export of molecule	1.95E-02	58
Jejunum	Corn stover	Negative	cell death of brain cells	1.97E-02	54
Jejunum	Corn stover	Negative	activation of hematopoietic progenitor cells	1.98E-02	12
Jejunum	Corn stover	Negative	synthesis of hyaluronic acid	1.98E-02	12
Jejunum	Corn stover	Negative	abnormal thermoregulation	1.98E-02	9
Jejunum	Corn stover	Negative	congenital disorder of glycosylation type 1	1.98E-02	9
Jejunum	Corn stover	Negative	metabolism of nucleoside triphosphate	1.98E-02	30
Jejunum	Corn stover	Negative	quantity of interleukin	1.98E-02	30
Jejunum	Corn stover	Negative	mass of adipose tissue	1.98E-02	39
Jejunum	Corn stover	Negative	chronic myeloid leukemia	1.98E-02	24
Jejunum	Corn stover	Negative	contractility of cardiac muscle	2.00E-02	31
Jejunum	Corn stover	Negative	differentiation of muscle cell lines	2.04E-02	34
Jejunum	Corn stover	Negative	Lymphocyte homeostasis	2.06E-02	102
Jejunum	Corn stover	Negative	migration of mesenchymal cells	2.06E-02	11
Jejunum	Corn stover	Negative	activation of vascular endothelial cells	2.08E-02	10
Jejunum	Corn stover	Negative	apoptosis of melanoma cell lines	2.08E-02	28
Jejunum	Corn	Negative	production of virus	2.09E-02	23

	stover				
Jejunum	Corn stover	Negative	release of nitric oxide	2.10E-02	20
Jejunum	Corn stover	Negative	morphology of brain	2.10E-02	88
Jejunum	Corn stover	Negative	stage 3 cancer	2.12E-02	29
Jejunum	Corn stover	Negative	cell death of cerebral cortex cells	2.14E-02	46
Jejunum	Corn stover	Negative	spatial learning	2.15E-02	38
Jejunum	Corn stover	Negative	bone mineral density	2.19E-02	36
Jejunum	Corn stover	Negative	abnormal morphology of lymph node	2.20E-02	32
Jejunum	Corn stover	Negative	apoptosis of stromal cells	2.21E-02	5
Jejunum	Corn stover	Negative	metabolism of fructose-6-phosphate	2.21E-02	5
Jejunum	Corn stover	Negative	re-entry into cell cycle progression of fibroblast cell lines	2.21E-02	5
Jejunum	Corn stover	Negative	abnormal morphology of marginal-zone B lymphocytes	2.24E-02	4
Jejunum	Corn stover	Negative	cell death of bone marrow stromal cells	2.24E-02	4
Jejunum	Corn stover	Negative	hereditary nonspherocytic hemolytic anemia	2.24E-02	4
Jejunum	Corn stover	Negative	quantity of renal tubule	2.24E-02	4
Jejunum	Corn stover	Negative	metabolism of nucleotide	2.27E-02	83
Jejunum	Corn stover	Negative	branching of neurons	2.28E-02	56
Jejunum	Corn stover	Negative	cell death of smooth muscle cells	2.28E-02	25
Jejunum	Corn stover	Negative	differentiation of bone	2.32E-02	78
Jejunum	Corn stover	Negative	Ph positive chronic myeloid leukemia in chronic phase	2.32E-02	8
Jejunum	Corn stover	Negative	advanced sarcoma	2.32E-02	8
Jejunum	Corn stover	Negative	quantity of sustentacular cells	2.32E-02	8
Jejunum	Corn	Negative	release of eicosanoid	2.32E-02	30

	stover				
Jejunum	Corn stover	Negative	quantity of protein in blood	2.32E-02	95
Jejunum	Corn stover	Negative	accumulation of antigen presenting cells	2.32E-02	20
Jejunum	Corn stover	Negative	autosomal dominant myopathy	2.32E-02	20
Jejunum	Corn stover	Negative	morphology of left ventricle	2.32E-02	20
Jejunum	Corn stover	Negative	production of HIV-1	2.32E-02	14
Jejunum	Corn stover	Negative	stress response of tumor cell lines	2.32E-02	14
Jejunum	Corn stover	Negative	cell death of leukocyte cell lines	2.32E-02	41
Jejunum	Corn stover	Negative	release of fatty acid	2.32E-02	35
Jejunum	Corn stover	Negative	formation of vesicles	2.32E-02	26
Jejunum	Corn stover	Negative	chemotaxis of leukocytes	2.33E-02	68
Jejunum	Corn stover	Negative	activation of enzyme	2.33E-02	74
Jejunum	Corn stover	Negative	disorder of lipid metabolism	2.35E-02	40
Jejunum	Corn stover	Negative	cell death of hematopoietic cell lines	2.37E-02	46
Jejunum	Corn stover	Negative	aggregation of blood platelets	2.39E-02	39
Jejunum	Corn stover	Negative	T cell development	2.40E-02	94
Jejunum	Corn stover	Negative	development of leukocytes	2.41E-02	109
Liver	Alfalfa hay	Positive	cancer	1.78E-32	3020
Liver	Alfalfa hay	Positive	malignant solid tumor	1.78E-32	2988
Liver	Alfalfa hay	Positive	morphology of cells	1.33E-26	681
Liver	Alfalfa hay	Positive	cell death	2.05E-25	1066
Liver	Alfalfa hay	Positive	organismal death	4.20E-24	798
Liver	Alfalfa	Positive	proliferation of cells	4.54E-24	1155

	hay				
Liver	Alfalfa hay	Positive	morbidity or mortality	5.60E-24	806
Liver	Alfalfa hay	Positive	necrosis	2.26E-21	834
Liver	Alfalfa hay	Positive	apoptosis	7.02E-20	846
Liver	Alfalfa hay	Positive	neoplasia of epithelial tissue	4.45E-16	2451
Liver	Alfalfa hay	Positive	angiogenesis	1.65E-15	328
Liver	Alfalfa hay	Positive	cell death of tumor cell lines	1.68E-15	510
Liver	Alfalfa hay	Positive	cell movement	1.68E-15	680
Liver	Alfalfa hay	Positive	abdominal neoplasm	2.69E-15	2428
Liver	Alfalfa hay	Positive	expression of RNA	2.72E-15	684
Liver	Alfalfa hay	Positive	abnormal morphology of cells	2.96E-15	433
Liver	Alfalfa hay	Positive	tumorigenesis of tissue	3.33E-15	2481
Liver	Alfalfa hay	Positive	quantity of cells	3.33E-15	577
Liver	Alfalfa hay	Positive	epithelial cancer	3.59E-15	2422
Liver	Alfalfa hay	Positive	transport of molecule	3.92E-15	532
Liver	Alfalfa hay	Positive	differentiation of cells	6.61E-15	710
Liver	Alfalfa hay	Positive	migration of cells	6.61E-15	613
Liver	Alfalfa hay	Positive	transcription of RNA	1.12E-14	599
Liver	Alfalfa hay	Positive	organization of cytoskeleton	1.26E-14	467
Liver	Alfalfa hay	Positive	transcription	1.26E-14	637
Liver	Alfalfa hay	Positive	organization of cytoplasm	1.45E-14	506
Liver	Alfalfa hay	Positive	cellular homeostasis	3.68E-14	501
Liver	Alfalfa	Positive	abdominal cancer	4.07E-14	2392

	hay				
Liver	Alfalfa hay	Positive	vasculogenesis	4.23E-14	269
Liver	Alfalfa hay	Positive	digestive organ tumor	5.20E-14	2109
Liver	Alfalfa hay	Positive	apoptosis of tumor cell lines	2.01E-13	408
Liver	Alfalfa hay	Positive	digestive system cancer	3.04E-13	2085
Liver	Alfalfa hay	Positive	morphology of cardiovascular system	7.12E-13	228
Liver	Alfalfa hay	Positive	metabolism of carbohydrate	6.95E-12	225
Liver	Alfalfa hay	Positive	quantity of leukocytes	9.88E-12	314
Liver	Alfalfa hay	Positive	microtubule dynamics	1.01E-11	392
Liver	Alfalfa hay	Positive	transcription of DNA	1.04E-11	492
Liver	Alfalfa hay	Positive	development of vasculature	1.10E-11	160
Liver	Alfalfa hay	Positive	cell survival	1.32E-11	451
Liver	Alfalfa hay	Positive	quantity of blood cells	2.27E-11	345
Liver	Alfalfa hay	Positive	cell spreading	3.66E-11	112
Liver	Alfalfa hay	Positive	cell movement of tumor cell lines	3.90E-11	292
Liver	Alfalfa hay	Positive	Viral Infection	4.17E-11	510
Liver	Alfalfa hay	Positive	cell death of connective tissue cells	4.71E-11	216
Liver	Alfalfa hay	Positive	synthesis of lipid	5.36E-11	250
Liver	Alfalfa hay	Positive	size of body	1.64E-10	289
Liver	Alfalfa hay	Positive	autosomal recessive disease	2.56E-10	309
Liver	Alfalfa hay	Positive	concentration of lipid	2.92E-10	276
Liver	Alfalfa hay	Positive	abnormal morphology of cardiovascular system	3.13E-10	202
Liver	Alfalfa	Positive	development of body trunk	3.17E-10	365

	hay				
Liver	Alfalfa hay	Positive	uptake of carbohydrate	3.27E-10	114
Liver	Alfalfa hay	Positive	cell viability	3.27E-10	417
Liver	Alfalfa hay	Positive	invasion of cells	3.35E-10	293
Liver	Alfalfa hay	Positive	quantity of connective tissue	4.84E-10	212
Liver	Alfalfa hay	Positive	proliferation of tumor cell lines	1.35E-09	483
Liver	Alfalfa hay	Positive	uptake of monosaccharide	1.76E-09	105
Liver	Alfalfa hay	Positive	function of cardiovascular system	2.14E-09	137
Liver	Alfalfa hay	Positive	invasion of tumor cell lines	2.71E-09	228
Liver	Alfalfa hay	Positive	abnormal morphology of thoracic cavity	2.88E-09	204
Liver	Alfalfa hay	Positive	morphology of body cavity	4.40E-09	381
Liver	Alfalfa hay	Positive	quantity of mononuclear leukocytes	4.51E-09	248
Liver	Alfalfa hay	Positive	activation of DNA endogenous promoter	5.42E-09	385
Liver	Alfalfa hay	Positive	necrosis of epithelial tissue	7.21E-09	203
Liver	Alfalfa hay	Positive	development of epithelial tissue	7.21E-09	181
Liver	Alfalfa hay	Positive	formation of cellular protrusions	7.93E-09	296
Liver	Alfalfa hay	Positive	formation of cytoskeleton	8.48E-09	137
Liver	Alfalfa hay	Positive	development of body axis	8.48E-09	339
Liver	Alfalfa hay	Positive	formation of cells	8.74E-09	319
Liver	Alfalfa hay	Positive	congenital anomaly of musculoskeletal system	1.30E-08	246
Liver	Alfalfa hay	Positive	development of cytoplasm	1.40E-08	163
Liver	Alfalfa hay	Positive	function of leukocytes	1.44E-08	186
Liver	Alfalfa	Positive	cell death of fibroblast cell lines	1.53E-08	152

	hay				
Liver	Alfalfa hay	Positive	quantity of lymphocytes	1.58E-08	237
Liver	Alfalfa hay	Positive	Movement Disorders	1.73E-08	339
Liver	Alfalfa hay	Positive	formation of muscle	2.28E-08	142
Liver	Alfalfa hay	Positive	fatty acid metabolism	2.76E-08	204
Liver	Alfalfa hay	Positive	abnormal morphology of body cavity	3.10E-08	361
Liver	Alfalfa hay	Positive	shape change of tumor cell lines	3.10E-08	64
Liver	Alfalfa hay	Positive	seizure disorder	3.10E-08	164
Liver	Alfalfa hay	Positive	growth of embryo	3.44E-08	166
Liver	Alfalfa hay	Positive	benign neoplasia	3.62E-08	299
Liver	Alfalfa hay	Positive	migration of tumor cell lines	3.62E-08	235
Liver	Alfalfa hay	Positive	morphology of vessel	3.62E-08	113
Liver	Alfalfa hay	Positive	growth of organism	3.62E-08	273
Liver	Alfalfa hay	Positive	proliferation of connective tissue cells	3.62E-08	201
Liver	Alfalfa hay	Positive	function of blood cells	3.78E-08	199
Liver	Alfalfa hay	Positive	cellular infiltration by leukocytes	4.21E-08	138
Liver	Alfalfa hay	Positive	growth of connective tissue	4.31E-08	216
Liver	Alfalfa hay	Positive	morphology of blood vessel	4.31E-08	105
Liver	Alfalfa hay	Positive	cell movement of leukocytes	4.89E-08	252
Liver	Alfalfa hay	Positive	development of head	5.36E-08	314
Liver	Alfalfa hay	Positive	cell transformation	5.61E-08	160
Liver	Alfalfa hay	Positive	synthesis of carbohydrate	5.70E-08	149
Liver	Alfalfa	Positive	cell death of epithelial cells	5.70E-08	171

	hay				
Liver	Alfalfa hay	Positive	formation of actin stress fibers	5.92E-08	91
Liver	Alfalfa hay	Positive	homing	6.67E-08	194
Liver	Alfalfa hay	Positive	cell movement of neutrophils	6.74E-08	109
Liver	Alfalfa hay	Positive	homing of cells	7.00E-08	189
Liver	Alfalfa hay	Positive	function of heart	7.70E-08	88
Liver	Alfalfa hay	Positive	formation of actin filaments	8.34E-08	111
Liver	Alfalfa hay	Positive	proliferation of fibroblasts	8.59E-08	126
Liver	Alfalfa hay	Positive	fibrogenesis	8.89E-08	144
Liver	Alfalfa hay	Positive	leukocyte migration	9.05E-08	281
Liver	Alfalfa hay	Positive	sprouting	1.03E-07	136
Liver	Alfalfa hay	Positive	breast or colorectal cancer	1.06E-07	1392
Liver	Alfalfa hay	Positive	cellular infiltration of cells	1.26E-07	139
Liver	Alfalfa hay	Positive	proliferation of blood cells	1.76E-07	271
Liver	Alfalfa hay	Positive	differentiation of leukocytes	2.03E-07	221
Liver	Alfalfa hay	Positive	synthesis of fatty acid	2.03E-07	112
Liver	Alfalfa hay	Positive	apoptosis of fibroblast cell lines	2.03E-07	117
Liver	Alfalfa hay	Positive	cell death of sarcoma cell lines	2.68E-07	85
Liver	Alfalfa hay	Positive	abnormal morphology of blood vessel	2.82E-07	97
Liver	Alfalfa hay	Positive	cellular infiltration	3.10E-07	150
Liver	Alfalfa hay	Positive	proliferation of immune cells	3.20E-07	255
Liver	Alfalfa hay	Positive	cell viability of tumor cell lines	3.40E-07	251
Liver	Alfalfa	Positive	quantity of carbohydrate	3.52E-07	174

	hay				
Liver	Alfalfa hay	Positive	formation of skin	3.57E-07	128
Liver	Alfalfa hay	Positive	degeneration of cells	3.75E-07	121
Liver	Alfalfa hay	Positive	cell death of cervical cancer cell lines	3.75E-07	123
Liver	Alfalfa hay	Positive	advanced malignant tumor	3.95E-07	270
Liver	Alfalfa hay	Positive	metabolism of membrane lipid derivative	4.54E-07	134
Liver	Alfalfa hay	Positive	differentiation of blood cells	4.65E-07	269
Liver	Alfalfa hay	Positive	uptake of D-hexose	4.82E-07	83
Liver	Alfalfa hay	Positive	morphology of connective tissue	4.92E-07	156
Liver	Alfalfa hay	Positive	concentration of phosphatidic acid	4.92E-07	55
Liver	Alfalfa hay	Positive	survival of organism	4.94E-07	228
Liver	Alfalfa hay	Positive	growth of tumor	5.07E-07	255
Liver	Alfalfa hay	Positive	production of reactive oxygen species	5.51E-07	127
Liver	Alfalfa hay	Positive	phosphorylation of protein	5.81E-07	237
Liver	Alfalfa hay	Positive	uptake of D-glucose	5.81E-07	82
Liver	Alfalfa hay	Positive	contractility of myocardium	5.98E-07	30
Liver	Alfalfa hay	Positive	disorder of basal ganglia	6.14E-07	246
Liver	Alfalfa hay	Positive	cell movement of phagocytes	6.36E-07	182
Liver	Alfalfa hay	Positive	contractility of ventricular myocardium	6.38E-07	28
Liver	Alfalfa hay	Positive	quantity of metal ion	6.60E-07	150
Liver	Alfalfa hay	Positive	function of muscle	7.03E-07	121
Liver	Alfalfa hay	Positive	cell movement of myeloid cells	7.11E-07	179
Liver	Alfalfa	Positive	metastatic colorectal cancer	7.11E-07	73

	hay				
Liver	Alfalfa hay	Positive	autophagy	7.81E-07	129
Liver	Alfalfa hay	Positive	formation of filaments	7.83E-07	136
Liver	Alfalfa hay	Positive	chemotaxis	8.99E-07	180
Liver	Alfalfa hay	Positive	metastasis	8.99E-07	243
Liver	Alfalfa hay	Positive	quantity of metal	9.26E-07	166
Liver	Alfalfa hay	Positive	neuronal cell death	9.71E-07	220
Liver	Alfalfa hay	Positive	morphology of heart	9.71E-07	138
Liver	Alfalfa hay	Positive	cardiogenesis	9.78E-07	158
Liver	Alfalfa hay	Positive	seizures	9.83E-07	136
Liver	Alfalfa hay	Positive	development of cardiovascular tissue	9.91E-07	127
Liver	Alfalfa hay	Positive	growth of muscle tissue	1.05E-06	128
Liver	Alfalfa hay	Positive	mitosis	1.08E-06	165
Liver	Alfalfa hay	Positive	development of digestive system	1.08E-06	131
Liver	Alfalfa hay	Positive	morphology of lymphatic system component	1.08E-06	141
Liver	Alfalfa hay	Positive	abnormal morphology of abdomen	1.08E-06	276
Liver	Alfalfa hay	Positive	cell spreading of tumor cell lines	1.09E-06	45
Liver	Alfalfa hay	Positive	chemotaxis of cells	1.23E-06	174
Liver	Alfalfa hay	Positive	growth of lymphatic system component	1.24E-06	72
Liver	Alfalfa hay	Positive	proliferation of muscle cells	1.24E-06	127
Liver	Alfalfa hay	Positive	morphology of muscle	1.39E-06	116
Liver	Alfalfa hay	Positive	mammary tumor	1.48E-06	488
Liver	Alfalfa	Positive	formation of eye	1.55E-06	147

	hay				
Liver	Alfalfa hay	Positive	neurological signs	1.55E-06	210
Liver	Alfalfa hay	Positive	quantity of myeloid cells	1.55E-06	122
Liver	Alfalfa hay	Positive	angiogenesis of lesion	1.70E-06	51
Liver	Alfalfa hay	Positive	branching of cells	1.71E-06	126
Liver	Alfalfa hay	Positive	synthesis of reactive oxygen species	1.87E-06	159
Liver	Alfalfa hay	Positive	epilepsy	1.88E-06	107
Liver	Alfalfa hay	Positive	transactivation	1.98E-06	188
Liver	Alfalfa hay	Positive	development of leukocytes	2.08E-06	191
Liver	Alfalfa hay	Positive	infection by Retroviridae	2.15E-06	229
Liver	Alfalfa hay	Positive	cell cycle progression	2.25E-06	322
Liver	Alfalfa hay	Positive	quantity of Ca2+	2.28E-06	137
Liver	Alfalfa hay	Positive	phosphorylation of amino acids	2.36E-06	80
Liver	Alfalfa hay	Positive	skin abnormality	2.58E-06	82
Liver	Alfalfa hay	Positive	abnormal morphology of epithelial tissue	2.58E-06	140
Liver	Alfalfa hay	Positive	T cell homeostasis	2.66E-06	169
Liver	Alfalfa hay	Positive	T cell development	2.66E-06	165
Liver	Alfalfa hay	Positive	morphology of heart ventricle	2.67E-06	71
Liver	Alfalfa hay	Positive	metastatic malignant neoplasm of digestive system	2.71E-06	84
Liver	Alfalfa hay	Positive	growth of epithelial tissue	2.71E-06	217
Liver	Alfalfa hay	Positive	adhesion of immune cells	2.71E-06	119
Liver	Alfalfa hay	Positive	development of endothelial tissue	2.77E-06	124
Liver	Alfalfa	Positive	Organ Degeneration	2.96E-06	162

	hay				
Liver	Alfalfa hay	Positive	Huntington's Disease	2.96E-06	186
Liver	Alfalfa hay	Positive	Growth Failure	2.98E-06	193
Liver	Alfalfa hay	Positive	apoptosis of hepatoma cell lines	3.08E-06	58
Liver	Alfalfa hay	Positive	Gastrointestinal Tract Cancer and Tumors	3.08E-06	1420
Liver	Alfalfa hay	Positive	metastatic gastrointestinal tract cancer	3.19E-06	80
Liver	Alfalfa hay	Positive	Lymphocyte homeostasis	3.34E-06	177
Liver	Alfalfa hay	Positive	dyskinesia	3.44E-06	198
Liver	Alfalfa hay	Positive	migration of endothelial cells	3.83E-06	112
Liver	Alfalfa hay	Positive	proliferation of hematopoietic progenitor cells	3.93E-06	86
Liver	Alfalfa hay	Positive	phosphorylation of L-amino acid	3.95E-06	79
Liver	Alfalfa hay	Positive	development of blood cells	4.16E-06	208
Liver	Alfalfa hay	Positive	cell death of epithelial cell lines	4.16E-06	98
Liver	Alfalfa hay	Positive	proliferation of lymphatic system cells	4.19E-06	73
Liver	Alfalfa hay	Positive	morphology of lymphoid organ	4.26E-06	131
Liver	Alfalfa hay	Positive	recruitment of phagocytes	4.28E-06	87
Liver	Alfalfa hay	Positive	Neurodegeneration	4.29E-06	106
Liver	Alfalfa hay	Positive	invasion of prostate cancer cell lines	4.29E-06	42
Liver	Alfalfa hay	Positive	metabolism of reactive oxygen species	4.40E-06	163
Liver	Alfalfa hay	Positive	gastrointestinal tract cancer	4.82E-06	1396
Liver	Alfalfa hay	Positive	homeostasis of leukocytes	4.96E-06	179
Liver	Alfalfa hay	Positive	morphology of bone	4.96E-06	148
Liver	Alfalfa	Positive	contractility of heart	5.22E-06	65

	hay				
Liver	Alfalfa hay	Positive	quantity of T lymphocytes	5.35E-06	173
Liver	Alfalfa hay	Positive	adhesion of blood cells	5.35E-06	126
Liver	Alfalfa hay	Positive	proliferation of neuronal cells	5.35E-06	185
Liver	Alfalfa hay	Positive	cell movement of endothelial cells	5.43E-06	119
Liver	Alfalfa hay	Positive	endothelial cell development	5.43E-06	119
Liver	Alfalfa hay	Positive	cell movement of granulocytes	5.43E-06	124
Liver	Alfalfa hay	Positive	infection by lentivirus	5.61E-06	225
Liver	Alfalfa hay	Positive	Bleeding	5.80E-06	140
Liver	Alfalfa hay	Positive	proliferation of hematopoietic cells	5.87E-06	89
Liver	Alfalfa hay	Positive	recruitment of leukocytes	6.15E-06	110
Liver	Alfalfa hay	Positive	protein kinase cascade	6.22E-06	139
Liver	Alfalfa hay	Positive	development of lymphocytes	6.25E-06	178
Liver	Alfalfa hay	Positive	replication of RNA virus	6.46E-06	164
Liver	Alfalfa hay	Positive	HIV infection	6.46E-06	224
Liver	Alfalfa hay	Positive	development of sensory organ	6.46E-06	181
Liver	Alfalfa hay	Positive	adenocarcinoma	6.46E-06	1771
Liver	Alfalfa hay	Positive	morphology of skin	6.66E-06	94
Liver	Alfalfa hay	Positive	development of mononuclear leukocytes	6.84E-06	179
Liver	Alfalfa hay	Positive	apoptosis of epithelial cells	6.84E-06	98
Liver	Alfalfa hay	Positive	plasma cell dyscrasia	7.13E-06	113
Liver	Alfalfa hay	Positive	accumulation of cells	7.21E-06	118
Liver	Alfalfa	Positive	angiogenesis of tumor	7.25E-06	47

	hay				
Liver	Alfalfa hay	Positive	cell death of pheochromocytoma cell lines	7.25E-06	47
Liver	Alfalfa hay	Positive	congenital malformation of skeleton	7.50E-06	139
Liver	Alfalfa hay	Positive	proliferation of mononuclear leukocytes	7.50E-06	231
Liver	Alfalfa hay	Positive	neuromuscular disease	8.07E-06	272
Liver	Alfalfa hay	Positive	quantity of antigen presenting cells	8.16E-06	95
Liver	Alfalfa hay	Positive	apoptosis of cardiomyocytes	8.16E-06	67
Liver	Alfalfa hay	Positive	metabolism of phospholipid	8.29E-06	82
Liver	Alfalfa hay	Positive	recruitment of cells	8.39E-06	119
Liver	Alfalfa hay	Positive	adenoma	8.56E-06	177
Liver	Alfalfa hay	Positive	interphase	8.74E-06	207
Liver	Alfalfa hay	Positive	perinatal death	9.02E-06	189
Liver	Alfalfa hay	Positive	breast or ovarian cancer	9.25E-06	555
Liver	Alfalfa hay	Positive	secondary neoplasm of digestive system	9.25E-06	88
Liver	Alfalfa hay	Positive	abdominal adenocarcinoma	9.31E-06	1614
Liver	Alfalfa hay	Positive	intestinal tumor	9.78E-06	1208
Liver	Alfalfa hay	Positive	adhesion of tumor cell lines	9.99E-06	98
Liver	Alfalfa hay	Positive	proliferation of lymphocytes	9.99E-06	227
Liver	Alfalfa hay	Positive	congenital anomaly of skin	1.02E-05	65
Liver	Alfalfa hay	Positive	abnormal morphology of lymphoid organ	1.08E-05	125
Liver	Alfalfa hay	Positive	apoptosis of heart cells	1.11E-05	68
Liver	Alfalfa hay	Positive	cell death of hepatoma cell lines	1.11E-05	62
Liver	Alfalfa	Positive	metabolism of phosphatidic acid	1.11E-05	62

	hay				
Liver	Alfalfa hay	Positive	apoptosis of cervical cancer cell lines	1.13E-05	96
Liver	Alfalfa hay	Positive	morphology of blood cells	1.13E-05	136
Liver	Alfalfa hay	Positive	cell death of blood cells	1.13E-05	217
Liver	Alfalfa hay	Positive	female genital tract serous cancer	1.14E-05	127
Liver	Alfalfa hay	Positive	cell movement of fibroblasts	1.17E-05	66
Liver	Alfalfa hay	Positive	concentration of phospholipid	1.17E-05	66
Liver	Alfalfa hay	Positive	accumulation of lipid	1.19E-05	101
Liver	Alfalfa hay	Positive	dysmyelination	1.21E-05	51
Liver	Alfalfa hay	Positive	colorectal neoplasia	1.25E-05	1203
Liver	Alfalfa hay	Positive	vascularization	1.26E-05	82
Liver	Alfalfa hay	Positive	apoptosis of connective tissue cells	1.27E-05	100
Liver	Alfalfa hay	Positive	infection by RNA virus	1.28E-05	266
Liver	Alfalfa hay	Positive	differentiation of epithelial tissue	1.28E-05	119
Liver	Alfalfa hay	Positive	activation of cells	1.37E-05	297
Liver	Alfalfa hay	Positive	small GTPase mediated signal transduction	1.38E-05	66
Liver	Alfalfa hay	Positive	synthesis of nitric oxide	1.44E-05	94
Liver	Alfalfa hay	Positive	quantity of phagocytes	1.45E-05	138
Liver	Alfalfa hay	Positive	degeneration of nervous system	1.55E-05	97
Liver	Alfalfa hay	Positive	replication of virus	1.55E-05	178
Liver	Alfalfa hay	Positive	apoptosis of epithelial cell lines	1.55E-05	78
Liver	Alfalfa hay	Positive	function of lymphocytes	1.56E-05	113
Liver	Alfalfa	Positive	cell viability of cervical cancer cell lines	1.64E-05	77

	hay				
Liver	Alfalfa hay	Positive	paralysis	1.65E-05	60
Liver	Alfalfa hay	Positive	differentiation of embryonic tissue	1.68E-05	87
Liver	Alfalfa hay	Positive	demyelination	1.73E-05	46
Liver	Alfalfa hay	Positive	binding of DNA	1.73E-05	166
Liver	Alfalfa hay	Positive	proliferation of tumor cells	1.77E-05	151
Liver	Alfalfa hay	Positive	concentration of acylglycerol	1.79E-05	111
Liver	Alfalfa hay	Positive	cell death of tumor	1.79E-05	125
Liver	Alfalfa hay	Positive	migration of tumor cells	1.79E-05	78
Liver	Alfalfa hay	Positive	cell death of skin	1.79E-05	41
Liver	Alfalfa hay	Positive	autophagy of cells	1.81E-05	90
Liver	Alfalfa hay	Positive	abnormal morphology of bone	1.88E-05	141
Liver	Alfalfa hay	Positive	ubiquitination of protein	1.90E-05	110
Liver	Alfalfa hay	Positive	differentiation of tumor cell lines	1.91E-05	124
Liver	Alfalfa hay	Positive	Encephalitis	1.94E-05	99
Liver	Alfalfa hay	Positive	formation of filopodia	1.94E-05	60
Liver	Alfalfa hay	Positive	proliferation of embryonic cells	2.15E-05	85
Liver	Alfalfa hay	Positive	keratosis	2.15E-05	46
Liver	Alfalfa hay	Positive	development of lymphatic system component	2.15E-05	103
Liver	Alfalfa hay	Positive	synthesis of DNA	2.17E-05	135
Liver	Alfalfa hay	Positive	cellular infiltration by granulocytes	2.17E-05	74
Liver	Alfalfa hay	Positive	inflammation of central nervous system	2.17E-05	101
Liver	Alfalfa	Positive	abnormal morphology of heart	2.18E-05	125

	hay				
Liver	Alfalfa hay	Positive	development of lymphatic system	2.27E-05	117
Liver	Alfalfa hay	Positive	necrosis of tumor	2.35E-05	124
Liver	Alfalfa hay	Positive	contractility of heart ventricle	2.36E-05	32
Liver	Alfalfa hay	Positive	cell movement of mononuclear leukocytes	2.39E-05	148
Liver	Alfalfa hay	Positive	ubiquitination	2.49E-05	111
Liver	Alfalfa hay	Positive	melanoma	2.61E-05	1798
Liver	Alfalfa hay	Positive	development of connective tissue	2.64E-05	121
Liver	Alfalfa hay	Positive	cell death of immune cells	2.67E-05	205
Liver	Alfalfa hay	Positive	differentiation of connective tissue	2.68E-05	225
Liver	Alfalfa hay	Positive	stress response of cells	2.73E-05	51
Liver	Alfalfa hay	Positive	apoptosis of dermal cells	2.76E-05	36
Liver	Alfalfa hay	Positive	epileptic seizure	2.77E-05	60
Liver	Alfalfa hay	Positive	infiltration by neutrophils	2.85E-05	59
Liver	Alfalfa hay	Positive	migration of prostate cancer cell lines	2.91E-05	41
Liver	Alfalfa hay	Positive	blood protein disorder	3.02E-05	122
Liver	Alfalfa hay	Positive	arrest in interphase	3.14E-05	134
Liver	Alfalfa hay	Positive	cell death of embryonic cells	3.23E-05	44
Liver	Alfalfa hay	Positive	contractility of cardiac muscle	3.27E-05	53
Liver	Alfalfa hay	Positive	proliferation of myeloid cells	3.28E-05	45
Liver	Alfalfa hay	Positive	transactivation of RNA	3.30E-05	171
Liver	Alfalfa hay	Positive	craniofacial abnormality	3.38E-05	116
Liver	Alfalfa	Positive	export of molecule	3.38E-05	98

	hay				
Liver	Alfalfa hay	Positive	metastatic solid tumor	3.52E-05	139
Liver	Alfalfa hay	Positive	proliferation of smooth muscle cells	3.67E-05	95
Liver	Alfalfa hay	Positive	inflammatory response	3.67E-05	238
Liver	Alfalfa hay	Positive	intestinal cancer	3.79E-05	1183
Liver	Alfalfa hay	Positive	immune response of cells	3.83E-05	179
Liver	Alfalfa hay	Positive	gastrointestinal carcinoma	3.84E-05	1245
Liver	Alfalfa hay	Positive	recruitment of neutrophils	3.84E-05	65
Liver	Alfalfa hay	Positive	development of abdomen	3.85E-05	181
Liver	Alfalfa hay	Positive	apoptosis of skin	3.86E-05	37
Liver	Alfalfa hay	Positive	morphology of leukocytes	4.02E-05	112
Liver	Alfalfa hay	Positive	morphology of head	4.08E-05	265
Liver	Alfalfa hay	Positive	cell movement of connective tissue cells	4.08E-05	76
Liver	Alfalfa hay	Positive	cell death of dermal cells	4.10E-05	38
Liver	Alfalfa hay	Positive	growth of plasma membrane projections	4.12E-05	148
Liver	Alfalfa hay	Positive	concentration of fatty acid	4.13E-05	93
Liver	Alfalfa hay	Positive	apoptosis of neurons	4.19E-05	139
Liver	Alfalfa hay	Positive	cell movement of lymphocytes	4.24E-05	127
Liver	Alfalfa hay	Positive	apoptosis of sarcoma cell lines	4.25E-05	66
Liver	Alfalfa hay	Positive	cell death of bone cancer cell lines	4.25E-05	62
Liver	Alfalfa hay	Positive	synthesis of phospholipid	4.25E-05	62
Liver	Alfalfa hay	Positive	metastasis of cells	4.36E-05	84
Liver	Alfalfa	Positive	advanced stage solid tumor	4.52E-05	162

	hay				
Liver	Alfalfa hay	Positive	demyelination of central nervous system	4.62E-05	21
Liver	Alfalfa hay	Positive	apoptosis of pheochromocytoma cell lines	4.63E-05	36
Liver	Alfalfa hay	Positive	quantity of bone	4.64E-05	83
Liver	Alfalfa hay	Positive	cell death of tumor cells	4.66E-05	120
Liver	Alfalfa hay	Positive	autosomal dominant disease	4.67E-05	197
Liver	Alfalfa hay	Positive	abnormal morphology of skin	4.94E-05	82
Liver	Alfalfa hay	Positive	quantity of reactive oxygen species	5.03E-05	62
Liver	Alfalfa hay	Positive	abnormal morphology of leukocytes	5.04E-05	77
Liver	Alfalfa hay	Positive	quantity of IL-6 in blood	5.19E-05	34
Liver	Alfalfa hay	Positive	colorectal cancer	5.27E-05	1179
Liver	Alfalfa hay	Positive	abnormal morphology of head	5.29E-05	252
Liver	Alfalfa hay	Positive	cleavage of cells	5.35E-05	55
Liver	Alfalfa hay	Positive	quantity of B lymphocytes	5.46E-05	112
Liver	Alfalfa hay	Positive	migration of cancer cells	5.53E-05	67
Liver	Alfalfa hay	Positive	infection of cells	5.60E-05	242
Liver	Alfalfa hay	Positive	differentiation of bone	5.62E-05	131
Liver	Alfalfa hay	Positive	growth of neurites	5.62E-05	146
Liver	Alfalfa hay	Positive	cell death of fibroblasts	5.63E-05	88
Liver	Alfalfa hay	Positive	cell death of heart	5.75E-05	77
Liver	Alfalfa hay	Positive	differentiation of connective tissue cells	5.82E-05	198
Liver	Alfalfa hay	Positive	quantity of epithelial tissue	5.86E-05	62
Liver	Alfalfa	Positive	quantity of lymphatic system cells	5.90E-05	91

	hay				
Liver	Alfalfa hay	Positive	necrosis of cardiac muscle	5.90E-05	74
Liver	Alfalfa hay	Positive	size of embryo	5.95E-05	115
Liver	Alfalfa hay	Positive	morphology of reproductive system	5.95E-05	167
Liver	Alfalfa hay	Positive	cell death of B-lymphocyte derived cell lines	6.02E-05	47
Liver	Alfalfa hay	Positive	abnormal morphology of muscle	6.64E-05	103
Liver	Alfalfa hay	Positive	quantity of granulocytes	6.64E-05	103
Liver	Alfalfa hay	Positive	quantity of diacylglycerol	6.80E-05	25
Liver	Alfalfa hay	Positive	development of gastrointestinal tract	6.80E-05	87
Liver	Alfalfa hay	Positive	hypersensitive reaction	6.80E-05	136
Liver	Alfalfa hay	Positive	size of animal	6.88E-05	62
Liver	Alfalfa hay	Positive	proliferation of hepatoma cell lines	7.07E-05	68
Liver	Alfalfa hay	Positive	serous neoplasm	7.14E-05	188
Liver	Alfalfa hay	Positive	abnormal morphology of reproductive system	7.14E-05	159
Liver	Alfalfa hay	Positive	formation of leukocytes	7.32E-05	56
Liver	Alfalfa hay	Positive	activation of neuroglia	7.32E-05	48
Liver	Alfalfa hay	Positive	colony formation of cells	7.36E-05	154
Liver	Alfalfa hay	Positive	cell death of neuroglia	7.41E-05	44
Liver	Alfalfa hay	Positive	growth of lymphoid organ	7.43E-05	60
Liver	Alfalfa hay	Positive	cognition	7.59E-05	143
Liver	Alfalfa hay	Positive	differentiation of T lymphocytes	7.83E-05	121
Liver	Alfalfa hay	Positive	proliferation of fibroblast cell lines	7.83E-05	134
Liver	Alfalfa	Positive	formation of muscle cells	8.04E-05	62

	hay				
Liver	Alfalfa hay	Positive	proliferation of T lymphocytes	8.25E-05	186
Liver	Alfalfa hay	Positive	Dermatitis	8.27E-05	137
Liver	Alfalfa hay	Positive	organization of actin cytoskeleton	8.33E-05	98
Liver	Alfalfa hay	Positive	arthropathy	8.33E-05	284
Liver	Alfalfa hay	Positive	morphology of respiratory system	8.41E-05	101
Liver	Alfalfa hay	Positive	proliferation of endothelial cells	8.41E-05	101
Liver	Alfalfa hay	Positive	behavior	8.41E-05	294
Liver	Alfalfa hay	Positive	differentiation of bone cells	8.48E-05	129
Liver	Alfalfa hay	Positive	cytostasis of tumor cell lines	8.58E-05	56
Liver	Alfalfa hay	Positive	morphology of cardiovascular tissue	8.59E-05	34
Liver	Alfalfa hay	Positive	encephalomyelitis	8.61E-05	85
Liver	Alfalfa hay	Positive	apoptosis of cerebral cortex cells	8.64E-05	40
Liver	Alfalfa hay	Positive	cell death of heart cells	8.84E-05	74
Liver	Alfalfa hay	Positive	function of cardiac muscle	8.84E-05	63
Liver	Alfalfa hay	Positive	quantity of protein in blood	8.89E-05	158
Liver	Alfalfa hay	Positive	learning	8.91E-05	132
Liver	Alfalfa hay	Positive	abnormal morphology of lymphocytes	8.95E-05	59
Liver	Alfalfa hay	Positive	inflammation of organ	9.28E-05	345
Liver	Alfalfa hay	Positive	production of antibody	9.29E-05	101
Liver	Alfalfa hay	Positive	breast cancer	9.48E-05	441
Liver	Alfalfa hay	Positive	cell death of embryonic stem cells	9.51E-05	22
Liver	Alfalfa	Positive	cell death of cardiomyocytes	9.91E-05	72

	hay				
Liver	Alfalfa hay	Positive	quantity of hematopoietic cells	1.01E-04	139
Liver	Alfalfa hay	Positive	quantity of lymphatic system component	1.01E-04	120
Liver	Alfalfa hay	Positive	formation of lymphatic system component	1.01E-04	95
Liver	Alfalfa hay	Positive	infection by HIV-1	1.01E-04	187
Liver	Alfalfa hay	Positive	development of central nervous system	1.01E-04	195
Liver	Alfalfa hay	Positive	colony formation	1.01E-04	166
Liver	Alfalfa hay	Positive	Breast Cancer and Tumors	1.01E-04	442
Liver	Alfalfa hay	Positive	apoptosis of muscle	1.02E-04	86
Liver	Alfalfa hay	Positive	apoptosis of endothelial cells	1.02E-04	63
Liver	Alfalfa hay	Positive	cell death of carcinoma cell lines	1.02E-04	93
Liver	Alfalfa hay	Positive	morphology of nervous system	1.06E-04	236
Liver	Alfalfa hay	Positive	growth of skin	1.06E-04	73
Liver	Alfalfa hay	Positive	cell movement of muscle cells	1.06E-04	62
Liver	Alfalfa hay	Positive	migration of connective tissue cells	1.06E-04	62
Liver	Alfalfa hay	Positive	activation of enzyme	1.06E-04	123
Liver	Alfalfa hay	Positive	abdominal carcinoma	1.07E-04	1621
Liver	Alfalfa hay	Positive	hemorrhagic disease	1.09E-04	83
Liver	Alfalfa hay	Positive	neuritogenesis	1.14E-04	179
Liver	Alfalfa hay	Positive	accumulation of blood cells	1.17E-04	91
Liver	Alfalfa hay	Positive	hypertrophy of heart	1.18E-04	108
Liver	Alfalfa hay	Positive	abnormal morphology of respiratory system	1.18E-04	99
Liver	Alfalfa	Positive	cell movement of tumor cells	1.18E-04	63

	hay				
Liver	Alfalfa hay	Positive	cell death of myeloma cell lines	1.20E-04	36
Liver	Alfalfa hay	Positive	degeneration of muscle cells	1.22E-04	23
Liver	Alfalfa hay	Positive	synthesis of phosphatidic acid	1.23E-04	49
Liver	Alfalfa hay	Positive	movement of vascular endothelial cells	1.24E-04	62
Liver	Alfalfa hay	Positive	quantity of hematopoietic progenitor cells	1.27E-04	138
Liver	Alfalfa hay	Positive	ion homeostasis of cells	1.29E-04	171
Liver	Alfalfa hay	Positive	damage of epithelial tissue	1.29E-04	39
Liver	Alfalfa hay	Positive	phagocytosis	1.29E-04	96
Liver	Alfalfa hay	Positive	apoptosis of fibrosarcoma cell lines	1.29E-04	25
Liver	Alfalfa hay	Positive	experimental autoimmune encephalomyelitis	1.29E-04	84
Liver	Alfalfa hay	Positive	morphogenesis of head	1.29E-04	84
Liver	Alfalfa hay	Positive	quantity of lymphoid organ	1.30E-04	99
Liver	Alfalfa hay	Positive	myelination	1.35E-04	66
Liver	Alfalfa hay	Positive	abnormal morphology of heart ventricle	1.35E-04	60
Liver	Alfalfa hay	Positive	apoptosis of muscle cells	1.37E-04	85
Liver	Alfalfa hay	Positive	differentiation of lymphocytes	1.37E-04	154
Liver	Alfalfa hay	Positive	generation of leukocytes	1.37E-04	56
Liver	Alfalfa hay	Positive	apoptosis of colon cancer cell lines	1.39E-04	79
Liver	Alfalfa hay	Positive	Hypertrophy	1.39E-04	147
Liver	Alfalfa hay	Positive	apoptosis of liver	1.42E-04	55
Liver	Alfalfa hay	Positive	production of protein	1.44E-04	105
Liver	Alfalfa	Positive	cell death of epidermal cells	1.44E-04	35

	hay				
Liver	Alfalfa hay	Positive	morphology of artery	1.48E-04	54
Liver	Alfalfa hay	Positive	quantity of connective tissue cells	1.48E-04	78
Liver	Alfalfa hay	Positive	mass of heart	1.48E-04	48
Liver	Alfalfa hay	Positive	vascularization of body region	1.48E-04	48
Liver	Alfalfa hay	Positive	apoptosis of hepatocytes	1.52E-04	47
Liver	Alfalfa hay	Positive	function of T lymphocytes	1.53E-04	85
Liver	Alfalfa hay	Positive	quantity of cytokine	1.55E-04	83
Liver	Alfalfa hay	Positive	cell movement of prostate cancer cell lines	1.57E-04	45
Liver	Alfalfa hay	Positive	apoptosis of myeloma cell lines	1.58E-04	32
Liver	Alfalfa hay	Positive	apoptosis of neuroblastoma cell lines	1.59E-04	44
Liver	Alfalfa hay	Positive	neovascularization	1.60E-04	56
Liver	Alfalfa hay	Positive	cell death of neuroblastoma cell lines	1.63E-04	65
Liver	Alfalfa hay	Positive	morphology of lymphocytes	1.63E-04	65
Liver	Alfalfa hay	Positive	adhesion of mononuclear leukocytes	1.63E-04	51
Liver	Alfalfa hay	Positive	cell death of breast cancer cell lines	1.63E-04	106
Liver	Alfalfa hay	Positive	growth of embryonic tissue	1.65E-04	89
Liver	Alfalfa hay	Positive	apoptosis of epidermal cells	1.66E-04	33
Liver	Alfalfa hay	Positive	abnormal morphology of nervous system	1.67E-04	218
Liver	Alfalfa hay	Positive	apoptosis of tumor cells	1.68E-04	92
Liver	Alfalfa hay	Positive	concentration of sterol	1.70E-04	98
Liver	Alfalfa hay	Positive	function of phagocytes	1.70E-04	98
Liver	Alfalfa	Positive	quantity of interleukin	1.72E-04	49

	hay				
Liver	Alfalfa hay	Positive	apoptosis of liver cells	1.72E-04	54
Liver	Alfalfa hay	Positive	estrous cycle	1.75E-04	30
Liver	Alfalfa hay	Positive	cell death of muscle	1.76E-04	109
Liver	Alfalfa hay	Positive	cellular infiltration of phagocytes	1.81E-04	60
Liver	Alfalfa hay	Positive	abnormal morphology of interalveolar septa	1.81E-04	19
Liver	Alfalfa hay	Positive	formation of connective tissue cells	1.85E-04	70
Liver	Alfalfa hay	Positive	apoptosis of fibroblasts	1.87E-04	72
Liver	Alfalfa hay	Positive	uterine serous papillary cancer	1.88E-04	76
Liver	Alfalfa hay	Positive	quantity of immunoglobulin	1.88E-04	95
Liver	Alfalfa hay	Positive	apoptosis of B-lymphocyte derived cell lines	1.91E-04	44
Liver	Alfalfa hay	Positive	morphology of genital organ	1.91E-04	132
Liver	Alfalfa hay	Positive	dysgenesis	1.94E-04	165
Liver	Alfalfa hay	Positive	chronic myeloid leukemia	1.94E-04	39
Liver	Alfalfa hay	Positive	uptake of 2-deoxyglucose	1.94E-04	39
Liver	Alfalfa hay	Positive	cytostasis	2.00E-04	91
Liver	Alfalfa hay	Positive	cell movement of macrophages	2.00E-04	86
Liver	Alfalfa hay	Positive	Hypoplasia	2.00E-04	155
Liver	Alfalfa hay	Positive	abnormal morphology of endothelial tissue	2.02E-04	26
Liver	Alfalfa hay	Positive	permeability of epithelial tissue	2.02E-04	26
Liver	Alfalfa hay	Positive	cell death of fibrosarcoma cell lines	2.11E-04	29
Liver	Alfalfa hay	Positive	apoptosis of cortical neurons	2.11E-04	33
Liver	Alfalfa	Positive	abnormal morphology of artery	2.19E-04	52

	hay				
Liver	Alfalfa hay	Positive	cell death of endothelial cells	2.19E-04	67
Liver	Alfalfa hay	Positive	morphology of mononuclear leukocytes	2.19E-04	67
Liver	Alfalfa hay	Positive	quantity of macrophages	2.19E-04	67
Liver	Alfalfa hay	Positive	synthesis of prostaglandin	2.19E-04	59
Liver	Alfalfa hay	Positive	arthritis	2.21E-04	276
Liver	Alfalfa hay	Positive	abnormal morphology of digestive system	2.25E-04	166
Liver	Alfalfa hay	Positive	atrophy of muscle	2.25E-04	51
Liver	Alfalfa hay	Positive	cell movement of epithelial cells	2.25E-04	51
Liver	Alfalfa hay	Positive	hyperkeratosis	2.25E-04	35
Liver	Alfalfa hay	Positive	secretion of molecule	2.25E-04	168
Liver	Alfalfa hay	Negative	cell death	1.06E-20	841
Liver	Alfalfa hay	Negative	necrosis	3.86E-20	672
Liver	Alfalfa hay	Negative	organismal death	4.09E-20	632
Liver	Alfalfa hay	Negative	apoptosis	4.09E-20	686
Liver	Alfalfa hay	Negative	morbidity or mortality	1.39E-19	636
Liver	Alfalfa hay	Negative	malignant solid tumor	5.74E-19	2294
Liver	Alfalfa hay	Negative	cancer	7.38E-19	2318
Liver	Alfalfa hay	Negative	proliferation of cells	1.18E-17	896
Liver	Alfalfa hay	Negative	morphology of cells	2.80E-15	509
Liver	Alfalfa hay	Negative	neoplasia of epithelial tissue	1.75E-14	1918
Liver	Alfalfa hay	Negative	cell death of tumor cell lines	1.86E-14	411
Liver	Alfalfa	Negative	tumorigenesis of tissue	1.86E-14	1946

	hay				
Liver	Alfalfa hay	Negative	epithelial cancer	5.34E-14	1898
Liver	Alfalfa hay	Negative	apoptosis of tumor cell lines	4.24E-13	333
Liver	Alfalfa hay	Negative	organization of cytoplasm	1.07E-12	404
Liver	Alfalfa hay	Negative	organization of cytoskeleton	1.07E-12	373
Liver	Alfalfa hay	Negative	cellular homeostasis	1.34E-12	401
Liver	Alfalfa hay	Negative	digestive organ tumor	1.64E-12	1653
Liver	Alfalfa hay	Negative	digestive system cancer	1.64E-12	1639
Liver	Alfalfa hay	Negative	abdominal neoplasm	2.87E-12	1889
Liver	Alfalfa hay	Negative	cell movement	1.79E-11	528
Liver	Alfalfa hay	Negative	quantity of cells	2.50E-11	449
Liver	Alfalfa hay	Negative	cell death of connective tissue cells	3.36E-11	180
Liver	Alfalfa hay	Negative	abdominal cancer	4.96E-11	1860
Liver	Alfalfa hay	Negative	cell survival	5.11E-11	364
Liver	Alfalfa hay	Negative	quantity of blood cells	2.61E-10	278
Liver	Alfalfa hay	Negative	quantity of leukocytes	2.68E-10	252
Liver	Alfalfa hay	Negative	development of lymphatic system	3.54E-10	114
Liver	Alfalfa hay	Negative	cell viability	3.54E-10	339
Liver	Alfalfa hay	Negative	differentiation of cells	4.13E-10	546
Liver	Alfalfa hay	Negative	transport of molecule	8.41E-10	406
Liver	Alfalfa hay	Negative	migration of cells	1.42E-09	468
Liver	Alfalfa hay	Negative	microtubule dynamics	2.04E-09	309
Liver	Alfalfa	Negative	expression of RNA	3.32E-09	518

	hay				
Liver	Alfalfa hay	Negative	development of leukocytes	3.80E-09	169
Liver	Alfalfa hay	Negative	Growth Failure	4.80E-09	171
Liver	Alfalfa hay	Negative	development of blood cells	4.99E-09	184
Liver	Alfalfa hay	Negative	abnormal morphology of cells	5.11E-09	325
Liver	Alfalfa hay	Negative	development of body trunk	7.43E-09	291
Liver	Alfalfa hay	Negative	quantity of lymphocytes	1.61E-08	195
Liver	Alfalfa hay	Negative	morphology of cardiovascular system	1.61E-08	174
Liver	Alfalfa hay	Negative	Movement Disorders	1.85E-08	276
Liver	Alfalfa hay	Negative	transcription	2.19E-08	480
Liver	Alfalfa hay	Negative	proliferation of hematopoietic progenitor cells	2.89E-08	79
Liver	Alfalfa hay	Negative	quantity of mononuclear leukocytes	3.63E-08	200
Liver	Alfalfa hay	Negative	proliferation of blood cells	3.63E-08	225
Liver	Alfalfa hay	Negative	disorder of basal ganglia	4.36E-08	207
Liver	Alfalfa hay	Negative	perinatal death	4.36E-08	166
Liver	Alfalfa hay	Negative	quantity of connective tissue	4.36E-08	168
Liver	Alfalfa hay	Negative	development of vasculature	4.36E-08	123
Liver	Alfalfa hay	Negative	growth of organism	4.36E-08	223
Liver	Alfalfa hay	Negative	abnormal morphology of thoracic cavity	4.54E-08	164
Liver	Alfalfa hay	Negative	transcription of RNA	4.79E-08	448
Liver	Alfalfa hay	Negative	Viral Infection	5.18E-08	395
Liver	Alfalfa hay	Negative	cell movement of tumor cell lines	5.44E-08	226
Liver	Alfalfa	Negative	development of lymphatic system component	5.81E-08	95

	hay				
Liver	Alfalfa hay	Negative	neuronal cell death	6.02E-08	186
Liver	Alfalfa hay	Negative	proliferation of hematopoietic cells	6.26E-08	81
Liver	Alfalfa hay	Negative	homeostasis of leukocytes	9.38E-08	155
Liver	Alfalfa hay	Negative	abnormal morphology of cardiovascular system	9.55E-08	158
Liver	Alfalfa hay	Negative	growth of connective tissue	1.13E-07	176
Liver	Alfalfa hay	Negative	development of lymphocytes	1.35E-07	154
Liver	Alfalfa hay	Negative	Huntington's Disease	1.39E-07	159
Liver	Alfalfa hay	Negative	dyskinesia	1.43E-07	169
Liver	Alfalfa hay	Negative	proliferation of immune cells	1.50E-07	210
Liver	Alfalfa hay	Negative	abnormal morphology of body cavity	1.57E-07	289
Liver	Alfalfa hay	Negative	vasculogenesis	1.57E-07	197
Liver	Alfalfa hay	Negative	size of body	1.90E-07	223
Liver	Alfalfa hay	Negative	development of hematopoietic system	1.97E-07	78
Liver	Alfalfa hay	Negative	morphology of body cavity	1.98E-07	300
Liver	Alfalfa hay	Negative	degeneration of cells	2.00E-07	102
Liver	Alfalfa hay	Negative	Lymphocyte homeostasis	2.12E-07	151
Liver	Alfalfa hay	Negative	cell death of blood cells	2.30E-07	186
Liver	Alfalfa hay	Negative	proliferation of connective tissue cells	2.70E-07	162
Liver	Alfalfa hay	Negative	cell death of fibroblast cell lines	2.70E-07	122
Liver	Alfalfa hay	Negative	degeneration of nervous system	3.16E-07	87
Liver	Alfalfa hay	Negative	ubiquitination of protein	3.73E-07	98
Liver	Alfalfa	Negative	migration of tumor cell lines	3.95E-07	188

	hay				
Liver	Alfalfa hay	Negative	neurological signs	3.98E-07	175
Liver	Alfalfa hay	Negative	ubiquitination	4.42E-07	99
Liver	Alfalfa hay	Negative	abnormal morphology of epithelial tissue	4.65E-07	119
Liver	Alfalfa hay	Negative	Gastrointestinal Tract Cancer and Tumors	4.76E-07	1129
Liver	Alfalfa hay	Negative	gastrointestinal tract cancer	4.96E-07	1112
Liver	Alfalfa hay	Negative	quantity of T lymphocytes	4.96E-07	147
Liver	Alfalfa hay	Negative	formation of mononuclear leukocytes	5.25E-07	46
Liver	Alfalfa hay	Negative	T cell development	5.49E-07	139
Liver	Alfalfa hay	Negative	neurodegeneration of neurites	5.62E-07	35
Liver	Alfalfa hay	Negative	development of cytoplasm	6.30E-07	129
Liver	Alfalfa hay	Negative	apoptosis of fibroblast cell lines	7.47E-07	96
Liver	Alfalfa hay	Negative	damage of nervous system	7.93E-07	70
Liver	Alfalfa hay	Negative	formation of leukocytes	8.06E-07	53
Liver	Alfalfa hay	Negative	Neurodegeneration	8.95E-07	91
Liver	Alfalfa hay	Negative	chronic myeloid leukemia	9.43E-07	39
Liver	Alfalfa hay	Negative	cell death of immune cells	9.94E-07	175
Liver	Alfalfa hay	Negative	T cell homeostasis	1.05E-06	141
Liver	Alfalfa hay	Negative	cell death of cervical cancer cell lines	1.05E-06	101
Liver	Alfalfa hay	Negative	formation of lymphocytes	1.05E-06	44
Liver	Alfalfa hay	Negative	cellular degradation	1.07E-06	77
Liver	Alfalfa hay	Negative	transcription of DNA	1.08E-06	369
Liver	Alfalfa	Negative	neuromuscular disease	1.08E-06	226

	hay				
Liver	Alfalfa hay	Negative	degeneration of neurons	1.25E-06	73
Liver	Alfalfa hay	Negative	formation of cells	1.34E-06	248
Liver	Alfalfa hay	Negative	morphology of lymph node	1.37E-06	53
Liver	Alfalfa hay	Negative	neonatal death	1.41E-06	122
Liver	Alfalfa hay	Negative	growth of embryo	1.45E-06	131
Liver	Alfalfa hay	Negative	function of cardiovascular system	1.49E-06	105
Liver	Alfalfa hay	Negative	homing of cells	1.59E-06	150
Liver	Alfalfa hay	Negative	development of digestive system	1.85E-06	108
Liver	Alfalfa hay	Negative	angiogenesis	1.89E-06	230
Liver	Alfalfa hay	Negative	colorectal neoplasia	2.17E-06	959
Liver	Alfalfa hay	Negative	abnormal morphology of lymph node	2.23E-06	50
Liver	Alfalfa hay	Negative	function of blood cells	2.39E-06	156
Liver	Alfalfa hay	Negative	homing	2.40E-06	153
Liver	Alfalfa hay	Negative	chemotaxis of cells	2.41E-06	142
Liver	Alfalfa hay	Negative	breast or colorectal cancer	3.04E-06	1085
Liver	Alfalfa hay	Negative	intestinal tumor	3.20E-06	960
Liver	Alfalfa hay	Negative	differentiation of leukocytes	3.75E-06	175
Liver	Alfalfa hay	Negative	neurodegeneration of axons	3.75E-06	32
Liver	Alfalfa hay	Negative	proliferation of mononuclear leukocytes	3.96E-06	190
Liver	Alfalfa hay	Negative	differentiation of blood cells	3.96E-06	214
Liver	Alfalfa hay	Negative	gastrointestinal carcinoma	4.04E-06	994
Liver	Alfalfa	Negative	cell cycle progression	4.37E-06	259

	hay				
Liver	Alfalfa hay	Negative	chemotaxis	4.37E-06	145
Liver	Alfalfa hay	Negative	cell death of epithelial cell lines	4.41E-06	82
Liver	Alfalfa hay	Negative	size of embryo	4.53E-06	100
Liver	Alfalfa hay	Negative	Bleeding	5.42E-06	116
Liver	Alfalfa hay	Negative	autosomal dominant disease	5.53E-06	166
Liver	Alfalfa hay	Negative	mitosis	5.62E-06	133
Liver	Alfalfa hay	Negative	morphology of lymphatic system component	5.70E-06	114
Liver	Alfalfa hay	Negative	colorectal cancer	6.13E-06	942
Liver	Alfalfa hay	Negative	cell spreading	6.36E-06	80
Liver	Alfalfa hay	Negative	apoptosis of epithelial cell lines	6.47E-06	67
Liver	Alfalfa hay	Negative	quantity of hematopoietic cells	6.71E-06	120
Liver	Alfalfa hay	Negative	intestinal cancer	6.75E-06	943
Liver	Alfalfa hay	Negative	formation of cytoskeleton	6.81E-06	104
Liver	Alfalfa hay	Negative	proliferation of lymphocytes	7.04E-06	186
Liver	Alfalfa hay	Negative	quantity of metal	7.04E-06	133
Liver	Alfalfa hay	Negative	cell movement of leukocytes	7.24E-06	195
Liver	Alfalfa hay	Negative	Thrombosis	7.86E-06	53
Liver	Alfalfa hay	Negative	proliferation of fibroblasts	8.76E-06	98
Liver	Alfalfa hay	Negative	quantity of lymphoid organ	8.99E-06	87
Liver	Alfalfa hay	Negative	formation of cellular protrusions	8.99E-06	225
Liver	Alfalfa hay	Negative	abnormal morphology of lymphoid organ	9.07E-06	104
Liver	Alfalfa	Negative	quantity of hematopoietic progenitor cells	9.30E-06	119

	hay				
Liver	Alfalfa hay	Negative	morphology of bone	9.40E-06	121
Liver	Alfalfa hay	Negative	cell viability of tumor cell lines	9.51E-06	197
Liver	Alfalfa hay	Negative	cellular infiltration by leukocytes	1.03E-05	106
Liver	Alfalfa hay	Negative	colon cancer	1.15E-05	818
Liver	Alfalfa hay	Negative	formation of T lymphocytes	1.15E-05	39
Liver	Alfalfa hay	Negative	quantity of antigen presenting cells	1.19E-05	79
Liver	Alfalfa hay	Negative	growth of epithelial tissue	1.19E-05	174
Liver	Alfalfa hay	Negative	Hypertrophy	1.24E-05	126
Liver	Alfalfa hay	Negative	formation of lymphoid organ	1.26E-05	76
Liver	Alfalfa hay	Negative	colon tumor	1.28E-05	822
Liver	Alfalfa hay	Negative	development of epithelial tissue	1.32E-05	136
Liver	Alfalfa hay	Negative	cytopenia	1.34E-05	84
Liver	Alfalfa hay	Negative	abnormal morphology of embryonic tissue	1.50E-05	143
Liver	Alfalfa hay	Negative	phosphorylation of protein	1.52E-05	186
Liver	Alfalfa hay	Negative	growth of lymphatic system component	1.53E-05	58
Liver	Alfalfa hay	Negative	concentration of lipid	1.55E-05	203
Liver	Alfalfa hay	Negative	cognition	1.55E-05	121
Liver	Alfalfa hay	Negative	morphology of axial skeleton	1.64E-05	60
Liver	Alfalfa hay	Negative	adenocarcinoma	1.68E-05	1387
Liver	Alfalfa hay	Negative	morphology of lymphoid organ	1.79E-05	106
Liver	Alfalfa hay	Negative	cell movement of blood cells	1.79E-05	217
Liver	Alfalfa	Negative	function of leukocytes	1.90E-05	140

	hay				
Liver	Alfalfa hay	Negative	formation of lymphatic system component	1.90E-05	82
Liver	Alfalfa hay	Negative	formation of blood cells	1.95E-05	59
Liver	Alfalfa hay	Negative	cellular infiltration of cells	1.96E-05	107
Liver	Alfalfa hay	Negative	maturation of cells	1.97E-05	121
Liver	Alfalfa hay	Negative	morphology of connective tissue	1.97E-05	122
Liver	Alfalfa hay	Negative	differentiation of connective tissue	1.97E-05	184
Liver	Alfalfa hay	Negative	quantity of thymocytes	2.01E-05	67
Liver	Alfalfa hay	Negative	tumorigenesis of intestine	2.06E-05	819
Liver	Alfalfa hay	Negative	migration of blood cells	2.12E-05	216
Liver	Alfalfa hay	Negative	damage of nervous tissue	2.12E-05	32
Liver	Alfalfa hay	Negative	infection of cells	2.21E-05	199
Liver	Alfalfa hay	Negative	quantity of lymphatic system component	2.38E-05	102
Liver	Alfalfa hay	Negative	Edema	2.48E-05	94
Liver	Alfalfa hay	Negative	proliferation of myeloid cells	2.48E-05	39
Liver	Alfalfa hay	Negative	cell movement of myeloid cells	2.48E-05	140
Liver	Alfalfa hay	Negative	abnormal morphology of respiratory system	2.58E-05	85
Liver	Alfalfa hay	Negative	gastrointestinal adenocarcinoma	2.62E-05	931
Liver	Alfalfa hay	Negative	phosphorylation of L-amino acid	2.62E-05	64
Liver	Alfalfa hay	Negative	morphology of respiratory system	2.64E-05	86
Liver	Alfalfa hay	Negative	leukocyte migration	2.68E-05	215
Liver	Alfalfa hay	Negative	autophagy	2.77E-05	101
Liver	Alfalfa	Negative	quantity of metal ion	2.88E-05	117

	hay				
Liver	Alfalfa hay	Negative	damage of neurons	2.98E-05	31
Liver	Alfalfa hay	Negative	development of connective tissue	2.98E-05	100
Liver	Alfalfa hay	Negative	learning	3.05E-05	111
Liver	Alfalfa hay	Negative	development of hematopoietic progenitor cells	3.06E-05	57
Liver	Alfalfa hay	Negative	activation of cells	3.17E-05	238
Liver	Alfalfa hay	Negative	differentiation of connective tissue cells	3.17E-05	163
Liver	Alfalfa hay	Negative	leukopenia	3.22E-05	42
Liver	Alfalfa hay	Negative	morphology of vertebral column	3.28E-05	45
Liver	Alfalfa hay	Negative	peripheral vascular disease	3.28E-05	102
Liver	Alfalfa hay	Negative	proliferation of tumor cell lines	3.28E-05	360
Liver	Alfalfa hay	Negative	abdominal adenocarcinoma	3.28E-05	1263
Liver	Alfalfa hay	Negative	seizure disorder	3.28E-05	123
Liver	Alfalfa hay	Negative	development of hematopoietic cells	3.28E-05	58
Liver	Alfalfa hay	Negative	cell movement of muscle cells	3.28E-05	54
Liver	Alfalfa hay	Negative	metastatic colorectal cancer	3.42E-05	57
Liver	Alfalfa hay	Negative	formation of lung	3.47E-05	83
Liver	Alfalfa hay	Negative	infection by Retroviridae	3.53E-05	180
Liver	Alfalfa hay	Negative	development of lymphatic system cells	3.64E-05	36
Liver	Alfalfa hay	Negative	fibrogenesis	3.76E-05	109
Liver	Alfalfa hay	Negative	abnormal morphology of axial skeleton	3.93E-05	57
Liver	Alfalfa hay	Negative	damage of central nervous system	4.08E-05	56
Liver	Alfalfa	Negative	development of cardiovascular tissue	4.08E-05	99

	hay				
Liver	Alfalfa hay	Negative	colorectal carcinoma	4.08E-05	812
Liver	Alfalfa hay	Negative	metabolism of protein	4.18E-05	236
Liver	Alfalfa hay	Negative	apoptosis of cervical cancer cell lines	4.42E-05	78
Liver	Alfalfa hay	Negative	protein kinase cascade	4.48E-05	111
Liver	Alfalfa hay	Negative	endothelial cell development	4.64E-05	95
Liver	Alfalfa hay	Negative	abnormal morphology of membrane tissue	4.80E-05	51
Liver	Alfalfa hay	Negative	connective or soft tissue tumor	4.90E-05	176
Liver	Alfalfa hay	Negative	formation of thymus gland	5.04E-05	49
Liver	Alfalfa hay	Negative	infection by RNA virus	5.11E-05	212
Liver	Alfalfa hay	Negative	hypertrophy of heart	5.21E-05	91
Liver	Alfalfa hay	Negative	Inflammatory Bowel Disease	5.27E-05	72
Liver	Alfalfa hay	Negative	respiratory system development	5.27E-05	92
Liver	Alfalfa hay	Negative	abnormal morphology of abdomen	5.37E-05	214
Liver	Alfalfa hay	Negative	apoptosis of connective tissue cells	5.37E-05	81
Liver	Alfalfa hay	Negative	cell surface receptor linked signal transduction	5.37E-05	81
Liver	Alfalfa hay	Negative	quantity of lymphatic system cells	5.56E-05	76
Liver	Alfalfa hay	Negative	apoptosis of pheochromocytoma cell lines	5.69E-05	31
Liver	Alfalfa hay	Negative	morphology of skeleton	5.77E-05	77
Liver	Alfalfa hay	Negative	abnormal morphology of body wall	5.86E-05	34
Liver	Alfalfa hay	Negative	necrosis of epithelial tissue	6.01E-05	149
Liver	Alfalfa hay	Negative	development of phagocytes	6.33E-05	28
Liver	Alfalfa	Negative	secretory pathway	6.66E-05	57

	hay				
Liver	Alfalfa hay	Negative	cellular infiltration	6.68E-05	114
Liver	Alfalfa hay	Negative	cell death of pheochromocytoma cell lines	7.07E-05	38
Liver	Alfalfa hay	Negative	development of myeloid cells	7.13E-05	31
Liver	Alfalfa hay	Negative	metastatic gastrointestinal tract cancer	7.13E-05	63
Liver	Alfalfa hay	Negative	cell death of T lymphocytes	7.35E-05	85
Liver	Alfalfa hay	Negative	development of endothelial tissue	7.38E-05	97
Liver	Alfalfa hay	Negative	hemorrhagic disease	7.40E-05	70
Liver	Alfalfa hay	Negative	proliferation of T lymphocytes	7.46E-05	152
Liver	Alfalfa hay	Negative	long-term potentiation	7.46E-05	68
Liver	Alfalfa hay	Negative	cell death of fibroblasts	7.50E-05	73
Liver	Alfalfa hay	Negative	apoptosis of blood cells	7.77E-05	127
Liver	Alfalfa hay	Negative	formation of skin	7.99E-05	97
Liver	Alfalfa hay	Negative	Hypoplasia	8.22E-05	129
Liver	Alfalfa hay	Negative	quantity of B lymphocytes	8.23E-05	92
Liver	Alfalfa hay	Negative	cell death of mononuclear leukocytes	8.32E-05	104
Liver	Alfalfa hay	Negative	memory	8.51E-05	71
Liver	Alfalfa hay	Negative	survival of organism	8.51E-05	174
Liver	Alfalfa hay	Negative	generation of lymphocytes	8.51E-05	43
Liver	Alfalfa hay	Negative	morphology of vertebrae	8.51E-05	42
Liver	Alfalfa hay	Negative	cell death of hepatoma cell lines	8.51E-05	50
Liver	Alfalfa hay	Negative	formation of thymocytes	8.58E-05	34
Liver	Alfalfa	Negative	damage of brain	8.75E-05	53

	hay				
Liver	Alfalfa hay	Negative	cell death of epithelial cells	8.80E-05	127
Liver	Alfalfa hay	Negative	abnormal morphology of bone	8.87E-05	113
Liver	Alfalfa hay	Negative	synthesis of reactive oxygen species	9.09E-05	123
Liver	Alfalfa hay	Negative	colorectal adenocarcinoma	9.09E-05	786
Liver	Alfalfa hay	Negative	quantity of connective tissue cells	9.19E-05	66
Liver	Alfalfa hay	Negative	proliferation of neuronal cells	9.19E-05	145
Liver	Alfalfa hay	Negative	differentiation of lymphocytes	9.37E-05	127
Liver	Alfalfa hay	Negative	cell death of lymphocytes	9.43E-05	101
Liver	Alfalfa hay	Negative	development of gastrointestinal tract	9.65E-05	72
Liver	Alfalfa hay	Negative	seizures	1.04E-04	104
Liver	Alfalfa hay	Negative	quantity of Ca2+	1.04E-04	106
Liver	Alfalfa hay	Negative	abnormal morphology of skeleton	1.04E-04	74
Liver	Alfalfa hay	Negative	metabolism of reactive oxygen species	1.18E-04	127
Liver	Alfalfa hay	Negative	morphology of mouth	1.19E-04	41
Liver	Alfalfa hay	Negative	synthesis of nitric oxide	1.21E-04	75
Liver	Alfalfa hay	Negative	invasion of cells	1.21E-04	210
Liver	Alfalfa hay	Negative	dysgenesis	1.21E-04	136
Liver	Alfalfa hay	Negative	malignant connective or soft tissue neoplasm	1.21E-04	112
Liver	Alfalfa hay	Negative	metastatic malignant neoplasm of digestive system	1.23E-04	65
Liver	Alfalfa hay	Negative	HIV infection	1.23E-04	175
Liver	Alfalfa hay	Negative	phosphorylation of L-tyrosine	1.23E-04	51
Liver	Alfalfa	Negative	biosynthesis of nucleoside triphosphate	1.25E-04	34

	hay				
Liver	Alfalfa hay	Negative	growth of lymphoid organ	1.28E-04	50
Liver	Alfalfa hay	Negative	morphology of vessel	1.29E-04	82
Liver	Alfalfa hay	Negative	apoptosis of hepatoma cell lines	1.29E-04	45
Liver	Alfalfa hay	Negative	morphology of digestive system	1.31E-04	143
Liver	Alfalfa hay	Negative	cell movement of phagocytes	1.32E-04	138
Liver	Alfalfa hay	Negative	accumulation of lipid	1.35E-04	80
Liver	Alfalfa hay	Negative	metabolism of nucleoside triphosphate	1.35E-04	42
Liver	Alfalfa hay	Negative	hypertrophy of tissue	1.35E-04	70
Liver	Alfalfa hay	Negative	abnormal morphology of vertebrae	1.35E-04	41
Liver	Alfalfa hay	Negative	congenital anomaly of musculoskeletal system	1.35E-04	180
Liver	Alfalfa hay	Negative	oral cancer	1.36E-04	63
Liver	Alfalfa hay	Negative	cell viability of lymphocytes	1.38E-04	54
Liver	Alfalfa hay	Negative	formation of filaments	1.40E-04	103
Liver	Alfalfa hay	Negative	development of antigen presenting cells	1.43E-04	24
Liver	Alfalfa hay	Negative	midline defect	1.44E-04	72
Liver	Alfalfa hay	Negative	cardiogenesis	1.51E-04	120
Liver	Alfalfa hay	Negative	morphology of blood cells	1.51E-04	107
Liver	Alfalfa hay	Negative	skin abnormality	1.52E-04	63
Liver	Alfalfa hay	Negative	proliferation of embryonic cells	1.53E-04	68
Liver	Alfalfa hay	Negative	migration of muscle cells	1.54E-04	48
Liver	Alfalfa hay	Negative	abnormal morphology of vertebral column	1.55E-04	42
Liver	Alfalfa	Negative	quantity of epithelial tissue	1.57E-04	51

	hay				
Liver	Alfalfa hay	Negative	development of bone marrow	1.58E-04	38
Liver	Alfalfa hay	Negative	growth of muscle tissue	1.62E-04	97
Liver	Alfalfa hay	Negative	ovarian cancer	1.62E-04	174
Liver	Alfalfa hay	Negative	binding of DNA	1.62E-04	131
Liver	Alfalfa hay	Negative	apoptosis of breast cancer cell lines	1.64E-04	77
Liver	Alfalfa hay	Negative	chronic leukemia	1.64E-04	117
Liver	Alfalfa hay	Negative	hyperplasia of tissue	1.66E-04	55
Liver	Alfalfa hay	Negative	generation of cells	1.68E-04	71
Liver	Alfalfa hay	Negative	abnormal morphology of digestive system	1.86E-04	136
Liver	Alfalfa hay	Negative	cell viability of connective tissue cells	1.86E-04	40
Liver	Alfalfa hay	Negative	recruitment of antigen presenting cells	1.86E-04	37
Liver	Alfalfa hay	Negative	chronic myeloproliferative disorder	1.89E-04	46
Liver	Alfalfa hay	Negative	oral cavity carcinoma	1.89E-04	55
Liver	Alfalfa hay	Negative	cell movement of fibroblasts	1.94E-04	52
Liver	Alfalfa hay	Negative	shape change of tumor cell lines	1.94E-04	45
Liver	Alfalfa hay	Negative	hypertrophy of cells	1.99E-04	77
Liver	Alfalfa hay	Negative	potentiation of synapse	1.99E-04	44
Liver	Alfalfa hay	Negative	differentiation of mononuclear leukocytes	1.99E-04	135
Liver	Alfalfa hay	Negative	synthesis of lipid	2.01E-04	174
Liver	Alfalfa hay	Negative	morphology of heart	2.04E-04	104
Liver	Alfalfa hay	Negative	proliferation of muscle cells	2.04E-04	96
Liver	Alfalfa	Negative	metabolism of carbohydrate	2.11E-04	153

	hay				
Liver	Alfalfa hay	Negative	proliferation of smooth muscle cells	2.13E-04	76
Liver	Alfalfa hay	Negative	relapsed leukemia	2.13E-04	22
Liver	Alfalfa hay	Negative	small GTPase mediated signal transduction	2.18E-04	52
Liver	Alfalfa hay	Negative	proliferation of lymphatic system cells	2.27E-04	56
Liver	Alfalfa hay	Negative	morphology of nervous system	2.29E-04	189
Liver	Alfalfa hay	Negative	cell death of breast cancer cell lines	2.30E-04	87
Liver	Alfalfa hay	Negative	quantity of phagocytes	2.33E-04	108
Liver	Alfalfa hay	Negative	cell movement of connective tissue cells	2.37E-04	61
Liver	Alfalfa hay	Negative	activation of DNA endogenous promoter	2.46E-04	281
Liver	Alfalfa hay	Negative	recruitment of macrophages	2.47E-04	33
Liver	Alfalfa hay	Negative	Ovarian Cancer and Tumors	2.51E-04	183
Liver	Alfalfa hay	Negative	serous neoplasm	2.51E-04	150
Liver	Alfalfa hay	Negative	mesoderm development	2.51E-04	34
Liver	Alfalfa hay	Negative	abdominal carcinoma	2.57E-04	1268
Liver	Alfalfa hay	Negative	quantity of myeloid cells	2.57E-04	92
Liver	Alfalfa hay	Negative	migration of connective tissue cells	2.59E-04	51
Liver	Alfalfa hay	Negative	tumorigenesis of gonad	2.59E-04	194
Liver	Alfalfa hay	Negative	abnormal morphology of enlarged lymph node	2.63E-04	30
Liver	Alfalfa hay	Negative	cell viability of mononuclear leukocytes	2.68E-04	55
Liver	Alfalfa hay	Negative	cell transformation	2.69E-04	116
Liver	Alfalfa hay	Negative	oral squamous cell carcinoma	2.69E-04	50
Liver	Alfalfa	Negative	quantity of reactive oxygen species	2.69E-04	50

	hay				
Liver	Alfalfa hay	Negative	expression of protein	2.71E-04	88
Liver	Alfalfa hay	Negative	growth of yeast	2.72E-04	31
Liver	Alfalfa hay	Negative	cell movement of macrophages	2.72E-04	71
Liver	Alfalfa hay	Negative	transformation of fibroblast cell lines	2.86E-04	69
Liver	Alfalfa hay	Negative	growth of embryonic tissue	2.88E-04	73
Liver	Alfalfa hay	Negative	attachment of cells	2.89E-04	40
Liver	Alfalfa hay	Negative	apoptosis of liver cells	2.90E-04	45
Liver	Alfalfa hay	Negative	sarcoma	2.91E-04	105
Liver	Alfalfa hay	Negative	generation of T lymphocytes	2.96E-04	35
Liver	Alfalfa hay	Negative	hyperesthesia	2.96E-04	35
Liver	Alfalfa hay	Negative	behavior	2.96E-04	233
Liver	Alfalfa hay	Negative	apoptosis of B-lymphocyte derived cell lines	2.96E-04	37
Liver	Alfalfa hay	Negative	proliferation of bone marrow cells	2.96E-04	36
Liver	Alfalfa hay	Negative	differentiation of T lymphocytes	2.96E-04	97
Liver	Alfalfa hay	Negative	development of macrophages	3.01E-04	16
Liver	Alfalfa hay	Negative	generation of blood cells	3.03E-04	47
Liver	Alfalfa hay	Negative	long-term potentiation of synapse	3.05E-04	43
Liver	Alfalfa hay	Negative	abnormal morphology of nervous system	3.11E-04	175
Liver	Alfalfa hay	Negative	benign neoplasia	3.13E-04	219
Liver	Alfalfa hay	Negative	cell movement of smooth muscle cells	3.13E-04	46
Liver	Alfalfa hay	Negative	recurrent cancer	3.13E-04	46
Liver	Alfalfa	Negative	formation of muscle	3.14E-04	101

	hay				
Liver	Alfalfa hay	Negative	cell death of gonadal cell lines	3.26E-04	25
Liver	Alfalfa hay	Negative	hyperplasia of blood cells	3.26E-04	25
Liver	Alfalfa hay	Negative	proliferation of fibroblast cell lines	3.26E-04	107
Liver	Alfalfa hay	Negative	transactivation	3.39E-04	142
Liver	Alfalfa hay	Negative	cell death of B-lymphocyte derived cell lines	3.40E-04	38
Liver	Alfalfa hay	Negative	quantity of bone	3.45E-04	66
Liver	Alfalfa hay	Negative	B-cell leukemia	3.46E-04	35
Liver	Alfalfa hay	Negative	cell death of liver cells	3.47E-04	54
Liver	Alfalfa hay	Negative	proliferation of endothelial cells	3.50E-04	81
Liver	Alfalfa hay	Negative	generation of leukocytes	3.57E-04	46
Liver	Alfalfa hay	Negative	refractory malignant tumor	3.58E-04	29
Liver	Alfalfa hay	Negative	synthesis of protein	3.59E-04	112
Liver	Alfalfa hay	Negative	colon carcinoma	3.66E-04	758
Liver	Alfalfa hay	Negative	synthesis of carbohydrate	3.77E-04	107
Liver	Alfalfa hay	Negative	advanced malignant tumor	3.79E-04	201
Liver	Alfalfa hay	Negative	autophagy of cells	3.83E-04	70
Liver	Alfalfa hay	Negative	hypoplasia of thorax	3.87E-04	52
Liver	Alfalfa hay	Negative	morphology of lung	3.87E-04	52
Liver	Alfalfa hay	Negative	aggregation of cells	3.90E-04	84
Liver	Alfalfa hay	Negative	metabolism of nucleotide	3.92E-04	110
Liver	Alfalfa hay	Negative	quantity of double-positive thymocyte	4.06E-04	34
Liver	Alfalfa	Negative	quantity of muscle cells	4.06E-04	35

	hay				
Liver	Alfalfa hay	Negative	morphology of blood vessel	4.15E-04	74
Liver	Alfalfa hay	Negative	gonadal tumor	4.23E-04	195
Liver	Alfalfa hay	Negative	function of muscle	4.34E-04	89
Liver	Alfalfa hay	Negative	generation of reactive oxygen species	4.34E-04	56
Liver	Alfalfa hay	Negative	cell death of sarcoma cell lines	4.35E-04	61
Liver	Alfalfa hay	Negative	apoptosis of retinal cells	4.36E-04	18
Liver	Alfalfa hay	Negative	function of heart	4.52E-04	62
Liver	Alfalfa hay	Negative	quantity of muscle	4.62E-04	38
Liver	Alfalfa hay	Negative	quantity of macrophages	4.62E-04	55
Liver	Alfalfa hay	Negative	morphology of lymphocytes	4.63E-04	53
Liver	Alfalfa hay	Negative	abnormal morphology of heart	4.63E-04	97
Liver	Alfalfa hay	Negative	ulcerative colitis	4.78E-04	33
Liver	Alfalfa hay	Negative	secretion of molecule	4.91E-04	135
Liver	Alfalfa hay	Negative	migration of fibroblasts	4.94E-04	41
Liver	Alfalfa hay	Negative	infection by HIV-1	5.10E-04	148
Liver	Alfalfa hay	Negative	abnormal morphology of spleen	5.11E-04	70
Liver	Alfalfa hay	Negative	abnormal morphology of head	5.42E-04	197
Liver	Alfalfa hay	Negative	abnormal morphology of mononuclear leukocytes	5.48E-04	48
Liver	Alfalfa hay	Negative	hypoplasia of organ	5.57E-04	111
Liver	Alfalfa hay	Negative	exocytosis	5.58E-04	52
Liver	Alfalfa hay	Negative	biosynthesis of purine ribonucleotide	5.60E-04	31
Liver	Alfalfa	Negative	peripheral arterial disease	5.73E-04	57

	hay				
Liver	Alfalfa hay	Negative	quantity of bone marrow cells	5.76E-04	47
Liver	Alfalfa hay	Negative	activation of blood cells	6.00E-04	178
Liver	Alfalfa hay	Negative	transport of metal	6.02E-04	85
Liver	Alfalfa hay	Negative	cell death of carcinoma cell lines	6.03E-04	74
Liver	Alfalfa hay	Negative	growth of lesion	6.07E-04	189
Liver	Alfalfa hay	Negative	development of body axis	6.13E-04	244
Liver	Alfalfa hay	Negative	Dermatitis	6.15E-04	108
Liver	Alfalfa hay	Negative	abnormal morphology of rib cage	6.16E-04	28
Liver	Alfalfa hay	Negative	morphology of head	6.16E-04	206
Liver	Alfalfa hay	Negative	response of mononuclear leukocytes	6.16E-04	62
Liver	Alfalfa hay	Negative	colon adenocarcinoma	6.18E-04	741
Liver	Alfalfa hay	Negative	abnormal morphology of lung	6.18E-04	50
Liver	Alfalfa hay	Negative	fibrosis of heart	6.18E-04	50
Liver	Alfalfa hay	Negative	degeneration of central nervous system	6.18E-04	38
Liver	Alfalfa hay	Negative	cell death of brain cells	6.21E-04	71
Liver	Alfalfa hay	Negative	proliferation of tumor cells	6.26E-04	116
Liver	Alfalfa hay	Negative	cellular infiltration by granulocytes	6.26E-04	57
Liver	Alfalfa hay	Negative	formation of hematopoietic progenitor cells	6.30E-04	37
Liver	Alfalfa hay	Negative	metabolism of nucleic acid component or derivative	6.33E-04	127
Liver	Alfalfa hay	Negative	inflammation of intestine	6.36E-04	73
Liver	Alfalfa hay	Negative	muscular hypertrophy	6.36E-04	55
Liver	Alfalfa	Negative	abnormal morphology of lymphocytes	6.36E-04	47

	hay				
Liver	Alfalfa hay	Negative	abnormal morphology of rib	6.37E-04	23
Liver	Alfalfa hay	Negative	cartilage development	6.40E-04	41
Liver	Alfalfa hay	Negative	apoptosis of leukocytes	6.44E-04	113
Liver	Alfalfa hay	Negative	export of molecule	6.55E-04	76
Liver	Alfalfa hay	Negative	development of sensory organ	6.62E-04	137
Liver	Alfalfa hay	Negative	central nervous system tumor	6.64E-04	214
Liver	Alfalfa hay	Negative	aggregation of blood platelets	6.82E-04	52
Liver	Alfalfa hay	Negative	influx of inorganic cation	6.82E-04	52
Liver	Alfalfa hay	Negative	concentration of phospholipid	6.83E-04	50
Liver	Alfalfa hay	Negative	Organ Degeneration	6.94E-04	121
Liver	Alfalfa hay	Negative	morphology of rib	7.02E-04	24
Liver	Alfalfa hay	Negative	fatty acid metabolism	7.02E-04	145
Liver	Alfalfa hay	Negative	apoptosis of hepatocytes	7.02E-04	38
Liver	Alfalfa hay	Negative	cell death of brain	7.02E-04	75
Liver	Alfalfa hay	Negative	abnormal morphology of blood vessel	7.07E-04	69
Liver	Alfalfa hay	Negative	dilation of heart ventricle	7.28E-04	19
Liver	Alfalfa hay	Negative	growth of tumor	7.31E-04	188
Liver	Alfalfa hay	Negative	head and neck carcinoma	7.36E-04	117
Liver	Alfalfa hay	Negative	cell viability of blood cells	7.62E-04	74
Liver	Alfalfa hay	Negative	cell death of gonadal cells	7.67E-04	50
Liver	Alfalfa hay	Negative	cytolysis	7.94E-04	70
Liver	Alfalfa	Negative	cell death of leukemia cell lines	8.00E-04	81

	hay				
Liver	Alfalfa hay	Negative	cell movement of antigen presenting cells	8.00E-04	88
Liver	Alfalfa hay	Negative	granulation tissue	8.04E-04	15
Liver	Alfalfa hay	Negative	morphology of mononuclear leukocytes	8.44E-04	54
Liver	Alfalfa hay	Negative	morphology of cardiovascular tissue	8.48E-04	27
Liver	Alfalfa hay	Negative	synthesis of nucleotide	8.58E-04	91
Liver	Alfalfa hay	Negative	development of bone marrow cells	8.80E-04	28
Liver	Alfalfa hay	Negative	thrombosis of vein	8.80E-04	20
Liver	Alfalfa hay	Negative	keratosis	8.82E-04	35
Liver	Alfalfa hay	Negative	apoptosis of T lymphocytes	8.90E-04	72
Liver	Alfalfa hay	Negative	morphology of secondary lymphoid organ	8.90E-04	72
Liver	Alfalfa hay	Negative	secondary neoplasm of digestive system	8.90E-04	66
Liver	Alfalfa hay	Negative	formation of eye	8.97E-04	108
Liver	Alfalfa hay	Negative	hyperplasia of leukocytes	8.97E-04	22
Liver	Alfalfa hay	Negative	colony formation of cells	9.05E-04	120
Liver	Alfalfa hay	Negative	apoptosis of heart cells	9.05E-04	51
Liver	Alfalfa hay	Negative	expansion of cells	9.24E-04	77
Liver	Alfalfa hay	Negative	anemia	9.31E-04	87
Liver	Alfalfa hay	Negative	apoptosis of lymphocytes	9.31E-04	87
Liver	Alfalfa hay	Negative	apoptosis of eye cells	9.52E-04	19
Liver	Alfalfa hay	Negative	proliferation of epithelial cells	9.63E-04	120
Liver	Alfalfa hay	Negative	neurotransmission	9.63E-04	102
Liver	Alfalfa	Negative	proliferation of dermal cells	9.65E-04	48

	hay				
Liver	Alfalfa hay	Negative	necrosis of liver	9.83E-04	62
Liver	Alfalfa hay	Negative	growth of plasma membrane projections	9.98E-04	114
Liver	Alfalfa hay	Negative	cell death of kidney cell lines	1.00E-03	77
Liver	Alfalfa hay	Negative	metastasis	1.02E-03	179
Liver	Alfalfa hay	Negative	cell death of cerebral cortex cells	1.03E-03	60
Liver	Alfalfa hay	Negative	Budd-Chiari syndrome	1.03E-03	11
Liver	Alfalfa hay	Negative	apoptosis of neurons	1.03E-03	107
Liver	Alfalfa hay	Negative	growth of thymus gland	1.06E-03	29
Liver	Alfalfa hay	Negative	congenital malformation of skeleton	1.08E-03	104
Liver	Alfalfa hay	Negative	proliferation of epidermal cells	1.08E-03	46
Liver	Alfalfa hay	Negative	apoptosis of fibrosarcoma cell lines	1.12E-03	20
Liver	Alfalfa hay	Negative	graft-vs-host disease	1.12E-03	16
Liver	Alfalfa hay	Negative	I-kappaB kinase/NF-kappaB cascade	1.16E-03	47
Liver	Alfalfa hay	Negative	apoptosis of mononuclear leukocytes	1.17E-03	89
Liver	Alfalfa hay	Negative	apoptosis of neuroblastoma cell lines	1.19E-03	35
Liver	Alfalfa hay	Negative	invasion of tissue	1.19E-03	52
Liver	Alfalfa hay	Negative	dephosphorylation of protein	1.22E-03	50
Liver	Alfalfa hay	Negative	size of animal	1.23E-03	48
Liver	Alfalfa hay	Negative	vascularization of body region	1.25E-03	38
Liver	Alfalfa hay	Negative	migration of smooth muscle cells	1.25E-03	41
Liver	Alfalfa hay	Negative	cell death of central nervous system cells	1.28E-03	74
Liver	Alfalfa	Negative	function of lymphocytes	1.28E-03	85

	hay				
Liver	Alfalfa hay	Negative	apoptosis of fibroblasts	1.28E-03	57
Liver	Alfalfa hay	Negative	outgrowth of cells	1.29E-03	106
Liver	Alfalfa hay	Negative	adult leukemia	1.30E-03	12
Liver	Alfalfa hay	Negative	apoptosis of osteoclasts	1.30E-03	12
Liver	Rice straw	Positive	proliferation of cells	2.10E-29	1198
Liver	Rice straw	Positive	cell death	7.03E-28	1091
Liver	Rice straw	Positive	malignant solid tumor	5.07E-25	2994
Liver	Rice straw	Positive	cancer	2.48E-24	3023
Liver	Rice straw	Positive	necrosis	1.31E-22	850
Liver	Rice straw	Positive	organization of cytoplasm	6.17E-22	546
Liver	Rice straw	Positive	transport of molecule	1.39E-21	568
Liver	Rice straw	Positive	cell movement	3.91E-21	716
Liver	Rice straw	Positive	morphology of cells	4.18E-21	663
Liver	Rice straw	Positive	apoptosis	7.30E-21	860
Liver	Rice straw	Positive	organismal death	7.28E-20	785
Liver	Rice straw	Positive	morbidity or mortality	1.87E-19	792
Liver	Rice straw	Positive	cell survival	3.50E-18	488
Liver	Rice straw	Positive	organization of cytoskeleton	7.97E-18	487
Liver	Rice straw	Positive	cell viability	1.22E-17	458
Liver	Rice straw	Positive	migration of cells	4.82E-17	631
Liver	Rice straw	Positive	cellular homeostasis	8.29E-16	515
Liver	Rice	Positive	differentiation of cells	2.09E-15	721

	straw				
Liver	Rice straw	Positive	neoplasia of epithelial tissue	3.13E-14	2467
Liver	Rice straw	Positive	tumorigenesis of tissue	5.42E-14	2503
Liver	Rice straw	Positive	microtubule dynamics	1.16E-13	406
Liver	Rice straw	Positive	expression of RNA	1.54E-13	681
Liver	Rice straw	Positive	epithelial cancer	2.13E-13	2439
Liver	Rice straw	Positive	transcription	3.87E-13	636
Liver	Rice straw	Positive	transcription of RNA	4.23E-13	597
Liver	Rice straw	Positive	quantity of cells	5.54E-13	571
Liver	Rice straw	Positive	abdominal neoplasm	3.43E-12	2434
Liver	Rice straw	Positive	development of cytoplasm	5.01E-12	177
Liver	Rice straw	Positive	angiogenesis	5.01E-12	316
Liver	Rice straw	Positive	vasculogenesis	5.01E-12	264
Liver	Rice straw	Positive	concentration of lipid	7.87E-12	286
Liver	Rice straw	Positive	abdominal cancer	2.88E-11	2400
Liver	Rice straw	Positive	formation of cytoskeleton	4.39E-11	146
Liver	Rice straw	Positive	transcription of DNA	4.75E-11	494
Liver	Rice straw	Positive	organization of organelle	6.36E-11	198
Liver	Rice straw	Positive	cell movement of tumor cell lines	1.33E-10	293
Liver	Rice straw	Positive	invasion of cells	1.65E-10	298
Liver	Rice straw	Positive	Movement Disorders	1.65E-10	354
Liver	Rice straw	Positive	proliferation of tumor cell lines	1.86E-10	494
Liver	Rice	Positive	quantity of leukocytes	2.35E-10	311

	straw				
Liver	Rice straw	Positive	cell death of tumor cell lines	3.54E-10	486
Liver	Rice straw	Positive	formation of filaments	3.73E-10	150
Liver	Rice straw	Positive	fibrogenesis	4.89E-10	154
Liver	Rice straw	Positive	quantity of blood cells	5.48E-10	342
Liver	Rice straw	Positive	cell viability of tumor cell lines	1.67E-09	266
Liver	Rice straw	Positive	neuronal cell death	2.13E-09	236
Liver	Rice straw	Positive	synthesis of lipid	2.13E-09	246
Liver	Rice straw	Positive	size of body	2.13E-09	287
Liver	Rice straw	Positive	protein kinase cascade	2.15E-09	155
Liver	Rice straw	Positive	transactivation	2.32E-09	204
Liver	Rice straw	Positive	abnormal morphology of cells	2.32E-09	407
Liver	Rice straw	Positive	formation of cellular protrusions	2.37E-09	302
Liver	Rice straw	Positive	dyskinesia	6.94E-09	214
Liver	Rice straw	Positive	growth of connective tissue	8.29E-09	222
Liver	Rice straw	Positive	cell movement of myeloid cells	8.29E-09	190
Liver	Rice straw	Positive	disorder of basal ganglia	8.29E-09	259
Liver	Rice straw	Positive	neurological signs	8.61E-09	224
Liver	Rice straw	Positive	migration of blood cells	1.10E-08	290
Liver	Rice straw	Positive	cell movement of phagocytes	1.29E-08	192
Liver	Rice straw	Positive	leukocyte migration	1.41E-08	289
Liver	Rice straw	Positive	quantity of carbohydrate	1.55E-08	182
Liver	Rice	Positive	neuromuscular disease	1.55E-08	292

	straw				
Liver	Rice straw	Positive	proliferation of connective tissue cells	1.55E-08	205
Liver	Rice straw	Positive	homing of cells	1.72E-08	194
Liver	Rice straw	Positive	Viral Infection	1.89E-08	499
Liver	Rice straw	Positive	morphology of body cavity	2.29E-08	381
Liver	Rice straw	Positive	transactivation of RNA	2.44E-08	189
Liver	Rice straw	Positive	concentration of fatty acid	2.44E-08	106
Liver	Rice straw	Positive	formation of actin filaments	2.54E-08	114
Liver	Rice straw	Positive	digestive organ tumor	3.02E-08	2084
Liver	Rice straw	Positive	development of cardiovascular tissue	3.94E-08	134
Liver	Rice straw	Positive	chemotaxis of cells	4.15E-08	183
Liver	Rice straw	Positive	homing	4.77E-08	197
Liver	Rice straw	Positive	cell movement of leukocytes	4.88E-08	255
Liver	Rice straw	Positive	digestive system cancer	5.27E-08	2063
Liver	Rice straw	Positive	Organ Degeneration	5.44E-08	172
Liver	Rice straw	Positive	formation of actin stress fibers	6.05E-08	92
Liver	Rice straw	Positive	phosphorylation of protein	6.36E-08	245
Liver	Rice straw	Positive	cell death of connective tissue cells	6.83E-08	205
Liver	Rice straw	Positive	apoptosis of tumor cell lines	8.24E-08	382
Liver	Rice straw	Positive	chemotaxis	8.48E-08	187
Liver	Rice straw	Positive	Huntington's Disease	8.49E-08	196
Liver	Rice straw	Positive	metabolism of carbohydrate	8.71E-08	210
Liver	Rice	Positive	abnormal morphology of body cavity	9.59E-08	362

	straw				
Liver	Rice straw	Positive	migration of tumor cell lines	1.21E-07	235
Liver	Rice straw	Positive	development of endothelial tissue	1.21E-07	131
Liver	Rice straw	Positive	development of body trunk	1.30E-07	354
Liver	Rice straw	Positive	endothelial cell development	1.31E-07	127
Liver	Rice straw	Positive	invasion of tumor cell lines	1.62E-07	222
Liver	Rice straw	Positive	morphology of cardiovascular system	1.88E-07	208
Liver	Rice straw	Positive	engulfment of cells	1.97E-07	143
Liver	Rice straw	Positive	inflammatory response	2.21E-07	255
Liver	Rice straw	Positive	seizure disorder	2.45E-07	162
Liver	Rice straw	Positive	development of epithelial tissue	2.91E-07	176
Liver	Rice straw	Positive	endocytosis	2.97E-07	123
Liver	Rice straw	Positive	secretory pathway	3.58E-07	76
Liver	Rice straw	Positive	cell cycle progression	4.38E-07	331
Liver	Rice straw	Positive	abnormal morphology of thoracic cavity	5.22E-07	196
Liver	Rice straw	Positive	exocytosis	7.02E-07	73
Liver	Rice straw	Positive	uptake of monosaccharide	7.15E-07	98
Liver	Rice straw	Positive	development of vasculature	8.95E-07	144
Liver	Rice straw	Positive	seizures	9.16E-07	138
Liver	Rice straw	Positive	quantity of mononuclear leukocytes	9.17E-07	239
Liver	Rice straw	Positive	activation of DNA endogenous promoter	9.76E-07	375
Liver	Rice straw	Positive	degeneration of nervous system	1.06E-06	103
Liver	Rice	Positive	growth of organism	1.09E-06	268

	straw				
Liver	Rice straw	Positive	development of connective tissue	1.09E-06	129
Liver	Rice straw	Positive	necrosis of epithelial tissue	1.12E-06	195
Liver	Rice straw	Positive	behavior	1.14E-06	312
Liver	Rice straw	Positive	long-term potentiation	1.43E-06	89
Liver	Rice straw	Positive	proliferation of blood cells	1.51E-06	269
Liver	Rice straw	Positive	ion homeostasis of cells	2.15E-06	184
Liver	Rice straw	Positive	invasion of carcinoma cell lines	2.28E-06	74
Liver	Rice straw	Positive	uptake of carbohydrate	2.28E-06	103
Liver	Rice straw	Positive	learning	2.34E-06	142
Liver	Rice straw	Positive	cognition	2.40E-06	153
Liver	Rice straw	Positive	function of leukocytes	2.40E-06	178
Liver	Rice straw	Positive	perinatal death	2.45E-06	195
Liver	Rice straw	Positive	interphase	2.84E-06	213
Liver	Rice straw	Positive	quantity of lymphocytes	2.90E-06	228
Liver	Rice straw	Positive	cell movement of fibroblasts	2.90E-06	69
Liver	Rice straw	Positive	hypersensitive reaction	2.95E-06	145
Liver	Rice straw	Positive	formation of plasma membrane projections	3.23E-06	195
Liver	Rice straw	Positive	neuritogenesis	3.24E-06	191
Liver	Rice straw	Positive	cell movement of connective tissue cells	3.76E-06	81
Liver	Rice straw	Positive	cell death of blood cells	4.18E-06	223
Liver	Rice straw	Positive	activation of cells	4.50E-06	305
Liver	Rice	Positive	quantity of metal	4.50E-06	165

	straw				
Liver	Rice straw	Positive	morphology of heart	4.55E-06	137
Liver	Rice straw	Positive	organization of actin cytoskeleton	4.65E-06	105
Liver	Rice straw	Positive	proliferation of neuronal cells	4.86E-06	188
Liver	Rice straw	Positive	reorganization of cytoskeleton	4.91E-06	71
Liver	Rice straw	Positive	chemotaxis of phagocytes	5.17E-06	103
Liver	Rice straw	Positive	degeneration of cells	5.30E-06	118
Liver	Rice straw	Positive	proliferation of immune cells	5.48E-06	251
Liver	Rice straw	Positive	cell death of epithelial cells	5.78E-06	164
Liver	Rice straw	Positive	abnormal morphology of cardiovascular system	5.96E-06	185
Liver	Rice straw	Positive	cell movement of carcinoma cell lines	6.29E-06	75
Liver	Rice straw	Positive	Growth Failure	6.37E-06	194
Liver	Rice straw	Positive	cell death of immune cells	6.37E-06	212
Liver	Rice straw	Positive	differentiation of connective tissue cells	6.45E-06	207
Liver	Rice straw	Positive	uterine serous papillary cancer	6.45E-06	83
Liver	Rice straw	Positive	synthesis of carbohydrate	6.81E-06	142
Liver	Rice straw	Positive	migration of connective tissue cells	7.52E-06	67
Liver	Rice straw	Positive	function of blood cells	7.72E-06	190
Liver	Rice straw	Positive	colony formation	8.31E-06	175
Liver	Rice straw	Positive	cell movement of granulocytes	8.76E-06	125
Liver	Rice straw	Positive	proliferation of endothelial cells	8.99E-06	107
Liver	Rice straw	Positive	cell death of fibroblast cell lines	9.48E-06	142
Liver	Rice	Positive	chemotaxis of myeloid cells	9.80E-06	101

	straw				
Liver	Rice straw	Positive	differentiation of connective tissue	1.14E-05	231
Liver	Rice straw	Positive	colony formation of cells	1.16E-05	161
Liver	Rice straw	Positive	metabolism of nucleotide	1.18E-05	145
Liver	Rice straw	Positive	morphology of head	1.18E-05	273
Liver	Rice straw	Positive	influx of Ca2+	1.28E-05	68
Liver	Rice straw	Positive	cell movement of neutrophils	1.40E-05	102
Liver	Rice straw	Positive	influx of cation	1.46E-05	70
Liver	Rice straw	Positive	long-term potentiation of synapse	1.49E-05	56
Liver	Rice straw	Positive	plasticity of synapse	1.53E-05	47
Liver	Rice straw	Positive	dephosphorylation of protein	1.53E-05	68
Liver	Rice straw	Positive	quantity of connective tissue	1.64E-05	193
Liver	Rice straw	Positive	cell movement of macrophages	1.90E-05	92
Liver	Rice straw	Positive	fatty acid metabolism	1.93E-05	192
Liver	Rice straw	Positive	Neurodegeneration	1.95E-05	105
Liver	Rice straw	Positive	proliferation of mononuclear leukocytes	1.95E-05	232
Liver	Rice straw	Positive	accumulation of cells	1.97E-05	118
Liver	Rice straw	Positive	female genital tract serous cancer	1.99E-05	128
Liver	Rice straw	Positive	growth of lesion	2.03E-05	249
Liver	Rice straw	Positive	cell movement of antigen presenting cells	2.08E-05	117
Liver	Rice straw	Positive	influx of inorganic cation	2.08E-05	69
Liver	Rice straw	Positive	quantity of metal ion	2.15E-05	145
Liver	Rice	Positive	branching of cells	2.20E-05	123

	straw				
Liver	Rice straw	Positive	synthesis of nucleotide	2.24E-05	121
Liver	Rice straw	Positive	secretion of molecule	2.31E-05	177
Liver	Rice straw	Positive	binding of DNA	2.32E-05	168
Liver	Rice straw	Positive	formation of skin	2.32E-05	122
Liver	Rice straw	Positive	activation of antigen presenting cells	2.41E-05	107
Liver	Rice straw	Positive	growth of tumor	2.45E-05	248
Liver	Rice straw	Positive	proliferation of lymphocytes	2.49E-05	228
Liver	Rice straw	Positive	Dermatitis	2.60E-05	142
Liver	Rice straw	Positive	metabolism of membrane lipid derivative	2.67E-05	128
Liver	Rice straw	Positive	killing of macrophages	2.76E-05	12
Liver	Rice straw	Positive	concentration of acylglycerol	2.81E-05	112
Liver	Rice straw	Positive	growth of epithelial tissue	2.87E-05	214
Liver	Rice straw	Positive	allergy	2.87E-05	133
Liver	Rice straw	Positive	flux of ion	3.25E-05	108
Liver	Rice straw	Positive	transport of carbohydrate	3.39E-05	69
Liver	Rice straw	Positive	morphology of lymphatic system component	3.58E-05	136
Liver	Rice straw	Positive	demyelination	3.64E-05	46
Liver	Rice straw	Positive	differentiation of muscle cell lines	3.75E-05	59
Liver	Rice straw	Positive	abnormal morphology of heart	3.80E-05	126
Liver	Rice straw	Positive	migration of fibroblasts	3.97E-05	53
Liver	Rice straw	Positive	cell movement of endothelial cells	4.04E-05	117
Liver	Rice	Positive	quantity of Ca2+	4.42E-05	133

	straw				
Liver	Rice straw	Positive	autophagy	4.71E-05	123
Liver	Rice straw	Positive	formation of cells	4.81E-05	299
Liver	Rice straw	Positive	organization of filaments	4.86E-05	72
Liver	Rice straw	Positive	growth of lymphatic system component	4.86E-05	68
Liver	Rice straw	Positive	morphology of heart ventricle	4.86E-05	68
Liver	Rice straw	Positive	transport of protein	5.06E-05	101
Liver	Rice straw	Positive	morphology of muscle	5.38E-05	111
Liver	Rice straw	Positive	proliferation of smooth muscle cells	5.38E-05	96
Liver	Rice straw	Positive	permeability of cells	5.41E-05	39
Liver	Rice straw	Positive	abnormal morphology of head	5.54E-05	256
Liver	Rice straw	Positive	quantity of myeloid cells	5.59E-05	117
Liver	Rice straw	Positive	dysmyelination	5.92E-05	50
Liver	Rice straw	Positive	flux of cation	6.01E-05	102
Liver	Rice straw	Positive	accumulation of blood cells	6.02E-05	94
Liver	Rice straw	Positive	growth of muscle tissue	6.09E-05	122
Liver	Rice straw	Positive	synthesis of DNA	6.21E-05	135
Liver	Rice straw	Positive	metabolism of nucleic acid component or derivative	6.24E-05	165
Liver	Rice straw	Positive	transport of alpha-amino acid	6.36E-05	30
Liver	Rice straw	Positive	abnormal morphology of abdomen	6.44E-05	268
Liver	Rice straw	Positive	cell movement of muscle cells	6.46E-05	64
Liver	Rice straw	Positive	quantity of phagocytes	6.48E-05	137
Liver	Rice	Positive	ubiquitination	6.53E-05	111

	straw				
Liver	Rice straw	Positive	development of neurons	6.70E-05	241
Liver	Rice straw	Positive	quantity of lymphatic system component	6.88E-05	123
Liver	Rice straw	Positive	activation of leukocytes	6.96E-05	217
Liver	Rice straw	Positive	hydrolysis of lipid	6.99E-05	66
Liver	Rice straw	Positive	concentration of phosphatidic acid	6.99E-05	50
Liver	Rice straw	Positive	proliferation of muscle cells	7.01E-05	121
Liver	Rice straw	Positive	cellular infiltration by macrophages	7.78E-05	57
Liver	Rice straw	Positive	transport of monosaccharide	7.78E-05	57
Liver	Rice straw	Positive	cell death of brain	7.97E-05	97
Liver	Rice straw	Positive	flux of Ca2+	7.97E-05	95
Liver	Rice straw	Positive	benign neoplasia	8.18E-05	281
Liver	Rice straw	Positive	ubiquitination of protein	8.22E-05	109
Liver	Rice straw	Positive	receptor-mediated endocytosis	8.26E-05	53
Liver	Rice straw	Positive	flux of inorganic cation	8.26E-05	100
Liver	Rice straw	Positive	homing of blood cells	8.26E-05	123
Liver	Rice straw	Positive	export of molecule	8.27E-05	98
Liver	Rice straw	Positive	neurotransmission	8.27E-05	133
Liver	Rice straw	Positive	release of fatty acid	8.31E-05	60
Liver	Rice straw	Positive	transport of cation	8.31E-05	130
Liver	Rice straw	Positive	sprouting	8.73E-05	125
Liver	Rice straw	Positive	adhesion of immune cells	8.91E-05	114
Liver	Rice	Positive	development of body axis	9.12E-05	316

	straw				
Liver	Rice straw	Positive	degeneration of neurons	9.30E-05	81
Liver	Rice straw	Positive	cell death of epithelial cell lines	9.30E-05	94
Liver	Rice straw	Positive	experimentally-induced diabetes	9.55E-05	37
Liver	Rice straw	Positive	cell death of hippocampal neurons	9.91E-05	34
Liver	Rice straw	Positive	concentration of phospholipid	1.01E-04	64
Liver	Rice straw	Positive	metastasis of cells	1.02E-04	84
Liver	Rice straw	Positive	morphology of nervous system	1.03E-04	240
Liver	Rice straw	Positive	cellular infiltration by leukocytes	1.06E-04	125
Liver	Rice straw	Positive	transmigration of cells	1.10E-04	56
Liver	Rice straw	Positive	cognitive impairment	1.20E-04	108
Liver	Rice straw	Positive	metabolism of protein	1.20E-04	293
Liver	Rice straw	Positive	epileptic seizure	1.20E-04	59
Liver	Rice straw	Positive	epilepsy	1.20E-04	101
Liver	Rice straw	Positive	release of eicosanoid	1.20E-04	51
Liver	Rice straw	Positive	cell death of hippocampal cells	1.22E-04	37
Liver	Rice straw	Positive	hydrolysis of phosphoinositide	1.23E-04	25
Liver	Rice straw	Positive	infection by RNA virus	1.23E-04	263
Liver	Rice straw	Positive	cellular degradation	1.23E-04	85
Liver	Rice straw	Positive	morphology of bone	1.23E-04	143
Liver	Rice straw	Positive	cell death of lymphocytes	1.23E-04	124
Liver	Rice straw	Positive	abnormal morphology of bone	1.23E-04	139
Liver	Rice	Positive	apoptosis of fibroblast cell lines	1.23E-04	107

	straw				
Liver	Rice straw	Positive	migration of endothelial cells	1.23E-04	107
Liver	Rice straw	Positive	activation of blood cells	1.23E-04	229
Liver	Rice straw	Positive	uptake of D-glucose	1.23E-04	75
Liver	Rice straw	Positive	cell death of heart	1.28E-04	77
Liver	Rice straw	Positive	cell death of T lymphocytes	1.28E-04	103
Liver	Rice straw	Positive	transport of ion	1.33E-04	159
Liver	Rice straw	Positive	cancer of cells	1.37E-04	85
Liver	Rice straw	Positive	apoptosis of blood cells	1.38E-04	156
Liver	Rice straw	Positive	migration of muscle cells	1.41E-04	58
Liver	Rice straw	Positive	differentiation of bone cells	1.42E-04	130
Liver	Rice straw	Positive	cell death of central nervous system cells	1.43E-04	96
Liver	Rice straw	Positive	replication of RNA virus	1.43E-04	159
Liver	Rice straw	Positive	cell death of mononuclear leukocytes	1.43E-04	127
Liver	Rice straw	Positive	formation of muscle	1.43E-04	127
Liver	Rice straw	Positive	homing of leukocytes	1.47E-04	121
Liver	Rice straw	Positive	abnormal morphology of heart ventricle	1.48E-04	61
Liver	Rice straw	Positive	release of neurotransmitter	1.52E-04	55
Liver	Rice straw	Positive	transport of metal	1.56E-04	108
Liver	Rice straw	Positive	arthropathy	1.70E-04	286
Liver	Rice straw	Positive	development of head	1.70E-04	294
Liver	Rice straw	Positive	infection by Retroviridae	1.70E-04	220
Liver	Rice	Positive	HIV infection	1.70E-04	218

	straw				
Liver	Rice straw	Positive	chemotaxis of leukocytes	1.75E-04	114
Liver	Rice straw	Positive	morphology of lymphoid organ	1.81E-04	125
Liver	Rice straw	Positive	syndromic mental retardation	1.81E-04	55
Liver	Rice straw	Positive	morphology of connective tissue	1.93E-04	145
Liver	Rice straw	Positive	cell death of heart cells	1.96E-04	74
Liver	Rice straw	Positive	arthritis	1.96E-04	281
Liver	Rice straw	Positive	abnormal morphology of nervous system	2.09E-04	221
Liver	Rice straw	Positive	transport of inorganic cation	2.09E-04	113
Liver	Rice straw	Positive	S phase	2.10E-04	86
Liver	Rice straw	Positive	neutropenia	2.10E-04	19
Liver	Rice straw	Positive	apoptosis of epithelial cell lines	2.10E-04	75
Liver	Rice straw	Positive	serous neoplasm	2.10E-04	188
Liver	Rice straw	Positive	I-kappaB kinase/NF-kappaB cascade	2.10E-04	60
Liver	Rice straw	Positive	production of reactive oxygen species	2.11E-04	117
Liver	Rice straw	Positive	concentration of eicosanoid	2.11E-04	42
Liver	Rice straw	Positive	quantity of B lymphocytes	2.11E-04	111
Liver	Rice straw	Positive	concentration of Ca2+	2.11E-04	46
Liver	Rice straw	Positive	small GTPase mediated signal transduction	2.14E-04	63
Liver	Rice straw	Positive	release of arachidonic acid	2.14E-04	35
Liver	Rice straw	Positive	mitosis	2.17E-04	155
Liver	Rice straw	Positive	cell viability of cervical cancer cell lines	2.17E-04	74
Liver	Rice	Positive	synthesis of fatty acid	2.18E-04	101

	straw				
Liver	Rice straw	Positive	proliferation of fibroblasts	2.23E-04	113
Liver	Rice straw	Positive	mass of connective tissue	2.24E-04	68
Liver	Rice straw	Positive	proliferation of lymphatic system cells	2.24E-04	68
Liver	Rice straw	Positive	accumulation of lipid	2.26E-04	97
Liver	Rice straw	Positive	development of blood cells	2.28E-04	200
Liver	Rice straw	Positive	development of lymphatic system component	2.31E-04	100
Liver	Rice straw	Positive	proliferation of T lymphocytes	2.34E-04	186
Liver	Rice straw	Positive	homeostasis of ion	2.40E-04	79
Liver	Rice straw	Positive	quantity of steroid	2.43E-04	146
Liver	Rice straw	Positive	binding of endothelial cells	2.43E-04	39
Liver	Rice straw	Positive	infection of cells	2.46E-04	241
Liver	Rice straw	Positive	degeneration of central nervous system	2.47E-04	47
Liver	Rice straw	Positive	apoptosis of neurons	2.47E-04	137
Liver	Rice straw	Positive	maturation of cells	2.51E-04	143
Liver	Rice straw	Positive	growth of embryo	2.51E-04	149
Liver	Rice straw	Positive	quantity of hematopoietic cells	2.55E-04	139
Liver	Rice straw	Positive	experimentally-induced arthritis	2.57E-04	53
Liver	Rice straw	Positive	neurodegeneration of cerebral cortex	2.58E-04	24
Liver	Rice straw	Positive	proliferation of glomerular cells	2.58E-04	24
Liver	Rice straw	Positive	proliferation of mesangial cells	2.59E-04	21
Liver	Rice straw	Positive	size of animal	2.62E-04	61
Liver	Rice	Positive	cell death of brain cells	2.63E-04	89

	straw				
Liver	Rice straw	Positive	homeostasis of inorganic cation	2.65E-04	64
Liver	Rice straw	Positive	apoptosis of leukocytes	2.68E-04	143
Liver	Rice straw	Positive	synaptic transmission	2.74E-04	108
Liver	Rice straw	Positive	development of leukocytes	2.77E-04	181
Liver	Rice straw	Positive	cell death of cerebral cortex cells	2.77E-04	76
Liver	Rice straw	Positive	formation of leukocytes	2.81E-04	55
Liver	Rice straw	Positive	activation of neuroglia	2.92E-04	47
Liver	Rice straw	Positive	proliferation of fibroblast cell lines	2.93E-04	133
Liver	Rice straw	Positive	modification of peptide	2.95E-04	39
Liver	Rice straw	Positive	abnormal morphology of embryonic tissue	2.99E-04	169
Liver	Rice straw	Positive	muscle contraction	2.99E-04	81
Liver	Rice straw	Positive	quantity of bone	2.99E-04	81
Liver	Rice straw	Positive	metabolism of monosaccharide	2.99E-04	44
Liver	Rice straw	Positive	activation of phagocytes	2.99E-04	118
Liver	Rice straw	Positive	cellular infiltration	3.12E-04	137
Liver	Rice straw	Positive	neovascularization	3.16E-04	56
Liver	Rice straw	Positive	quantity of hematopoietic progenitor cells	3.16E-04	138
Liver	Rice straw	Positive	Bleeding	3.16E-04	133
Liver	Rice straw	Positive	aggregation of cells	3.19E-04	104
Liver	Rice straw	Positive	function of cardiovascular system	3.19E-04	117
Liver	Rice straw	Positive	accumulation of leukocytes	3.29E-04	87
Liver	Rice	Positive	quantity of T lymphocytes	3.35E-04	165

	straw				
Liver	Rice straw	Positive	growth of renal glomerulus	3.36E-04	28
Liver	Rice straw	Positive	cleavage of lipid	3.43E-04	67
Liver	Rice straw	Positive	autophagy of cells	3.50E-04	86
Liver	Rice straw	Positive	transport of L-amino acid	3.52E-04	26
Liver	Rice straw	Positive	excitatory postsynaptic potential	3.60E-04	44
Liver	Rice straw	Positive	transport of metal ion	3.64E-04	102
Liver	Rice straw	Positive	differentiation of muscle	3.72E-04	97
Liver	Rice straw	Positive	development of lymphocytes	3.72E-04	170
Liver	Rice straw	Positive	advanced malignant tumor	3.82E-04	253
Liver	Rice straw	Positive	chronic psoriasis	3.82E-04	50
Liver	Rice straw	Positive	concentration of sterol	3.85E-04	98
Liver	Rice straw	Positive	quantity of granulocytes	3.88E-04	101
Liver	Rice straw	Positive	intracranial hemorrhage	3.91E-04	49
Liver	Rice straw	Positive	migration of carcinoma cell lines	3.93E-04	58
Liver	Rice straw	Positive	cell transformation	3.96E-04	143
Liver	Rice straw	Positive	apoptosis of kidney cell lines	3.97E-04	78
Liver	Rice straw	Positive	development of mononuclear leukocytes	3.97E-04	171
Liver	Rice straw	Positive	entrance of Ca2+	3.97E-04	30
Liver	Rice straw	Positive	abnormal morphology of lymphoid organ	3.97E-04	119
Liver	Rice straw	Positive	replication of virus	3.97E-04	172
Liver	Rice straw	Positive	cell death of tumor	4.05E-04	120
Liver	Rice	Positive	oxidative stress response of cells	4.05E-04	25

	straw				
Liver	Rice straw	Positive	concentration of triacylglycerol	4.22E-04	98
Liver	Rice straw	Positive	proliferation of leukemia cell lines	4.30E-04	70
Liver	Rice straw	Positive	abnormal quantity of lipid	4.32E-04	51
Liver	Rice straw	Positive	homeostasis of metal ion	4.33E-04	62
Liver	Rice straw	Positive	development of abdomen	4.34E-04	177
Liver	Rice straw	Positive	neurodegeneration of brain	4.34E-04	40
Liver	Rice straw	Positive	inflammation of organ	4.40E-04	344
Liver	Rice straw	Positive	serine phosphorylation of peptide	4.48E-04	32
Liver	Rice straw	Positive	cell movement of mononuclear leukocytes	4.59E-04	143
Liver	Rice straw	Positive	growth of neurites	4.59E-04	143
Liver	Rice straw	Positive	phagocytosis	4.59E-04	95
Liver	Rice straw	Positive	vascularization	4.70E-04	77
Liver	Rice straw	Positive	consumption of oxygen	4.70E-04	53
Liver	Rice straw	Positive	concentration of cholesterol	4.70E-04	93
Liver	Rice straw	Positive	assembly of protein-protein complex	4.70E-04	63
Liver	Rice straw	Positive	morphology of respiratory system	4.78E-04	99
Liver	Rice straw	Positive	abnormal morphology of skull	4.90E-04	72
Liver	Rice straw	Positive	differentiation of epithelial tissue	4.90E-04	113
Liver	Rice straw	Positive	branching of neurites	4.90E-04	89
Liver	Rice straw	Positive	serine phosphorylation	4.96E-04	35
Liver	Rice straw	Positive	cell death of kidney cells	4.96E-04	110
Liver	Rice	Positive	growth of plasma membrane projections	4.96E-04	144

	straw				
Liver	Rice straw	Positive	cell viability of blood cells	4.96E-04	92
Liver	Rice straw	Positive	adhesion of blood cells	4.98E-04	118
Liver	Rice straw	Positive	degeneration of brain	5.01E-04	44
Liver	Rice straw	Positive	necrosis of tumor	5.07E-04	119
Liver	Rice straw	Positive	peripheral vascular disease	5.07E-04	119
Liver	Rice straw	Positive	abnormal morphology of right ventricle	5.09E-04	21
Liver	Rice straw	Positive	homeostasis of divalent cations	5.11E-04	50
Liver	Rice straw	Positive	proliferation of hematopoietic cell lines	5.15E-04	73
Liver	Rice straw	Positive	development of lymphatic system	5.15E-04	112
Liver	Rice straw	Positive	neonatal death	5.21E-04	136
Liver	Rice straw	Positive	mass of adipose tissue	5.30E-04	63
Liver	Rice straw	Positive	morphology of right ventricle	5.37E-04	23
Liver	Rice straw	Positive	phosphorylation of L-amino acid	5.45E-04	72
Liver	Rice straw	Positive	cell movement of lymphocytes	5.45E-04	123
Liver	Rice straw	Positive	mobilization of cells	5.54E-04	32
Liver	Rice straw	Positive	vascularization of body region	5.55E-04	47
Liver	Rice straw	Positive	synthesis of phospholipid	5.58E-04	59
Liver	Rice straw	Positive	homeostasis of leukocytes	5.86E-04	169
Liver	Rice straw	Positive	necrosis of kidney	5.93E-04	114
Liver	Rice straw	Positive	autophosphorylation of protein	6.06E-04	54
Liver	Rice straw	Positive	quantity of polyunsaturated fatty acids	6.07E-04	43
Liver	Rice	Positive	morphology of skin	6.11E-04	87

	straw				
Liver	Rice straw	Positive	metastasis	6.28E-04	227
Liver	Rice straw	Positive	cell death of kidney cell lines	6.35E-04	96
Liver	Rice straw	Positive	muscular hypertrophy	6.39E-04	67
Liver	Rice straw	Positive	activation of Protein kinase	6.43E-04	81
Liver	Rice straw	Positive	cellular infiltration of phagocytes	6.46E-04	59
Liver	Rice straw	Positive	branching of neurons	6.46E-04	91
Liver	Rice straw	Positive	necrosis of cardiac muscle	6.50E-04	71
Liver	Rice straw	Positive	activation of macrophages	6.50E-04	77
Liver	Rice straw	Positive	abnormal morphology of respiratory system	6.50E-04	97
Liver	Rice straw	Positive	autosomal recessive disease	6.50E-04	272
Liver	Rice straw	Positive	synthesis of purine nucleotide	6.75E-04	58
Liver	Rice straw	Positive	recruitment of leukocytes	6.92E-04	102
Liver	Rice straw	Positive	quantity of antigen presenting cells	7.08E-04	88
Liver	Rice straw	Positive	exocytosis by cells	7.09E-04	44
Liver	Rice straw	Positive	cell viability of mononuclear leukocytes	7.09E-04	65
Liver	Rice straw	Positive	outgrowth of neurites	7.27E-04	125
Liver	Rice straw	Positive	transport of D-glucose	7.27E-04	49
Liver	Rice straw	Positive	morphogenesis of neurons	7.29E-04	131
Liver	Rice straw	Positive	migration of mesenchymal cells	7.61E-04	17
Liver	Rice straw	Positive	chemotaxis of antigen presenting cells	7.61E-04	52
Liver	Rice straw	Positive	chronic myeloid leukemia	7.62E-04	38
Liver	Rice	Positive	exocytosis of granules	7.62E-04	13

	straw				
Liver	Rice straw	Positive	internalization of lipid	7.62E-04	13
Liver	Rice straw	Positive	necrosis of muscle	7.90E-04	107
Liver	Rice straw	Positive	size of embryo	7.90E-04	111
Liver	Rice straw	Positive	hypertrophy of cardiomyocytes	7.96E-04	46
Liver	Rice straw	Positive	transport of amino acids	7.96E-04	46
Liver	Rice straw	Positive	cell movement of smooth muscle cells	8.09E-04	54
Liver	Rice straw	Positive	hydrolysis of carbohydrate	8.19E-04	45
Liver	Rice straw	Positive	T cell development	8.27E-04	153
Liver	Rice straw	Positive	neoplasia of cells	8.36E-04	136
Liver	Rice straw	Positive	miniature excitatory postsynaptic currents	8.36E-04	24
Liver	Rice straw	Positive	relaxation of muscle	8.38E-04	31
Liver	Rice straw	Positive	shape change of neurites	8.40E-04	90
Liver	Rice straw	Positive	transport of synaptic vesicles	8.53E-04	27
Liver	Rice straw	Positive	activation of enzyme	8.55E-04	120
Liver	Rice straw	Positive	immediate hypersensitivity	8.61E-04	100
Liver	Rice straw	Positive	synthesis of progesterone	8.63E-04	32
Liver	Rice straw	Positive	Lymphocyte homeostasis	8.63E-04	165
Liver	Rice straw	Positive	outgrowth of cells	8.66E-04	133
Liver	Rice straw	Positive	keratosis	8.67E-04	42
Liver	Rice straw	Positive	abnormal morphology of skeleton	8.78E-04	86
Liver	Rice straw	Positive	phagocytosis of cells	8.78E-04	86
Liver	Rice	Positive	differentiation of muscle cells	8.89E-04	89

	straw				
Liver	Rice straw	Positive	binding of mononuclear leukocytes	8.94E-04	40
Liver	Rice straw	Positive	binding of lymphocytes	8.95E-04	34
Liver	Rice straw	Positive	uptake of fatty acid	9.04E-04	28
Liver	Rice straw	Positive	mental retardation	9.07E-04	75
Liver	Rice straw	Positive	metabolism of phospholipid	9.07E-04	75
Liver	Rice straw	Positive	response of mononuclear leukocytes	9.07E-04	75
Liver	Rice straw	Positive	cell death of tumor cells	9.14E-04	115
Liver	Rice straw	Positive	cellularity	9.15E-04	25
Liver	Rice straw	Positive	apoptosis of heart cells	9.26E-04	62
Liver	Rice straw	Positive	tyrosine phosphorylation of protein	9.26E-04	62
Liver	Rice straw	Positive	function of muscle	9.43E-04	108
Liver	Rice straw	Positive	transmigration of blood cells	9.78E-04	44
Liver	Rice straw	Positive	mobilization of Ca2+	1.02E-03	107
Liver	Rice straw	Positive	formation of filopodia	1.02E-03	55
Liver	Rice straw	Positive	cell death of cardiomyocytes	1.04E-03	69
Liver	Rice straw	Positive	morphology of skeleton	1.05E-03	88
Liver	Rice straw	Positive	metabolism of acylglycerol	1.05E-03	47
Liver	Rice straw	Positive	hydrolysis of phosphatidylinositol	1.09E-03	33
Liver	Rice straw	Positive	degranulation of cells	1.10E-03	72
Liver	Rice straw	Positive	contraction of heart	1.10E-03	59
Liver	Rice straw	Positive	formation of connective tissue cells	1.10E-03	68
Liver	Rice	Positive	quantity of lymphoid organ	1.11E-03	96

	straw				
Liver	Rice straw	Positive	morphogenesis of neurites	1.11E-03	128
Liver	Rice straw	Positive	quantity of cellular protrusions	1.11E-03	53
Liver	Rice straw	Positive	synthesis of nitric oxide	1.12E-03	87
Liver	Rice straw	Positive	contraction of striated muscle	1.12E-03	45
Liver	Rice straw	Positive	cell viability of leukocytes	1.13E-03	82
Liver	Rice straw	Positive	cell death of muscle cells	1.13E-03	104
Liver	Rice straw	Positive	abnormal morphology of axial skeleton	1.14E-03	63
Liver	Rice straw	Positive	trafficking of cells	1.15E-03	28
Liver	Rice straw	Positive	recruitment of cells	1.18E-03	110
Liver	Rice straw	Positive	formation of osteoclasts	1.23E-03	42
Liver	Rice straw	Positive	differentiation of leukocytes	1.23E-03	200
Liver	Rice straw	Positive	MAPKKK cascade	1.24E-03	66
Liver	Rice straw	Positive	differentiation of blood cells	1.24E-03	248
Liver	Rice straw	Positive	adhesion of tumor cell lines	1.24E-03	90
Liver	Rice straw	Positive	autosomal dominant disease	1.27E-03	190
Liver	Rice straw	Positive	glucose metabolism disorder	1.27E-03	369
Liver	Rice straw	Positive	release of granules	1.28E-03	15
Liver	Rice straw	Positive	binding of cells	1.28E-03	145
Liver	Rice straw	Positive	development of connective tissue cells	1.30E-03	71
Liver	Rice straw	Positive	quantity of macrophages	1.30E-03	65
Liver	Rice straw	Negative	proliferation of cells	1.75E-41	1567
Liver	Rice	Negative	malignant solid tumor	7.69E-39	3940

	straw				
Liver	Rice straw	Negative	cancer	7.69E-39	3982
Liver	Rice straw	Negative	cell death	3.00E-33	1398
Liver	Rice straw	Negative	organismal death	5.97E-33	1050
Liver	Rice straw	Negative	morphology of cells	2.01E-32	878
Liver	Rice straw	Negative	morbidity or mortality	2.39E-32	1059
Liver	Rice straw	Negative	cell movement	4.66E-30	937
Liver	Rice straw	Negative	necrosis	4.66E-30	1102
Liver	Rice straw	Negative	apoptosis	9.80E-28	1117
Liver	Rice straw	Negative	migration of cells	1.45E-24	828
Liver	Rice straw	Negative	organization of cytoplasm	4.35E-24	685
Liver	Rice straw	Negative	tumorigenesis of tissue	1.39E-23	3306
Liver	Rice straw	Negative	neoplasia of epithelial tissue	4.99E-23	3253
Liver	Rice straw	Negative	epithelial cancer	1.93E-22	3221
Liver	Rice straw	Negative	organization of cytoskeleton	2.67E-20	616
Liver	Rice straw	Negative	differentiation of cells	5.74E-20	934
Liver	Rice straw	Negative	transport of molecule	2.10E-19	694
Liver	Rice straw	Negative	abnormal morphology of cells	2.30E-19	562
Liver	Rice straw	Negative	abdominal neoplasm	3.27E-19	3205
Liver	Rice straw	Negative	angiogenesis	3.87E-19	421
Liver	Rice straw	Negative	cell viability	4.37E-19	574
Liver	Rice straw	Negative	cell survival	8.27E-19	608
Liver	Rice	Negative	expression of RNA	1.20E-18	888

	straw				
Liver	Rice straw	Negative	abdominal cancer	1.20E-18	3166
Liver	Rice straw	Negative	microtubule dynamics	3.77E-18	525
Liver	Rice straw	Negative	transcription	1.06E-17	827
Liver	Rice straw	Negative	quantity of cells	1.18E-17	743
Liver	Rice straw	Negative	vasculogenesis	1.78E-17	346
Liver	Rice straw	Negative	morphology of cardiovascular system	3.32E-17	297
Liver	Rice straw	Negative	cell death of connective tissue cells	1.36E-16	288
Liver	Rice straw	Negative	transcription of RNA	2.29E-16	769
Liver	Rice straw	Negative	cellular homeostasis	2.74E-16	644
Liver	Rice straw	Negative	Viral Infection	3.98E-16	677
Liver	Rice straw	Negative	cell death of tumor cell lines	8.48E-16	643
Liver	Rice straw	Negative	cell movement of tumor cell lines	6.88E-15	382
Liver	Rice straw	Negative	quantity of leukocytes	1.36E-14	406
Liver	Rice straw	Negative	abnormal morphology of cardiovascular system	2.18E-14	266
Liver	Rice straw	Negative	autosomal recessive disease	2.48E-14	407
Liver	Rice straw	Negative	digestive system cancer	6.29E-14	2729
Liver	Rice straw	Negative	growth of organism	1.02E-13	371
Liver	Rice straw	Negative	quantity of blood cells	1.04E-13	445
Liver	Rice straw	Negative	development of cytoplasm	1.15E-13	220
Liver	Rice straw	Negative	synthesis of lipid	2.87E-13	321
Liver	Rice straw	Negative	migration of tumor cell lines	4.90E-13	317
Liver	Rice	Negative	digestive organ tumor	5.15E-13	2745

	straw				
Liver	Rice straw	Negative	cell movement of blood cells	9.35E-13	382
Liver	Rice straw	Negative	migration of blood cells	1.01E-12	381
Liver	Rice straw	Negative	phosphorylation of protein	1.01E-12	327
Liver	Rice straw	Negative	formation of cellular protrusions	1.19E-12	392
Liver	Rice straw	Negative	leukocyte migration	1.21E-12	380
Liver	Rice straw	Negative	apoptosis of tumor cell lines	1.73E-12	508
Liver	Rice straw	Negative	size of body	1.84E-12	371
Liver	Rice straw	Negative	cell cycle progression	2.05E-12	446
Liver	Rice straw	Negative	cell death of fibroblast cell lines	2.59E-12	201
Liver	Rice straw	Negative	proliferation of blood cells	2.59E-12	367
Liver	Rice straw	Negative	quantity of mononuclear leukocytes	8.45E-12	323
Liver	Rice straw	Negative	cell movement of leukocytes	9.99E-12	335
Liver	Rice straw	Negative	development of blood cells	1.01E-11	290
Liver	Rice straw	Negative	concentration of lipid	1.06E-11	352
Liver	Rice straw	Negative	transcription of DNA	1.83E-11	622
Liver	Rice straw	Negative	quantity of lymphocytes	2.38E-11	310
Liver	Rice straw	Negative	Movement Disorders	2.39E-11	445
Liver	Rice straw	Negative	invasion of cells	2.41E-11	373
Liver	Rice straw	Negative	abnormal morphology of thoracic cavity	2.58E-11	262
Liver	Rice straw	Negative	proliferation of tumor cell lines	3.17E-11	625
Liver	Rice straw	Negative	neuronal cell death	6.10E-11	298
Liver	Rice	Negative	engulfment of cells	6.81E-11	188

	straw				
Liver	Rice straw	Negative	infection of cells	6.94E-11	343
Liver	Rice straw	Negative	apoptosis of fibroblast cell lines	1.04E-10	155
Liver	Rice straw	Negative	proliferation of immune cells	1.04E-10	340
Liver	Rice straw	Negative	protein kinase cascade	1.17E-10	193
Liver	Rice straw	Negative	development of lymphocytes	1.23E-10	246
Liver	Rice straw	Negative	development of leukocytes	1.23E-10	260
Liver	Rice straw	Negative	necrosis of epithelial tissue	1.23E-10	260
Liver	Rice straw	Negative	formation of cytoskeleton	1.63E-10	174
Liver	Rice straw	Negative	homeostasis of leukocytes	1.69E-10	246
Liver	Rice straw	Negative	Lymphocyte homeostasis	1.73E-10	242
Liver	Rice straw	Negative	neurological signs	1.73E-10	284
Liver	Rice straw	Negative	development of vasculature	1.89E-10	191
Liver	Rice straw	Negative	morphology of connective tissue	1.91E-10	208
Liver	Rice straw	Negative	development of lymphatic system component	2.21E-10	146
Liver	Rice straw	Negative	morphology of heart	3.10E-10	185
Liver	Rice straw	Negative	morphology of body cavity	3.16E-10	489
Liver	Rice straw	Negative	transactivation	3.18E-10	254
Liver	Rice straw	Negative	infection by RNA virus	3.38E-10	366
Liver	Rice straw	Negative	formation of cells	3.43E-10	410
Liver	Rice straw	Negative	interphase	4.52E-10	284
Liver	Rice straw	Negative	T cell development	5.51E-10	223
Liver	Rice	Negative	abnormal morphology of body cavity	5.58E-10	468

	straw				
Liver	Rice straw	Negative	invasion of tumor cell lines	5.94E-10	287
Liver	Rice straw	Negative	development of lymphatic system	5.95E-10	164
Liver	Rice straw	Negative	dyskinesia	5.95E-10	268
Liver	Rice straw	Negative	disorder of basal ganglia	6.97E-10	326
Liver	Rice straw	Negative	growth of lymphatic system component	7.20E-10	96
Liver	Rice straw	Negative	proliferation of hematopoietic cells	7.34E-10	122
Liver	Rice straw	Negative	organization of organelle	7.38E-10	237
Liver	Rice straw	Negative	quantity of lymphatic system component	8.22E-10	172
Liver	Rice straw	Negative	infection by Retroviridae	9.10E-10	307
Liver	Rice straw	Negative	development of body trunk	1.00E-09	457
Liver	Rice straw	Negative	HIV infection	1.02E-09	304
Liver	Rice straw	Negative	homing	1.02E-09	250
Liver	Rice straw	Negative	proliferation of mononuclear leukocytes	1.02E-09	314
Liver	Rice straw	Negative	ubiquitination	1.12E-09	155
Liver	Rice straw	Negative	proliferation of connective tissue cells	1.31E-09	257
Liver	Rice straw	Negative	cell viability of tumor cell lines	1.35E-09	329
Liver	Rice straw	Negative	proliferation of lymphocytes	1.38E-09	309
Liver	Rice straw	Negative	homing of cells	1.38E-09	243
Liver	Rice straw	Negative	proliferation of hematopoietic progenitor cells	1.59E-09	116
Liver	Rice straw	Negative	infection by HIV-1	1.75E-09	263
Liver	Rice straw	Negative	T cell homeostasis	1.84E-09	226
Liver	Rice	Negative	colony formation	1.94E-09	234

	straw				
Liver	Rice straw	Negative	ubiquitination of protein	2.06E-09	152
Liver	Rice straw	Negative	growth of connective tissue	2.14E-09	276
Liver	Rice straw	Negative	fibrogenesis	2.15E-09	184
Liver	Rice straw	Negative	cell death of blood cells	2.15E-09	295
Liver	Rice straw	Negative	function of leukocytes	2.19E-09	234
Liver	Rice straw	Negative	colony formation of cells	2.28E-09	216
Liver	Rice straw	Negative	cell movement of myeloid cells	2.30E-09	235
Liver	Rice straw	Negative	differentiation of blood cells	2.87E-09	352
Liver	Rice straw	Negative	cell spreading	2.87E-09	129
Liver	Rice straw	Negative	differentiation of leukocytes	3.19E-09	286
Liver	Rice straw	Negative	metabolism of carbohydrate	3.32E-09	266
Liver	Rice straw	Negative	concentration of fatty acid	3.32E-09	130
Liver	Rice straw	Negative	formation of leukocytes	3.66E-09	80
Liver	Rice straw	Negative	proliferation of neuronal cells	4.12E-09	248
Liver	Rice straw	Negative	endocytosis	4.72E-09	156
Liver	Rice straw	Negative	chorea	4.72E-09	248
Liver	Rice straw	Negative	inflammatory response	4.79E-09	326
Liver	Rice straw	Negative	Huntington's Disease	5.12E-09	247
Liver	Rice straw	Negative	cell movement of phagocytes	5.25E-09	237
Liver	Rice straw	Negative	cell death of immune cells	5.25E-09	280
Liver	Rice straw	Negative	small GTPase mediated signal transduction	5.77E-09	90
Liver	Rice	Negative	abnormal morphology of heart	6.01E-09	171

	straw				
Liver	Rice straw	Negative	autosomal dominant disease	6.01E-09	271
Liver	Rice straw	Negative	abnormal morphology of epithelial tissue	6.19E-09	185
Liver	Rice straw	Negative	development of connective tissue	6.21E-09	166
Liver	Rice straw	Negative	cell death of epithelial cells	6.35E-09	216
Liver	Rice straw	Negative	survival of organism	7.35E-09	296
Liver	Rice straw	Negative	growth of epithelial tissue	7.66E-09	287
Liver	Rice straw	Negative	formation of blood cells	1.13E-08	95
Liver	Rice straw	Negative	function of blood cells	1.16E-08	250
Liver	Rice straw	Negative	perinatal death	1.16E-08	253
Liver	Rice straw	Negative	formation of lymphatic system component	1.48E-08	133
Liver	Rice straw	Negative	cell movement of neutrophils	1.57E-08	135
Liver	Rice straw	Negative	quantity of lymphoid organ	1.72E-08	139
Liver	Rice straw	Negative	formation of filaments	1.72E-08	175
Liver	Rice straw	Negative	chemotaxis	1.72E-08	233
Liver	Rice straw	Negative	transactivation of RNA	2.19E-08	232
Liver	Rice straw	Negative	synthesis of carbohydrate	2.81E-08	185
Liver	Rice straw	Negative	morphology of vessel	2.81E-08	138
Liver	Rice straw	Negative	degeneration of nervous system	2.81E-08	130
Liver	Rice straw	Negative	formation of lymphoid organ	2.83E-08	121
Liver	Rice straw	Negative	formation of actin filaments	3.01E-08	137
Liver	Rice straw	Negative	neuromuscular disease	3.01E-08	361
Liver	Rice	Negative	chemotaxis of cells	3.02E-08	225

	straw				
Liver	Rice straw	Negative	cell movement of granulocytes	3.36E-08	163
Liver	Rice straw	Negative	quantity of connective tissue	3.48E-08	254
Liver	Rice straw	Negative	fatty acid metabolism	3.80E-08	253
Liver	Rice straw	Negative	metabolism of protein	3.91E-08	394
Liver	Rice straw	Negative	Neurodegeneration	5.01E-08	138
Liver	Rice straw	Negative	cell death of epithelial cell lines	6.55E-08	127
Liver	Rice straw	Negative	breast or colorectal cancer	7.35E-08	1815
Liver	Rice straw	Negative	activation of DNA endogenous promoter	8.30E-08	479
Liver	Rice straw	Negative	formation of muscle	8.66E-08	172
Liver	Rice straw	Negative	abnormal morphology of abdomen	8.70E-08	356
Liver	Rice straw	Negative	formation of lymphocytes	9.28E-08	63
Liver	Rice straw	Negative	differentiation of mononuclear leukocytes	9.37E-08	228
Liver	Rice straw	Negative	formation of mononuclear leukocytes	9.47E-08	65
Liver	Rice straw	Negative	growth of embryo	1.05E-07	203
Liver	Rice straw	Negative	Growth Failure	1.08E-07	250
Liver	Rice straw	Negative	abnormal morphology of digestive system	1.11E-07	229
Liver	Rice straw	Negative	activation of cells	1.13E-07	393
Liver	Rice straw	Negative	morphology of digestive system	1.32E-07	239
Liver	Rice straw	Negative	formation of thymocytes	1.38E-07	54
Liver	Rice straw	Negative	cell transformation	1.44E-07	196
Liver	Rice straw	Negative	activation of blood cells	1.45E-07	306
Liver	Rice	Negative	quantity of hematopoietic cells	1.45E-07	189

	straw				
Liver	Rice straw	Negative	quantity of hematopoietic progenitor cells	1.87E-07	188
Liver	Rice straw	Negative	apoptosis of blood cells	1.90E-07	209
Liver	Rice straw	Negative	benign neoplasia	2.01E-07	372
Liver	Rice straw	Negative	synthesis of fatty acid	2.03E-07	137
Liver	Rice straw	Negative	quantity of T lymphocytes	2.24E-07	224
Liver	Rice straw	Negative	growth of lymphoid organ	2.32E-07	81
Liver	Rice straw	Negative	formation of T lymphocytes	2.36E-07	58
Liver	Rice straw	Negative	quantity of B lymphocytes	2.37E-07	150
Liver	Rice straw	Negative	cell death of cervical cancer cell lines	2.37E-07	152
Liver	Rice straw	Negative	morphology of blood vessel	2.45E-07	125
Liver	Rice straw	Negative	quantity of carbohydrate	2.75E-07	217
Liver	Rice straw	Negative	proliferation of T lymphocytes	2.85E-07	250
Liver	Rice straw	Negative	cell death of kidney cells	3.06E-07	151
Liver	Rice straw	Negative	cellular infiltration by leukocytes	3.12E-07	166
Liver	Rice straw	Negative	abnormal morphology of head	3.30E-07	336
Liver	Rice straw	Negative	morphology of head	3.52E-07	352
Liver	Rice straw	Negative	proliferation of lymphatic system cells	3.62E-07	92
Liver	Rice straw	Negative	growth of muscle tissue	3.78E-07	160
Liver	Rice straw	Negative	apoptosis of epithelial cell lines	4.10E-07	101
Liver	Rice straw	Negative	proliferation of muscle cells	4.11E-07	159
Liver	straw	Negative	cellular infiltration of cells	4.47E-07	169
Liver	Rice	Negative	differentiation of lymphocytes	4.56E-07	208

	straw				
Liver	Rice straw	Negative	cell movement of endothelial cells	4.56E-07	152
Liver	Rice straw	Negative	cell death of sarcoma cell lines	4.73E-07	102
Liver	Rice straw	Negative	formation of actin stress fibers	5.12E-07	107
Liver	Rice straw	Negative	apoptosis of connective tissue cells	5.48E-07	129
Liver	Rice straw	Negative	mitosis	5.67E-07	207
Liver	Rice straw	Negative	cell death of heart cells	5.79E-07	99
Liver	Rice straw	Negative	cell death of heart	5.85E-07	102
Liver	Rice straw	Negative	activation of leukocytes	6.63E-07	284
Liver	Rice straw	Negative	cell death of T lymphocytes	7.57E-07	136
Liver	Rice straw	Negative	cell death of mononuclear leukocytes	7.61E-07	168
Liver	Rice straw	Negative	development of hematopoietic system	7.61E-07	110
Liver	Rice straw	Negative	arthropathy	8.55E-07	378
Liver	Rice straw	Negative	development of cardiovascular tissue	9.20E-07	157
Liver	Rice straw	Negative	cell death of muscle	9.74E-07	147
Liver	Rice straw	Negative	generation of leukocytes	1.13E-06	75
Liver	Rice straw	Negative	binding of DNA	1.19E-06	215
Liver	Rice straw	Negative	cellular infiltration	1.19E-06	183
Liver	Rice straw	Negative	proliferation of fibroblasts	1.23E-06	150
Liver	Rice straw	Negative	necrosis of muscle	1.25E-06	146
Liver	Rice straw	Negative	morphology of heart ventricle	1.25E-06	87
Liver	Rice straw	Negative	necrosis of cardiac muscle	1.28E-06	97
Liver	Rice	Negative	synthesis of reactive oxygen species	1.29E-06	199

	straw				
Liver	Rice straw	Negative	replication of RNA virus	1.34E-06	209
Liver	Rice straw	Negative	apoptosis of kidney cell lines	1.34E-06	105
Liver	Rice straw	Negative	cell death of muscle cells	1.34E-06	143
Liver	Rice straw	Negative	generation of blood cells	1.36E-06	76
Liver	Rice straw	Negative	cognition	1.36E-06	189
Liver	Rice straw	Negative	replication of virus	1.36E-06	230
Liver	Rice straw	Negative	growth of neurites	1.36E-06	192
Liver	Rice straw	Negative	endothelial cell development	1.38E-06	150
Liver	Rice straw	Negative	growth of lesion	1.40E-06	319
Liver	Rice straw	Negative	apoptosis of leukocytes	1.40E-06	190
Liver	Rice straw	Negative	cell movement of fibroblasts	1.53E-06	83
Liver	Rice straw	Negative	hypersensitive reaction	1.61E-06	179
Liver	Rice straw	Negative	migration of connective tissue cells	1.62E-06	82
Liver	Rice straw	Negative	cell viability of blood cells	1.63E-06	124
Liver	Rice straw	Negative	quantity of phagocytes	1.65E-06	177
Liver	Rice straw	Negative	cell death of lymphocytes	1.65E-06	162
Liver	Rice straw	Negative	growth of tumor	1.66E-06	318
Liver	Rice straw	Negative	arthritis	1.66E-06	370
Liver	Rice straw	Negative	size of animal	1.68E-06	81
Liver	Rice straw	Negative	cell death of cardiomyocytes	1.73E-06	95
Liver	Rice straw	Negative	differentiation of connective tissue	1.75E-06	293
Liver	Rice	Negative	accumulation of lipid	1.95E-06	128

	straw				
Liver	Rice straw	Negative	growth of thymus gland	2.04E-06	48
Liver	Rice straw	Negative	development of endothelial tissue	2.07E-06	154
Liver	Rice straw	Negative	apoptosis of heart cells	2.13E-06	85
Liver	Rice straw	Negative	Organ Degeneration	2.13E-06	203
Liver	Rice straw	Negative	secretory pathway	2.21E-06	88
Liver	Rice straw	Negative	S phase	2.22E-06	113
Liver	Rice straw	Negative	secretion of molecule	2.25E-06	225
Liver	Rice straw	Negative	phosphorylation of L-amino acid	2.26E-06	97
Liver	Rice straw	Negative	quantity of thymus gland	2.37E-06	102
Liver	Rice straw	Negative	morphology of cardiovascular tissue	2.38E-06	44
Liver	Rice straw	Negative	apoptosis of cervical cancer cell lines	2.39E-06	121
Liver	Rice straw	Negative	migration of endothelial cells	2.44E-06	139
Liver	Rice straw	Negative	development of hematopoietic progenitor cells	2.46E-06	86
Liver	Rice straw	Negative	quantity of thymocytes	2.52E-06	101
Liver	Rice straw	Negative	degeneration of cells	2.76E-06	145
Liver	Rice straw	Negative	accumulation of cells	2.90E-06	148
Liver	Rice straw	Negative	differentiation of connective tissue cells	2.90E-06	259
Liver	Rice straw	Negative	targeting of protein	2.90E-06	61
Liver	Rice straw	Negative	cell death of kidney cell lines	2.90E-06	129
Liver	Rice straw	Negative	morphology of endothelial tissue	3.05E-06	42
Liver	Rice straw	Negative	cell movement of cancer cells	3.06E-06	68
Liver	Rice	Negative	apoptosis of epithelial cells	3.08E-06	122

	straw				
Liver	Rice straw	Negative	proliferation of endothelial cells	3.12E-06	132
Liver	Rice straw	Negative	learning	3.36E-06	173
Liver	Rice straw	Negative	abnormal morphology of blood vessel	3.47E-06	114
Liver	Rice straw	Negative	development of head	3.58E-06	384
Liver	Rice straw	Negative	transport of carbohydrate	3.83E-06	86
Liver	Rice straw	Negative	aggregation of cells	3.93E-06	137
Liver	Rice straw	Negative	formation of skin	3.95E-06	153
Liver	Rice straw	Negative	aggregation of blood platelets	4.07E-06	85
Liver	Rice straw	Negative	cell movement of connective tissue cells	4.09E-06	97
Liver	Rice straw	Negative	congenital anomaly of musculoskeletal system	4.10E-06	293
Liver	Rice straw	Negative	branching of cells	4.16E-06	154
Liver	Rice straw	Negative	modification of peptide	4.22E-06	51
Liver	Rice straw	Negative	cell death of embryonic cells	4.22E-06	55
Liver	Rice straw	Negative	necrosis of kidney	4.23E-06	152
Liver	Rice straw	Negative	cell viability of lymphocytes	4.26E-06	84
Liver	Rice straw	Negative	generation of lymphocytes	4.40E-06	65
Liver	Rice straw	Negative	peripheral vascular disease	4.49E-06	158
Liver	Rice straw	Negative	development of epithelial tissue	4.80E-06	210
Liver	Rice straw	Negative	outgrowth of neurites	4.80E-06	167
Liver	Rice straw	Negative	apoptosis of cardiomyocytes	4.82E-06	82
Liver	Rice straw	Negative	sprouting	5.01E-06	160
Liver	Rice	Negative	morphology of nervous system	5.19E-06	310

	straw				
Liver	Rice straw	Negative	colon cancer	5.25E-06	1349
Liver	Rice straw	Negative	cell viability of mononuclear leukocytes	5.37E-06	87
Liver	Rice straw	Negative	proliferation of smooth muscle cells	5.44E-06	121
Liver	Rice straw	Negative	generation of mononuclear leukocytes	5.48E-06	66
Liver	Rice straw	Negative	invasion of tissue	5.73E-06	86
Liver	Rice straw	Negative	gastrointestinal tract cancer	5.77E-06	1822
Liver	Rice straw	Negative	development of body axis	5.98E-06	408
Liver	Rice straw	Negative	neoplasia of colon	6.01E-06	1352
Liver	Rice straw	Negative	colon tumor	6.01E-06	1356
Liver	Rice straw	Negative	abdominal adenocarcinoma	6.05E-06	2116
Liver	Rice straw	Negative	behavior	6.14E-06	385
Liver	Rice straw	Negative	infection of embryonic cell lines	6.26E-06	117
Liver	Rice straw	Negative	infection of epithelial cell lines	6.26E-06	117
Liver	Rice straw	Negative	morphology of muscle	6.26E-06	140
Liver	Rice straw	Negative	concentration of phospholipid	6.26E-06	81
Liver	Rice straw	Negative	formation of thymus gland	6.32E-06	73
Liver	Rice straw	Negative	recruitment of cells	6.65E-06	148
Liver	Rice straw	Negative	angiogenesis of lesion	6.86E-06	59
Liver	Rice straw	Negative	formation of hematopoietic progenitor cells	6.86E-06	59
Liver	Rice straw	Negative	migration of tumor cells	7.02E-06	97
Liver	Rice straw	Negative	differentiation of epithelial tissue	7.02E-06	149
Liver	Rice	Negative	tumorigenesis of intestine	7.13E-06	1354

	straw				
Liver	Rice straw	Negative	adenocarcinoma	7.54E-06	2320
Liver	Rice straw	Negative	damage of epithelial tissue	7.80E-06	50
Liver	Rice straw	Negative	mass of organism	7.86E-06	139
Liver	Rice straw	Negative	chemotaxis of phagocytes	7.95E-06	124
Liver	Rice straw	Negative	I-kappaB kinase/NF-kappaB cascade	7.95E-06	77
Liver	Rice straw	Negative	catabolism of protein	8.73E-06	241
Liver	Rice straw	Negative	metabolism of reactive oxygen species	9.10E-06	202
Liver	Rice straw	Negative	cell viability of leukocytes	9.19E-06	110
Liver	Rice straw	Negative	recruitment of phagocytes	9.63E-06	105
Liver	Rice straw	Negative	cell viability of T lymphocytes	1.04E-05	54
Liver	Rice straw	Negative	function of lymphocytes	1.04E-05	141
Liver	Rice straw	Negative	proliferation of epithelial cells	1.04E-05	199
Liver	Rice straw	Negative	Bleeding	1.04E-05	173
Liver	Rice straw	Negative	exocytosis	1.04E-05	83
Liver	Rice straw	Negative	metabolism of membrane lipid derivative	1.04E-05	159
Liver	Rice straw	Negative	invasion of carcinoma cell lines	1.04E-05	86
Liver	Rice straw	Negative	female genital tract serous cancer	1.14E-05	158
Liver	Rice straw	Negative	infection of kidney cell lines	1.21E-05	119
Liver	Rice straw	Negative	apoptosis of neurons	1.23E-05	177
Liver	Rice straw	Negative	cell death of melanoma cell lines	1.27E-05	106
Liver	Rice straw	Negative	migration of muscle cells	1.28E-05	73
Liver	Rice	Negative	quantity of antigen presenting cells	1.31E-05	116

	straw				
Liver	Rice straw	Negative	outgrowth of cells	1.33E-05	176
Liver	Rice straw	Negative	cell movement of muscle cells	1.37E-05	79
Liver	Rice straw	Negative	differentiation of T lymphocytes	1.48E-05	155
Liver	Rice straw	Negative	transmembrane potential of mitochondria	1.50E-05	92
Liver	Rice straw	Negative	cell movement of lymphocytes	1.50E-05	161
Liver	Rice straw	Negative	differentiation of bone	1.52E-05	167
Liver	Rice straw	Negative	apoptosis of muscle	1.53E-05	110
Liver	Rice straw	Negative	apoptosis of endothelial cells	1.58E-05	80
Liver	Rice straw	Negative	internalization of cells	1.60E-05	73
Liver	Rice straw	Negative	concentration of Ca2+	1.62E-05	58
Liver	Rice straw	Negative	neuritogenesis	1.62E-05	232
Liver	Rice straw	Negative	migration of fibroblasts	1.62E-05	64
Liver	Rice straw	Negative	uptake of carbohydrate	1.63E-05	121
Liver	Rice straw	Negative	morphology of lymphatic system component	1.63E-05	169
Liver	Rice straw	Negative	immune response of cells	1.63E-05	228
Liver	Rice straw	Negative	Gastrointestinal Tract Cancer and Tumors	1.63E-05	1844
Liver	Rice straw	Negative	growth of embryonic tissue	1.64E-05	115
Liver	Rice straw	Negative	development of neurons	1.64E-05	306
Liver	Rice straw	Negative	neoplasia of tumor cell lines	1.75E-05	131
Liver	Rice straw	Negative	angiogenesis of tumor	1.75E-05	55
Liver	Rice straw	Negative	function of cardiovascular system	1.76E-05	151
Liver	Rice	Negative	cell movement of smooth muscle cells	1.77E-05	71

	straw				
Liver	Rice straw	Negative	morphogenesis of neurons	1.82E-05	172
Liver	Rice straw	Negative	transport of monosaccharide	1.87E-05	70
Liver	Rice straw	Negative	cell movement of mononuclear leukocytes	1.87E-05	186
Liver	Rice straw	Negative	activation of enzyme	1.88E-05	158
Liver	Rice straw	Negative	gastrointestinal carcinoma	1.89E-05	1631
Liver	Rice straw	Negative	cell movement of tumor cells	1.89E-05	80
Liver	Rice straw	Negative	apoptosis of muscle cells	1.90E-05	109
Liver	Rice straw	Negative	proliferation of thymocytes	1.90E-05	43
Liver	Rice straw	Negative	seizures	1.96E-05	162
Liver	Rice straw	Negative	differentiation of bone cells	1.96E-05	165
Liver	Rice straw	Negative	adenoma	2.00E-05	220
Liver	Rice straw	Negative	metastasis	2.07E-05	297
Liver	Rice straw	Negative	killing of macrophages	2.27E-05	13
Liver	Rice straw	Negative	Dermatitis	2.28E-05	175
Liver	Rice straw	Negative	breast or ovarian cancer	2.28E-05	711
Liver	Rice straw	Negative	infection of tumor cell lines	2.28E-05	204
Liver	Rice straw	Negative	homing of blood cells	2.28E-05	154
Liver	Rice straw	Negative	glucose metabolism disorder	2.28E-05	487
Liver	Rice straw	Negative	cell death of neuroblastoma cell lines	2.28E-05	83
Liver	Rice straw	Negative	formation of plasma membrane projections	2.30E-05	236
Liver	Rice straw	Negative	accumulation of blood cells	2.39E-05	116
Liver	Rice	Negative	epileptic seizure	2.40E-05	73

	straw				
Liver	Rice straw	Negative	peripheral arterial disease	2.43E-05	90
Liver	Rice straw	Negative	concentration of acylglycerol	2.44E-05	137
Liver	Rice straw	Negative	cell death of astrocytes	2.45E-05	23
Liver	Rice straw	Negative	adhesion of blood cells	2.45E-05	153
Liver	Rice straw	Negative	morphology of respiratory system	2.53E-05	128
Liver	Rice straw	Negative	morphology of skin	2.59E-05	113
Liver	Rice straw	Negative	recruitment of macrophages	2.59E-05	49
Liver	Rice straw	Negative	maturation of cells	2.62E-05	183
Liver	Rice straw	Negative	cell death of endothelial cells	2.69E-05	86
Liver	Rice straw	Negative	migration of cancer cells	2.76E-05	83
Liver	Rice straw	Negative	proliferation of embryonic cells	2.79E-05	104
Liver	Rice straw	Negative	mammary tumor	2.94E-05	613
Liver	Rice straw	Negative	abnormal morphology of nervous system	2.94E-05	283
Liver	Rice straw	Negative	vascularization	2.95E-05	99
Liver	Rice straw	Negative	abnormal morphology of respiratory system	2.97E-05	126
Liver	Rice straw	Negative	cell death of bone cancer cell lines	2.99E-05	76
Liver	Rice straw	Negative	quantity of immunoglobulin	3.08E-05	122
Liver	Rice straw	Negative	abnormal morphology of embryonic tissue	3.08E-05	217
Liver	Rice straw	Negative	dysmyelination	3.08E-05	60
Liver	Rice straw	Negative	formation of filopodia	3.13E-05	72
Liver	Rice straw	Negative	apoptosis of sarcoma cell lines	3.16E-05	81
Liver	Rice	Negative	bone mineral density	3.17E-05	75

	straw				
Liver	Rice straw	Negative	movement of vascular endothelial cells	3.18E-05	78
Liver	Rice straw	Negative	degeneration of neurons	3.20E-05	100
Liver	Rice straw	Negative	morphology of bone	3.32E-05	180
Liver	Rice straw	Negative	dephosphorylation of protein	3.38E-05	80
Liver	Rice straw	Negative	expansion of cells	3.48E-05	124
Liver	Rice straw	Negative	transformation of fibroblast cell lines	3.54E-05	107
Liver	Rice straw	Negative	permeability of cells	3.59E-05	46
Liver	Rice straw	Negative	cell death of central nervous system cells	3.59E-05	120
Liver	Rice straw	Negative	chemotaxis of myeloid cells	3.59E-05	120
Liver	Rice straw	Negative	quantity of reactive oxygen species	3.63E-05	76
Liver	Rice straw	Negative	production of reactive oxygen species	3.65E-05	148
Liver	Rice straw	Negative	phagocytosis of cells	3.77E-05	112
Liver	Rice straw	Negative	arrest in interphase	3.77E-05	167
Liver	Rice straw	Negative	migration of smooth muscle cells	3.77E-05	65
Liver	Rice straw	Negative	cell movement of macrophages	3.77E-05	110
Liver	Rice straw	Negative	homing of leukocytes	3.82E-05	152
Liver	Rice straw	Negative	concentration of phosphatidic acid	3.88E-05	60
Liver	Rice straw	Negative	size of cells	3.99E-05	172
Liver	Rice straw	Negative	recruitment of blood cells	4.02E-05	134
Liver	Rice straw	Negative	multiple congenital anomalies	4.03E-05	169
Liver	Rice straw	Negative	cell death of hematopoietic cells	4.04E-05	99
Liver	Rice	Negative	uptake of monosaccharide	4.04E-05	111

	straw				
Liver	Rice straw	Negative	recruitment of leukocytes	4.11E-05	132
Liver	Rice straw	Negative	autophagy	4.12E-05	151
Liver	Rice straw	Negative	dysgenesis	4.12E-05	213
Liver	Rice straw	Negative	generation of T lymphocytes	4.12E-05	52
Liver	Rice straw	Negative	adhesion of tumor cell lines	4.14E-05	118
Liver	Rice straw	Negative	synthesis of terpenoid	4.21E-05	116
Liver	Rice straw	Negative	branching of neurites	4.26E-05	114
Liver	Rice straw	Negative	formation of eye	4.35E-05	175
Liver	Rice straw	Negative	experimentally-induced arthritis	4.40E-05	66
Liver	Rice straw	Negative	advanced malignant tumor	4.50E-05	326
Liver	Rice straw	Negative	permeability of epithelial tissue	4.65E-05	32
Liver	Rice straw	Negative	cellular degradation	4.77E-05	105
Liver	Rice straw	Negative	size of lesion	4.77E-05	105
Liver	Rice straw	Negative	apoptosis of embryonic cells	4.79E-05	45
Liver	Rice straw	Negative	experimentally-induced diabetes	4.82E-05	44
Liver	Rice straw	Negative	morphology of muscle cells	4.96E-05	71
Liver	Rice straw	Negative	activation of lymphocytes	4.96E-05	183
Liver	Rice straw	Negative	recruitment of antigen presenting cells	4.98E-05	54
Liver	Rice straw	Negative	morphology of blood cells	5.08E-05	166
Liver	Rice straw	Negative	cell death of brain	5.09E-05	119
Liver	Rice straw	Negative	chronic inflammatory disorder	5.10E-05	372
Liver	Rice	Negative	branching of neurons	5.18E-05	117

	straw				
Liver	Rice straw	Negative	morphogenesis of neurites	5.24E-05	167
Liver	Rice straw	Negative	degeneration of central nervous system	5.35E-05	58
Liver	Rice straw	Negative	anoikis	5.56E-05	51
Liver	Rice straw	Negative	phagocytosis	5.78E-05	121
Liver	Rice straw	Negative	cell viability of cervical cancer cell lines	5.85E-05	92
Liver	Rice straw	Negative	size of bone	6.27E-05	113
Liver	Rice straw	Negative	neoplasia of cells	6.31E-05	176
Liver	Rice straw	Negative	metabolism of DNA	6.37E-05	159
Liver	Rice straw	Negative	engulfment of tumor cell lines	6.62E-05	63
Liver	Rice straw	Negative	size of embryo	7.07E-05	143
Liver	Rice straw	Negative	Wound	7.11E-05	94
Liver	Rice straw	Negative	cell death of lymphatic system cells	7.39E-05	51
Liver	Rice straw	Negative	concentration of cholesterol	7.51E-05	118
Liver	Rice straw	Negative	migration of vascular endothelial cells	7.62E-05	71
Liver	Rice straw	Negative	mass of connective tissue	7.74E-05	84
Liver	Rice straw	Negative	allergy	7.76E-05	161
Liver	Rice straw	Negative	apoptosis of T lymphocytes	7.80E-05	114
Liver	Rice straw	Negative	G1 phase	7.92E-05	155
Liver	Rice straw	Negative	adhesion of immune cells	7.94E-05	140
Liver	Rice straw	Negative	cell movement of antigen presenting cells	7.94E-05	140
Liver	Rice straw	Negative	apoptosis of mononuclear leukocytes	7.97E-05	143
Liver	Rice	Negative	morphology of lymphoid organ	8.18E-05	156

	straw				
Liver	Rice straw	Negative	development of connective tissue cells	8.29E-05	92
Liver	Rice straw	Negative	invasion of lung cancer cell lines	8.32E-05	47
Liver	Rice straw	Negative	cell death of hematopoietic progenitor cells	8.34E-05	94
Liver	Rice straw	Negative	differentiation of muscle cell lines	8.65E-05	69
Liver	Rice straw	Negative	formation of lamellipodia	8.65E-05	69
Liver	Rice straw	Negative	transport of D-glucose	8.78E-05	62
Liver	Rice straw	Negative	regeneration of muscle	8.80E-05	37
Liver	Rice straw	Negative	abdominal carcinoma	8.85E-05	2128
Liver	Rice straw	Negative	seizure disorder	9.01E-05	185
Liver	Rice straw	Negative	cell death of stem cells	9.04E-05	39
Liver	Rice straw	Negative	abnormal morphology of lymph node	9.04E-05	65
Liver	Rice straw	Negative	apoptosis of myeloid cells	9.26E-05	79
Liver	Rice straw	Negative	cellular infiltration by granulocytes	9.53E-05	88
Liver	Rice straw	Negative	cancer of cells	9.63E-05	104
Liver	Rice straw	Negative	diabetic nephropathy	9.63E-05	64
Liver	Rice straw	Negative	function of muscle	9.70E-05	139
Liver	Rice straw	Negative	morphology of intestine	9.77E-05	94
Liver	Rice straw	Negative	shape change of neurons	9.79E-05	118
Liver	Rice straw	Negative	differentiation of epithelial cells	1.00E-04	126
Liver	Rice straw	Negative	morphology of reproductive system	1.01E-04	209
Liver	Rice straw	Negative	migration of carcinoma cell lines	1.05E-04	72
Liver	Rice	Negative	binding of endothelial cells	1.09E-04	47

	straw				
Liver	Rice straw	Negative	shape change of neurites	1.09E-04	115
Liver	Rice straw	Negative	cytopenia	1.10E-04	120
Liver	Rice straw	Negative	concentration of sterol	1.13E-04	123
Liver	Rice straw	Negative	function of phagocytes	1.13E-04	123
Liver	Rice straw	Negative	cell death of myeloid cells	1.13E-04	88
Liver	Rice straw	Negative	colorectal neoplasia	1.15E-04	1555
Liver	Rice straw	Negative	expansion of blood cells	1.15E-04	94
Liver	Rice straw	Negative	aggregation of blood cells	1.15E-04	96
Liver	Rice straw	Negative	synthesis of polysaccharide	1.15E-04	76
Liver	Rice straw	Negative	formation of lung	1.15E-04	121
Liver	Rice straw	Negative	abnormal morphology of lymphoid organ	1.17E-04	150
Liver	Rice straw	Negative	chemotaxis of leukocytes	1.19E-04	141
Liver	Rice straw	Negative	serine phosphorylation	1.19E-04	43
Liver	Rice straw	Negative	concentration of glutathione	1.22E-04	37
Liver	Corn stover	Positive	proliferation of cells	1.19E-53	1663
Liver	Corn stover	Positive	cancer	2.00E-40	4119
Liver	Corn stover	Positive	cell death	2.00E-40	1472
Liver	Corn stover	Positive	malignant solid tumor	4.04E-39	4069
Liver	Corn stover	Positive	organismal death	6.73E-38	1100
Liver	Corn stover	Positive	morbidity or mortality	8.40E-38	1112
Liver	Corn stover	Positive	morphology of cells	2.19E-37	921
Liver	Corn	Positive	necrosis	1.05E-33	1150

	stover				
Liver	Corn stover	Positive	apoptosis	2.22E-32	1171
Liver	Corn stover	Positive	cell movement	2.98E-32	972
Liver	Corn stover	Positive	organization of cytoplasm	6.47E-30	727
Liver	Corn stover	Positive	migration of cells	3.25E-28	867
Liver	Corn stover	Positive	neoplasia of epithelial tissue	3.12E-26	3378
Liver	Corn stover	Positive	tumorigenesis of tissue	4.41E-26	3428
Liver	Corn stover	Positive	epithelial cancer	1.18E-24	3339
Liver	Corn stover	Positive	organization of cytoskeleton	1.18E-24	651
Liver	Corn stover	Positive	cellular homeostasis	1.41E-24	701
Liver	Corn stover	Positive	vasculogenesis	4.30E-24	377
Liver	Corn stover	Positive	angiogenesis	6.11E-24	449
Liver	Corn stover	Positive	abnormal morphology of cells	3.18E-23	593
Liver	Corn stover	Positive	expression of RNA	4.05E-23	937
Liver	Corn stover	Positive	transcription	9.10E-23	877
Liver	Corn stover	Positive	abdominal neoplasm	2.09E-22	3330
Liver	Corn stover	Positive	cell survival	4.32E-22	639
Liver	Corn stover	Positive	transport of molecule	7.74E-22	724
Liver	Corn stover	Positive	quantity of cells	3.08E-21	782
Liver	Corn stover	Positive	abdominal cancer	7.15E-21	3284
Liver	Corn stover	Positive	differentiation of cells	9.89E-21	964
Liver	Corn stover	Positive	cell death of tumor cell lines	1.06E-20	685
Liver	Corn	Positive	transcription of RNA	1.40E-20	813

	stover				
Liver	Corn stover	Positive	invasion of cells	1.65E-20	421
Liver	Corn stover	Positive	cell viability	3.10E-20	594
Liver	Corn stover	Positive	cell movement of tumor cell lines	3.93E-20	412
Liver	Corn stover	Positive	microtubule dynamics	2.25E-19	544
Liver	Corn stover	Positive	Viral Infection	2.58E-19	712
Liver	Corn stover	Positive	apoptosis of tumor cell lines	1.03E-18	552
Liver	Corn stover	Positive	proliferation of tumor cell lines	1.71E-18	683
Liver	Corn stover	Positive	cell death of connective tissue cells	1.71E-18	301
Liver	Corn stover	Positive	morphology of cardiovascular system	4.16E-18	307
Liver	Corn stover	Positive	digestive organ tumor	1.14E-17	2872
Liver	Corn stover	Positive	digestive system cancer	1.14E-17	2847
Liver	Corn stover	Positive	migration of tumor cell lines	1.48E-17	342
Liver	Corn stover	Positive	quantity of leukocytes	2.17E-17	428
Liver	Corn stover	Positive	quantity of blood cells	6.80E-17	471
Liver	Corn stover	Positive	invasion of tumor cell lines	1.55E-16	321
Liver	Corn stover	Positive	Movement Disorders	1.58E-16	482
Liver	Corn stover	Positive	proliferation of blood cells	2.12E-16	394
Liver	Corn stover	Positive	proliferation of connective tissue cells	3.06E-15	286
Liver	Corn stover	Positive	morphology of body cavity	4.23E-15	529
Liver	Corn stover	Positive	growth of connective tissue	4.33E-15	307
Liver	Corn stover	Positive	proliferation of immune cells	4.82E-15	368
Liver	Corn	Positive	development of body trunk	4.96E-15	498

	stover				
Liver	Corn stover	Positive	synthesis of lipid	8.57E-15	335
Liver	Corn stover	Positive	protein kinase cascade	8.57E-15	211
Liver	Corn stover	Positive	transcription of DNA	1.27E-14	658
Liver	Corn stover	Positive	function of leukocytes	1.41E-14	260
Liver	Corn stover	Positive	quantity of lymphocytes	1.59E-14	331
Liver	Corn stover	Positive	quantity of mononuclear leukocytes	2.18E-14	342
Liver	Corn stover	Positive	proliferation of mononuclear leukocytes	2.32E-14	343
Liver	Corn stover	Positive	growth of epithelial tissue	2.32E-14	319
Liver	Corn stover	Positive	autosomal recessive disease	2.83E-14	417
Liver	Corn stover	Positive	proliferation of lymphocytes	2.83E-14	338
Liver	Corn stover	Positive	survival of organism	3.13E-14	328
Liver	Corn stover	Positive	function of blood cells	4.30E-14	279
Liver	Corn stover	Positive	formation of cellular protrusions	5.74E-14	408
Liver	Corn stover	Positive	abnormal morphology of body cavity	7.31E-14	502
Liver	Corn stover	Positive	organization of organelle	8.84E-14	258
Liver	Corn stover	Positive	concentration of lipid	9.79E-14	370
Liver	Corn stover	Positive	size of body	1.78E-13	385
Liver	Corn stover	Positive	cell death of fibroblast cell lines	2.17E-13	209
Liver	Corn stover	Positive	benign neoplasia	2.92E-13	414
Liver	Corn stover	Positive	migration of blood cells	2.92E-13	393
Liver	Corn stover	Positive	cell death of blood cells	2.97E-13	320
Liver	Corn	Positive	leukocyte migration	3.60E-13	392

	stover				
Liver	Corn stover	Positive	abnormal morphology of cardiovascular system	5.27E-13	267
Liver	Corn stover	Positive	development of vasculature	5.46E-13	204
Liver	Corn stover	Positive	transactivation	6.67E-13	271
Liver	Corn stover	Positive	abnormal morphology of thoracic cavity	9.14E-13	274
Liver	Corn stover	Positive	apoptosis of fibroblast cell lines	1.28E-12	164
Liver	Corn stover	Positive	neuronal cell death	1.71E-12	312
Liver	Corn stover	Positive	disorder of basal ganglia	2.55E-12	346
Liver	Corn stover	Positive	development of cytoplasm	3.10E-12	220
Liver	Corn stover	Positive	growth of organism	3.90E-12	373
Liver	Corn stover	Positive	cell cycle progression	4.33E-12	456
Liver	Corn stover	Positive	phosphorylation of protein	4.89E-12	332
Liver	Corn stover	Positive	cell death of immune cells	5.49E-12	301
Liver	Corn stover	Positive	cell movement of leukocytes	7.08E-12	344
Liver	Corn stover	Positive	cell transformation	7.68E-12	218
Liver	Corn stover	Positive	proliferation of fibroblast cell lines	9.83E-12	202
Liver	Corn stover	Positive	abnormal morphology of abdomen	1.10E-11	387
Liver	Corn stover	Positive	cell viability of tumor cell lines	1.76E-11	347
Liver	Corn stover	Positive	development of blood cells	1.77E-11	296
Liver	Corn stover	Positive	cell movement of myeloid cells	2.83E-11	249
Liver	Corn stover	Positive	development of leukocytes	2.83E-11	269
Liver	Corn stover	Positive	necrosis of epithelial tissue	2.83E-11	269
Liver	Corn	Positive	morphology of lymphatic system component	3.76E-11	197

	stover				
Liver	Corn stover	Positive	degeneration of nervous system	3.83E-11	142
Liver	Corn stover	Positive	quantity of connective tissue	4.33E-11	274
Liver	Corn stover	Positive	infection by Retroviridae	4.91E-11	321
Liver	Corn stover	Positive	ubiquitination	4.91E-11	163
Liver	Corn stover	Positive	interphase	6.41E-11	295
Liver	Corn stover	Positive	metabolism of membrane lipid derivative	6.79E-11	184
Liver	Corn stover	Positive	formation of cytoskeleton	7.44E-11	179
Liver	Corn stover	Positive	fatty acid metabolism	7.95E-11	272
Liver	Corn stover	Positive	quantity of T lymphocytes	8.53E-11	245
Liver	Corn stover	Positive	ubiquitination of protein	8.88E-11	160
Liver	Corn stover	Positive	cell death of epithelial cells	9.67E-11	229
Liver	Corn stover	Positive	proliferation of T lymphocytes	9.85E-11	273
Liver	Corn stover	Positive	proliferation of hematopoietic cells	1.00E-10	127
Liver	Corn stover	Positive	activation of DNA endogenous promoter	1.19E-10	511
Liver	Corn stover	Positive	neuromuscular disease	1.33E-10	384
Liver	Corn stover	Positive	growth of embryo	1.52E-10	220
Liver	Corn stover	Positive	perinatal death	1.67E-10	268
Liver	Corn stover	Positive	development of cardiovascular tissue	1.70E-10	175
Liver	Corn stover	Positive	development of lymphocytes	1.97E-10	251
Liver	Corn stover	Positive	infection by lentivirus	2.12E-10	316
Liver	Corn stover	Positive	HIV infection	2.29E-10	315
Liver	Corn	Positive	adenocarcinoma	2.51E-10	2451

	stover				
Liver	Corn stover	Positive	metabolism of carbohydrate	2.58E-10	278
Liver	Corn stover	Positive	infection by RNA virus	2.74E-10	376
Liver	Corn stover	Positive	development of mononuclear leukocytes	2.89E-10	252
Liver	Corn stover	Positive	small GTPase mediated signal transduction	3.15E-10	95
Liver	Corn stover	Positive	development of epithelial tissue	3.26E-10	235
Liver	Corn stover	Positive	cell movement of phagocytes	3.89E-10	248
Liver	Corn stover	Positive	neurological signs	4.72E-10	289
Liver	Corn stover	Positive	replication of RNA virus	4.72E-10	230
Liver	Corn stover	Positive	Neurodegeneration	4.84E-10	148
Liver	Corn stover	Positive	transactivation of RNA	5.68E-10	245
Liver	Corn stover	Positive	colony formation	6.04E-10	242
Liver	Corn stover	Positive	differentiation of connective tissue cells	6.35E-10	285
Liver	Corn stover	Positive	Organ Degeneration	7.26E-10	224
Liver	Corn stover	Positive	morphology of lymphoid organ	7.79E-10	182
Liver	Corn stover	Positive	dyskinesia	8.25E-10	274
Liver	Corn stover	Positive	development of connective tissue	9.41E-10	173
Liver	Corn stover	Positive	morphology of connective tissue	1.00E-09	210
Liver	Corn stover	Positive	proliferation of hematopoietic progenitor cells	1.00E-09	119
Liver	Corn stover	Positive	morphology of vessel	1.03E-09	146
Liver	Corn stover	Positive	apoptosis of blood cells	1.17E-09	224
Liver	Corn stover	Positive	morphology of heart	1.17E-09	187
Liver	Corn	Positive	autophagy	1.28E-09	174

	stover				
Liver	Corn stover	Positive	Lymphocyte homeostasis	1.29E-09	244
Liver	Corn stover	Positive	apoptosis of connective tissue cells	1.32E-09	141
Liver	Corn stover	Positive	quantity of lymphatic system component	1.44E-09	175
Liver	Corn stover	Positive	fibrogenesis	1.45E-09	189
Liver	Corn stover	Positive	endothelial cell development	1.49E-09	165
Liver	Corn stover	Positive	colony formation of cells	1.55E-09	222
Liver	Corn stover	Positive	breast or ovarian cancer	1.60E-09	770
Liver	Corn stover	Positive	development of endothelial tissue	1.60E-09	170
Liver	Corn stover	Positive	abdominal adenocarcinoma	1.66E-09	2229
Liver	Corn stover	Positive	development of lymphatic system	1.67E-09	166
Liver	Corn stover	Positive	replication of virus	1.69E-09	250
Liver	Corn stover	Positive	development of lymphatic system component	1.90E-09	146
Liver	Corn stover	Positive	homeostasis of leukocytes	2.13E-09	247
Liver	Corn stover	Positive	quantity of carbohydrate	2.22E-09	232
Liver	Corn stover	Positive	activation of cells	2.43E-09	414
Liver	Corn stover	Positive	cell death of fibroblasts	2.43E-09	127
Liver	Corn stover	Positive	abnormal morphology of epithelial tissue	2.49E-09	191
Liver	Corn stover	Positive	metabolism of protein	2.49E-09	412
Liver	Corn stover	Positive	morphology of digestive system	2.80E-09	253
Liver	Corn stover	Positive	cellular infiltration by leukocytes	2.90E-09	178
Liver	Corn stover	Positive	engulfment of cells	2.90E-09	186
Liver	Corn	Positive	abnormal morphology of digestive system	3.01E-09	242

	stover				
Liver	Corn stover	Positive	female genital tract serous cancer	3.06E-09	177
Liver	Corn stover	Positive	T cell development	3.07E-09	225
Liver	Corn stover	Positive	chorea	3.14E-09	255
Liver	Corn stover	Positive	differentiation of connective tissue	3.37E-09	316
Liver	Corn stover	Positive	Huntington's Disease	3.37E-09	254
Liver	Corn stover	Positive	concentration of phospholipid	3.39E-09	92
Liver	Corn stover	Positive	autosomal dominant disease	3.59E-09	279
Liver	Corn stover	Positive	arthropathy	3.82E-09	403
Liver	Corn stover	Positive	cell movement of neutrophils	3.84E-09	140
Liver	Corn stover	Positive	morphology of bone	4.05E-09	203
Liver	Corn stover	Positive	congenital anomaly of musculoskeletal system	4.12E-09	318
Liver	Corn stover	Positive	advanced malignant tumor	4.45E-09	361
Liver	Corn stover	Positive	secretion of molecule	4.51E-09	244
Liver	Corn stover	Positive	infection of cells	4.60E-09	342
Liver	Corn stover	Positive	cellular infiltration of cells	4.63E-09	181
Liver	Corn stover	Positive	quantity of lymphoid organ	4.63E-09	144
Liver	Corn stover	Positive	cell death of T lymphocytes	4.78E-09	147
Liver	Corn stover	Positive	vascularization	4.78E-09	114
Liver	Corn stover	Positive	proliferation of epithelial cells	4.91E-09	220
Liver	Corn stover	Positive	breast or colorectal cancer	5.41E-09	1885
Liver	Corn stover	Positive	abnormal morphology of lymphoid organ	5.42E-09	173
Liver	Corn	Positive	formation of leukocytes	5.44E-09	81

	stover				
Liver	Corn stover	Positive	differentiation of leukocytes	5.48E-09	292
Liver	Corn stover	Positive	synthesis of carbohydrate	6.05E-09	192
Liver	Corn stover	Positive	endocytosis	6.23E-09	159
Liver	Corn stover	Positive	experimentally-induced diabetes	6.23E-09	53
Liver	Corn stover	Positive	morphology of blood vessel	6.56E-09	133
Liver	Corn stover	Positive	genital tumor	6.83E-09	1548
Liver	Corn stover	Positive	cellular infiltration	6.95E-09	197
Liver	Corn stover	Positive	arthritis	7.26E-09	395
Liver	Corn stover	Positive	quantity of B lymphocytes	7.45E-09	159
Liver	Corn stover	Positive	synthesis of fatty acid	7.87E-09	145
Liver	Corn stover	Positive	morphology of nervous system	8.38E-09	335
Liver	Corn stover	Positive	peripheral vascular disease	9.16E-09	173
Liver	Corn stover	Positive	homing of cells	9.48E-09	245
Liver	Corn stover	Positive	formation of blood cells	9.56E-09	97
Liver	Corn stover	Positive	formation of filaments	9.56E-09	180
Liver	Corn stover	Positive	formation of lymphoid organ	9.56E-09	125
Liver	Corn stover	Positive	formation of cells	9.73E-09	412
Liver	Corn stover	Positive	growth of lesion	1.01E-08	340
Liver	Corn stover	Positive	T cell homeostasis	1.03E-08	228
Liver	Corn stover	Positive	function of phagocytes	1.13E-08	142
Liver	Corn stover	Positive	differentiation of epithelial tissue	1.13E-08	164
Liver	Corn	Positive	growth of tumor	1.22E-08	339

	stover				
Liver	Corn stover	Positive	formation of lymphatic system component	1.29E-08	136
Liver	Corn stover	Positive	cell death of epithelial cell lines	1.29E-08	132
Liver	Corn stover	Positive	gastrointestinal carcinoma	1.40E-08	1722
Liver	Corn stover	Positive	infection by HIV-1	1.41E-08	265
Liver	Corn stover	Positive	serous neoplasm	1.41E-08	265
Liver	Corn stover	Positive	cell death of mononuclear leukocytes	1.42E-08	179
Liver	Corn stover	Positive	cell movement of granulocytes	1.48E-08	168
Liver	Corn stover	Positive	abnormal morphology of bone	1.48E-08	195
Liver	Corn stover	Positive	binding of DNA	1.70E-08	229
Liver	Corn stover	Positive	degeneration of cells	1.75E-08	157
Liver	Corn stover	Positive	morphology of head	1.84E-08	369
Liver	Corn stover	Positive	I-kappaB kinase/NF-kappaB cascade	1.87E-08	86
Liver	Corn stover	Positive	homing	1.91E-08	250
Liver	Corn stover	Positive	formation of skin	2.01E-08	166
Liver	Corn stover	Positive	function of myeloid cells	2.01E-08	113
Liver	Corn stover	Positive	gonadal tumor	2.02E-08	347
Liver	Corn stover	Positive	morphology of skin	2.03E-08	127
Liver	Corn stover	Positive	concentration of fatty acid	2.15E-08	130
Liver	Corn stover	Positive	metabolism of phospholipid	2.16E-08	111
Liver	Corn stover	Positive	epileptic seizure	2.52E-08	83
Liver	Corn stover	Positive	cell movement of endothelial cells	3.02E-08	160
Liver	Corn	Positive	cell death of lymphocytes	3.02E-08	173

	stover				
Liver	Corn stover	Positive	apoptosis of leukocytes	3.19E-08	202
Liver	Corn stover	Positive	size of embryo	3.37E-08	161
Liver	Corn stover	Positive	congenital malformation of skeleton	3.37E-08	188
Liver	Corn stover	Positive	behavior	3.41E-08	411
Liver	Corn stover	Positive	differentiation of bone	3.44E-08	183
Liver	Corn stover	Positive	development of abdomen	3.44E-08	251
Liver	Corn stover	Positive	development of head	3.44E-08	408
Liver	Corn stover	Positive	sprouting	3.52E-08	173
Liver	Corn stover	Positive	cell spreading	3.62E-08	128
Liver	Corn stover	Positive	synthesis of nitric oxide	3.62E-08	128
Liver	Corn stover	Positive	concentration of phosphatidic acid	3.78E-08	69
Liver	Corn stover	Positive	adenoma	3.95E-08	240
Liver	Corn stover	Positive	apoptosis of cervical cancer cell lines	3.98E-08	130
Liver	Corn stover	Positive	quantity of immunoglobulin	4.24E-08	136
Liver	Corn stover	Positive	abnormal morphology of head	4.24E-08	350
Liver	Corn stover	Positive	size of animal	4.27E-08	87
Liver	Corn stover	Positive	proliferation of fibroblasts	4.45E-08	159
Liver	Corn stover	Positive	proliferation of neuronal cells	4.45E-08	249
Liver	Corn stover	Positive	generation of leukocytes	4.45E-08	80
Liver	Corn stover	Positive	differentiation of bone cells	4.53E-08	181
Liver	Corn stover	Positive	synthesis of DNA	4.70E-08	185
Liver	Corn	Positive	cell death of cervical cancer cell lines	4.70E-08	158

	stover				
Liver	Corn stover	Positive	development of body axis	4.72E-08	434
Liver	Corn stover	Positive	tumorigenesis of gonad	5.07E-08	340
Liver	Corn stover	Positive	cell death of liver	5.11E-08	112
Liver	Corn stover	Positive	abdominal carcinoma	5.18E-08	2242
Liver	Corn stover	Positive	activation of enzyme	5.66E-08	173
Liver	Corn stover	Positive	recruitment of cells	5.85E-08	160
Liver	Corn stover	Positive	growth of skin	5.89E-08	103
Liver	Corn stover	Positive	generation of blood cells	5.89E-08	81
Liver	Corn stover	Positive	gastrointestinal tract cancer	6.02E-08	1905
Liver	Corn stover	Positive	G1 phase	6.67E-08	173
Liver	Corn stover	Positive	necrosis of liver	6.70E-08	111
Liver	Corn stover	Positive	hypersensitive reaction	7.79E-08	189
Liver	Corn stover	Positive	abnormal morphology of lymph node	8.00E-08	75
Liver	Corn stover	Positive	morphology of lymph node	8.02E-08	79
Liver	Corn stover	Positive	Oral Cancer and Tumors	8.04E-08	107
Liver	Corn stover	Positive	metastasis	8.18E-08	320
Liver	Corn stover	Positive	growth of lymphatic system component	8.48E-08	92
Liver	Corn stover	Positive	Ovarian Cancer and Tumors	8.80E-08	319
Liver	Corn stover	Positive	craniofacial abnormality	8.80E-08	159
Liver	Corn stover	Positive	quantity of hematopoietic cells	9.96E-08	194
Liver	Corn stover	Positive	differentiation of blood cells	1.03E-07	352
Liver	Corn	Positive	oral cancer	1.06E-07	106

	stover				
Liver	Corn stover	Positive	cell death of liver cells	1.07E-07	93
Liver	Corn stover	Positive	apoptosis of epithelial cells	1.07E-07	130
Liver	Corn stover	Positive	function of mononuclear leukocytes	1.11E-07	153
Liver	Corn stover	Positive	neoplasia of cells	1.15E-07	194
Liver	Corn stover	Positive	arrest in interphase	1.18E-07	183
Liver	Corn stover	Positive	organization of filaments	1.19E-07	97
Liver	Corn stover	Positive	quantity of hematopoietic progenitor cells	1.23E-07	193
Liver	Corn stover	Positive	uptake of monosaccharide	1.31E-07	123
Liver	Corn stover	Positive	migration of endothelial cells	1.38E-07	147
Liver	Corn stover	Positive	mammary tumor	1.39E-07	652
Liver	Corn stover	Positive	multiple congenital anomalies	1.39E-07	185
Liver	Corn stover	Positive	function of lymphocytes	1.40E-07	152
Liver	Corn stover	Positive	cell viability of carcinoma cell lines	1.41E-07	71
Liver	Corn stover	Positive	cardiogenesis	1.42E-07	205
Liver	Corn stover	Positive	morphology of cardiovascular tissue	1.42E-07	47
Liver	Corn stover	Positive	quantity of thymus gland	1.49E-07	108
Liver	Corn stover	Positive	abnormal morphology of embryonic tissue	1.57E-07	235
Liver	Corn stover	Positive	development of neurons	1.57E-07	327
Liver	Corn stover	Positive	growth of muscle tissue	1.57E-07	165
Liver	Corn stover	Positive	paraproteinemia	1.57E-07	150
Liver	Corn stover	Positive	abnormal morphology of heart	1.65E-07	169
Liver	Corn	Positive	quantity of epithelial tissue	1.67E-07	85

	stover				
Liver	Corn stover	Positive	proliferation of muscle cells	1.68E-07	164
Liver	Corn stover	Positive	Gastrointestinal Tract Cancer and Tumors	1.70E-07	1929
Liver	Corn stover	Positive	midline defect	1.75E-07	121
Liver	Corn stover	Positive	production of antibody	1.75E-07	140
Liver	Corn stover	Positive	apoptosis of epithelial cell lines	1.76E-07	104
Liver	Corn stover	Positive	morphology of secondary lymphoid organ	1.83E-07	127
Liver	Corn stover	Positive	expansion of cells	1.87E-07	136
Liver	Corn stover	Positive	cell death of skin	1.98E-07	54
Liver	Corn stover	Positive	degeneration of neurons	2.07E-07	110
Liver	Corn stover	Positive	development of hematopoietic system	2.07E-07	114
Liver	Corn stover	Positive	function of cardiovascular system	2.10E-07	163
Liver	Corn stover	Positive	uptake of carbohydrate	2.11E-07	131
Liver	Corn stover	Positive	quantity of phagocytes	2.11E-07	185
Liver	Corn stover	Positive	Dermatitis	2.13E-07	189
Liver	Corn stover	Positive	differentiation of embryonic tissue	2.28E-07	116
Liver	Corn stover	Positive	chemotaxis of cells	2.31E-07	226
Liver	Corn stover	Positive	proliferation of epidermal cells	2.32E-07	81
Liver	Corn stover	Positive	plasma cell dyscrasia	2.33E-07	149
Liver	Corn stover	Positive	organization of actin cytoskeleton	2.37E-07	135
Liver	Corn stover	Positive	cell viability of connective tissue cells	2.37E-07	66
Liver	Corn stover	Positive	differentiation of epithelial cells	2.47E-07	140
Liver	Corn	Positive	differentiation of lymphocytes	2.47E-07	214

	stover				
Liver	Corn stover	Positive	differentiation of embryonic cells	2.60E-07	105
Liver	Corn stover	Positive	morphology of respiratory system	2.62E-07	139
Liver	Corn stover	Positive	proliferation of endothelial cells	2.62E-07	139
Liver	Corn stover	Positive	ovarian cancer	2.78E-07	298
Liver	Corn stover	Positive	function of antigen presenting cells	2.79E-07	112
Liver	Corn stover	Positive	morphology of spleen	2.80E-07	126
Liver	Corn stover	Positive	production of protein	2.81E-07	146
Liver	Corn stover	Positive	ion homeostasis of cells	2.81E-07	237
Liver	Corn stover	Positive	Inflammatory Bowel Disease	3.21E-07	117
Liver	Corn stover	Positive	recruitment of leukocytes	3.22E-07	144
Liver	Corn stover	Positive	inflammation of organ	3.31E-07	475
Liver	Corn stover	Positive	generation of lymphocytes	3.34E-07	69
Liver	Corn stover	Positive	Rheumatic Disease	3.50E-07	438
Liver	Corn stover	Positive	peripheral arterial disease	3.56E-07	98
Liver	Corn stover	Positive	chemotaxis	3.59E-07	232
Liver	Corn stover	Positive	Growth Failure	3.81E-07	253
Liver	Corn stover	Positive	seizure disorder	3.94E-07	202
Liver	Corn stover	Positive	formation of osteoclasts	4.02E-07	61
Liver	Corn stover	Positive	apoptosis of skin	4.09E-07	49
Liver	Corn stover	Positive	recruitment of macrophages	4.13E-07	54
Liver	Corn stover	Positive	inflammatory response	4.19E-07	323
Liver	Corn	Positive	female genital neoplasm	4.23E-07	1351

	stover				
Liver	Corn stover	Positive	blood protein disorder	4.26E-07	164
Liver	Corn stover	Positive	apoptosis of liver	4.27E-07	76
Liver	Corn stover	Positive	formation of lung	4.28E-07	134
Liver	Corn stover	Positive	cellular degradation	4.33E-07	115
Liver	Corn stover	Positive	abnormal morphology of nervous system	4.33E-07	302
Liver	Corn stover	Positive	cell death of breast cancer cell lines	4.33E-07	147
Liver	Corn stover	Positive	cell death of keratinocytes	4.38E-07	46
Liver	Corn stover	Positive	generation of mononuclear leukocytes	4.42E-07	70
Liver	Corn stover	Positive	development of digestive system	4.65E-07	167
Liver	Corn stover	Positive	synthesis of phospholipid	4.73E-07	83
Liver	Corn stover	Positive	production of reactive oxygen species	4.73E-07	160
Liver	Corn stover	Positive	mitosis	4.74E-07	212
Liver	Corn stover	Positive	cell death of heart cells	4.74E-07	101
Liver	Corn stover	Positive	apoptosis of dermal cells	4.88E-07	47
Liver	Corn stover	Positive	cell death of embryonic cells	4.93E-07	58
Liver	Corn stover	Positive	apoptosis of hematopoietic cell lines	4.94E-07	95
Liver	Corn stover	Positive	apoptosis of fibroblasts	4.94E-07	100
Liver	Corn stover	Positive	cell death of heart	5.05E-07	104
Liver	Corn stover	Positive	maturation of cells	5.11E-07	196
Liver	Corn stover	Positive	morphology of endothelial tissue	5.13E-07	44
Liver	Corn stover	Positive	recruitment of antigen presenting cells	5.23E-07	60
Liver	Corn	Positive	abnormal morphology of respiratory system	5.23E-07	136

	stover				
Liver	Corn stover	Positive	cell death of epidermal cells	5.34E-07	48
Liver	Corn stover	Positive	neuritogenesis	5.40E-07	246
Liver	Corn stover	Positive	Bleeding	5.57E-07	183
Liver	Corn stover	Positive	cell death of tumor	5.69E-07	166
Liver	Corn stover	Positive	growth of embryonic tissue	5.70E-07	123
Liver	Corn stover	Positive	recruitment of blood cells	5.70E-07	145
Liver	Corn stover	Positive	quantity of thymocytes	5.86E-07	105
Liver	Corn stover	Positive	branching of cells	5.89E-07	161
Liver	Corn stover	Positive	synthesis of reactive oxygen species	5.91E-07	205
Liver	Corn stover	Positive	proliferation of dermal cells	5.92E-07	83
Liver	Corn stover	Positive	abnormal morphology of blood vessel	5.94E-07	119
Liver	Corn stover	Positive	cell viability of blood cells	6.00E-07	128
Liver	Corn stover	Positive	apoptosis of T lymphocytes	6.00E-07	125
Liver	Corn stover	Positive	apoptosis of mononuclear leukocytes	6.01E-07	156
Liver	Corn stover	Positive	differentiation of mononuclear leukocytes	6.14E-07	229
Liver	Corn stover	Positive	cell death of dermal cells	6.24E-07	50
Liver	Corn stover	Positive	proliferation of smooth muscle cells	6.35E-07	127
Liver	Corn stover	Positive	cytopenia	6.64E-07	132
Liver	Corn stover	Positive	S phase	6.64E-07	117
Liver	Corn stover	Positive	abnormal morphology of skin	6.66E-07	110
Liver	Corn stover	Positive	learning	6.73E-07	180
Liver	Corn	Positive	polymerization of protein	6.73E-07	161

	stover				
Liver	Corn stover	Positive	apoptosis of keratinocytes	6.73E-07	43
Liver	Corn stover	Positive	apoptosis of neurons	7.18E-07	187
Liver	Corn stover	Positive	tumorigenesis of genital organ	7.18E-07	1478
Liver	Corn stover	Positive	benign neoplasm of female genital organ	7.20E-07	183
Liver	Corn stover	Positive	dysgenesis	7.40E-07	228
Liver	Corn stover	Positive	quantity of IgG	7.88E-07	103
Liver	Corn stover	Positive	apoptosis of muscle	8.02E-07	117
Liver	Corn stover	Positive	transmembrane potential of mitochondria	8.16E-07	98
Liver	Corn stover	Positive	genital tract cancer	8.39E-07	1465
Liver	Corn stover	Positive	cognition	8.63E-07	194
Liver	Corn stover	Positive	apoptosis of epidermal cells	8.64E-07	45
Liver	Corn stover	Positive	seizures	8.91E-07	172
Liver	Corn stover	Positive	quantity of glycosphingolipid	9.08E-07	50
Liver	Corn stover	Positive	autophagy of cells	9.11E-07	118
Liver	Corn stover	Positive	migration of connective tissue cells	9.26E-07	84
Liver	Corn stover	Positive	apoptosis of liver cells	9.41E-07	74
Liver	Corn stover	Positive	cell death of hematopoietic cells	9.57E-07	107
Liver	Corn stover	Positive	skin abnormality	9.65E-07	103
Liver	Corn stover	Positive	formation of actin filaments	9.72E-07	134
Liver	Corn stover	Positive	chronic inflammatory disorder	9.72E-07	395
Liver	Corn stover	Positive	neoplasia of tumor cell lines	9.78E-07	139
Liver	Corn	Positive	accumulation of cells	9.98E-07	153

	stover				
Liver	Corn stover	Positive	bone mineral density	1.01E-06	81
Liver	Corn stover	Positive	morphogenesis of neurons	1.01E-06	182
Liver	Corn stover	Positive	apoptosis of muscle cells	1.01E-06	116
Liver	Corn stover	Positive	allergy	1.02E-06	174
Liver	Corn stover	Positive	uptake of D-hexose	1.06E-06	101
Liver	Corn stover	Positive	proliferation of lymphatic system cells	1.06E-06	92
Liver	Corn stover	Positive	adhesion of tumor cell lines	1.07E-06	127
Liver	Corn stover	Positive	metabolism of nucleic acid component or derivative	1.09E-06	219
Liver	Corn stover	Positive	cell death of hematopoietic cell lines	1.11E-06	104
Liver	Corn stover	Positive	uptake of D-glucose	1.11E-06	100
Liver	Corn stover	Positive	immune response of cells	1.13E-06	240
Liver	Corn stover	Positive	necrosis of tumor	1.14E-06	164
Liver	Corn stover	Positive	cell death of muscle cells	1.16E-06	146
Liver	Corn stover	Positive	cell surface receptor linked signal transduction	1.25E-06	130
Liver	Corn stover	Positive	morphology of reproductive system	1.31E-06	225
Liver	Corn stover	Positive	apoptosis of heart cells	1.31E-06	87
Liver	Corn stover	Positive	formation of plasma membrane projections	1.33E-06	249
Liver	Corn stover	Positive	migration of fibroblasts	1.33E-06	68
Liver	Corn stover	Positive	metabolism of reactive oxygen species	1.34E-06	211
Liver	Corn stover	Positive	neonatal death	1.40E-06	187
Liver	Corn stover	Positive	growth of neurites	1.42E-06	196
Liver	Corn	Positive	apoptosis of lymphocytes	1.42E-06	150

	stover				
Liver	Corn stover	Positive	differentiation of tumor cell lines	1.42E-06	163
Liver	Corn stover	Positive	recruitment of phagocytes	1.43E-06	110
Liver	Corn stover	Positive	migration of muscle cells	1.45E-06	77
Liver	Corn stover	Positive	transport of protein	1.54E-06	132
Liver	Corn stover	Positive	activation of Protein kinase	1.55E-06	112
Liver	Corn stover	Positive	abnormal morphology of spleen	1.56E-06	118
Liver	Corn stover	Positive	MAPKKK cascade	1.57E-06	93
Liver	Corn stover	Positive	respiratory system development	1.63E-06	148
Liver	Corn stover	Positive	breast cancer	1.65E-06	601
Liver	Corn stover	Positive	development of connective tissue cells	1.68E-06	100
Liver	Corn stover	Positive	concentration of acylglycerol	1.71E-06	145
Liver	Corn stover	Positive	apoptosis of embryonic cells	1.75E-06	49
Liver	Corn stover	Positive	generation of cells	1.76E-06	116
Liver	Corn stover	Positive	invasion of carcinoma cell lines	1.80E-06	90
Liver	Corn stover	Positive	degeneration of central nervous system	1.80E-06	63
Liver	Corn stover	Positive	cell death of hematopoietic progenitor cells	1.85E-06	102
Liver	Corn stover	Positive	cell movement of fibroblasts	1.85E-06	84
Liver	Corn stover	Positive	export of molecule	1.86E-06	129
Liver	Corn stover	Positive	aggregation of cells	1.91E-06	141
Liver	Corn stover	Positive	cell movement of muscle cells	1.92E-06	83
Liver	Corn stover	Positive	growth of plasma membrane projections	1.93E-06	197
Liver	Corn	Positive	function of macrophages	1.99E-06	82

	stover				
Liver	Corn stover	Positive	Hypoplasia	2.03E-06	212
Liver	Corn stover	Positive	quantity of protein in blood	2.14E-06	213
Liver	Corn stover	Positive	formation of connective tissue cells	2.18E-06	95
Liver	Corn stover	Positive	proliferation of breast cancer cell lines	2.26E-06	184
Liver	Corn stover	Positive	binding of protein binding site	2.28E-06	126
Liver	Corn stover	Positive	generation of T lymphocytes	2.29E-06	56
Liver	Corn stover	Positive	cell movement of macrophages	2.39E-06	117
Liver	Corn stover	Positive	apoptosis of hepatocytes	2.42E-06	63
Liver	Corn stover	Positive	apoptosis of hematopoietic cells	2.42E-06	97
Liver	Corn stover	Positive	congenital anomaly of skin	2.54E-06	82
Liver	Corn stover	Positive	arrest in proliferation of cells	2.55E-06	96
Liver	Corn stover	Positive	cell death of cardiomyocytes	2.55E-06	96
Liver	Corn stover	Positive	cell death of muscle	2.58E-06	148
Liver	Corn stover	Positive	activation of leukocytes	2.66E-06	287
Liver	Corn stover	Positive	quantity of metal ion	2.70E-06	187
Liver	Corn stover	Positive	abnormal morphology of reproductive system	2.72E-06	213
Liver	Corn stover	Positive	growth of lymphoid organ	2.84E-06	79
Liver	Corn stover	Positive	cell death of sarcoma cell lines	2.92E-06	101
Liver	Corn stover	Positive	apoptosis of cardiomyocytes	2.94E-06	84
Liver	Corn stover	Positive	cell death of central nervous system cells	3.00E-06	127
Liver	Corn stover	Positive	quantity of IgM	3.17E-06	65
Liver	Corn	Positive	formation of actin stress fibers	3.24E-06	106

	stover				
Liver	Corn stover	Positive	necrosis of muscle	3.24E-06	147
Liver	Corn stover	Positive	cell death of B-lymphocyte derived cell lines	3.25E-06	61
Liver	Corn stover	Positive	formation of thymus gland	3.26E-06	75
Liver	Corn stover	Positive	morphogenesis of neurites	3.26E-06	177
Liver	Corn stover	Positive	activation of blood cells	3.29E-06	305
Liver	Corn stover	Positive	urogenital cancer	3.44E-06	1665
Liver	Corn stover	Positive	phosphorylation of L-amino acid	3.45E-06	98
Liver	Corn stover	Positive	intermediate disease stage peripheral arterial disease	3.45E-06	73
Liver	Corn stover	Positive	necrosis of cardiac muscle	3.65E-06	97
Liver	Corn stover	Positive	transformation of fibroblast cell lines	3.68E-06	113
Liver	Corn stover	Positive	migration of smooth muscle cells	3.86E-06	69
Liver	Corn stover	Positive	differentiation of T lymphocytes	3.91E-06	161
Liver	Corn stover	Positive	quantity of metal	3.91E-06	208
Liver	Corn stover	Positive	formation of filopodia	4.03E-06	76
Liver	Corn stover	Positive	function of T lymphocytes	4.15E-06	114
Liver	Corn stover	Positive	migration of breast cancer cell lines	4.18E-06	108
Liver	Corn stover	Positive	cell death of tumor cells	4.19E-06	158
Liver	Corn stover	Positive	mass of organism	4.28E-06	143
Liver	Corn stover	Positive	female genital tract serous carcinoma	4.29E-06	90
Liver	Corn stover	Positive	cell movement of smooth muscle cells	4.30E-06	74
Liver	Corn stover	Positive	accumulation of lipid	4.30E-06	129
Liver	Corn	Positive	long-term potentiation	4.42E-06	107

	stover				
Liver	Corn stover	Positive	cellular infiltration by macrophages	4.43E-06	73
Liver	Corn stover	Positive	cell death of brain	4.46E-06	126
Liver	Corn stover	Positive	occlusion of blood vessel	4.47E-06	201
Liver	Corn stover	Positive	proliferation of embryonic cells	4.70E-06	109
Liver	Corn stover	Positive	cell death of hepatocytes	4.70E-06	71
Liver	Corn stover	Positive	quantity of antigen presenting cells	4.72E-06	120
Liver	Corn stover	Positive	apoptosis of hematopoietic progenitor cells	4.74E-06	92
Liver	Corn stover	Positive	cell movement of breast cancer cell lines	4.74E-06	125
Liver	Corn stover	Negative	proliferation of cells	1.09E-43	1322
Liver	Corn stover	Negative	cell death	7.74E-37	1187
Liver	Corn stover	Negative	morbidity or mortality	1.22E-36	910
Liver	Corn stover	Negative	organismal death	1.57E-36	899
Liver	Corn stover	Negative	necrosis	3.28E-36	952
Liver	Corn stover	Negative	apoptosis	3.45E-32	957
Liver	Corn stover	Negative	morphology of cells	2.08E-29	731
Liver	Corn stover	Negative	malignant solid tumor	3.52E-29	3184
Liver	Corn stover	Negative	cancer	3.78E-29	3218
Liver	Corn stover	Negative	cell movement	3.77E-24	765
Liver	Corn stover	Negative	cell death of tumor cell lines	3.66E-23	574
Liver	Corn stover	Negative	differentiation of cells	4.16E-22	793
Liver	Corn stover	Negative	organization of cytoplasm	9.64E-22	570
Liver	Corn	Negative	neoplasia of epithelial tissue	2.33E-21	2656

	stover				
Liver	Corn stover	Negative	tumorigenesis of tissue	4.41E-21	2694
Liver	Corn stover	Negative	cell survival	4.58E-21	523
Liver	Corn stover	Negative	epithelial cancer	3.27E-20	2626
Liver	Corn stover	Negative	cell viability	3.43E-20	490
Liver	Corn stover	Negative	quantity of cells	3.59E-20	636
Liver	Corn stover	Negative	apoptosis of tumor cell lines	4.38E-20	461
Liver	Corn stover	Negative	abdominal neoplasm	1.48E-19	2626
Liver	Corn stover	Negative	organization of cytoskeleton	1.48E-19	517
Liver	Corn stover	Negative	migration of cells	6.57E-19	671
Liver	Corn stover	Negative	abdominal cancer	1.33E-18	2592
Liver	Corn stover	Negative	expression of RNA	2.15E-18	742
Liver	Corn stover	Negative	transport of molecule	4.02E-18	578
Liver	Corn stover	Negative	transcription	5.72E-18	694
Liver	Corn stover	Negative	transcription of RNA	2.78E-17	649
Liver	Corn stover	Negative	quantity of leukocytes	7.58E-17	353
Liver	Corn stover	Negative	microtubule dynamics	1.16E-16	438
Liver	Corn stover	Negative	angiogenesis	1.35E-16	349
Liver	Corn stover	Negative	quantity of blood cells	2.44E-16	387
Liver	Corn stover	Negative	cell death of connective tissue cells	2.68E-16	246
Liver	Corn stover	Negative	development of cytoplasm	5.49E-16	196
Liver	Corn stover	Negative	digestive organ tumor	6.71E-16	2270
Liver	Corn	Negative	abnormal morphology of cells	6.71E-16	460

	stover				
Liver	Corn stover	Negative	cellular homeostasis	1.28E-15	538
Liver	Corn stover	Negative	digestive system cancer	1.44E-15	2248
Liver	Corn stover	Negative	morphology of cardiovascular system	3.57E-15	248
Liver	Corn stover	Negative	formation of cytoskeleton	9.08E-15	162
Liver	Corn stover	Negative	vasculogenesis	1.63E-14	285
Liver	Corn stover	Negative	Viral Infection	1.70E-14	560
Liver	Corn stover	Negative	proliferation of tumor cell lines	5.14E-14	538
Liver	Corn stover	Negative	fibrogenesis	7.11E-14	172
Liver	Corn stover	Negative	morphology of body cavity	1.04E-13	428
Liver	Corn stover	Negative	transcription of DNA	1.30E-13	532
Liver	Corn stover	Negative	growth of organism	1.31E-13	314
Liver	Corn stover	Negative	protein kinase cascade	2.26E-13	174
Liver	Corn stover	Negative	development of body trunk	2.34E-13	402
Liver	Corn stover	Negative	formation of cellular protrusions	2.84E-13	334
Liver	Corn stover	Negative	abnormal morphology of body cavity	5.85E-13	408
Liver	Corn stover	Negative	transactivation	7.09E-13	226
Liver	Corn stover	Negative	development of epithelial tissue	8.19E-13	205
Liver	Corn stover	Negative	formation of filaments	1.89E-12	163
Liver	Corn stover	Negative	cell death of fibroblast cell lines	2.13E-12	173
Liver	Corn stover	Negative	Growth Failure	2.28E-12	231
Liver	Corn stover	Negative	size of body	2.97E-12	313
Liver	Corn	Negative	quantity of lymphocytes	3.99E-12	266

	stover				
Liver	Corn stover	Negative	quantity of mononuclear leukocytes	4.28E-12	275
Liver	Corn stover	Negative	abnormal morphology of cardiovascular system	6.41E-12	219
Liver	Corn stover	Negative	necrosis of epithelial tissue	6.50E-12	226
Liver	Corn stover	Negative	cell movement of leukocytes	8.36E-12	284
Liver	Corn stover	Negative	migration of blood cells	8.88E-12	318
Liver	Corn stover	Negative	development of vasculature	8.92E-12	168
Liver	Corn stover	Negative	cell movement of tumor cell lines	8.99E-12	311
Liver	Corn stover	Negative	proliferation of blood cells	9.32E-12	308
Liver	Corn stover	Negative	transactivation of RNA	1.11E-11	210
Liver	Corn stover	Negative	leukocyte migration	1.11E-11	317
Liver	Corn stover	Negative	cell death of epithelial cells	1.32E-11	194
Liver	Corn stover	Negative	abnormal morphology of thoracic cavity	1.32E-11	224
Liver	Corn stover	Negative	behavior	1.92E-11	353
Liver	Corn stover	Negative	growth of epithelial tissue	2.06E-11	254
Liver	Corn stover	Negative	homing	4.07E-11	218
Liver	Corn stover	Negative	homing of cells	6.03E-11	212
Liver	Corn stover	Negative	growth of embryo	6.22E-11	185
Liver	Corn stover	Negative	apoptosis of fibroblast cell lines	7.45E-11	134
Liver	Corn stover	Negative	function of blood cells	8.23E-11	221
Liver	Corn stover	Negative	morphology of connective tissue	8.48E-11	179
Liver	Corn stover	Negative	Movement Disorders	1.01E-10	371
Liver	Corn	Negative	proliferation of immune cells	1.14E-10	287

	stover				
Liver	Corn stover	Negative	formation of actin filaments	1.47E-10	125
Liver	Corn stover	Negative	cell death of fibroblasts	1.53E-10	111
Liver	Corn stover	Negative	cell viability of tumor cell lines	1.65E-10	282
Liver	Corn stover	Negative	disorder of basal ganglia	1.82E-10	278
Liver	Corn stover	Negative	organization of organelle	1.82E-10	204
Liver	Corn stover	Negative	proliferation of connective tissue cells	2.69E-10	221
Liver	Corn stover	Negative	cell cycle progression	2.77E-10	365
Liver	Corn stover	Negative	development of cardiovascular tissue	3.76E-10	146
Liver	Corn stover	Negative	survival of organism	4.08E-10	256
Liver	Corn stover	Negative	morphology of heart	4.27E-10	158
Liver	Corn stover	Negative	morphogenesis of neurons	6.10E-10	164
Liver	Corn stover	Negative	proliferation of neuronal cells	6.19E-10	214
Liver	Corn stover	Negative	long-term potentiation	6.23E-10	102
Liver	Corn stover	Negative	activation of DNA endogenous promoter	6.23E-10	413
Liver	Corn stover	Negative	invasion of cells	6.30E-10	308
Liver	Corn stover	Negative	neuromuscular disease	6.79E-10	312
Liver	Corn stover	Negative	neuritogenesis	7.00E-10	216
Liver	Corn stover	Negative	infection of cells	7.00E-10	285
Liver	Corn stover	Negative	metabolism of protein	7.09E-10	340
Liver	Corn stover	Negative	proliferation of mononuclear leukocytes	7.15E-10	266
Liver	Corn stover	Negative	quantity of T lymphocytes	8.09E-10	200
Liver	Corn	Negative	proliferation of lymphocytes	8.81E-10	262

	stover				
Liver	Corn stover	Negative	degeneration of cells	9.27E-10	136
Liver	Corn stover	Negative	Neurodegeneration	9.45E-10	124
Liver	Corn stover	Negative	function of leukocytes	1.20E-09	200
Liver	Corn stover	Negative	development of endothelial tissue	1.20E-09	143
Liver	Corn stover	Negative	formation of plasma membrane projections	1.44E-09	219
Liver	Corn stover	Negative	growth of connective tissue	1.87E-09	234
Liver	Corn stover	Negative	cell movement of myeloid cells	2.06E-09	200
Liver	Corn stover	Negative	differentiation of connective tissue cells	2.50E-09	233
Liver	Corn stover	Negative	formation of actin stress fibers	2.50E-09	99
Liver	Corn stover	Negative	growth of muscle tissue	3.29E-09	144
Liver	Corn stover	Negative	proliferation of endothelial cells	3.58E-09	123
Liver	Corn stover	Negative	proliferation of muscle cells	3.94E-09	143
Liver	Corn stover	Negative	infection by RNA virus	4.02E-09	303
Liver	Corn stover	Negative	development of lymphatic system	4.29E-09	138
Liver	Corn stover	Negative	development of neurons	4.96E-09	276
Liver	Corn stover	Negative	maturation of cells	4.96E-09	171
Liver	Corn stover	Negative	breast or colorectal cancer	4.96E-09	1501
Liver	Corn stover	Negative	chemotaxis	4.99E-09	200
Liver	Corn stover	Negative	phosphorylation of protein	5.25E-09	261
Liver	Corn stover	Negative	proliferation of fibroblast cell lines	5.25E-09	160
Liver	Corn stover	Negative	cognition	5.77E-09	170
Liver	Corn	Negative	morphology of nervous system	5.85E-09	276

	stover				
Liver	Corn stover	Negative	morphogenesis of neurites	6.45E-09	158
Liver	Corn stover	Negative	ubiquitination of protein	6.60E-09	129
Liver	Corn stover	Negative	endothelial cell development	8.37E-09	136
Liver	Corn stover	Negative	cell death of cervical cancer cell lines	8.46E-09	135
Liver	Corn stover	Negative	development of lymphatic system component	8.46E-09	121
Liver	Corn stover	Negative	differentiation of connective tissue	9.27E-09	258
Liver	Corn stover	Negative	chemotaxis of cells	9.73E-09	193
Liver	Corn stover	Negative	ubiquitination	1.08E-08	130
Liver	Corn stover	Negative	cell death of blood cells	1.15E-08	246
Liver	Corn stover	Negative	quantity of connective tissue	1.19E-08	217
Liver	Corn stover	Negative	growth of embryonic tissue	1.21E-08	109
Liver	Corn stover	Negative	degeneration of nervous system	1.22E-08	113
Liver	Corn stover	Negative	neuronal cell death	1.24E-08	242
Liver	Corn stover	Negative	migration of tumor cell lines	1.24E-08	250
Liver	Corn stover	Negative	Lymphocyte homeostasis	1.42E-08	198
Liver	Corn stover	Negative	size of embryo	1.54E-08	136
Liver	Corn stover	Negative	homeostasis of leukocytes	1.56E-08	201
Liver	Corn stover	Negative	quantity of antigen presenting cells	1.74E-08	109
Liver	Corn stover	Negative	learning	1.84E-08	156
Liver	Corn stover	Negative	seizure disorder	1.94E-08	173
Liver	Corn stover	Negative	formation of skin	1.94E-08	139
Liver	Corn	Negative	formation of cells	1.94E-08	335

	stover				
Liver	Corn stover	Negative	cellular degradation	2.39E-08	101
Liver	Corn stover	Negative	synthesis of lipid	2.43E-08	251
Liver	Corn stover	Negative	neurological signs	2.43E-08	231
Liver	Corn stover	Negative	apoptosis of connective tissue cells	2.43E-08	115
Liver	Corn stover	Negative	dyskinesia	2.43E-08	220
Liver	Corn stover	Negative	abnormal morphology of heart	2.63E-08	144
Liver	Corn stover	Negative	concentration of lipid	2.72E-08	281
Liver	Corn stover	Negative	proliferation of embryonic cells	2.75E-08	99
Liver	Corn stover	Negative	gastrointestinal tract cancer	3.07E-08	1518
Liver	Corn stover	Negative	Huntington's Disease	3.07E-08	206
Liver	Corn stover	Negative	cell death of immune cells	3.29E-08	233
Liver	Corn stover	Negative	cell spreading	3.51E-08	108
Liver	Corn stover	Negative	perinatal death	3.59E-08	212
Liver	Corn stover	Negative	differentiation of tumor cell lines	3.69E-08	142
Liver	Corn stover	Negative	abnormal morphology of embryonic tissue	3.70E-08	197
Liver	Corn stover	Negative	Gastrointestinal Tract Cancer and Tumors	3.92E-08	1540
Liver	Corn stover	Negative	cell movement of mononuclear leukocytes	4.23E-08	169
Liver	Corn stover	Negative	morphology of lymphatic system component	4.39E-08	154
Liver	Corn stover	Negative	colony formation	4.45E-08	193
Liver	Corn stover	Negative	differentiation of blood cells	4.45E-08	290
Liver	Corn stover	Negative	T cell development	4.49E-08	182
Liver	Corn	Negative	degeneration of neurons	4.51E-08	95

	stover				
Liver	Corn stover	Negative	sprouting	4.51E-08	144
Liver	Corn stover	Negative	development of blood cells	4.57E-08	230
Liver	Corn stover	Negative	differentiation of epithelial tissue	5.05E-08	135
Liver	Corn stover	Negative	differentiation of leukocytes	5.05E-08	236
Liver	Corn stover	Negative	neonatal death	5.67E-08	161
Liver	Corn stover	Negative	function of cardiovascular system	6.27E-08	138
Liver	Corn stover	Negative	formation of muscle	6.27E-08	147
Liver	Corn stover	Negative	development of leukocytes	6.27E-08	209
Liver	Corn stover	Negative	morphology of head	6.42E-08	299
Liver	Corn stover	Negative	proliferation of smooth muscle cells	6.76E-08	110
Liver	Corn stover	Negative	abnormal morphology of abdomen	7.00E-08	299
Liver	Corn stover	Negative	infection by Retroviridae	7.17E-08	250
Liver	Corn stover	Negative	interphase	7.63E-08	230
Liver	Corn stover	Negative	function of lymphocytes	8.11E-08	128
Liver	Corn stover	Negative	apoptosis of fibroblasts	8.43E-08	87
Liver	Corn stover	Negative	proliferation of fibroblasts	8.46E-08	132
Liver	Corn stover	Negative	T cell homeostasis	8.88E-08	185
Liver	Corn stover	Negative	accumulation of lipid	8.97E-08	114
Liver	Corn stover	Negative	seizures	9.34E-08	147
Liver	Corn stover	Negative	function of muscle	1.04E-07	130
Liver	Corn stover	Negative	quantity of phagocytes	1.04E-07	155
Liver	Corn	Negative	cellular infiltration by leukocytes	1.07E-07	143

	stover				
Liver	Corn stover	Negative	branching of cells	1.13E-07	137
Liver	Corn stover	Negative	binding of DNA	1.20E-07	186
Liver	Corn stover	Negative	mitosis	1.22E-07	178
Liver	Corn stover	Negative	abnormal morphology of head	1.22E-07	284
Liver	Corn stover	Negative	cell movement of phagocytes	1.22E-07	195
Liver	Corn stover	Negative	quantity of macrophages	1.30E-07	81
Liver	Corn stover	Negative	apoptosis of epithelial cells	1.32E-07	109
Liver	Corn stover	Negative	cell death of epithelial cell lines	1.33E-07	108
Liver	Corn stover	Negative	size of animal	1.41E-07	73
Liver	Corn stover	Negative	development of lymphocytes	1.44E-07	196
Liver	Corn stover	Negative	development of connective tissue	1.48E-07	137
Liver	Corn stover	Negative	abnormal morphology of nervous system	1.50E-07	250
Liver	Corn stover	Negative	colony formation of cells	1.67E-07	176
Liver	Corn stover	Negative	development of mononuclear leukocytes	1.71E-07	197
Liver	Corn stover	Negative	Hypertrophy	1.71E-07	170
Liver	Corn stover	Negative	morphology of muscle	1.81E-07	125
Liver	Corn stover	Negative	abnormal morphology of epithelial tissue	1.95E-07	152
Liver	Corn stover	Negative	cell movement of macrophages	1.95E-07	102
Liver	Corn stover	Negative	infection by lentivirus	2.06E-07	246
Liver	Corn stover	Negative	cellular infiltration	2.24E-07	158
Liver	Corn stover	Negative	quantity of B lymphocytes	2.24E-07	128
Liver	Corn	Negative	HIV infection	2.39E-07	245

	stover				
Liver	Corn stover	Negative	proliferation of T lymphocytes	2.45E-07	211
Liver	Corn stover	Negative	differentiation of bone	2.48E-07	149
Liver	Corn stover	Negative	formation of lymphatic system component	2.97E-07	110
Liver	Corn stover	Negative	MAPKKK cascade	2.97E-07	81
Liver	Corn stover	Negative	activation of cells	3.22E-07	326
Liver	Corn stover	Negative	cell movement of endothelial cells	3.28E-07	130
Liver	Corn stover	Negative	apoptosis of pheochromocytoma cell lines	3.41E-07	42
Liver	Corn stover	Negative	cellular infiltration of cells	3.46E-07	144
Liver	Corn stover	Negative	differentiation of bone cells	4.05E-07	147
Liver	Corn stover	Negative	hepatocellular carcinoma	4.24E-07	1258
Liver	Corn stover	Negative	proliferation of hematopoietic cells	4.96E-07	97
Liver	Corn stover	Negative	proliferation of hematopoietic progenitor cells	5.03E-07	93
Liver	Corn stover	Negative	growth of lymphatic system component	6.14E-07	76
Liver	Corn stover	Negative	invasion of tumor cell lines	6.92E-07	228
Liver	Corn stover	Negative	benign neoplasia	7.27E-07	308
Liver	Corn stover	Negative	engulfment of cells	7.27E-07	146
Liver	Corn stover	Negative	apoptosis of blood cells	8.71E-07	174
Liver	Corn stover	Negative	infection by HIV-1	9.28E-07	210
Liver	Corn stover	Negative	quantity of carbohydrate	9.62E-07	181
Liver	Corn stover	Negative	quantity of lymphatic system component	9.62E-07	136
Liver	Corn stover	Negative	colon tumor	1.02E-06	1122
Liver	Corn	Negative	growth of neurites	1.04E-06	163

	stover				
Liver	Corn stover	Negative	cellular infiltration by macrophages	1.14E-06	64
Liver	Corn stover	Negative	formation of lymphoid organ	1.17E-06	99
Liver	Corn stover	Negative	potentiation of synapse	1.19E-06	61
Liver	Corn stover	Negative	adenocarcinoma	1.20E-06	1905
Liver	Corn stover	Negative	growth of plasma membrane projections	1.25E-06	164
Liver	Corn stover	Negative	cell transformation	1.38E-06	162
Liver	Corn stover	Negative	gastrointestinal carcinoma	1.39E-06	1350
Liver	Corn stover	Negative	differentiation of osteoblasts	1.51E-06	103
Liver	Corn stover	Negative	intestinal tumor	1.56E-06	1301
Liver	Corn stover	Negative	development of digestive system	1.56E-06	137
Liver	Corn stover	Negative	colorectal neoplasia	1.61E-06	1297
Liver	Corn stover	Negative	tumorigenesis of intestine	1.62E-06	1119
Liver	Corn stover	Negative	differentiation of mononuclear leukocytes	1.66E-06	187
Liver	Corn stover	Negative	Organ Degeneration	1.66E-06	172
Liver	Corn stover	Negative	inflammatory response	1.66E-06	261
Liver	Corn stover	Negative	apoptosis of cervical cancer cell lines	1.70E-06	104
Liver	Corn stover	Negative	autophagy	1.73E-06	134
Liver	Corn stover	Negative	colon cancer	1.75E-06	1113
Liver	Corn stover	Negative	long-term potentiation of synapse	1.76E-06	60
Liver	Corn stover	Negative	neoplasia of colon	1.76E-06	1116
Liver	Corn stover	Negative	morphology of vessel	1.85E-06	112
Liver	Corn	Negative	abdominal adenocarcinoma	1.93E-06	1736

	stover				
Liver	Corn stover	Negative	formation of lung	1.96E-06	110
Liver	Corn stover	Negative	morphology of cardiovascular tissue	2.01E-06	39
Liver	Corn stover	Negative	cell death of tumor	2.04E-06	136
Liver	Corn stover	Negative	outgrowth of plasma membrane projections	2.12E-06	144
Liver	Corn stover	Negative	cell movement of smooth muscle cells	2.18E-06	64
Liver	Corn stover	Negative	outgrowth of neurites	2.21E-06	143
Liver	Corn stover	Negative	morphology of lymphoid organ	2.32E-06	139
Liver	Corn stover	Negative	proliferation of epithelial cells	2.37E-06	171
Liver	Corn stover	Negative	colorectal cancer	2.44E-06	1278
Liver	Corn stover	Negative	formation of leukocytes	2.61E-06	63
Liver	Corn stover	Negative	cell death of pheochromocytoma cell lines	2.61E-06	50
Liver	Corn stover	Negative	necrosis of tumor	2.74E-06	135
Liver	Corn stover	Negative	cell movement of fibroblasts	2.76E-06	71
Liver	Corn stover	Negative	mass of organism	2.89E-06	120
Liver	Corn stover	Negative	intestinal cancer	3.03E-06	1279
Liver	Corn stover	Negative	liver lesion	3.09E-06	1296
Liver	Corn stover	Negative	catabolism of protein	3.09E-06	205
Liver	Corn stover	Negative	cell death of mononuclear leukocytes	3.20E-06	140
Liver	Corn stover	Negative	abnormal morphology of lymphoid organ	3.21E-06	134
Liver	Corn stover	Negative	liver tumor	3.21E-06	1295
Liver	Corn stover	Negative	cell movement of muscle cells	3.27E-06	70
Liver	Corn	Negative	apoptosis of leukocytes	3.38E-06	159

	stover				
Liver	Corn stover	Negative	congenital anomaly of musculoskeletal system	3.38E-06	246
Liver	Corn stover	Negative	hepatobiliary system cancer	3.40E-06	1292
Liver	Corn stover	Negative	vascularization	3.48E-06	88
Liver	Corn stover	Negative	morphology of endothelial tissue	3.50E-06	37
Liver	Corn stover	Negative	differentiation of epithelial cells	3.59E-06	113
Liver	Corn stover	Negative	cell movement of lymphocytes	3.71E-06	139
Liver	Corn stover	Negative	liver cancer	3.71E-06	1286
Liver	Corn stover	Negative	cell death of lymphocytes	3.72E-06	136
Liver	Corn stover	Negative	respiratory system development	3.93E-06	122
Liver	Corn stover	Negative	branching of neurons	4.05E-06	104
Liver	Corn stover	Negative	metabolism of carbohydrate	4.05E-06	210
Liver	Corn stover	Negative	migration of smooth muscle cells	4.06E-06	59
Liver	Corn stover	Negative	transport of carbohydrate	4.17E-06	74
Liver	Corn stover	Negative	quantity of interleukin	4.28E-06	56
Liver	Corn stover	Negative	branching of neurites	4.35E-06	101
Liver	Corn stover	Negative	accumulation of cells	4.50E-06	125
Liver	Corn stover	Negative	arthropathy	4.50E-06	311
Liver	Corn stover	Negative	cell movement of antigen presenting cells	4.68E-06	124
Liver	Corn stover	Negative	cardiogenesis	4.69E-06	163
Liver	Corn stover	Negative	arthritis	4.75E-06	306
Liver	Corn stover	Negative	dysgenesis	5.05E-06	184
Liver	Corn	Negative	morphology of blood cells	5.05E-06	145

	stover				
Liver	Corn stover	Negative	fatty acid metabolism	5.33E-06	203
Liver	Corn stover	Negative	uptake of monosaccharide	5.42E-06	98
Liver	Corn stover	Negative	hypertrophy of heart	5.42E-06	120
Liver	Corn stover	Negative	I-kappaB kinase/NF-kappaB cascade	5.42E-06	67
Liver	Corn stover	Negative	development of head	5.46E-06	319
Liver	Corn stover	Negative	cellular infiltration of phagocytes	5.52E-06	68
Liver	Corn stover	Negative	advanced malignant tumor	5.77E-06	278
Liver	Corn stover	Negative	cell death of cerebral cortex cells	5.78E-06	85
Liver	Corn stover	Negative	differentiation of muscle cell lines	5.90E-06	63
Liver	Corn stover	Negative	activation of enzyme	5.90E-06	136
Liver	Corn stover	Negative	cell death of brain	5.92E-06	105
Liver	Corn stover	Negative	abnormal morphology of muscle	6.03E-06	113
Liver	Corn stover	Negative	migration of muscle cells	6.05E-06	64
Liver	Corn stover	Negative	Ovarian Cancer and Tumors	6.10E-06	251
Liver	Corn stover	Negative	synthesis of nitric oxide	6.86E-06	100
Liver	Corn stover	Negative	apoptosis of epithelial cell lines	6.96E-06	83
Liver	Corn stover	Negative	apoptosis of hepatocytes	6.99E-06	53
Liver	Corn stover	Negative	activation of Protein kinase	7.10E-06	92
Liver	Corn stover	Negative	morphology of central nervous system	7.10E-06	175
Liver	Corn stover	Negative	morphology of respiratory system	7.40E-06	111
Liver	Corn stover	Negative	apoptosis of muscle	7.76E-06	95
Liver	Corn	Negative	quantity of protein in blood	7.89E-06	173

	stover				
Liver	Corn stover	Negative	movement of vascular endothelial cells	8.26E-06	69
Liver	Corn stover	Negative	recruitment of antigen presenting cells	8.35E-06	49
Liver	Corn stover	Negative	cell death of tumor cells	8.62E-06	130
Liver	Corn stover	Negative	development of gastrointestinal tract	9.05E-06	95
Liver	Corn stover	Negative	synthesis of reactive oxygen species	9.19E-06	164
Liver	Corn stover	Negative	metabolism of nucleotide	9.19E-06	151
Liver	Corn stover	Negative	migration of endothelial cells	9.19E-06	116
Liver	Corn stover	Negative	cell death of muscle	9.80E-06	121
Liver	Corn stover	Negative	metabolism of nucleic acid component or derivative	1.01E-05	176
Liver	Corn stover	Negative	formation of blood cells	1.03E-05	74
Liver	Corn stover	Negative	abnormal morphology of respiratory system	1.04E-05	109
Liver	Corn stover	Negative	apoptosis of muscle cells	1.07E-05	94
Liver	Corn stover	Negative	differentiation of epidermal cells	1.09E-05	63
Liver	Corn stover	Negative	cell death of brain cells	1.12E-05	98
Liver	Corn stover	Negative	morphology of digestive system	1.13E-05	192
Liver	Corn stover	Negative	breast or ovarian cancer	1.14E-05	590
Liver	Corn stover	Negative	uptake of D-hexose	1.15E-05	82
Liver	Corn stover	Negative	differentiation of lymphocytes	1.18E-05	169
Liver	Corn stover	Negative	apoptosis of heart cells	1.22E-05	71
Liver	Corn stover	Negative	invasion of tissue	1.22E-05	73
Liver	Corn stover	Negative	cell death of central nervous system cells	1.22E-05	104
Liver	Corn	Negative	S phase	1.23E-05	94

	stover				
Liver	Corn stover	Negative	apoptosis of liver	1.26E-05	61
Liver	Corn stover	Negative	cell death of breast cancer cell lines	1.29E-05	117
Liver	Corn stover	Negative	necrosis of muscle	1.29E-05	120
Liver	Corn stover	Negative	incidence of tumor	1.32E-05	113
Liver	Corn stover	Negative	differentiation of skin	1.43E-05	68
Liver	Corn stover	Negative	small GTPase mediated signal transduction	1.44E-05	69
Liver	Corn stover	Negative	differentiation of muscle	1.45E-05	107
Liver	Corn stover	Negative	quantity of muscle cells	1.48E-05	47
Liver	Corn stover	Negative	quantity of immunoglobulin	1.56E-05	105
Liver	Corn stover	Negative	angiogenesis of tumor	1.61E-05	48
Liver	Corn stover	Negative	plasticity of synapse	1.61E-05	48
Liver	Corn stover	Negative	cell death of kidney cell lines	1.66E-05	107
Liver	Corn stover	Negative	uptake of carbohydrate	1.66E-05	103
Liver	Corn stover	Negative	differentiation of keratinocytes	1.74E-05	58
Liver	Corn stover	Negative	gonadal tumor	1.74E-05	267
Liver	Corn stover	Negative	production of protein	1.80E-05	115
Liver	Corn stover	Negative	outgrowth of cells	1.80E-05	148
Liver	Corn stover	Negative	cell death of muscle cells	1.88E-05	117
Liver	Corn stover	Negative	cell movement of connective tissue cells	1.89E-05	81
Liver	Corn stover	Negative	abdominal carcinoma	1.90E-05	1747
Liver	Corn stover	Negative	memory	2.03E-05	92
Liver	Corn	Negative	shape change of fibroblast cell lines	2.04E-05	38

	stover				
Liver	Corn stover	Negative	quantity of lymphoid organ	2.07E-05	108
Liver	Corn stover	Negative	cell viability of connective tissue cells	2.08E-05	52
Liver	Corn stover	Negative	recruitment of macrophages	2.20E-05	43
Liver	Corn stover	Negative	hypertrophy of cells	2.26E-05	102
Liver	Corn stover	Negative	cell death of kidney cells	2.29E-05	121
Liver	Corn stover	Negative	cell death of liver	2.35E-05	86
Liver	Corn stover	Negative	apoptosis of lymphocytes	2.39E-05	120
Liver	Corn stover	Negative	morphology of endothelial cells	2.40E-05	28
Liver	Corn stover	Negative	metabolism of reactive oxygen species	2.44E-05	168
Liver	Corn stover	Negative	cell death of liver cells	2.45E-05	72
Liver	Corn stover	Negative	activation of blood cells	2.45E-05	244
Liver	Corn stover	Negative	colorectal carcinoma	2.46E-05	1100
Liver	Corn stover	Negative	autophagy of cells	2.53E-05	94
Liver	Corn stover	Negative	production of antibody	2.57E-05	109
Liver	Corn stover	Negative	uptake of D-glucose	2.63E-05	80
Liver	Corn stover	Negative	development of body axis	2.64E-05	335
Liver	Corn stover	Negative	growth of lesion	2.65E-05	259
Liver	Corn stover	Negative	quantity of IL-6 in blood	2.68E-05	36
Liver	Corn stover	Negative	homing of blood cells	2.70E-05	130
Liver	Corn stover	Negative	quantity of metal ion	2.71E-05	150
Liver	Corn stover	Negative	female genital tract serous cancer	2.75E-05	132
Liver	Corn	Negative	tumorigenesis of gonad	2.79E-05	262

	stover				
Liver	Corn stover	Negative	quantity of epithelial tissue	2.91E-05	66
Liver	Corn stover	Negative	apoptosis of mononuclear leukocytes	2.94E-05	123
Liver	Corn stover	Negative	Bleeding	2.96E-05	144
Liver	Corn stover	Negative	gastrointestinal adenocarcinoma	3.17E-05	1259
Liver	Corn stover	Negative	homing of leukocytes	3.17E-05	129
Liver	Corn stover	Negative	necrosis of liver	3.21E-05	85
Liver	Corn stover	Negative	growth of tumor	3.22E-05	258
Liver	Corn stover	Negative	apoptosis of liver cells	3.23E-05	59
Liver	Corn stover	Negative	necrosis of kidney	3.38E-05	125
Liver	Corn stover	Negative	abnormal morphology of digestive system	3.38E-05	181
Liver	Corn stover	Negative	internalization of cells	3.38E-05	62
Liver	Corn stover	Negative	growth of lymphoid organ	3.44E-05	64
Liver	Corn stover	Negative	apoptosis of cardiomyocytes	3.46E-05	68
Liver	Corn stover	Negative	morphology of neuroglia	3.60E-05	46
Liver	Corn stover	Negative	morphology of blood vessel	3.68E-05	99
Liver	Corn stover	Negative	accumulation of blood cells	3.81E-05	98
Liver	Corn stover	Negative	cell death of hepatocytes	3.86E-05	58
Liver	Corn stover	Negative	engulfment of leukocytes	4.00E-05	60
Liver	Corn stover	Negative	hyperplasia of tissue	4.07E-05	71
Liver	Corn stover	Negative	migration of vascular endothelial cells	4.09E-05	62
Liver	Corn stover	Negative	morphology of leukocytes	4.11E-05	118
Liver	Corn	Negative	cytostasis	4.20E-05	99

	stover				
Liver	Corn stover	Negative	expansion of cells	4.35E-05	105
Liver	Corn stover	Negative	immune response of cells	4.48E-05	189
Liver	Corn stover	Negative	quantity of metal	4.72E-05	166
Liver	Corn stover	Negative	infection of kidney cell lines	4.82E-05	99
Liver	Corn stover	Negative	epileptic seizure	4.95E-05	62
Liver	Corn stover	Negative	engulfment of blood cells	4.97E-05	63
Liver	Corn stover	Negative	abnormal morphology of blood vessel	5.22E-05	93
Liver	Corn stover	Negative	autosomal recessive disease	5.27E-05	294
Liver	Corn stover	Negative	ovarian cancer	5.28E-05	231
Liver	Corn stover	Negative	apoptosis of breast cancer cell lines	5.28E-05	100
Liver	Corn stover	Negative	serous neoplasm	5.32E-05	200
Liver	Corn stover	Negative	angiogenesis of lesion	5.41E-05	49
Liver	Corn stover	Negative	modification of peptide	5.52E-05	42
Liver	Corn stover	Negative	activation of leukocytes	5.61E-05	227
Liver	Corn stover	Negative	formation of mononuclear leukocytes	5.64E-05	50
Liver	Corn stover	Negative	long-term potentiation of brain	5.64E-05	50
Liver	Corn stover	Negative	transport of monosaccharide	5.77E-05	59
Liver	Corn stover	Negative	migration of connective tissue cells	5.90E-05	66
Liver	Corn stover	Negative	infection of embryonic cell lines	6.08E-05	96
Liver	Corn stover	Negative	infection of epithelial cell lines	6.08E-05	96
Liver	Corn stover	Negative	autosomal dominant disease	6.08E-05	208
Liver	Corn	Negative	uterine serous papillary cancer	6.22E-05	82

	stover				
Liver	Corn stover	Negative	glandular intraepithelial neoplasm	6.42E-05	48
Liver	Corn stover	Negative	metastasis	6.44E-05	245
Liver	Corn stover	Negative	tumorigenesis of malignant tumor	6.52E-05	94
Liver	Corn stover	Negative	morphology of skeleton	6.68E-05	97
Liver	Corn stover	Negative	development of connective tissue cells	6.69E-05	79
Liver	Corn stover	Negative	hypertrophy of heart cells	6.88E-05	58
Liver	Corn stover	Negative	formation of thymus gland	7.00E-05	60
Liver	Corn stover	Negative	Hypoplasia	7.02E-05	167
Liver	Corn stover	Negative	cell death of T lymphocytes	7.08E-05	108
Liver	Corn stover	Negative	dyspnea	7.17E-05	51
Liver	Corn stover	Negative	function of T lymphocytes	7.17E-05	91
Liver	Corn stover	Negative	prostatic intraepithelial neoplasia	7.17E-05	46
Liver	Corn stover	Negative	synthesis of nucleotide	7.17E-05	123
Liver	Corn stover	Negative	ploidy of cells	7.38E-05	52
Liver	Corn stover	Negative	synaptic transmission	7.38E-05	115
Liver	Corn stover	Negative	wasting	7.55E-05	40
Liver	Corn stover	Negative	Edema	7.55E-05	117
Liver	Corn stover	Negative	proliferation of lymphatic system cells	7.61E-05	72
Liver	Corn stover	Negative	cell death of heart cells	7.87E-05	78
Liver	Corn stover	Negative	atrophy of muscle	7.87E-05	55
Liver	Corn stover	Negative	metabolism of glycosphingolipid	7.87E-05	48
Liver	Corn	Negative	shape change of tumor cell lines	8.11E-05	57

	stover				
Liver	Corn stover	Negative	cell death of skin	8.20E-05	41
Liver	Corn stover	Negative	abnormal morphology of central nervous system	8.32E-05	160
Liver	Corn stover	Negative	abnormal morphology of skeleton	8.35E-05	94
Liver	Corn stover	Negative	phagocytosis of cells	8.35E-05	94
Liver	Corn stover	Negative	Wound	8.64E-05	80
Liver	Corn stover	Negative	cell death of sarcoma cell lines	8.64E-05	80
Liver	Corn stover	Negative	vascularization of body region	8.73E-05	51
Liver	Corn stover	Negative	neurotransmission	8.86E-05	138
Liver	Corn stover	Negative	weight loss	8.88E-05	84
Liver	Corn stover	Negative	hypersensitive reaction	9.30E-05	143
Liver	Corn stover	Negative	morphology of heart ventricle	9.30E-05	69
Liver	Corn stover	Negative	function of smooth muscle	9.33E-05	37
Liver	Corn stover	Negative	proliferation of vascular endothelial cells	9.33E-05	54
Liver	Corn stover	Negative	differentiation of muscle cells	9.61E-05	97
Liver	Corn stover	Negative	abnormal morphology of lymph node	9.61E-05	56
Liver	Corn stover	Negative	peripheral arterial disease	9.70E-05	75
Liver	Corn stover	Negative	morphology of lymph node	9.82E-05	59
Liver	Corn stover	Negative	hypertrophy of tissue	9.88E-05	89
Liver	Corn stover	Negative	cell death of heart	9.88E-05	80
Liver	Corn stover	Negative	differentiation of embryonic cells	9.88E-05	80
Liver	Corn stover	Negative	formation of vascular lesion	9.92E-05	44
Liver	Corn	Negative	quantity of muscle	9.96E-05	49

	stover				
Liver	Corn stover	Negative	development of abdomen	9.99E-05	189
Liver	Corn stover	Negative	size of cells	1.01E-04	143
Liver	Corn stover	Negative	damage of nervous system	1.01E-04	79
Liver	Corn stover	Negative	synthesis of DNA	1.01E-04	139
Liver	Corn stover	Negative	morphology of brain	1.01E-04	154
Liver	Corn stover	Negative	gliosis	1.02E-04	50
Liver	Corn stover	Negative	Encephalitis	1.03E-04	101
Liver	Corn stover	Negative	export of molecule	1.03E-04	101
Liver	Corn stover	Negative	formation of embryonic tissue	1.03E-04	101
Liver	Corn stover	Negative	synthesis of ceramide	1.07E-04	30
Liver	Corn stover	Negative	morphology of bone	1.08E-04	149
Liver	Corn stover	Negative	concentration of fatty acid	1.11E-04	96
Liver	Corn stover	Negative	differentiation of phagocytes	1.11E-04	85
Liver	Corn stover	Negative	secretion of molecule	1.15E-04	180
Liver	Corn stover	Negative	cell surface receptor linked signal transduction	1.17E-04	101
Liver	Corn stover	Negative	inflammation of central nervous system	1.21E-04	103
Liver	Corn stover	Negative	development of epithelial cells	1.22E-04	49
Liver	Corn stover	Negative	Fibrosis	1.23E-04	174
Liver	Corn stover	Negative	cell death of leukemia cell lines	1.23E-04	108
Liver	Corn stover	Negative	cachexia	1.25E-04	35
Liver	Corn stover	Negative	migration of mononuclear leukocytes	1.26E-04	124
Liver	Corn	Negative	transformation of fibroblast cell lines	1.27E-04	89

	stover				
Liver	Corn stover	Negative	apoptosis of carcinoma cell lines	1.27E-04	85
Liver	Corn stover	Negative	Lymphocyte migration	1.34E-04	116
Mammary gland	Alfalfa hay	Positive	proliferation of cells	9.69E-71	1966
Mammary gland	Alfalfa hay	Positive	cancer	8.33E-59	4871
Mammary gland	Alfalfa hay	Positive	malignant solid tumor	2.90E-57	4814
Mammary gland	Alfalfa hay	Positive	cell death	4.59E-52	1734
Mammary gland	Alfalfa hay	Positive	organismal death	2.39E-46	1287
Mammary gland	Alfalfa hay	Positive	morbidity or mortality	6.29E-46	1300
Mammary gland	Alfalfa hay	Positive	apoptosis	8.55E-45	1393
Mammary gland	Alfalfa hay	Positive	necrosis	7.00E-43	1353
Mammary gland	Alfalfa hay	Positive	morphology of cells	6.28E-41	1059
Mammary gland	Alfalfa hay	Positive	organization of cytoplasm	1.34E-38	857
Mammary gland	Alfalfa hay	Positive	tumorigenesis of tissue	2.42E-32	4028
Mammary gland	Alfalfa hay	Positive	cell movement	5.43E-32	1106
Mammary gland	Alfalfa hay	Positive	organization of cytoskeleton	1.10E-31	767
Mammary gland	Alfalfa hay	Positive	neoplasia of epithelial tissue	2.17E-31	3962
Mammary gland	Alfalfa hay	Positive	epithelial cancer	6.14E-31	3925
Mammary gland	Alfalfa hay	Positive	abdominal neoplasm	4.35E-29	3921
Mammary gland	Alfalfa hay	Positive	migration of cells	2.66E-27	983
Mammary gland	Alfalfa hay	Positive	abdominal cancer	4.02E-27	3867
Mammary gland	Alfalfa hay	Positive	quantity of cells	6.23E-27	920
Mammary	Alfalfa	Positive	transport of molecule	6.61E-26	844

gland	hay				
Mammary gland	Alfalfa hay	Positive	microtubule dynamics	1.43E-25	644
Mammary gland	Alfalfa hay	Positive	cellular homeostasis	1.43E-25	801
Mammary gland	Alfalfa hay	Positive	cell death of tumor cell lines	2.27E-25	802
Mammary gland	Alfalfa hay	Positive	cell survival	3.12E-25	740
Mammary gland	Alfalfa hay	Positive	differentiation of cells	1.60E-24	1125
Mammary gland	Alfalfa hay	Positive	transcription	2.19E-24	1010
Mammary gland	Alfalfa hay	Positive	abnormal morphology of cells	2.34E-24	677
Mammary gland	Alfalfa hay	Positive	cell viability	4.77E-24	692
Mammary gland	Alfalfa hay	Positive	cell death of connective tissue cells	1.46E-23	354
Mammary gland	Alfalfa hay	Positive	expression of RNA	1.73E-23	1073
Mammary gland	Alfalfa hay	Positive	morphology of cardiovascular system	2.11E-23	362
Mammary gland	Alfalfa hay	Positive	morphology of body cavity	2.33E-23	640
Mammary gland	Alfalfa hay	Positive	abnormal morphology of body cavity	5.04E-23	614
Mammary gland	Alfalfa hay	Positive	angiogenesis	1.22E-22	501
Mammary gland	Alfalfa hay	Positive	transcription of RNA	1.42E-22	939
Mammary gland	Alfalfa hay	Positive	digestive organ tumor	6.83E-22	3374
Mammary gland	Alfalfa hay	Positive	digestive system cancer	1.59E-21	3342
Mammary gland	Alfalfa hay	Positive	proliferation of tumor cell lines	1.80E-21	794
Mammary gland	Alfalfa hay	Positive	apoptosis of tumor cell lines	1.23E-20	636
Mammary gland	Alfalfa hay	Positive	Viral Infection	1.28E-20	819
Mammary gland	Alfalfa hay	Positive	Movement Disorders	2.74E-20	564
Mammary	Alfalfa	Positive	vasculogenesis	2.74E-20	410

gland	hay				
Mammary gland	Alfalfa hay	Positive	concentration of lipid	5.34E-20	445
Mammary gland	Alfalfa hay	Positive	abnormal morphology of thoracic cavity	9.73E-20	334
Mammary gland	Alfalfa hay	Positive	proliferation of connective tissue cells	2.25E-19	336
Mammary gland	Alfalfa hay	Positive	abnormal morphology of cardiovascular system	2.89E-19	323
Mammary gland	Alfalfa hay	Positive	cell cycle progression	9.05E-19	552
Mammary gland	Alfalfa hay	Positive	invasion of cells	1.22E-18	468
Mammary gland	Alfalfa hay	Positive	cell movement of tumor cell lines	1.45E-18	459
Mammary gland	Alfalfa hay	Positive	cell viability of tumor cell lines	1.53E-18	424
Mammary gland	Alfalfa hay	Positive	size of body	2.09E-18	458
Mammary gland	Alfalfa hay	Positive	quantity of blood cells	2.28E-18	541
Mammary gland	Alfalfa hay	Positive	formation of cellular protrusions	2.88E-18	482
Mammary gland	Alfalfa hay	Positive	development of body trunk	7.62E-18	580
Mammary gland	Alfalfa hay	Positive	quantity of leukocytes	1.05E-17	486
Mammary gland	Alfalfa hay	Positive	ubiquitination	1.48E-17	201
Mammary gland	Alfalfa hay	Positive	cell death of fibroblast cell lines	1.60E-17	247
Mammary gland	Alfalfa hay	Positive	growth of connective tissue	2.99E-17	354
Mammary gland	Alfalfa hay	Positive	ubiquitination of protein	5.11E-17	197
Mammary gland	Alfalfa hay	Positive	organization of organelle	8.14E-17	301
Mammary gland	Alfalfa hay	Positive	autosomal recessive disease	1.30E-16	484
Mammary gland	Alfalfa hay	Positive	synthesis of lipid	1.91E-16	385
Mammary gland	Alfalfa hay	Positive	growth of organism	3.54E-16	442
Mammary	Alfalfa	Positive	quantity of connective tissue	6.77E-16	329

gland	hay				
Mammary gland	Alfalfa hay	Positive	migration of tumor cell lines	1.41E-15	378
Mammary gland	Alfalfa hay	Positive	colony formation	1.99E-15	295
Mammary gland	Alfalfa hay	Positive	colony formation of cells	2.03E-15	273
Mammary gland	Alfalfa hay	Positive	phosphorylation of protein	3.81E-15	390
Mammary gland	Alfalfa hay	Positive	apoptosis of fibroblast cell lines	4.81E-15	190
Mammary gland	Alfalfa hay	Positive	invasion of tumor cell lines	4.98E-15	356
Mammary gland	Alfalfa hay	Positive	necrosis of epithelial tissue	4.99E-15	319
Mammary gland	Alfalfa hay	Positive	quantity of lymphocytes	5.29E-15	376
Mammary gland	Alfalfa hay	Positive	quantity of mononuclear leukocytes	6.42E-15	389
Mammary gland	Alfalfa hay	Positive	development of vasculature	1.34E-14	234
Mammary gland	Alfalfa hay	Positive	development of cytoplasm	2.00E-14	255
Mammary gland	Alfalfa hay	Positive	cell death of epithelial cells	2.12E-14	272
Mammary gland	Alfalfa hay	Positive	disorder of basal ganglia	3.30E-14	401
Mammary gland	Alfalfa hay	Positive	abnormal morphology of abdomen	4.20E-14	452
Mammary gland	Alfalfa hay	Positive	function of leukocytes	6.44E-14	290
Mammary gland	Alfalfa hay	Positive	neuromuscular disease	6.67E-14	454
Mammary gland	Alfalfa hay	Positive	neuronal cell death	7.52E-14	359
Mammary gland	Alfalfa hay	Positive	transactivation	1.04E-13	309
Mammary gland	Alfalfa hay	Positive	growth of epithelial tissue	1.61E-13	357
Mammary gland	Alfalfa hay	Positive	growth of muscle tissue	1.83E-13	208
Mammary gland	Alfalfa hay	Positive	morphology of heart	1.85E-13	224
Mammary	Alfalfa	Positive	formation of cells	1.88E-13	498

gland	hay				
Mammary gland	Alfalfa hay	Positive	transcription of DNA	2.28E-13	745
Mammary gland	Alfalfa hay	Positive	proliferation of muscle cells	3.64E-13	206
Mammary gland	Alfalfa hay	Positive	morphology of connective tissue	3.69E-13	250
Mammary gland	Alfalfa hay	Positive	proliferation of smooth muscle cells	5.04E-13	163
Mammary gland	Alfalfa hay	Positive	abdominal adenocarcinoma	5.11E-13	2632
Mammary gland	Alfalfa hay	Positive	benign neoplasia	6.45E-13	469
Mammary gland	Alfalfa hay	Positive	interphase	6.89E-13	343
Mammary gland	Alfalfa hay	Positive	cell transformation	7.03E-13	249
Mammary gland	Alfalfa hay	Positive	adenocarcinoma	8.66E-13	2880
Mammary gland	Alfalfa hay	Positive	cell death of blood cells	9.33E-13	360
Mammary gland	Alfalfa hay	Positive	function of blood cells	1.11E-12	309
Mammary gland	Alfalfa hay	Positive	protein kinase cascade	1.23E-12	229
Mammary gland	Alfalfa hay	Positive	Growth Failure	3.05E-12	313
Mammary gland	Alfalfa hay	Positive	proliferation of neuronal cells	3.54E-12	302
Mammary gland	Alfalfa hay	Positive	activation of cells	4.08E-12	489
Mammary gland	Alfalfa hay	Positive	development of blood cells	4.29E-12	338
Mammary gland	Alfalfa hay	Positive	cell movement of blood cells	6.22E-12	441
Mammary gland	Alfalfa hay	Positive	migration of blood cells	6.26E-12	440
Mammary gland	Alfalfa hay	Positive	development of leukocytes	6.28E-12	307
Mammary gland	Alfalfa hay	Positive	abnormal morphology of heart	6.52E-12	208
Mammary gland	Alfalfa hay	Positive	abnormal morphology of epithelial tissue	7.18E-12	225
Mammary	Alfalfa	Positive	leukocyte migration	7.18E-12	439

gland	hay				
Mammary gland	Alfalfa hay	Positive	metabolism of carbohydrate	7.39E-12	322
Mammary gland	Alfalfa hay	Positive	cell death of immune cells	8.32E-12	340
Mammary gland	Alfalfa hay	Positive	proliferation of blood cells	8.77E-12	425
Mammary gland	Alfalfa hay	Positive	formation of cytoskeleton	8.77E-12	204
Mammary gland	Alfalfa hay	Positive	binding of DNA	8.88E-12	274
Mammary gland	Alfalfa hay	Positive	cell death of T lymphocytes	8.92E-12	174
Mammary gland	Alfalfa hay	Positive	fibrogenesis	9.64E-12	221
Mammary gland	Alfalfa hay	Positive	quantity of T lymphocytes	1.39E-11	280
Mammary gland	Alfalfa hay	Positive	differentiation of connective tissue cells	1.41E-11	331
Mammary gland	Alfalfa hay	Positive	abnormal morphology of embryonic tissue	1.41E-11	286
Mammary gland	Alfalfa hay	Positive	replication of RNA virus	1.47E-11	266
Mammary gland	Alfalfa hay	Positive	synthesis of DNA	1.60E-11	223
Mammary gland	Alfalfa hay	Positive	cell death of epithelial cell lines	1.95E-11	157
Mammary gland	Alfalfa hay	Positive	dyskinesia	2.08E-11	318
Mammary gland	Alfalfa hay	Positive	degeneration of nervous system	2.25E-11	159
Mammary gland	Alfalfa hay	Positive	neurological signs	2.29E-11	334
Mammary gland	Alfalfa hay	Positive	transactivation of RNA	2.42E-11	283
Mammary gland	Alfalfa hay	Positive	cell death of lymphocytes	2.44E-11	207
Mammary gland	Alfalfa hay	Positive	fatty acid metabolism	2.46E-11	310
Mammary gland	Alfalfa hay	Positive	proliferation of fibroblasts	2.73E-11	191
Mammary gland	Alfalfa hay	Positive	genital tumor	2.84E-11	1822
Mammary	Alfalfa	Positive	apoptosis of connective tissue cells	2.88E-11	163

gland	hay				
Mammary	Alfalfa				
gland	hay	Positive	behavior	3.24E-11	489
Mammary	Alfalfa				
gland	hay	Positive	breast or colorectal cancer	3.43E-11	2216
Mammary	Alfalfa				
gland	hay	Positive	Huntington's Disease	3.45E-11	297
Mammary	Alfalfa				
gland	hay	Positive	chorea	3.45E-11	298
Mammary	Alfalfa				
gland	hay	Positive	cell death of mononuclear leukocytes	3.45E-11	212
Mammary	Alfalfa				
gland	hay	Positive	Organ Degeneration	4.24E-11	258
Mammary	Alfalfa				
gland	hay	Positive	apoptosis of blood cells	4.25E-11	259
Mammary	Alfalfa				
gland	hay	Positive	small GTPase mediated signal transduction	4.70E-11	107
Mammary	Alfalfa				
gland	hay	Positive	cell movement of leukocytes	5.46E-11	386
Mammary	Alfalfa				
gland	hay	Positive	development of head	5.97E-11	484
Mammary	Alfalfa				
gland	hay	Positive	abdominal carcinoma	6.26E-11	2647
Mammary	Alfalfa				
gland	hay	Positive	survival of organism	6.70E-11	356
Mammary	Alfalfa				
gland	hay	Positive	formation of filaments	8.32E-11	211
Mammary	Alfalfa				
gland	hay	Positive	replication of virus	8.39E-11	289
Mammary	Alfalfa				
gland	hay	Positive	infection by Retroviridae	8.39E-11	363
Mammary	Alfalfa				
gland	hay	Positive	growth of embryo	8.39E-11	249
Mammary	Alfalfa				
gland	hay	Positive	Neurodegeneration	8.40E-11	168
Mammary	Alfalfa				
gland	hay	Positive	morphology of respiratory system	8.84E-11	169
Mammary	Alfalfa				
gland	hay	Positive	mitosis	8.96E-11	258
Mammary	Alfalfa				
gland	hay	Positive	development of lymphocytes	9.88E-11	285
Mammary	Alfalfa				
gland	hay	Positive	perinatal death	1.14E-10	304
Mammary	Alfalfa	Positive	congenital anomaly of musculoskeletal system	1.20E-10	370

gland	hay				
Mammary gland	Alfalfa hay	Positive	development of body axis	1.22E-10	514
Mammary gland	Alfalfa hay	Positive	infection by RNA virus	1.26E-10	430
Mammary gland	Alfalfa hay	Positive	concentration of fatty acid	1.28E-10	153
Mammary gland	Alfalfa hay	Positive	morphology of head	1.34E-10	434
Mammary gland	Alfalfa hay	Positive	cell movement of myeloid cells	1.39E-10	278
Mammary gland	Alfalfa hay	Positive	infection of cells	1.41E-10	398
Mammary gland	Alfalfa hay	Positive	size of animal	1.54E-10	103
Mammary gland	Alfalfa hay	Positive	development of mononuclear leukocytes	1.65E-10	286
Mammary gland	Alfalfa hay	Positive	female genital neoplasm	1.76E-10	1606
Mammary gland	Alfalfa hay	Positive	infection by lentivirus	1.82E-10	359
Mammary gland	Alfalfa hay	Positive	HIV infection	1.83E-10	358
Mammary gland	Alfalfa hay	Positive	morphology of digestive system	1.92E-10	292
Mammary gland	Alfalfa hay	Positive	sprouting	1.97E-10	204
Mammary gland	Alfalfa hay	Positive	endocytosis	2.12E-10	184
Mammary gland	Alfalfa hay	Positive	synthesis of fatty acid	2.24E-10	168
Mammary gland	Alfalfa hay	Positive	development of epithelial tissue	2.28E-10	266
Mammary gland	Alfalfa hay	Positive	abnormal morphology of digestive system	2.30E-10	279
Mammary gland	Alfalfa hay	Positive	growth of plasma membrane projections	2.39E-10	242
Mammary gland	Alfalfa hay	Positive	proliferation of immune cells	2.59E-10	394
Mammary gland	Alfalfa hay	Positive	morphology of lymphatic system component	2.60E-10	218
Mammary gland	Alfalfa hay	Positive	morphology of nervous system	2.74E-10	390
Mammary	Alfalfa	Positive	hypersensitive reaction	2.85E-10	224

gland	hay				
Mammary gland	Alfalfa hay	Positive	differentiation of connective tissue	2.96E-10	365
Mammary gland	Alfalfa hay	Positive	metabolism of protein	3.00E-10	476
Mammary gland	Alfalfa hay	Positive	abnormal morphology of respiratory system	3.13E-10	165
Mammary gland	Alfalfa hay	Positive	quantity of lymphatic system component	3.25E-10	199
Mammary gland	Alfalfa hay	Positive	infection by HIV-1	3.33E-10	309
Mammary gland	Alfalfa hay	Positive	differentiation of leukocytes	3.52E-10	338
Mammary gland	Alfalfa hay	Positive	arthropathy	4.22E-10	466
Mammary gland	Alfalfa hay	Positive	Lymphocyte homeostasis	4.56E-10	278
Mammary gland	Alfalfa hay	Positive	abnormal morphology of reproductive system	4.56E-10	261
Mammary gland	Alfalfa hay	Positive	autophagy	4.80E-10	197
Mammary gland	Alfalfa hay	Positive	formation of plasma membrane projections	4.84E-10	302
Mammary gland	Alfalfa hay	Positive	abnormal morphology of head	4.95E-10	411
Mammary gland	Alfalfa hay	Positive	growth of lesion	4.97E-10	395
Mammary gland	Alfalfa hay	Positive	apoptosis of epithelial cell lines	5.13E-10	124
Mammary gland	Alfalfa hay	Positive	morphology of reproductive system	5.37E-10	273
Mammary gland	Alfalfa hay	Positive	quantity of carbohydrate	5.37E-10	265
Mammary gland	Alfalfa hay	Positive	proliferation of hematopoietic cells	5.46E-10	139
Mammary gland	Alfalfa hay	Positive	arthritis	5.47E-10	458
Mammary gland	Alfalfa hay	Positive	development of connective tissue	5.50E-10	195
Mammary gland	Alfalfa hay	Positive	proliferation of fibroblast cell lines	5.80E-10	220
Mammary gland	Alfalfa hay	Positive	morphology of vessel	6.08E-10	164
Mammary	Alfalfa	Positive	engulfment of cells	6.29E-10	212

gland	hay				
Mammary gland	Alfalfa hay	Positive	development of lymphatic system component	6.32E-10	165
Mammary gland	Alfalfa hay	Positive	development of cardiovascular tissue	6.62E-10	194
Mammary gland	Alfalfa hay	Positive	neuritogenesis	6.75E-10	295
Mammary gland	Alfalfa hay	Positive	growth of neurites	6.86E-10	238
Mammary gland	Alfalfa hay	Positive	Bleeding	6.96E-10	220
Mammary gland	Alfalfa hay	Positive	homing of cells	7.10E-10	283
Mammary gland	Alfalfa hay	Positive	activation of DNA endogenous promoter	7.31E-10	579
Mammary gland	Alfalfa hay	Positive	T cell development	7.31E-10	257
Mammary gland	Alfalfa hay	Positive	growth of tumor	8.55E-10	393
Mammary gland	Alfalfa hay	Positive	homing	9.63E-10	290
Mammary gland	Alfalfa hay	Positive	morphology of muscle	9.90E-10	177
Mammary gland	Alfalfa hay	Positive	homeostasis of leukocytes	1.00E-09	281
Mammary gland	Alfalfa hay	Positive	cell death of cervical cancer cell lines	1.23E-09	184
Mammary gland	Alfalfa hay	Positive	branching of cells	1.23E-09	193
Mammary gland	Alfalfa hay	Positive	function of cardiovascular system	1.23E-09	193
Mammary gland	Alfalfa hay	Positive	breast or ovarian cancer	1.31E-09	887
Mammary gland	Alfalfa hay	Positive	proliferation of epithelial cells	1.51E-09	251
Mammary gland	Alfalfa hay	Positive	morphogenesis of neurons	1.52E-09	219
Mammary gland	Alfalfa hay	Positive	quantity of lymphoid organ	1.52E-09	163
Mammary gland	Alfalfa hay	Positive	cell movement of endothelial cells	1.56E-09	185
Mammary gland	Alfalfa hay	Positive	synthesis of nitric oxide	1.59E-09	148
Mammary	Alfalfa	Positive	development of neurons	1.69E-09	385

gland	hay				
Mammary gland	Alfalfa hay	Positive	development of lymphatic system	2.01E-09	186
Mammary gland	Alfalfa hay	Positive	outgrowth of plasma membrane projections	2.04E-09	210
Mammary gland	Alfalfa hay	Positive	apoptosis of lymphocytes	2.20E-09	181
Mammary gland	Alfalfa hay	Positive	mass of connective tissue	2.24E-09	111
Mammary gland	Alfalfa hay	Positive	vascularization	2.26E-09	128
Mammary gland	Alfalfa hay	Positive	apoptosis of mononuclear leukocytes	2.48E-09	186
Mammary gland	Alfalfa hay	Positive	T cell homeostasis	2.52E-09	261
Mammary gland	Alfalfa hay	Positive	uptake of carbohydrate	2.76E-09	154
Mammary gland	Alfalfa hay	Positive	apoptosis of T lymphocytes	2.79E-09	149
Mammary gland	Alfalfa hay	Positive	apoptosis of neurons	2.79E-09	223
Mammary gland	Alfalfa hay	Positive	proliferation of hematopoietic progenitor cells	2.79E-09	131
Mammary gland	Alfalfa hay	Positive	morphology of blood vessel	2.91E-09	150
Mammary gland	Alfalfa hay	Positive	apoptosis of leukocytes	2.93E-09	233
Mammary gland	Alfalfa hay	Positive	autosomal dominant disease	3.05E-09	317
Mammary gland	Alfalfa hay	Positive	female genital tract serous cancer	3.13E-09	199
Mammary gland	Alfalfa hay	Positive	gastrointestinal carcinoma	3.32E-09	2008
Mammary gland	Alfalfa hay	Positive	activation of enzyme	3.94E-09	200
Mammary gland	Alfalfa hay	Positive	cognition	4.23E-09	231
Mammary gland	Alfalfa hay	Positive	formation of lymphatic system component	4.47E-09	154
Mammary gland	Alfalfa hay	Positive	morphogenesis of neurites	4.62E-09	214
Mammary gland	Alfalfa hay	Positive	development of endothelial tissue	4.97E-09	189
Mammary	Alfalfa	Positive	outgrowth of neurites	5.26E-09	207

gland	hay				
Mammary gland	Alfalfa hay	Positive	advanced malignant tumor	5.65E-09	411
Mammary gland	Alfalfa hay	Positive	differentiation of blood cells	5.76E-09	410
Mammary gland	Alfalfa hay	Positive	cell spreading	6.63E-09	146
Mammary gland	Alfalfa hay	Positive	transport of protein	6.65E-09	158
Mammary gland	Alfalfa hay	Positive	morphology of genital organ	6.89E-09	216
Mammary gland	Alfalfa hay	Positive	quantity of protein in blood	7.12E-09	255
Mammary gland	Alfalfa hay	Positive	tumorigenesis of genital organ	7.36E-09	1742
Mammary gland	Alfalfa hay	Positive	cellular degradation	8.12E-09	135
Mammary gland	Alfalfa hay	Positive	formation of lymphoid organ	8.16E-09	140
Mammary gland	Alfalfa hay	Positive	proliferation of mononuclear leukocytes	8.76E-09	361
Mammary gland	Alfalfa hay	Positive	activation of neuroglia	8.81E-09	76
Mammary gland	Alfalfa hay	Positive	uptake of monosaccharide	8.97E-09	142
Mammary gland	Alfalfa hay	Positive	cell death of kidney cells	9.68E-09	180
Mammary gland	Alfalfa hay	Positive	accumulation of lipid	9.83E-09	156
Mammary gland	Alfalfa hay	Positive	endothelial cell development	1.00E-08	182
Mammary gland	Alfalfa hay	Positive	formation of lung	1.01E-08	157
Mammary gland	Alfalfa hay	Positive	quantity of steroid	1.04E-08	235
Mammary gland	Alfalfa hay	Positive	gastrointestinal tract cancer	1.04E-08	2226
Mammary gland	Alfalfa hay	Positive	learning	1.17E-08	212
Mammary gland	Alfalfa hay	Positive	Gastrointestinal Tract Cancer and Tumors	1.20E-08	2260
Mammary gland	Alfalfa hay	Positive	quantity of adipose tissue	1.37E-08	146
Mammary	Alfalfa	Positive	gonadal tumor	1.40E-08	397

gland	hay				
Mammary gland	Alfalfa hay	Positive	cell movement of phagocytes	1.43E-08	273
Mammary gland	Alfalfa hay	Positive	differentiation of mononuclear leukocytes	1.47E-08	269
Mammary gland	Alfalfa hay	Positive	cell movement of muscle cells	1.50E-08	99
Mammary gland	Alfalfa hay	Positive	apoptosis of epithelial cells	1.53E-08	149
Mammary gland	Alfalfa hay	Positive	phosphorylation of amino acids	1.83E-08	118
Mammary gland	Alfalfa hay	Positive	morphology of lymphoid organ	1.89E-08	199
Mammary gland	Alfalfa hay	Positive	abnormal morphology of nervous system	1.96E-08	353
Mammary gland	Alfalfa hay	Positive	abnormal morphology of blood vessel	2.12E-08	139
Mammary gland	Alfalfa hay	Positive	chemotaxis of cells	2.15E-08	262
Mammary gland	Alfalfa hay	Positive	accumulation of cells	2.22E-08	180
Mammary gland	Alfalfa hay	Positive	serous neoplasm	2.26E-08	300
Mammary gland	Alfalfa hay	Positive	size of embryo	2.28E-08	182
Mammary gland	Alfalfa hay	Positive	differentiation of lymphocytes	2.34E-08	248
Mammary gland	Alfalfa hay	Positive	inflammatory response	2.36E-08	377
Mammary gland	Alfalfa hay	Positive	cell movement of smooth muscle cells	2.37E-08	89
Mammary gland	Alfalfa hay	Positive	apoptosis of muscle	2.44E-08	137
Mammary gland	Alfalfa hay	Positive	synthesis of carbohydrate	2.44E-08	214
Mammary gland	Alfalfa hay	Positive	respiratory system development	2.45E-08	175
Mammary gland	Alfalfa hay	Positive	outgrowth of cells	2.45E-08	218
Mammary gland	Alfalfa hay	Positive	abnormal morphology of lymphoid organ	2.54E-08	192
Mammary gland	Alfalfa hay	Positive	peripheral vascular disease	2.56E-08	193
Mammary	Alfalfa	Positive	tumorigenesis of gonad	2.60E-08	390

gland	hay				
Mammary gland	Alfalfa hay	Positive	dysgenesis	2.76E-08	267
Mammary gland	Alfalfa hay	Positive	Ovarian Cancer and Tumors	2.82E-08	367
Mammary gland	Alfalfa hay	Positive	genital tract cancer	2.82E-08	1721
Mammary gland	Alfalfa hay	Positive	proliferation of lymphocytes	2.82E-08	353
Mammary gland	Alfalfa hay	Positive	peripheral arterial disease	2.85E-08	113
Mammary gland	Alfalfa hay	Positive	phosphorylation of L-amino acid	2.86E-08	117
Mammary gland	Alfalfa hay	Positive	apoptosis of muscle cells	2.90E-08	136
Mammary gland	Alfalfa hay	Positive	cell movement of carcinoma cell lines	2.99E-08	110
Mammary gland	Alfalfa hay	Positive	craniofacial abnormality	3.26E-08	181
Mammary gland	Alfalfa hay	Positive	necrosis of kidney	3.43E-08	185
Mammary gland	Alfalfa hay	Positive	quantity of hematopoietic progenitor cells	3.44E-08	221
Mammary gland	Alfalfa hay	Positive	secretion of molecule	3.52E-08	272
Mammary gland	Alfalfa hay	Positive	chemotaxis	3.63E-08	269
Mammary gland	Alfalfa hay	Positive	mass of adipose tissue	3.66E-08	102
Mammary gland	Alfalfa hay	Positive	concentration of phospholipid	3.73E-08	99
Mammary gland	Alfalfa hay	Positive	infection of embryonic cell lines	3.83E-08	143
Mammary gland	Alfalfa hay	Positive	infection of epithelial cell lines	3.83E-08	143
Mammary gland	Alfalfa hay	Positive	formation of blood cells	3.83E-08	106
Mammary gland	Alfalfa hay	Positive	arrest in interphase	4.00E-08	209
Mammary gland	Alfalfa hay	Positive	metastasis	4.02E-08	367
Mammary gland	Alfalfa hay	Positive	export of molecule	4.06E-08	152
Mammary	Alfalfa	Positive	Hypoplasia	4.21E-08	250

gland	hay				
Mammary gland	Alfalfa hay	Positive	morphology of lung	4.47E-08	101
Mammary gland	Alfalfa hay	Positive	activation of blood cells	4.51E-08	361
Mammary gland	Alfalfa hay	Positive	abnormal morphology of genital organ	4.71E-08	205
Mammary gland	Alfalfa hay	Positive	differentiation of bone	4.72E-08	206
Mammary gland	Alfalfa hay	Positive	cell death of brain	4.76E-08	150
Mammary gland	Alfalfa hay	Positive	Dermatitis	4.76E-08	217
Mammary gland	Alfalfa hay	Positive	formation of leukocytes	4.84E-08	87
Mammary gland	Alfalfa hay	Positive	migration of smooth muscle cells	4.94E-08	82
Mammary gland	Alfalfa hay	Positive	migration of muscle cells	4.97E-08	90
Mammary gland	Alfalfa hay	Positive	targeting of protein	5.16E-08	73
Mammary gland	Alfalfa hay	Positive	differentiation of T lymphocytes	5.17E-08	191
Mammary gland	Alfalfa hay	Positive	growth of lymphatic system component	5.24E-08	103
Mammary gland	Alfalfa hay	Positive	differentiation of muscle	5.41E-08	155
Mammary gland	Alfalfa hay	Positive	proliferation of lymphatic system cells	5.41E-08	107
Mammary gland	Alfalfa hay	Positive	metabolism of DNA	5.41E-08	200
Mammary gland	Alfalfa hay	Positive	urogenital cancer	5.41E-08	1962
Mammary gland	Alfalfa hay	Positive	differentiation of bone cells	5.47E-08	204
Mammary gland	Alfalfa hay	Positive	formation of actin filaments	5.47E-08	156
Mammary gland	Alfalfa hay	Positive	migration of endothelial cells	5.59E-08	167
Mammary gland	Alfalfa hay	Positive	degeneration of neurons	5.69E-08	125
Mammary gland	Alfalfa hay	Positive	cell death of fibroblasts	5.96E-08	137
Mammary	Alfalfa	Positive	blood protein disorder	6.03E-08	189

gland	hay				
Mammary gland	Alfalfa hay	Positive	accumulation of blood cells	6.03E-08	144
Mammary gland	Alfalfa hay	Positive	apoptosis of cervical cancer cell lines	6.23E-08	145
Mammary gland	Alfalfa hay	Positive	quantity of thymus gland	6.24E-08	122
Mammary gland	Alfalfa hay	Positive	quantity of B lymphocytes	6.28E-08	175
Mammary gland	Alfalfa hay	Positive	infection of kidney cell lines	6.39E-08	146
Mammary gland	Alfalfa hay	Positive	cell death of muscle cells	7.18E-08	170
Mammary gland	Alfalfa hay	Positive	generation of leukocytes	7.43E-08	88
Mammary gland	Alfalfa hay	Positive	degeneration of cells	7.58E-08	174
Mammary gland	Alfalfa hay	Positive	cell movement of neutrophils	7.58E-08	152
Mammary gland	Alfalfa hay	Positive	invasion of carcinoma cell lines	8.07E-08	105
Mammary gland	Alfalfa hay	Positive	transmembrane potential of mitochondria	8.10E-08	113
Mammary gland	Alfalfa hay	Positive	function of phagocytes	8.34E-08	156
Mammary gland	Alfalfa hay	Positive	synthesis of reactive oxygen species	8.38E-08	237
Mammary gland	Alfalfa hay	Positive	production of reactive oxygen species	8.39E-08	184
Mammary gland	Alfalfa hay	Positive	paraproteinemia	8.71E-08	170
Mammary gland	Alfalfa hay	Positive	quantity of antigen presenting cells	8.85E-08	142
Mammary gland	Alfalfa hay	Positive	quantity of phagocytes	9.22E-08	211
Mammary gland	Alfalfa hay	Positive	tumorigenesis of reproductive tract	9.44E-08	1517
Mammary gland	Alfalfa hay	Positive	cell death of central nervous system cells	1.09E-07	149
Mammary gland	Alfalfa hay	Positive	generation of blood cells	1.15E-07	89
Mammary gland	Alfalfa hay	Positive	abnormal morphology of internal genitalia	1.15E-07	176
Mammary	Alfalfa	Positive	quantity of thymocytes	1.22E-07	120

Mammary gland	Alfalfa hay	Positive	cell death of kidney cell lines	1.23E-07	154
Mammary gland	Alfalfa hay	Positive	branching of neurons	1.25E-07	146
Mammary gland	Alfalfa hay	Positive	I-kappaB kinase/NF-kappaB cascade	1.26E-07	93
Mammary gland	Alfalfa hay	Positive	plasma cell dyscrasia	1.29E-07	169
Mammary gland	Alfalfa hay	Positive	concentration of cholesterol	1.32E-07	148
Mammary gland	Alfalfa hay	Positive	function of mononuclear leukocytes	1.33E-07	172
Mammary gland	Alfalfa hay	Positive	cell death of muscle	1.34E-07	173
Mammary gland	Alfalfa hay	Positive	abnormal morphology of muscle	1.36E-07	160
Mammary gland	Alfalfa hay	Positive	Hypertrophy	1.39E-07	233
Mammary gland	Alfalfa hay	Positive	activation of leukocytes	1.50E-07	336
Mammary gland	Alfalfa hay	Positive	concentration of sterol	1.53E-07	155
Mammary gland	Alfalfa hay	Positive	metabolism of membrane lipid derivative	1.53E-07	193
Mammary gland	Alfalfa hay	Positive	cell viability of cervical cancer cell lines	1.53E-07	115
Mammary gland	Alfalfa hay	Positive	function of lymphocytes	1.59E-07	171
Mammary gland	Alfalfa hay	Positive	necrosis of muscle	1.61E-07	172
Mammary gland	Alfalfa hay	Positive	cell death of tumor	1.83E-07	190
Mammary gland	Alfalfa hay	Positive	cellular infiltration of blood cells	1.84E-07	193
Mammary gland	Alfalfa hay	Positive	branching of neurites	2.13E-07	141
Mammary gland	Alfalfa hay	Positive	proliferation of vascular smooth muscle cells	2.15E-07	83
Mammary gland	Alfalfa hay	Positive	morphology of pulmonary alveolus	2.20E-07	66
Mammary gland	Alfalfa hay	Positive	autophagy of cells	2.23E-07	135
Mammary	Alfalfa	Positive	growth of skin	2.34E-07	113

gland	hay				
Mammary gland	Alfalfa hay	Positive	morphology of heart ventricle	2.47E-07	101
Mammary gland	Alfalfa hay	Positive	female genital tract cancer	2.49E-07	1503
Mammary gland	Alfalfa hay	Positive	neoplasia of cells	2.49E-07	218
Mammary gland	Alfalfa hay	Positive	incidence of tumor	2.56E-07	161
Mammary gland	Alfalfa hay	Positive	cell movement of granulocytes	2.58E-07	184
Mammary gland	Alfalfa hay	Positive	development of digestive system	2.62E-07	190
Mammary gland	Alfalfa hay	Positive	cellular infiltration by leukocytes	2.62E-07	192
Mammary gland	Alfalfa hay	Positive	allergy	2.62E-07	200
Mammary gland	Alfalfa hay	Positive	morphology of muscle cells	2.64E-07	88
Mammary gland	Alfalfa hay	Positive	biosynthesis of polyunsaturated fatty acids	2.69E-07	116
Mammary gland	Alfalfa hay	Positive	mass of organism	2.73E-07	167
Mammary gland	Alfalfa hay	Positive	cell death of hematopoietic cells	2.76E-07	122
Mammary gland	Alfalfa hay	Positive	bone mineral density	2.81E-07	92
Mammary gland	Alfalfa hay	Positive	formation of actin stress fibers	2.87E-07	123
Mammary gland	Alfalfa hay	Positive	epileptic seizure	2.88E-07	89
Mammary gland	Alfalfa hay	Positive	progression of tumor	2.91E-07	86
Mammary gland	Alfalfa hay	Positive	abnormal morphology of lung	2.91E-07	96
Mammary gland	Alfalfa hay	Positive	cell death of cerebral cortex cells	2.93E-07	118
Mammary gland	Alfalfa hay	Positive	congenital malformation of skeleton	3.00E-07	208
Mammary gland	Alfalfa hay	Positive	cellular infiltration of cells	3.08E-07	196
Mammary gland	Alfalfa hay	Positive	mammary tumor	3.14E-07	748
Mammary	Alfalfa	Positive	development of hematopoietic system	3.32E-07	127

gland	hay				
Mammary gland	Alfalfa hay	Positive	function of muscle	3.45E-07	173
Mammary gland	Alfalfa hay	Positive	apoptosis of kidney cell lines	3.48E-07	122
Mammary gland	Alfalfa hay	Positive	cell death of tumor cells	3.63E-07	184
Mammary gland	Alfalfa hay	Positive	morphology of gonad	3.65E-07	187
Mammary gland	Alfalfa hay	Positive	necrosis of tumor	3.65E-07	188
Mammary gland	Alfalfa hay	Positive	metabolism of reactive oxygen species	3.67E-07	243
Mammary gland	Alfalfa hay	Positive	transformation of fibroblast cell lines	3.77E-07	131
Mammary gland	Alfalfa hay	Positive	hepatobiliary system cancer	4.02E-07	1903
Mammary gland	Alfalfa hay	Positive	differentiation of muscle cells	4.06E-07	142
Mammary gland	Alfalfa hay	Positive	ovarian cancer	4.22E-07	339
Mammary gland	Alfalfa hay	Positive	migration of carcinoma cell lines	4.23E-07	90
Mammary gland	Alfalfa hay	Positive	binding of protein binding site	4.32E-07	145
Mammary gland	Alfalfa hay	Positive	vascular lesion	4.35E-07	116
Mammary gland	Alfalfa hay	Positive	intermediate disease stage peripheral arterial disease	4.41E-07	84
Mammary gland	Alfalfa hay	Positive	quantity of cytokine	4.42E-07	129
Mammary gland	Alfalfa hay	Positive	development of abdomen	4.55E-07	279
Mammary gland	Alfalfa hay	Positive	cell death of pheochromocytoma cell lines	4.61E-07	66
Mammary gland	Alfalfa hay	Positive	cellular infiltration	4.70E-07	214
Mammary gland	Alfalfa hay	Positive	generation of lymphocytes	4.70E-07	76
Mammary gland	Alfalfa hay	Positive	movement of vascular endothelial cells	4.70E-07	95
Mammary gland	Alfalfa hay	Positive	cell death of embryonic cells	4.76E-07	64
Mammary	Alfalfa	Positive	S phase	4.80E-07	132

gland	hay				
Mammary gland	Alfalfa hay	Positive	cell viability of connective tissue cells	5.57E-07	72
Mammary gland	Alfalfa hay	Positive	concentration of acylglycerol	5.70E-07	166
Mammary gland	Alfalfa hay	Positive	catabolism of protein	5.76E-07	289
Mammary gland	Alfalfa hay	Positive	secretory pathway	5.77E-07	102
Mammary gland	Alfalfa hay	Positive	Rheumatic Disease	5.78E-07	501
Mammary gland	Alfalfa hay	Positive	G1 phase	6.09E-07	191
Mammary gland	Alfalfa hay	Positive	synthesis of eicosanoid	6.17E-07	113
Mammary gland	Alfalfa hay	Positive	quantity of macrophages	6.50E-07	104
Mammary gland	Alfalfa hay	Positive	hepatocellular carcinoma	6.51E-07	1834
Mammary gland	Alfalfa hay	Positive	apoptosis of pheochromocytoma cell lines	7.04E-07	52
Mammary gland	Alfalfa hay	Positive	skin abnormality	7.04E-07	116
Mammary gland	Alfalfa hay	Positive	quantity of double-positive thymocyte	7.31E-07	62
Mammary gland	Alfalfa hay	Positive	colorectal neoplasia	7.35E-07	1901
Mammary gland	Alfalfa hay	Positive	generation of mononuclear leukocytes	7.41E-07	77
Mammary gland	Alfalfa hay	Positive	function of antigen presenting cells	7.43E-07	124
Mammary gland	Alfalfa hay	Positive	Cytosis	7.83E-07	159
Mammary gland	Alfalfa hay	Positive	formation of filopodia	7.87E-07	87
Mammary gland	Alfalfa hay	Positive	formation of connective tissue cells	7.99E-07	108
Mammary gland	Alfalfa hay	Positive	metabolism of nucleic acid component or derivative	7.99E-07	250
Mammary gland	Alfalfa hay	Positive	cell death of cortical neurons	8.12E-07	91
Mammary gland	Alfalfa hay	Positive	adhesion of immune cells	8.47E-07	172
Mammary	Alfalfa	Positive	Oral Cancer and Tumors	8.88E-07	116

gland	hay				
Mammary gland	Alfalfa hay	Positive	morphology of intestine	8.88E-07	116
Mammary gland	Alfalfa hay	Positive	formation of eye	8.89E-07	213
Mammary gland	Alfalfa hay	Positive	morphology of secondary lymphoid organ	8.90E-07	140
Mammary gland	Alfalfa hay	Positive	mass of fat pad	8.91E-07	61
Mammary gland	Alfalfa hay	Positive	shape change of neurites	9.21E-07	142
Mammary gland	Alfalfa hay	Positive	benign neoplasm of female genital organ	9.24E-07	207
Mammary gland	Alfalfa hay	Positive	liver tumor	9.58E-07	1902
Mammary gland	Alfalfa hay	Positive	adenoma	9.64E-07	265
Mammary gland	Alfalfa hay	Positive	accumulation of leukocytes	9.64E-07	134
Mammary gland	Alfalfa hay	Positive	liver lesion	9.75E-07	1903
Mammary gland	Alfalfa hay	Positive	DNA replication	9.75E-07	126
Mammary gland	Alfalfa hay	Positive	recruitment of cells	1.01E-06	175
Mammary gland	Alfalfa hay	Positive	quantity of natural killer T lymphocytes	1.01E-06	39
Mammary gland	Alfalfa hay	Positive	pelvic tumor	1.02E-06	1907
Mammary gland	Alfalfa hay	Positive	cell death of sarcoma cell lines	1.07E-06	115
Mammary gland	Alfalfa hay	Positive	oral cancer	1.07E-06	115
Mammary gland	Alfalfa hay	Positive	colon cancer	1.09E-06	1625
Mammary gland	Alfalfa hay	Positive	proliferation of T lymphocytes	1.10E-06	288
Mammary gland	Alfalfa hay	Positive	shape change of tumor cell lines	1.10E-06	81
Mammary gland	Alfalfa hay	Positive	tumorigenesis of intestine	1.15E-06	1633
Mammary gland	Alfalfa hay	Positive	urination disorder	1.15E-06	144
Mammary	Alfalfa	Positive	intestinal tumor	1.15E-06	1904

gland	hay				
Mammary gland	Alfalfa hay	Positive	gastrointestinal adenocarcinoma	1.20E-06	1866
Mammary gland	Alfalfa hay	Positive	development of central nervous system	1.21E-06	303
Mammary gland	Alfalfa hay	Positive	pelvic cancer	1.22E-06	1894
Mammary gland	Alfalfa hay	Positive	cell death of brain cells	1.22E-06	136
Mammary gland	Alfalfa hay	Positive	organization of actin cytoskeleton	1.22E-06	149
Mammary gland	Alfalfa hay	Positive	uptake of D-hexose	1.24E-06	113
Mammary gland	Alfalfa hay	Positive	function of myeloid cells	1.26E-06	120
Mammary gland	Alfalfa hay	Positive	morphology of spleen	1.29E-06	139
Mammary gland	Alfalfa hay	Positive	apoptosis of liver	1.32E-06	83
Mammary gland	Alfalfa hay	Positive	cell death of heart	1.33E-06	115
Mammary gland	Alfalfa hay	Positive	cell death of hematopoietic progenitor cells	1.33E-06	115
Mammary gland	Alfalfa hay	Positive	cell death of neuroblastoma cell lines	1.34E-06	99
Mammary gland	Alfalfa hay	Positive	formation of osteoclasts	1.34E-06	66
Mammary gland	Alfalfa hay	Positive	quantity of interleukin	1.35E-06	74
Mammary gland	Alfalfa hay	Positive	phosphorylation of L-tyrosine	1.38E-06	91
Mammary gland	Alfalfa hay	Positive	abnormal morphology of gonad	1.40E-06	178
Mammary gland	Alfalfa hay	Positive	cell death of liver cells	1.41E-06	100
Mammary gland	Alfalfa hay	Positive	neoplasia of colon	1.42E-06	1628
Mammary gland	Alfalfa hay	Positive	morphology of testis	1.46E-06	135
Mammary gland	Alfalfa hay	Positive	development of sensory organ	1.50E-06	269
Mammary gland	Alfalfa hay	Positive	formation of vascular lesion	1.50E-06	62
Mammary	Alfalfa	Positive	liver cancer	1.51E-06	1887

gland	hay				
Mammary gland	Alfalfa hay	Positive	colon tumor	1.67E-06	1632
Mammary gland	Alfalfa hay	Positive	cell movement of fibroblasts	1.68E-06	94
Mammary gland	Alfalfa hay	Positive	colorectal cancer	1.70E-06	1871
Mammary gland	Alfalfa hay	Positive	cytopenia	1.75E-06	147
Mammary gland	Alfalfa hay	Positive	apoptosis of fibroblasts	1.75E-06	110
Mammary gland	Alfalfa hay	Positive	binding of cells	1.77E-06	230
Mammary gland	Alfalfa hay	Positive	morphology of skin	1.79E-06	135
Mammary gland	Alfalfa hay	Positive	cellular infiltration of phagocytes	1.80E-06	91
Mammary gland	Alfalfa hay	Positive	quantity of reactive oxygen species	1.80E-06	91
Mammary gland	Alfalfa hay	Positive	cell death of heart cells	1.81E-06	111
Mammary gland	Alfalfa hay	Positive	ion homeostasis of cells	1.82E-06	265
Mammary gland	Alfalfa hay	Positive	production of HIV	1.82E-06	35
Mammary gland	Alfalfa hay	Positive	connective or soft tissue tumor	1.90E-06	332
Mammary gland	Alfalfa hay	Positive	polymerization of protein	1.94E-06	180
Mammary gland	Alfalfa hay	Positive	differentiation of epithelial tissue	1.96E-06	175
Mammary gland	Alfalfa hay	Positive	apoptosis of hematopoietic cells	2.13E-06	109
Mammary gland	Alfalfa hay	Positive	function of heart	2.15E-06	116
Mammary gland	Alfalfa hay	Positive	uterine serous papillary cancer	2.15E-06	116
Mammary gland	Alfalfa hay	Negative	organismal death	2.24E-18	502
Mammary gland	Alfalfa hay	Negative	morbidity or mortality	2.71E-18	506
Mammary gland	Alfalfa hay	Negative	malignant solid tumor	4.23E-18	1770
Mammary	Alfalfa	Negative	cancer	4.23E-18	1788

gland	hay				
Mammary gland	Alfalfa hay	Negative	morphology of cells	1.04E-15	411
Mammary gland	Alfalfa hay	Negative	organization of cytoplasm	1.30E-13	330
Mammary gland	Alfalfa hay	Negative	cell death	1.38E-13	632
Mammary gland	Alfalfa hay	Negative	proliferation of cells	1.98E-13	688
Mammary gland	Alfalfa hay	Negative	organization of cytoskeleton	4.15E-13	303
Mammary gland	Alfalfa hay	Negative	tumorigenesis of tissue	4.97E-12	1495
Mammary gland	Alfalfa hay	Negative	apoptosis	1.11E-11	508
Mammary gland	Alfalfa hay	Negative	neoplasia of epithelial tissue	1.48E-11	1470
Mammary gland	Alfalfa hay	Negative	transport of molecule	6.71E-11	331
Mammary gland	Alfalfa hay	Negative	cell movement	7.74E-11	418
Mammary gland	Alfalfa hay	Negative	epithelial cancer	9.72E-11	1452
Mammary gland	Alfalfa hay	Negative	necrosis	1.21E-10	490
Mammary gland	Alfalfa hay	Negative	microtubule dynamics	1.64E-10	254
Mammary gland	Alfalfa hay	Negative	development of vasculature	9.99E-10	107
Mammary gland	Alfalfa hay	Negative	morphology of cardiovascular system	3.69E-09	145
Mammary gland	Alfalfa hay	Negative	Movement Disorders	6.79E-09	225
Mammary gland	Alfalfa hay	Negative	abnormal morphology of cells	1.95E-08	259
Mammary gland	Alfalfa hay	Negative	migration of cells	4.78E-08	365
Mammary gland	Alfalfa hay	Negative	differentiation of cells	8.03E-08	421
Mammary gland	Alfalfa hay	Negative	proliferation of connective tissue cells	1.71E-07	134
Mammary gland	Alfalfa hay	Negative	abnormal morphology of cardiovascular system	1.85E-07	129
Mammary	Alfalfa	Negative	quantity of cells	1.85E-07	339

gland	hay				
Mammary gland	Alfalfa hay	Negative	transcription	2.21E-07	376
Mammary gland	Alfalfa hay	Negative	growth of connective tissue	2.23E-07	143
Mammary gland	Alfalfa hay	Negative	formation of cellular protrusions	2.41E-07	190
Mammary gland	Alfalfa hay	Negative	urination disorder	2.61E-07	74
Mammary gland	Alfalfa hay	Negative	abdominal neoplasm	2.70E-07	1429
Mammary gland	Alfalfa hay	Negative	transcription of RNA	3.19E-07	352
Mammary gland	Alfalfa hay	Negative	cell viability	3.79E-07	258
Mammary gland	Alfalfa hay	Negative	cell death of tumor cell lines	9.49E-07	294
Mammary gland	Alfalfa hay	Negative	cell movement of tumor cell lines	9.58E-07	178
Mammary gland	Alfalfa hay	Negative	expression of RNA	1.08E-06	396
Mammary gland	Alfalfa hay	Negative	cell survival	1.15E-06	271
Mammary gland	Alfalfa hay	Negative	apoptosis of tumor cell lines	1.20E-06	240
Mammary gland	Alfalfa hay	Negative	neuromuscular disease	1.20E-06	183
Mammary gland	Alfalfa hay	Negative	angiogenesis	1.31E-06	187
Mammary gland	Alfalfa hay	Negative	Viral Infection	1.46E-06	307
Mammary gland	Alfalfa hay	Negative	ruffling	1.50E-06	34
Mammary gland	Alfalfa hay	Negative	morphology of vessel	1.51E-06	75
Mammary gland	Alfalfa hay	Negative	abdominal cancer	3.31E-06	1404
Mammary gland	Alfalfa hay	Negative	cell viability of tumor cell lines	3.76E-06	162
Mammary gland	Alfalfa hay	Negative	morphology of blood vessel	3.88E-06	69
Mammary gland	Alfalfa hay	Negative	growth of organism	4.08E-06	172
Mammary	Alfalfa	Negative	quantity of leukocytes	4.16E-06	186

gland	hay				
Mammary gland	Alfalfa hay	Negative	synthesis of lipid	4.33E-06	150
Mammary gland	Alfalfa hay	Negative	vasculogenesis	4.35E-06	154
Mammary gland	Alfalfa hay	Negative	quantity of blood cells	4.75E-06	205
Mammary gland	Alfalfa hay	Negative	organization of actin cytoskeleton	5.86E-06	72
Mammary gland	Alfalfa hay	Negative	metabolism of carbohydrate	7.32E-06	132
Mammary gland	Alfalfa hay	Negative	necrosis of epithelial tissue	7.50E-06	126
Mammary gland	Alfalfa hay	Negative	digestive organ tumor	7.72E-06	1233
Mammary gland	Alfalfa hay	Negative	digestive system cancer	8.17E-06	1222
Mammary gland	Alfalfa hay	Negative	benign neoplasia	8.22E-06	186
Mammary gland	Alfalfa hay	Negative	cellular homeostasis	1.11E-05	286
Mammary gland	Alfalfa hay	Negative	dyskinesia	1.35E-05	130
Mammary gland	Alfalfa hay	Negative	leukocyte migration	1.70E-05	175
Mammary gland	Alfalfa hay	Negative	transcription of DNA	1.77E-05	287
Mammary gland	Alfalfa hay	Negative	formation of skin	1.77E-05	83
Mammary gland	Alfalfa hay	Negative	proliferation of tumor cell lines	1.77E-05	289
Mammary gland	Alfalfa hay	Negative	seizures	1.92E-05	89
Mammary gland	Alfalfa hay	Negative	proteinuria	1.93E-05	43
Mammary gland	Alfalfa hay	Negative	disorder of basal ganglia	2.01E-05	156
Mammary gland	Alfalfa hay	Negative	invasion of carcinoma cell lines	2.01E-05	50
Mammary gland	Alfalfa hay	Negative	invasion of cells	2.05E-05	174
Mammary gland	Alfalfa hay	Negative	neurological signs	2.31E-05	135
Mammary	Alfalfa	Negative	growth of epithelial tissue	2.50E-05	140

gland	hay				
Mammary gland	Alfalfa hay	Negative	development of body trunk	3.65E-05	215
Mammary gland	Alfalfa hay	Negative	cell death of epithelial cells	3.77E-05	106
Mammary gland	Alfalfa hay	Negative	seizure disorder	3.77E-05	101
Mammary gland	Alfalfa hay	Negative	morphology of heart	3.83E-05	89
Mammary gland	Alfalfa hay	Negative	proliferation of fibroblasts	4.02E-05	79
Mammary gland	Alfalfa hay	Negative	Huntington's Disease	4.02E-05	120
Mammary gland	Alfalfa hay	Negative	growth of embryo	4.35E-05	102
Mammary gland	Alfalfa hay	Negative	Growth Failure	4.73E-05	124
Mammary gland	Alfalfa hay	Negative	cellular infiltration	6.89E-05	94
Mammary gland	Alfalfa hay	Negative	cell death of connective tissue cells	7.04E-05	124
Mammary gland	Alfalfa hay	Negative	abnormal morphology of embryonic tissue	7.20E-05	114
Mammary gland	Alfalfa hay	Negative	abnormal morphology of thoracic cavity	7.20E-05	121
Mammary gland	Alfalfa hay	Negative	Fibrosis	7.88E-05	111
Mammary gland	Alfalfa hay	Negative	abnormal morphology of epithelial tissue	8.55E-05	90
Mammary gland	Alfalfa hay	Negative	migration of tumor cell lines	8.68E-05	142
Mammary gland	Alfalfa hay	Negative	abnormal morphology of blood vessel	1.01E-04	61
Mammary gland	Alfalfa hay	Negative	endocytosis	1.01E-04	76
Mammary gland	Alfalfa hay	Negative	synthesis of nitric oxide	1.10E-04	63
Mammary gland	Alfalfa hay	Negative	cell movement of leukocytes	1.11E-04	152
Mammary gland	Alfalfa hay	Negative	cell death of epithelial cell lines	1.18E-04	64
Mammary gland	Alfalfa hay	Negative	accumulation of cells	1.24E-04	77
Mammary	Alfalfa	Negative	neuritogenesis	1.37E-04	119

gland	hay				
Mammary gland	Alfalfa hay	Negative	formation of plasma membrane projections	1.65E-04	121
Mammary gland	Alfalfa hay	Negative	proliferation of hematopoietic progenitor cells	1.65E-04	56
Mammary gland	Alfalfa hay	Negative	transport of alpha-amino acid	1.66E-04	22
Mammary gland	Alfalfa hay	Negative	proliferation of smooth muscle cells	1.70E-04	64
Mammary gland	Alfalfa hay	Negative	reorganization of cytoskeleton	1.89E-04	46
Mammary gland	Alfalfa hay	Negative	proliferation of hematopoietic cells	1.99E-04	58
Mammary gland	Alfalfa hay	Negative	size of body	2.08E-04	165
Mammary gland	Alfalfa hay	Negative	morphology of bone	2.18E-04	94
Mammary gland	Alfalfa hay	Negative	dysgenesis	2.36E-04	110
Mammary gland	Alfalfa hay	Negative	formation of thymus gland	2.58E-04	40
Mammary gland	Alfalfa hay	Negative	neuronal cell death	2.58E-04	135
Mammary gland	Alfalfa hay	Negative	morphogenesis of neurons	2.72E-04	89
Mammary gland	Alfalfa hay	Negative	synthesis of carbohydrate	2.72E-04	89
Mammary gland	Alfalfa hay	Negative	invasion of tumor cell lines	2.72E-04	132
Mammary gland	Alfalfa hay	Negative	cell death of phagocytes	2.76E-04	49
Mammary gland	Alfalfa hay	Negative	abnormal morphology of bone	2.76E-04	91
Mammary gland	Alfalfa hay	Negative	function of blood cells	2.76E-04	118
Mammary gland	Alfalfa hay	Negative	adhesion of immune cells	2.79E-04	75
Mammary gland	Alfalfa hay	Negative	organization of filaments	2.98E-04	48
Mammary gland	Alfalfa hay	Negative	concentration of lipid	3.07E-04	157
Mammary gland	Alfalfa hay	Negative	quantity of lymphocytes	3.07E-04	139
Mammary	Alfalfa	Negative	Organ Degeneration	3.14E-04	101

gland	hay				
Mammary gland	Alfalfa hay	Negative	abnormal morphology of heart	3.14E-04	81
Mammary gland	Alfalfa hay	Negative	perinatal death	3.14E-04	119
Mammary gland	Alfalfa hay	Negative	Hypoplasia	3.14E-04	103
Mammary gland	Alfalfa hay	Negative	cell death of fibroblasts	3.16E-04	59
Mammary gland	Alfalfa hay	Negative	proliferation of embryonic cells	3.25E-04	56
Mammary gland	Alfalfa hay	Negative	transactivation	3.27E-04	116
Mammary gland	Alfalfa hay	Negative	formation of cells	3.32E-04	186
Mammary gland	Alfalfa hay	Negative	proliferation of bone marrow cells	3.47E-04	31
Mammary gland	Alfalfa hay	Negative	cell movement of myeloid cells	3.57E-04	109
Mammary gland	Alfalfa hay	Negative	development of cytoplasm	3.57E-04	95
Mammary gland	Alfalfa hay	Negative	proliferation of fibroblast cell lines	3.58E-04	88
Mammary gland	Alfalfa hay	Negative	accumulation of lipid	3.85E-04	65
Mammary gland	Alfalfa hay	Negative	function of cardiovascular system	3.91E-04	78
Mammary gland	Alfalfa hay	Negative	cell death of pheochromocytoma cell lines	3.94E-04	31
Mammary gland	Alfalfa hay	Negative	adenoma	3.94E-04	111
Mammary gland	Alfalfa hay	Negative	function of leukocytes	4.43E-04	108
Mammary gland	Alfalfa hay	Negative	apoptosis of neutrophils	4.44E-04	19
Mammary gland	Alfalfa hay	Negative	cellular infiltration by leukocytes	4.50E-04	81
Mammary gland	Alfalfa hay	Negative	metabolism of membrane lipid derivative	4.50E-04	81
Mammary gland	Alfalfa hay	Negative	adhesion of blood cells	4.55E-04	79
Mammary gland	Alfalfa hay	Negative	cell death of embryonic cells	4.59E-04	30
Mammary	Alfalfa	Negative	apoptosis of phagocytes	4.70E-04	44

gland	hay				
Mammary gland	Alfalfa hay	Negative	transmigration of cells	4.70E-04	38
Mammary gland	Alfalfa hay	Negative	organization of organelle	5.11E-04	108
Mammary gland	Alfalfa hay	Negative	secretion of molecule	5.43E-04	110
Mammary gland	Alfalfa hay	Negative	homing of cells	5.75E-04	111
Mammary gland	Alfalfa hay	Negative	development of hematopoietic system	5.94E-04	55
Mammary gland	Alfalfa hay	Negative	long-term potentiation	5.99E-04	54
Mammary gland	Alfalfa hay	Negative	proliferation of lymphatic system cells	6.01E-04	46
Mammary gland	Alfalfa hay	Negative	degeneration of nervous system	6.01E-04	62
Mammary gland	Alfalfa hay	Negative	morphology of head	6.03E-04	166
Mammary gland	Alfalfa hay	Negative	migration of mesenchymal stem cells	6.21E-04	11
Mammary gland	Alfalfa hay	Negative	cellular infiltration of cells	6.27E-04	82
Mammary gland	Alfalfa hay	Negative	quantity of embryonic cells	6.28E-04	22
Mammary gland	Alfalfa hay	Negative	cell movement of endothelial cells	6.71E-04	74
Mammary gland	Alfalfa hay	Negative	proliferation of epithelial cells	6.76E-04	99
Mammary gland	Alfalfa hay	Negative	cell movement of muscle cells	6.76E-04	42
Mammary gland	Alfalfa hay	Negative	migration of muscle cells	6.80E-04	39
Mammary gland	Alfalfa hay	Negative	cell movement of phagocytes	6.80E-04	109
Mammary gland	Alfalfa hay	Negative	transactivation of RNA	6.80E-04	108
Mammary gland	Alfalfa hay	Negative	proliferation of muscle cells	7.10E-04	77
Mammary gland	Alfalfa hay	Negative	cell death of myeloid cells	7.10E-04	48
Mammary gland	Alfalfa hay	Negative	cell death of kidney cells	7.28E-04	73
Mammary	Alfalfa	Negative	autosomal recessive disease	7.31E-04	173

gland	hay				
Mammary gland	Alfalfa hay	Negative	protein kinase cascade	7.31E-04	86
Mammary gland	Alfalfa hay	Negative	accumulation of macrophages	7.31E-04	22
Mammary gland	Alfalfa hay	Negative	cell cycle progression	7.31E-04	194
Mammary gland	Alfalfa hay	Negative	formation of filopodia	7.35E-04	39
Mammary gland	Alfalfa hay	Negative	morphology of connective tissue	7.35E-04	93
Mammary gland	Alfalfa hay	Negative	homing	7.64E-04	113
Mammary gland	Alfalfa hay	Negative	binding of mononuclear leukocytes	7.85E-04	29
Mammary gland	Alfalfa hay	Negative	quantity of mononuclear leukocytes	7.91E-04	141
Mammary gland	Alfalfa hay	Negative	transport of L-amino acid	7.92E-04	19
Mammary gland	Alfalfa hay	Negative	apoptosis of connective tissue cells	7.95E-04	63
Mammary gland	Alfalfa hay	Negative	cell death of cervical cancer cell lines	7.95E-04	73
Mammary gland	Alfalfa hay	Negative	concentration of fatty acid	7.95E-04	60
Mammary gland	Alfalfa hay	Negative	morphology of reproductive system	7.95E-04	106
Mammary gland	Alfalfa hay	Negative	proliferation of neuronal cells	8.78E-04	113
Mammary gland	Alfalfa hay	Negative	apoptosis of myeloid cells	8.82E-04	43
Mammary gland	Alfalfa hay	Negative	morphogenesis of neurites	8.93E-04	85
Mammary gland	Alfalfa hay	Negative	epileptic seizure	8.95E-04	39
Mammary gland	Alfalfa hay	Negative	Bleeding	8.96E-04	86
Mammary gland	Alfalfa hay	Negative	binding of endothelial cells	9.21E-04	27
Mammary gland	Alfalfa hay	Negative	oral cancer	9.64E-04	50
Mammary gland	Alfalfa hay	Negative	morphology of body cavity	1.07E-03	217
Mammary	Alfalfa	Negative	cell death of macrophages	1.07E-03	31

gland	hay				
Mammary gland	Alfalfa hay	Negative	apoptosis of macrophages	1.07E-03	27
Mammary gland	Alfalfa hay	Negative	formation of vessel component	1.07E-03	20
Mammary gland	Alfalfa hay	Negative	development of neurons	1.13E-03	148
Mammary gland	Alfalfa hay	Negative	cell transformation	1.13E-03	92
Mammary gland	Alfalfa hay	Negative	congenital malformation of brain	1.15E-03	63
Mammary gland	Alfalfa hay	Negative	migration of smooth muscle cells	1.22E-03	35
Mammary gland	Alfalfa hay	Negative	chronic myeloid leukemia	1.25E-03	27
Mammary gland	Alfalfa hay	Negative	accumulation of antigen presenting cells	1.26E-03	23
Mammary gland	Alfalfa hay	Negative	accumulation of myeloid cells	1.26E-03	40
Mammary gland	Alfalfa hay	Negative	activation of antigen presenting cells	1.28E-03	66
Mammary gland	Alfalfa hay	Negative	quantity of cellular protrusions	1.30E-03	37
Mammary gland	Alfalfa hay	Negative	secretory pathway	1.30E-03	44
Mammary gland	Alfalfa hay	Negative	survival of organism	1.30E-03	134
Mammary gland	Alfalfa hay	Negative	synthesis of fatty acid	1.31E-03	65
Mammary gland	Alfalfa hay	Negative	hypersensitive reaction	1.34E-03	86
Mammary gland	Alfalfa hay	Negative	abnormal morphology of head	1.34E-03	156
Mammary gland	Alfalfa hay	Negative	hypoplasia of organ	1.37E-03	89
Mammary gland	Alfalfa hay	Negative	accumulation of blood cells	1.38E-03	59
Mammary gland	Alfalfa hay	Negative	abnormal morphology of reproductive system	1.40E-03	100
Mammary gland	Alfalfa hay	Negative	cell death of brain	1.51E-03	61
Mammary gland	Alfalfa hay	Negative	oral squamous cell carcinoma	1.52E-03	40
Mammary	Alfalfa	Negative	quantity of interleukin	1.53E-03	33

gland	hay				
Mammary gland	Alfalfa hay	Negative	chemotaxis of cells	1.54E-03	103
Mammary gland	Alfalfa hay	Negative	cell movement of vascular smooth muscle cells	1.55E-03	28
Mammary gland	Alfalfa hay	Negative	phosphorylation of protein	1.55E-03	139
Mammary gland	Alfalfa hay	Negative	cell movement of smooth muscle cells	1.57E-03	37
Mammary gland	Alfalfa hay	Negative	chemotaxis	1.59E-03	106
Mammary gland	Alfalfa hay	Negative	cognition	1.60E-03	90
Mammary gland	Alfalfa hay	Negative	congenital anomaly of musculoskeletal system	1.60E-03	139
Mammary gland	Alfalfa hay	Negative	necrosis of kidney	1.61E-03	74
Mammary gland	Alfalfa hay	Negative	killing of cells	1.62E-03	47
Mammary gland	Alfalfa hay	Negative	activation of DNA endogenous promoter	1.62E-03	218
Mammary gland	Alfalfa hay	Negative	apoptosis of cervical cancer cell lines	1.69E-03	59
Mammary gland	Alfalfa hay	Negative	development of connective tissue	1.74E-03	75
Mammary gland	Alfalfa hay	Negative	formation of vascular lesion	1.74E-03	28
Mammary gland	Alfalfa hay	Negative	polyneuropathy	1.81E-03	27
Mammary gland	Alfalfa hay	Negative	apoptosis of hepatoma cell lines	1.81E-03	35
Mammary gland	Alfalfa hay	Negative	activation of enzyme	1.81E-03	78
Mammary gland	Alfalfa hay	Negative	cell movement of antigen presenting cells	1.82E-03	71
Mammary gland	Alfalfa hay	Negative	skin abnormality	1.85E-03	49
Mammary gland	Alfalfa hay	Negative	Hypertrophy	1.86E-03	93
Mammary gland	Alfalfa hay	Negative	synthesis of DNA	1.89E-03	83
Mammary gland	Alfalfa hay	Negative	binding of lymphocytes	1.92E-03	24
Mammary	Alfalfa	Negative	Dermatitis	1.92E-03	86

gland	hay				
Mammary gland	Alfalfa hay	Negative	response of mononuclear leukocytes	1.96E-03	50
Mammary gland	Alfalfa hay	Negative	development of leukocytes	1.96E-03	113
Mammary gland	Alfalfa hay	Negative	exocytosis	1.96E-03	42
Mammary gland	Alfalfa hay	Negative	cell movement of breast cancer cell lines	1.98E-03	60
Mammary gland	Alfalfa hay	Negative	migration of endothelial cells	1.98E-03	67
Mammary gland	Alfalfa hay	Negative	formation of neointima	1.98E-03	19
Mammary gland	Alfalfa hay	Negative	cell death of brain cells	2.02E-03	57
Mammary gland	Alfalfa hay	Negative	abnormal morphology of zona fasciculata	2.03E-03	7
Mammary gland	Alfalfa hay	Negative	activation of female germ cells	2.04E-03	6
Mammary gland	Alfalfa hay	Negative	development of lymphatic system	2.04E-03	72
Mammary gland	Alfalfa hay	Negative	formation of thymocytes	2.06E-03	26
Mammary gland	Alfalfa hay	Negative	cell death of cerebral cortex cells	2.12E-03	49
Mammary gland	Alfalfa hay	Negative	apoptosis of epithelial cell lines	2.16E-03	48
Mammary gland	Alfalfa hay	Negative	formation of T lymphocytes	2.17E-03	28
Mammary gland	Alfalfa hay	Negative	cell death of embryonic cell lines	2.19E-03	51
Mammary gland	Alfalfa hay	Negative	morphology of nervous system	2.21E-03	146
Mammary gland	Alfalfa hay	Negative	differentiation of leukocytes	2.22E-03	127
Mammary gland	Alfalfa hay	Negative	binding of leukocytes	2.31E-03	40
Mammary gland	Alfalfa hay	Negative	quantity of metal ion	2.33E-03	87
Mammary gland	Alfalfa hay	Negative	abnormal morphology of skull	2.38E-03	47
Mammary gland	Alfalfa hay	Negative	proliferation of vascular smooth muscle cells	2.39E-03	35
Mammary	Alfalfa	Negative	vascularization	2.42E-03	50

gland	hay				
Mammary gland	Alfalfa hay	Negative	cell death of gonadal cell lines	2.45E-03	20
Mammary gland	Alfalfa hay	Negative	glomerulosclerosis	2.45E-03	30
Mammary gland	Alfalfa hay	Negative	cell movement of neutrophils	2.45E-03	61
Mammary gland	Alfalfa hay	Negative	cell movement of granulocytes	2.48E-03	74
Mammary gland	Alfalfa hay	Negative	cell viability of carcinoma cell lines	2.48E-03	33
Mammary gland	Alfalfa hay	Negative	synthesis of phospholipid	2.48E-03	39
Mammary gland	Alfalfa hay	Negative	quantity of IL-6 in blood	2.51E-03	22
Mammary gland	Alfalfa hay	Negative	cell death of central nervous system cells	2.51E-03	60
Mammary gland	Alfalfa hay	Negative	formation of lymphatic system component	2.51E-03	60
Mammary gland	Alfalfa hay	Negative	ubiquitination	2.51E-03	68
Mammary gland	Alfalfa hay	Negative	development of lymphatic system component	2.53E-03	63
Mammary gland	Alfalfa hay	Negative	transport of glutamine family amino acid	2.53E-03	16
Mammary gland	Alfalfa hay	Negative	development of genitourinary system	2.53E-03	163
Mammary gland	Alfalfa hay	Negative	cell viability of muscle cells	2.55E-03	18
Mammary gland	Alfalfa hay	Negative	formation of lymphoid organ	2.55E-03	55
Mammary gland	Alfalfa hay	Negative	allergy	2.55E-03	80
Mammary gland	Alfalfa hay	Negative	proliferation of hepatoma cell lines	2.63E-03	43
Mammary gland	Alfalfa hay	Negative	killing of macrophages	2.63E-03	8
Mammary gland	Alfalfa hay	Negative	fatty acid metabolism	2.74E-03	114
Mammary gland	Alfalfa hay	Negative	ruffling of plasma membrane	2.79E-03	14
Mammary gland	Alfalfa hay	Negative	damage of cellular membrane	2.82E-03	9
Mammary	Alfalfa	Negative	homeostasis of leukocytes	2.82E-03	106

gland	hay				
Mammary gland	Alfalfa hay	Negative	apoptosis of pheochromocytoma cell lines	2.82E-03	23
Mammary gland	Alfalfa hay	Negative	learning	2.88E-03	82
Mammary gland	Alfalfa hay	Negative	cell movement of fibroblasts	2.92E-03	40
Mammary gland	Alfalfa hay	Negative	transport of metal	2.92E-03	67
Mammary gland	Alfalfa hay	Negative	heart rate	2.93E-03	57
Mammary gland	Alfalfa hay	Negative	apoptosis of melanoma cell lines	2.97E-03	30
Mammary gland	Alfalfa hay	Negative	reorganization of actin cytoskeleton	2.97E-03	30
Mammary gland	Alfalfa hay	Negative	development of head	2.97E-03	178
Mammary gland	Alfalfa hay	Negative	infection by Retroviridae	2.98E-03	134
Mammary gland	Alfalfa hay	Negative	apoptosis of neurons	3.02E-03	85
Mammary gland	Alfalfa hay	Negative	cell movement of carcinoma cell lines	3.02E-03	44
Mammary gland	Alfalfa hay	Negative	inflammatory response	3.04E-03	144
Mammary gland	Alfalfa hay	Negative	cell movement of connective tissue cells	3.10E-03	47
Mammary gland	Alfalfa hay	Negative	cell death of antigen presenting cells	3.10E-03	36
Mammary gland	Alfalfa hay	Negative	abnormal morphology of body cavity	3.13E-03	204
Mammary gland	Alfalfa hay	Negative	binding of T lymphocytes	3.13E-03	19
Mammary gland	Alfalfa hay	Negative	inflammation of blood vessel	3.13E-03	11
Mammary gland	Alfalfa hay	Negative	cell death of melanoma cell lines	3.14E-03	52
Mammary gland	Alfalfa hay	Negative	metabolism of phospholipid	3.16E-03	49
Mammary gland	Alfalfa hay	Negative	transport of ion	3.20E-03	97
Mammary gland	Alfalfa hay	Negative	transport of cation	3.25E-03	79
Mammary	Alfalfa	Negative	formation of vesicles	3.30E-03	28

gland	hay				
Mammary gland	Alfalfa hay	Negative	morphology of endothelial cells	3.31E-03	17
Mammary gland	Alfalfa hay	Negative	necrosis of tumor	3.34E-03	75
Mammary gland	Alfalfa hay	Negative	transport of inorganic cation	3.35E-03	70
Mammary gland	Alfalfa hay	Negative	apoptosis of cardiomyocytes	3.37E-03	40
Mammary gland	Alfalfa hay	Negative	organization of actin filaments	3.37E-03	25
Mammary gland	Alfalfa hay	Negative	quantity of carbohydrate	3.45E-03	99
Mammary gland	Alfalfa hay	Negative	cell movement of microglia	3.45E-03	13
Mammary gland	Alfalfa hay	Negative	airway hyperresponsiveness	3.46E-03	27
Mammary gland	Alfalfa hay	Negative	epilepsy	3.47E-03	62
Mammary gland	Alfalfa hay	Negative	activation of Protein kinase	3.60E-03	52
Mammary gland	Alfalfa hay	Negative	morphology of blood cells	3.61E-03	81
Mammary gland	Alfalfa hay	Negative	development of blood cells	3.61E-03	122
Mammary gland	Alfalfa hay	Negative	transport of metal ion	3.61E-03	64
Mammary gland	Alfalfa hay	Negative	invasion of squamous cell carcinoma cell lines	3.61E-03	16
Mammary gland	Alfalfa hay	Negative	activation of oocytes	3.63E-03	5
Mammary gland	Alfalfa hay	Negative	formation of cytoskeleton	3.63E-03	74
Mammary gland	Alfalfa hay	Negative	proliferation of blood cells	3.65E-03	154
Mammary gland	Alfalfa hay	Negative	central nervous system tumor	3.65E-03	166
Mammary gland	Alfalfa hay	Negative	proliferation of lung cancer cell lines	3.66E-03	55
Mammary gland	Alfalfa hay	Negative	uptake of monosaccharide	3.66E-03	55
Mammary gland	Alfalfa hay	Negative	cognitive impairment	3.68E-03	66
Mammary	Alfalfa	Negative	differentiation of epithelial tissue	3.70E-03	71

gland	hay				
Mammary gland	Alfalfa hay	Negative	invasion of lung cancer cell lines	3.73E-03	25
Mammary gland	Alfalfa hay	Negative	migration of vascular smooth muscle cells	3.73E-03	25
Mammary gland	Alfalfa hay	Negative	cell viability of lung cancer cell lines	3.81E-03	27
Mammary gland	Alfalfa hay	Negative	synthesis of reactive oxygen species	3.84E-03	92
Mammary gland	Alfalfa hay	Negative	Neurodegeneration	3.84E-03	62
Mammary gland	Alfalfa hay	Negative	ubiquitination of protein	3.88E-03	66
Mammary gland	Alfalfa hay	Negative	growth of tumor	3.88E-03	146
Mammary gland	Alfalfa hay	Negative	migration of connective tissue cells	3.91E-03	39
Mammary gland	Alfalfa hay	Negative	cell death of hepatoma cell lines	3.93E-03	37
Mammary gland	Alfalfa hay	Negative	arthropathy	4.00E-03	172
Mammary gland	Alfalfa hay	Negative	accumulation of phagocytes	4.14E-03	32
Mammary gland	Alfalfa hay	Negative	quantity of metal	4.14E-03	95
Mammary gland	Alfalfa hay	Negative	development of abdomen	4.16E-03	109
Mammary gland	Alfalfa hay	Negative	morphology of gonad	4.21E-03	74
Mammary gland	Alfalfa hay	Negative	Lymphocyte homeostasis	4.24E-03	103
Mammary gland	Alfalfa hay	Negative	apoptosis of embryonic cell lines	4.24E-03	39
Mammary gland	Alfalfa hay	Negative	migration of mesenchymal cells	4.24E-03	12
Mammary gland	Alfalfa hay	Negative	quantity of diacylglycerol	4.24E-03	16
Mammary gland	Alfalfa hay	Negative	quantity of antigen presenting cells	4.24E-03	56
Mammary gland	Alfalfa hay	Negative	quantity of myeloid cells	4.26E-03	70
Mammary gland	Alfalfa hay	Negative	morphology of genital organ	4.26E-03	82
Mammary	Alfalfa	Negative	arthritis	4.33E-03	169

gland	hay				
Mammary gland	Alfalfa hay	Negative	development of body axis	4.33E-03	188
Mammary gland	Alfalfa hay	Negative	necrosis of liver	4.33E-03	49
Mammary gland	Alfalfa hay	Negative	hemolysis	4.33E-03	24
Mammary gland	Alfalfa hay	Negative	development of epithelial tissue	4.34E-03	98
Mammary gland	Alfalfa hay	Negative	metabolism of reactive oxygen species	4.45E-03	95
Mammary gland	Alfalfa hay	Negative	size of embryo	4.48E-03	70
Mammary gland	Alfalfa hay	Negative	shape change of kidney cell lines	4.49E-03	17
Mammary gland	Alfalfa hay	Negative	cell movement of macrophages	4.51E-03	54
Mammary gland	Alfalfa hay	Negative	cancer of cells	4.51E-03	52
Mammary gland	Alfalfa hay	Negative	fibrosis of heart	4.54E-03	39
Mammary gland	Alfalfa hay	Negative	engulfment of leukocytes	4.59E-03	35
Mammary gland	Alfalfa hay	Negative	migration of myeloid cells	4.59E-03	36
Mammary gland	Alfalfa hay	Negative	hereditary motor and sensory neuropathy	4.59E-03	22
Mammary gland	Alfalfa hay	Negative	apoptosis of kidney cell lines	4.59E-03	49
Mammary gland	Alfalfa hay	Negative	inflammation of organ	4.61E-03	208
Mammary gland	Alfalfa hay	Negative	metastasis of cells	4.61E-03	51
Mammary gland	Alfalfa hay	Negative	homo-oligomerization of protein	4.65E-03	30
Mammary gland	Alfalfa hay	Negative	killing of tumor cell lines	4.66E-03	21
Mammary gland	Alfalfa hay	Negative	apoptosis of antigen presenting cells	4.78E-03	31
Mammary gland	Alfalfa hay	Negative	generation of mononuclear leukocytes	4.88E-03	32
Mammary gland	Alfalfa hay	Negative	HIV infection	4.90E-03	131
Mammary	Alfalfa	Negative	invasion of ovarian cancer cell lines	4.92E-03	14

gland	hay				
Mammary gland	Alfalfa hay	Negative	colony formation	4.97E-03	101
Mammary gland	Alfalfa hay	Negative	differentiation of blood cells	4.97E-03	153
Mammary gland	Alfalfa hay	Negative	migration of vascular endothelial cells	4.97E-03	36
Mammary gland	Alfalfa hay	Negative	branching of cells	4.97E-03	72
Mammary gland	Alfalfa hay	Negative	mental retardation	5.05E-03	48
Mammary gland	Alfalfa hay	Negative	accumulation of leukocytes	5.05E-03	54
Mammary gland	Alfalfa hay	Negative	cell death of leukemia cell lines	5.05E-03	63
Mammary gland	Alfalfa hay	Negative	apoptosis of carcinoma cell lines	5.05E-03	50
Mammary gland	Alfalfa hay	Negative	hypertrophy of tissue	5.05E-03	52
Mammary gland	Alfalfa hay	Negative	degranulation of mast cells	5.10E-03	30
Mammary gland	Alfalfa hay	Negative	uptake of 2-deoxyglucose	5.13E-03	25
Mammary gland	Alfalfa hay	Negative	peripheral arterial disease	5.18E-03	44
Mammary gland	Alfalfa hay	Negative	differentiation of connective tissue cells	5.20E-03	119
Mammary gland	Alfalfa hay	Negative	abnormal morphology of retinal pigment epithelium	5.21E-03	12
Mammary gland	Alfalfa hay	Negative	quantity of trophoblast cells	5.21E-03	12
Mammary gland	Alfalfa hay	Negative	size of animal	5.27E-03	38
Mammary gland	Alfalfa hay	Negative	cell death of hippocampal neurons	5.27E-03	21
Mammary gland	Alfalfa hay	Negative	infection of cells	5.28E-03	145
Mammary gland	Alfalfa hay	Negative	chemoattraction of natural killer cells	5.28E-03	4
Mammary gland	Alfalfa hay	Negative	osmotic water permeability of cells	5.28E-03	4
Mammary gland	Alfalfa hay	Negative	polarization of B-lymphocyte derived cell lines	5.28E-03	4
Mammary	Alfalfa	Negative	syndromic mental retardation	5.28E-03	34

gland	hay				
Mammary gland	Alfalfa hay	Negative	generation of leukocytes	5.28E-03	35
Mammary gland	Alfalfa hay	Negative	migration of breast cancer cell lines	5.28E-03	50
Mammary gland	Alfalfa hay	Negative	differentiation of muscle	5.28E-03	60
Mammary gland	Alfalfa hay	Negative	cell death of carcinoma cell lines	5.28E-03	57
Mammary gland	Alfalfa hay	Negative	apoptosis of fibroblasts	5.31E-03	45
Mammary gland	Alfalfa hay	Negative	cell death of liver cells	5.37E-03	41
Mammary gland	Alfalfa hay	Negative	oral cavity carcinoma	5.37E-03	41
Mammary gland	Alfalfa hay	Negative	fibrogenesis	5.42E-03	79
Mammary gland	Alfalfa hay	Negative	chronic inflammatory disorder	5.56E-03	174
Mammary gland	Alfalfa hay	Negative	cell death of kidney cell lines	5.60E-03	60
Mammary gland	Alfalfa hay	Negative	apoptosis of microglia	5.60E-03	8
Mammary gland	Alfalfa hay	Negative	adhesion of mononuclear leukocytes	5.69E-03	32
Mammary gland	Alfalfa hay	Negative	cell death of endothelial cells	5.70E-03	42
Mammary gland	Alfalfa hay	Negative	serous neoplasm	5.77E-03	113
Mammary gland	Alfalfa hay	Negative	exencephaly	5.77E-03	28
Mammary gland	Alfalfa hay	Negative	targeting of protein	5.98E-03	29
Mammary gland	Alfalfa hay	Negative	migration of tumor cells	6.00E-03	46
Mammary gland	Alfalfa hay	Negative	abnormal morphology of joint	6.00E-03	17
Mammary gland	Alfalfa hay	Negative	outgrowth of plasma membrane projections	6.00E-03	78
Mammary gland	Alfalfa hay	Negative	extension of cellular protrusions	6.00E-03	43
Mammary gland	Alfalfa hay	Negative	attachment of cells	6.08E-03	30
Mammary	Alfalfa	Negative	transport of L-glutamic acid	6.10E-03	10

gland	hay				
Mammary gland	Alfalfa hay	Negative	generation of lymphocytes	6.17E-03	31
Mammary gland	Alfalfa hay	Negative	cell death of neuroglia	6.17E-03	27
Mammary gland	Alfalfa hay	Negative	proliferation of myeloid cells	6.17E-03	27
Mammary gland	Alfalfa hay	Negative	quantity of T lymphocytes	6.22E-03	100
Mammary gland	Alfalfa hay	Negative	septic shock	6.24E-03	33
Mammary gland	Alfalfa hay	Negative	adhesion of tumor cell lines	6.32E-03	57
Mammary gland	Alfalfa hay	Negative	adenocarcinoma	6.34E-03	1041
Mammary gland	Alfalfa hay	Negative	quantity of granulocytes	6.43E-03	62
Mammary gland	Alfalfa hay	Negative	development of macrophages	6.43E-03	12
Mammary gland	Alfalfa hay	Negative	morphology of filaments	6.43E-03	11
Mammary gland	Alfalfa hay	Negative	cell death of granulocytes	6.47E-03	22
Mammary gland	Alfalfa hay	Negative	development of myeloid cells	6.47E-03	22
Mammary gland	Alfalfa hay	Negative	formation of muscle	6.57E-03	76
Mammary gland	Alfalfa hay	Negative	differentiation of embryonic tissue	6.57E-03	51
Mammary gland	Alfalfa hay	Negative	quantity of cytokine	6.57E-03	51
Mammary gland	Alfalfa hay	Negative	morphology of cardiovascular tissue	6.69E-03	21
Mammary gland	Alfalfa hay	Negative	metabolism of phosphatidic acid	6.69E-03	36
Mammary gland	Alfalfa hay	Negative	formation of blood cells	6.69E-03	41
Mammary gland	Alfalfa hay	Negative	quantity of B lymphocytes	6.69E-03	67
Mammary gland	Alfalfa hay	Negative	formation of leukocytes	6.77E-03	34
Mammary gland	Alfalfa hay	Negative	branching of neurites	6.77E-03	55
Mammary	Alfalfa	Negative	demyelination	6.77E-03	27

gland	hay				
Mammary gland	Alfalfa hay	Negative	development of hematopoietic progenitor cells	6.80E-03	40
Mammary gland	Alfalfa hay	Negative	sprouting	6.83E-03	74
Mammary gland	Alfalfa hay	Negative	removal of cells	6.92E-03	23
Mammary gland	Alfalfa hay	Negative	organic aciduria	6.92E-03	14
Mammary gland	Alfalfa hay	Negative	phosphorylation of carbohydrate	6.92E-03	14
Mammary gland	Alfalfa hay	Negative	cell viability of cervical cancer cell lines	6.92E-03	45
Mammary gland	Alfalfa hay	Negative	proliferation of immune cells	6.93E-03	143
Mammary gland	Alfalfa hay	Negative	anemia	6.99E-03	67
Mammary gland	Alfalfa hay	Negative	movement of vascular endothelial cells	7.00E-03	38
Mammary gland	Alfalfa hay	Negative	fertility	7.07E-03	70
Mammary gland	Alfalfa hay	Negative	export of molecule	7.07E-03	58
Mammary gland	Alfalfa hay	Negative	S phase	7.07E-03	52
Mammary gland	Alfalfa hay	Negative	outgrowth of neurites	7.09E-03	77
Mammary gland	Alfalfa hay	Negative	engulfment of blood cells	7.16E-03	36
Mammary gland	Alfalfa hay	Negative	morphology of uterus	7.19E-03	22
Mammary gland	Alfalfa hay	Negative	migration of fibroblasts	7.24E-03	31
Mammary gland	Alfalfa hay	Negative	abnormal morphology of gonad	7.46E-03	70
Mammary gland	Alfalfa hay	Negative	ion homeostasis of cells	7.51E-03	103
Mammary gland	Alfalfa hay	Negative	abnormal morphology of neurosensory retina	7.51E-03	21
Mammary gland	Alfalfa hay	Negative	shape change of neurites	7.76E-03	56
Mammary gland	Alfalfa hay	Negative	infection by HIV-1	7.77E-03	112
Mammary	Alfalfa	Negative	permeability of cells	7.77E-03	23

gland	hay				
Mammary gland	Alfalfa hay	Negative	morphology of endothelial tissue	7.77E-03	20
Mammary gland	Alfalfa hay	Negative	activation of neuroglia	7.81E-03	29
Mammary gland	Alfalfa hay	Negative	internalization of cells	7.88E-03	35
Mammary gland	Alfalfa hay	Negative	synthesis of phosphatidic acid	7.88E-03	30
Mammary gland	Alfalfa hay	Negative	necrosis of glioma	7.88E-03	12
Mammary gland	Alfalfa hay	Negative	apoptosis of seminiferous tubule cells	7.88E-03	5
Mammary gland	Alfalfa hay	Negative	development of connective tissue cells	7.88E-03	45
Mammary gland	Alfalfa hay	Negative	abnormal morphology of PNS glial cells	7.88E-03	8
Mammary gland	Alfalfa hay	Negative	cell death of blood cells	7.88E-03	126
Mammary gland	Alfalfa hay	Negative	migration of granulocytes	7.88E-03	31
Mammary gland	Alfalfa hay	Negative	degranulation of phagocytes	7.88E-03	33
Mammary gland	Alfalfa hay	Negative	aciduria	7.88E-03	17
Mammary gland	Alfalfa hay	Negative	differentiation of smooth muscle	7.88E-03	17
Mammary gland	Alfalfa hay	Negative	thoracic neoplasm	7.88E-03	199
Mammary gland	Alfalfa hay	Negative	apoptosis of epithelial cells	8.05E-03	56
Mammary gland	Alfalfa hay	Negative	apoptosis of tumor cells	8.05E-03	56
Mammary gland	Alfalfa hay	Negative	apoptosis of testicular cells	8.05E-03	7
Mammary gland	Alfalfa hay	Negative	retinal degeneration	8.24E-03	51
Mammary gland	Alfalfa hay	Negative	activation of cells	8.24E-03	173
Mammary gland	Alfalfa hay	Negative	formation of hematopoietic progenitor cells	8.33E-03	28
Mammary gland	Alfalfa hay	Negative	Shock Response	8.33E-03	36
Mammary	Alfalfa	Negative	apoptosis of granulocytes	8.40E-03	21

gland	hay				
Mammary gland	Alfalfa hay	Negative	transport of divalent cations	8.43E-03	35
Mammary gland	Alfalfa hay	Negative	abnormal morphology of nervous system	8.53E-03	131
Mammary gland	Alfalfa hay	Negative	branching of neurons	8.54E-03	56
Mammary gland	Alfalfa hay	Negative	hereditary polyneuropathy	8.56E-03	23
Mammary gland	Alfalfa hay	Negative	growth of skin	8.62E-03	44
Mammary gland	Alfalfa hay	Negative	breast or colorectal cancer	8.67E-03	802
Mammary gland	Alfalfa hay	Negative	shape change of plasma membrane	8.72E-03	15
Mammary gland	Alfalfa hay	Negative	orientation of cells	8.87E-03	37
Mammary gland	Alfalfa hay	Negative	degeneration of cells	8.87E-03	66
Mammary gland	Alfalfa hay	Negative	quantity of Ca2+	8.87E-03	77
Mammary gland	Alfalfa hay	Negative	formation of osteoclasts	8.99E-03	27
Mammary gland	Alfalfa hay	Negative	permeability of blood vessel	9.04E-03	22
Mammary gland	Alfalfa hay	Negative	growth of plasma membrane projections	9.14E-03	87
Mammary gland	Alfalfa hay	Negative	arrest in growth of organism	9.14E-03	35
Mammary gland	Alfalfa hay	Negative	development of antigen presenting cells	9.15E-03	17
Mammary gland	Alfalfa hay	Negative	melanoma	9.16E-03	1059
Mammary gland	Alfalfa hay	Negative	dendritic growth/branching	9.20E-03	44
Mammary gland	Alfalfa hay	Negative	formation of lamellipodia	9.20E-03	34
Mammary gland	Alfalfa hay	Negative	transport of amino acids	9.23E-03	29
Mammary gland	Alfalfa hay	Negative	abnormal morphology of genital organ	9.24E-03	77
Mammary gland	Alfalfa hay	Negative	female genital tract serous cancer	9.35E-03	73
Mammary	Alfalfa	Negative	inflammation of artery	9.36E-03	9

gland	hay				
Mammary gland	Alfalfa hay	Negative	congenital anomaly of skin	9.55E-03	37
Mammary gland	Alfalfa hay	Negative	abnormal morphology of vascular endothelial cells	9.60E-03	12
Mammary gland	Alfalfa hay	Negative	allodynia	9.76E-03	14
Mammary gland	Alfalfa hay	Negative	phagocytosis of neutrophils	9.76E-03	14
Mammary gland	Alfalfa hay	Negative	benign pelvic disease	9.76E-03	75
Mammary gland	Alfalfa hay	Negative	formation of filaments	9.76E-03	75
Mammary gland	Alfalfa hay	Negative	formation of eye	9.86E-03	82
Mammary gland	Alfalfa hay	Negative	infection by RNA virus	9.92E-03	154
Mammary gland	Alfalfa hay	Negative	cell movement of mononuclear leukocytes	9.98E-03	86
Mammary gland	Alfalfa hay	Negative	apoptosis of cerebral cortex cells	9.98E-03	24
Mammary gland	Alfalfa hay	Negative	quantity of trophoblast giant cells	9.98E-03	11
Mammary gland	Alfalfa hay	Negative	apoptosis of glioma cells	9.98E-03	10
Mammary gland	Rice straw	Positive	proliferation of cells	8.08E-19	851
Mammary gland	Rice straw	Positive	cell death	2.71E-18	777
Mammary gland	Rice straw	Positive	organization of cytoplasm	1.88E-17	403
Mammary gland	Rice straw	Positive	necrosis	5.70E-17	616
Mammary gland	Rice straw	Positive	organismal death	5.70E-17	580
Mammary gland	Rice straw	Positive	cancer	9.22E-17	2151
Mammary gland	Rice straw	Positive	morbidity or mortality	9.22E-17	585
Mammary gland	Rice straw	Positive	malignant solid tumor	1.15E-16	2127
Mammary gland	Rice straw	Positive	morphology of cells	1.68E-15	480
Mammary	Rice	Positive	apoptosis	3.53E-15	620

gland	straw				
Mammary gland	Rice straw	Positive	organization of cytoskeleton	4.34E-15	362
Mammary gland	Rice straw	Positive	epithelial cancer	6.26E-15	1776
Mammary gland	Rice straw	Positive	neoplasia of epithelial tissue	8.97E-15	1790
Mammary gland	Rice straw	Positive	cellular homeostasis	1.07E-14	387
Mammary gland	Rice straw	Positive	tumorigenesis of tissue	3.11E-14	1813
Mammary gland	Rice straw	Positive	quantity of cells	3.03E-13	432
Mammary gland	Rice straw	Positive	cell movement	3.79E-13	505
Mammary gland	Rice straw	Positive	Movement Disorders	1.17E-12	279
Mammary gland	Rice straw	Positive	migration of cells	2.46E-12	455
Mammary gland	Rice straw	Positive	dyskinesia	3.60E-12	177
Mammary gland	Rice straw	Positive	neurological signs	3.61E-12	185
Mammary gland	Rice straw	Positive	cell survival	3.74E-12	348
Mammary gland	Rice straw	Positive	disorder of basal ganglia	5.33E-12	211
Mammary gland	Rice straw	Positive	cell viability	1.25E-11	326
Mammary gland	Rice straw	Positive	abdominal neoplasm	2.20E-11	1755
Mammary gland	Rice straw	Positive	microtubule dynamics	3.86E-11	299
Mammary gland	Rice straw	Positive	differentiation of cells	4.53E-11	518
Mammary gland	Rice straw	Positive	Huntington's Disease	1.08E-10	162
Mammary gland	Rice straw	Positive	neuromuscular disease	1.91E-10	231
Mammary gland	Rice straw	Positive	cell death of tumor cell lines	2.13E-10	367
Mammary gland	Rice straw	Positive	abdominal cancer	2.15E-10	1729
Mammary	Rice	Positive	apoptosis of tumor cell lines	2.34E-10	301

gland	straw				
Mammary gland	Rice straw	Positive	quantity of leukocytes	6.12E-10	236
Mammary gland	Rice straw	Positive	quantity of blood cells	6.37E-10	260
Mammary gland	Rice straw	Positive	transport of molecule	9.87E-10	381
Mammary gland	Rice straw	Positive	transcription	1.23E-09	458
Mammary gland	Rice straw	Positive	expression of RNA	1.38E-09	488
Mammary gland	Rice straw	Positive	angiogenesis	2.92E-09	231
Mammary gland	Rice straw	Positive	formation of cellular protrusions	5.21E-09	229
Mammary gland	Rice straw	Positive	cell movement of tumor cell lines	2.53E-08	215
Mammary gland	Rice straw	Positive	transcription of RNA	3.27E-08	422
Mammary gland	Rice straw	Positive	abnormal morphology of cells	4.35E-08	301
Mammary gland	Rice straw	Positive	advanced malignant tumor	6.23E-08	212
Mammary gland	Rice straw	Positive	vasculogenesis	6.83E-08	188
Mammary gland	Rice straw	Positive	cell death of connective tissue cells	1.45E-07	157
Mammary gland	Rice straw	Positive	invasion of cells	1.79E-07	215
Mammary gland	Rice straw	Positive	digestive system cancer	2.33E-07	1488
Mammary gland	Rice straw	Positive	migration of tumor cell lines	2.44E-07	179
Mammary gland	Rice straw	Positive	development of cytoplasm	2.89E-07	124
Mammary gland	Rice straw	Positive	function of leukocytes	2.92E-07	141
Mammary gland	Rice straw	Positive	cell movement of blood cells	3.00E-07	215
Mammary gland	Rice straw	Positive	morphology of cardiovascular system	3.63E-07	159
Mammary gland	Rice straw	Positive	organization of organelle	3.73E-07	141
Mammary	Rice	Positive	migration of blood cells	3.76E-07	214

gland	straw				
Mammary gland	Rice straw	Positive	metabolism of carbohydrate	4.12E-07	159
Mammary gland	Rice straw	Positive	proliferation of fibroblasts	4.60E-07	98
Mammary gland	Rice straw	Positive	leukocyte migration	5.08E-07	213
Mammary gland	Rice straw	Positive	Organ Degeneration	5.11E-07	130
Mammary gland	Rice straw	Positive	digestive organ tumor	5.43E-07	1497
Mammary gland	Rice straw	Positive	formation of cytoskeleton	5.69E-07	103
Mammary gland	Rice straw	Positive	growth of connective tissue	7.11E-07	163
Mammary gland	Rice straw	Positive	cell viability of tumor cell lines	8.08E-07	192
Mammary gland	Rice straw	Positive	synthesis of lipid	8.25E-07	178
Mammary gland	Rice straw	Positive	benign neoplasia	9.16E-07	223
Mammary gland	Rice straw	Positive	invasion of tumor cell lines	9.46E-07	167
Mammary gland	Rice straw	Positive	cell movement of leukocytes	9.55E-07	189
Mammary gland	Rice straw	Positive	proliferation of connective tissue cells	9.66E-07	151
Mammary gland	Rice straw	Positive	development of leukocytes	9.90E-07	150
Mammary gland	Rice straw	Positive	proliferation of tumor cell lines	1.42E-06	349
Mammary gland	Rice straw	Positive	degeneration of cells	1.65E-06	94
Mammary gland	Rice straw	Positive	transcription of DNA	1.66E-06	346
Mammary gland	Rice straw	Positive	morphology of body cavity	1.74E-06	277
Mammary gland	Rice straw	Positive	concentration of lipid	1.82E-06	197
Mammary gland	Rice straw	Positive	ubiquitination	1.99E-06	92
Mammary gland	Rice straw	Positive	abnormal morphology of thoracic cavity	2.20E-06	148
Mammary	Rice	Positive	protein kinase cascade	2.38E-06	111

gland	straw				
Mammary gland	Rice straw	Positive	abnormal morphology of body cavity	2.76E-06	265
Mammary gland	Rice straw	Positive	ubiquitination of protein	3.50E-06	90
Mammary gland	Rice straw	Positive	function of blood cells	3.71E-06	147
Mammary gland	Rice straw	Positive	development of body trunk	3.76E-06	259
Mammary gland	Rice straw	Positive	proliferation of blood cells	3.76E-06	202
Mammary gland	Rice straw	Positive	cell transformation	3.81E-06	119
Mammary gland	Rice straw	Positive	quantity of lymphocytes	4.01E-06	173
Mammary gland	Rice straw	Positive	proliferation of immune cells	4.23E-06	191
Mammary gland	Rice straw	Positive	development of blood cells	4.32E-06	161
Mammary gland	Rice straw	Positive	binding of DNA	4.70E-06	132
Mammary gland	Rice straw	Positive	development of epithelial tissue	4.82E-06	131
Mammary gland	Rice straw	Positive	degeneration of nervous system	5.23E-06	79
Mammary gland	Rice straw	Positive	quantity of mononuclear leukocytes	6.23E-06	178
Mammary gland	Rice straw	Positive	cell movement of endothelial cells	6.23E-06	94
Mammary gland	Rice straw	Positive	metastasis	6.89E-06	183
Mammary gland	Rice straw	Positive	adenocarcinoma	7.32E-06	1299
Mammary gland	Rice straw	Positive	homing of cells	8.16E-06	139
Mammary gland	Rice straw	Positive	function of macrophages	8.62E-06	53
Mammary gland	Rice straw	Positive	abnormal morphology of cardiovascular system	8.74E-06	141
Mammary gland	Rice straw	Positive	abnormal morphology of epithelial tissue	8.93E-06	108
Mammary gland	Rice straw	Positive	growth of epithelial tissue	1.03E-05	165
Mammary	Rice	Positive	apoptosis of connective tissue cells	1.03E-05	80

gland	straw				
Mammary gland	Rice straw	Positive	growth of lesion	1.03E-05	190
Mammary gland	Rice straw	Positive	homing	1.03E-05	142
Mammary gland	Rice straw	Positive	advanced stage solid tumor	1.13E-05	129
Mammary gland	Rice straw	Positive	formation of eye	1.13E-05	112
Mammary gland	Rice straw	Positive	morphology of head	1.24E-05	206
Mammary gland	Rice straw	Positive	binding of protein binding site	1.25E-05	78
Mammary gland	Rice straw	Positive	development of mononuclear leukocytes	1.27E-05	138
Mammary gland	Rice straw	Positive	development of lymphocytes	1.31E-05	137
Mammary gland	Rice straw	Positive	growth of tumor	1.35E-05	189
Mammary gland	Rice straw	Positive	chemotaxis	1.40E-05	135
Mammary gland	Rice straw	Positive	chemotaxis of cells	1.51E-05	131
Mammary gland	Rice straw	Positive	Viral Infection	1.54E-05	354
Mammary gland	Rice straw	Positive	peripheral arterial disease	1.55E-05	60
Mammary gland	Rice straw	Positive	infection by Retroviridae	1.78E-05	172
Mammary gland	Rice straw	Positive	cell death of fibroblasts	1.78E-05	72
Mammary gland	Rice straw	Positive	Neurodegeneration	1.92E-05	82
Mammary gland	Rice straw	Positive	cell movement of fibroblasts	2.02E-05	53
Mammary gland	Rice straw	Positive	behavior	2.14E-05	228
Mammary gland	Rice straw	Positive	immune response of cells	2.23E-05	140
Mammary gland	Rice straw	Positive	congenital anomaly of musculoskeletal system	2.24E-05	175
Mammary gland	Rice straw	Positive	development of head	2.48E-05	226
Mammary	Rice	Positive	female genital neoplasm	2.49E-05	734

gland	straw				
Mammary gland	Rice straw	Positive	proliferation of mononuclear leukocytes	2.71E-05	175
Mammary gland	Rice straw	Positive	branching of cells	2.71E-05	95
Mammary gland	Rice straw	Positive	metabolism of membrane lipid derivative	2.73E-05	99
Mammary gland	Rice straw	Positive	quantity of phagocytes	2.92E-05	107
Mammary gland	Rice straw	Positive	fibrogenesis	3.17E-05	104
Mammary gland	Rice straw	Positive	movement of vascular endothelial cells	3.33E-05	52
Mammary gland	Rice straw	Positive	growth of organism	3.37E-05	195
Mammary gland	Rice straw	Positive	benign neoplasm of female genital organ	3.44E-05	107
Mammary gland	Rice straw	Positive	proliferation of lymphocytes	3.44E-05	172
Mammary gland	Rice straw	Positive	formation of vascular lesion	3.52E-05	36
Mammary gland	Rice straw	Positive	female genital tract serous cancer	3.56E-05	98
Mammary gland	Rice straw	Positive	arthropathy	3.56E-05	219
Mammary gland	Rice straw	Positive	infection by lentivirus	3.91E-05	169
Mammary gland	Rice straw	Positive	long-term potentiation	3.91E-05	66
Mammary gland	Rice straw	Positive	angiogenesis of lesion	4.03E-05	39
Mammary gland	Rice straw	Positive	necrosis of epithelial tissue	4.25E-05	142
Mammary gland	Rice straw	Positive	autosomal recessive disease	4.25E-05	212
Mammary gland	Rice straw	Positive	formation of actin stress fibers	4.26E-05	65
Mammary gland	Rice straw	Positive	cell death of epithelial cells	4.50E-05	122
Mammary gland	Rice straw	Positive	development of cardiovascular tissue	4.52E-05	94
Mammary gland	Rice straw	Positive	function of cardiovascular system	4.52E-05	94
Mammary	Rice	Positive	adenoma	4.67E-05	134

gland	straw				
Mammary gland	Rice straw	Positive	morphology of heart ventricle	4.67E-05	54
Mammary gland	Rice straw	Positive	inflammatory response	4.67E-05	182
Mammary gland	Rice straw	Positive	HIV infection	4.67E-05	168
Mammary gland	Rice straw	Positive	morphology of nervous system	4.68E-05	183
Mammary gland	Rice straw	Positive	function of antigen presenting cells	4.68E-05	66
Mammary gland	Rice straw	Positive	accumulation of cells	4.72E-05	90
Mammary gland	Rice straw	Positive	cellular degradation	4.78E-05	68
Mammary gland	Rice straw	Positive	formation of filaments	4.89E-05	100
Mammary gland	Rice straw	Positive	abnormal morphology of head	4.99E-05	193
Mammary gland	Rice straw	Positive	fatty acid metabolism	5.10E-05	144
Mammary gland	Rice straw	Positive	genital tumor	5.39E-05	823
Mammary gland	Rice straw	Positive	apoptosis of cervical cancer cell lines	5.46E-05	74
Mammary gland	Rice straw	Positive	endothelial cell development	5.55E-05	90
Mammary gland	Rice straw	Positive	colony formation	5.55E-05	130
Mammary gland	Rice straw	Positive	neuritogenesis	5.55E-05	140
Mammary gland	Rice straw	Positive	arthritis	6.03E-05	214
Mammary gland	Rice straw	Positive	activation of Protein kinase	6.54E-05	67
Mammary gland	Rice straw	Positive	cell death of cervical cancer cell lines	6.81E-05	89
Mammary gland	Rice straw	Positive	T cell homeostasis	7.32E-05	125
Mammary gland	Rice straw	Positive	cellular infiltration	7.38E-05	108
Mammary gland	Rice straw	Positive	accumulation of blood cells	7.43E-05	73
Mammary	Rice	Positive	T cell development	7.56E-05	122

gland	straw				
Mammary gland	Rice straw	Positive	cell movement of phagocytes	7.76E-05	132
Mammary gland	Rice straw	Positive	apoptosis of fibroblasts	7.94E-05	59
Mammary gland	Rice straw	Positive	formation of plasma membrane projections	8.10E-05	142
Mammary gland	Rice straw	Positive	development of endothelial tissue	8.20E-05	92
Mammary gland	Rice straw	Positive	differentiation of leukocytes	8.41E-05	158
Mammary gland	Rice straw	Positive	homeostasis of leukocytes	8.41E-05	133
Mammary gland	Rice straw	Positive	apoptosis of macrophages	8.45E-05	33
Mammary gland	Rice straw	Positive	abnormal morphology of nervous system	8.54E-05	169
Mammary gland	Rice straw	Positive	size of body	9.39E-05	195
Mammary gland	Rice straw	Positive	cognition	9.57E-05	111
Mammary gland	Rice straw	Positive	small GTPase mediated signal transduction	9.60E-05	51
Mammary gland	Rice straw	Positive	activation of cells	9.75E-05	221
Mammary gland	Rice straw	Positive	tumorigenesis of gonad	9.84E-05	186
Mammary gland	Rice straw	Positive	apoptosis of skin	9.87E-05	30
Mammary gland	Rice straw	Positive	biosynthesis of nucleoside triphosphate	1.01E-04	33
Mammary gland	Rice straw	Positive	sprouting	1.01E-04	96
Mammary gland	Rice straw	Positive	formation of actin filaments	1.02E-04	78
Mammary gland	Rice straw	Positive	infection by RNA virus	1.02E-04	198
Mammary gland	Rice straw	Positive	metastatic colorectal cancer	1.02E-04	53
Mammary gland	Rice straw	Positive	proliferation of T lymphocytes	1.02E-04	143
Mammary gland	Rice straw	Positive	necrosis of liver	1.02E-04	63
Mammary	Rice	Positive	morphology of heart	1.14E-04	100

gland	straw				
Mammary gland	Rice straw	Positive	cell death of fibrosarcoma cell lines	1.14E-04	25
Mammary gland	Rice straw	Positive	breast or ovarian cancer	1.14E-04	407
Mammary gland	Rice straw	Positive	function of myeloid cells	1.14E-04	63
Mammary gland	Rice straw	Positive	migration of connective tissue cells	1.14E-04	50
Mammary gland	Rice straw	Positive	cell movement of myeloid cells	1.15E-04	129
Mammary gland	Rice straw	Positive	cell viability of connective tissue cells	1.16E-04	39
Mammary gland	Rice straw	Positive	Lymphocyte homeostasis	1.17E-04	130
Mammary gland	Rice straw	Positive	hypersensitive reaction	1.19E-04	105
Mammary gland	Rice straw	Positive	function of phagocytes	1.19E-04	78
Mammary gland	Rice straw	Positive	advanced stage peripheral arterial disease	1.25E-04	43
Mammary gland	Rice straw	Positive	degeneration of neurons	1.25E-04	63
Mammary gland	Rice straw	Positive	proliferation of endothelial cells	1.28E-04	79
Mammary gland	Rice straw	Positive	ovarian cancer	1.28E-04	165
Mammary gland	Rice straw	Positive	development of body axis	1.29E-04	235
Mammary gland	Rice straw	Positive	vascular lesion	1.34E-04	60
Mammary gland	Rice straw	Positive	immune response of leukocytes	1.43E-04	78
Mammary gland	Rice straw	Positive	oral cavity carcinoma	1.44E-04	53
Mammary gland	Rice straw	Positive	seizures	1.46E-04	98
Mammary gland	Rice straw	Positive	cell movement of connective tissue cells	1.46E-04	59
Mammary gland	Rice straw	Positive	metastatic gastrointestinal tract cancer	1.46E-04	59
Mammary gland	Rice straw	Positive	oral cancer	1.48E-04	60
Mammary	Rice	Positive	learning	1.48E-04	102

gland	straw				
Mammary gland	Rice straw	Positive	atherogenesis	1.48E-04	26
Mammary gland	Rice straw	Positive	colony formation of cells	1.48E-04	118
Mammary gland	Rice straw	Positive	differentiation of blood cells	1.48E-04	192
Mammary gland	Rice straw	Positive	cell death of fibroblast cell lines	1.48E-04	104
Mammary gland	Rice straw	Positive	hereditary neuropathy	1.48E-04	46
Mammary gland	Rice straw	Positive	quantity of antigen presenting cells	1.48E-04	71
Mammary gland	Rice straw	Positive	activation of DNA endogenous promoter	1.51E-04	266
Mammary gland	Rice straw	Positive	gonadal tumor	1.51E-04	187
Mammary gland	Rice straw	Positive	Ovarian Cancer and Tumors	1.57E-04	174
Mammary gland	Rice straw	Positive	cell death of tumor	1.58E-04	94
Mammary gland	Rice straw	Positive	branching of neurons	1.59E-04	73
Mammary gland	Rice straw	Positive	female genital tract serous carcinoma	1.59E-04	54
Mammary gland	Rice straw	Positive	branching of neurites	1.61E-04	71
Mammary gland	Rice straw	Positive	cell death of lung cell lines	1.68E-04	18
Mammary gland	Rice straw	Positive	transmigration of cells	1.69E-04	44
Mammary gland	Rice straw	Positive	central nervous system tumor	1.71E-04	206
Mammary gland	Rice straw	Positive	cellular infiltration by leukocytes	1.79E-04	95
Mammary gland	Rice straw	Positive	migration of endothelial cells	1.79E-04	82
Mammary gland	Rice straw	Positive	formation of lamellipodia	1.79E-04	45
Mammary gland	Rice straw	Positive	cellular infiltration of cells	1.80E-04	97
Mammary gland	Rice straw	Positive	organization of actin cytoskeleton	1.81E-04	76
Mammary	Rice	Positive	synthesis of carbohydrate	1.85E-04	103

gland	straw				
Mammary gland	Rice straw	Positive	cell movement of mononuclear leukocytes	1.85E-04	111
Mammary gland	Rice straw	Positive	cell death of neuroblastoma cell lines	1.87E-04	52
Mammary gland	Rice straw	Positive	activation of enzyme	1.90E-04	95
Mammary gland	Rice straw	Positive	spatial learning	1.98E-04	50
Mammary gland	Rice straw	Positive	necrosis of tumor	2.22E-04	93
Mammary gland	Rice straw	Positive	neuronal cell death	2.33E-04	157
Mammary gland	Rice straw	Positive	shape change of neurites	2.33E-04	72
Mammary gland	Rice straw	Positive	metabolism of protein	2.33E-04	217
Mammary gland	Rice straw	Positive	cell death of tumor cells	2.34E-04	91
Mammary gland	Rice straw	Positive	apoptosis of dermal cells	2.37E-04	28
Mammary gland	Rice straw	Positive	development of hematopoietic system	2.46E-04	64
Mammary gland	Rice straw	Positive	development of vasculature	2.47E-04	101
Mammary gland	Rice straw	Positive	morphogenesis of neurites	2.47E-04	101
Mammary gland	Rice straw	Positive	angiogenesis of tumor	2.47E-04	35
Mammary gland	Rice straw	Positive	apoptosis of epithelial cells	2.51E-04	72
Mammary gland	Rice straw	Positive	seizure disorder	2.51E-04	112
Mammary gland	Rice straw	Positive	morphology of connective tissue	2.53E-04	110
Mammary gland	Rice straw	Positive	development of sensory organ	2.56E-04	132
Mammary gland	Rice straw	Positive	abnormal morphology of abdomen	2.56E-04	197
Mammary gland	Rice straw	Positive	cell death of liver cells	2.66E-04	52
Mammary gland	Rice straw	Positive	serous ovarian carcinoma	2.73E-04	53
Mammary	Rice	Positive	autosomal dominant myopathy	2.82E-04	27

gland	straw				
Mammary gland	Rice straw	Positive	migration of fibroblasts	2.93E-04	40
Mammary gland	Rice straw	Positive	morphogenesis of neurons	2.95E-04	102
Mammary gland	Rice straw	Positive	contractility of myocardium	3.01E-04	21
Mammary gland	Rice straw	Positive	function of mononuclear leukocytes	3.02E-04	84
Mammary gland	Rice straw	Positive	apoptosis of blood cells	3.07E-04	117
Mammary gland	Rice straw	Positive	apoptosis of liver cells	3.09E-04	43
Mammary gland	Rice straw	Positive	apoptosis of antigen presenting cells	3.10E-04	39
Mammary gland	Rice straw	Positive	progression of tumor	3.27E-04	44
Mammary gland	Rice straw	Positive	cell death of skin	3.34E-04	31
Mammary gland	Rice straw	Positive	hereditary polyneuropathy	3.40E-04	30
Mammary gland	Rice straw	Positive	female genital tract cancer	3.40E-04	689
Mammary gland	Rice straw	Positive	quantity of macrophages	3.41E-04	53
Mammary gland	Rice straw	Positive	advanced malignant solid tumor	3.64E-04	59
Mammary gland	Rice straw	Positive	quantity of T lymphocytes	3.67E-04	125
Mammary gland	Rice straw	Positive	gliomatosis	3.70E-04	179
Mammary gland	Rice straw	Positive	cell movement of lymphocytes	3.72E-04	95
Mammary gland	Rice straw	Positive	migration of vascular endothelial cells	3.88E-04	45
Mammary gland	Rice straw	Positive	morphology of cardiac muscle	3.88E-04	45
Mammary gland	Rice straw	Positive	survival of organism	3.97E-04	160
Mammary gland	Rice straw	Positive	metastatic malignant neoplasm of digestive system	4.01E-04	60
Mammary gland	Rice straw	Positive	quantity of connective tissue	4.05E-04	139
Mammary	Rice	Positive	abdominal adenocarcinoma	4.10E-04	1164

gland	straw				
Mammary gland	Rice straw	Positive	tumorigenesis of reproductive tract	4.21E-04	692
Mammary gland	Rice straw	Positive	formation of cells	4.22E-04	217
Mammary gland	Rice straw	Positive	quantity of lymphatic system component	4.22E-04	91
Mammary gland	Rice straw	Positive	function of lymphocytes	4.22E-04	83
Mammary gland	Rice straw	Positive	quantity of hematopoietic cells	4.22E-04	105
Mammary gland	Rice straw	Positive	cell movement of muscle cells	4.25E-04	48
Mammary gland	Rice straw	Positive	epileptic seizure	4.34E-04	45
Mammary gland	Rice straw	Positive	cell death of renal glomerulus	4.35E-04	20
Mammary gland	Rice straw	Positive	cell spreading	4.43E-04	69
Mammary gland	Rice straw	Positive	laminopathy	4.50E-04	23
Mammary gland	Rice straw	Positive	Dermatitis	4.52E-04	103
Mammary gland	Rice straw	Positive	accumulation of leukocytes	4.53E-04	67
Mammary gland	Rice straw	Positive	infection of cells	4.61E-04	179
Mammary gland	Rice straw	Positive	hereditary spastic paraplegia	4.64E-04	16
Mammary gland	Rice straw	Positive	abnormal morphology of heart	4.65E-04	92
Mammary gland	Rice straw	Positive	secondary neoplasm of digestive system	4.65E-04	64
Mammary gland	Rice straw	Positive	serous neoplasm	4.72E-04	140
Mammary gland	Rice straw	Positive	cell death of dermal cells	4.82E-04	29
Mammary gland	Rice straw	Positive	formation of focal adhesions	4.82E-04	42
Mammary gland	Rice straw	Positive	apoptosis of fibroblast cell lines	4.86E-04	80
Mammary gland	Rice straw	Positive	autophagy	4.91E-04	90
Mammary	Rice	Positive	neuritogenesis of pheochromocytoma cell lines	4.91E-04	10

gland	straw				
Mammary gland	Rice straw	Positive	cell death of breast cancer cell lines	5.13E-04	81
Mammary gland	Rice straw	Positive	development of lymphatic system	5.18E-04	86
Mammary gland	Rice straw	Positive	mixed papillary and follicular thyroid cancer	5.19E-04	9
Mammary gland	Rice straw	Positive	glioma cancer	5.37E-04	76
Mammary gland	Rice straw	Positive	transactivation	5.41E-04	133
Mammary gland	Rice straw	Positive	peripheral vascular disease	5.51E-04	91
Mammary gland	Rice straw	Positive	apoptosis of fibrosarcoma cell lines	5.51E-04	20
Mammary gland	Rice straw	Positive	quantity of hematopoietic progenitor cells	5.60E-04	104
Mammary gland	Rice straw	Positive	I-kappaB kinase/NF-kappaB cascade	5.60E-04	46
Mammary gland	Rice straw	Positive	breast or colorectal cancer	5.67E-04	985
Mammary gland	Rice straw	Positive	tumorigenesis of genital organ	5.80E-04	785
Mammary gland	Rice straw	Positive	apoptosis of keratinocytes	5.84E-04	25
Mammary gland	Rice straw	Positive	regression of tumor	5.84E-04	25
Mammary gland	Rice straw	Positive	hereditary motor and sensory neuropathy	5.87E-04	27
Mammary gland	Rice straw	Positive	quantity of IL-6 in blood	5.87E-04	26
Mammary gland	Rice straw	Positive	Emery-Dreifuss muscular dystrophy	6.03E-04	21
Mammary gland	Rice straw	Positive	adhesion of immune cells	6.36E-04	84
Mammary gland	Rice straw	Positive	cell movement of antigen presenting cells	6.36E-04	84
Mammary gland	Rice straw	Positive	cell movement of cancer cells	6.37E-04	40
Mammary gland	Rice straw	Positive	cell movement of smooth muscle cells	6.42E-04	43
Mammary gland	Rice straw	Positive	metabolism of nucleic acid component or derivative	6.61E-04	120
Mammary	Rice	Positive	retinal degeneration	6.64E-04	64

gland	straw				
Mammary gland	Rice straw	Positive	formation of filopodia	6.64E-04	44
Mammary gland	Rice straw	Positive	infection by HIV-1	6.76E-04	139
Mammary gland	Rice straw	Positive	cell viability of carcinoma cell lines	6.87E-04	39
Mammary gland	Rice straw	Positive	sensory neuropathy	6.92E-04	28
Mammary gland	Rice straw	Positive	advanced stage carcinoma	7.00E-04	55
Mammary gland	Rice straw	Positive	cell viability of cervical cancer cell lines	7.00E-04	56
Mammary gland	Rice straw	Positive	abnormal morphology of heart ventricle	7.00E-04	46
Mammary gland	Rice straw	Positive	apoptosis of epidermal cells	7.06E-04	26
Mammary gland	Rice straw	Positive	apoptosis of tumor cells	7.08E-04	70
Mammary gland	Rice straw	Positive	degeneration of eye	7.08E-04	65
Mammary gland	Rice straw	Positive	cell movement of tumor cells	7.28E-04	48
Mammary gland	Rice straw	Positive	export of molecule	7.38E-04	72
Mammary gland	Rice straw	Positive	genital tract cancer	7.49E-04	777
Mammary gland	Rice straw	Positive	atherosclerotic lesion	7.49E-04	41
Mammary gland	Rice straw	Positive	contractility of heart	7.86E-04	46
Mammary gland	Rice straw	Positive	pulmonary interstitial fibrosis	7.88E-04	7
Mammary gland	Rice straw	Positive	activation of leukocytes	7.93E-04	157
Mammary gland	Rice straw	Positive	size of lesion	7.98E-04	63
Mammary gland	Rice straw	Positive	formation of thymus gland	8.09E-04	43
Mammary gland	Rice straw	Positive	head and neck cancer	8.09E-04	237
Mammary gland	Rice straw	Positive	cell viability of brain cells	8.13E-04	28
Mammary	Rice	Positive	apoptosis of neuroblastoma cell lines	8.41E-04	34

gland	straw				
Mammary gland	Rice straw	Positive	cell death of keratinocytes	8.44E-04	26
Mammary gland	Rice straw	Positive	morphology of cardiovascular tissue	8.44E-04	26
Mammary gland	Rice straw	Positive	morphology of endothelial tissue	8.46E-04	25
Mammary gland	Rice straw	Positive	polyneuropathy	8.50E-04	31
Mammary gland	Rice straw	Positive	connective or soft tissue tumor	8.70E-04	158
Mammary gland	Rice straw	Positive	oral squamous cell carcinoma	8.70E-04	46
Mammary gland	Rice straw	Positive	formation of thymocytes	8.91E-04	30
Mammary gland	Rice straw	Positive	binding of epithelial cell lines	8.99E-04	13
Mammary gland	Rice straw	Positive	necrosis of renal glomerulus	8.99E-04	18
Mammary gland	Rice straw	Positive	glioblastoma cancer	9.11E-04	73
Mammary gland	Rice straw	Positive	formation of lymphoid organ	9.12E-04	65
Mammary gland	Rice straw	Positive	potentiation of synapse	9.12E-04	40
Mammary gland	Rice straw	Positive	apoptosis of leukocytes	9.58E-04	106
Mammary gland	Rice straw	Positive	cell death of muscle	9.58E-04	82
Mammary gland	Rice straw	Positive	Bleeding	9.79E-04	99
Mammary gland	Rice straw	Positive	cell death of epidermal cells	9.87E-04	27
Mammary gland	Rice straw	Positive	metabolism of nucleotide	9.88E-04	102
Mammary gland	Rice straw	Positive	dephosphorylation of protein	9.98E-04	48
Mammary gland	Rice straw	Positive	Rheumatic Disease	9.98E-04	233
Mammary gland	Rice straw	Positive	concentration of fatty acid	1.02E-03	68
Mammary gland	Rice straw	Positive	morphology of eye	1.02E-03	68
Mammary	Rice	Positive	growth of embryo	1.03E-03	110

gland	straw				
Mammary gland	Rice straw	Positive	gastrointestinal tract cancer	1.05E-03	998
Mammary gland	Rice straw	Positive	abnormal morphology of lymphoid organ	1.08E-03	89
Mammary gland	Rice straw	Positive	cell viability of fibroblast cell lines	1.09E-03	32
Mammary gland	Rice straw	Positive	autosomal dominant Emery-Dreifuss muscular dystrophy	1.10E-03	20
Mammary gland	Rice straw	Positive	abnormal morphology of eye	1.10E-03	64
Mammary gland	Rice straw	Positive	morphology of lymphatic system component	1.12E-03	97
Mammary gland	Rice straw	Positive	differentiation of tumor cell lines	1.16E-03	89
Mammary gland	Rice straw	Positive	apoptosis of hepatoma cell lines	1.18E-03	40
Mammary gland	Rice straw	Positive	progressive motor neuropathy	1.21E-03	119
Mammary gland	Rice straw	Positive	abnormal morphology of vascular endothelial cells	1.22E-03	15
Mammary gland	Rice straw	Positive	transformation of fibroblast cell lines	1.23E-03	63
Mammary gland	Rice straw	Positive	female genital tract adenocarcinoma	1.24E-03	583
Mammary gland	Rice straw	Positive	amyotrophic lateral sclerosis	1.24E-03	55
Mammary gland	Rice straw	Positive	migration of mononuclear leukocytes	1.24E-03	87
Mammary gland	Rice straw	Positive	G2/M phase of fibroblasts	1.24E-03	6
Mammary gland	Rice straw	Positive	quantity of metal	1.25E-03	114
Mammary gland	Rice straw	Positive	formation of T lymphocytes	1.25E-03	32
Mammary gland	Rice straw	Positive	gastrointestinal carcinoma	1.28E-03	897
Mammary gland	Rice straw	Positive	necrosis of muscle	1.32E-03	81
Mammary gland	Rice straw	Positive	uterine tumor	1.34E-03	642
Mammary gland	Rice straw	Positive	Ras protein signal transduction	1.38E-03	20
Mammary	Rice	Positive	migration of cancer cells	1.39E-03	49

gland	straw				
Mammary gland	Rice straw	Positive	generation of leukocytes	1.41E-03	42
Mammary gland	Rice straw	Positive	gait disturbance	1.44E-03	36
Mammary gland	Rice straw	Positive	long-term potentiation of synapse	1.45E-03	39
Mammary gland	Rice straw	Positive	vascularization	1.45E-03	58
Mammary gland	Rice straw	Positive	head and neck carcinoma	1.45E-03	109
Mammary gland	Rice straw	Positive	cell death of blood cells	1.45E-03	154
Mammary gland	Rice straw	Positive	formation of lymphatic system component	1.46E-03	70
Mammary gland	Rice straw	Positive	activation of blood cells	1.46E-03	165
Mammary gland	Rice straw	Positive	proliferation of hematopoietic cells	1.48E-03	62
Mammary gland	Rice straw	Positive	oral tumor	1.49E-03	72
Mammary gland	Rice straw	Positive	apoptosis of breast cancer cell lines	1.49E-03	69
Mammary gland	Rice straw	Positive	quantity of lesion	1.49E-03	46
Mammary gland	Rice straw	Positive	muscular hypertrophy	1.52E-03	51
Mammary gland	Rice straw	Positive	cell cycle progression	1.54E-03	225
Mammary gland	Rice straw	Positive	liver tumor	1.55E-03	862
Mammary gland	Rice straw	Positive	morphology of lymphoid organ	1.60E-03	91
Mammary gland	Rice straw	Positive	juvenile dermatomyositis	1.61E-03	19
Mammary gland	Rice straw	Positive	cell death of muscle cells	1.61E-03	79
Mammary gland	Rice straw	Positive	morphology of muscle	1.61E-03	79
Mammary gland	Rice straw	Positive	attachment of cells	1.61E-03	36
Mammary gland	Rice straw	Positive	Shock Response	1.61E-03	44
Mammary	Rice	Positive	abdominal carcinoma	1.62E-03	1171

gland	straw				
Mammary gland	Rice straw	Positive	interphase	1.62E-03	146
Mammary gland	Rice straw	Positive	cell death of macrophages	1.65E-03	34
Mammary gland	Rice straw	Positive	apoptosis of mesangial cells	1.67E-03	11
Mammary gland	Rice straw	Positive	production of lactic acid	1.67E-03	17
Mammary gland	Rice straw	Positive	abnormal morphology of endothelial tissue	1.67E-03	20
Mammary gland	Rice straw	Positive	function of respiratory system	1.67E-03	20
Mammary gland	Rice straw	Positive	granulation tissue	1.72E-03	14
Mammary gland	Rice straw	Positive	trafficking of mononuclear leukocytes	1.72E-03	14
Mammary gland	Rice straw	Positive	contractility of cardiac muscle	1.74E-03	38
Mammary gland	Rice straw	Positive	binding of embryonic cell lines	1.79E-03	12
Mammary gland	Rice straw	Positive	cell viability of sarcoma cell lines	1.80E-03	13
Mammary gland	Rice straw	Positive	vaso-occlusion	1.80E-03	109
Mammary gland	Rice straw	Positive	proliferation of tumor cells	1.85E-03	107
Mammary gland	Rice straw	Positive	contractility of ventricular myocardium	1.85E-03	18
Mammary gland	Rice straw	Positive	hepatobiliary system cancer	1.85E-03	859
Mammary gland	Rice straw	Positive	migration of prostate cancer cells	1.86E-03	7
Mammary gland	Rice straw	Positive	activation of lymphocytes	1.89E-03	105
Mammary gland	Rice straw	Positive	generation of lymphocytes	1.89E-03	37
Mammary gland	Rice straw	Positive	proliferation of neuronal cells	1.90E-03	129
Mammary gland	Rice straw	Positive	development of lymphatic system component	1.93E-03	73
Mammary gland	Rice straw	Positive	damage of nervous system	1.93E-03	55
Mammary	Rice	Positive	recruitment of cells	1.98E-03	83

gland	straw				
Mammary gland	Rice straw	Positive	urogenital cancer	1.98E-03	880
Mammary gland	Rice straw	Positive	synthesis of purine nucleotide	1.98E-03	44
Mammary gland	Rice straw	Positive	generation of cells	1.99E-03	63
Mammary gland	Rice straw	Positive	proliferation of cervical cancer cell lines	2.01E-03	50
Mammary gland	Rice straw	Positive	development of hematopoietic progenitor cells	2.02E-03	48
Mammary gland	Rice straw	Positive	Gastrointestinal Tract Cancer and Tumors	2.02E-03	1009
Mammary gland	Rice straw	Positive	interphase of cervical cancer cell lines	2.07E-03	24
Mammary gland	Rice straw	Positive	transactivation of RNA	2.09E-03	122
Mammary gland	Rice straw	Positive	quantity of metal ion	2.09E-03	101
Mammary gland	Rice straw	Positive	transmigration of blood cells	2.12E-03	34
Mammary gland	Rice straw	Positive	occlusion of blood vessel	2.12E-03	109
Mammary gland	Rice straw	Positive	migration of muscle cells	2.14E-03	42
Mammary gland	Rice straw	Positive	cell death of hepatoma cell lines	2.17E-03	43
Mammary gland	Rice straw	Positive	epithelial-mesenchymal transition	2.20E-03	51
Mammary gland	Rice straw	Positive	adhesion of blood cells	2.21E-03	87
Mammary gland	Rice straw	Positive	apoptosis of myeloid cells	2.22E-03	47
Mammary gland	Rice straw	Positive	apoptosis of phagocytes	2.22E-03	47
Mammary gland	Rice straw	Positive	liver cancer	2.24E-03	854
Mammary gland	Rice straw	Positive	metastatic solid tumor	2.26E-03	99
Mammary gland	Rice straw	Positive	proliferation of breast cancer cell lines	2.26E-03	99
Mammary gland	Rice straw	Positive	mammary tumor	2.26E-03	340
Mammary	Rice	Positive	synthesis of ATP	2.27E-03	26

gland	straw				
Mammary gland	Rice straw	Positive	trafficking of blood cells	2.29E-03	18
Mammary gland	Rice straw	Positive	synthesis of nucleotide	2.29E-03	84
Mammary gland	Rice straw	Positive	metabolism of sphingolipid	2.29E-03	36
Mammary gland	Rice straw	Positive	inflammation of organ	2.31E-03	249
Mammary gland	Rice straw	Positive	abnormal posture	2.36E-03	25
Mammary gland	Rice straw	Positive	apoptosis of melanoma cell lines	2.39E-03	34
Mammary gland	Rice straw	Positive	biosynthesis of purine ribonucleotide	2.40E-03	28
Mammary gland	Rice straw	Positive	morphology of endothelial cells	2.40E-03	19
Mammary gland	Rice straw	Positive	cancer of cells	2.40E-03	61
Mammary gland	Rice straw	Positive	antiviral response of cells	2.40E-03	16
Mammary gland	Rice straw	Positive	apoptosis of retinal cells	2.40E-03	16
Mammary gland	Rice straw	Positive	quantity of smooth muscle cells	2.40E-03	16
Mammary gland	Rice straw	Positive	transport of carbohydrate	2.41E-03	48
Mammary gland	Rice straw	Positive	G2 phase of fibroblasts	2.41E-03	11
Mammary gland	Rice straw	Positive	atrophy of muscle	2.41E-03	38
Mammary gland	Rice straw	Positive	perinatal death	2.41E-03	132
Mammary gland	Rice straw	Positive	cell death of melanoma cell lines	2.43E-03	60
Mammary gland	Rice straw	Positive	Lymphocyte migration	2.43E-03	80
Mammary gland	Rice straw	Positive	Wound	2.44E-03	55
Mammary gland	Rice straw	Positive	cell death of sarcoma cell lines	2.44E-03	55
Mammary gland	Rice straw	Positive	vascularization of tumor	2.44E-03	20
Mammary	Rice	Positive	peripheral neuropathy	2.45E-03	39

gland	straw				
Mammary gland	Rice straw	Positive	septic shock	2.45E-03	39
Mammary gland	Rice straw	Positive	synthesis of fatty acid	2.49E-03	73
Mammary gland	Rice straw	Positive	secretion of molecule	2.50E-03	123
Mammary gland	Rice straw	Positive	hepatocellular carcinoma	2.57E-03	827
Mammary gland	Rice straw	Positive	quantity of lymphoid organ	2.57E-03	72
Mammary gland	Rice straw	Positive	development of neurons	2.65E-03	170
Mammary gland	Rice straw	Positive	occlusion of artery	2.65E-03	107
Mammary gland	Rice straw	Positive	demyelination of central nervous system	2.69E-03	15
Mammary gland	Rice straw	Positive	migration of smooth muscle cells	2.69E-03	38
Mammary gland	Rice straw	Positive	polyarticular juvenile rheumatoid arthritis	2.73E-03	25
Mammary gland	Rice straw	Positive	cell death of immune cells	2.73E-03	145
Mammary gland	Rice straw	Positive	accumulation of lipid	2.73E-03	70
Mammary gland	Rice straw	Positive	dysmyelination	2.76E-03	35
Mammary gland	Rice straw	Positive	endometrium tumor	2.83E-03	579
Mammary gland	Rice straw	Positive	metabolism of nucleoside triphosphate	2.87E-03	36
Mammary gland	Rice straw	Positive	formation of lymphocytes	2.88E-03	33
Mammary gland	Rice straw	Positive	growth of plasma membrane projections	2.89E-03	105
Mammary gland	Rice straw	Positive	growth of yeast	2.89E-03	27
Mammary gland	Rice straw	Positive	apoptosis of muscle	2.95E-03	62
Mammary gland	Rice straw	Positive	morphology of myocardium	2.95E-03	31
Mammary gland	Rice straw	Positive	proliferation of hematopoietic progenitor cells	2.98E-03	58
Mammary	Rice	Positive	abnormal morphology of digestive system	3.05E-03	121

gland	straw				
Mammary gland	Rice straw	Positive	formation of skin lesion	3.05E-03	26
Mammary gland	Rice straw	Positive	autosomal dominant disease	3.13E-03	140
Mammary gland	Rice straw	Positive	cell viability of central nervous system cells	3.19E-03	30
Mammary gland	Rice straw	Positive	glycolysis of cells	3.19E-03	30
Mammary gland	Rice straw	Positive	neoplasia of cells	3.19E-03	100
Mammary gland	Rice straw	Positive	migration of tumor cells	3.24E-03	54
Mammary gland	Rice straw	Negative	cell death	1.06E-37	1347
Mammary gland	Rice straw	Negative	proliferation of cells	7.76E-34	1449
Mammary gland	Rice straw	Negative	cancer	9.73E-33	3722
Mammary gland	Rice straw	Negative	malignant solid tumor	3.27E-31	3675
Mammary gland	Rice straw	Negative	apoptosis	5.17E-29	1066
Mammary gland	Rice straw	Negative	necrosis	8.84E-29	1040
Mammary gland	Rice straw	Negative	organismal death	8.41E-27	969
Mammary gland	Rice straw	Negative	morbidity or mortality	2.40E-26	978
Mammary gland	Rice straw	Negative	organization of cytoplasm	3.02E-26	660
Mammary gland	Rice straw	Negative	transport of molecule	2.30E-23	677
Mammary gland	Rice straw	Negative	organization of cytoskeleton	2.81E-23	598
Mammary gland	Rice straw	Negative	morphology of cells	1.83E-22	791
Mammary gland	Rice straw	Negative	tumorigenesis of tissue	1.77E-18	3080
Mammary gland	Rice straw	Negative	microtubule dynamics	2.19E-18	501
Mammary gland	Rice straw	Negative	cell movement	2.32E-18	834
Mammary	Rice	Negative	neoplasia of epithelial tissue	2.41E-18	3032

gland	straw				
Mammary gland	Rice straw	Negative	epithelial cancer	5.34E-18	3003
Mammary gland	Rice straw	Negative	cell death of tumor cell lines	3.17E-17	618
Mammary gland	Rice straw	Negative	abdominal neoplasm	1.52E-16	2998
Mammary gland	Rice straw	Negative	cell survival	1.91E-16	567
Mammary gland	Rice straw	Negative	abdominal cancer	4.44E-16	2962
Mammary gland	Rice straw	Negative	differentiation of cells	7.12E-16	862
Mammary gland	Rice straw	Negative	cellular homeostasis	2.33E-15	607
Mammary gland	Rice straw	Negative	expression of RNA	2.55E-15	823
Mammary gland	Rice straw	Negative	migration of cells	6.51E-15	737
Mammary gland	Rice straw	Negative	cell viability	8.19E-15	526
Mammary gland	Rice straw	Negative	angiogenesis	1.52E-14	383
Mammary gland	Rice straw	Negative	Movement Disorders	5.57E-14	436
Mammary gland	Rice straw	Negative	cell movement of tumor cell lines	6.11E-14	360
Mammary gland	Rice straw	Negative	vasculogenesis	1.19E-13	316
Mammary gland	Rice straw	Negative	concentration of lipid	1.76E-13	343
Mammary gland	Rice straw	Negative	cell death of connective tissue cells	2.34E-13	264
Mammary gland	Rice straw	Negative	apoptosis of tumor cell lines	2.34E-13	487
Mammary gland	Rice straw	Negative	transcription	4.91E-13	757
Mammary gland	Rice straw	Negative	abnormal morphology of cells	9.45E-13	503
Mammary gland	Rice straw	Negative	transcription of RNA	1.56E-12	707
Mammary gland	Rice straw	Negative	morphology of cardiovascular system	1.89E-12	267
Mammary	Rice	Negative	formation of cellular protrusions	1.96E-12	372

gland	straw				
Mammary gland	Rice straw	Negative	size of body	1.98E-12	353
Mammary gland	Rice straw	Negative	proliferation of tumor cell lines	4.12E-12	598
Mammary gland	Rice straw	Negative	development of vasculature	5.26E-12	188
Mammary gland	Rice straw	Negative	digestive system cancer	9.85E-12	2550
Mammary gland	Rice straw	Negative	invasion of cells	1.45E-11	356
Mammary gland	Rice straw	Negative	digestive organ tumor	1.45E-11	2571
Mammary gland	Rice straw	Negative	development of body trunk	1.69E-11	444
Mammary gland	Rice straw	Negative	necrosis of epithelial tissue	7.80E-11	249
Mammary gland	Rice straw	Negative	cell death of epithelial cells	9.04E-11	214
Mammary gland	Rice straw	Negative	Viral Infection	1.31E-10	611
Mammary gland	Rice straw	Negative	cell death of cervical cancer cell lines	1.45E-10	157
Mammary gland	Rice straw	Negative	proliferation of connective tissue cells	1.87E-10	249
Mammary gland	Rice straw	Negative	development of cytoplasm	2.64E-10	199
Mammary gland	Rice straw	Negative	quantity of cells	2.64E-10	662
Mammary gland	Rice straw	Negative	degeneration of nervous system	3.17E-10	131
Mammary gland	Rice straw	Negative	Organ Degeneration	4.46E-10	210
Mammary gland	Rice straw	Negative	protein kinase cascade	6.70E-10	182
Mammary gland	Rice straw	Negative	cell viability of tumor cell lines	6.75E-10	315
Mammary gland	Rice straw	Negative	perinatal death	7.32E-10	247
Mammary gland	Rice straw	Negative	dyskinesia	8.85E-10	255
Mammary gland	Rice straw	Negative	disorder of basal ganglia	9.06E-10	310
Mammary	Rice	Negative	formation of cytoskeleton	9.73E-10	164

gland	straw				
Mammary gland	Rice straw	Negative	autosomal recessive disease	1.22E-09	365
Mammary gland	Rice straw	Negative	abnormal morphology of cardiovascular system	1.23E-09	236
Mammary gland	Rice straw	Negative	migration of tumor cell lines	1.46E-09	287
Mammary gland	Rice straw	Negative	growth of connective tissue	1.51E-09	264
Mammary gland	Rice straw	Negative	phosphorylation of protein	1.82E-09	297
Mammary gland	Rice straw	Negative	metabolism of carbohydrate	1.88E-09	255
Mammary gland	Rice straw	Negative	neurological signs	1.99E-09	266
Mammary gland	Rice straw	Negative	synthesis of lipid	2.08E-09	289
Mammary gland	Rice straw	Negative	cell death of fibroblast cell lines	2.35E-09	182
Mammary gland	Rice straw	Negative	neuromuscular disease	3.09E-09	349
Mammary gland	Rice straw	Negative	concentration of fatty acid	3.18E-09	125
Mammary gland	Rice straw	Negative	transcription of DNA	3.36E-09	575
Mammary gland	Rice straw	Negative	Neurodegeneration	4.30E-09	136
Mammary gland	Rice straw	Negative	Huntington's Disease	4.71E-09	236
Mammary gland	Rice straw	Negative	transport of carbohydrate	5.08E-09	91
Mammary gland	Rice straw	Negative	degeneration of cells	5.83E-09	149
Mammary gland	Rice straw	Negative	quantity of leukocytes	6.18E-09	359
Mammary gland	Rice straw	Negative	Growth Failure	7.16E-09	244
Mammary gland	Rice straw	Negative	invasion of tumor cell lines	9.05E-09	268
Mammary gland	Rice straw	Negative	morphology of body cavity	1.05E-08	455
Mammary gland	Rice straw	Negative	fibrogenesis	1.50E-08	173
Mammary	Rice	Negative	cell death of epithelial cell lines	1.55E-08	124

gland	straw				
Mammary gland	Rice straw	Negative	abnormal morphology of thoracic cavity	1.62E-08	238
Mammary gland	Rice straw	Negative	apoptosis of fibroblast cell lines	2.09E-08	141
Mammary gland	Rice straw	Negative	development of leukocytes	2.42E-08	238
Mammary gland	Rice straw	Negative	development of lymphocytes	2.46E-08	225
Mammary gland	Rice straw	Negative	apoptosis of cervical cancer cell lines	2.57E-08	123
Mammary gland	Rice straw	Negative	development of blood cells	2.82E-08	261
Mammary gland	Rice straw	Negative	growth of lesion	3.19E-08	313
Mammary gland	Rice straw	Negative	development of mononuclear leukocytes	3.20E-08	226
Mammary gland	Rice straw	Negative	quantity of blood cells	3.21E-08	394
Mammary gland	Rice straw	Negative	growth of tumor	3.90E-08	312
Mammary gland	Rice straw	Negative	cell viability of cervical cancer cell lines	4.07E-08	99
Mammary gland	Rice straw	Negative	growth of organism	4.20E-08	326
Mammary gland	Rice straw	Negative	abnormal morphology of body cavity	5.94E-08	432
Mammary gland	Rice straw	Negative	organization of organelle	6.02E-08	218
Mammary gland	Rice straw	Negative	secretory pathway	8.18E-08	89
Mammary gland	Rice straw	Negative	neuronal cell death	8.54E-08	269
Mammary gland	Rice straw	Negative	cell transformation	1.02E-07	188
Mammary gland	Rice straw	Negative	growth of epithelial tissue	1.07E-07	268
Mammary gland	Rice straw	Negative	T cell development	1.31E-07	203
Mammary gland	Rice straw	Negative	growth of muscle tissue	1.33E-07	155
Mammary gland	Rice straw	Negative	proliferation of muscle cells	1.50E-07	154
Mammary	Rice	Negative	cellular degradation	1.52E-07	110

gland	straw				
Mammary gland	Rice straw	Negative	small GTPase mediated signal transduction	1.75E-07	83
Mammary gland	Rice straw	Negative	growth of embryo	1.75E-07	193
Mammary gland	Rice straw	Negative	degeneration of neurons	1.85E-07	104
Mammary gland	Rice straw	Negative	cell spreading	2.10E-07	118
Mammary gland	Rice straw	Negative	apoptosis of epithelial cell lines	2.21E-07	98
Mammary gland	Rice straw	Negative	transport of monosaccharide	2.24E-07	73
Mammary gland	Rice straw	Negative	formation of filaments	2.51E-07	163
Mammary gland	Rice straw	Negative	proliferation of smooth muscle cells	2.63E-07	121
Mammary gland	Rice straw	Negative	metabolism of protein	3.25E-07	369
Mammary gland	Rice straw	Negative	ubiquitination of protein	3.32E-07	138
Mammary gland	Rice straw	Negative	homeostasis of leukocytes	3.63E-07	220
Mammary gland	Rice straw	Negative	Bleeding	3.95E-07	172
Mammary gland	Rice straw	Negative	behavior	3.98E-07	374
Mammary gland	Rice straw	Negative	Lymphocyte homeostasis	4.38E-07	216
Mammary gland	Rice straw	Negative	morphology of vessel	5.14E-07	128
Mammary gland	Rice straw	Negative	transport of protein	5.37E-07	126
Mammary gland	Rice straw	Negative	cell cycle progression	5.38E-07	393
Mammary gland	Rice straw	Negative	T cell homeostasis	5.38E-07	205
Mammary gland	Rice straw	Negative	autophagy	5.58E-07	153
Mammary gland	Rice straw	Negative	ubiquitination	5.58E-07	139
Mammary gland	Rice straw	Negative	colony formation	6.69E-07	212
Mammary	Rice	Negative	morphology of heart	7.14E-07	164

gland	straw				
Mammary gland	Rice straw	Negative	quantity of carbohydrate	7.80E-07	205
Mammary gland	Rice straw	Negative	cell death of immune cells	7.91E-07	256
Mammary gland	Rice straw	Negative	size of animal	8.21E-07	79
Mammary gland	Rice straw	Negative	organization of actin cytoskeleton	8.21E-07	125
Mammary gland	Rice straw	Negative	Edema	8.36E-07	141
Mammary gland	Rice straw	Negative	congenital anomaly of musculoskeletal system	8.48E-07	283
Mammary gland	Rice straw	Negative	female genital tract serous cancer	9.59E-07	156
Mammary gland	Rice straw	Negative	formation of filopodia	9.78E-07	74
Mammary gland	Rice straw	Negative	function of cardiovascular system	1.03E-06	150
Mammary gland	Rice straw	Negative	transport of D-hexose	1.07E-06	66
Mammary gland	Rice straw	Negative	cognition	1.07E-06	181
Mammary gland	Rice straw	Negative	fatty acid metabolism	1.09E-06	234
Mammary gland	Rice straw	Negative	exocytosis	1.12E-06	83
Mammary gland	Rice straw	Negative	cell death of blood cells	1.15E-06	267
Mammary gland	Rice straw	Negative	metabolism of nucleotide	1.20E-06	175
Mammary gland	Rice straw	Negative	serous neoplasm	1.22E-06	237
Mammary gland	Rice straw	Negative	development of lymphatic system component	1.48E-06	127
Mammary gland	Rice straw	Negative	transport of D-glucose	1.48E-06	65
Mammary gland	Rice straw	Negative	activation of cells	1.59E-06	366
Mammary gland	Rice straw	Negative	proliferation of fibroblasts	1.63E-06	143
Mammary gland	Rice straw	Negative	activation of enzyme	1.64E-06	156
Mammary	Rice	Negative	abdominal adenocarcinoma	1.82E-06	2000

gland	straw				
Mammary gland	Rice straw	Negative	development of lymphatic system	1.97E-06	144
Mammary gland	Rice straw	Negative	differentiation of leukocytes	2.14E-06	258
Mammary gland	Rice straw	Negative	intermediate disease stage peripheral arterial disease	2.20E-06	70
Mammary gland	Rice straw	Negative	neonatal death	2.26E-06	174
Mammary gland	Rice straw	Negative	cell death of kidney cells	2.35E-06	141
Mammary gland	Rice straw	Negative	colony formation of cells	2.38E-06	193
Mammary gland	Rice straw	Negative	quantity of mononuclear leukocytes	2.41E-06	280
Mammary gland	Rice straw	Negative	arthropathy	2.41E-06	356
Mammary gland	Rice straw	Negative	learning	2.41E-06	166
Mammary gland	Rice straw	Negative	transactivation	2.41E-06	224
Mammary gland	Rice straw	Negative	engulfment of cells	2.49E-06	162
Mammary gland	Rice straw	Negative	seizures	2.64E-06	159
Mammary gland	Rice straw	Negative	proliferation of neuronal cells	2.66E-06	223
Mammary gland	Rice straw	Negative	phosphorylation of L-amino acid	2.66E-06	93
Mammary gland	Rice straw	Negative	arthritis	2.73E-06	350
Mammary gland	Rice straw	Negative	uptake of carbohydrate	2.88E-06	119
Mammary gland	Rice straw	Negative	uptake of monosaccharide	2.96E-06	111
Mammary gland	Rice straw	Negative	concentration of Ca2+	2.96E-06	58
Mammary gland	Rice straw	Negative	morphology of skin	3.14E-06	112
Mammary gland	Rice straw	Negative	morphology of nervous system	3.38E-06	296
Mammary gland	Rice straw	Negative	differentiation of lymphocytes	3.74E-06	194
Mammary	Rice	Negative	survival of organism	4.41E-06	267

gland	straw				
Mammary gland	Rice straw	Negative	growth of lymphatic system component	4.42E-06	82
Mammary gland	Rice straw	Negative	peripheral arterial disease	4.95E-06	89
Mammary gland	Rice straw	Negative	degeneration of central nervous system	4.95E-06	59
Mammary gland	Rice straw	Negative	metabolism of nucleic acid component or derivative	5.05E-06	201
Mammary gland	Rice straw	Negative	morphology of blood vessel	5.27E-06	115
Mammary gland	Rice straw	Negative	synthesis of carbohydrate	5.27E-06	167
Mammary gland	Rice straw	Negative	Thrombosis	5.37E-06	73
Mammary gland	Rice straw	Negative	adenocarcinoma	5.56E-06	2186
Mammary gland	Rice straw	Negative	function of blood cells	5.56E-06	225
Mammary gland	Rice straw	Negative	autosomal dominant disease	5.56E-06	243
Mammary gland	Rice straw	Negative	seizure disorder	5.88E-06	183
Mammary gland	Rice straw	Negative	dysmyelination	5.89E-06	60
Mammary gland	Rice straw	Negative	uterine serous papillary cancer	6.00E-06	96
Mammary gland	Rice straw	Negative	proliferation of epithelial cells	6.36E-06	191
Mammary gland	Rice straw	Negative	cell movement of fibroblasts	6.36E-06	78
Mammary gland	Rice straw	Negative	differentiation of muscle cell lines	6.95E-06	70
Mammary gland	Rice straw	Negative	formation of lymphatic system component	7.05E-06	118
Mammary gland	Rice straw	Negative	metabolism of sphingolipid	7.05E-06	61
Mammary gland	Rice straw	Negative	morphology of head	7.17E-06	326
Mammary gland	Rice straw	Negative	quantity of lymphocytes	7.43E-06	267
Mammary gland	Rice straw	Negative	benign neoplasia	7.57E-06	343
Mammary	Rice	Negative	peripheral vascular disease	7.71E-06	150

gland	straw				
Mammary gland	Rice straw	Negative	formation of skin	7.72E-06	145
Mammary gland	Rice straw	Negative	proliferation of blood cells	7.98E-06	314
Mammary gland	Rice straw	Negative	differentiation of connective tissue cells	7.98E-06	244
Mammary gland	Rice straw	Negative	morphology of bone	8.28E-06	175
Mammary gland	Rice straw	Negative	cell death of T lymphocytes	8.36E-06	126
Mammary gland	Rice straw	Negative	apoptosis of connective tissue cells	8.38E-06	119
Mammary gland	Rice straw	Negative	gastrointestinal carcinoma	8.50E-06	1542
Mammary gland	Rice straw	Negative	demyelination	8.50E-06	54
Mammary gland	Rice straw	Negative	replication of RNA virus	8.86E-06	195
Mammary gland	Rice straw	Negative	apoptosis of epithelial cells	9.46E-06	115
Mammary gland	Rice straw	Negative	formation of actin filaments	9.48E-06	122
Mammary gland	Rice straw	Negative	metabolism of glycosphingolipid	9.82E-06	56
Mammary gland	Rice straw	Negative	morphology of reproductive system	1.04E-05	205
Mammary gland	Rice straw	Negative	cell death of stem cells	1.04E-05	40
Mammary gland	Rice straw	Negative	differentiation of mononuclear leukocytes	1.04E-05	207
Mammary gland	Rice straw	Negative	abnormal morphology of abdomen	1.04E-05	325
Mammary gland	Rice straw	Negative	mitosis	1.05E-05	191
Mammary gland	Rice straw	Negative	synthesis of nucleotide	1.05E-05	143
Mammary gland	Rice straw	Negative	quantity of connective tissue	1.05E-05	229
Mammary gland	Rice straw	Negative	cell death of kidney cell lines	1.22E-05	121
Mammary gland	Rice straw	Negative	abnormal morphology of heart	1.26E-05	150
Mammary	Rice	Negative	concentration of phospholipid	1.26E-05	77

gland	straw				
Mammary gland	Rice straw	Negative	differentiation of blood cells	1.27E-05	313
Mammary gland	Rice straw	Negative	proliferation of immune cells	1.28E-05	295
Mammary gland	Rice straw	Negative	breast or colorectal cancer	1.37E-05	1682
Mammary gland	Rice straw	Negative	necrosis of kidney	1.38E-05	143
Mammary gland	Rice straw	Negative	function of leukocytes	1.40E-05	205
Mammary gland	Rice straw	Negative	migration of smooth muscle cells	1.40E-05	64
Mammary gland	Rice straw	Negative	activation of Protein kinase	1.43E-05	102
Mammary gland	Rice straw	Negative	concentration of acylglycerol	1.53E-05	132
Mammary gland	Rice straw	Negative	migration of muscle cells	1.61E-05	70
Mammary gland	Rice straw	Negative	metabolism of membrane lipid derivative	1.63E-05	151
Mammary gland	Rice straw	Negative	cell movement of connective tissue cells	1.65E-05	91
Mammary gland	Rice straw	Negative	movement of vascular endothelial cells	1.68E-05	76
Mammary gland	Rice straw	Negative	formation of cells	1.69E-05	360
Mammary gland	Rice straw	Negative	cell death of muscle cells	1.74E-05	132
Mammary gland	Rice straw	Negative	development of endothelial tissue	1.77E-05	143
Mammary gland	Rice straw	Negative	growth of embryonic tissue	1.84E-05	110
Mammary gland	Rice straw	Negative	cell death of muscle	1.89E-05	135
Mammary gland	Rice straw	Negative	abnormal morphology of embryonic tissue	1.89E-05	208
Mammary gland	Rice straw	Negative	abnormal morphology of reproductive system	2.03E-05	194
Mammary gland	Rice straw	Negative	development of cardiovascular tissue	2.05E-05	144
Mammary gland	Rice straw	Negative	cell death of fibroblasts	2.05E-05	106
Mammary	Rice	Negative	formation of lymphoid organ	2.05E-05	106

gland	straw				
Mammary gland	Rice straw	Negative	biosynthesis of nucleoside triphosphate	2.06E-05	48
Mammary gland	Rice straw	Negative	binding of DNA	2.27E-05	198
Mammary gland	Rice straw	Negative	cell movement of smooth muscle cells	2.39E-05	68
Mammary gland	Rice straw	Negative	necrosis of muscle	2.39E-05	134
Mammary gland	Rice straw	Negative	abdominal carcinoma	2.42E-05	2012
Mammary gland	Rice straw	Negative	synthesis of sphingolipid	2.43E-05	50
Mammary gland	Rice straw	Negative	quantity of B lymphocytes	2.43E-05	135
Mammary gland	Rice straw	Negative	proliferation of fibroblast cell lines	2.44E-05	163
Mammary gland	Rice straw	Negative	metabolism of nucleoside triphosphate	2.44E-05	60
Mammary gland	Rice straw	Negative	abnormal morphology of head	2.48E-05	307
Mammary gland	Rice straw	Negative	replication of virus	2.50E-05	212
Mammary gland	Rice straw	Negative	apoptosis of kidney cell lines	2.65E-05	96
Mammary gland	Rice straw	Negative	apoptosis of pheochromocytoma cell lines	2.66E-05	42
Mammary gland	Rice straw	Negative	cell movement of muscle cells	2.70E-05	75
Mammary gland	Rice straw	Negative	development of epithelial tissue	2.71E-05	196
Mammary gland	Rice straw	Negative	activation of blood cells	2.81E-05	277
Mammary gland	Rice straw	Negative	cell death of lymphocytes	2.82E-05	149
Mammary gland	Rice straw	Negative	formation of plasma membrane projections	2.97E-05	224
Mammary gland	Rice straw	Negative	abnormal morphology of nervous system	2.99E-05	269
Mammary gland	Rice straw	Negative	cell death of mononuclear leukocytes	2.99E-05	153
Mammary gland	Rice straw	Negative	gastrointestinal tract cancer	3.17E-05	1706
Mammary	Rice	Negative	hepatic steatosis	3.20E-05	106

gland	straw				
Mammary gland	Rice straw	Negative	paraproteinemia	3.20E-05	131
Mammary gland	Rice straw	Negative	morphology of respiratory system	3.25E-05	122
Mammary gland	Rice straw	Negative	neuritogenesis	3.27E-05	219
Mammary gland	Rice straw	Negative	stress response of cells	3.29E-05	59
Mammary gland	Rice straw	Negative	homing of cells	3.29E-05	210
Mammary gland	Rice straw	Negative	congenital malformation of skeleton	3.31E-05	162
Mammary gland	Rice straw	Negative	endocytosis	3.40E-05	134
Mammary gland	Rice straw	Negative	function of muscle	3.48E-05	135
Mammary gland	Rice straw	Negative	endothelial cell development	3.63E-05	137
Mammary gland	Rice straw	Negative	growth of skin	3.63E-05	88
Mammary gland	Rice straw	Negative	synthesis of fatty acid	3.68E-05	122
Mammary gland	Rice straw	Negative	differentiation of epithelial tissue	3.76E-05	139
Mammary gland	Rice straw	Negative	proliferation of hematopoietic progenitor cells	3.76E-05	97
Mammary gland	Rice straw	Negative	differentiation of T lymphocytes	3.76E-05	146
Mammary gland	Rice straw	Negative	synthesis of reactive oxygen species	3.80E-05	182
Mammary gland	Rice straw	Negative	degeneration of brain	3.83E-05	54
Mammary gland	Rice straw	Negative	abnormal morphology of respiratory system	3.94E-05	120
Mammary gland	Rice straw	Negative	synthesis of ceramide	4.03E-05	34
Mammary gland	Rice straw	Negative	Gastrointestinal Tract Cancer and Tumors	4.03E-05	1731
Mammary gland	Rice straw	Negative	interphase	4.10E-05	244
Mammary gland	Rice straw	Negative	synthesis of glycosphingolipid	4.10E-05	45
Mammary	Rice	Negative	proliferation of embryonic cells	4.10E-05	99

gland	straw				
Mammary gland	Rice straw	Negative	infection by RNA virus	4.21E-05	317
Mammary gland	Rice straw	Negative	MAPKKK cascade	4.22E-05	83
Mammary gland	Rice straw	Negative	proliferation of epidermal cells	4.22E-05	70
Mammary gland	Rice straw	Negative	cell death of pheochromocytoma cell lines	4.41E-05	52
Mammary gland	Rice straw	Negative	plasma cell dyscrasia	4.44E-05	130
Mammary gland	Rice straw	Negative	accumulation of cells	4.44E-05	136
Mammary gland	Rice straw	Negative	multiple congenital anomalies	4.45E-05	161
Mammary gland	Rice straw	Negative	proliferation of hematopoietic cells	4.45E-05	101
Mammary gland	Rice straw	Negative	Rheumatic Disease	4.45E-05	389
Mammary gland	Rice straw	Negative	activation of DNA endogenous promoter	4.58E-05	433
Mammary gland	Rice straw	Negative	abnormal morphology of bone	4.62E-05	166
Mammary gland	Rice straw	Negative	abnormal quantity of lipid	4.77E-05	62
Mammary gland	Rice straw	Negative	abnormal morphology of internal genitalia	4.89E-05	135
Mammary gland	Rice straw	Negative	cell death of skin	5.05E-05	46
Mammary gland	Rice straw	Negative	quantity of IL-6 in blood	5.25E-05	39
Mammary gland	Rice straw	Negative	biosynthesis of purine ribonucleotide	5.37E-05	45
Mammary gland	Rice straw	Negative	keratosis	5.62E-05	52
Mammary gland	Rice straw	Negative	formation of lamellipodia	5.62E-05	67
Mammary gland	Rice straw	Negative	cell movement of endothelial cells	5.66E-05	136
Mammary gland	Rice straw	Negative	morphology of heart ventricle	5.87E-05	78
Mammary gland	Rice straw	Negative	cell death of tumor	5.88E-05	146
Mammary	Rice	Negative	autophagy of cells	5.88E-05	104

gland	straw				
Mammary gland	Rice straw	Negative	dephosphorylation of protein	5.97E-05	76
Mammary gland	Rice straw	Negative	development of abdomen	6.00E-05	216
Mammary gland	Rice straw	Negative	homing	6.03E-05	214
Mammary gland	Rice straw	Negative	mass of connective tissue	6.07E-05	81
Mammary gland	Rice straw	Negative	uptake of D-hexose	6.10E-05	89
Mammary gland	Rice straw	Negative	formation of actin stress fibers	6.10E-05	95
Mammary gland	Rice straw	Negative	secretion of molecule	6.10E-05	206
Mammary gland	Rice straw	Negative	cell movement of myeloid cells	6.13E-05	202
Mammary gland	Rice straw	Negative	metabolism of glycolipid	6.61E-05	63
Mammary gland	Rice straw	Negative	abnormal morphology of blood vessel	6.74E-05	104
Mammary gland	Rice straw	Negative	transactivation of RNA	6.76E-05	203
Mammary gland	Rice straw	Negative	quantity of metal	6.80E-05	187
Mammary gland	Rice straw	Negative	retinal degeneration	6.88E-05	101
Mammary gland	Rice straw	Negative	development of hematopoietic system	7.01E-05	98
Mammary gland	Rice straw	Negative	attachment of cells	7.37E-05	57
Mammary gland	Rice straw	Negative	concentration of phosphatidic acid	7.37E-05	57
Mammary gland	Rice straw	Negative	synthesis of DNA	7.37E-05	158
Mammary gland	Rice straw	Negative	mass of fat pad	7.40E-05	48
Mammary gland	Rice straw	Negative	infection of cells	8.04E-05	291
Mammary gland	Rice straw	Negative	homo-oligomerization of protein	8.08E-05	56
Mammary gland	Rice straw	Negative	metabolism of reactive oxygen species	8.29E-05	187
Mammary	Rice	Negative	experimentally-induced diabetes	8.29E-05	42

gland	straw				
Mammary gland	Rice straw	Negative	damage of nervous system	8.38E-05	89
Mammary gland	Rice straw	Negative	Waldenstrom's macroglobulinemia	8.45E-05	76
Mammary gland	Rice straw	Negative	apoptosis of macrophages	8.51E-05	46
Mammary gland	Rice straw	Negative	gastrointestinal adenocarcinoma	8.80E-05	1442
Mammary gland	Rice straw	Negative	cell movement of breast cancer cell lines	8.80E-05	112
Mammary gland	Rice straw	Negative	outgrowth of plasma membrane projections	8.83E-05	154
Mammary gland	Rice straw	Negative	formation of vascular lesion	8.83E-05	49
Mammary gland	Rice straw	Negative	morphology of connective tissue	8.83E-05	173
Mammary gland	Rice straw	Negative	growth of lymphoid organ	8.87E-05	70
Mammary gland	Rice straw	Negative	cardiogenesis	8.88E-05	177
Mammary gland	Rice straw	Negative	migration of vascular endothelial cells	8.90E-05	68
Mammary gland	Rice straw	Negative	aggregation of blood platelets	8.95E-05	77
Mammary gland	Rice straw	Negative	morphogenesis of neurites	8.98E-05	158
Mammary gland	Rice straw	Negative	morphology of genital organ	9.10E-05	160
Mammary gland	Rice straw	Negative	differentiation of connective tissue	9.35E-05	267
Mammary gland	Rice straw	Negative	migration of connective tissue cells	9.40E-05	73
Mammary gland	Rice straw	Negative	formation of lung	9.67E-05	116
Mammary gland	Rice straw	Negative	apoptosis of blood cells	9.84E-05	185
Mammary gland	Rice straw	Negative	apoptosis of carcinoma cell lines	9.85E-05	96
Mammary gland	Rice straw	Negative	morphogenesis of neurons	1.01E-04	160
Mammary gland	Rice straw	Negative	concentration of triacylglycerol	1.01E-04	118
Mammary	Rice	Negative	quantity of interleukin	1.02E-04	58

gland	straw				
Mammary gland	Rice straw	Negative	outgrowth of cells	1.02E-04	163
Mammary gland	Rice straw	Negative	activation of leukocytes	1.03E-04	256
Mammary gland	Rice straw	Negative	oligomerization of protein	1.04E-04	94
Mammary gland	Rice straw	Negative	epileptic seizure	1.07E-04	68
Mammary gland	Rice straw	Negative	degeneration of eye	1.07E-04	102
Mammary gland	Rice straw	Negative	necrosis of tumor	1.09E-04	144
Mammary gland	Rice straw	Negative	morphology of muscle	1.09E-04	128
Mammary gland	Rice straw	Negative	quantity of cytokine	1.10E-04	99
Mammary gland	Rice straw	Negative	chronic inflammatory disorder	1.11E-04	350
Mammary gland	Rice straw	Negative	sprouting	1.11E-04	146
Mammary gland	Rice straw	Negative	quantity of metal ion	1.14E-04	166
Mammary gland	Rice straw	Negative	apoptosis of leukocytes	1.16E-04	171
Mammary gland	Rice straw	Negative	gliosis of brain	1.16E-04	21
Mammary gland	Rice straw	Negative	paralysis	1.16E-04	67
Mammary gland	Rice straw	Negative	blood protein disorder	1.18E-04	142
Mammary gland	Rice straw	Negative	neurodegeneration of central nervous system	1.18E-04	48
Mammary gland	Rice straw	Negative	spatial learning	1.19E-04	74
Mammary gland	Rice straw	Negative	proliferation of mononuclear leukocytes	1.22E-04	269
Mammary gland	Rice straw	Negative	differentiation of muscle	1.22E-04	116
Mammary gland	Rice straw	Negative	abnormal morphology of skin	1.24E-04	95
Mammary gland	Rice straw	Negative	long-term potentiation	1.24E-04	95
Mammary	Rice	Negative	cell death of macrophages	1.25E-04	53

gland	straw				
Mammary gland	Rice straw	Negative	formation of thymus gland	1.28E-04	66
Mammary gland	Rice straw	Negative	outgrowth of neurites	1.28E-04	152
Mammary gland	Rice straw	Negative	internalization of protein	1.29E-04	43
Mammary gland	Rice straw	Negative	cell movement of phagocytes	1.34E-04	203
Mammary gland	Rice straw	Negative	proliferation of dermal cells	1.34E-04	71
Mammary gland	Rice straw	Negative	quantity of T lymphocytes	1.36E-04	198
Mammary gland	Rice straw	Negative	chronic psoriasis	1.38E-04	59
Mammary gland	Rice straw	Negative	proliferation of tumor cells	1.40E-04	175
Mammary gland	Rice straw	Negative	formation of focal adhesions	1.40E-04	63
Mammary gland	Rice straw	Negative	apoptosis of renal glomerulus	1.40E-04	27
Mammary gland	Rice straw	Negative	targeting of protein	1.42E-04	54
Mammary gland	Rice straw	Negative	outgrowth of neurons	1.43E-04	153
Mammary gland	Rice straw	Negative	vascularization	1.45E-04	92
Mammary gland	Rice straw	Negative	apoptosis of skin	1.45E-04	41
Mammary gland	Rice straw	Negative	cell death of renal glomerulus	1.45E-04	28
Mammary gland	Rice straw	Negative	skin abnormality	1.47E-04	89
Mammary gland	Rice straw	Negative	chemotaxis of cells	1.48E-04	195
Mammary gland	Rice straw	Negative	apoptosis of stem cells	1.51E-04	32
Mammary gland	Rice straw	Negative	angiogenesis of lesion	1.55E-04	53
Mammary gland	Rice straw	Negative	Cytosis	1.55E-04	122
Mammary gland	Rice straw	Negative	abnormal morphology of genital organ	1.58E-04	153
Mammary	Rice	Negative	prostatic intraepithelial neoplasia	1.59E-04	50

gland	straw				
Mammary gland	Rice straw	Negative	neurodegeneration of brain	1.59E-04	47
Mammary gland	Rice straw	Negative	advanced malignant tumor	1.61E-04	305
Mammary gland	Rice straw	Negative	proliferation of lymphocytes	1.71E-04	264
Mammary gland	Rice straw	Negative	stabilization of filaments	1.73E-04	46
Mammary gland	Rice straw	Negative	cell death of heart cells	1.73E-04	86
Mammary gland	Rice straw	Negative	mass of adipose tissue	1.77E-04	75
Mammary gland	Rice straw	Negative	cell death of dermal cells	1.78E-04	42
Mammary gland	Rice straw	Negative	differentiation of epidermal cells	1.84E-04	66
Mammary gland	Rice straw	Negative	synthesis of protein	1.84E-04	166
Mammary gland	Rice straw	Negative	craniofacial abnormality	1.86E-04	134
Mammary gland	Rice straw	Negative	infection by Retroviridae	1.90E-04	261
Mammary gland	Rice straw	Negative	expression of protein	1.93E-04	128
Mammary gland	Rice straw	Negative	cell death of carcinoma cell lines	1.93E-04	109
Mammary gland	Rice straw	Negative	cell death of liver cells	2.01E-04	77
Mammary gland	Rice straw	Negative	abnormal morphology of body wall	2.01E-04	44
Mammary gland	Rice straw	Negative	differentiation of endothelial cells	2.01E-04	34
Mammary gland	Rice straw	Negative	uptake of D-glucose	2.01E-04	86
Mammary gland	Rice straw	Negative	migration of endothelial cells	2.03E-04	124
Mammary gland	Rice straw	Negative	differentiation of keratinocytes	2.05E-04	61
Mammary gland	Rice straw	Negative	fusion of vesicles	2.06E-04	33
Mammary gland	Rice straw	Negative	development of body axis	2.06E-04	374
Mammary	Rice	Negative	proliferation of hepatoma cell lines	2.11E-04	78

gland	straw				
Mammary gland	Rice straw	Negative	fusion of cellular membrane	2.11E-04	28
Mammary gland	Rice straw	Negative	juvenile dermatomyositis	2.11E-04	28
Mammary gland	Rice straw	Negative	abnormal morphology of rib cage	2.12E-04	39
Mammary gland	Rice straw	Negative	apoptosis of dermal cells	2.12E-04	39
Mammary gland	Rice straw	Negative	morphology of lymphatic system component	2.15E-04	155
Mammary gland	Rice straw	Negative	cell death of sarcoma cell lines	2.17E-04	88
Mammary gland	Rice straw	Negative	concentration of sterol	2.23E-04	116
Mammary gland	Rice straw	Negative	chemotaxis	2.24E-04	200
Mammary gland	Rice straw	Negative	morphology of lung	2.29E-04	74
Mammary gland	Rice straw	Negative	I-kappaB kinase/NF-kappaB cascade	2.29E-04	69
Mammary gland	Rice straw	Negative	export of molecule	2.32E-04	112
Mammary gland	Rice straw	Negative	cell viability of fibroblast cell lines	2.34E-04	48
Mammary gland	Rice straw	Negative	abnormal morphology of lung	2.38E-04	72
Mammary gland	Rice straw	Negative	incidence of tumor	2.43E-04	121
Mammary gland	Rice straw	Negative	quantity of lymphatic system component	2.44E-04	141
Mammary gland	Rice straw	Negative	cell death of heart	2.52E-04	88
Mammary gland	Rice straw	Negative	apoptosis of muscle	2.52E-04	100
Mammary gland	Rice straw	Negative	formation of pathological cyst	2.54E-04	47
Mammary gland	Rice straw	Negative	cell death of liver	2.54E-04	92
Mammary gland	Rice straw	Negative	exocytosis by cells	2.57E-04	52
Mammary gland	Rice straw	Negative	activation of neuroglia	2.65E-04	54
Mammary	Rice	Negative	growth of plasma membrane projections	2.67E-04	172

gland	straw				
Mammary gland	Rice straw	Negative	accumulation of lipid	2.67E-04	113
Mammary gland	Rice straw	Negative	consumption of oxygen	2.68E-04	62
Mammary gland	Rice straw	Negative	Pathological Cyst	2.68E-04	56
Mammary gland	Rice straw	Negative	morphology of pulmonary alveolus	2.68E-04	49
Mammary gland	Rice straw	Negative	arrest in proliferation of cells	2.69E-04	83
Mammary gland	Rice straw	Negative	morphology of rib	2.79E-04	33
Mammary gland	Rice straw	Negative	cell movement of blood cells	2.79E-04	313
Mammary gland	Rice straw	Negative	cell movement of leukocytes	2.79E-04	276
Mammary gland	Rice straw	Negative	concentration of cholesterol	2.82E-04	110
Mammary gland	Rice straw	Negative	cell death of breast cancer cell lines	2.86E-04	125
Mammary gland	Rice straw	Negative	production of HIV	2.98E-04	27
Mammary gland	Rice straw	Negative	migration of blood cells	3.03E-04	312
Mammary gland	Rice straw	Negative	quantity of protein in blood	3.08E-04	186
Mammary gland	Rice straw	Negative	polarization of tumor cell lines	3.08E-04	21
Mammary gland	Rice straw	Negative	differentiation of skin	3.10E-04	71
Mammary gland	Rice straw	Negative	failure of heart	3.11E-04	94
Mammary gland	Rice straw	Negative	familial Alzheimer's disease	3.11E-04	16
Mammary gland	Rice straw	Negative	breast or ovarian cancer	3.12E-04	659
Mammary gland	Rice straw	Negative	apoptosis of heart cells	3.14E-04	74
Mammary gland	Rice straw	Negative	apoptosis of muscle cells	3.15E-04	99
Mammary gland	Rice straw	Negative	proliferation of endothelial cells	3.20E-04	117
Mammary	Rice	Negative	necrosis of liver	3.21E-04	91

gland	straw				
Mammary gland	Rice straw	Negative	apoptosis of antigen presenting cells	3.25E-04	56
Mammary gland	Rice straw	Negative	apoptosis of fibroblasts	3.25E-04	84
Mammary gland	Rice straw	Negative	neoplasia of tumor cell lines	3.29E-04	119
Mammary gland	Rice straw	Negative	metastasis	3.30E-04	273
Mammary gland	Rice straw	Negative	cell death of embryonic cell lines	3.31E-04	92
Mammary gland	Rice straw	Negative	hyperkeratosis	3.33E-04	40
Mammary gland	Rice straw	Negative	demyelination of central nervous system	3.40E-04	22
Mammary gland	Rice straw	Negative	glandular intraepithelial neoplasm	3.42E-04	51
Mammary gland	Rice straw	Negative	homologous recombination	3.42E-04	51
Mammary gland	Rice straw	Negative	leukocyte migration	3.43E-04	311
Mammary gland	Rice straw	Negative	apoptosis of liver	3.44E-04	63
Mammary gland	Rice straw	Negative	cell death of tumor cells	3.58E-04	138
Mammary gland	Rice straw	Negative	colon cancer	3.59E-04	1246
Mammary gland	Rice straw	Negative	quantity of muscle cells	3.60E-04	48
Mammary gland	Rice straw	Negative	colorectal carcinoma	3.64E-04	1247
Mammary gland	Rice straw	Negative	infection by lentivirus	3.69E-04	257
Mammary gland	Rice straw	Negative	cell movement of antigen presenting cells	3.73E-04	130
Mammary gland	Rice straw	Negative	cell death of neuroblastoma cell lines	3.87E-04	75
Mammary gland	Rice straw	Negative	advanced stage peripheral arterial disease	3.89E-04	60
Mammary gland	Rice straw	Negative	transport of amino acids	3.91E-04	54
Mammary gland	Rice straw	Negative	apoptosis of glomerular cells	3.91E-04	24
Mammary	Rice	Negative	abnormal morphology of pulmonary alveolus	3.92E-04	47

gland	straw				
Mammary gland	Rice straw	Negative	branching of cells	3.93E-04	137
Mammary gland	Rice straw	Negative	abnormal morphology of rib	3.99E-04	31
Mammary gland	Rice straw	Negative	HIV infection	4.00E-04	256
Mammary gland	Rice straw	Negative	modification of peptide	4.00E-04	44
Mammary gland	Rice straw	Negative	necrosis of renal glomerulus	4.05E-04	25
Mammary gland	Rice straw	Negative	angiogenesis of tumor	4.07E-04	49
Mammary gland	Rice straw	Negative	plasticity of synapse	4.07E-04	49
Mammary gland	Rice straw	Negative	apoptosis of mesangial cells	4.10E-04	15
Mammary gland	Rice straw	Negative	formation of eye	4.17E-04	161
Mammary gland	Rice straw	Negative	proliferation of vascular smooth muscle cells	4.18E-04	61
Mammary gland	Rice straw	Negative	migration of fibroblasts	4.29E-04	57
Mammary gland	Rice straw	Negative	polymerization of protein	4.35E-04	137
Mammary gland	Rice straw	Negative	differentiation of tumor cell lines	4.42E-04	140
Mammary gland	Rice straw	Negative	repair of DNA	4.43E-04	109
Mammary gland	Rice straw	Negative	neoplasia of colon	4.45E-04	1248
Mammary gland	Rice straw	Negative	growth of neurites	4.53E-04	169
Mammary gland	Rice straw	Negative	synthesis of nitric oxide	4.53E-04	104
Mammary gland	Rice straw	Negative	maturation of cells	4.54E-04	167
Mammary gland	Rice straw	Negative	cell death of epidermal cells	4.55E-04	39
Mammary gland	Rice straw	Negative	cell death of cardiomyocytes	4.55E-04	82
Mammary gland	Rice straw	Negative	abnormal morphology of lymph node	4.59E-04	60
Mammary	Rice	Negative	accumulation of blood cells	4.59E-04	105

gland	straw				
Mammary gland	Corn stover	Positive	proliferation of cells	3.05E-25	940
Mammary gland	Corn stover	Positive	organismal death	8.39E-24	650
Mammary gland	Corn stover	Positive	morbidity or mortality	2.01E-23	655
Mammary gland	Corn stover	Positive	cell death	7.18E-20	836
Mammary gland	Corn stover	Positive	necrosis	4.16E-19	667
Mammary gland	Corn stover	Positive	morphology of cells	1.90E-18	525
Mammary gland	Corn stover	Positive	cell movement	9.18E-17	558
Mammary gland	Corn stover	Positive	apoptosis	6.22E-16	666
Mammary gland	Corn stover	Positive	cancer	1.34E-15	2309
Mammary gland	Corn stover	Positive	transport of molecule	8.92E-15	433
Mammary gland	Corn stover	Positive	malignant solid tumor	2.50E-14	2277
Mammary gland	Corn stover	Positive	organization of cytoplasm	9.96E-14	410
Mammary gland	Corn stover	Positive	migration of cells	1.43E-13	492
Mammary gland	Corn stover	Positive	Viral Infection	6.83E-13	424
Mammary gland	Corn stover	Positive	cellular homeostasis	1.41E-12	402
Mammary gland	Corn stover	Positive	angiogenesis	1.45E-12	260
Mammary gland	Corn stover	Positive	development of body trunk	5.45E-12	307
Mammary gland	Corn stover	Positive	organization of cytoskeleton	1.82E-11	368
Mammary gland	Corn stover	Positive	vasculogenesis	9.78E-11	212
Mammary gland	Corn stover	Positive	differentiation of cells	1.41E-10	551
Mammary gland	Corn stover	Positive	abnormal morphology of cells	1.66E-10	334
Mammary	Corn	Positive	expression of RNA	3.59E-10	526

gland	stover				
Mammary gland	Corn stover	Positive	morphology of cardiovascular system	3.72E-10	181
Mammary gland	Corn stover	Positive	formation of cellular protrusions	3.72E-10	249
Mammary gland	Corn stover	Positive	abdominal neoplasm	4.74E-10	1878
Mammary gland	Corn stover	Positive	perinatal death	1.07E-09	173
Mammary gland	Corn stover	Positive	abdominal cancer	1.07E-09	1855
Mammary gland	Corn stover	Positive	tumorigenesis of tissue	1.16E-09	1917
Mammary gland	Corn stover	Positive	microtubule dynamics	1.19E-09	311
Mammary gland	Corn stover	Positive	neoplasia of epithelial tissue	1.46E-09	1887
Mammary gland	Corn stover	Positive	cell movement of tumor cell lines	1.57E-09	234
Mammary gland	Corn stover	Positive	cell death of tumor cell lines	4.62E-09	384
Mammary gland	Corn stover	Positive	epithelial cancer	5.13E-09	1866
Mammary gland	Corn stover	Positive	transcription	6.22E-09	485
Mammary gland	Corn stover	Positive	abnormal morphology of cardiovascular system	7.97E-09	163
Mammary gland	Corn stover	Positive	transcription of RNA	1.03E-08	454
Mammary gland	Corn stover	Positive	digestive system cancer	1.66E-08	1611
Mammary gland	Corn stover	Positive	apoptosis of tumor cell lines	1.72E-08	311
Mammary gland	Corn stover	Positive	replication of virus	1.85E-08	160
Mammary gland	Corn stover	Positive	digestive organ tumor	2.07E-08	1624
Mammary gland	Corn stover	Positive	size of body	2.93E-08	228
Mammary gland	Corn stover	Positive	concentration of lipid	4.20E-08	218
Mammary gland	Corn stover	Positive	replication of RNA virus	4.58E-08	145
Mammary	Corn	Positive	necrosis of epithelial tissue	4.68E-08	165

gland	stover				
Mammary gland	Corn stover	Positive	proliferation of connective tissue cells	4.68E-08	166
Mammary gland	Corn stover	Positive	quantity of cells	5.10E-08	430
Mammary gland	Corn stover	Positive	autophagy of cells	5.41E-08	84
Mammary gland	Corn stover	Positive	autophagy	5.41E-08	112
Mammary gland	Corn stover	Positive	autosomal recessive disease	8.08E-08	242
Mammary gland	Corn stover	Positive	cell death of connective tissue cells	9.75E-08	167
Mammary gland	Corn stover	Positive	transactivation	1.01E-07	161
Mammary gland	Corn stover	Positive	development of vasculature	1.01E-07	122
Mammary gland	Corn stover	Positive	dyskinesia	1.14E-07	170
Mammary gland	Corn stover	Positive	growth of organism	1.47E-07	221
Mammary gland	Corn stover	Positive	morphology of heart	1.70E-07	118
Mammary gland	Corn stover	Positive	neurological signs	2.16E-07	177
Mammary gland	Corn stover	Positive	Movement Disorders	3.04E-07	270
Mammary gland	Corn stover	Positive	morphology of body cavity	4.83E-07	299
Mammary gland	Corn stover	Positive	abnormal morphology of thoracic cavity	5.36E-07	160
Mammary gland	Corn stover	Positive	Huntington's Disease	5.76E-07	157
Mammary gland	Corn stover	Positive	cell death of epithelial cells	7.12E-07	138
Mammary gland	Corn stover	Positive	Organ Degeneration	7.68E-07	137
Mammary gland	Corn stover	Positive	organization of organelle	7.77E-07	148
Mammary gland	Corn stover	Positive	migration of tumor cell lines	9.90E-07	187
Mammary gland	Corn stover	Positive	growth of connective tissue	1.22E-06	172
Mammary	Corn	Positive	cardiogenesis	1.25E-06	131

gland	stover				
Mammary gland	Corn stover	Positive	ubiquitination	1.25E-06	98
Mammary gland	Corn stover	Positive	transactivation of RNA	1.32E-06	148
Mammary gland	Corn stover	Positive	quantity of connective tissue	1.32E-06	162
Mammary gland	Corn stover	Positive	morphology of nervous system	1.38E-06	204
Mammary gland	Corn stover	Positive	fibrogenesis	1.41E-06	116
Mammary gland	Corn stover	Positive	seizures	1.42E-06	113
Mammary gland	Corn stover	Positive	abnormal morphology of body cavity	1.63E-06	284
Mammary gland	Corn stover	Positive	cell death of epithelial cell lines	1.66E-06	84
Mammary gland	Corn stover	Positive	ubiquitination of protein	1.94E-06	96
Mammary gland	Corn stover	Positive	morphology of head	2.13E-06	224
Mammary gland	Corn stover	Positive	function of cardiovascular system	2.28E-06	105
Mammary gland	Corn stover	Positive	abnormal morphology of nervous system	2.58E-06	189
Mammary gland	Corn stover	Positive	development of cytoplasm	2.87E-06	127
Mammary gland	Corn stover	Positive	protein kinase cascade	3.24E-06	117
Mammary gland	Corn stover	Positive	abnormal morphology of head	3.46E-06	213
Mammary gland	Corn stover	Positive	transcription of DNA	3.66E-06	367
Mammary gland	Corn stover	Positive	formation of filaments	3.75E-06	111
Mammary gland	Corn stover	Positive	behavior	3.77E-06	248
Mammary gland	Corn stover	Positive	cell viability	3.85E-06	316
Mammary gland	Corn stover	Positive	invasion of cells	3.93E-06	221
Mammary gland	Corn stover	Positive	proliferation of fibroblast cell lines	4.17E-06	117
Mammary	Corn	Positive	seizure disorder	4.42E-06	128

gland	stover				
Mammary gland	Corn stover	Positive	morphology of heart ventricle	4.61E-06	60
Mammary gland	Corn stover	Positive	proliferation of neuronal cells	4.61E-06	153
Mammary gland	Corn stover	Positive	abnormal morphology of heart	4.82E-06	107
Mammary gland	Corn stover	Positive	cell death of embryonic cell lines	4.82E-06	72
Mammary gland	Corn stover	Positive	infection by Retroviridae	5.12E-06	186
Mammary gland	Corn stover	Positive	formation of cytoskeleton	5.55E-06	105
Mammary gland	Corn stover	Positive	metabolism of carbohydrate	5.81E-06	163
Mammary gland	Corn stover	Positive	formation of plasma membrane projections	6.62E-06	157
Mammary gland	Corn stover	Positive	development of abdomen	6.75E-06	153
Mammary gland	Corn stover	Positive	proliferation of fibroblasts	6.95E-06	99
Mammary gland	Corn stover	Positive	development of digestive system	8.65E-06	106
Mammary gland	Corn stover	Positive	long-term potentiation of synapse	8.96E-06	48
Mammary gland	Corn stover	Positive	neuronal cell death	9.02E-06	176
Mammary gland	Corn stover	Positive	neuritogenesis	1.00E-05	153
Mammary gland	Corn stover	Positive	infection by lentivirus	1.17E-05	183
Mammary gland	Corn stover	Positive	abnormal morphology of respiratory system	1.18E-05	87
Mammary gland	Corn stover	Positive	morphology of respiratory system	1.23E-05	88
Mammary gland	Corn stover	Positive	neuromuscular disease	1.23E-05	221
Mammary gland	Corn stover	Positive	branching of cells	1.27E-05	102
Mammary gland	Corn stover	Positive	cell survival	1.34E-05	332
Mammary gland	Corn stover	Positive	abnormal morphology of reproductive system	1.34E-05	135
Mammary	Corn	Positive	cognition	1.39E-05	122

gland	stover				
Mammary gland	Corn stover	Positive	HIV infection	1.39E-05	182
Mammary gland	Corn stover	Positive	morphology of reproductive system	1.41E-05	141
Mammary gland	Corn stover	Positive	infection of embryonic cell lines	1.48E-05	79
Mammary gland	Corn stover	Positive	infection of epithelial cell lines	1.48E-05	79
Mammary gland	Corn stover	Positive	development of lymphatic system component	1.56E-05	87
Mammary gland	Corn stover	Positive	cell death of cervical cancer cell lines	1.61E-05	97
Mammary gland	Corn stover	Positive	congenital anomaly of musculoskeletal system	1.63E-05	187
Mammary gland	Corn stover	Positive	growth of embryo	1.64E-05	127
Mammary gland	Corn stover	Positive	synthesis of lipid	1.64E-05	182
Mammary gland	Corn stover	Positive	abnormal morphology of heart ventricle	1.67E-05	54
Mammary gland	Corn stover	Positive	neonatal death	1.67E-05	118
Mammary gland	Corn stover	Positive	autophagy of tumor cell lines	1.67E-05	47
Mammary gland	Corn stover	Positive	function of leukocytes	1.69E-05	141
Mammary gland	Corn stover	Positive	Hypertrophy	1.71E-05	126
Mammary gland	Corn stover	Positive	cell death of fibroblast cell lines	1.74E-05	115
Mammary gland	Corn stover	Positive	development of neurons	1.89E-05	197
Mammary gland	Corn stover	Positive	development of lymphatic system	1.91E-05	98
Mammary gland	Corn stover	Positive	function of blood cells	2.09E-05	152
Mammary gland	Corn stover	Positive	apoptosis of connective tissue cells	2.46E-05	83
Mammary gland	Corn stover	Positive	learning	2.71E-05	112
Mammary gland	Corn stover	Positive	differentiation of muscle cell lines	2.83E-05	50
Mammary	Corn	Positive	formation of lung	2.87E-05	84

gland	stover				
Mammary gland	Corn stover	Positive	apoptosis of neuroblastoma cell lines	2.90E-05	40
Mammary gland	Corn stover	Positive	long-term potentiation	3.08E-05	70
Mammary gland	Corn stover	Positive	differentiation of connective tissue cells	3.20E-05	164
Mammary gland	Corn stover	Positive	proliferation of tumor cell lines	3.20E-05	362
Mammary gland	Corn stover	Positive	necrosis of kidney	3.20E-05	99
Mammary gland	Corn stover	Positive	formation of lymphoid organ	3.23E-05	75
Mammary gland	Corn stover	Positive	quantity of blood cells	3.24E-05	250
Mammary gland	Corn stover	Positive	abnormal morphology of embryonic tissue	3.27E-05	142
Mammary gland	Corn stover	Positive	epileptic seizure	3.76E-05	51
Mammary gland	Corn stover	Positive	apoptosis of fibroblast cell lines	3.80E-05	90
Mammary gland	Corn stover	Positive	quantity of leukocytes	3.80E-05	225
Mammary gland	Corn stover	Positive	growth of epithelial tissue	3.83E-05	172
Mammary gland	Corn stover	Positive	cell death of kidney cells	3.85E-05	95
Mammary gland	Corn stover	Positive	infection by RNA virus	3.92E-05	214
Mammary gland	Corn stover	Positive	abnormal morphology of internal genitalia	4.25E-05	95
Mammary gland	Corn stover	Positive	hypertrophy of heart	4.37E-05	92
Mammary gland	Corn stover	Positive	Growth Failure	4.43E-05	153
Mammary gland	Corn stover	Positive	cell movement of muscle cells	4.63E-05	54
Mammary gland	Corn stover	Positive	formation of lymphatic system component	4.65E-05	81
Mammary gland	Corn stover	Positive	abnormal morphology of epithelial tissue	4.69E-05	111
Mammary gland	Corn stover	Positive	infection of kidney cell lines	4.74E-05	79
Mammary	Corn	Positive	necrosis of muscle	4.83E-05	93

gland	stover				
Mammary gland	Corn stover	Positive	apoptosis of heart cells	4.91E-05	56
Mammary gland	Corn stover	Positive	cell death of muscle cells	5.67E-05	91
Mammary gland	Corn stover	Positive	size of embryo	5.67E-05	96
Mammary gland	Corn stover	Positive	uptake of monosaccharide	6.26E-05	75
Mammary gland	Corn stover	Positive	apoptosis of epithelial cell lines	6.79E-05	64
Mammary gland	Corn stover	Positive	cell death of kidney cell lines	6.85E-05	83
Mammary gland	Corn stover	Positive	dephosphorylation of protein	6.85E-05	55
Mammary gland	Corn stover	Positive	infection of cells	6.85E-05	197
Mammary gland	Corn stover	Positive	metabolism of monosaccharide	6.96E-05	39
Mammary gland	Corn stover	Positive	morphology of blood vessel	7.00E-05	78
Mammary gland	Corn stover	Positive	disorder of basal ganglia	7.03E-05	190
Mammary gland	Corn stover	Positive	respiratory system development	7.66E-05	92
Mammary gland	Corn stover	Positive	concentration of fatty acid	8.44E-05	77
Mammary gland	Corn stover	Positive	abnormal morphology of abdomen	8.45E-05	214
Mammary gland	Corn stover	Positive	abnormal morphology of digestive system	8.45E-05	139
Mammary gland	Corn stover	Positive	morphology of testis	8.57E-05	75
Mammary gland	Corn stover	Positive	attachment of cells	8.68E-05	42
Mammary gland	Corn stover	Positive	Bleeding	9.08E-05	111
Mammary gland	Corn stover	Positive	congenital heart disease	9.23E-05	60
Mammary gland	Corn stover	Positive	cell cycle progression	9.23E-05	251
Mammary gland	Corn stover	Positive	limb development	9.56E-05	66
Mammary	Corn	Positive	replication of Influenza virus	9.85E-05	79

gland	stover				
Mammary gland	Corn stover	Positive	mass of adipose tissue	1.01E-04	55
Mammary gland	Corn stover	Positive	ruffling	1.05E-04	35
Mammary gland	Corn stover	Positive	dendritic growth/branching	1.06E-04	62
Mammary gland	Corn stover	Positive	cell death of fibroblasts	1.07E-04	73
Mammary gland	Corn stover	Positive	migration of muscle cells	1.07E-04	49
Mammary gland	Corn stover	Positive	urination disorder	1.07E-04	79
Mammary gland	Corn stover	Positive	apoptosis of embryonic cell lines	1.08E-04	53
Mammary gland	Corn stover	Positive	cell movement of smooth muscle cells	1.09E-04	48
Mammary gland	Corn stover	Positive	metabolism of membrane lipid derivative	1.09E-04	102
Mammary gland	Corn stover	Positive	morphology of connective tissue	1.09E-04	119
Mammary gland	Corn stover	Positive	apoptosis of cervical cancer cell lines	1.09E-04	77
Mammary gland	Corn stover	Positive	proliferation of muscle cells	1.14E-04	98
Mammary gland	Corn stover	Positive	cell death of heart	1.17E-04	64
Mammary gland	Corn stover	Positive	serous neoplasm	1.22E-04	153
Mammary gland	Corn stover	Positive	cell death of heart cells	1.32E-04	62
Mammary gland	Corn stover	Positive	development of gastrointestinal tract	1.41E-04	72
Mammary gland	Corn stover	Positive	cell movement of fibroblasts	1.41E-04	53
Mammary gland	Corn stover	Positive	morphology of genital organ	1.42E-04	110
Mammary gland	Corn stover	Positive	proteinuria	1.46E-04	48
Mammary gland	Corn stover	Positive	organization of actin cytoskeleton	1.51E-04	81
Mammary gland	Corn stover	Positive	sprouting	1.51E-04	101
Mammary	Corn	Positive	development of head	1.61E-04	235

gland	stover				
Mammary gland	Corn stover	Positive	small GTPase mediated signal transduction	1.61E-04	53
Mammary gland	Corn stover	Positive	secretion of molecule	1.61E-04	139
Mammary gland	Corn stover	Positive	apoptosis of kidney cell lines	1.64E-04	66
Mammary gland	Corn stover	Positive	multiple congenital anomalies	1.80E-04	109
Mammary gland	Corn stover	Positive	development of body axis	1.80E-04	250
Mammary gland	Corn stover	Positive	mass of fat pad	1.80E-04	35
Mammary gland	Corn stover	Positive	differentiation of connective tissue	1.81E-04	179
Mammary gland	Corn stover	Positive	apoptosis of cardiomyocytes	1.81E-04	53
Mammary gland	Corn stover	Positive	apoptosis of muscle cells	1.81E-04	71
Mammary gland	Corn stover	Positive	transport of alpha-amino acid	1.81E-04	25
Mammary gland	Corn stover	Positive	congenital anomaly of digestive system	1.83E-04	64
Mammary gland	Corn stover	Positive	growth of lesion	1.88E-04	194
Mammary gland	Corn stover	Positive	autosomal dominant disease	1.94E-04	158
Mammary gland	Corn stover	Positive	morphology of digestive system	1.94E-04	143
Mammary gland	Corn stover	Positive	transport of metal	2.04E-04	88
Mammary gland	Corn stover	Positive	uptake of D-hexose	2.06E-04	62
Mammary gland	Corn stover	Positive	midline defect	2.07E-04	72
Mammary gland	Corn stover	Positive	transport of carbohydrate	2.10E-04	55
Mammary gland	Corn stover	Positive	migration of smooth muscle cells	2.10E-04	44
Mammary gland	Corn stover	Positive	congenital anomaly of cardiovascular system	2.15E-04	63
Mammary gland	Corn stover	Positive	replication of Influenza A virus	2.15E-04	77
Mammary	Corn	Positive	activation of neuroglia	2.20E-04	40

gland	stover				
Mammary gland	Corn stover	Positive	transport of cation	2.20E-04	104
Mammary gland	Corn stover	Positive	growth of tumor	2.35E-04	193
Mammary gland	Corn stover	Positive	formation of actin filaments	2.36E-04	81
Mammary gland	Corn stover	Positive	development of epithelial tissue	2.47E-04	130
Mammary gland	Corn stover	Positive	function of muscle	2.47E-04	91
Mammary gland	Corn stover	Positive	transport of inorganic cation	2.49E-04	92
Mammary gland	Corn stover	Positive	hepatic steatosis	2.49E-04	72
Mammary gland	Corn stover	Positive	quantity of cytokine	2.49E-04	69
Mammary gland	Corn stover	Positive	development of cardiovascular tissue	2.53E-04	96
Mammary gland	Corn stover	Positive	abnormal morphology of genital organ	2.54E-04	105
Mammary gland	Corn stover	Positive	cognitive impairment	2.54E-04	87
Mammary gland	Corn stover	Positive	infection by HIV-1	2.56E-04	151
Mammary gland	Corn stover	Positive	cell transformation	2.56E-04	117
Mammary gland	Corn stover	Positive	uptake of D-glucose	2.66E-04	61
Mammary gland	Corn stover	Positive	length of cells	2.69E-04	32
Mammary gland	Corn stover	Positive	phosphorylation of protein	2.69E-04	179
Mammary gland	Corn stover	Positive	breast or ovarian cancer	2.69E-04	432
Mammary gland	Corn stover	Positive	morphology of brain	2.71E-04	118
Mammary gland	Corn stover	Positive	transport of ion	2.84E-04	127
Mammary gland	Corn stover	Positive	formation of thymus gland	2.86E-04	47
Mammary gland	Corn stover	Positive	congenital malformation of skeleton	2.89E-04	108
Mammary	Corn	Positive	development of connective tissue	2.90E-04	96

gland	stover				
Mammary gland	Corn stover	Positive	cell movement of sarcoma cell lines	2.90E-04	30
Mammary gland	Corn stover	Positive	growth of neurites	2.90E-04	117
Mammary gland	Corn stover	Positive	formation of muscle	2.90E-04	102
Mammary gland	Corn stover	Positive	formation of leukocytes	2.90E-04	46
Mammary gland	Corn stover	Positive	differentiation of muscle	2.90E-04	80
Mammary gland	Corn stover	Positive	proliferation of smooth muscle cells	2.93E-04	76
Mammary gland	Corn stover	Positive	morphology of vessel	2.94E-04	81
Mammary gland	Corn stover	Positive	invasion of tumor cell lines	2.95E-04	163
Mammary gland	Corn stover	Positive	mass of connective tissue	3.05E-04	56
Mammary gland	Corn stover	Positive	necrosis of cardiac muscle	3.05E-04	60
Mammary gland	Corn stover	Positive	biosynthesis of purine ribonucleotide	3.15E-04	32
Mammary gland	Corn stover	Positive	cell movement of connective tissue cells	3.21E-04	61
Mammary gland	Corn stover	Positive	cell death of cardiomyocytes	3.24E-04	59
Mammary gland	Corn stover	Positive	uptake of carbohydrate	3.26E-04	77
Mammary gland	Corn stover	Positive	differentiation of epithelial tissue	3.29E-04	93
Mammary gland	Corn stover	Positive	abnormal morphology of testis	3.31E-04	70
Mammary gland	Corn stover	Positive	abnormal morphology of blood vessel	3.41E-04	71
Mammary gland	Corn stover	Positive	morphology of lymphatic system component	3.54E-04	106
Mammary gland	Corn stover	Positive	morphology of central nervous system	3.82E-04	128
Mammary gland	Corn stover	Positive	metabolism of nucleoside triphosphate	3.84E-04	41
Mammary gland	Corn stover	Positive	biosynthesis of nucleoside triphosphate	3.90E-04	33
Mammary	Corn	Positive	mass of heart	3.90E-04	40

gland	stover				
Mammary gland	Corn stover	Positive	syndromic mental retardation	3.93E-04	45
Mammary gland	Corn stover	Positive	homing of cells	3.94E-04	138
Mammary gland	Corn stover	Positive	quantity of carbohydrate	3.97E-04	129
Mammary gland	Corn stover	Positive	cell viability of pheochromocytoma cell lines	3.97E-04	16
Mammary gland	Corn stover	Positive	quantity of epithelial tissue	4.14E-04	50
Mammary gland	Corn stover	Positive	interphase	4.15E-04	160
Mammary gland	Corn stover	Positive	morphogenesis of neurites	4.44E-04	106
Mammary gland	Corn stover	Positive	cell viability of tumor cell lines	4.54E-04	187
Mammary gland	Corn stover	Positive	endothelial cell development	4.54E-04	91
Mammary gland	Corn stover	Positive	length of neurons	4.62E-04	28
Mammary gland	Corn stover	Positive	transport of metal ion	4.68E-04	83
Mammary gland	Corn stover	Positive	formation of filopodia	4.75E-04	47
Mammary gland	Corn stover	Positive	benign neoplasia	4.77E-04	219
Mammary gland	Corn stover	Positive	cell spreading	4.78E-04	73
Mammary gland	Corn stover	Positive	mental retardation	4.79E-04	63
Mammary gland	Corn stover	Positive	activation of DNA endogenous promoter	4.86E-04	280
Mammary gland	Corn stover	Positive	development of urinary tract	4.96E-04	75
Mammary gland	Corn stover	Positive	quantity of protein in blood	5.01E-04	126
Mammary gland	Corn stover	Positive	homing	5.03E-04	141
Mammary gland	Corn stover	Positive	differentiation of muscle cells	5.36E-04	74
Mammary gland	Corn stover	Positive	morphology of lung	5.38E-04	52
Mammary	Corn	Positive	MAPKKK cascade	5.39E-04	56

gland	stover				
Mammary gland	Corn stover	Positive	morphogenesis of neurons	5.40E-04	107
Mammary gland	Corn stover	Positive	hypertrophy of tissue	5.64E-04	68
Mammary gland	Corn stover	Positive	formation of cardiac muscle	5.93E-04	29
Mammary gland	Corn stover	Positive	degeneration of nervous system	5.99E-04	75
Mammary gland	Corn stover	Positive	chemotaxis of cells	6.13E-04	130
Mammary gland	Corn stover	Positive	formation of actin stress fibers	6.13E-04	64
Mammary gland	Corn stover	Positive	morphology of gonad	6.19E-04	96
Mammary gland	Corn stover	Positive	cell death of neuroblastoma cell lines	6.46E-04	53
Mammary gland	Corn stover	Positive	formation of thymocytes	6.46E-04	32
Mammary gland	Corn stover	Positive	development of endothelial tissue	6.76E-04	93
Mammary gland	Corn stover	Positive	migration of connective tissue cells	6.79E-04	50
Mammary gland	Corn stover	Positive	formation of kidney	6.97E-04	73
Mammary gland	Corn stover	Positive	abnormal morphology of bone	7.13E-04	109
Mammary gland	Corn stover	Positive	activation of enzyme	7.19E-04	98
Mammary gland	Corn stover	Positive	morphology of bone	7.40E-04	112
Mammary gland	Corn stover	Positive	differentiation of embryonic tissue	7.40E-04	67
Mammary gland	Corn stover	Positive	modification of peptide	7.75E-04	32
Mammary gland	Corn stover	Positive	migration of breast cancer cell lines	7.76E-04	65
Mammary gland	Corn stover	Positive	phosphorylation of L-amino acid	7.76E-04	59
Mammary gland	Corn stover	Positive	cell movement of breast cancer cell lines	7.85E-04	75
Mammary gland	Corn stover	Positive	quantity of IL-6 in blood	7.93E-04	27
Mammary	Corn	Positive	abnormal morphology of right ventricle	7.94E-04	18

gland	stover				
Mammary gland	Corn stover	Positive	congenital malformation of face	8.36E-04	43
Mammary gland	Corn stover	Positive	quantity of subcutaneous fat	8.58E-04	21
Mammary gland	Corn stover	Positive	morphology of intercellular junctions	8.69E-04	29
Mammary gland	Corn stover	Positive	chemotaxis	8.98E-04	133
Mammary gland	Corn stover	Positive	mitosis	8.98E-04	122
Mammary gland	Corn stover	Positive	cell death of hepatoma cell lines	8.98E-04	47
Mammary gland	Corn stover	Positive	development of blood cells	9.14E-04	157
Mammary gland	Corn stover	Positive	abnormal morphology of gonad	9.19E-04	92
Mammary gland	Corn stover	Positive	neoplasia of cells	9.23E-04	110
Mammary gland	Corn stover	Positive	abnormal morphology of central nervous system	9.35E-04	119
Mammary gland	Corn stover	Positive	outgrowth of neurites	9.38E-04	101
Mammary gland	Corn stover	Positive	activation of cells	9.55E-04	228
Mammary gland	Corn stover	Positive	morphology of left ventricle	9.62E-04	27
Mammary gland	Corn stover	Positive	quantity of steroid	9.88E-04	115
Mammary gland	Corn stover	Positive	accumulation of cells	9.93E-04	89
Mammary gland	Corn stover	Positive	quantity of mononuclear leukocytes	1.00E-03	175
Mammary gland	Corn stover	Positive	apoptosis of fibroblasts	1.02E-03	58
Mammary gland	Corn stover	Positive	permeability of blood vessel	1.03E-03	29
Mammary gland	Corn stover	Positive	serine phosphorylation	1.03E-03	29
Mammary gland	Corn stover	Positive	cell death of pheochromocytoma cell lines	1.05E-03	35
Mammary gland	Corn stover	Positive	morphology of muscle	1.07E-03	85
Mammary	Corn	Positive	metabolism of hexose	1.07E-03	32

gland	stover				
Mammary gland	Corn stover	Positive	quantity of mitochondria	1.10E-03	17
Mammary gland	Corn stover	Positive	abnormal morphology of left ventricle	1.11E-03	26
Mammary gland	Corn stover	Positive	organization of filaments	1.11E-03	55
Mammary gland	Corn stover	Positive	abnormal morphology of brain	1.12E-03	110
Mammary gland	Corn stover	Positive	proliferation of embryonic cells	1.12E-03	65
Mammary gland	Corn stover	Positive	development of striated muscle	1.14E-03	49
Mammary gland	Corn stover	Positive	cell movement of endothelial cells	1.14E-03	89
Mammary gland	Corn stover	Positive	endocytosis	1.15E-03	87
Mammary gland	Corn stover	Positive	development of leukocytes	1.17E-03	142
Mammary gland	Corn stover	Positive	development of mononuclear leukocytes	1.21E-03	135
Mammary gland	Corn stover	Positive	differentiation of smooth muscle	1.22E-03	22
Mammary gland	Corn stover	Positive	abnormal morphology of skeleton	1.23E-03	70
Mammary gland	Corn stover	Positive	development of lymphocytes	1.23E-03	134
Mammary gland	Corn stover	Positive	abnormal morphology of skull	1.23E-03	58
Mammary gland	Corn stover	Positive	invasion of carcinoma cell lines	1.24E-03	53
Mammary gland	Corn stover	Positive	metabolism of sphingolipid	1.24E-03	39
Mammary gland	Corn stover	Positive	leukocyte migration	1.26E-03	204
Mammary gland	Corn stover	Positive	hypertrophy of cells	1.28E-03	74
Mammary gland	Corn stover	Positive	quantity of lymphocytes	1.29E-03	168
Mammary gland	Corn stover	Positive	vascularization of bone	1.30E-03	7
Mammary gland	Corn stover	Positive	quantity of cellular protrusions	1.30E-03	44
Mammary	Corn	Positive	fertility	1.30E-03	91

gland	stover				
Mammary gland	Corn stover	Positive	weight gain	1.31E-03	79
Mammary gland	Corn stover	Positive	congenital anomaly of gastrointestinal tract	1.32E-03	46
Mammary gland	Corn stover	Positive	function of mononuclear leukocytes	1.32E-03	86
Mammary gland	Corn stover	Positive	synthesis of ceramide	1.32E-03	23
Mammary gland	Corn stover	Positive	synthesis of nitric oxide	1.33E-03	71
Mammary gland	Corn stover	Positive	metabolism of glycosphingolipid	1.34E-03	36
Mammary gland	Corn stover	Positive	migration of fibroblasts	1.35E-03	40
Mammary gland	Corn stover	Positive	craniofacial abnormality	1.41E-03	89
Mammary gland	Corn stover	Positive	apoptosis of hepatoma cell lines	1.42E-03	42
Mammary gland	Corn stover	Positive	mass of organism	1.42E-03	84
Mammary gland	Corn stover	Positive	cell movement of fibrosarcoma cell lines	1.43E-03	17
Mammary gland	Corn stover	Positive	function of endothelial cells	1.43E-03	17
Mammary gland	Corn stover	Positive	damage of epithelial tissue	1.44E-03	31
Mammary gland	Corn stover	Positive	branching of neurites	1.44E-03	71
Mammary gland	Corn stover	Positive	apoptosis of neurons	1.46E-03	107
Mammary gland	Corn stover	Positive	branching of neurons	1.47E-03	73
Mammary gland	Corn stover	Positive	muscular hypertrophy	1.59E-03	54
Mammary gland	Corn stover	Positive	serine phosphorylation of peptide	1.59E-03	26
Mammary gland	Corn stover	Positive	abnormal morphology of epiblast	1.60E-03	10
Mammary gland	Corn stover	Positive	cell death of brain	1.62E-03	74
Mammary gland	Corn stover	Positive	stage 2-3 breast cancer	1.63E-03	23
Mammary	Corn	Positive	morphology of right ventricle	1.63E-03	19

gland	stover				
Mammary gland	Corn stover	Positive	size of animal	1.66E-03	48
Mammary gland	Corn stover	Positive	cellularity	1.68E-03	21
Mammary gland	Corn stover	Positive	transport of L-amino acid	1.68E-03	21
Mammary gland	Corn stover	Positive	apoptosis of embryonic cells	1.68E-03	29
Mammary gland	Corn stover	Positive	antiviral response of fibroblast cell lines	1.70E-03	8
Mammary gland	Corn stover	Positive	estrous cycle	1.74E-03	24
Mammary gland	Corn stover	Positive	concentration of phospholipid	1.74E-03	49
Mammary gland	Corn stover	Positive	morphology of vertebral column	1.74E-03	40
Mammary gland	Corn stover	Positive	function of lymphocytes	1.77E-03	85
Mammary gland	Corn stover	Positive	accumulation of sterol	1.86E-03	22
Mammary gland	Corn stover	Positive	morphology of skeleton	1.87E-03	71
Mammary gland	Corn stover	Positive	formation of pulmonary alveolus	1.88E-03	20
Mammary gland	Corn stover	Positive	morphology of limb	1.88E-03	60
Mammary gland	Corn stover	Positive	colony formation of cells	1.88E-03	119
Mammary gland	Corn stover	Positive	abnormal morphology of enlarged heart	1.89E-03	26
Mammary gland	Corn stover	Positive	morphology of fibroblast cell lines	1.89E-03	26
Mammary gland	Corn stover	Positive	growth of lymphatic system component	1.90E-03	51
Mammary gland	Corn stover	Positive	cell viability of lung cancer cell lines	1.90E-03	33
Mammary gland	Corn stover	Positive	metabolism of D-glucose	1.93E-03	27
Mammary gland	Corn stover	Positive	function of vascular endothelial cells	2.01E-03	14
Mammary gland	Corn stover	Positive	differentiation of embryonic cells	2.02E-03	59
Mammary	Corn	Positive	fatty acid metabolism	2.02E-03	143

gland	stover				
Mammary gland	Corn stover	Positive	shape change of neurites	2.02E-03	72
Mammary gland	Corn stover	Positive	proliferation of vascular smooth muscle cells	2.03E-03	42
Mammary gland	Corn stover	Positive	gonadal tumor	2.07E-03	191
Mammary gland	Corn stover	Positive	cell viability of muscle cells	2.08E-03	21
Mammary gland	Corn stover	Positive	release of L-amino acid	2.21E-03	25
Mammary gland	Corn stover	Positive	apoptosis of epithelial cells	2.21E-03	72
Mammary gland	Corn stover	Positive	cell death of embryonic cells	2.21E-03	33
Mammary gland	Corn stover	Positive	formation of T lymphocytes	2.21E-03	33
Mammary gland	Corn stover	Positive	epilepsy	2.24E-03	77
Mammary gland	Corn stover	Positive	migration of macrophages	2.31E-03	27
Mammary gland	Corn stover	Positive	congenital anomaly of mouth	2.33E-03	44
Mammary gland	Corn stover	Positive	glandular intraepithelial neoplasm	2.43E-03	35
Mammary gland	Corn stover	Positive	neoplasia of tumor cell lines	2.43E-03	79
Mammary gland	Corn stover	Positive	abnormal morphology of limb	2.44E-03	59
Mammary gland	Corn stover	Positive	survival of organism	2.44E-03	165
Mammary gland	Corn stover	Positive	quantity of reactive oxygen species	2.44E-03	47
Mammary gland	Corn stover	Positive	Lymphocyte homeostasis	2.56E-03	130
Mammary gland	Corn stover	Positive	Edema	2.56E-03	85
Mammary gland	Corn stover	Positive	degranulation of mast cells	2.57E-03	37
Mammary gland	Corn stover	Positive	long-term memory	2.60E-03	21
Mammary gland	Corn stover	Positive	bleeding of tissue	2.64E-03	19
Mammary	Corn	Positive	proliferation of blood cells	2.66E-03	195

gland	stover				
Mammary gland	Corn stover	Positive	mammary tumor	2.67E-03	364
Mammary gland	Corn stover	Positive	function of T lymphocytes	2.71E-03	66
Mammary gland	Corn stover	Positive	activation of microglia	2.72E-03	30
Mammary gland	Corn stover	Positive	homeostasis of leukocytes	2.72E-03	132
Mammary gland	Corn stover	Positive	metabolism of glycolipid	2.73E-03	41
Mammary gland	Corn stover	Positive	apoptosis of pheochromocytoma cell lines	2.73E-03	27
Mammary gland	Corn stover	Positive	release of amino acids	2.74E-03	28
Mammary gland	Corn stover	Positive	Thrombosis	2.74E-03	45
Mammary gland	Corn stover	Positive	formation of cells	2.76E-03	225
Mammary gland	Corn stover	Positive	TNM stage III breast cancer	2.77E-03	22
Mammary gland	Corn stover	Positive	synthesis of nucleotide	2.82E-03	89
Mammary gland	Corn stover	Positive	abnormal morphology of lung	2.82E-03	48
Mammary gland	Corn stover	Positive	invasion of tissue	2.85E-03	51
Mammary gland	Corn stover	Positive	size of cells	2.85E-03	104
Mammary gland	Corn stover	Positive	dyspnea	2.89E-03	37
Mammary gland	Corn stover	Positive	autosomal dominant Emery-Dreifuss muscular dystrophy	2.90E-03	20
Mammary gland	Corn stover	Positive	proliferation of neural stem cells	2.90E-03	20
Mammary gland	Corn stover	Positive	dysplasia	2.90E-03	55
Mammary gland	Corn stover	Positive	cell viability of carcinoma cell lines	2.99E-03	39
Mammary gland	Corn stover	Positive	neurotransmission	3.02E-03	100
Mammary gland	Corn stover	Positive	abnormal morphology of embryoblast	3.18E-03	17
Mammary	Corn	Positive	Ovarian Cancer and Tumors	3.18E-03	176

gland	stover				
Mammary gland	Corn stover	Positive	morphology of mitochondria	3.18E-03	29
Mammary gland	Corn stover	Positive	synthesis of glycosphingolipid	3.18E-03	29
Mammary gland	Corn stover	Positive	binding of DNA	3.18E-03	124
Mammary gland	Corn stover	Positive	synthesis of ATP	3.20E-03	27
Mammary gland	Corn stover	Positive	proliferation of endothelial cells	3.24E-03	77
Mammary gland	Corn stover	Positive	migration of mesenchymal stem cells	3.24E-03	11
Mammary gland	Corn stover	Positive	development of genitourinary system	3.25E-03	204
Mammary gland	Corn stover	Positive	abnormal morphology of vertebrae	3.27E-03	37
Mammary gland	Corn stover	Positive	morphology of pulmonary alveolus	3.36E-03	33
Mammary gland	Corn stover	Positive	cartilage development	3.37E-03	39
Mammary gland	Corn stover	Positive	morphology of lymphoid organ	3.44E-03	95
Mammary gland	Corn stover	Positive	transmembrane potential of mitochondria	3.52E-03	55
Mammary gland	Corn stover	Positive	migration of sarcoma cell lines	3.54E-03	23
Mammary gland	Corn stover	Positive	abnormal morphology of vertebral column	3.55E-03	38
Mammary gland	Corn stover	Positive	differentiation of tumor cell lines	3.57E-03	92
Mammary gland	Corn stover	Positive	proliferation of immune cells	3.59E-03	183
Mammary gland	Corn stover	Positive	shape change of kidney cell lines	3.60E-03	20
Mammary gland	Corn stover	Positive	function of blood vessel	3.65E-03	18
Mammary gland	Corn stover	Positive	neurogenesis of brain	3.65E-03	18
Mammary gland	Corn stover	Positive	concentration of acylglycerol	3.65E-03	82
Mammary gland	Corn stover	Positive	colony formation	3.69E-03	127
Mammary	Corn	Positive	morphology of uterus	3.75E-03	27

gland	stover				
Mammary gland	Corn stover	Positive	cell death of brain cells	3.75E-03	68
Mammary gland	Corn stover	Positive	export of molecule	3.78E-03	73
Mammary gland	Corn stover	Positive	development of sensory organ	3.78E-03	133
Mammary gland	Corn stover	Positive	outgrowth of cells	3.85E-03	104
Mammary gland	Corn stover	Positive	differentiation of bone cells	4.00E-03	98
Mammary gland	Corn stover	Positive	activation of astrocytes	4.06E-03	17
Mammary gland	Corn stover	Positive	transport of monosaccharide	4.06E-03	42
Mammary gland	Corn stover	Positive	cell death of cerebral cortex cells	4.06E-03	58
Mammary gland	Corn stover	Positive	fusion of cellular membrane	4.06E-03	19
Mammary gland	Corn stover	Positive	juvenile dermatomyositis	4.06E-03	19
Mammary gland	Corn stover	Positive	differentiation of osteoblasts	4.06E-03	69
Mammary gland	Corn stover	Positive	tumorigenesis of gonad	4.07E-03	186
Mammary gland	Corn stover	Positive	cell movement of leukocytes	4.18E-03	177
Mammary gland	Corn stover	Positive	Shock Response	4.18E-03	45
Mammary gland	Corn stover	Positive	transport of monovalent inorganic cation	4.19E-03	55
Mammary gland	Corn stover	Positive	localization of protein	4.22E-03	48
Mammary gland	Corn stover	Positive	activation of Protein kinase	4.28E-03	63
Mammary gland	Corn stover	Positive	death of perinatal stage organism	4.32E-03	24
Mammary gland	Corn stover	Positive	quantity of glycosphingolipid	4.33E-03	28
Mammary gland	Corn stover	Positive	oral squamous cell carcinoma	4.33E-03	46
Mammary gland	Corn stover	Positive	cancer of secretory structure	4.33E-03	430
Mammary	Corn	Positive	cell death of central nervous system cells	4.37E-03	72

gland	stover				
Mammary gland	Corn stover	Positive	fragmentation of mitochondria	4.37E-03	16
Mammary gland	Corn stover	Positive	metabolism of protein	4.46E-03	221
Mammary gland	Corn stover	Positive	cell movement of epithelial cell lines	4.53E-03	32
Mammary gland	Corn stover	Positive	migration of endothelial cells	4.54E-03	80
Mammary gland	Corn stover	Positive	metabolism of peptide	4.67E-03	48
Mammary gland	Corn stover	Positive	concentration of sterol	4.68E-03	75
Mammary gland	Corn stover	Positive	stress response of cells	4.72E-03	37
Mammary gland	Corn stover	Positive	growth of embryonic tissue	4.72E-03	68
Mammary gland	Corn stover	Positive	migration of fibrosarcoma cell lines	4.72E-03	15
Mammary gland	Corn stover	Positive	differentiation of leukocytes	4.74E-03	156
Mammary gland	Corn stover	Positive	myopathy of heart	4.74E-03	11
Mammary gland	Corn stover	Positive	familial cardiovascular disease	4.78E-03	46
Mammary gland	Corn stover	Positive	cell movement of embryonic cell lines	4.78E-03	30
Mammary gland	Corn stover	Positive	cell death of breast cancer cell lines	4.80E-03	81
Mammary gland	Corn stover	Positive	development of tumor cell lines	4.84E-03	22
Mammary gland	Corn stover	Positive	length of neurites	4.84E-03	22
Mammary gland	Corn stover	Negative	malignant solid tumor	9.26E-53	4810
Mammary gland	Corn stover	Negative	cancer	1.28E-52	4860
Mammary gland	Corn stover	Negative	proliferation of cells	2.26E-50	1888
Mammary gland	Corn stover	Negative	cell death	4.65E-50	1729
Mammary gland	Corn stover	Negative	apoptosis	8.51E-43	1388
Mammary	Corn	Negative	morbidity or mortality	4.85E-42	1287

gland	stover				
Mammary gland	Corn stover	Negative	organismal death	1.40E-41	1270
Mammary gland	Corn stover	Negative	necrosis	3.08E-38	1336
Mammary gland	Corn stover	Negative	neoplasia of epithelial tissue	5.38E-34	3986
Mammary gland	Corn stover	Negative	tumorigenesis of tissue	1.83E-33	4043
Mammary gland	Corn stover	Negative	organization of cytoplasm	3.00E-33	839
Mammary gland	Corn stover	Negative	morphology of cells	5.74E-33	1029
Mammary gland	Corn stover	Negative	abdominal neoplasm	9.09E-33	3951
Mammary gland	Corn stover	Negative	epithelial cancer	3.00E-32	3941
Mammary gland	Corn stover	Negative	abdominal cancer	3.59E-30	3894
Mammary gland	Corn stover	Negative	organization of cytoskeleton	2.78E-29	759
Mammary gland	Corn stover	Negative	cell movement	5.35E-29	1094
Mammary gland	Corn stover	Negative	migration of cells	7.90E-26	978
Mammary gland	Corn stover	Negative	expression of RNA	2.81E-24	1080
Mammary gland	Corn stover	Negative	transport of molecule	3.52E-24	838
Mammary gland	Corn stover	Negative	cell survival	3.98E-24	737
Mammary gland	Corn stover	Negative	cell death of tumor cell lines	7.46E-24	797
Mammary gland	Corn stover	Negative	cellular homeostasis	7.46E-24	795
Mammary gland	Corn stover	Negative	quantity of cells	3.84E-23	904
Mammary gland	Corn stover	Negative	Movement Disorders	1.24E-22	575
Mammary gland	Corn stover	Negative	transcription	2.22E-22	1002
Mammary gland	Corn stover	Negative	differentiation of cells	3.28E-22	1115
Mammary	Corn	Negative	apoptosis of tumor cell lines	3.85E-22	644

gland	stover				
Mammary gland	Corn stover	Negative	microtubule dynamics	4.26E-22	631
Mammary gland	Corn stover	Negative	cell viability	4.28E-22	685
Mammary gland	Corn stover	Negative	Viral Infection	4.28E-22	828
Mammary gland	Corn stover	Negative	transcription of RNA	2.28E-21	935
Mammary gland	Corn stover	Negative	proliferation of tumor cell lines	4.29E-21	794
Mammary gland	Corn stover	Negative	digestive organ tumor	5.31E-21	3375
Mammary gland	Corn stover	Negative	digestive system cancer	1.18E-20	3343
Mammary gland	Corn stover	Negative	disorder of basal ganglia	1.10E-18	420
Mammary gland	Corn stover	Negative	quantity of blood cells	3.15E-18	542
Mammary gland	Corn stover	Negative	angiogenesis	3.65E-18	485
Mammary gland	Corn stover	Negative	cell death of connective tissue cells	2.66E-17	335
Mammary gland	Corn stover	Negative	abnormal morphology of cells	2.82E-17	647
Mammary gland	Corn stover	Negative	concentration of lipid	4.58E-17	435
Mammary gland	Corn stover	Negative	neuromuscular disease	9.21E-17	468
Mammary gland	Corn stover	Negative	quantity of leukocytes	1.20E-16	483
Mammary gland	Corn stover	Negative	cell movement of tumor cell lines	1.66E-16	452
Mammary gland	Corn stover	Negative	vasculogenesis	1.86E-16	397
Mammary gland	Corn stover	Negative	Huntington's Disease	2.03E-16	318
Mammary gland	Corn stover	Negative	morphology of cardiovascular system	2.09E-16	340
Mammary gland	Corn stover	Negative	size of body	2.24E-16	451
Mammary gland	Corn stover	Negative	cell viability of tumor cell lines	3.39E-16	416
Mammary	Corn	Negative	function of blood cells	3.43E-16	323

gland	stover				
Mammary gland	Corn stover	Negative	formation of cellular protrusions	4.69E-16	474
Mammary gland	Corn stover	Negative	dyskinesia	5.62E-16	337
Mammary gland	Corn stover	Negative	synthesis of lipid	1.16E-15	383
Mammary gland	Corn stover	Negative	neurological signs	1.65E-15	352
Mammary gland	Corn stover	Negative	morphology of body cavity	1.68E-15	606
Mammary gland	Corn stover	Negative	function of leukocytes	2.08E-15	296
Mammary gland	Corn stover	Negative	cell cycle progression	2.17E-15	538
Mammary gland	Corn stover	Negative	abnormal morphology of body cavity	4.24E-15	580
Mammary gland	Corn stover	Negative	development of cytoplasm	7.87E-15	257
Mammary gland	Corn stover	Negative	growth of organism	9.71E-15	437
Mammary gland	Corn stover	Negative	cell movement of blood cells	2.01E-14	454
Mammary gland	Corn stover	Negative	phosphorylation of protein	2.02E-14	388
Mammary gland	Corn stover	Negative	migration of blood cells	3.36E-14	452
Mammary gland	Corn stover	Negative	infection by RNA virus	3.41E-14	449
Mammary gland	Corn stover	Negative	leukocyte migration	3.92E-14	451
Mammary gland	Corn stover	Negative	concentration of fatty acid	5.50E-14	163
Mammary gland	Corn stover	Negative	cell movement of leukocytes	5.64E-14	401
Mammary gland	Corn stover	Negative	transcription of DNA	1.04E-13	749
Mammary gland	Corn stover	Negative	invasion of cells	1.07E-13	448
Mammary gland	Corn stover	Negative	migration of tumor cell lines	1.26E-13	371
Mammary gland	Corn stover	Negative	cell spreading	2.06E-13	160
Mammary	Corn	Negative	growth of connective tissue	2.59E-13	340

gland	stover				
Mammary gland	Corn stover	Negative	cell death of blood cells	3.18E-13	363
Mammary gland	Corn stover	Negative	necrosis of epithelial tissue	3.19E-13	313
Mammary gland	Corn stover	Negative	proliferation of connective tissue cells	3.20E-13	315
Mammary gland	Corn stover	Negative	quantity of lymphocytes	4.41E-13	369
Mammary gland	Corn stover	Negative	growth of epithelial tissue	4.46E-13	356
Mammary gland	Corn stover	Negative	transactivation	5.38E-13	307
Mammary gland	Corn stover	Negative	infection of cells	9.43E-13	410
Mammary gland	Corn stover	Negative	protein kinase cascade	9.53E-13	230
Mammary gland	Corn stover	Negative	infection by Retroviridae	1.03E-12	373
Mammary gland	Corn stover	Negative	degeneration of nervous system	1.33E-12	163
Mammary gland	Corn stover	Negative	cell death of immune cells	1.60E-12	344
Mammary gland	Corn stover	Negative	abnormal morphology of cardiovascular system	1.65E-12	300
Mammary gland	Corn stover	Negative	autosomal recessive disease	1.79E-12	466
Mammary gland	Corn stover	Negative	proliferation of blood cells	2.25E-12	429
Mammary gland	Corn stover	Negative	quantity of mononuclear leukocytes	2.28E-12	379
Mammary gland	Corn stover	Negative	Neurodegeneration	3.00E-12	173
Mammary gland	Corn stover	Negative	formation of cytoskeleton	3.16E-12	206
Mammary gland	Corn stover	Negative	abnormal morphology of thoracic cavity	5.52E-12	307
Mammary gland	Corn stover	Negative	homing of cells	6.17E-12	293
Mammary gland	Corn stover	Negative	infection by lentivirus	6.81E-12	367
Mammary gland	Corn stover	Negative	HIV infection	6.81E-12	366
Mammary	Corn	Negative	organization of organelle	6.81E-12	284

gland	stover				
Mammary gland	Corn stover	Negative	degeneration of cells	8.17E-12	189
Mammary gland	Corn stover	Negative	abnormal morphology of epithelial tissue	1.04E-11	225
Mammary gland	Corn stover	Negative	cell death of fibroblast cell lines	1.16E-11	229
Mammary gland	Corn stover	Negative	cell transformation	1.35E-11	245
Mammary gland	Corn stover	Negative	fatty acid metabolism	1.36E-11	312
Mammary gland	Corn stover	Negative	homing	1.60E-11	299
Mammary gland	Corn stover	Negative	cell death of epithelial cells	1.76E-11	262
Mammary gland	Corn stover	Negative	proliferation of immune cells	1.99E-11	401
Mammary gland	Corn stover	Negative	abnormal morphology of abdomen	2.04E-11	440
Mammary gland	Corn stover	Negative	Organ Degeneration	2.07E-11	260
Mammary gland	Corn stover	Negative	cell movement of myeloid cells	2.55E-11	282
Mammary gland	Corn stover	Negative	cellular degradation	2.69E-11	143
Mammary gland	Corn stover	Negative	formation of muscle	2.75E-11	213
Mammary gland	Corn stover	Negative	ubiquitination of protein	3.20E-11	181
Mammary gland	Corn stover	Negative	apoptosis of blood cells	3.76E-11	260
Mammary gland	Corn stover	Negative	migration of muscle cells	4.13E-11	98
Mammary gland	Corn stover	Negative	function of cardiovascular system	4.31E-11	199
Mammary gland	Corn stover	Negative	ubiquitination	4.52E-11	183
Mammary gland	Corn stover	Negative	neuronal cell death	6.44E-11	347
Mammary gland	Corn stover	Negative	transactivation of RNA	6.45E-11	282
Mammary gland	Corn stover	Negative	development of connective tissue	6.92E-11	199
Mammary	Corn	Negative	development of epithelial tissue	7.21E-11	269

gland	stover				
Mammary gland	Corn stover	Negative	chemotaxis of cells	7.39E-11	274
Mammary gland	Corn stover	Negative	invasion of tumor cell lines	7.85E-11	339
Mammary gland	Corn stover	Negative	function of phagocytes	9.05E-11	167
Mammary gland	Corn stover	Negative	fibrogenesis	9.47E-11	218
Mammary gland	Corn stover	Negative	morphology of heart	1.10E-10	215
Mammary gland	Corn stover	Negative	inflammatory response	1.13E-10	391
Mammary gland	Corn stover	Negative	development of body trunk	1.27E-10	544
Mammary gland	Corn stover	Negative	formation of cells	1.33E-10	484
Mammary gland	Corn stover	Negative	mitosis	1.40E-10	258
Mammary gland	Corn stover	Negative	chemotaxis	1.56E-10	281
Mammary gland	Corn stover	Negative	differentiation of connective tissue cells	1.61E-10	327
Mammary gland	Corn stover	Negative	engulfment of cells	1.66E-10	215
Mammary gland	Corn stover	Negative	quantity of connective tissue	1.76E-10	308
Mammary gland	Corn stover	Negative	proliferation of lymphocytes	1.76E-10	366
Mammary gland	Corn stover	Negative	proliferation of mononuclear leukocytes	1.92E-10	371
Mammary gland	Corn stover	Negative	infection by HIV-1	2.04E-10	311
Mammary gland	Corn stover	Negative	quantity of B lymphocytes	2.04E-10	185
Mammary gland	Corn stover	Negative	growth of lymphatic system component	2.05E-10	110
Mammary gland	Corn stover	Negative	growth of embryo	2.20E-10	248
Mammary gland	Corn stover	Negative	accumulation of lipid	2.40E-10	162
Mammary gland	Corn stover	Negative	cell movement of muscle cells	2.47E-10	104
Mammary	Corn	Negative	growth of muscle tissue	2.47E-10	198

gland	stover				
Mammary gland	Corn stover	Negative	cell movement of phagocytes	2.52E-10	282
Mammary gland	Corn stover	Negative	proliferation of fibroblasts	2.82E-10	188
Mammary gland	Corn stover	Negative	apoptosis of fibroblast cell lines	2.83E-10	176
Mammary gland	Corn stover	Negative	apoptosis of leukocytes	2.85E-10	238
Mammary gland	Corn stover	Negative	differentiation of connective tissue	3.06E-10	366
Mammary gland	Corn stover	Negative	activation of cells	3.84E-10	479
Mammary gland	Corn stover	Negative	colony formation of cells	4.00E-10	254
Mammary gland	Corn stover	Negative	formation of filaments	4.02E-10	209
Mammary gland	Corn stover	Negative	morphology of connective tissue	4.26E-10	239
Mammary gland	Corn stover	Negative	function of antigen presenting cells	4.40E-10	135
Mammary gland	Corn stover	Negative	proliferation of muscle cells	4.45E-10	196
Mammary gland	Corn stover	Negative	degeneration of neurons	4.51E-10	132
Mammary gland	Corn stover	Negative	synthesis of reactive oxygen species	4.51E-10	248
Mammary gland	Corn stover	Negative	cell death of cervical cancer cell lines	5.12E-10	186
Mammary gland	Corn stover	Negative	development of blood cells	5.25E-10	329
Mammary gland	Corn stover	Negative	migration of smooth muscle cells	5.50E-10	87
Mammary gland	Corn stover	Negative	replication of virus	5.78E-10	286
Mammary gland	Corn stover	Negative	formation of actin stress fibers	6.00E-10	132
Mammary gland	Corn stover	Negative	function of myeloid cells	7.49E-10	131
Mammary gland	Corn stover	Negative	growth of lesion	8.25E-10	395
Mammary gland	Corn stover	Negative	seizure disorder	9.28E-10	242
Mammary	Corn	Negative	growth of tumor	9.63E-10	394

gland	stover				
Mammary gland	Corn stover	Negative	replication of RNA virus	9.92E-10	259
Mammary gland	Corn stover	Negative	small GTPase mediated signal transduction	9.92E-10	104
Mammary gland	Corn stover	Negative	cellular infiltration of blood cells	9.92E-10	203
Mammary gland	Corn stover	Negative	uptake of carbohydrate	9.94E-10	156
Mammary gland	Corn stover	Negative	morphology of nervous system	1.04E-09	388
Mammary gland	Corn stover	Negative	colony formation	1.17E-09	273
Mammary gland	Corn stover	Negative	Growth Failure	1.21E-09	302
Mammary gland	Corn stover	Negative	demyelination	1.24E-09	72
Mammary gland	Corn stover	Negative	development of vasculature	1.25E-09	217
Mammary gland	Corn stover	Negative	function of macrophages	1.29E-09	101
Mammary gland	Corn stover	Negative	morphology of digestive system	1.29E-09	289
Mammary gland	Corn stover	Negative	cellular infiltration	1.38E-09	226
Mammary gland	Corn stover	Negative	formation of skin	1.43E-09	192
Mammary gland	Corn stover	Negative	cellular infiltration by leukocytes	1.44E-09	202
Mammary gland	Corn stover	Negative	synthesis of DNA	1.47E-09	216
Mammary gland	Corn stover	Negative	uptake of monosaccharide	1.47E-09	145
Mammary gland	Corn stover	Negative	abnormal morphology of digestive system	1.58E-09	276
Mammary gland	Corn stover	Negative	interphase	1.73E-09	328
Mammary gland	Corn stover	Negative	development of cardiovascular tissue	1.79E-09	193
Mammary gland	Corn stover	Negative	cellular infiltration of cells	1.88E-09	206
Mammary gland	Corn stover	Negative	cell movement of smooth muscle cells	1.96E-09	92
Mammary	Corn	Negative	differentiation of epithelial tissue	1.96E-09	188

gland	stover				
Mammary gland	Corn stover	Negative	cell movement of endothelial cells	2.27E-09	185
Mammary gland	Corn stover	Negative	metabolism of membrane lipid derivative	2.62E-09	201
Mammary gland	Corn stover	Negative	activation of DNA endogenous promoter	2.62E-09	577
Mammary gland	Corn stover	Negative	quantity of lymphatic system component	2.93E-09	196
Mammary gland	Corn stover	Negative	seizures	3.51E-09	206
Mammary gland	Corn stover	Negative	quantity of T lymphocytes	3.78E-09	270
Mammary gland	Corn stover	Negative	development of leukocytes	3.81E-09	295
Mammary gland	Corn stover	Negative	endothelial cell development	4.31E-09	184
Mammary gland	Corn stover	Negative	autophagy	4.31E-09	194
Mammary gland	Corn stover	Negative	metabolism of reactive oxygen species	4.64E-09	253
Mammary gland	Corn stover	Negative	genital tumor	4.70E-09	1803
Mammary gland	Corn stover	Negative	secretion of molecule	5.06E-09	277
Mammary gland	Corn stover	Negative	adenocarcinoma	5.14E-09	2845
Mammary gland	Corn stover	Negative	dysmyelination	5.14E-09	78
Mammary gland	Corn stover	Negative	recruitment of cells	5.39E-09	185
Mammary gland	Corn stover	Negative	benign neoplasia	6.24E-09	448
Mammary gland	Corn stover	Negative	proliferation of epithelial cells	6.54E-09	249
Mammary gland	Corn stover	Negative	metabolism of protein	7.14E-09	469
Mammary gland	Corn stover	Negative	hypersensitive reaction	7.14E-09	219
Mammary gland	Corn stover	Negative	development of endothelial tissue	7.23E-09	189
Mammary gland	Corn stover	Negative	development of mononuclear leukocytes	8.07E-09	279
Mammary	Corn	Negative	development of lymphocytes	8.15E-09	277

Mammary gland	Corn stover				
Mammary gland	Corn stover	Negative	abnormal morphology of heart	8.25E-09	197
Mammary gland	Corn stover	Negative	cell death of epithelial cell lines	8.28E-09	149
Mammary gland	Corn stover	Negative	hepatocellular carcinoma	8.50E-09	1862
Mammary gland	Corn stover	Negative	survival of organism	8.85E-09	346
Mammary gland	Corn stover	Negative	liver tumor	9.71E-09	1932
Mammary gland	Corn stover	Negative	concentration of sterol	1.00E-08	160
Mammary gland	Corn stover	Negative	proliferation of T lymphocytes	1.05E-08	300
Mammary gland	Corn stover	Negative	Hypertrophy	1.09E-08	239
Mammary gland	Corn stover	Negative	proliferation of smooth muscle cells	1.09E-08	150
Mammary gland	Corn stover	Negative	formation of actin filaments	1.22E-08	159
Mammary gland	Corn stover	Negative	binding of DNA	1.22E-08	261
Mammary gland	Corn stover	Negative	abnormal morphology of nervous system	1.32E-08	355
Mammary gland	Corn stover	Negative	epileptic seizure	1.36E-08	93
Mammary gland	Corn stover	Negative	metabolism of carbohydrate	1.52E-08	307
Mammary gland	Corn stover	Negative	cell movement of mononuclear leukocytes	1.53E-08	233
Mammary gland	Corn stover	Negative	synthesis of fatty acid	1.68E-08	162
Mammary gland	Corn stover	Negative	neurodegeneration of neurites	1.76E-08	55
Mammary gland	Corn stover	Negative	hepatobiliary system cancer	1.76E-08	1925
Mammary gland	Corn stover	Negative	differentiation of muscle cell lines	1.81E-08	90
Mammary gland	Corn stover	Negative	quantity of antigen presenting cells	1.81E-08	145
Mammary gland	Corn stover	Negative	peripheral arterial disease	1.81E-08	114
Mammary	Corn	Negative	cell death of mononuclear leukocytes	1.87E-08	202

gland	stover				
Mammary gland	Corn stover	Negative	arthritis	1.87E-08	450
Mammary gland	Corn stover	Negative	liver cancer	2.00E-08	1916
Mammary gland	Corn stover	Negative	apoptosis of connective tissue cells	2.00E-08	154
Mammary gland	Corn stover	Negative	arthropathy	2.00E-08	457
Mammary gland	Corn stover	Negative	cell death of central nervous system cells	2.37E-08	152
Mammary gland	Corn stover	Negative	endocytosis	2.38E-08	177
Mammary gland	Corn stover	Negative	morphology of head	2.40E-08	422
Mammary gland	Corn stover	Negative	metabolism of nucleotide	2.50E-08	221
Mammary gland	Corn stover	Negative	homeostasis of leukocytes	2.83E-08	275
Mammary gland	Corn stover	Negative	autosomal dominant disease	2.83E-08	313
Mammary gland	Corn stover	Negative	concentration of cholesterol	2.86E-08	151
Mammary gland	Corn stover	Negative	neuritogenesis	2.86E-08	288
Mammary gland	Corn stover	Negative	recruitment of phagocytes	3.00E-08	130
Mammary gland	Corn stover	Negative	transport of protein	3.37E-08	156
Mammary gland	Corn stover	Negative	Lymphocyte homeostasis	3.41E-08	270
Mammary gland	Corn stover	Negative	apoptosis of epithelial cell lines	3.41E-08	119
Mammary gland	Corn stover	Negative	cell death of brain	3.58E-08	151
Mammary gland	Corn stover	Negative	phosphorylation of L-amino acid	3.93E-08	117
Mammary gland	Corn stover	Negative	T cell development	3.93E-08	250
Mammary gland	Corn stover	Negative	paraproteinemia	3.99E-08	172
Mammary gland	Corn stover	Negative	proliferation of neuronal cells	4.00E-08	284
Mammary	Corn	Negative	production of reactive oxygen species	4.05E-08	186

gland	stover				
Mammary gland	Corn stover	Negative	development of body axis	4.35E-08	499
Mammary gland	Corn stover	Negative	cell death of lymphocytes	4.47E-08	195
Mammary gland	Corn stover	Negative	migration of endothelial cells	4.49E-08	168
Mammary gland	Corn stover	Negative	quantity of interleukin	5.27E-08	78
Mammary gland	Corn stover	Negative	function of muscle	5.33E-08	177
Mammary gland	Corn stover	Negative	development of head	5.40E-08	467
Mammary gland	Corn stover	Negative	activation of enzyme	5.43E-08	196
Mammary gland	Corn stover	Negative	size of embryo	5.62E-08	181
Mammary gland	Corn stover	Negative	plasma cell dyscrasia	5.87E-08	171
Mammary gland	Corn stover	Negative	movement of vascular endothelial cells	6.20E-08	98
Mammary gland	Corn stover	Negative	recruitment of blood cells	6.86E-08	168
Mammary gland	Corn stover	Negative	quantity of steroid	6.89E-08	232
Mammary gland	Corn stover	Negative	differentiation of bone	6.89E-08	206
Mammary gland	Corn stover	Negative	T cell homeostasis	7.09E-08	255
Mammary gland	Corn stover	Negative	immune response of cells	7.44E-08	281
Mammary gland	Corn stover	Negative	cell death of kidney cells	7.90E-08	177
Mammary gland	Corn stover	Negative	differentiation of bone cells	8.19E-08	204
Mammary gland	Corn stover	Negative	metabolism of nucleic acid component or derivative	8.21E-08	256
Mammary gland	Corn stover	Negative	cognition	8.22E-08	226
Mammary gland	Corn stover	Negative	Bleeding	8.27E-08	212
Mammary gland	Corn stover	Negative	morphology of cardiovascular tissue	8.36E-08	52
Mammary	Corn	Negative	necrosis of kidney	8.48E-08	184

gland	stover				
Mammary gland	Corn stover	Negative	differentiation of epithelial cells	8.64E-08	160
Mammary gland	Corn stover	Negative	blood protein disorder	8.86E-08	189
Mammary gland	Corn stover	Negative	apoptosis of cervical cancer cell lines	8.89E-08	145
Mammary gland	Corn stover	Negative	growth of lymphoid organ	9.30E-08	93
Mammary gland	Corn stover	Negative	recruitment of leukocytes	9.52E-08	165
Mammary gland	Corn stover	Negative	abnormal morphology of embryonic tissue	9.63E-08	269
Mammary gland	Corn stover	Negative	morphology of lymphatic system component	9.63E-08	208
Mammary gland	Corn stover	Negative	infection of tumor cell lines	9.64E-08	252
Mammary gland	Corn stover	Negative	neurodegeneration of axons	9.83E-08	51
Mammary gland	Corn stover	Negative	recruitment of macrophages	9.90E-08	61
Mammary gland	Corn stover	Negative	cell death of liver cells	1.02E-07	104
Mammary gland	Corn stover	Negative	uptake of D-glucose	1.03E-07	116
Mammary gland	Corn stover	Negative	peripheral vascular disease	1.06E-07	191
Mammary gland	Corn stover	Negative	morphology of endothelial cells	1.06E-07	39
Mammary gland	Corn stover	Negative	quantity of carbohydrate	1.08E-07	255
Mammary gland	Corn stover	Negative	uptake of D-hexose	1.08E-07	117
Mammary gland	Corn stover	Negative	quantity of reactive oxygen species	1.11E-07	95
Mammary gland	Corn stover	Negative	formation of plasma membrane projections	1.11E-07	291
Mammary gland	Corn stover	Negative	perinatal death	1.12E-07	290
Mammary gland	Corn stover	Negative	behavior	1.18E-07	468
Mammary gland	Corn stover	Negative	abnormal morphology of head	1.21E-07	398
Mammary	Corn	Negative	recruitment of antigen presenting cells	1.26E-07	68

gland	stover				
Mammary gland	Corn stover	Negative	quantity of phagocytes	1.34E-07	211
Mammary gland	Corn stover	Negative	differentiation of muscle	1.37E-07	154
Mammary gland	Corn stover	Negative	cell death of brain cells	1.45E-07	140
Mammary gland	Corn stover	Negative	proliferation of endothelial cells	1.50E-07	158
Mammary gland	Corn stover	Negative	autophagy of cells	1.59E-07	136
Mammary gland	Corn stover	Negative	quantity of metal	1.59E-07	245
Mammary gland	Corn stover	Negative	activation of Protein kinase	1.59E-07	130
Mammary gland	Corn stover	Negative	I-kappaB kinase/NF-kappaB cascade	1.67E-07	93
Mammary gland	Corn stover	Negative	abdominal adenocarcinoma	1.67E-07	2575
Mammary gland	Corn stover	Negative	quantity of protein in blood	1.79E-07	249
Mammary gland	Corn stover	Negative	proliferation of fibroblast cell lines	1.88E-07	210
Mammary gland	Corn stover	Negative	cell death of cerebral cortex cells	2.01E-07	119
Mammary gland	Corn stover	Negative	apoptosis of mononuclear leukocytes	2.02E-07	179
Mammary gland	Corn stover	Negative	permeability of cells	2.17E-07	57
Mammary gland	Corn stover	Negative	female genital tract serous cancer	2.19E-07	192
Mammary gland	Corn stover	Negative	differentiation of leukocytes	2.20E-07	324
Mammary gland	Corn stover	Negative	activation of leukocytes	2.28E-07	336
Mammary gland	Corn stover	Negative	vascularization	2.31E-07	122
Mammary gland	Corn stover	Negative	synthesis of nitric oxide	2.31E-07	141
Mammary gland	Corn stover	Negative	apoptosis of muscle	2.31E-07	134
Mammary gland	Corn stover	Negative	morphology of endothelial tissue	2.31E-07	49
Mammary	Corn	Negative	cell death of tumor	2.58E-07	190

gland	stover				
Mammary gland	Corn stover	Negative	concentration of acylglycerol	2.62E-07	168
Mammary gland	Corn stover	Negative	internalization by tumor cell lines	2.83E-07	66
Mammary gland	Corn stover	Negative	apoptosis of muscle cells	2.83E-07	133
Mammary gland	Corn stover	Negative	synthesis of nucleotide	2.96E-07	181
Mammary gland	Corn stover	Negative	apoptosis of pheochromocytoma cell lines	2.96E-07	53
Mammary gland	Corn stover	Negative	quantity of metal ion	3.00E-07	218
Mammary gland	Corn stover	Negative	quantity of cytokine	3.20E-07	130
Mammary gland	Corn stover	Negative	quantity of lymphoid organ	3.20E-07	155
Mammary gland	Corn stover	Negative	quantity of IL-6 in blood	3.20E-07	50
Mammary gland	Corn stover	Negative	cell death of muscle	3.29E-07	172
Mammary gland	Corn stover	Negative	apoptosis of lymphocytes	3.32E-07	173
Mammary gland	Corn stover	Negative	apoptosis of liver	3.48E-07	85
Mammary gland	Corn stover	Negative	S phase	3.52E-07	133
Mammary gland	Corn stover	Negative	cell movement of granulocytes	3.57E-07	184
Mammary gland	Corn stover	Negative	learning	3.65E-07	206
Mammary gland	Corn stover	Negative	allergy	3.66E-07	200
Mammary gland	Corn stover	Negative	Waldenstrom's macroglobulinemia	3.66E-07	99
Mammary gland	Corn stover	Negative	function of lymphocytes	3.88E-07	170
Mammary gland	Corn stover	Negative	necrosis of muscle	3.90E-07	171
Mammary gland	Corn stover	Negative	cell death of T lymphocytes	4.06E-07	158
Mammary gland	Corn stover	Negative	breast or colorectal cancer	4.11E-07	2173
Mammary	Corn	Negative	apoptosis of cardiomyocytes	4.16E-07	97

gland	stover				
Mammary gland	Corn stover	Negative	quantity of macrophages	4.18E-07	105
Mammary gland	Corn stover	Negative	occlusion of blood vessel	4.22E-07	235
Mammary gland	Corn stover	Negative	vaso-occlusion	4.25E-07	234
Mammary gland	Corn stover	Negative	apoptosis of epithelial cells	4.69E-07	144
Mammary gland	Corn stover	Negative	development of lymphatic system component	4.70E-07	155
Mammary gland	Corn stover	Negative	cell death of heart	4.77E-07	117
Mammary gland	Corn stover	Negative	quantity of hematopoietic cells	4.78E-07	217
Mammary gland	Corn stover	Negative	apoptosis of heart cells	4.78E-07	99
Mammary gland	Corn stover	Negative	development of lymphatic system	4.85E-07	177
Mammary gland	Corn stover	Negative	abnormal morphology of lymphoid organ	5.02E-07	187
Mammary gland	Corn stover	Negative	cell movement of fibroblasts	5.02E-07	96
Mammary gland	Corn stover	Negative	concentration of phospholipid	5.02E-07	96
Mammary gland	Corn stover	Negative	necrosis of tumor	5.02E-07	188
Mammary gland	Corn stover	Negative	transformation of fibroblast cell lines	5.02E-07	131
Mammary gland	Corn stover	Negative	cell death of muscle cells	5.38E-07	167
Mammary gland	Corn stover	Negative	differentiation of muscle cells	5.44E-07	142
Mammary gland	Corn stover	Negative	quantity of hematopoietic progenitor cells	5.57E-07	216
Mammary gland	Corn stover	Negative	binding of protein binding site	5.81E-07	145
Mammary gland	Corn stover	Negative	morphology of lymphoid organ	5.99E-07	193
Mammary gland	Corn stover	Negative	Cytosis	6.03E-07	160
Mammary gland	Corn stover	Negative	growth of skin	6.05E-07	112
Mammary	Corn	Negative	congenital anomaly of musculoskeletal system	6.32E-07	350

gland	stover				
Mammary gland	Corn stover	Negative	cell death of heart cells	6.35E-07	113
Mammary gland	Corn stover	Negative	activation of blood cells	6.38E-07	355
Mammary gland	Corn stover	Negative	migration of breast cancer cell lines	6.43E-07	125
Mammary gland	Corn stover	Negative	tumorigenesis of genital organ	6.50E-07	1723
Mammary gland	Corn stover	Negative	occlusion of artery	6.73E-07	231
Mammary gland	Corn stover	Negative	advanced malignant tumor	6.74E-07	399
Mammary gland	Corn stover	Negative	differentiation of blood cells	6.89E-07	398
Mammary gland	Corn stover	Negative	apoptosis of liver cells	6.91E-07	83
Mammary gland	Corn stover	Negative	cell movement of lymphocytes	6.98E-07	194
Mammary gland	Corn stover	Negative	generation of blood cells	7.49E-07	87
Mammary gland	Corn stover	Negative	apoptosis of myeloid cells	7.53E-07	98
Mammary gland	Corn stover	Negative	Dermatitis	7.73E-07	212
Mammary gland	Corn stover	Negative	cell viability of cervical cancer cell lines	8.05E-07	113
Mammary gland	Corn stover	Negative	proliferation of embryonic cells	8.31E-07	126
Mammary gland	Corn stover	Negative	cell movement of macrophages	8.38E-07	134
Mammary gland	Corn stover	Negative	genital tract cancer	8.82E-07	1707
Mammary gland	Corn stover	Negative	colony formation of tumor cell lines	9.26E-07	138
Mammary gland	Corn stover	Negative	hypertrophy of heart	9.29E-07	167
Mammary gland	Corn stover	Negative	proliferation of tumor cells	9.31E-07	228
Mammary gland	Corn stover	Negative	cell death of kidney cell lines	9.50E-07	151
Mammary gland	Corn stover	Negative	differentiation of skin	9.83E-07	94
Mammary	Corn	Negative	migration of connective tissue cells	9.83E-07	94

gland	stover				
Mammary gland	Corn stover	Negative	female genital neoplasm	1.00E-06	1568
Mammary gland	Corn stover	Negative	cell death of fibroblasts	1.01E-06	133
Mammary gland	Corn stover	Negative	cell movement of neutrophils	1.11E-06	148
Mammary gland	Corn stover	Negative	proliferation of dermal cells	1.13E-06	92
Mammary gland	Corn stover	Negative	generation of leukocytes	1.13E-06	85
Mammary gland	Corn stover	Negative	cell movement of antigen presenting cells	1.14E-06	172
Mammary gland	Corn stover	Negative	metastasis	1.15E-06	359
Mammary gland	Corn stover	Negative	morphology of intestine	1.15E-06	116
Mammary gland	Corn stover	Negative	necrosis of cardiac muscle	1.19E-06	111
Mammary gland	Corn stover	Negative	hypertrophy of cells	1.27E-06	144
Mammary gland	Corn stover	Negative	atherosclerosis	1.30E-06	224
Mammary gland	Corn stover	Negative	morphology of reproductive system	1.34E-06	257
Mammary gland	Corn stover	Negative	proliferation of hematopoietic cells	1.36E-06	128
Mammary gland	Corn stover	Negative	abnormal morphology of reproductive system	1.37E-06	245
Mammary gland	Corn stover	Negative	cell death of cardiomyocytes	1.38E-06	109
Mammary gland	Corn stover	Negative	growth of thymus gland	1.39E-06	54
Mammary gland	Corn stover	Negative	abnormal morphology of respiratory system	1.41E-06	152
Mammary gland	Corn stover	Negative	infection of cervical cancer cell lines	1.42E-06	213
Mammary gland	Corn stover	Negative	atrophy of muscle	1.42E-06	78
Mammary gland	Corn stover	Negative	morphology of respiratory system	1.43E-06	154
Mammary gland	Corn stover	Negative	Thrombosis	1.43E-06	88
Mammary	Corn	Negative	proliferation of epidermal cells	1.43E-06	88

gland	stover				
Mammary gland	Corn stover	Negative	uterine serous papillary cancer	1.49E-06	117
Mammary gland	Corn stover	Negative	adhesion of tumor cell lines	1.50E-06	143
Mammary gland	Corn stover	Negative	cell viability of carcinoma cell lines	1.57E-06	76
Mammary gland	Corn stover	Negative	organization of actin cytoskeleton	1.64E-06	149
Mammary gland	Corn stover	Negative	cellular infiltration by macrophages	1.69E-06	83
Mammary gland	Corn stover	Negative	growth of plasma membrane projections	1.71E-06	225
Mammary gland	Corn stover	Negative	cell death of neuroblastoma cell lines	1.74E-06	99
Mammary gland	Corn stover	Negative	skin abnormality	1.74E-06	115
Mammary gland	Corn stover	Negative	arrest in interphase	1.77E-06	202
Mammary gland	Corn stover	Negative	quantity of Ca2+	1.81E-06	198
Mammary gland	Corn stover	Negative	urogenital cancer	1.87E-06	1947
Mammary gland	Corn stover	Negative	sprouting	1.88E-06	188
Mammary gland	Corn stover	Negative	consumption of oxygen	1.88E-06	81
Mammary gland	Corn stover	Negative	serous neoplasm	1.93E-06	290
Mammary gland	Corn stover	Negative	differentiation of keratinocytes	2.13E-06	79
Mammary gland	Corn stover	Negative	differentiation of dermal cells	2.16E-06	86
Mammary gland	Corn stover	Negative	cell movement of breast cancer cell lines	2.23E-06	143
Mammary gland	Corn stover	Negative	cell death of tumor cells	2.27E-06	181
Mammary gland	Corn stover	Negative	expansion of cells	2.41E-06	149
Mammary gland	Corn stover	Negative	formation of lamellipodia	2.46E-06	84
Mammary gland	Corn stover	Negative	cell movement of connective tissue cells	2.49E-06	112
Mammary	Corn	Negative	cell death of neuroglia	2.61E-06	64

gland	stover				
Mammary gland	Corn stover	Negative	cell death of breast cancer cell lines	2.65E-06	163
Mammary gland	Corn stover	Negative	differentiation of epidermal cells	2.65E-06	85
Mammary gland	Corn stover	Negative	apoptosis of neurons	2.65E-06	210
Mammary gland	Corn stover	Negative	breast or ovarian cancer	2.73E-06	859
Mammary gland	Corn stover	Negative	growth of neurites	2.73E-06	222
Mammary gland	Corn stover	Negative	synthesis of carbohydrate	2.76E-06	205
Mammary gland	Corn stover	Negative	intermediate disease stage peripheral arterial disease	2.76E-06	82
Mammary gland	Corn stover	Negative	migration of vascular endothelial cells	2.85E-06	86
Mammary gland	Corn stover	Negative	experimentally-induced arthritis	2.85E-06	79
Mammary gland	Corn stover	Negative	activation of neuroglia	2.86E-06	70
Mammary gland	Corn stover	Negative	Rheumatic Disease	2.92E-06	497
Mammary gland	Corn stover	Negative	quantity of lymphatic system cells	2.95E-06	136
Mammary gland	Corn stover	Negative	chronic inflammatory disorder	2.97E-06	451
Mammary gland	Corn stover	Negative	gastrointestinal tract cancer	3.00E-06	2199
Mammary gland	Corn stover	Negative	arteriosclerosis	3.01E-06	225
Mammary gland	Corn stover	Negative	aggregation of cells	3.06E-06	159
Mammary gland	Corn stover	Negative	apoptosis of kidney cell lines	3.07E-06	119
Mammary gland	Corn stover	Negative	shape change of tumor cell lines	3.08E-06	80
Mammary gland	Corn stover	Negative	adhesion of immune cells	3.14E-06	170
Mammary gland	Corn stover	Negative	development of neurons	3.19E-06	366
Mammary gland	Corn stover	Negative	generation of lymphocytes	3.19E-06	74
Mammary	Corn	Negative	neovascularization	3.19E-06	84

gland	stover				
Mammary gland	Corn stover	Negative	Gastrointestinal Tract Cancer and Tumors	3.33E-06	2233
Mammary gland	Corn stover	Negative	cell death of pheochromocytoma cell lines	3.64E-06	64
Mammary gland	Corn stover	Negative	branching of cells	3.68E-06	179
Mammary gland	Corn stover	Negative	hypertrophy of tissue	3.73E-06	127
Mammary gland	Corn stover	Negative	abnormal morphology of intestine	3.73E-06	111
Mammary gland	Corn stover	Negative	cell death of liver	3.81E-06	119
Mammary gland	Corn stover	Negative	receptor-mediated endocytosis	3.89E-06	76
Mammary gland	Corn stover	Negative	formation of connective tissue cells	3.90E-06	106
Mammary gland	Corn stover	Negative	cell death of myeloid cells	4.06E-06	107
Mammary gland	Corn stover	Negative	concentration of triacylglycerol	4.16E-06	150
Mammary gland	Corn stover	Negative	adhesion of blood cells	4.28E-06	182
Mammary gland	Corn stover	Negative	quantity of thymus gland	4.36E-06	116
Mammary gland	Corn stover	Negative	morphology of pulmonary alveolus	4.47E-06	63
Mammary gland	Corn stover	Negative	cancer of cells	4.48E-06	126
Mammary gland	Corn stover	Negative	necrosis of liver	4.60E-06	118
Mammary gland	Corn stover	Negative	development of connective tissue cells	4.67E-06	111
Mammary gland	Corn stover	Negative	apoptosis of mesangial cells	4.67E-06	19
Mammary gland	Corn stover	Negative	concentration of eicosanoid	4.77E-06	61
Mammary gland	Corn stover	Negative	quantity of immunoglobulin	4.78E-06	145
Mammary gland	Corn stover	Negative	cellular infiltration of phagocytes	4.78E-06	90
Mammary gland	Corn stover	Negative	damage of nervous system	4.78E-06	112
Mammary	Corn	Negative	degeneration of central nervous system	4.78E-06	69

gland	stover				
Mammary gland	Corn stover	Negative	stress response of cells	4.80E-06	72
Mammary gland	Corn stover	Negative	formation of muscle cells	5.04E-06	91
Mammary gland	Corn stover	Negative	mass of connective tissue	5.04E-06	101
Mammary gland	Corn stover	Negative	proliferation of lymphatic system cells	5.04E-06	101
Mammary gland	Corn stover	Negative	familial dementia	5.22E-06	28
Mammary gland	Corn stover	Negative	concentration of Ca2+	5.24E-06	67
Mammary gland	Corn stover	Negative	apoptosis of macrophages	5.25E-06	57
Mammary gland	Corn stover	Negative	cell movement of carcinoma cell lines	5.48E-06	103
Mammary gland	Corn stover	Negative	synthesis of steroid	5.53E-06	127
Mammary gland	Corn stover	Negative	ion homeostasis of cells	5.65E-06	263
Mammary gland	Corn stover	Negative	migration of fibroblasts	5.82E-06	74
Mammary gland	Corn stover	Negative	invasion of tumor	5.89E-06	120
Mammary gland	Corn stover	Negative	proliferation of hematopoietic progenitor cells	5.89E-06	120
Mammary gland	Corn stover	Negative	cell death of phagocytes	5.89E-06	105
Mammary gland	Corn stover	Negative	Fibrosis	5.95E-06	252
Mammary gland	Corn stover	Negative	morphology of blood cells	5.95E-06	199
Mammary gland	Corn stover	Negative	activation of antigen presenting cells	5.95E-06	154
Mammary gland	Corn stover	Negative	neoplasia of tumor cell lines	5.95E-06	154
Mammary gland	Corn stover	Negative	immediate hypersensitivity	5.96E-06	155
Mammary gland	Corn stover	Negative	quantity of blastocyst	6.02E-06	26
Mammary gland	Corn stover	Negative	cell death of sensory neurons	6.03E-06	36
Mammary	Corn	Negative	apoptosis of phagocytes	6.13E-06	95

gland	stover				
Mammary gland	Corn stover	Negative	abdominal carcinoma	6.14E-06	2589
Mammary gland	Corn stover	Negative	metastasis of cells	6.18E-06	123
Mammary gland	Corn stover	Negative	apoptosis of T lymphocytes	6.28E-06	137
Mammary gland	Corn stover	Negative	metabolism of DNA	6.29E-06	191
Mammary gland	Corn stover	Negative	quantity of trophoblast cells	6.30E-06	25
Mammary gland	Corn stover	Negative	synthesis of terpenoid	6.31E-06	138
Mammary gland	Corn stover	Negative	cell death of skin	6.31E-06	56
Mammary gland	Corn stover	Negative	migration of vascular smooth muscle cells	6.31E-06	56
Mammary gland	Corn stover	Negative	apoptosis of hepatocytes	6.37E-06	69
Mammary gland	Corn stover	Negative	muscular hypertrophy	6.46E-06	102

Figure S1. A working hypothesis of mechanism through which quality of forage affects amino acids and energy metabolism function in dairy cows, suggesting roles that could be played by microRNAs (miRNAs). *Note*: positively-associated miRNAs (positively correlated with nitrogen efficiency and feed efficiency or negatively correlated with milk urea nitrogen content) and differentially expressed (DE, AL vs RS or (and) AL vs CS) in cows fed AL is indicated by (+) while negative-associated miRNAs (negatively associated with nitrogen efficiency and efficiency or positively associated with milk urea nitrogen content) and differentially expressed (DE, AL vs RS or (and) AL vs CS) in cows fed AL is indicated by (-). The molecular flows are indicated by slender arrows. Bolded solid arrows indicate increase (up) and decrease (down) for either molecules quantity or expression of miRNA. Broken line indicates certain metabolites regulate biological process of molecules. Abbreviations: AL= alfalfa hay diet; RS= rice straw diet; CS= corn stover diet; RDP= rumen degradable protein; AAs= amino acids; MCP= microbial crude protein; VFAs= volatile fatty acids; RUP= rumen undegradable protein.

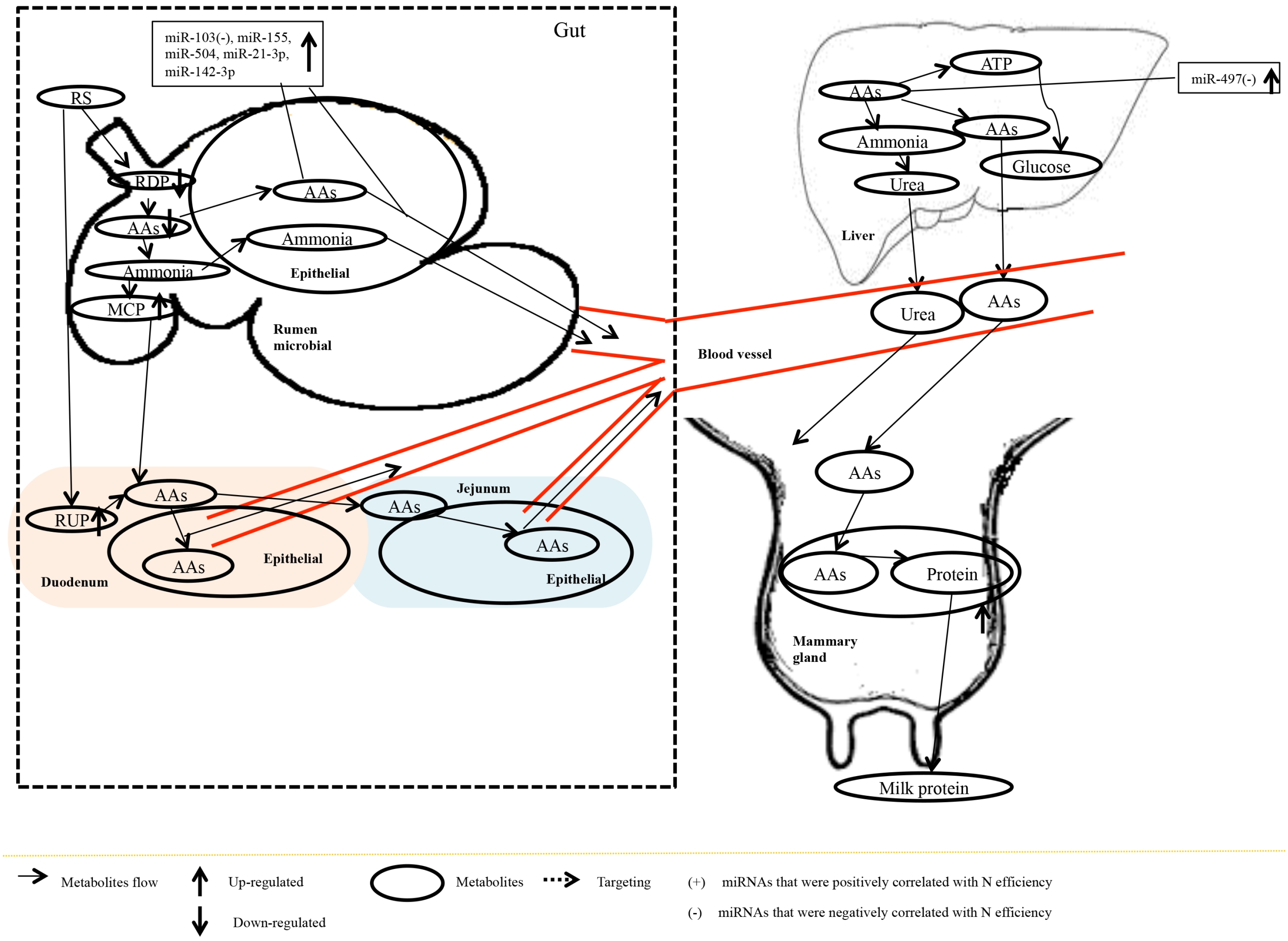
Gut
miR-103(-), miR-155, miR-504, miR-21-3p, miR-142-3p
RS
RDP
AAs
Ammonia
MCP
AAs
Ammonia
Epithelial
Rumen microbial
RUP
AAs
AAs
Epithelial
Duodenum
AAs
Jejunum
AAs
Epithelial
Blood vessel
ATP
miR-497(-)
AAs
AAs
Ammonia
Glucose
Urea
Liver
Urea
AAs
AAs
AAs
Protein
Mammary gland
Milk protein
Metabolites flow
Up-regulated
Down-regulated
Metabolites
Targeting
(+) miRNAs that were positively correlated with N efficiency
(-) miRNAs that were negatively correlated with N efficiency

Figure S2. A working hypothesis of mechanism through which quality of forage affects amino acids and energy metabolism function in dairy cows, suggesting roles that could be played by microRNAs (miRNAs). *Note*: positively-associated miRNAs (positively correlated with nitrogen efficiency and feed efficiency or negatively correlated with milk urea nitrogen content) and differentially expressed (DE, AL vs RS or (and) AL vs CS) in cows fed AL is indicated by (+) while negative-associated miRNAs (negatively associated with nitrogen efficiency and efficiency or positively associated with milk urea nitrogen content) and differentially expressed (DE, AL vs RS or (and) AL vs CS) in cows fed AL is indicated by (-). The molecular flows are indicated by slender arrows. Bolded solid arrows indicate increase (up) and decrease (down) for either molecules quantity or expression of miRNA. Broken line indicates certain metabolites regulate biological process of molecules. Abbreviations: AL= alfalfa hay diet; RS= rice straw diet; CS= corn stover diet; RDP= rumen degradable protein; AAs= amino acids; MCP= microbial crude protein; VFAs= volatile fatty acids; RUP= rumen undegradable protein.

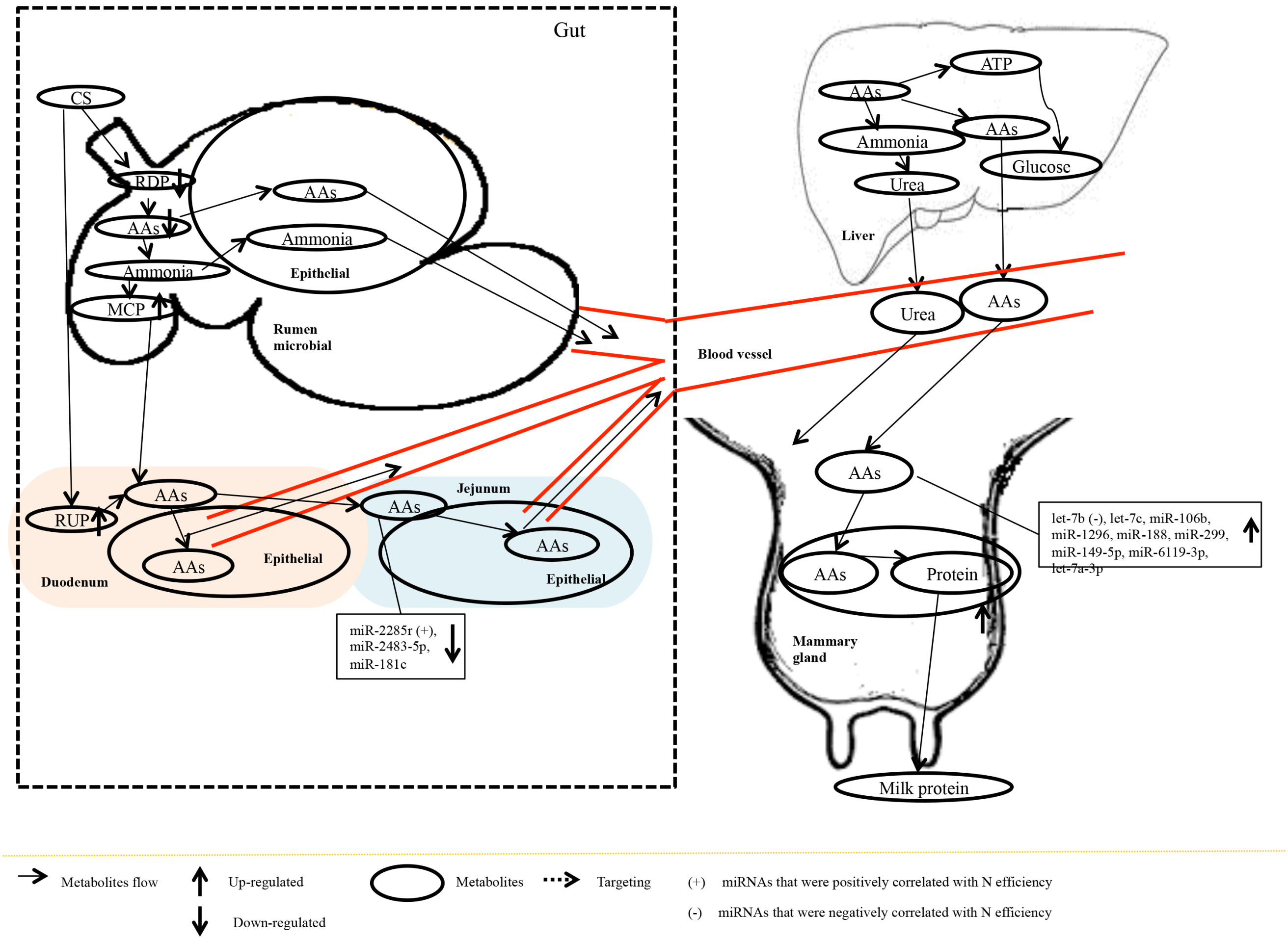
Gut
CS
RDP
AAs
Ammonia
MCP
AAs
Ammonia
Epithelial
Rumen microbial
RUP
AAs
AAs
Epithelial
Duodenum
AAs
Jejunum
AAs
Epithelial
miR-2285r (+), miR-2483-5p, miR-181c
Blood vessel
ATP
AAs
AAs
Ammonia
Glucose
Urea
Liver
Urea
AAs
AAs
AAs
Protein
let-7b (-), let-7c, miR-106b, miR-1296, miR-188, miR-299, miR-149-5p, miR-6119-3p, let-7a-3p
Mammary gland
Milk protein
Metabolites flow
Up-regulated
Down-regulated
Metabolites
Targeting
(+) miRNAs that were positively correlated with N efficiency
(-) miRNAs that were negatively correlated with N efficiency
